# Synthesis of Thioxanthone 10,10-Dioxides and Sulfone-Fluoresceins via Pd-Catalyzed Sulfonylative Homocoupling

**DOI:** 10.1021/acs.orglett.3c04300

**Published:** 2024-01-18

**Authors:** Gergely Knorr, Mariano L. Bossi, Alexey N. Butkevich, Stefan W. Hell

**Affiliations:** † Department of Optical Nanoscopy, Max Planck Institute for Medical Research, Jahnstraße 29, 69120 Heidelberg, Germany; ‡ Department of NanoBiophotonics, Max Planck Institute for Multidisciplinary Sciences, Am Faßberg 11, 37077 Göttingen, Germany

## Abstract

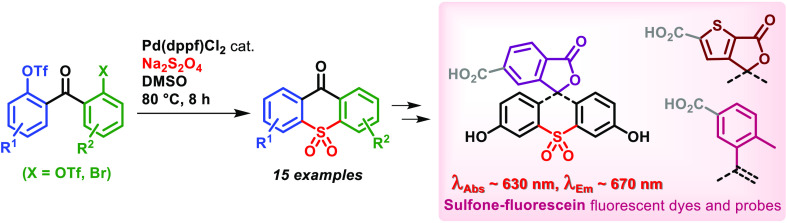

Our report describes the facile and scalable preparation of 9*H*-thioxanthen-9-one 10,10-dioxides via Pd-catalyzed sulfonylative homocoupling of the appropriately substituted benzophenones. This transformation provides a straightforward route to previously unreported sulfone-fluoresceins and -fluorones. Several examples of these red fluorescent dyes have been prepared, characterized, and evaluated as live-cell permeant labels compatible with super-resolution fluorescence microscopy with 775 nm stimulated emission depletion.

Sulfone-fluorescein (Scheme 1a; X = SO_2_) is the sulfone-bridged analogue of the long-established green-emitting fluorescent dye fluorescein (3′,6′-dihydroxyfluoran). The introduction of a strongly electron-deficient bridging group has been previously demonstrated to shift the absorption and emission maxima, mainly by decreasing the LUMO energy level of the fluorophore,^1a^ into the red range (>600 nm) preferred for a greater light penetration depth and lower phototoxicity. Unlike the spirolactone variants with a modified xanthene core (thiofluorescein,^2a^ carbofluorescein,^2b^ Si-fluorescein,^2c^ and their halogenated versions^2d^), only the derivatives with a 2-alkyl- or 2-alkoxyphenyl pendant ring have been reported for bora-fluorescein^2e^ and phospha-fluorescein,^1^ while sulfone-bridged fluorone^3^ analogues of fluorescein remain unknown outside of the patent literature.^4^

**Scheme 1:**
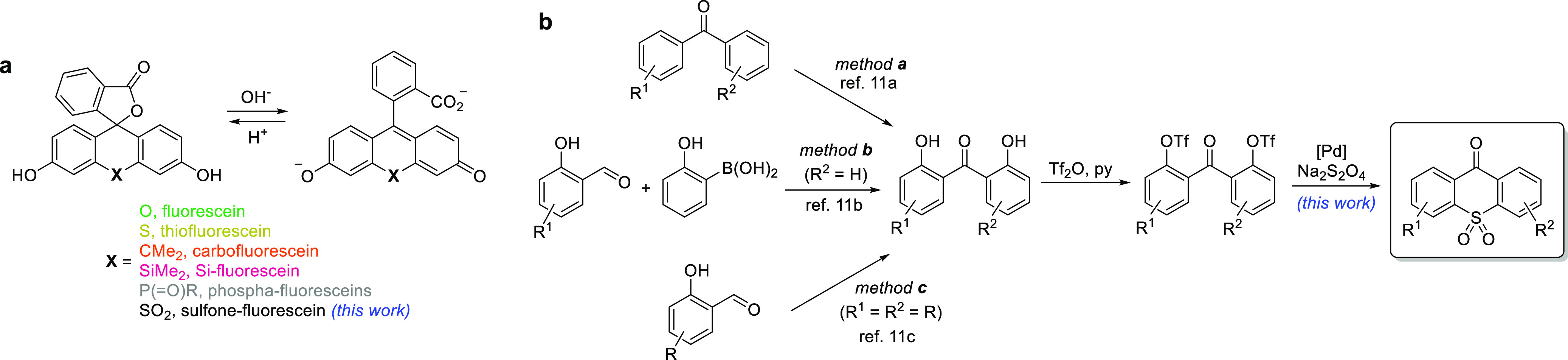
(a) Fluorescein Analogues with a Xanthene Ring Modified by Varying Bridging Groups and (b) Modular Synthetic Approach to Thioxanthone 10,10-Dioxides Method a: PhI(OAc)_2_ (2 equiv), [Ru(*p*-cymene)Cl_2_]_2_ (2.5 mol %), TFAA, TFA, 80 °C, 16 h. Method b: Cu(OAc)_2_ (2 equiv), [Cp*RhCl_2_]_2_ (4 mol %), DMF, 80 °C, 18–21 h. Method c: Cu(OAc)_2_ (2 equiv), Rh(CO)_2_(acac) (5 mol %), Na_2_CO_3_ (2 equiv), DMF, 120 °C, 24 h.

A concise synthetic strategy for accessing sulfone-fluoresceins would involve nucleophilic addition of aryllithium or arylmagnesium reagents to the keto group of appropriately substituted 9*H*-thioxanthen-9-one 10,10-dioxides. These heterocycles have found use in organic electronics as acceptor units^5^ in building blocks for thermally activated delayed fluorescence emitters, in particular in challenging recent applications such as phosphorescent^6^ and circularly polarized OLEDs.^7^

Thioxanthone 10,10-dioxides have previously been prepared through nucleophilic^8^ or electrophilic^9^ ring closure of diaryl sulfones obtained in a multistep sequence or, by far most commonly, via oxidation of preassembled thioxanthones,^7,10^ thus limiting the scope of compatible functional groups. Instead, we envisaged an alternative synthetic approach involving a Pd-catalyzed sulfonylative homocoupling of benzophenones bearing the leaving groups at positions 2 and 2′, readily accessible via established methods^11^ (Scheme 1b). For this, we were inspired by the recent reports of Wu^12a^ and Wang and Jiang^12b^ relying on sodium dithionite as a masked “SO_2_^2–^” synthone in their preparation of alkyl aryl sulfones, because the reported systems employing other SO_2_ surrogates^13^ (K_2_S_2_O_5_, DABSO, or formamidinesulfinic acid) consistently failed when tested with our substrates.

Gratifyingly, after a brief investigation of the reaction conditions (Table S1), we determined that the target thioxanthone 10,10-dioxides formed in good yields [>70% for many examples (see Scheme 2)] with an air-stable and inexpensive Pd(II) catalyst Pd(dppf)Cl_2_ when DMSO was used as the reaction solvent with mild heating (80 °C). Distinct from the reported conditions,^12a^ no addition of an external base or quaternary ammonium salts was needed. At least 1.5 equiv of Na_2_S_2_O_4_ was required to achieve complete conversion of the starting material, but a larger excess of dithionite was well tolerated and found necessary in certain cases. Decreasing the Pd catalyst loading resulted in substantially lower reaction yields. The reaction was sensitive to the nature of the solvent, and even though other dipolar aprotic solvents (in particular DMF) were suitable, the reproducibility suffered because of the significant induction time as determined by means of *in situ* IR spectroscopy (see Figure S1). The substrates bearing strong electron-withdrawing groups yielded xanthones (e.g., **2m′** and **2o′**) as the major products, likely via competing hydrolysis of aryl triflates to phenols if the main reaction was slowed, and the presence of aryl halides (as in **1r**) other than fluorine was not tolerated. Most peculiarly, and in stark contrast with previous reports, the transformation was successful only in the intramolecular version; otherwise, alternative reactivity with the formation of mixtures containing diaryl sulfides (**2p**) or disulfides (**2q**) was noticed, while other bridged (**1t** and **1u**) or simple aryl triflates (**1v**) were unreactive.

**Scheme 2:**
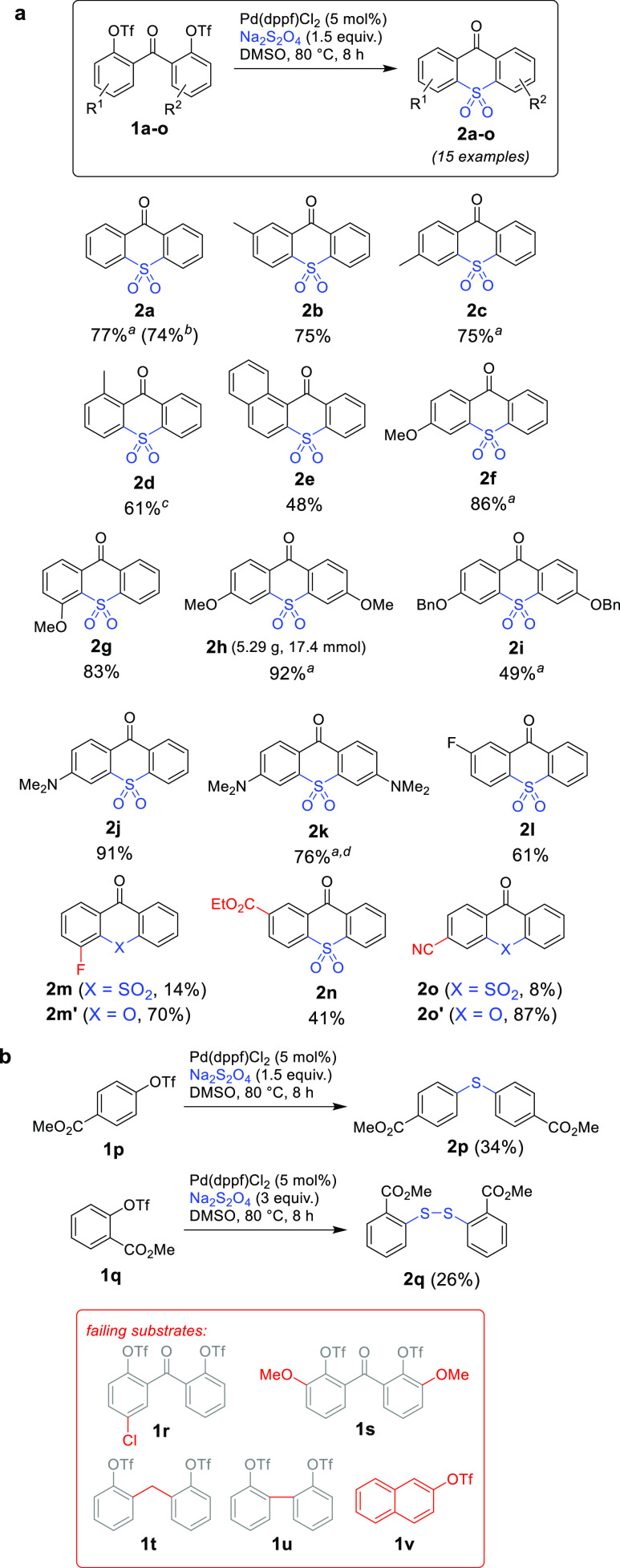
Preparation of Thioxanthone 10,10-Dioxides via Sulfonylative Homocoupling: (a) Scope of Substrates and (b) Limitations and Side Reactions With 3 equiv of Na_2_S_2_O_4_. From **1a′**. At 100 °C for 8 h. At 80 °C for 24 h.

The required presence of a coordinating 2-carbonyl group suggested the initial formation of an oxidative addition complex (i) [exemplified by **S11** (Scheme 3)], possibly stabilized via chelation, which should then undergo an insertion of SO_2_^2–^ (sulfoxylate dianion arising by disproportionation from dithionite dissociated into two sulfur dioxide radical anions^14^) generating the corresponding Pd(0)-chelated arylsulfinate intermediate (ii). In the absence of an intramolecular electrophile (i.e., in the case of monotriflate **1u**), the intermediate complex dissociates to form the free sulfinate product (iii). This product was trapped by alkylation with an excess of CH_3_I, leading to aryl methyl sulfone **2u** in 58% yield. Otherwise, the second (intramolecular) oxidative addition of an aryl triflate (for **1a**) or bromide^15^ (for **1a′**) leads to the intermediate (iv), which then undergoes reductive elimination of **2a**, regenerating the active catalyst. In a control experiment with 2-bromobenzophenone, it was confirmed that only aryl triflates underwent the initial oxidative addition under these reaction conditions, as no formation of **2u** was detected upon addition of excess CH_3_I electrophile.

**Scheme 3:**
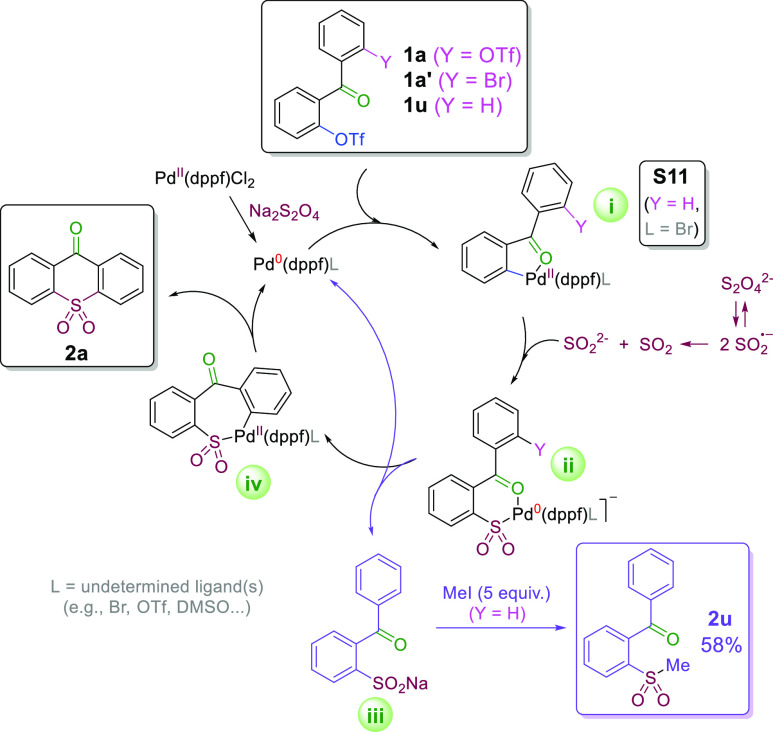
Proposed Reaction Mechanism

The robustness of the proposed method allowed us to prepare **3** (Scheme 4), the key building block for the synthesis of sulfone-fluoresceins, on a multigram scale by demethylation of **2h** in a three-step sequence starting from the commercial ultraviolet absorber benzophenone-6 (2,2′-dihydroxy-4,4′-dimethoxybenzophenone), in high yield and purity without chromatographic separation. While several protecting groups^2b,16^ were evaluated for acidic phenol groups of **3**, TBS-protected compound **4a** was the substrate of choice for the preparation of unsubstituted sulfone-fluoresceins **5a** and **5b** and sulfone-fluorone **5c** (Scheme 4a). The photophysical properties of these fluorophores are compiled in Table 1 and Figures S2 and S3.

**Table 1:** Properties of Fluorophores **5a**–**5c** and HaloTag Ligands **7a-Halo**–**7c-Halo**^a^

dye	λ_max_^abs^ (nm) [ε (M^–1^ cm^–1^)]	λ_max_^em^ (nm) (Φ_fl_^b^)	τ^c^ (ns)	p*K*_a_
**5a**	625 (3000)	663 (0.11)	1.35	8.1
**5b**	631 (42000)	670 (0.08)	1.12	5.7
**5c**	629 (25000)	667 (0.11)	1.31	5.3
**7a-Halo**	630 (13400)	670 (0.11)	1.35	7.8
**7b-Halo**	636 (58000)	677 (0.08)	1.02	5.3
**7c-Halo**	633 (27000)	670 (0.10)	1.27	5.3

a Optical properties measured in 0.1 M phosphate buffer (pH 9.0).

b Fluorescence quantum yield.

c Fluorescence lifetime.

**Scheme 4:**
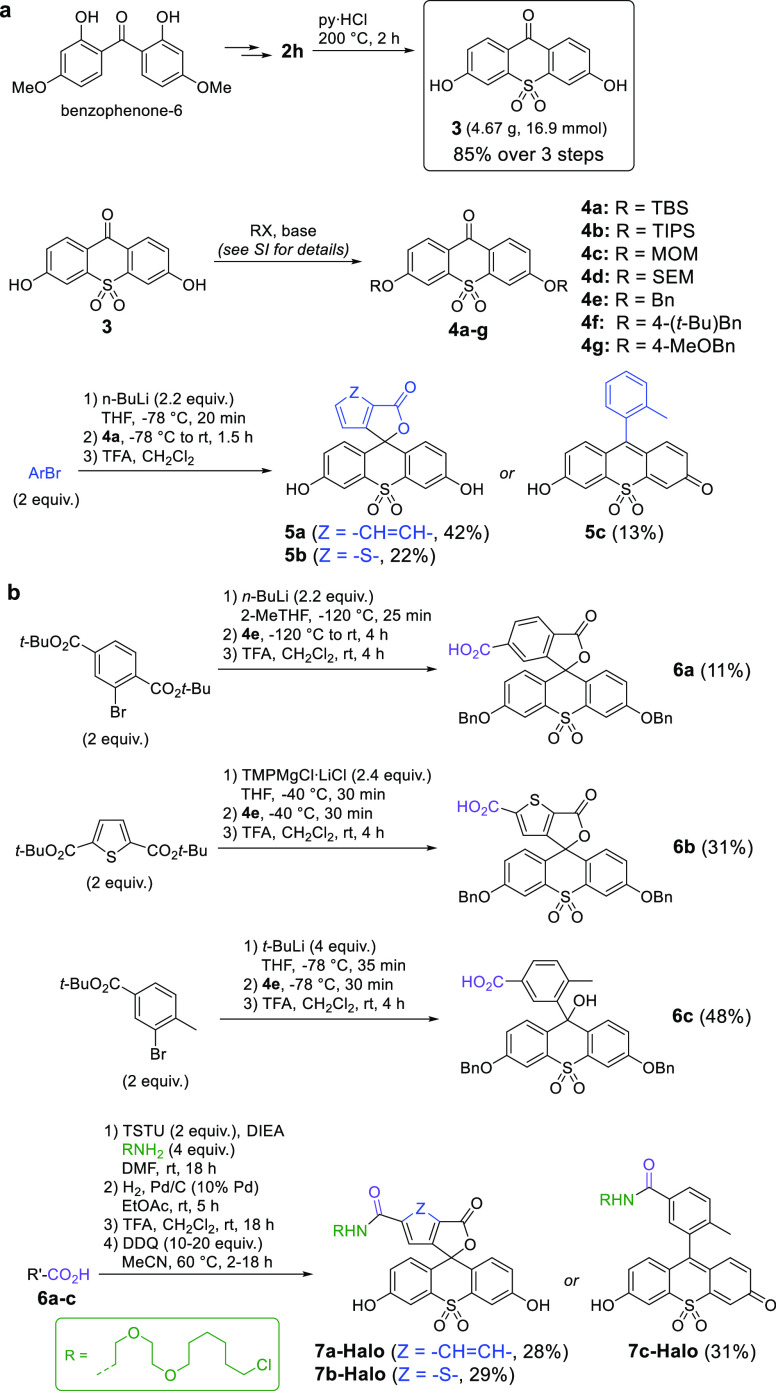
Preparation of (a) Sulfone-Fluoresceins **5a** and **5b** and Sulfone-Fluorone **5c** and (b) Fluorescent HaloTag(O2) Ligands **7a-Halo**–**7c-Halo**

Both dyes **5a** and **5b** have absorption maxima within the range of 620–630 nm (nearly optimal for excitation with a 630–640 nm pulsed laser) and are characterized by far-red emission; however, their quantum yields in aqueous buffer are modest. The fluorescent forms of sulfone-fluoresceins and sulfone-fluorones undergo protonation closing into the colorless spirolactones (for **5a**, with a p*K*_a_ of ∼8) or form triarylmethanol water adducts (for **5b** and **5c**, with corresponding p*K*_a_ values of ∼5–6); in particular, the color of the anionic form of **5c** quickly fades in solution. This electrophilic reactivity of the sulfone-fluorone core also accounts for lower than expected values of molar attenuation coefficients for sulfone-fluoresceins (ε = 3 × 10^3^ M^–1^ cm^–1^ at pH 9 for **5a** vs ε = 9 × 10^4^ M^–1^ cm^–1^ at pH 10 for fluorescein^17^).

For the preparation of the fluorophores tagged with a free carboxylic acid (suitable for conversion into targeted labels for fixed or live-cell imaging via attachment to a suitable high-affinity ligand), di-*O*-benzyl-protected **4e** was chosen for the technical ease of separation and maintained orthogonality to the *tert*-butyl ester group. As a test ligand, the bioorthogonal ω-chloroalkane/HaloTag protein system^18^ was selected for its extremely high reaction rates with triarylmethane dye-derived probes (*k*_app_ values of 1.0 × 10^6^ M^–1^ s^–1^ for fluorescein ligand^19a^ and approaching 10^8^ M^–1^ s^–1^ for certain rhodamines^19b^), leading to rapid and irreversible covalent linking within the live-cell environment. Benzyl-protected sulfone-fluorescein carboxylic acids **6a**–**6c** were coupled to the HaloTag(O2) amine, and target fluorescent probes **7a-Halo**–**7c-Halo** were liberated by hydrogenolysis followed by sequential treatment with TFA and DDQ (Scheme 4b and Figure S4).

Test labeling was performed in living U2OS-Vim-Halo cells engineered using the CRISPR-Cas technology, which were preferred for their high cell-to-cell reproducibility as opposed to transient transfection methods.^20^ HaloTag-fused vimentin (a cytoskeletal structural protein) was stained with **7a-Halo** (500 nM overnight in complete cell growth medium), followed by two washing steps (30 min each). Cells were imaged live (Figure 1) or after fixation with paraformaldehyde (Figure S5). Relatively long integration times [line or frame accumulation (see Table S2 for detailed imaging parameters)] were used to compensate for the fraction of markers in nonfluorescent spirolactone form at the cytosolic pH. The imaging results demonstrated the cell membrane permeability of **7a-Halo**, selective staining of the intermediate filaments, and good compatibility of the label with stimulated emission depletion (STED) super-resolution fluorescence imaging. The attainable resolution was limited by the low molecular brightness of fluorophore **5a** and the dynamics of intermediate filaments in living cells, despite the distinct fluorogenic response of the dye upon binding to HaloTag7 protein (Figure S6). Therefore, live-cell labeling with postfixation, permitting a longer total imaging time on immobile structures, was attempted and indeed demonstrated improved resolution (Figure S5). The samples stained with **7b-Halo** and **7c-Halo** were characterized by a significantly inferior quality of labeling, making the imaging impracticable.

**Figure 1:**
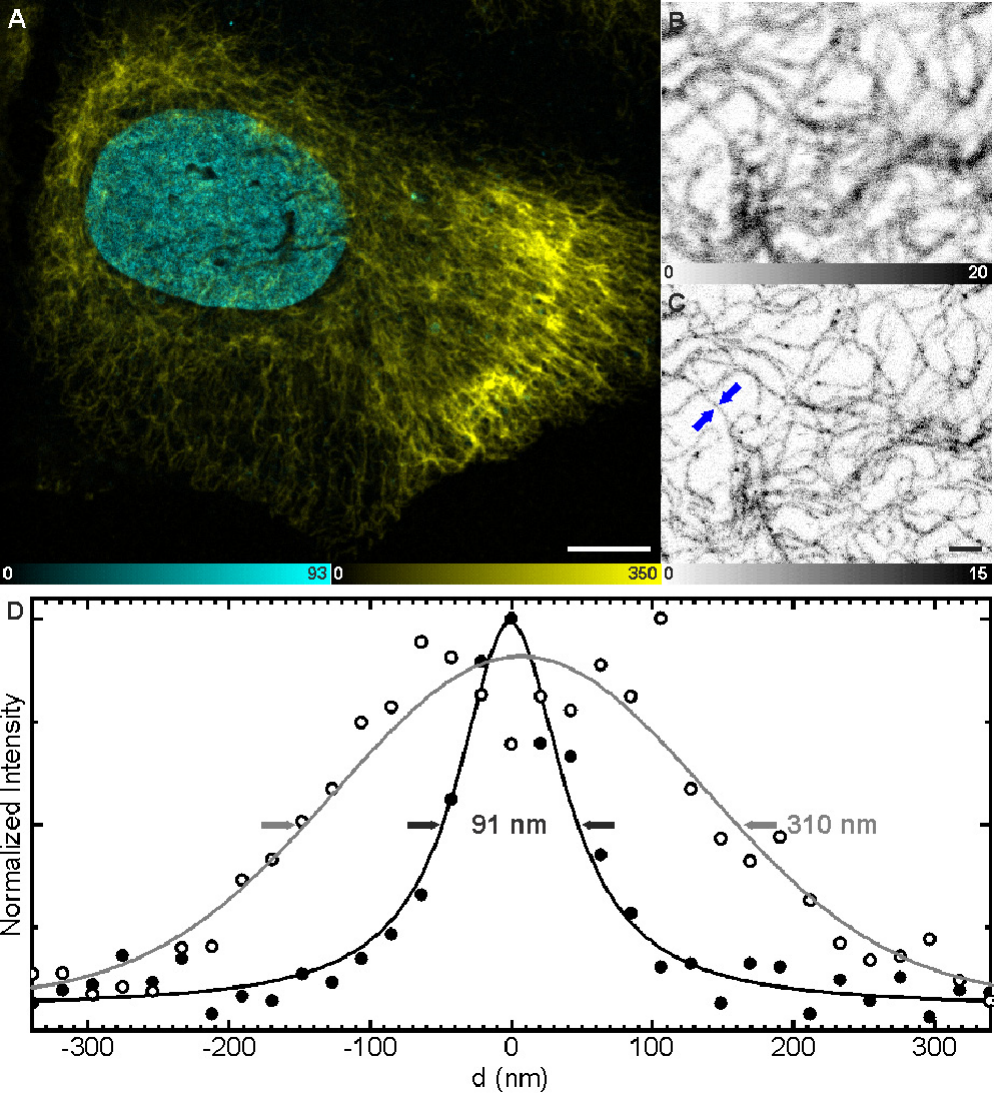
(A) Two-color overview confocal image of a living U2OS-Vim-Halo cell, labeled with probe **7a-Halo** (500 nM, overnight; yellow) and nuclear stain Hoechst 33342 (3.6 μM, 10 min; cyan). (B) Confocal and (C) STED images of labeled vimentin filaments of the same cell. (D) Line profiles (average of five pixels) across a vimentin filament indicated with blue arrows in panel C, with the corresponding fits to a Gaussian function (for the confocal image) and a Lorentzian function (for STED). The corresponding full widths at half-maximum (fwhm) are indicated, demonstrating the resolution below the diffraction limit in panel C. The scale bars are 10 μm in panel A and 1 μm in panels B and C.

In summary, we have developed an original entry in the synthesis of sulfone-fluorescein fluorophores and performed their photophysical characterization and preliminary evaluation as live-cell compatible far-red-emitting fluorescent labels. The proposed synthetic approach to thioxanthone 10,10-dioxides increases the availability of these building blocks for organophotocatalysis, as well as for photovoltaics, electroluminescence, and other material science applications. Furthermore, we are currently exploring variations of the reported reactivity toward the synthesis of other sulfone-embedded heterocycles.

## Data Availability

The data underlying this study are available in the published article and its Supporting Information.

## Supporting Information Available

Confocal time-lapse video of living U2OS cells stably expressing the vimentin-HaloTag fusion protein labeled with probe **7a-Halo** (Video S1) (AVI)
Additional experimental details, materials, methods, and characterization data for all new compounds, including Figures S1–S6 and Tables S1–S2, and NMR spectra (PDF)

## Supplementary Material

ol3c04300_si_001.avi

## Notes

Initial draft version of this work has been deposited in the preprint repository *chemRxiv*.^21^
