# Supplementary material for: Synthesis of Thioxanthone 10,10-Dioxides and Sulfone-Fluoresceins via Pd-Catalyzed Sulfonylative Homocoupling
Source: Org Lett. 2024 Jan 18;26(4):945–9. doi: 10.1021/acs.orglett.3c04300 (PMC10845149; doi:10.1021/acs.orglett.3c04300)

**Supplementary information**

# Synthesis of Thioxanthone 10,10-Dioxides and Sulfone-Fluoresceins via Pd-catalyzed Sulfonylative Homocoupling

Gergely Knorr[a], Mariano L. Bossi[a], Alexey N. Butkevich[a*] and Stefan W. Hell[a,b*]

[a] Department of Optical Nanoscopy, Max Planck Institute for Medical Research, Jahnstraße 29, 69120 Heidelberg, Germany.

[b] Department of NanoBiophotonics, Max Planck Institute for Multidisciplinary Sciences, Am Faßberg 11, 37077 Göttingen, Germany.

*Corresponding authors' e-mails: alexey.butkevich@mr.mpg.de, stefan.hell@mpinat.mpg.de.

## Table of Contents


Supplementary Video ........ 5

Video S1. Time-lapse video of the dynamics of vimentin filaments, labeled with **7a-Halo**, in a living U2OS-Vim-Halo cell. ........ 5

Supplementary Tables........ 6

Table S1. Pd-catalyzed sulfonylative homocoupling of **1a**: investigation of the reaction parameters ........ 6

Table S2. Imaging parameters for live- and fixed-cell confocal and STED images. ........ 7

Supplementary Figures ........ 8

Figure S1. *In situ* IR spectroscopy of Pd-catalyzed sulfonylative homocoupling of **1h**........ 9

Figure S2. Absorption and fluorescence emission spectra of compounds **5a-c** in 100 mM phosphate buffer at different pH values........ 10

Figure S3. pH-Dependent equilibria of sulfone-fluoresceins **5a,b** and sulfone-fluorone **5c**.... 11

Figure S4. pH-Dependent equilibria of HaloTag ligands **7a-Halo**, **7b-Halo**, **7c-Halo** ........ 12

Figure S5. Confocal image of fixed U2OS cells, stably expressing vimentin-HaloTag fusion protein, labeled with compound **7a-Halo**. ........ 13

Figure S6. Fluorogenic behavior of the probe **7a-Halo**. ........ 14

Supplementary Methods........ 15

General experimental information and synthesis........ 15

Thin layer chromatography ........ 15

Preparative flash column chromatography........ 15

High-Performance Liquid Chromatography (HPLC) and Mass Spectrometry (MS) ........ 15

NMR spectra ........ 16

Optical spectroscopy ........ 16

Fluorogenic properties of **7a-Halo** ........ 17

Cell culture and labeling ........ 17

Confocal and STED microscopy........ 18

Preparation of 2,2'-dihydroxybenzophenones ........ 19

**S1** ........ 19

**S2** ........ 19

**S3** ........ 20

**S4** ........ 21

**S5** ........ 21

**S6** ........ 22

**S7** ........ 23

**S8** ........ 23

**S9** ........ 24

**S10** ........ 25

Preparation of aryl triflates **1a'**, **1b-o** and **1r-t** ........ 25

General procedure for the preparation of aryl triflates ........ 25

**1a'** ........ 26

**1b** ........ 26

**1c** ........ 27

**1d** ........ 27

**1e** ........ 28

**1f** ........ 28

**1g** ........ 29

**1h** ........ 29

**1i** ........ 30

**1j** ........ 30

**1k** ........ 31

**1l** ........ 31

**1m** ........ 32

**1n** ........ 32

**1o** ........ 33

**1r** ........ 33

**1s** ........ 34

**1t** ........ 34

Preparation of thioxanthone 10,10-dioxides **2a-o** ........ 35

General procedure for Pd-catalyzed sulfonylative homocoupling ........ 35

**2a** ........ 35

**2b** ........ 36

**2c** ........ 37

**2d** ........ 37

**2e** ........ 38

**2f** ........ 38

**2g** ........ 39

**2h** ........ 39

**2i** ........ 40

**2j** ........ 40

**2k** ........ 41

**2l** ........ 41

**2m** and **2m'** ........ 42

**2n** ........ 42

**2o** and **2o'** ........ 43

Isolation and characterization of compounds **2p**, **2q** and **2u** ........ 44

**2p** ........ 44

**2q** ........ 44

**2u** ........ 45

Reaction of the oxidative addition complex **(i)** with sodium dithionite ........ 45

Compound **3** and its *O*-protected derivatives **4a-g** ........ 46

**3** ........ 46

**4a** ........ 47

**4b** ........ 47

**4c** ........ 48

**4d** ........ 49

**4e** ........ 49

**4f** ........ 50

**4g** ........ 50

Preparation of sulfone-fluoresceins **5a,b** and sulfone-fluorone **5c** ........ 51

**5a** ........ 51

**5b** ........ 51

**5c** ........ 52

Preparation of HaloTag ligands **7a-c** ........ 54

**6a** ........ 54

**6a-Halo** ........ 55

**7a-Halo** ........ 56

**S12** ........ 57

**6b** ........ 57

**6b-Halo** ........ 58

**7b-Halo** ........ 59

**6c *tert*-butyl ester** ........ 60

**6c** ........ 60

**6c-Halo** ........ 61

**7c-Halo** ........ 62

Supplementary references ........ 64

NMR spectra ........ 66

## Supplementary Video

**Video S1.** Time-lapse video of the dynamics of vimentin filaments, labeled with **7a-Halo**, in a living U2OS-Vim-Halo cell. Frame sequence of confocal images of a living U2OS cell, stably expressing vimentin-HaloTag construct, labelled with compound **7a-Halo** (500 nM, overnight). A total of 40 frames were acquired with a time acquisition of 0.4 seconds/frame, displayed at a framerate of 2 frames/s.

## Supplementary Tables

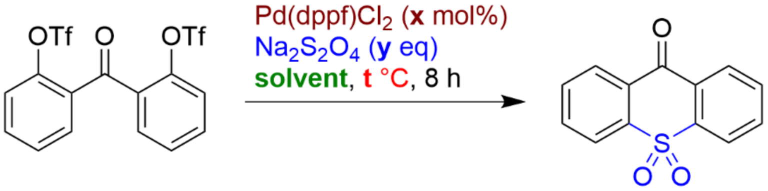


**1a** **2a**

| entry | Pd(dppf)Cl₂, mol% | $Na_2S_2O_4$, equiv. | solvent | temp, °C | conversion of **1a**, % (HPLC) | yield of **2a**, % (HPLC) |
|---|---|---|---|---|---|---|
| 1 | 5 | 0.5 | DMSO | 80 | 65 | 49 |
| 2 | 5 | 1 | DMSO | 80 | 89 | 85 |
| 3 | 5 | 2 | DMSO | 80 | 98 | 85 |
| 4 | 0.5 | 1.5 | DMSO | 80 | 53 | 0 |
| 5 | 2 | 1.5 | DMSO | 80 | 84 | 58 |
| 6 | 5 | 1.5 | 1,4-dioxane | 80 | 17 | 0 |
| 7 | 5 | 1.5 | acetonitrile | 80 | 40 | 4 |
| 8 | 5 | 1.5 | toluene | 80 | 31 | 0 |
| 9 | 5 | 1.5 | *tert*-butanol | 80 | 50 | 0 |
| 10 | 5 | 1.5 | propylene carbonate | 80 | 40 | 5 |
| 11 | 5 | 1.5 | DIEA | 80 | 82 | 0 |
| 12 | 5 | 1.5 | DMI | 80 | 78 | 64 |
| 13 | 5 | 1.5 | NMP | 80 | 83 | 64 |
| 14 | 5 | 1.5 | DMF | 80 | 95 | 92 |
| 15 | 5 | 1.5 | DMSO | 60 | 69 | 46 |
| 16 | 5 | 1.5 | DMSO | 100 | 100 | 78 |

**Table S1.** Pd-catalyzed sulfonylative homocoupling of **1a**: investigation of the reaction parameters. Solvents: DMSO – dimethyl sulfoxide, DIEA – *N*-ethyldiisopropylamine, DMI – 1,3-dimethyl-2-imidazolidinone, NMP – 1-methyl-2-pyrrolidone, DMF – *N*,*N*-dimethylformamide. HPLC conversions and yields were determined by integration of peak areas (UV-vis detection, absorption at 254 nm; see Supplementary Methods for the LC-MS system configuration) of **1a** and **2a** relative to biphenyl (0.5 equiv.) as an internal standard.

| Figure | excitation (wavelength [nm]/ power [µW])[a] | STED[b] (wavelength [nm]/ power [mW])[a] | detection window [nm] | pixel size [nm] | dwell time [µs] | line/frame accumulation | multiplexing |
|---|---|---|---|---|---|---|---|
| **1A** | 640/19 | --- | 650-763 | 70 | 20 | 2/0 | frame |
| | 405/13 | --- | 415-583 | 70 | 10 | 1/0 | frame |
| **1B** | 640/19 | --- | 650-763 | 30 | 20 | 4/0 | line |
| **1C** | 640/19 | 775/37 | 650-763 | 30 | 20 | 12/0 | line |
| **S5A** | 640/19 | --- | 650-763 | 70 | 20 | 6/0 | --- |
| **S5B** | 640/19 | --- | 650-763 | 90 | 20 | 1/16 | frame |
| **S5C** | 640/22 | 775/114 | 650-763 | 30 | 20 | 3/14 | frame |

**Table S2.** Imaging parameters for live- and fixed-cell confocal and STED images.
[a] Powers measured at the back focal plane of the objective lens; [b] Delay and gate of the STED laser were set to 750 ps and 8 ns, respectively.

**Supplementary Figures**

**a**

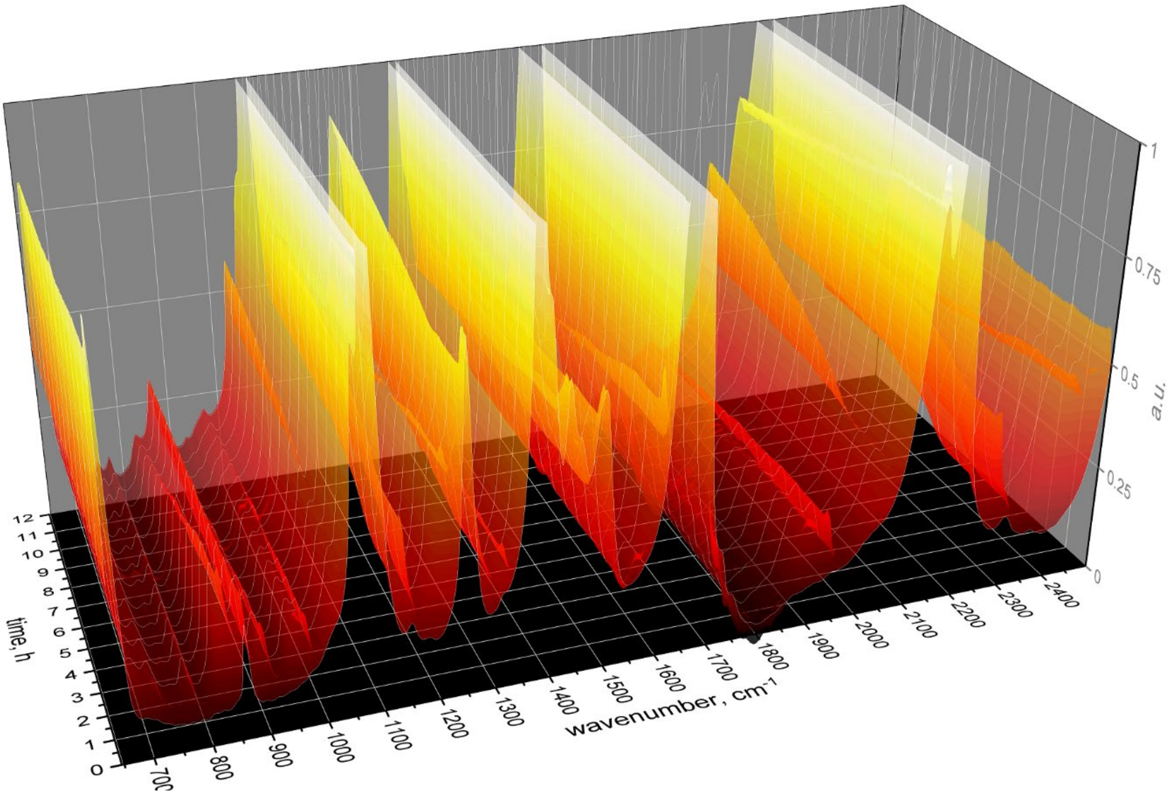


**b**

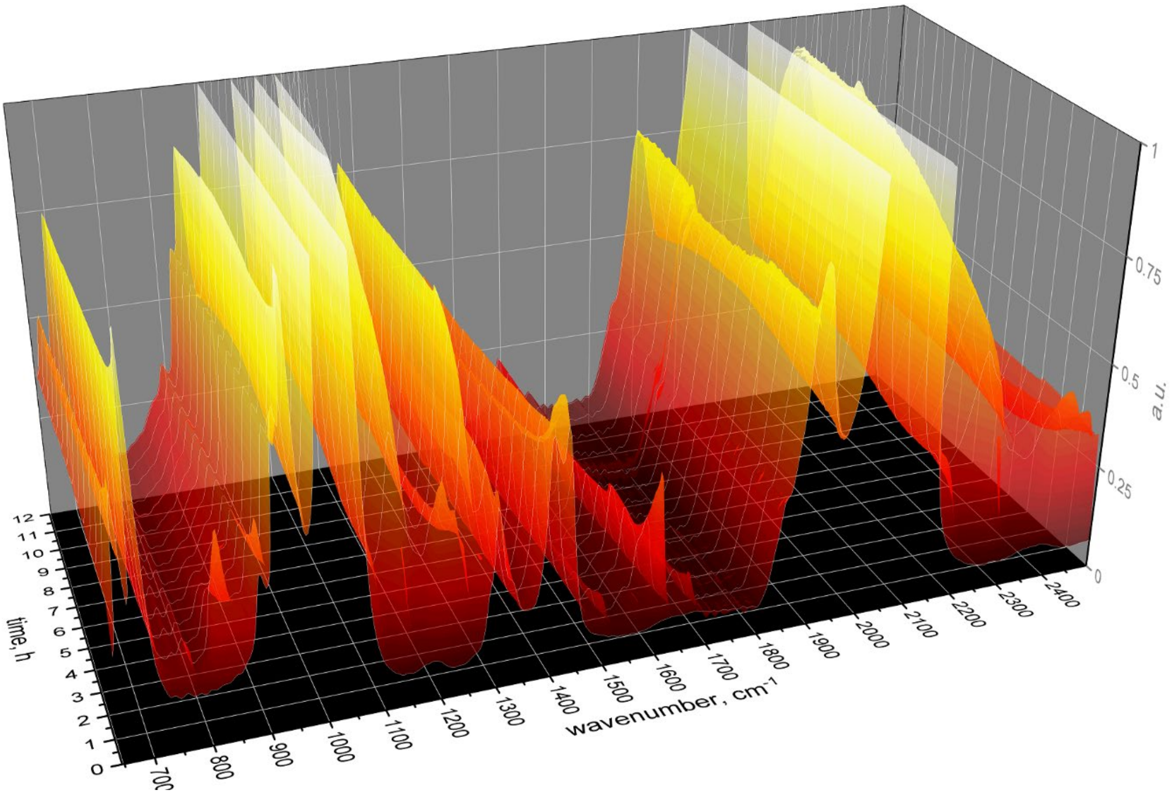


*(continued)*

**c**

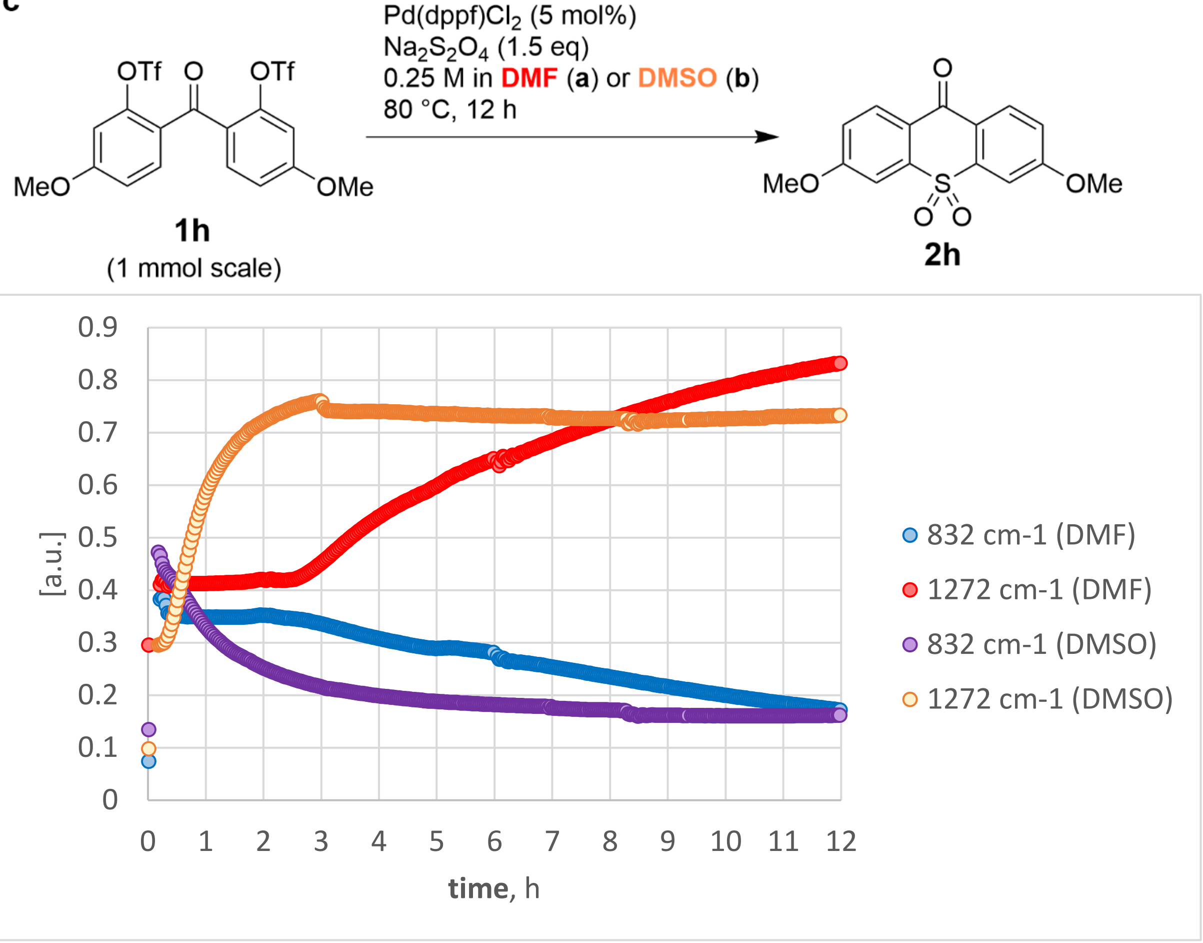


**Figure S1.** *In situ* IR spectroscopy of Pd-catalyzed sulfonylative homocoupling of **1h** in DMF (**a**) *vs* DMSO (**b**) at 80 °C over 12 h. Isolated traces at 832 $cm^{-1}$ and 1272 $cm^{-1}$ (corresponding to **1h** and **2h**, respectively) are shown in (**c**). HPLC-MS analysis of the reaction mixture from run (**a**) at 12 h showed 14:86 ratio of **1h**:**2h** (peak area at 254 nm).

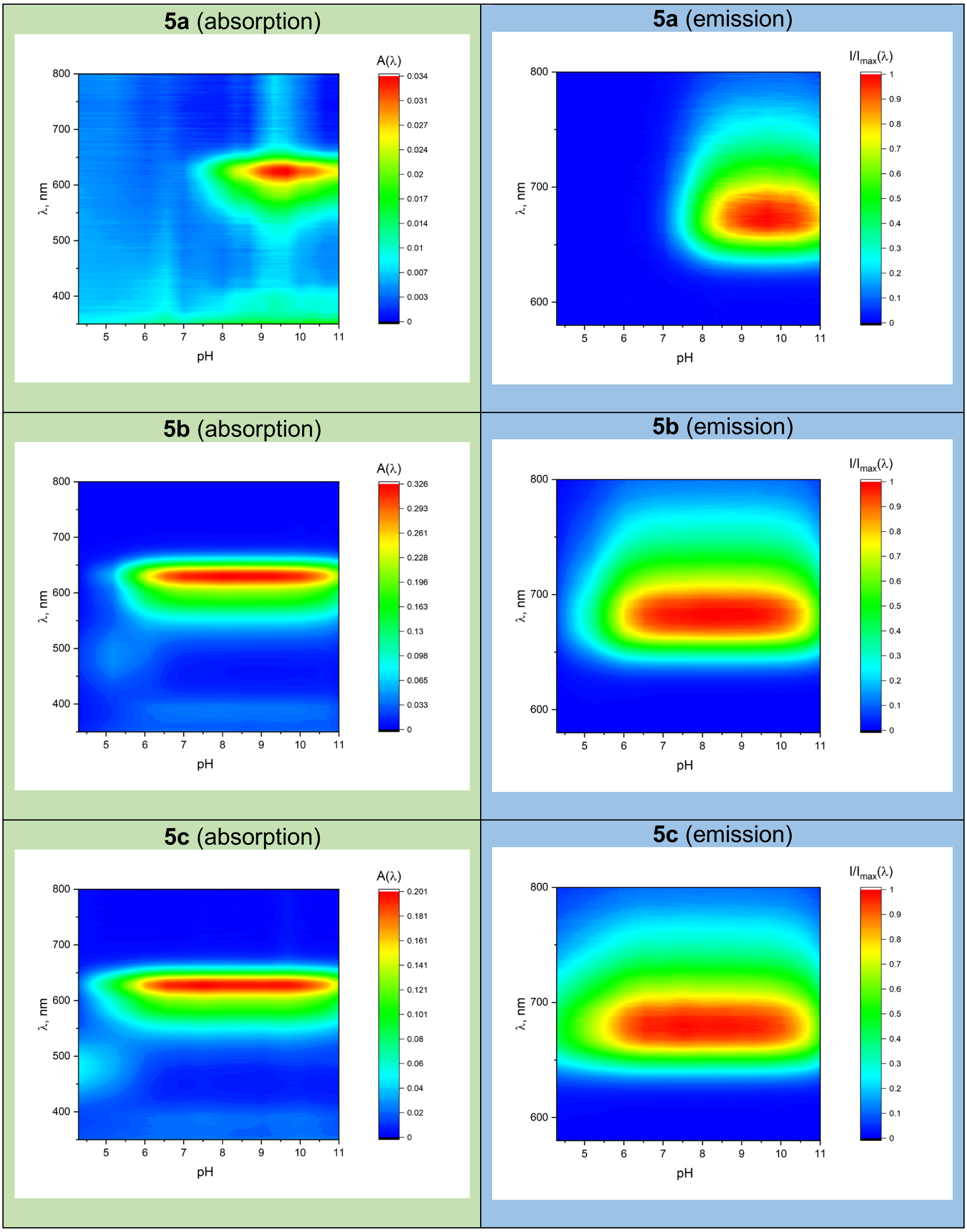


**Figure S2.** Absorption and fluorescence emission spectra of compounds **5a-c** in 100 mM phosphate buffer at different pH values (pH 4-11, +0.1% (v/v) DMSO).

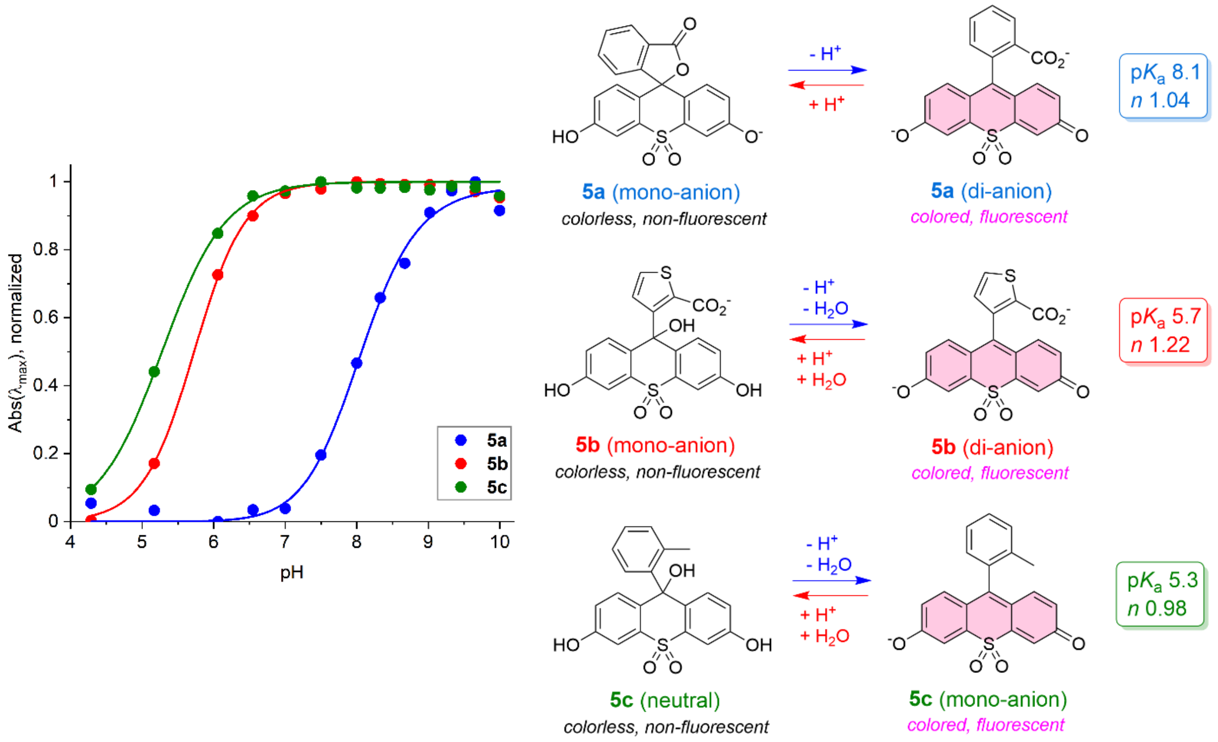


**Figure S3.** pH-Dependent equilibria of sulfone-fluoresceins **5a,b** and sulfone-fluorone **5c**. Absorption in Britton-Robinson buffer (0.04 M each of $H_3PO_4$, AcOH and $H_3BO_3$ in water [1]) at 630 nm (averaged in triplicate) was plotted against pH, normalized to the maximum value between pH 4-10 and fitted to the Hill equation using Origin 2020b software (see Supplementary Methods). *n* – fitted values of Hill's coefficient; *n* = 1 corresponds to ideal Henderson-Hasselbalch behavior for single protonation/deprotonation).

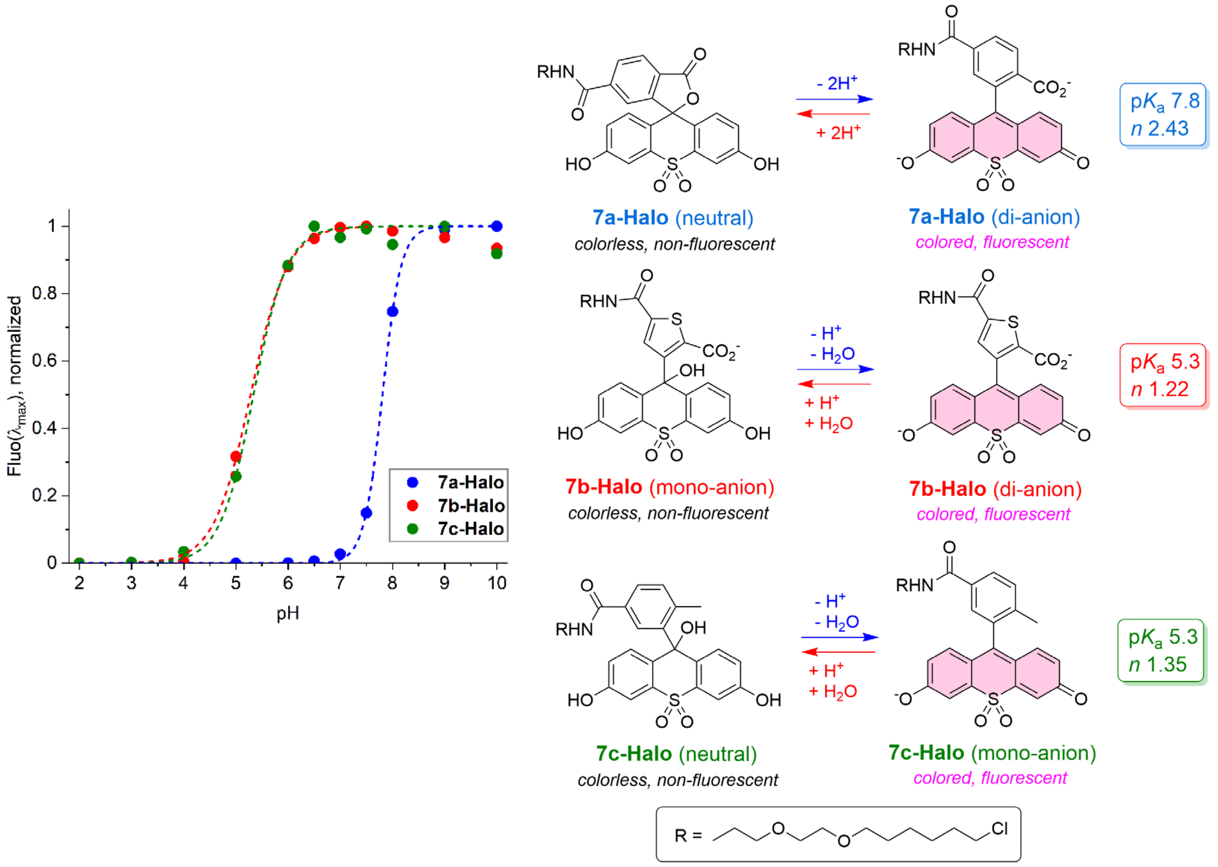


**Figure S4.** pH-Dependent equilibria of HaloTag ligands **7a-Halo**, **7b-Halo**, **7c-Halo**. Fluorescence emission in Britton-Robinson buffer (0.04 M each of $H_3PO_4$, AcOH and $H_3BO_3$ in water [1]) at 670 nm (averaged in duplicate) was plotted against pH, normalized to the maximum value between pH 2-10 and fitted to the Hill equation using Origin 2020b software (see Supplementary Methods). *n* – fitted values of Hill's coefficient; *n* = 1 corresponds to ideal Henderson-Hasselbalch behavior for single protonation/deprotonation, *n* = 2 indicates cooperative protonation).

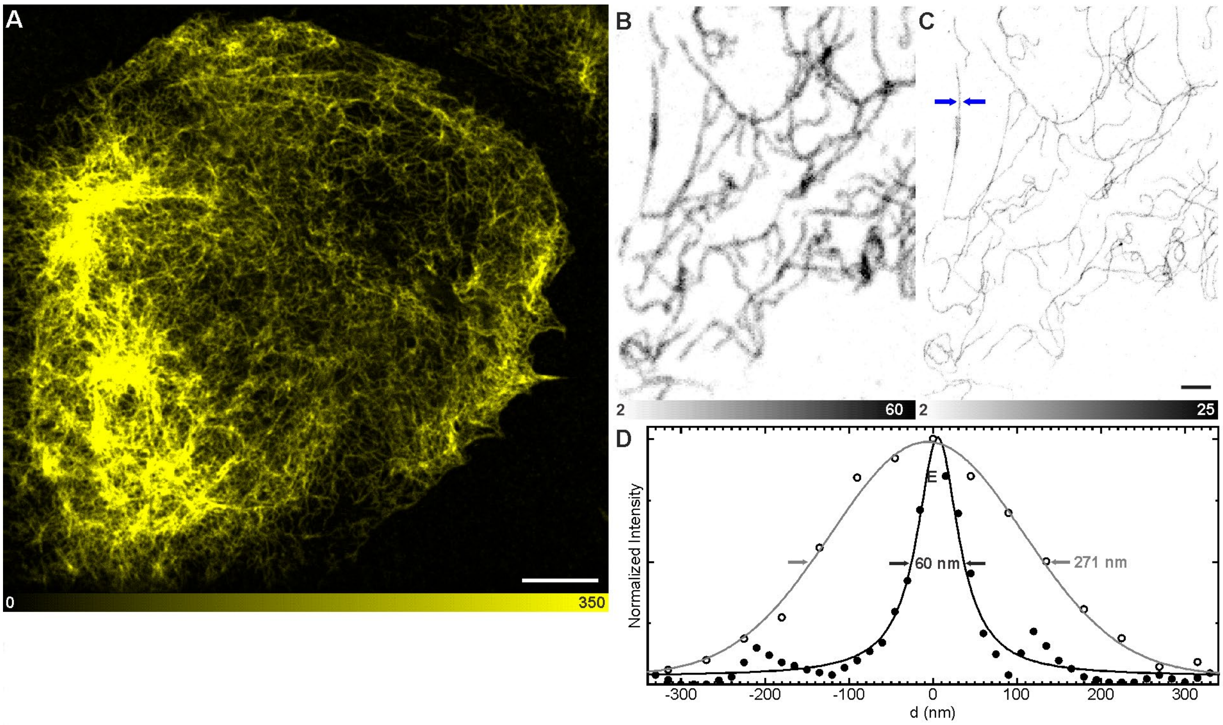


**Figure S5.** Confocal image of fixed U2OS cells, stably expressing vimentin-HaloTag fusion protein, labeled with compound **7a-Halo**. Cells were labeled live (500 nm of **7a-Halo** overnight), fixed with paraformaldehyde and mounted in Mowiol mounting medium before imaging. (A) Confocal overview image of a whole cell. Confocal (B) and STED (C) images of a region of interest from the same cell. (D) Line profiles across a vimentin filament (indicated with blue arrows in (C)), and the corresponding fits to a Gaussian (for confocal image) and a Lorentzian (for STED) function. The corresponding corresponding full width at half maximum (FWHM) values are indicated. Scale bars: 10 µm (A), 2 µm (B,C).

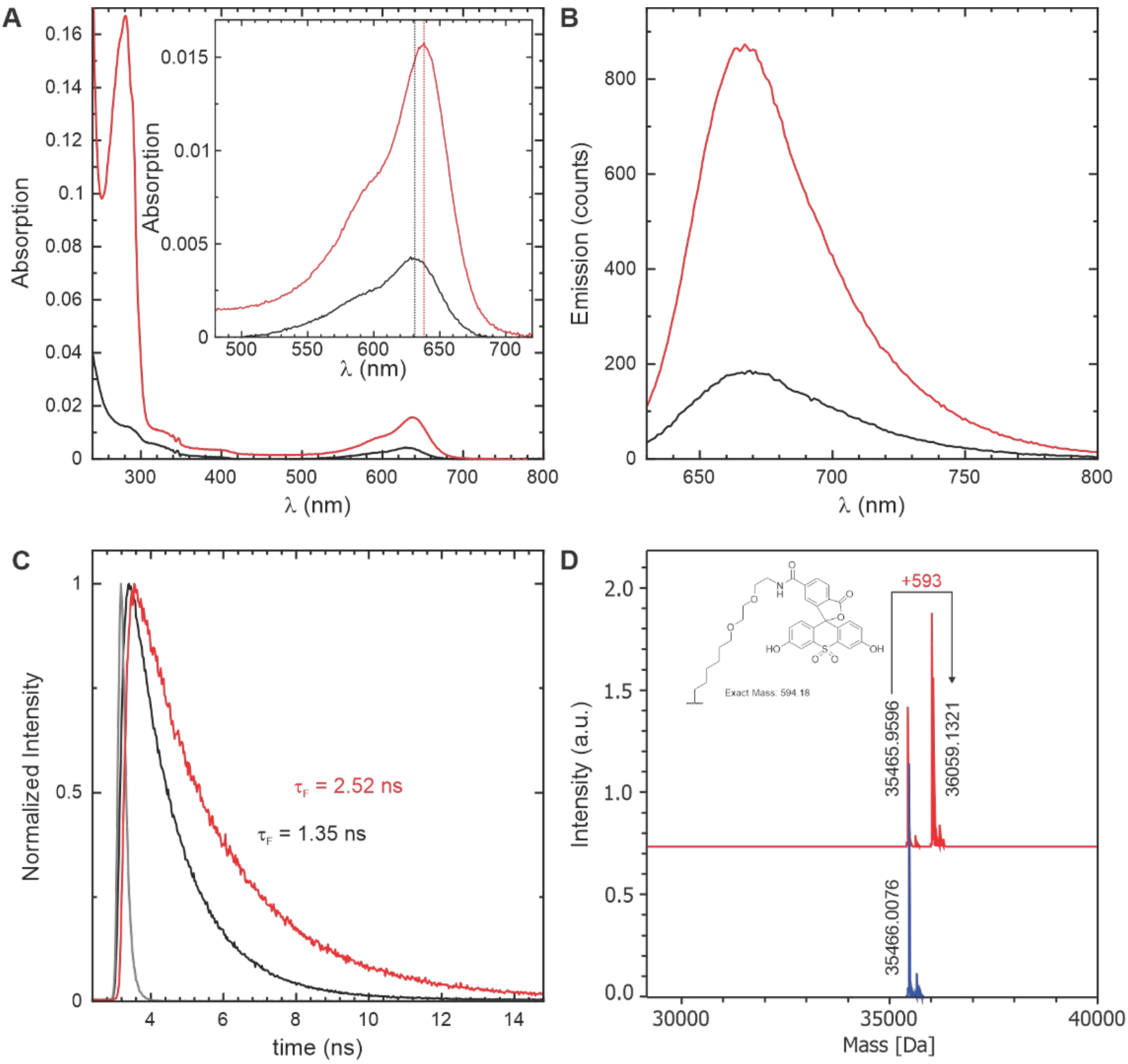


**Figure S6.** Fluorogenic behavior of the probe **7a-Halo**. Absorption (A), fluorescence emission intensity (B) and fluorescence lifetime (C) changes of the probe **7a-Halo** in 100 mM phosphate-buffered saline (pH 7.4) upon binding to the target protein HaloTag7. Black lines – free dye, red lines – after equilibration with 1.5 equiv. of the protein. (D) ESI mass spectra of HaloTag7 protein before (blue line) and after the addition of **7a-Halo** (red line).

## Supplementary Methods

### General experimental information and synthesis

Unless indicated otherwise, the reaction mixtures were heated using stirred silicone oil baths (Fisher Scientific, Cat. No. 11470673) pre-equilibrated to the indicated temperature. The reactions set up in closed volume (Ace pressure tubes, see preparations of **S1** and **S4**) were performed behind a polycarbonate blast shield (Bel-Art SP Scienceware, Cat. No. H249620000).

### Thin layer chromatography

Analytical TLC (normal phase) was performed on Merck Millipore ready-to-use aluminum sheets coated with silica gel 60 ($F_{254}$) (Cat. No. 1.05554.0001). Analytical TLC on reversed phase (RP-$C_{18}$) was performed on Merck Millipore ready-to-use aluminum sheets coated with RP-18 60 ($F_{254}$s) (Cat. No. 1.05560.0001). Compounds were detected by exposing TLC plates to UV-light (254 or 366 nm) or by heating with vanillin stain (6 g vanillin and 1.5 mL conc. $H_2SO_4$ in 100 mL ethanol), 1 N NaOH or 1 N HCl as indicated.

### Preparative flash column chromatography

Automated separations on normal phase (silica gel) were performed with an Isolera Spektra One system (Biotage AG, Sweden) using commercially available cartridges of suitable size (BGB Scorpius 40-60 µm series from BGB Analytik, Puriflash Silica HP 30µm series from Interchim) and solvent gradient indicated.

### High-Performance Liquid Chromatography (HPLC) and Mass Spectrometry (MS)

Analytical liquid chromatography-mass spectrometry was performed on an LC-MS system (Shimadzu): 2x LC-20AD HPLC pumps with DGU-20A3R solvent degassing unit, SIL-20ACHT autosampler, CTO-20AC column oven, SPD-M30A diode array detector and CBM-20A communication bus module, integrated with CAMAG TLC-MS interface 2, FCV-20A$H_2$ diverter valve and LCMS-2020 spectrometer with electrospray ionization (ESI, 100 – 1500 m/z). Analytical column: Hypersil GOLD 50×2.1 mm 1.9µm, standard conditions: sample volume 1-2 µL, solvent flow rate 0.5 mL/min, column temperature 30 °C. General method: isocratic 90:10 A:B over 2 min, then gradient 90:10 to 1:99 A:B over 5 min, then isocratic 1:99 A:B over 2 min; solvent A – water + 0.1% (v/v) $HCO_2H$, solvent B – acetonitrile + 0.1% (v/v) $HCO_2H$.

High resolution mass spectra (HRMS) were obtained on a maXis II ETD (Bruker) with electrospray ionization (ESI) and quadruple time-of-flight (QTOF) mass analyzer at the Mass Spectrometry Core facility of the Max-Planck Institute for Medical Research (Heidelberg, Germany).

Preparative high-performance liquid chromatography was performed on a Büchi Reveleris Prep system using the suitable preparative columns and conditions as indicated for individual preparations. Method scouting was performed on a HPLC system (Shimadzu): 2x LC-20AD HPLC pumps with DGU-20A3R solvent degassing unit, CTO-20AC column oven equipped with a manual injector with a 20 µL sample loop, SPD-M20A diode array detector, RF-20A fluorescence detector and CBM-20A communication bus module; or on a Dionex Ultimate 3000 UPLC system: LPG-3400SD pump, WPS-3000SL autosampler, TCC-3000SD column compartment with 2× 7-port 6-position valves and DAD-3000RS diode array detector. The test runs were performed on analytical columns with matching phases (HPLC: Interchim 250×4.6 mm 10 µm C18HQ, Interchim 250×4.6 mm 5 µm PhC4, solvent flow rate 1.2 mL/min; UPLC: Interchim C18HQ or PhC4 75×2.1 mm 2.2 µm, ThermoFisher Hypersil GOLD 100×2.1 mm 1.9 µm, solvent flow rate 0.5 mL/min).

**NMR spectra**

NMR spectra were recorded at 25 °C with a Bruker Ascend 400 spectrometer at 400.15 MHz ($^1$H), 376.52 MHz ($^{19}$F) and 100.62 MHz ($^{13}$C) and are reported in ppm. All $^1$H spectra are referenced to tetramethylsilane as an internal standard (δ = 0.00 ppm). $^{13}$C spectra are referenced to tetramethylsilane (*δ* = 0 ppm) using the signals of the solvent: $CDCl_3$ (77.16 ppm), $\underline{C}D_3CN$ (1.32 ppm) or DMSO-$d_6$ (39.52 ppm). Multiplicities of the signals are described as follows: s = singlet, d = doublet, t = triplet, q = quartet, m = multiplet or overlap of non-equivalent resonances; br = broad signal. Coupling constants $^nJ_{X\text{-}Y}$ are given in Hz, where *n* is the number of bonds between the coupled nuclei X and Y ($J_{H\text{-}H}$ are always listed as *J* without indices).

**Optical spectroscopy**

Absorption spectra were recorded with a Varian Cary 4000 UV-Vis double-beam spectrophotometer (Agilent Technologies, USA). The emission spectra were recorded with a Varian Cary Eclipse fluorescence spectrophotometer (Agilent). The spectra were recorded in quartz cuvettes (light path 10 mm; Hellma Analytics, cat. # 119-10-40). For the data shown in Figure S2, absorption and fluorescence emission spectra were

recorded in triplicate with a CLARIOstar Plus microplate reader (BMG LABTECH GmbH, Germany) in 96-well microplates (200 µL/well): non-binding polystyrene F-bottom, µClear (Greiner Bio-One GmbH, Ref. 655906). All spectra were recorded at 25 °C in air-saturated solvents and are background corrected.

$pK_a$ values (apparent) were determined from the absorbance data measured at the longest wavelength absorption maxima $A(\lambda_{max})$ within the visible range (400…700 nm) in the presence of 0.1 (v/v) DMSO and fitted to the following equation using Origin 2020b software:

$$A(\lambda_{max}) = \frac{1}{1 + 10^{n(pK_a - pH)}}$$

Fluorescence quantum yields (absolute method determinations) were obtained with a Quantaurus-QY absolute PL quantum yield spectrometer (model C11347-11, Hamamatsu) according to the manufacturer's instructions. Fluorescence lifetimes were measured with a FluoTime 300 fluorescence lifetime spectrometer (PicoQuant, controlled with the EasyTau1.4 software). All measurements were performed in air-saturated solvents at ambient temperature.

### Fluorogenic properties of 7a-Halo

To investigate the fluorogenicity of the label **7a-Halo** upon binding to its target protein, the photophysical properties were measured in bulk solution of the free dye (1.67 µM in PBS) and after the reaction with 1.5 equiv. (2.49 µM) of HaloTag7. The absorption, emission and fluorescence lifetime were measured. To ensure complete reaction, the emission intensity was measured every 90 s after the addition until no further changes were observed. Upon binding, the absorption is slightly red-shifted by 7 nm (from 631 to 638 nm) and increases 3.8-fold (Figure S6, A). The emission intensity increases 4.2-fold (total integrated intensity from Figure S6, B) and the fluorescence lifetime 1.87-fold (from 1.35 ns to 2.52 ns; Figure S6, C).

### Cell culture and labeling

U2OS-Vim-Halo cells [2] were cultured in DMEM (Dulbecco's modified Eagle medium), high glucose, GlutaMAX Supplement, pyruvate (ThermoFisher, cat. # 10569010), supplemented with 10% (v/v) fetal bovine serum (FBS, ThermoFisher cat. # 10500064) and 1% penicillin-streptomycin (ThermoFisher, cat. # 15140122) in a humidified 5% $CO_2$ incubator at 37 °C. Cells were split at 80-90% confluency and regularly tested for

mycoplasma contamination. Prior to labelling, cells were grown for 48 h on glass coverslips. Then, a stock solution of **7a-Halo** in DMSO (500 μM) was diluted in the cell medium to a final concentration of 500 nM. Cells were incubated overnight with this solution and then the medium was exchanged with free medium (without the dye) and incubated for 30 minutes (this washing process was repeated one more time).

**Live-cell imaging.** For cells co-stained with Hoechst 33342 (ThermoFisher, cat. # H3570), a 10 mg/mL solution in DMSO was diluted in the cell medium to a final concentration of 2 μg/mL (ca. 3.6 μM), incubated for 10 minutes in this solution and then washed in dye-free medium for another 10 minutes. Then, the coverslips with labeled cells were transferred to a live-cell magnetic chamber (Live Cell Instrument Co. Ltd., cat. # CM-B18-1) and filled with 1 mL of FluoroBrite DMEM medium (ThermoFisher, cat. # A1896701) supplemented with 10% (v/v) FBS and GlutaMAX (2%), and imaged for ca. 1 h under these conditions.

**Fixed-cell imaging.** Cells were rinsed with warm 100 mM phosphate buffered saline (PBS, pH 7.4) and fixed with paraformaldehyde (PFA, 4%) for 20 minutes at rt, rinsed with PBS again and treated with a quenching solution (0.1 M $NH_4Cl$ and 0.1 M glycine in PBS) for 10 min at rt. The samples were then washed with PBS (2× 5 min at rt), mounted in Mowiol mounting medium (without additives) and imaged after ca. 3 h.

**Confocal and STED microscopy**

Imaging was performed with an Abberior expert line microscope (Abberior Instruments GmbH) built on a motorized inverted microscope IX83 (Olympus). The microscope is equipped with 405 nm excitation CW laser, a 640 nm excitation pulsed (40 MHz) laser, and a 775 nm pulsed (40 MHz) STED laser shaped by a Spatial Light Modulator. Spectral detection was performed with avalanche photodiodes (APD) in the indicated spectral windows. Images were acquired with a 100x/1.40 UPlanSApo Oil immersion objective lens (Olympus), and the pinhole set to 100 μm (1 Airy unit). A *z*-focus drift compensation unit was used to minimized axial drift during measurement. The imaging parameters for live- and fixed-cell confocal and STED images presented in this work are summarized in Table S2.

## Preparation of 2,2'-dihydroxybenzophenones

**S1**

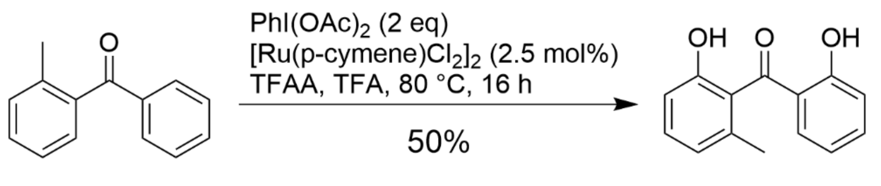


**S1**

The procedure from [3] was followed. In a 100 mL Ace pressure tube (back seal, capacity 100 mL, length 17.8 cm, diameter 38.1 mm), (diacetoxyiodo)benzene (2.58 g, 8 mmol, 2 eq) and dichloro(*p*-cymene)ruthenium(II) dimer (61 mg, 0.10 mmol, 2.5 mol%) were added to the solution of 2-methylbenzophenone (784 mg, 4 mmol) in the mixture of trifluoroacetic acid (16 mL) and trifluoroacetic anhydride (8 mL), and the tube was sealed. The reaction mixture was stirred at 80 °C for 16 h (behind a safety shield), cooled down to rt and evaporated to brown oil, which was wet-loaded with minimal amount of $CH_2Cl_2$ on top of a primed 40 g BGB Scorpius 50 μm silica flash cartridge. The crude product was isolated by flash column chromatography (gradient 5% to 60% EtOAc/hexane) and repurified by second flash column chromatography (25 g Interchim SiHP 30 μm cartridge, gradient 50% to 100 % $CH_2Cl_2$/hexane followed by 0% to 10 % EtOAc/$CH_2Cl_2$). Viscous yellow oil, yield 453 mg (50%).

$^1$H NMR (400 MHz, $CDCl_3$): δ 12.03 (s, 1H), 7.49 (ddd, J = 8.5, 7.1, 1.7 Hz, 1H), 7.33 (ddd, J = 7.9, 1.7, 0.4 Hz, 1H), 7.24 (t, J = 7.9 Hz, 1H), 7.05 (ddd, J = 8.5, 1.1, 0.4 Hz, 1H), 6.86 – 6.80 (m, 2H), 6.78 – 6.74 (m, 1H), 5.95 (br.s, 1H), 2.15 (s, 3H).
$^{13}$C NMR (101 MHz, $CDCl_3$): δ 203.9, 162.9, 153.5, 137.2, 137.0, 133.3, 131.3, 125.5, 122.9, 120.7, 119.5, 118.5, 114.1, 20.1.
HRMS (ESI-QTOF) *m/z*: $[M + H]^+$ Calcd for $C_{14}H_{13}O_3$ 229.0859; Found 229.0859.

**S2**

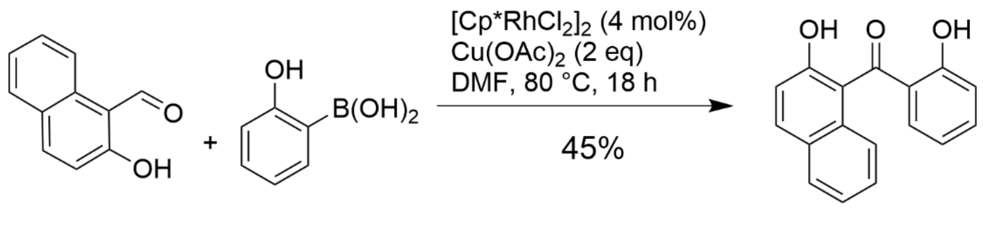


**S2**

The procedure from [4] was followed. Anhydrous DMF (10 mL) was added to the mixture of 2-hydroxy-1-naphthaldehyde (172 mg, 1 mmol), 2-hydroxyphenylboronic acid (276 mg, 2 mmol, 2 eq), copper(II) acetate (364 mg, 2 mmol, 2 eq) and pentamethylcyclopentadienylrhodium(III) chloride dimer (25 mg, 0.04 mmol, 4 mol%), the reaction mixture was degassed and stirred at

80 °C for 18 h. On cooling, the mixture was diluted with $CH_2Cl_2$ and poured into 50 mL of sat. aq. $NH_4Cl$. The product was extracted with $CH_2Cl_2$ (3×30 mL), the combined extracts were washed with water (2×150 mL) and brine (50 mL), dried over $Na_2SO_4$. The product was isolated by flash column chromatography (25 g Interchim SiHP 30 µm cartridge, gradient 10% to 70% EtOAc/hexane) and freeze-dried from 1,4-dioxane to give 139 mg (45% considering purity) of **S2** as light tan solid with ~85% purity, which was used in the next step without additional purification.

$^1$H NMR (400 MHz, $CDCl_3$): δ 11.87 (s, 1H), 8.06 (s, 1H), 7.90 (d, J = 8.9 Hz, 1H), 7.83 – 7.77 (m, 1H), 7.53 – 7.44 (m, 2H), 7.37 – 7.29 (m, 2H), 7.26 – 7.20 (m, 3H), 7.10 (dd, J = 8.5, 1.1 Hz, 1H), 6.69 (ddd, J = 8.2, 7.2, 1.1 Hz, 1H).
$^{13}$C NMR (101 MHz, $CDCl_3$): δ 203.1, 162.8, 155.6, 137.2, 134.2, 134.0, 132.1, 128.6, 128.5, 127.4, 125.5, 124.2, 121.1, 119.2, 118.8, 118.7, 116.8.

**S3**

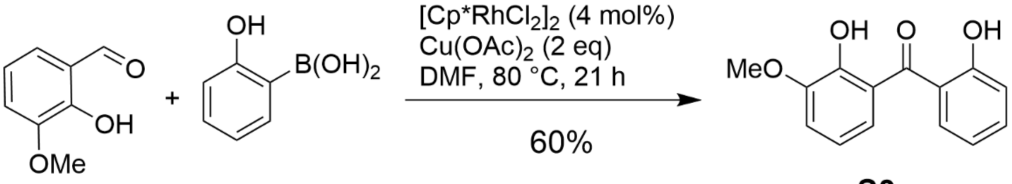


**S3**

The procedure from [4] was followed. Anhydrous DMF (10 mL) was added to the mixture of *o*-vanillin (152 mg, 1 mmol), 2-hydroxyphenylboronic acid (276 mg, 2 mmol, 2 eq), copper(II) acetate (364 mg, 2 mmol, 2 eq) and pentamethylcyclopentadienylrhodium(III) chloride dimer (25 mg, 0.04 mmol, 4 mol%), the reaction mixture was degassed and stirred at 80 °C for 21 h. On cooling, the mixture was diluted with $CH_2Cl_2$ and poured into 50 mL of sat. aq. $NH_4Cl$. The product was extracted with $CH_2Cl_2$ (3×30 mL), the combined extracts were washed with 0.1 N HCl (100 mL) and brine (50 mL), dried over $Na_2SO_4$. The product was isolated by flash column chromatography (25 g Interchim SiHP 30 µm cartridge, gradient 10% to 60% EtOAc/hexane) and dried *in vacuo* to give 147 mg (60%) of **S3** as viscous yellow oil.

$^1$H NMR (400 MHz, $CDCl_3$): δ 11.13 (s, 1H), 9.11 (s, 1H), 7.58 (ddd, J = 8.0, 1.7, 0.4 Hz, 1H), 7.50 (ddd, J = 8.6, 7.2, 1.7 Hz, 1H), 7.12 (dd, J = 8.0, 1.4 Hz, 1H), 7.11 – 7.03 (m, 2H), 6.94 – 6.87 (m, 2H), 3.95 (s, 3H).
$^{13}$C NMR (101 MHz, $CDCl_3$): δ 202.2, 162.3, 149.0, 148.5, 136.3, 133.6, 123.4, 121.9, 120.1, 119.0, 118.9, 118.5, 115.5, 56.4.
HRMS (ESI-QTOF) *m/z*: $[M + H]^+$ Calcd for $C_{14}H_{13}O_4$ 245.0808; Found 245.0807.

**S4**

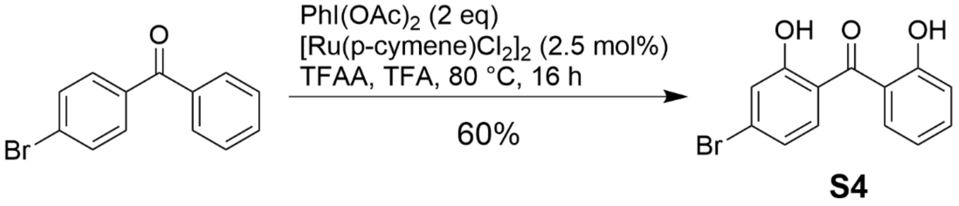


The procedure from [3] was followed. In a 100 mL Ace pressure tube (back seal, capacity 100 mL, length 17.8 cm, diameter 38.1 mm), (diacetoxyiodo)benzene (2.58 g, 8 mmol, 2 eq) and dichloro(*p*-cymene)ruthenium(II) dimer (61 mg, 0.10 mmol, 2.5 mol%) were added to the solution of 4-bromobenzophenone (1.04 g, 4 mmol) in the mixture of trifluoroacetic acid (16 mL) and trifluoroacetic anhydride (8 mL), and the tube was sealed. The reaction mixture was stirred at 80 °C for 16 h (behind a safety shield), cooled down to rt and evaporated to brown oil, which was wet-loaded with minimal amount of $CH_2Cl_2$ on top of a primed 40 g BGB Scorpius 50 µm silica flash cartridge. The crude product was isolated by flash column chromatography (gradient 0% to 20% EtOAc/hexane) and freeze-dried from 1,4-dioxane to give yellow solid, yield 703 mg (60%).

$^1$H NMR (400 MHz, $CDCl_3$): δ 10.74 (s, 1H), 10.45 (s, 1H), 7.58 – 7.50 (m, 2H), 7.48 (d, J = 8.5 Hz, 1H), 7.29 (d, J = 1.9 Hz, 1H), 7.13 – 7.05 (m, 2H), 6.95 (ddd, J = 8.3, 7.3, 1.2 Hz, 1H).
$^{13}$C NMR (101 MHz, $CDCl_3$): δ 201.8, 162.4, 161.9, 136.4, 134.1, 132.8, 130.7, 122.6, 122.0, 119.8, 119.2, 118.9, 118.8.
HRMS (ESI-QTOF) *m/z*: [M + H]$^+$ Calcd for $C_{13}H_{10}BrO_3$ 292.9808; Found 292.9807.

**S5**

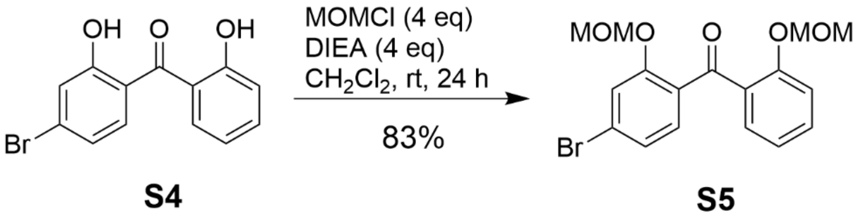


A solution of **S4** (700 mg, 2.39 mmol) and *N*-ethyldiisopropylamine (DIEA; 1.67 mL, 9.56 mmol, 4 eq) in dry $CH_2Cl_2$ (10 mL) was placed in a rt water bath, and chloromethyl methyl ether (0.74 mL, 9.56 mmol, 4 eq) was added dropwise. The resulting orange-brown reaction mixture was left stirring at rt for 24 h. It was then poured into sat. aq. $NaHCO_3$ (50 mL), extracted with $CH_2Cl_2$ (3×30 mL), the combined extracts were washed with brine (50 mL) and dried over $Na_2SO_4$. The product was isolated by flash column chromatography (40 g BGB Scorpius 50 µm silica cartridge, gradient 0% to 40% EtOAc/hexane with 20% $CH_2Cl_2$ constant additive) and freeze-dried from 1,4-dioxane to give 756 mg (83%) of **S5** as white solid.

$^{1}H$ NMR (400 MHz, $CDCl_3$): δ 7.52 (dd, J = 7.6, 1.8 Hz, 1H), 7.45 – 7.39 (m, 2H), 7.30 (d, J = 1.8 Hz, 1H), 7.21 (dd, J = 8.2, 1.7 Hz, 1H), 7.11 (dd, J = 8.3, 1.0 Hz, 1H), 7.06 (td, J = 7.5, 1.0 Hz, 1H), 4.98 (s, 2H), 4.95 (s, 2H), 3.28 (s, 3H), 3.26 (s, 3H).
$^{13}C$ NMR (101 MHz, $CDCl_3$): δ 194.6, 156.4, 155.8, 132.9, 131.3, 131.0, 130.4, 130.2, 126.4, 125.0, 121.9, 118.5, 115.1, 95.0, 94.9, 56.4, 56.3.
HRMS (ESI-QTOF) *m/z*: $[M + Na]^+$ Calcd for $C_{17}H_{17}BrO_5Na$ 403.0152; Found 403.0149.

**S6**

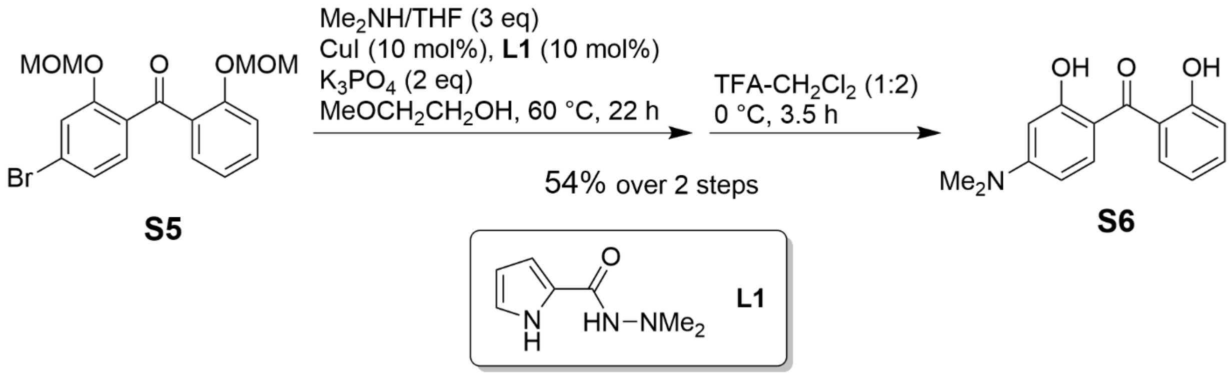


Dimethylamine (1.5 mL of 2 M solution in THF, 3 mmol, 3 eq) was added to a degassed mixture of **S5** (381 mmol, 1 mmol), copper(I) iodide (19 mg, 0.1 mmol, 10 mol%), ligand **L1** (28 mg, 0.1 mmol, 10 mol%; known compound [5]) and anhydrous $K_3PO_4$ (424 mg, 2 mmol, 2 eq) in 2-methoxyethanol (4 mL), and the reaction mixture was stirred at 60 °C for 22 h. It was then diluted with brine (50 mL), extracted with EtOAc (3×25 mL), and the combined extracts were dried over $Na_2SO_4$. The di-MOM-protected intermediate was isolated by flash column chromatography (25 g Interchim SiHP 30 µm cartridge, gradient 20% to 100% EtOAc/hexane) and used directly in the next step.

The material from the previous step was dissolved in $CH_2Cl_2$ (10 mL), cooled in ice-water bath, and trifluoroacetic acid (5 mL) was added. After 3.5 h (TLC control: 60% EtOAc/hexane on silica, $R_f$(starting material) = 0.45, $R_f$(**S6**) = 0.60), the reaction mixture was diluted with toluene, evaporated to dryness and chased with toluene. The product was isolated by flash column chromatography (25 g Interchim SiHP 30 µm cartridge, gradient 10% to 60% EtOAc/hexane) and freeze-dried from 1,4-dioxane to give 140 mg (54% over 2 steps) of **S6** as bright yellow solid.

$^{1}H$ NMR (400 MHz, $CDCl_3$): δ 12.17 (s, 1H), 10.27 (s, 1H), 7.56 – 7.51 (m, 2H), 7.42 (ddd, J = 8.4, 7.3, 1.7 Hz, 1H), 7.04 (dd, J = 8.3, 1.2 Hz, 1H), 6.90 (ddd, J = 7.8, 7.3, 1.2 Hz, 1H), 6.23 (dd, J = 9.2, 2.6 Hz, 1H), 6.18 (d, J = 2.6 Hz, 1H), 3.09 (s, 6H).
$^{13}C$ NMR (101 MHz, $CDCl_3$): δ 198.4, 165.9, 160.4, 156.1, 135.4, 134.2, 132.2, 121.1, 118.6, 118.2, 109.4, 104.2, 98.3, 40.1.

HRMS (ESI-QTOF) *m/z*: [M + H]$^+$ Calcd for $C_{15}H_{16}NO_3$ 258.1125; Found 258.1121.

**S7**

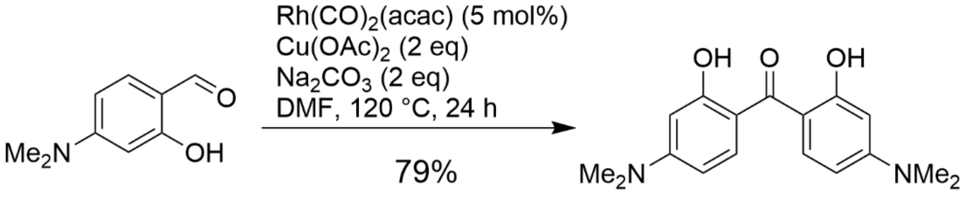


**S7**

The procedure from [6] was followed. Anhydrous DMF (18 mL) was added to the mixture of 4-(dimethylamino)-2-hydroxybenzaldehyde (495 mg, 3 mmol), copper(II) acetate (1.09 g, 6 mmol, 2 eq), sodium carbonate (636 mg, 6 mmol, 2 eq) and (acetylacetonato)dicarbonylrhodium(I) (CARAC; 39 mg, 0.15 mmol, 5 mol%), the reaction mixture was degassed and stirred at 120 °C for 24 h. On cooling, the mixture was diluted with ethyl acetate and poured into 100 mL of 0.5 M HCl. The mixture was adjusted to pH ~3 with 1 N NaOH and the product extracted with EtOAc (3×50 mL), the combined extracts were washed with brine (50 mL) and dried over $Na_2SO_4$. The product was isolated by flash column chromatography (25 g Interchim SiHP 30 μm cartridge, gradient 0% to 50% EtOAc/hexane with 20% $CH_2Cl_2$ constant additive) and freeze-dried from 1,4-dioxane to give 357 mg (79%) of **S7** as yellow solid.

$^1$H NMR (400 MHz, $CDCl_3$): δ 11.82 (s, 2H), 7.54 – 7.48 (m, 2H), 6.24 – 6.18 (m, 4H), 3.06 (s, 12H).
$^{13}$C NMR (101 MHz, $CDCl_3$): δ 196.8, 164.4, 155.1, 134.5, 109.8, 103.5, 98.7, 40.1.
HRMS (ESI-QTOF) *m/z*: [M + H]$^+$ Calcd for $C_{17}H_{21}N_2O_3$ 301.1547; Found 301.1546.

**S8**

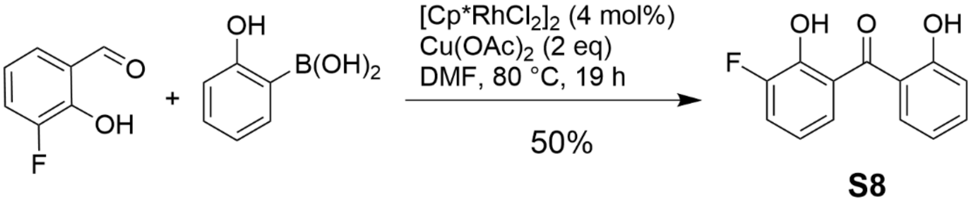


**S8**

The procedure from [4] was followed. Anhydrous DMF (15 mL) was added to the mixture of 3-fluorosalicylaldehyde (210 mg, 1.5 mmol), 2-hydroxyphenylboronic acid (414 mg, 3 mmol, 2 eq), copper(II) acetate (546 mg, 3 mmol, 2 eq) and pentamethylcyclopentadienylrhodium(III) chloride dimer (37 mg, 0.06 mmol, 4 mol%), the reaction mixture was degassed and stirred at 80 °C for 19 h. On cooling, the mixture was diluted with $CH_2Cl_2$ and poured into 100 mL of sat. aq. $NH_4Cl$. The product was extracted with $CH_2Cl_2$ (3×30 mL), the combined extracts were washed 0.1 N HCl (100 mL), brine (50 mL) and dried over $Na_2SO_4$. The product was isolated by flash column

chromatography (25 g Interchim SiHP 30 µm cartridge, gradient 20% to 100% $CH_2Cl_2$/hexane) and freeze-dried from 1,4-dioxane to give 175 mg (50%) of **S8** as yellow solid.

$^1$H NMR (400 MHz, $CDCl_3$): δ 10.65 (s, 1H), 10.14 (s, 1H), 7.60 (dd, J = 8.0, 1.7 Hz, 1H), 7.54 (ddd, J = 8.8, 7.3, 1.7 Hz, 1H), 7.39 (dt, J = 8.1, 1.4 Hz, 1H), 7.33 (ddd, J = 10.5, 8.1, 1.5 Hz, 1H), 7.10 (dd, J = 8.4, 1.1 Hz, 1H), 6.95 (ddd, J = 8.3, 7.2, 1.2 Hz, 1H), 6.90 (td, J = 7.9, 4.4 Hz, 1H).
$^{19}$F NMR (376 MHz, $CDCl_3$): δ -135.2.
$^{13}$C NMR (101 MHz, $CDCl_3$): δ 201.9 (d, J = 2.6 Hz), 162.2, 152.1 (d, J = 247.3 Hz), 149.6 (d, J = 12.7 Hz), 136.6, 133.2, 128.0 (d, J = 3.8 Hz), 122.4 (d, J = 2.6 Hz), 121.5 (d, J = 17.6 Hz), 119.7, 119.2, 118.9, 118.6 (d, J = 6.6 Hz).
HRMS (ESI-QTOF) *m/z*: $[M + H]^+$ Calcd for $C_{13}H_{10}FO_3$ 233.0608; Found 233.0608.

**S9**

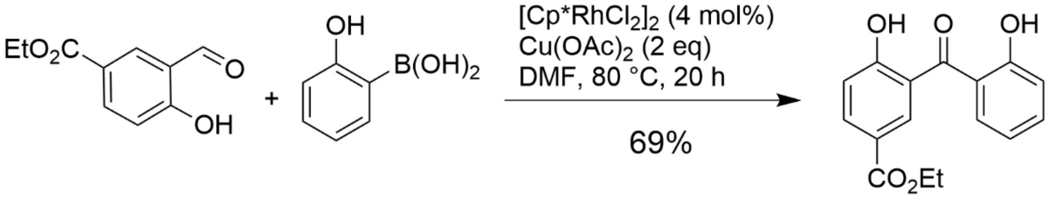


**S9**

The procedure from [4] was followed. Anhydrous DMF (10 mL) was added to the mixture of ethyl 3-formyl-4-hydroxybenzoate (194 mg, 1 mmol), 2-hydroxyphenylboronic acid (276 mg, 2 mmol, 2 eq), copper(II) acetate (364 mg, 2 mmol, 2 eq) and pentamethylcyclopentadienylrhodium(III) chloride dimer (25 mg, 0.04 mmol, 4 mol%), the reaction mixture was degassed and stirred at 80 °C for 20 h. On cooling, the mixture was diluted with $CH_2Cl_2$ and poured into 50 mL of sat. aq. $NH_4Cl$. The product was extracted with $CH_2Cl_2$ (3×30 mL), the combined extracts were washed with water (2×100 mL) and brine (50 mL), dried over $Na_2SO_4$. The product was isolated by flash column chromatography (25 g Interchim SiHP 30 µm cartridge, gradient 2% to 40% EtOAc/hexane with 20% $CH_2Cl_2$ constant additive) and freeze-dried from 1,4-dioxane to give 197 mg (69%) of **S9** as light yellow solid.

$^1$H NMR (400 MHz, $CDCl_3$): δ 10.98 (s, 1H), 10.53 (s, 1H), 8.38 (d, *J* = 2.2 Hz, 1H), 8.18 (dd, *J* = 8.7, 2.2 Hz, 1H), 7.61 (dd, *J* = 8.0, 1.7 Hz, 1H), 7.56 (ddd, *J* = 8.7, 7.2, 1.7 Hz, 1H), 7.15 – 7.09 (m, 2H), 6.98 (ddd, *J* = 8.2, 7.2, 1.2 Hz, 1H), 4.36 (q, *J* = 7.1 Hz, 2H), 1.37 (t, *J* = 7.1 Hz, 3H).
$^{13}$C NMR (101 MHz, $CDCl_3$): δ 202.0, 165.5, 165.3, 162.1, 136.8, 136.6, 135.4, 133.0, 121.7, 119.6, 119.41, 119.38, 118.9, 118.8, 61.3, 14.5.
HRMS (ESI-QTOF) *m/z*: $[M + H]^+$ Calcd for $C_{16}H_{15}O_5$ 287.0914; Found 287.0914.

**S10**

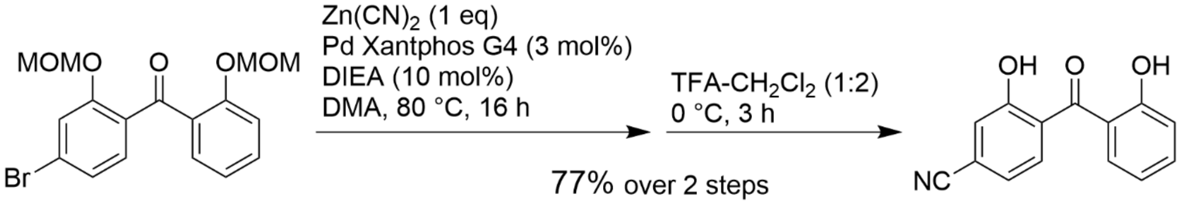


**S5** **S10**

*N*-Ethyldiisopropylamine (DIEA; 16 μL, 0.092 mmol, 10 mol%) was injected to a degassed mixture of **S5** (351 mmol, 0.92 mmol), zinc cyanide (108 mg, 0.92 mmol, 1 eq) and Pd Xantphos G4 (26 mg, 27.6 μmol, 3 mol%) in anhydrous *N*,*N*-dimethylacetamide (DMA; 1 mL), and the reaction mixture was stirred at 80 °C for 16 h. It was then diluted with $CH_2Cl_2$ and poured into water (100 mL), extracted with $CH_2Cl_2$ (3×20 mL), the combined extracts were washed with water (100 mL), brine (50 mL) and dried over $Na_2SO_4$. The di-MOM-protected intermediate was isolated by flash column chromatography (25 g Interchim SiHP 30 μm cartridge, gradient 5% to 50% EtOAc/hexane) and used directly in the next step.

The material from the previous step was dissolved in $CH_2Cl_2$ (10 mL), cooled in ice-water bath, and trifluoroacetic acid (5 mL) was added. After 3 h, the reaction mixture was diluted with toluene (20 mL), evaporated to dryness and chased with toluene. The product was isolated by flash column chromatography (25 g Interchim SiHP 30 μm cartridge, gradient 10% to 60% EtOAc/hexane) and freeze-dried from 1,4-dioxane to give 169 mg (77% over 2 steps) of **S10** as yellow solid.

$^1H$ NMR (400 MHz, $CDCl_3$): δ 10.58 (s, 1H), 10.29 (s, 1H), 7.70 (d, J = 8.2 Hz, 1H), 7.58 (ddd, J = 8.7, 7.2, 1.7 Hz, 1H), 7.54 (dd, J = 8.1, 1.6 Hz, 1H), 7.38 (d, J = 1.5 Hz, 1H), 7.23 (dd, J = 8.2, 1.6 Hz, 1H), 7.12 (dd, J = 8.4, 1.1 Hz, 1H), 6.97 (ddd, J = 8.2, 7.2, 1.2 Hz, 1H).
$^{13}C$ NMR (101 MHz, $CDCl_3$): δ 201.4, 162.5, 160.9, 137.2, 133.5, 132.8, 123.3, 122.6, 122.0, 119.5, 119.3, 119.2, 118.3, 117.5.
HRMS (ESI-QTOF) *m/z*: $[M + H]^+$ Calcd for $C_{14}H_{10}NO_3$ 240.0655; Found 240.0656.

## Preparation of aryl triflates 1a', 1b-o and 1r-t

### General procedure for the preparation of aryl triflates

Trifluoromethanesulfonic anhydride (1.0 mL, 6.14 mmol, 3 eq) was added quickly dropwise to a stirred solution of the corresponding 2,2'-dihydroxybenzophenone (2.05 mmol) and pyridine (1.0 mL, 12.3 mmol, 6 equiv) in dry $CH_2Cl_2$ (15 mL), cooled in ice-water bath. The reaction mixture was allowed to warm up and left stirring at rt for 1 h. The mixture was then diluted with brine (50

mL), extracted with $CH_2Cl_2$ (3×25 mL) and the combined extracts were dried over $Na_2SO_4$, and the product was isolated by flash column chromatography (silica, EtOAc/hexane, unless indicated otherwise) and freeze-dried from 1,4-dioxane (for solid products) or dried *in vacuo* (for liquids).

**1a'**

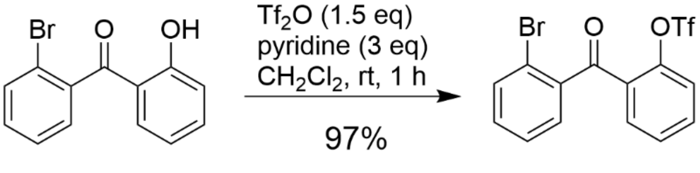


**1a'**

Trifluoromethanesulfonic anhydride (0.35 mL of 1 M solution in $CH_2Cl_2$, ~0.35 mmol, 1.5 eq) was added to the stirred solution of 2-bromo-2'-hydroxybenzophenone (65 mg, 0.24 mmol; known compound [7]) and pyridine (60 µL, 0.71 mmol, 3 equiv) in dry $CH_2Cl_2$ (2 mL), and the reaction mixture was left stirring at rt for 1 h. The mixture was diluted with brine (40 mL), extracted with $CH_2Cl_2$ (3×20 mL) and the combined extracts were dried over $Na_2SO_4$. The product was isolated by flash column chromatography (12 g Interchim SiHP 30 µm cartridge, gradient 5% to 60% EtOAc/hexane) and freeze-dried from 1,4-dioxane to give 93 mg (97%) of **1a'** as white solid.

$^1$H NMR (400 MHz, $CDCl_3$): δ 7.68 – 7.63 (m, 2H), 7.59 (dd, J = 7.8, 1.8 Hz, 1H), 7.52 – 7.36 (m, 5H).
$^{19}$F NMR (376 MHz, $CDCl_3$): δ -73.3.
$^{13}$C NMR (101 MHz, $CDCl_3$): δ 192.6, 147.7, 139.4, 134.3, 133.9, 133.0, 132.7, 131.4, 131.1, 128.5, 127.6, 122.9, 120.7, 118.70 (q, J = 320.7 Hz).
HRMS (ESI-QTOF) *m/z*: $[M + H]^+$ Calcd for $C_{14}H_9BrF_3O_4S$ 408.9352; Found 408.9354.

**1b**

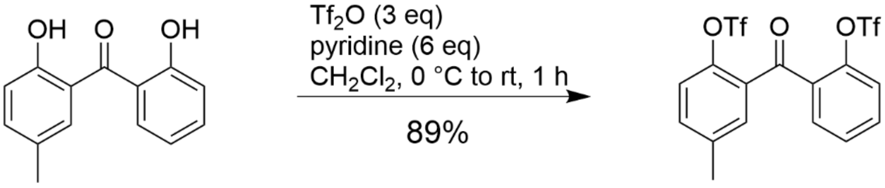


**1b**

Prepared according to the general procedure from 2,2'-dihydroxy-5-methylbenzophenone (467 mg, 2.05 mmol; known compound [8]). The product was isolated by flash column chromatography (25 g Interchim SiHP 30 µm cartridge, gradient 5% to 60% EtOAc/hexane) and freeze-dried from 1,4-dioxane to give 900 mg (89%) of **1b** as white solid.

$^1$H NMR (400 MHz, $CDCl_3$): δ 7.71 – 7.64 (m, 2H), 7.55 – 7.46 (m, 1H), 7.47 – 7.43 (m, 2H), 7.43 – 7.36 (m, 1H), 7.28 – 7.24 (m, 1H), 2.42 (s, 3H).
$^{19}$F NMR (376 MHz, $CDCl_3$): δ -73.15, -73.19.
$^{13}$C NMR (101 MHz, $CDCl_3$): δ 189.4, 147.4, 145.3, 139.1, 134.8, 134.3, 132.8, 132.6, 131.8, 131.4, 128.6, 122.6, 122.4, 118.61 (q, $J$ = 320.8 Hz), 21.0.
HRMS (ESI-QTOF) *m/z*: $[M + Na]^+$ Calcd for $C_{16}H_{10}F_6O_7S_2Na$ 514.9664; Found 514.9659.

**1c**

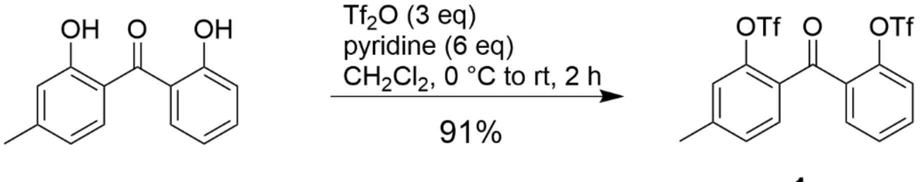


**1c**

Prepared according to the general procedure from 2,2'-dihydroxy-4-methylbenzophenone (400 mg, 1.75 mmol; known compound [8]). The product was isolated by flash column chromatography (25 g Interchim SiHP 30 µm cartridge, gradient 5% to 50% EtOAc/hexane) and freeze-dried from 1,4-dioxane to give 786 mg (91%) of **1c** as white solid.

$^1$H NMR (400 MHz, $CDCl_3$): δ 7.69 – 7.63 (m, 2H), 7.55 (d, J = 7.9 Hz, 1H), 7.49 (ddd, J = 8.1, 7.3, 1.1 Hz, 1H), 7.41 – 7.37 (m, 1H), 7.29 (ddd, J = 7.9, 1.6, 0.8 Hz, 1H), 7.21 – 7.17 (m, 1H), 2.49 (d, J = 0.7 Hz, 3H).
$^{19}$F NMR (376 MHz, $CDCl_3$): δ -73.18, -73.23.
$^{13}$C NMR (101 MHz, $CDCl_3$): δ 189.1, 147.4, 147.3, 146.3, 134.0, 132.6, 132.4, 132.0, 129.2, 128.8, 128.5, 123.1, 122.6, 118.6 (q, $J$ = 320.7 Hz), 21.8.
HRMS (ESI-QTOF) *m/z*: $[M + Na]^+$ Calcd for $C_{16}H_{10}F_6O_7S_2Na$ 514.9664; Found 514.9662.

**1d**

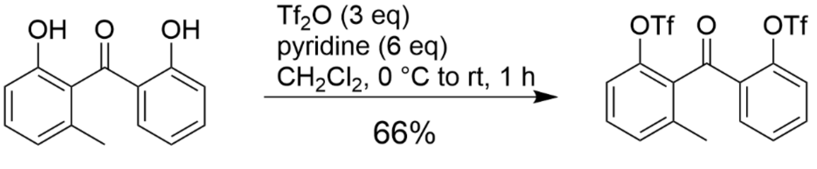


**S1** **1d**

Prepared according to the general procedure from **S1** (439 mg, 1.92 mmol). The product was isolated by flash column chromatography (25 g Interchim SiHP 30 µm cartridge, gradient 5% to 60% EtOAc/hexane) and freeze-dried from 1,4-dioxane to give 626 mg (66%) of **1d** as viscous yellowish oil, which slowly crystallized in freezer.

$^{1}H$ NMR (400 MHz, $CDCl_3$): δ 7.70 (ddd, J = 8.3, 7.4, 1.8 Hz, 1H), 7.65 (dd, J = 7.8, 1.8 Hz, 1H), 7.51 – 7.45 (m, 2H), 7.42 – 7.38 (m, 1H), 7.33 (dt, J = 7.8, 0.9 Hz, 1H), 7.27 – 7.24 (m, 1H), 2.33 (s, 3H).

$^{19}F$ NMR (376 MHz, $CDCl_3$): δ -73.35, -73.60.

$^{13}C$ NMR (101 MHz, $CDCl_3$): δ 190.3, 147.9, 146.5, 139.9, 135.5, 133.4, 132.0, 131.7, 130.9, 130.4, 128.9, 123.17, 123.15, 118.9, 118.8 (q, *J* = 320.6 Hz), 118.4 (q, *J* = 320.5 Hz), 19.8.

HRMS (ESI-QTOF) *m/z*: $[M + Na]^+$ Calcd for $C_{16}H_{10}F_6O_7S_2Na$ 514.9664; Found 514.9663.

**1e**

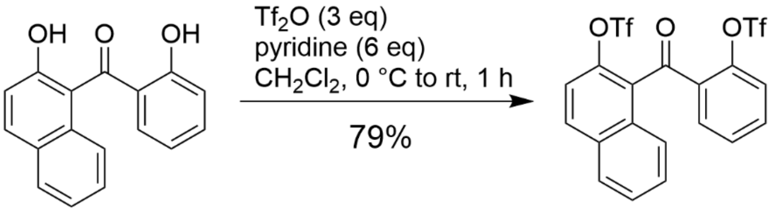


**S2** **1e**

Prepared according to the general procedure from **S2** (139 mg, <0.53 mmol, crude material from the previous step). The product was isolated by flash column chromatography (12 g Interchim SiHP 30 µm cartridge, gradient 5% to 60% EtOAc/hexane) and freeze-dried from 1,4-dioxane to give 220 mg (79%, or 42% over 2 steps) of **1e** as light tan solid.

$^{1}H$ NMR (400 MHz, $CDCl_3$): δ 8.09 (dd, J = 9.1, 0.9 Hz, 1H), 7.99 – 7.95 (m, 1H), 7.84 (ddd, J = 7.9, 1.6, 0.7 Hz, 1H), 7.70 (ddd, J = 8.3, 7.4, 1.8 Hz, 1H), 7.65 – 7.55 (m, 3H), 7.51 (d, J = 9.1 Hz, 1H), 7.46 – 7.39 (m, 2H).

$^{19}F$ NMR (376 MHz, $CDCl_3$): δ -73.34, -73.54.

$^{13}C$ NMR (101 MHz, $CDCl_3$): δ 190.2, 147.9, 144.1, 135.6, 134.0, 133.2, 132.5, 131.3, 130.6, 129.1, 128.84, 128.77, 128.6, 127.9, 125.7, 123.3, 119.0, 118.8 (q, *J* = 320.7 Hz), 118.4 (q, *J* = 320.5 Hz).

HRMS (ESI-QTOF) *m/z*: $[M + Na]^+$ Calcd for $C_{19}H_{10}F_6O_7S_2Na$ 550.9664; Found 550.9666.

**1f**

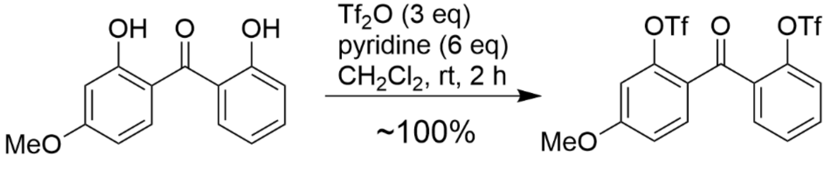


**1f**

Prepared according to the general procedure from 2,2'-dihydroxy-4-methoxybenzophenone (1.22 g, 5 mmol). The product was isolated by flash column chromatography (40 g BGB Scorpius

50 μm cartridge, gradient 20% to 80% EtOAc/hexane) and freeze-dried from 1,4-dioxane to give 2.55 g (~100%, remainder dioxane) of **1f** as white solid.

$^{1}$H NMR (400 MHz, $CDCl_3$): δ 7.68 – 7.60 (m, 3H), 7.49 (td, J = 7.5, 1.1 Hz, 1H), 7.42 – 7.35 (m, 1H), 6.96 (dd, J = 8.8, 2.4 Hz, 1H), 6.88 (d, J = 2.4 Hz, 1H), 3.91 (s, 3H).
$^{19}$F NMR (376 MHz, $CDCl_3$): δ -73.21, -73.13.
$^{13}$C NMR (101 MHz, $CDCl_3$): δ 188.4, 164.3, 149.0, 147.1, 134.5, 133.7, 132.5, 132.1, 128.5, 123.8, 122.5, 118.64 (q, *J* = 320.8 Hz), 118.60 (q, *J* = 320.8 Hz), 113.3, 109.1, 56.3.
HRMS (ESI-QTOF) *m/z*: $[M + Na]^+$ Calcd for $C_{16}H_{10}F_6O_8S_2Na$ 530.9613; Found 530.9609.

**1g**

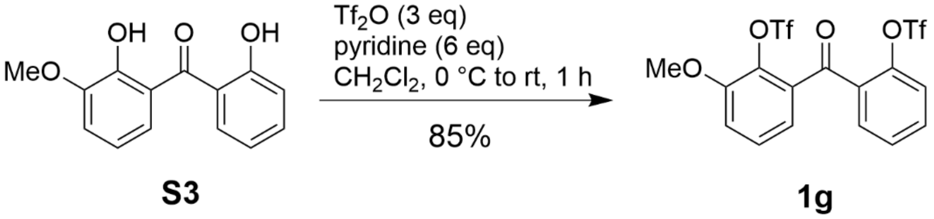


Prepared according to the general procedure from **S3** (147 mg, 0.60 mmol). The product was isolated by flash column chromatography (12 g Interchim SiHP 30 μm cartridge, gradient 20% to 80% EtOAc/hexane) and freeze-dried from 1,4-dioxane to give 261 mg (85%) of **1g** as yellowish solid.

$^{1}$H NMR (400 MHz, $CDCl_3$): δ 7.70 – 7.64 (m, 2H), 7.48 (ddd, J = 8.1, 7.3, 1.1 Hz, 1H), 7.44 – 7.37 (m, 2H), 7.26 (dd, J = 8.4, 1.5 Hz, 1H), 7.15 (dd, J = 7.8, 1.5 Hz, 1H), 3.96 (s, 3H).
$^{19}$F NMR (376 MHz, $CDCl_3$): δ -73.17, -73.56.
$^{13}$C NMR (101 MHz, $CDCl_3$): δ 189.2, 151.9, 147.6, 136.8, 134.4, 132.9, 132.9, 131.4, 128.8, 128.4, 123.1, 122.7, 118.7 (q, *J* = 320.7 Hz), 118.6 (q, *J* = 320.7 Hz), 117.0, 56.7.
HRMS (ESI-QTOF) *m/z*: $[M + Na]^+$ Calcd for $C_{16}H_{10}F_6O_8S_2Na$ 530.9613; Found 530.9607.

**1h**

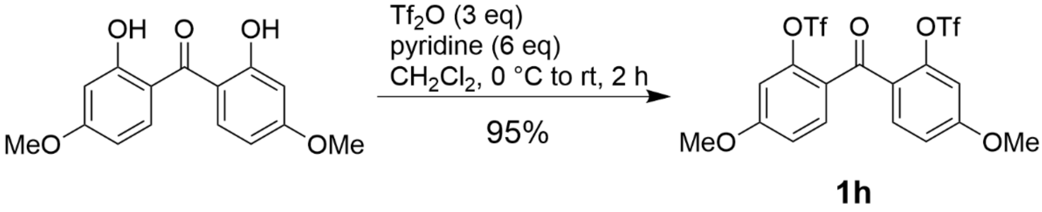


Trifluoromethanesulfonic anhydride (10 mL, 60 mmol, 3 eq) was added quickly dropwise to a stirred solution of 2,2'-dihydroxy-4,4'-dimethoxybenzophenone (5.48 g, 20 mmol) and pyridine (9.7 mL, 120 mmol, 6 equiv) in dry $CH_2Cl_2$ (100 mL), cooled in ice-water bath, and the reaction mixture was allowed to warm up and left stirring at rt for 2 h. The mixture was cooled in ice-

water, diluted with water (100 mL), the layers were separated and the aqueous layer was extracted with $CH_2Cl_2$ (2×50 mL). The combined extracts were washed with water (2×200 mL), brine (100 mL), dried over $Na_2SO_4$ and filtered through a 3 cm plug of silica, washing with additional $CH_2Cl_2$ until all product is eluted (to a ~300 mL total volume). The filtrate was evaporated, and the product was crystallized from EtOAc/hexane to give 10.22 g (95%) of **1h** as white crystals.

$^1$H NMR (400 MHz, $CDCl_3$): δ 7.61 (d, J = 8.7 Hz, 2H), 6.96 (dd, J = 8.7, 2.4 Hz, 2H), 6.86 (d, J = 2.4 Hz, 2H), 3.91 (s, 6H).
$^{19}$F NMR (376 MHz, $CDCl_3$): δ -73.2.
$^{13}$C NMR (101 MHz, $CDCl_3$): δ 187.7, 163.9, 148.7, 134.0, 124.6, 118.6 (q, *J* = 320.8 Hz), 113.4, 108.8, 56.3.
HRMS (ESI-QTOF) *m/z*: $[M + H]^+$ Calcd for $C_{17}H_{13}F_6O_9S_2$ 538.9900; Found 538.9897.

**1i**

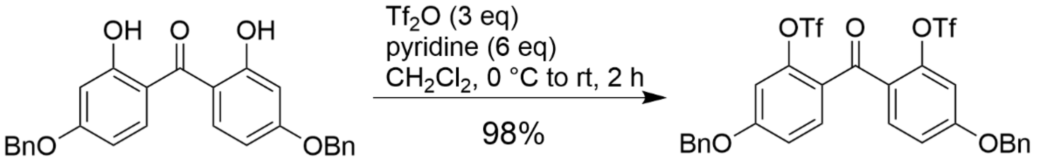


**1i**

Prepared according to the general procedure from 4,4'-dibenzyloxy-2,2'-dihydroxybenzophenone (500 mg, 1.17 mmol; known compound [9]). The product was isolated by flash column chromatography (25 g Interchim SiHP 30 µm cartridge, gradient 5% to 50% EtOAc/hexane) and freeze-dried from 1,4-dioxane to give 792 mg (98%) of **1i** as white solid.

$^1$H NMR (400 MHz, $CDCl_3$): δ 7.60 (d, J = 8.7 Hz, 2H), 7.44 – 7.35 (m, 10H), 7.02 (dd, J = 8.7, 2.4 Hz, 2H), 6.93 (d, J = 2.3 Hz, 2H), 5.14 (s, 4H).
$^{19}$F NMR (376 MHz, $CDCl_3$): δ -73.15.
$^{13}$C NMR (101 MHz, $CDCl_3$): δ 187.7, 162.9, 148.6, 135.3, 134.0, 129.0, 128.8, 127.7, 124.7, 118.6 (q, *J* = 320.9 Hz), 114.2, 109.6, 71.1.
HRMS (ESI-QTOF) *m/z*: $[M + H]^+$ Calcd for $C_{29}H_{21}F_6O_9S_2$ 691.0526; Found 691.0518.

**1j**

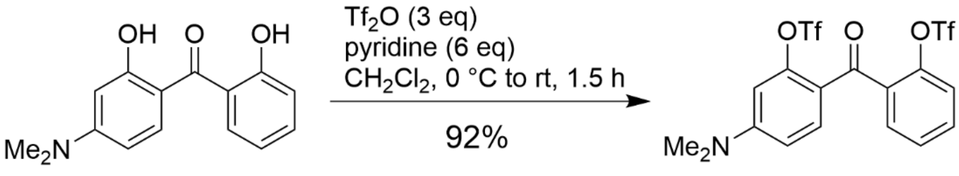


**S6** **1j**

Prepared according to the general procedure from **S6** (130 mg, 0.51 mmol). The product was isolated by flash column chromatography (12 g Interchim SiHP 30 µm cartridge, gradient 10% to 60% EtOAc/hexane) and freeze-dried from 1,4-dioxane to give 243 mg (92%) of **1j** as light-yellow solid.

$^{1}$H NMR (400 MHz, $CDCl_3$): δ 7.62 – 7.56 (m, 2H), 7.50 – 7.43 (m, 2H), 7.40 – 7.33 (m, 1H), 6.58 (dd, J = 9.0, 2.5 Hz, 1H), 6.49 (d, J = 2.5 Hz, 1H), 3.09 (s, 6H).
$^{19}$F NMR (376 MHz, $CDCl_3$): δ -73.29, -73.36.
$^{13}$C NMR (101 MHz, $CDCl_3$): δ 187.8, 154.4, 150.2, 146.9, 135.0, 133.4, 132.7, 131.7, 128.3, 122.2, 118.8 (q, *J* = 320.9 Hz), 118.6 (q, *J* = 320.7 Hz), 117.5, 109.9, 104.8, 40.2.
HRMS (ESI-QTOF) *m/z*: $[M + H]^+$ Calcd for $C_{17}H_{14}F_6NO_7S_2$ 522.0110; Found 522.0108.

**1k**

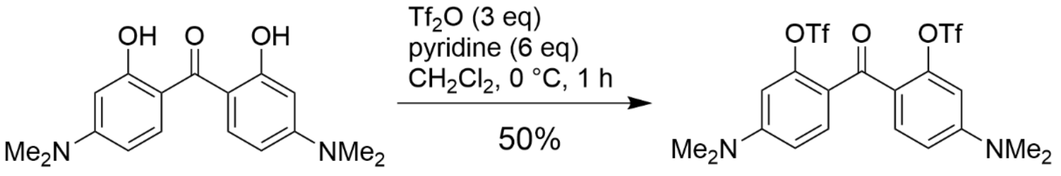


**S7** **1k**

Prepared according to the general procedure from **S7** (347 mg, 1.16 mmol). The product was isolated by flash column chromatography (25 g Interchim SiHP 30 µm cartridge, gradient 20 to 80% EtOAc/hexane) and freeze-dried from 1,4-dioxane to give 325 mg (50%) of **1k** as light-yellow solid.

$^{1}$H NMR (400 MHz, $CDCl_3$): δ 7.50 (d, J = 8.9 Hz, 2H), 6.60 (dd, J = 8.9, 2.5 Hz, 2H), 6.48 (d, J = 2.5 Hz, 2H), 3.07 (s, 12H).
$^{19}$F NMR (376 MHz, $CDCl_3$): δ -73.25.
$^{13}$C NMR (101 MHz, $CDCl_3$): δ 187.5, 153.7, 149.5, 134.1, 119.4, 118.7 (q, *J* = 320.9 Hz), 110.1, 104.5, 40.2.
HRMS (ESI-QTOF) *m/z*: $[M + H]^+$ Calcd for $C_{19}H_{19}F_6N_2O_7S_2$ 565.0532; Found 565.0530.

**1l**

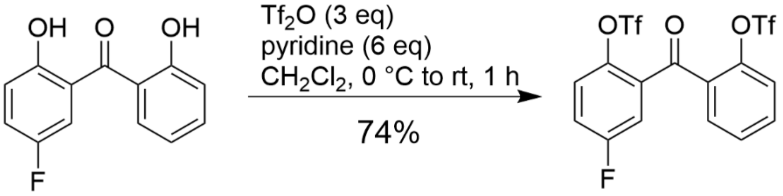


**1l**

Prepared according to the general procedure from 5-fluoro-2,2'-dihydroxybenzophenone (319 mg, 1.38 mmol; known compound [8]). The product was isolated by flash column

chromatography (25 g Interchim SiHP 30 µm cartridge, gradient 10% to 60% EtOAc/hexane) and freeze-dried from 1,4-dioxane to give 509 mg (74%) of **1l** as off-white solid.

$^{1}$H NMR (400 MHz, $CDCl_3$): δ 7.74 – 7.68 (m, 2H), 7.53 (ddd, J = 8.2, 7.3, 1.1 Hz, 1H), 7.43 – 7.33 (m, 4H).
$^{19}$F NMR (376 MHz, $CDCl_3$): δ -72.95, -73.03, -110.24.
$^{13}$C NMR (101 MHz, $CDCl_3$): δ 187.9 (d, J = 1.4 Hz), 161.2 (d, J = 252.4 Hz), 147.4, 142.9 (d, J = 3.5 Hz), 134.8, 133.5 (d, J = 6.8 Hz), 132.6, 131.1, 128.7, 124.6 (d, J = 8.5 Hz), 122.7, 121.0 (d, J = 23.9 Hz), 119.3 (d, J = 25.3 Hz), 118.61 (q, *J* = 320.8 Hz), 118.57 (q, *J* = 320.9 Hz).
HRMS (ESI-QTOF) *m/z*: $[M + Na]^+$ Calcd for $C_{15}H_7F_7O_7S_2Na$ 518.9414; Found 518.9413.

**1m**

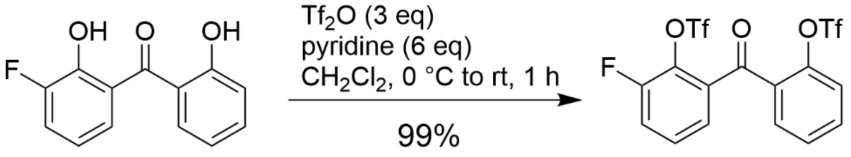


**S8** **1m**

Prepared according to the general procedure from **S8** (319 mg, 1.38 mmol). The product was isolated by flash column chromatography (12 g Interchim SiHP 30 µm cartridge, gradient 10% to 60% EtOAc/hexane) and freeze-dried from 1,4-dioxane to give 367 mg (99%) of **1m** as off-white solid.

$^{1}$H NMR (400 MHz, $CDCl_3$): δ 7.73 – 7.66 (m, 2H), 7.55 – 7.39 (m, 5H).
$^{19}$F NMR (376 MHz, $CDCl_3$): δ -73.09, -73.16 (d, *J* = 10.9 Hz), -125.03 (d, *J* = 10.9 Hz).
$^{13}$C NMR (101 MHz, $CDCl_3$): δ 188.4 (d, J = 2.7 Hz), 154.1 (d, J = 255.7 Hz), 147.5, 135.3 (d, J = 14.2 Hz), 134.7, 133.9, 132.8, 131.2, 129.4 (d, J = 7.5 Hz), 128.6, 127.4 (d, J = 3.6 Hz), 122.8, 121.6 (d, J = 19.2 Hz), 118.62 (q, J = 320.8 Hz), 118.59 (q, *J* = 321.0 Hz).
HRMS (ESI-QTOF) *m/z*: $[M + NH_4]^+$ Calcd for $C_{15}H_{11}F_7NO_7S_2$ 513.9860; Found 513.9859.

**1n**

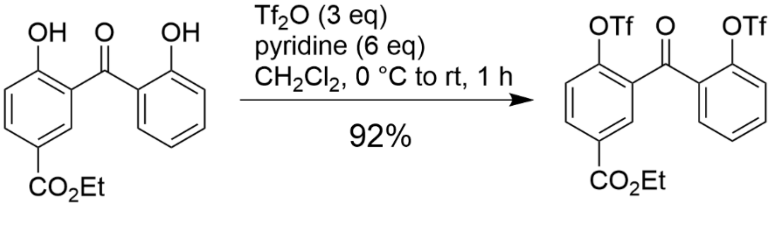


**S9** **1n**

Prepared according to the general procedure from **S9** (197 mg, 0.69 mmol). The product was isolated by flash column chromatography (25 g Interchim SiHP 30 µm cartridge, gradient 0% to

30% EtOAc/hexane with 20% $CH_2Cl_2$ constant additive) and freeze-dried from 1,4-dioxane to give 349 mg (92%) of **1n** as viscous colorless oil, which did not solidify.

$^1$H NMR (400 MHz, $CDCl_3$): δ 8.35 – 8.31 (m, 2H), 7.75 – 7.67 (m, 2H), 7.53 (td, J = 7.6, 1.1 Hz, 1H), 7.50 – 7.46 (m, 1H), 7.44 – 7.39 (m, 1H), 4.41 (q, J = 7.1 Hz, 2H), 1.39 (t, J = 7.1 Hz, 3H).
$^{19}$F NMR (376 MHz, $CDCl_3$): δ -73.00, -73.10.
$^{13}$C NMR (101 MHz, $CDCl_3$): δ 188.4, 164.2, 150.0, 147.5, 135.2, 134.8, 133.6, 132.6, 131.8, 131.1, 131.0, 128.7, 122.7, 118.60 (q, *J* = 320.8 Hz), 118.55 (q, *J* = 321.0 Hz), 62.2, 14.4.
HRMS (ESI-QTOF) *m/z*: [M + Na]$^+$ Calcd for $C_{18}H_{12}F_6O_9S_2Na$ 572.9719; Found 572.9719.

**1o**

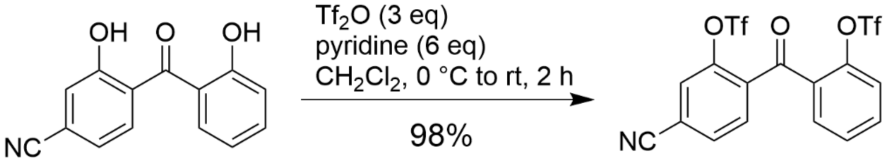


**S10** **1o**

Prepared according to the general procedure from **S10** (160 mg, 0.67 mmol). The product was isolated by flash column chromatography (12 g Interchim SiHP 30 µm cartridge, gradient 10% to 60% EtOAc/hexane) and freeze-dried from 1,4-dioxane to give 329 mg (98%) of **1o** as white solid.

$^1$H NMR (400 MHz, $CDCl_3$): δ 7.81 (d, J = 0.9 Hz, 2H), 7.74 (ddd, J = 8.2, 7.4, 1.8 Hz, 1H), 7.71 – 7.68 (m, 2H), 7.55 (td, J = 7.6, 1.1 Hz, 1H), 7.41 (dd, J = 8.2, 1.0 Hz, 1H).
$^{19}$F NMR (376 MHz, $CDCl_3$): δ -72.74, -72.89.
$^{13}$C NMR (101 MHz, $CDCl_3$): δ 187.8, 147.4, 146.9, 135.7, 135.3, 133.1, 132.6, 132.2, 130.7, 128.9, 126.2, 122.8, 118.6 (q, *J* = 321.0 Hz), 118.5 (q, *J* = 321.0 Hz), 117.7, 116.1.
HRMS (ESI-QTOF) *m/z*: [M + Na]$^+$ Calcd for $C_{16}H_7F_6NO_7S_2Na$ 525.9460; Found 525.9461.

**1r**

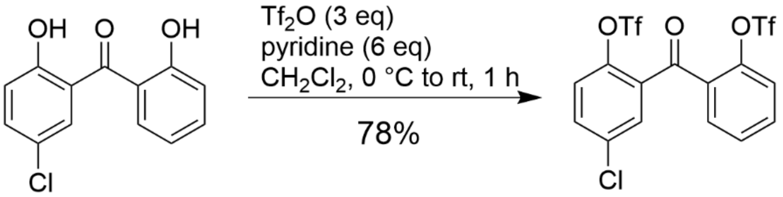


**1r**

Prepared according to the general procedure from 5-chloro-2,2'-dihydroxybenzophenone (311 mg, 1.25 mmol; known compound [8]). The product was isolated by flash column chromatography (25 g Interchim SiHP 30 µm cartridge, gradient 5% to 60% EtOAc/hexane) and freeze-dried from 1,4-dioxane to give 500 mg (78%) of **1r** as white solid.

$^1$H NMR (400 MHz, $CDCl_3$): δ 7.74 – 7.67 (m, 2H), 7.65 – 7.61 (m, 2H), 7.57 – 7.51 (m, 1H), 7.44 – 7.39 (m, 1H), 7.36 – 7.32 (m, 1H).
$^{19}$F NMR (376 MHz, $CDCl_3$): δ -72.95, -73.04.
$^{13}$C NMR (101 MHz, $CDCl_3$): δ 187.9, 147.4, 145.5, 134.8, 134.7, 134.0, 133.1, 132.6, 132.1, 131.1, 128.7, 124.0, 122.8, 118.61 (q, $J$ = 320.9 Hz), 118.55 (q, $J$ = 321.0 Hz).
HRMS (ESI-QTOF) *m/z*: $[M + Na]^+$ Calcd for $C_{15}H_7ClF_6O_7S_2Na$ 534.9118; Found 534.9121.

**1s**

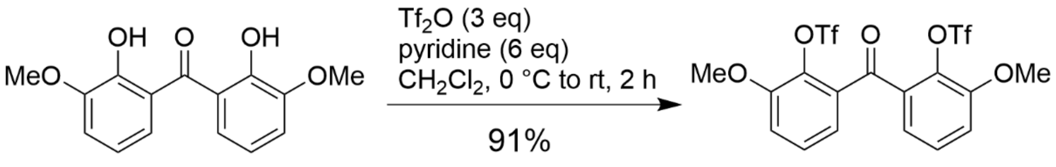


**1s**

Prepared according to the general procedure from 2,2'-dihydroxy-3,3'-dimethoxybenzophenone (318 mg, 1.16 mmol; known compound [6]) and pyridine (0.57 mL, 6.96 mmol, 6 equiv), reaction time 2 h. The product was isolated by flash column chromatography (25 g Interchim SiHP 30 µm cartridge, gradient 10 to 80% EtOAc/hexane) and freeze-dried from 1,4-dioxane to give 567 mg (91%) of **1s** as yellowish solid.

$^1$H NMR (400 MHz, $CDCl_3$): δ 7.38 (dd, J = 8.4, 7.8 Hz, 2H), 7.25 (dd, J = 8.4, 1.5 Hz, 2H), 7.12 (dd, J = 7.8, 1.5 Hz, 2H), 3.96 (s, 6H).
$^{19}$F NMR (376 MHz, $CDCl_3$): δ -73.49.
$^{13}$C NMR (101 MHz, $CDCl_3$): δ 189.2, 151.9, 136.9, 132.7, 128.6, 123.4, 118.7 (q, $J$ = 320.1 Hz), 117.1, 56.7.
HRMS (ESI-QTOF) *m/z*: $[M + Na]^+$ Calcd for $C_{17}H_{12}F_6O_9S_2Na$ 560.9719; Found 560.9717.

**1t**

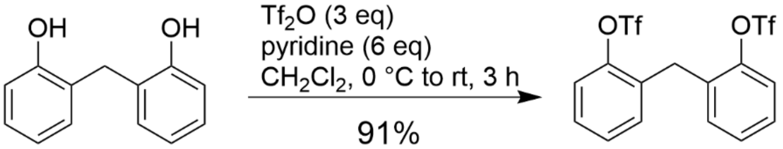


**1t**

Prepared according to the general procedure from bis(2-hydroxyphenyl)methane (500 mg, 2.50 mmol). The product was isolated by flash column chromatography (25 g Interchim SiHP 30 µm cartridge, gradient 5% to 40% EtOAc/hexane) and to give 1.06 g (91%) of **1t** as colorless oil.

$^1$H NMR (400 MHz, $CDCl_3$): δ 7.39 – 7.29 (m, 6H), 7.19 – 7.12 (m, 2H), 4.19 (s, 2H).
$^{19}$F NMR (376 MHz, $CDCl_3$): δ -73.74.

$^{13}C$ NMR (101 MHz, $CDCl_3$): δ 148.1, 131.9, 131.5, 129.0, 128.7, 121.7, 118.7 (q, $J$ = 320.2 Hz), 30.0.
HRMS (ESI-QTOF) *m/z*: $[M + NH_4]^+$ Calcd for $C_{15}H_{14}F_6NO_6S_2$ 482.0161; Found 482.0165.

## Preparation of thioxanthone 10,10-dioxides 2a-o

### General procedure for Pd-catalyzed sulfonylative homocoupling

A small (5-25 mL) round-bottom flask or a 10 mL microwave vial (Biotage) was loaded with the corresponding 2,2'-dihydroxybenzophenone bis(trifluoromethanesulfonate) (0.5 mmol, 1 equiv.), $Pd(dppf)Cl_2{\cdot}CH_2Cl_2$ catalyst (5 mol%, 0.025 mmol, 20 mg) and sodium dithionite (1.5 equiv., 0.75 mmol, 131 mg). Dry DMSO solvent (0.25 M, 2 mL) was added, the reaction mixture was degassed on a Schlenk line, placed in a preheated 80 °C silicone oil bath and stirred for 8 h (the color of the reaction mixture changes from orange to yellow and finally to light brown). Upon cooling down to rt, the mixture was diluted with brine (50 mL), extracted with $CH_2Cl_2$ (3×20 mL) and the combined extracts were dried over $Na_2SO_4$. The product was isolated by flash column chromatography (12 g Interchim SiHP 30 μm cartridge, EtOAc/hexane) and freeze-dried from 1,4-dioxane, unless indicated otherwise in individual preparations.

**2a**

1)

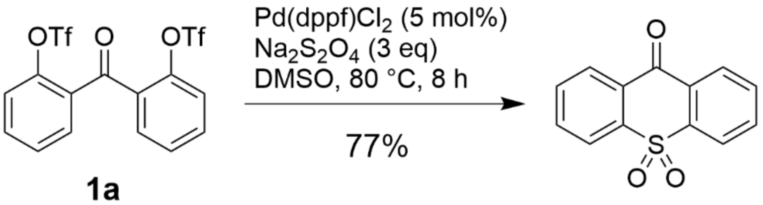


**1a** **2a**

The reaction was performed according to the general synthetic procedure from 2,2'-dihydroxybenzophenone bis(trifluoromethanesulfonate) **1a** (478 mg, 1 mmol; known compound [10]) using 3 equiv (522 mg, 3 mmol) of $Na_2S_2O_4$. Yield of **2a**: 187 mg (77%), white solid.

2)

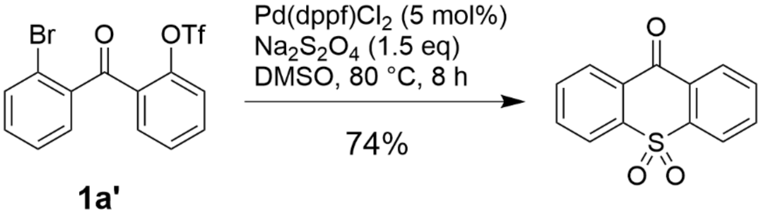


**1a'** **2a**

Following the same general synthetic procedure, the same compound **2a** was prepared from **1a'** (50 mg, 0.122 mmol) in 0.5 mL DMSO, yield 22 mg (74%).

3)

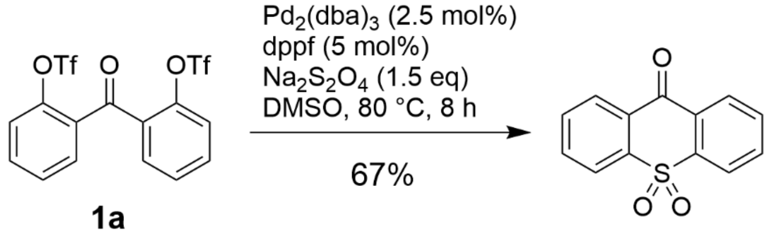


**1a** **2a**

A 10 mL microwave vial (Biotage) was loaded with **1a** (120 mg, 0.25 mmol), $Pd_2(dba)_3$ (5.7 mg, 6.25 μmol, 2.5 mol% = 5 mol% Pd), 1,1'-bis(diphenylphosphino)ferrocene (dppf; 6.9 mg, 12.5 μmol, 5 mol%) and sodium dithionite (65 mg, 0.375 mmol, 1.5 equiv.). Dry DMSO solvent (1 mL) was added, the reaction mixture was degassed on a Schlenk line, placed in a preheated 80 °C silicone oil bath and stirred for 8 h. Upon cooling down to rt, the mixture was diluted with brine (50 mL), extracted with $CH_2Cl_2$ (3×20 mL) and the combined extracts were dried over $Na_2SO_4$. The product was isolated by flash column chromatography (12 g Interchim SiHP 30 μm cartridge, 0% to 50% EtOAc/hexane + 20% $CH_2Cl_2$ constant additive) and freeze-dried from 1,4-dioxane to give 41 mg (67%) of **2a** as white solid.

$^1$H NMR (400 MHz, $CDCl_3$): δ 8.35 (ddd, J = 7.9, 1.4, 0.5 Hz, 2H), 8.21 – 8.16 (ddd, J = 7.9, 1.4, 0.5 Hz, 2H), 7.89 (td, J = 7.7, 1.4 Hz, 2H), 7.80 (td, J = 7.7, 1.4 Hz, 2H).
$^{13}$C NMR (101 MHz, $CDCl_3$): δ 178.5, 141.1, 134.8, 133.4, 130.8, 129.3, 123.7.
HRMS (ESI-QTOF) *m/z*: $[M + H]^+$ Calcd for $C_{13}H_9O_3S$ 245.0267; Found 245.0265.

**2b**

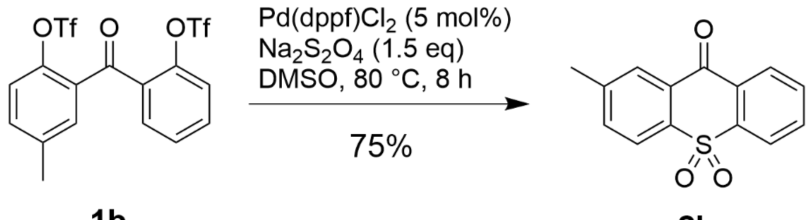


**1b** **2b**

The reaction was performed according to the general synthetic procedure from **1b** (148 mg, 0.3 mmol), yield of **2b**: 58 mg (75%), off-white solid.

$^1$H NMR (400 MHz, $CDCl_3$): δ 8.33 (dd, J = 7.8, 1.4 Hz, 1H), 8.17 (dd, J = 7.7, 1.3 Hz, 1H), 8.15 – 8.12 (m, 1H), 8.07 (d, J = 8.0 Hz, 1H), 7.87 (td, J = 7.7, 1.4 Hz, 1H), 7.78 (td, J = 7.8, 1.3 Hz, 1H), 7.67 (ddd, J = 8.0, 1.8, 0.8 Hz, 1H), 2.54 (d, J = 0.8 Hz, 3H).
$^{13}$C NMR (101 MHz, $CDCl_3$): δ 178.8, 144.5, 141.3, 138.3, 135.4, 134.7, 133.3, 130.9, 130.7, 129.6, 129.3, 123.8, 123.6, 21.8.

HRMS (ESI-QTOF) *m/z*: $[M + H]^+$ Calcd for $C_{14}H_{11}O_3S$ 259.0423; Found 259.0420.

**2c**

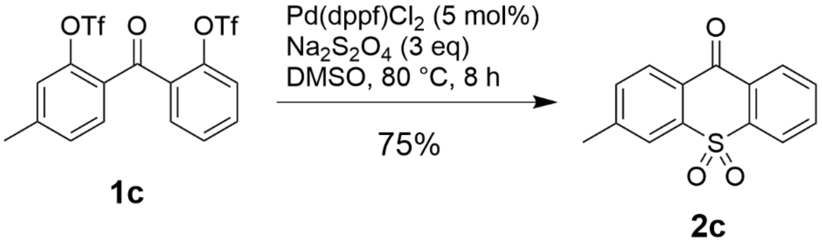

**1c**

**2c**

The reaction was performed according to the general synthetic procedure from **1c** (370 mg, 0.75 mmol) using 3 equiv (392 mg, 2.25 mmol) of $Na_2S_2O_4$. Yield of **2c**: 145 mg (75%), white solid.

$^1H$ NMR (400 MHz, $CDCl_3$): δ 8.35 (dd, J = 7.9, 1.4 Hz, 1H), 8.24 (d, J = 8.1 Hz, 1H), 8.17 (dd, J = 7.8, 1.3 Hz, 1H), 8.00 – 7.95 (m, 1H), 7.87 (td, J = 7.6, 1.4 Hz, 1H), 7.79 (td, J = 7.7, 1.3 Hz, 1H), 7.58 (ddq, J = 8.1, 1.7, 0.8 Hz, 1H), 2.56 (s, 3H).
$^{13}C$ NMR (101 MHz, $CDCl_3$): δ 178.2, 146.7, 141.1, 141.0, 134.6, 134.2, 133.3, 130.8, 129.5, 129.2, 128.3, 123.8, 123.6, 22.1.
HRMS (ESI-QTOF) *m/z*: $[M + H]^+$ Calcd for $C_{14}H_{11}O_3S$ 259.0423; Found 259.0424.

**2d**

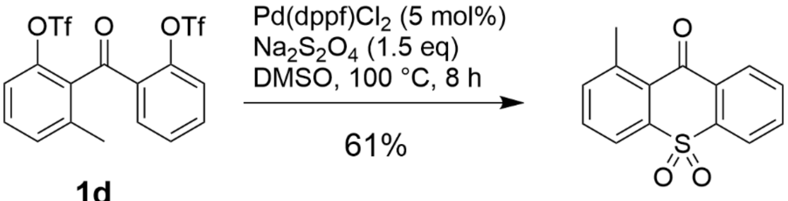

**1d**

**2d**

The reaction was performed according to the general synthetic procedure from **1d** (148 mg, 0.3 mmol) at 100 °C. Yield of **2d**: 47 mg (61%), white solid.

$^1H$ NMR (400 MHz, $CDCl_3$): δ 8.18 – 8.12 (m, 2H), 8.09 (ddd, J = 7.7, 1.3, 0.6 Hz, 1H), 7.83 – 7.74 (m, 2H), 7.68 (t, J = 7.8 Hz, 1H), 7.57 (ddq, J = 7.8, 1.4, 0.7 Hz, 1H), 2.76 (s, 3H).
$^{13}C$ NMR (101 MHz, $CDCl_3$): δ 181.8, 143.0, 142.1, 139.5, 137.3, 134.0, 133.6, 133.5, 133.2, 130.3, 129.1, 123.1, 122.1, 23.0.
HRMS (ESI-QTOF) *m/z*: $[M + H]^+$ Calcd for $C_{14}H_{11}O_3S$ 259.0423; Found 259.0423.

**2e**

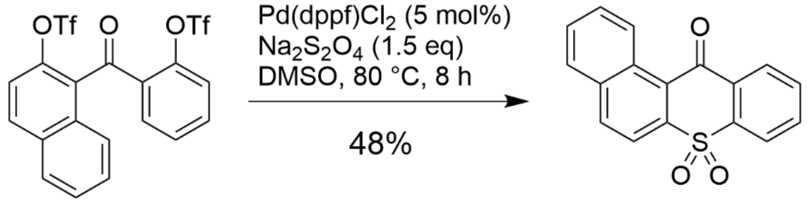


**1e** **2e**

The reaction was performed according to the general synthetic procedure from **1e** (106 mg, 0.2 mmol). Yield of **2e**: 28 mg (48%), yellow solid.

$^1$H NMR (400 MHz, $CDCl_3$): δ 9.20 (dd, J = 8.8, 1.0 Hz, 1H), 8.30 (d, J = 8.9 Hz, 1H), 8.27 – 8.24 (m, 1H), 8.21 (d, J = 8.6 Hz, 1H), 8.19 – 8.15 (m, 1H), 7.98 – 7.94 (m, 1H), 7.86 – 7.76 (m, 3H), 7.70 (ddd, J = 8.1, 6.8, 1.2 Hz, 1H).

$^{13}$C NMR (101 MHz, $CDCl_3$): δ 182.2, 141.1, 139.2, 136.0, 135.7, 134.3, 133.7, 133.6, 130.8, 130.2, 129.2, 129.1, 128.5, 127.8, 123.2, 118.4.

HRMS (ESI-QTOF) *m/z*: $[M + H]^+$ Calcd for $C_{17}H_{11}O_3S$ 295.0423; Found 295.0422.

**2f**

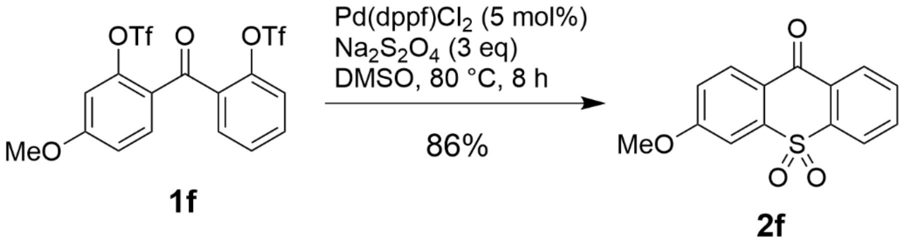


**1f** **2f**

The reaction was performed according to the general synthetic procedure from **1f** (508 mg, 1 mmol) using 3 equiv (522 mg, 3 mmol) of $Na_2S_2O_4$. Yield of **2f**: 236 mg (86%), white solid.

$^1$H NMR (400 MHz, $CDCl_3$): δ 8.36 (ddd, J = 7.7, 1.4, 0.5 Hz, 1H), 8.31 (d, J = 8.9 Hz, 1H), 8.17 (ddd, J = 7.8, 1.4, 0.5 Hz, 1H), 7.86 (td, J = 7.6, 1.4 Hz, 1H), 7.79 (td, J = 7.6, 1.3 Hz, 1H), 7.58 (d, J = 2.6 Hz, 1H), 7.24 (dd, J = 8.8, 2.6 Hz, 1H), 4.01 (s, 3H).

$^{13}$C NMR (101 MHz, $CDCl_3$): δ 177.3, 164.7, 143.1, 141.1, 134.4, 133.4, 132.0, 130.8, 129.3, 123.7, 123.6, 120.1, 107.3, 56.5.

HRMS (ESI-QTOF) *m/z*: $[M + H]^+$ Calcd for $C_{14}H_{11}O_4S$ 275.0373; Found 275.0372.

**2g**

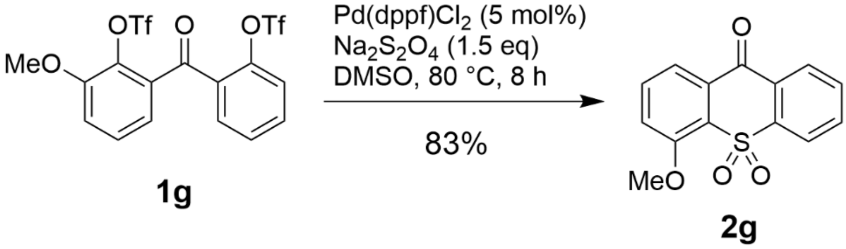

The reaction was performed according to the general synthetic procedure from **1g** (150 mg, 0.295 mmol). Yield of **2g**: 68 mg (83%), off-white solid.

$^{1}$H NMR (400 MHz, $CDCl_3$): δ 8.34 (ddd, J = 7.9, 1.4, 0.5 Hz, 1H), 8.18 (ddd, J = 7.9, 1.2, 0.5 Hz, 1H), 8.01 (dd, J = 7.9, 1.0 Hz, 1H), 7.89 (ddd, J = 7.9, 7.4, 1.3 Hz, 1H), 7.76 (ddd, J = 7.9, 7.4, 1.2 Hz, 1H), 7.71 (dd, J = 8.4, 7.9 Hz, 1H), 7.39 (dd, J = 8.4, 1.1 Hz, 1H), 4.11 (s, 3H).
$^{13}$C NMR (101 MHz, $CDCl_3$): δ 178.0, 157.5, 143.3, 135.2, 134.1, 132.9, 131.6, 129.1, 128.7, 128.4, 123.8, 121.3, 118.3, 57.4.
HRMS (ESI-QTOF) *m/z*: $[M + H]^+$ Calcd for $C_{14}H_{11}O_4S$ 275.0373; Found 275.0373.

**2h**

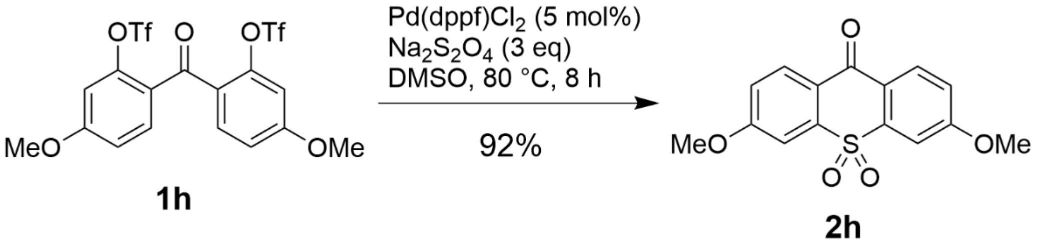

The reaction was performed according to the general synthetic procedure from **1h** (10.22 g, 19 mmol) using 3 equiv (9.92 g, 57 mmol) of $Na_2S_2O_4$ and 5 mol% (667 mg, 0.95 mmol) of $Pd(dppf)Cl_2{\cdot}CH_2Cl_2$ in 80 mL DMSO in a 250 mL round-bottom flask. On cooling to rt, the reaction mixture was diluted with EtOAc and poured into water (800 mL), the product was extracted with EtOAc (5×100 mL) and $CH_2Cl_2$ (5×100 mL). The combined extracts were washed with brine (100 mL), dried over $Na_2SO_4$ and filtered through a 3 cm plug of silica in a large funnel (diam. 10 cm), completely eluting the product with additional $CH_2Cl_2$ and EtOAc–$CH_2Cl_2$ (1:4). The filtrate was evaporated, the residue was heated to boiling with EtOAc (60 mL), diluted with hexane, allowed to cool down and left in freezer (-15 °C) overnight to complete crystallization. The product was filtered off and washed with hexane to give 5.29 g (92%) of **2h** as light cream-colored crystals.

$^{1}$H NMR (400 MHz, $CDCl_3$): δ 8.31 (d, J = 8.8 Hz, 2H), 7.56 (d, J = 2.5 Hz, 2H), 7.23 (dd, J = 8.8, 2.5 Hz, 2H), 4.00 (s, 6H).
$^{13}$C NMR (101 MHz, $CDCl_3$): δ 176.4, 164.3, 142.9, 131.8, 123.8, 120.1, 107.2, 56.4.

HRMS (ESI-QTOF) *m/z*: $[M + H]^+$ Calcd for $C_{15}H_{13}O_5S$ 305.0478; Found 305.0475.

**2i**

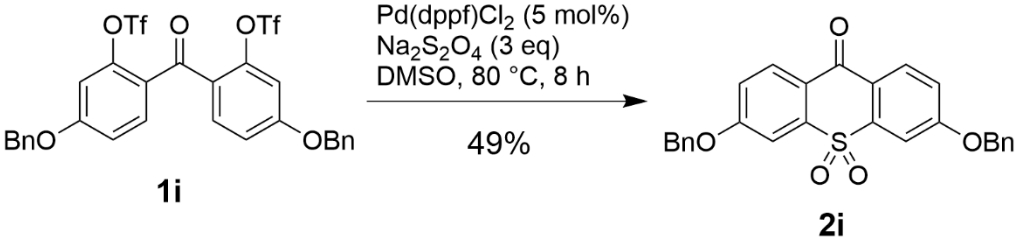


The reaction was performed according to the general synthetic procedure from **1i** (400 mg, 0.58 mmol) using 3 equiv (303 mg, 1.74 mmol) of $Na_2S_2O_4$. Yield of **2i**: 129 mg (49%), light tan solid.

$^1$H NMR (400 MHz, $CDCl_3$): δ 8.30 (d, J = 8.8 Hz, 2H), 7.66 (d, J = 2.5 Hz, 2H), 7.48 – 7.35 (m, 10H), 7.28 (dd, J = 8.8, 2.5 Hz, 2H), 5.24 (s, 4H).
$^{13}$C NMR (101 MHz, $CDCl_3$): δ 176.3, 163.4, 142.9, 135.1, 131.9, 129.0, 128.8, 127.9, 123.9, 120.5, 108.3, 71.2.
HRMS (ESI-QTOF) *m/z*: $[M + H]^+$ Calcd for $C_{27}H_{21}O_5S$ 457.1104; Found 457.1106.

**2j**

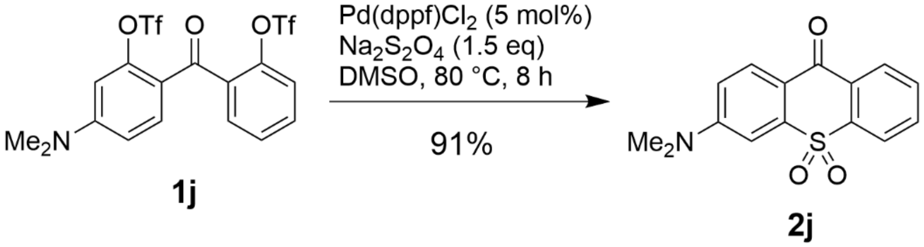


The reaction was performed according to the general synthetic procedure from **1j** (148 mg, 0.28 mmol). Yield of **2j**: 74 mg (91%), bright yellow solid.

$^1$H NMR (400 MHz, $CDCl_3$): δ 8.38 – 8.33 (m, 1H), 8.21 (d, J = 9.1 Hz, 1H), 8.16 – 8.12 (m, 1H), 7.80 (td, J = 7.5, 1.6 Hz, 1H), 7.75 (td, J = 7.6, 1.5 Hz, 1H), 7.23 (d, J = 2.7 Hz, 1H), 6.89 (dd, J = 9.1, 2.7 Hz, 1H), 3.19 (s, 6H).
$^{13}$C NMR (101 MHz, $CDCl_3$): δ 176.5, 153.8, 142.9, 140.9, 133.6, 133.1, 131.7, 131.4, 128.9, 123.4, 118.1, 114.9, 104.3, 40.4.
HRMS (ESI-QTOF) *m/z*: $[M + H]^+$ Calcd for $C_{15}H_{14}NO_3S$ 288.0689; Found 288.0687.

**2k**

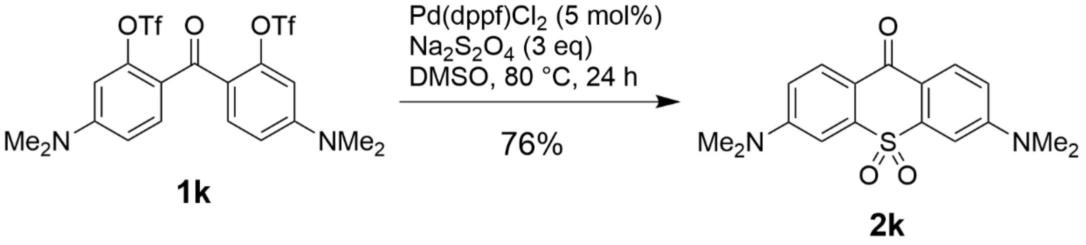


The reaction was performed according to the general synthetic procedure from **1k** (282 mg, 0.5 mmol) using 3 equiv (261 mg, 1.50 mmol) of $Na_2S_2O_4$, reaction time 24 h. Yield of **2k**: 125 mg (76%), bright yellow crystals (known compound [11]).

$^1$H NMR (400 MHz, $CDCl_3$): δ 8.21 (d, *J* = 9.0 Hz, 2H), 7.21 (d, *J* = 2.7 Hz, 2H), 6.88 (dd, *J* = 9.0, 2.7 Hz, 2H), 3.16 (s, 12H).
$^{13}$C NMR (101 MHz, $CDCl_3$): δ 175.8, 153.2, 142.7, 131.2, 118.9, 114.9, 104.2, 40.4.

**2l**

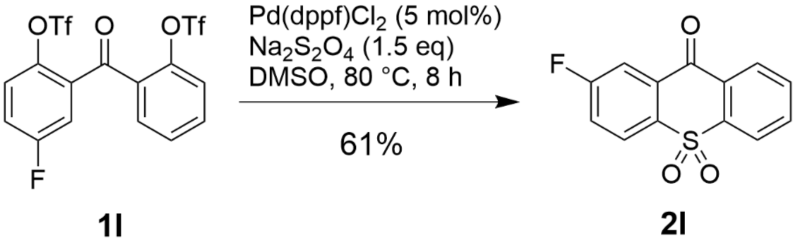


The reaction was performed according to the general synthetic procedure from **1l** (99 mg, 0.20 mmol). Yield of **2l**: 32 mg (61%), white solid.

$^1$H NMR (400 MHz, $CDCl_3$): δ 8.35 (dd, J = 7.9, 1.3 Hz, 1H), 8.24 – 8.17 (m, 2H), 8.00 (dd, J = 8.7, 2.7 Hz, 1H), 7.91 (td, J = 7.7, 1.4 Hz, 1H), 7.81 (td, J = 7.7, 1.3 Hz, 1H), 7.56 (ddd, J = 8.7, 7.6, 2.7 Hz, 1H).
$^{19}$F NMR (376 MHz, $CDCl_3$): δ -101.94.
$^{13}$C NMR (101 MHz, $CDCl_3$): δ 177.5 (d, J = 1.6 Hz), 165.2 (d, J = 257.5 Hz), 141.1, 137.3 (d, J = 3.8 Hz), 135.1, 133.6 (d, J = 7.6 Hz), 133.5, 130.5 (d, J = 1.4 Hz), 129.5, 126.9 (d, J = 8.8 Hz), 123.8, 122.2 (d, J = 23.1 Hz), 116.2 (d, J = 24.0 Hz).
HRMS (ESI-QTOF) *m/z*: $[M + H]^+$ Calcd for $C_{13}H_8FO_3S$ 263.0173; Found 263.0174.

**2m and 2m'**

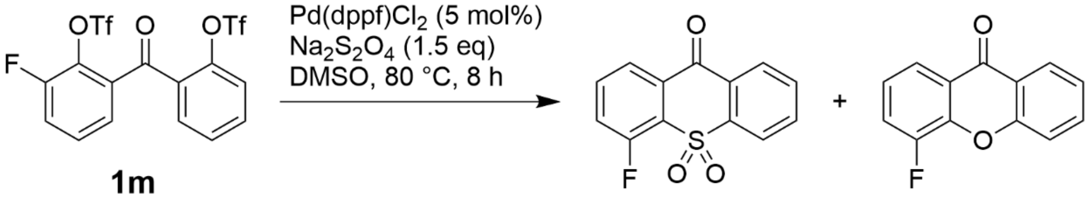


**1m** **2m** (14%) **2m'** (70%)

The reaction was performed according to the general synthetic procedure from **1m** (149 mg, 0.30 mmol). Yield of **2m**: 11 mg (14%), light yellow solid. The major product (the corresponding xanthone **2m'**, less polar) was also isolated as yellowish solid, yield 45 mg (70%).

**2m:**

$^1$H NMR (400 MHz, $CDCl_3$): δ 8.37 (ddd, J = 7.9, 1.4, 0.5 Hz, 1H), 8.22 (dt, J = 7.8, 0.9 Hz, 1H), 8.20 – 8.16 (m, 1H), 7.93 (td, J = 7.7, 1.3 Hz, 1H), 7.84 – 7.75 (m, 2H), 7.59 (ddd, J = 9.5, 8.3, 1.2 Hz, 1H).

$^{19}$F NMR (376 MHz, $CDCl_3$): δ -109.90.

$^{13}$C NMR (101 MHz, $CDCl_3$): δ 177.2 (d, J = 3.0 Hz), 158.9 (d, J = 260.8 Hz), 142.2, 135.4, 134.6 (d, J = 8.7 Hz), 133.4, 131.9, 129.3, 129.2, 125.3 (d, J = 3.4 Hz), 123.7, 123.1 (d, J = 20.4 Hz).

HRMS (ESI-QTOF) *m/z*: $[M + H]^+$ Calcd for $C_{13}H_8FO_3S$ 263.0173; Found 263.0173.

**2m':**

$^1$H NMR (400 MHz, $CDCl_3$): δ 8.34 (dd, J = 8.0, 1.7 Hz, 1H), 8.10 (dt, J = 8.1, 1.5 Hz, 1H), 7.77 (ddd, J = 8.7, 7.1, 1.8 Hz, 1H), 7.58 (dd, J = 8.5, 1.1 Hz, 1H), 7.51 (ddd, J = 10.5, 8.0, 1.6 Hz, 1H), 7.42 (ddd, J = 8.1, 7.1, 1.1 Hz, 1H), 7.31 (td, J = 8.0, 4.5 Hz, 1H).

$^{19}$F NMR (376 MHz, $CDCl_3$): δ -133.97.

$^{13}$C NMR (101 MHz, $CDCl_3$): δ 176.4 (d, J = 3.0 Hz), 155.8, 151.3 (d, J = 252.0 Hz), 145.1 (d, J = 11.3 Hz), 133.4, 127.0, 124.7, 123.9, 123.4 (d, J = 6.5 Hz), 121.9 (d, J = 4.1 Hz), 121.8, 120.6 (d, J = 16.9 Hz), 118.3.

HRMS (ESI-QTOF) *m/z*: $[M + H]^+$ Calcd for $C_{13}H_8FO_2$ 215.0503; Found 215.0501.

**2n**

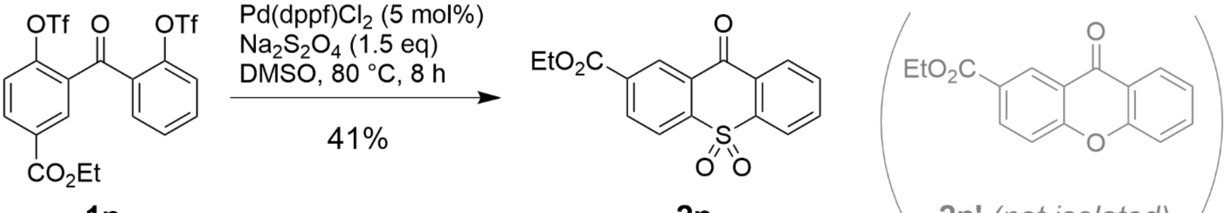


**1n** **2n** **2n'** *(not isolated)*

The reaction was performed according to the general synthetic procedure from **1n** (165 mg, 0.30 mmol). Yield of **2n**: 39 mg (41%), white solid. The corresponding xanthone (**2n'**) was identified as the major by-product in the reaction mixture but was not isolated.

$^1$H NMR (400 MHz, $CDCl_3$): δ 8.96 (dd, J = 1.7, 0.5 Hz, 1H), 8.51 (dd, J = 8.2, 1.7 Hz, 1H), 8.40 – 8.36 (m, 1H), 8.26 (dd, J = 8.2, 0.5 Hz, 1H), 8.22 – 8.18 (m, 1H), 7.91 (td, J = 7.7, 1.4 Hz, 1H), 7.83 (td, J = 7.7, 1.3 Hz, 1H), 4.48 (q, J = 7.2 Hz, 2H), 1.46 (t, J = 7.1 Hz, 3H).
$^{13}$C NMR (101 MHz, $CDCl_3$): δ 177.9, 164.3, 144.1, 140.8, 135.3, 135.2, 135.1, 133.6, 131.1, 130.6, 130.5, 129.5, 124.1, 123.8, 62.4, 14.4.
HRMS (ESI-QTOF) *m/z*: $[M + H]^+$ Calcd for $C_{16}H_{13}O_5S$ 317.0478; Found 317.0478.

## 2o and 2o'

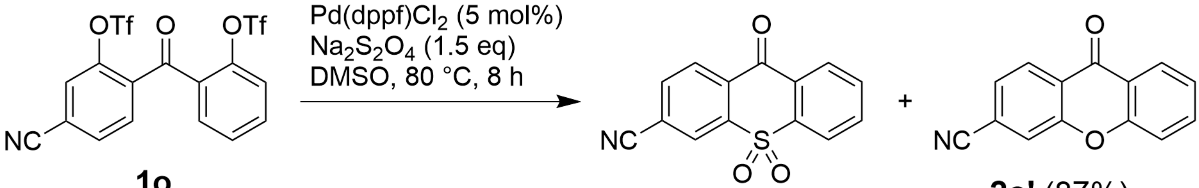


**1o** **2o** (8%) **2o'** (87%)

The reaction was performed according to the general synthetic procedure from **1o** (151 mg, 0.30 mmol) with 1.5 eq (78 mg, 0.45 mmol) of sodium dithionite. Yield of **2o**: 6.5 mg (8%), off-white solid. The major product (the corresponding xanthone **2o'**, less polar) was also isolated, yield 58 mg (87%), white solid.

**2o:**

$^1$H NMR (400 MHz, $CDCl_3$ + 10%(v/v) DMSO-$d_6$): 8.49 – 8.45 (m, 2H), 8.36 (dd, J = 7.8, 1.4 Hz, 1H), 8.20 (dd, J = 7.8, 1.2 Hz, 1H), 8.10 (dd, J = 8.2, 1.5 Hz, 1H), 7.97 (td, J = 7.7, 1.4 Hz, 1H), 7.88 (td, J = 7.7, 1.2 Hz, 1H).
$^{13}$C NMR (101 MHz, $CDCl_3$ + 10%(v/v) DMSO-$d_6$): δ 177.1, 141.8, 140.1, 136.1, 135.2, 133.7, 133.0, 130.0, 129.8, 129.4, 127.3, 123.5, 118.1, 116.0.
HRMS (ESI-QTOF) *m/z*: $[M + H]^+$ Calcd for $C_{14}H_8NO_3S$ 270.0219; Found 270.0219.

**2o':**

$^1$H NMR (400 MHz, $CDCl_3$): δ 8.43 (dd, J = 8.2, 0.6 Hz, 1H), 8.33 (dd, J = 8.0, 1.6 Hz, 1H), 7.84 (dd, J = 1.5, 0.6 Hz, 1H), 7.80 (ddd, J = 8.7, 7.1, 1.7 Hz, 1H), 7.62 (dd, J = 8.2, 1.5 Hz, 1H), 7.54 (dd, J = 8.4, 1.0 Hz, 1H), 7.45 (ddd, J = 8.1, 7.1, 1.0 Hz, 1H).
$^{13}$C NMR (101 MHz, $CDCl_3$): δ 176.1, 156.2, 155.5, 135.9, 128.3, 127.0, 126.5, 125.0, 124.6, 122.8, 121.9, 118.3, 117.9, 117.5.
HRMS (ESI-QTOF) *m/z*: $[M + H]^+$ Calcd for $C_{14}H_8NO_2$ 222.0550; Found 222.0551.

## Isolation and characterization of compounds 2p, 2q and 2u

### 2p

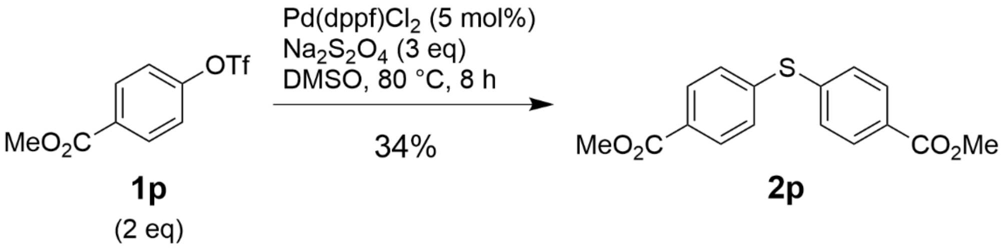


The reaction was performed according to the general synthetic procedure from **1p** (284 mg, 1 mmol; known compound [12]) using 3 equiv (528 mg, 3 mmol) of $Na_2S_2O_4$ in 1 mL DMSO. Yield of diaryl sulfide product **2p**: 51 mg (34%), white solid (analytical data match those of the known compound [13]).

$^1$H NMR (400 MHz, $CDCl_3$):  δ 8.12 – 7.93 (m, 4H), 7.44 – 7.33 (m, 4H), 3.92 (s, 6H).
$^{13}$C NMR (101 MHz, $CDCl_3$): δ 166.6, 141.0, 130.60, 130.57, 129.2, 52.4.
HRMS (ESI-QTOF) *m/z*: $[M + H]^+$ Calcd for $C_{16}H_{15}O_4S$ 303.0686; Found 303.0684.

### 2q

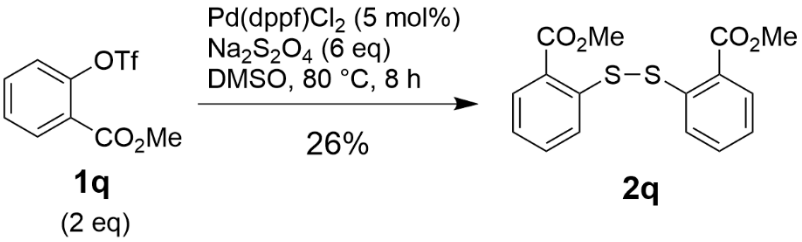


The reaction was performed according to the general synthetic procedure from **1q** (284 mg, 1 mmol; known compound [14]) using 6 equiv (1.06 g, 6 mmol) of $Na_2S_2O_4$ in 1 mL DMSO. Yield of diaryl disulfide product **2q**: 44 mg (26%), white solid (analytical data match those of the known compound [15]).

$^1$H NMR (400 MHz, $CDCl_3$): δ 8.06 (dd, J = 7.8, 1.5 Hz, 2H), 7.76 (dd, J = 8.3, 1.1 Hz, 2H), 7.41 (ddd, J = 8.3, 7.3, 1.5 Hz, 2H), 7.23 (ddd, J = 7.8, 7.3, 1.1 Hz, 2H), 3.99 (s, 6H).
$^{13}$C NMR (101 MHz, $CDCl_3$): δ 167.1, 140.5, 133.2, 131.6, 127.4, 126.0, 125.6, 52.6.
HRMS (ESI-QTOF) *m/z*: $[M + Na]^+$ Calcd for $C_{16}H_{14}O_4S_2Na$ 357.0226; Found 357.0225.

**2u**

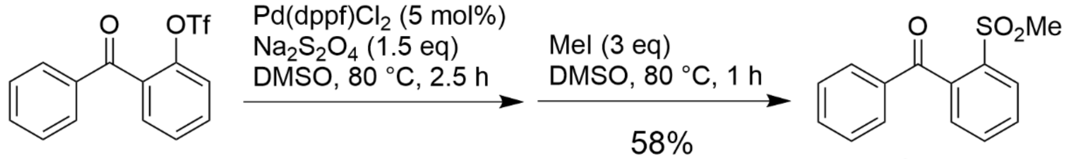


**1u** **2u**

A 10 mL microwave vial (Biotage) was loaded with **1u** (165 mg, 0.5 mmol; known compound [14]), $Pd(dppf)Cl_2 \cdot CH_2Cl_2$ catalyst (5 mol%, 0.025 mmol, 20 mg) and sodium dithionite (1.5 equiv., 0.75 mmol, 131 mg). Dry DMSO solvent (2 mL) was added, the reaction mixture was degassed on a Schlenk line, placed in a preheated 80 °C silicone oil bath and stirred for 2.5 h. Upon cooling down to rt, iodomethane (0.15 mL, 2.5 mmol, 5 equiv.) was injected through the septum, and the reaction mixture was heated at 80 °C for 1 h. The mixture was diluted with brine (50 mL), extracted with $CH_2Cl_2$ (3×20 mL) and the combined extracts were dried over $Na_2SO_4$. The product was isolated by flash column chromatography (12 g Interchim SiHP 30 µm cartridge, gradient 10% to 80% EtOAc/hexane) to give 75 mg (58%) of **2u** as light tan solid (analytical data match those of the known compound [16]).

$^1$H NMR (400 MHz, $CDCl_3$): δ 8.20 – 8.13 (m, 1H), 7.84 – 7.78 (m, 2H), 7.73 – 7.66 (m, 2H), 7.61 (ddt, $J$ = 8.7, 7.0, 1.3 Hz, 1H), 7.50 – 7.44 (m, 2H), 7.42 – 7.37 (m, 1H), 3.28 (s, 3H).
$^{13}$C NMR (101 MHz, $CDCl_3$): δ 196.4, 140.3, 139.5, 136.4, 134.1, 133.1, 130.6, 130.3, 130.1, 128.7, 128.5, 46.4.
HRMS (ESI-QTOF) *m/z*: [M + H]$^+$ Calcd for $C_{14}H_{13}O_3S$ 261.0580; Found 261.0581.

## Reaction of the oxidative addition complex (i) with sodium dithionite

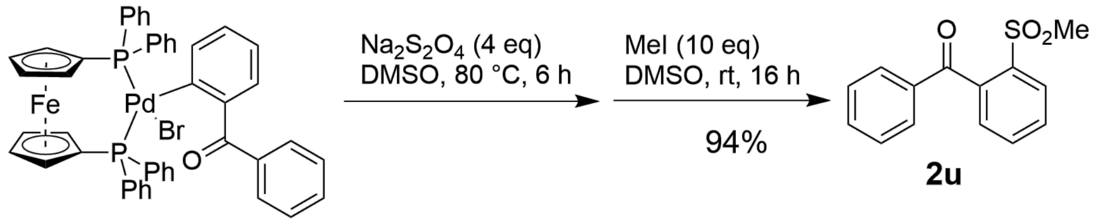


**S11**

Sodium dithionite (140 mg, 0.8 mmol, 4 equiv) was added in two portions to a degassed suspension of arylpalladium(II) complex **S11** (177 mg, 0.19 mmol; prepared from 2-bromobenzophenone, bis(tri-*o*-tolylphosphine)palladium(0) and dppf as described in [17]) in dry DMSO (1.5 mL), stirred at 80 °C under argon. The insoluble light yellow complex quickly dissolved on heating, and the color of the mixture changed to orange and finally to orange-brown. The formation of arylsulfinic acid was confirmed by LC-MS analysis: $t_{\mathbf{S11}}$ = 7.11 min, *m/z*(+) = 841.0, ArPd(dppf)$^+$; $t_{ArSO2H}$ = 4.15 min, *m/z*(-) = 245.1, $ArSO_2^-$. The reaction mixture

was cooled to rt, excess iodomethane (0.12 mL, 1.9 mmol, 10 equiv.) was injected and the mixture was left stirring overnight (16 h). The mixture was diluted with brine (50 mL), extracted with $CH_2Cl_2$ (3×20 mL) and the combined extracts were dried over $Na_2SO_4$. The product was purified by flash column chromatography (12 g Interchim SiHP 30 µm cartridge, gradient 50% to 100% $CH_2Cl_2$/hexane) twice to separate the closely eluted Pd-containing impurity and dried *in vacuo* to give 46 mg (94%) of **2u** as brown solid (NMR and MS data match those of **2u** sample from the catalytic reaction above).

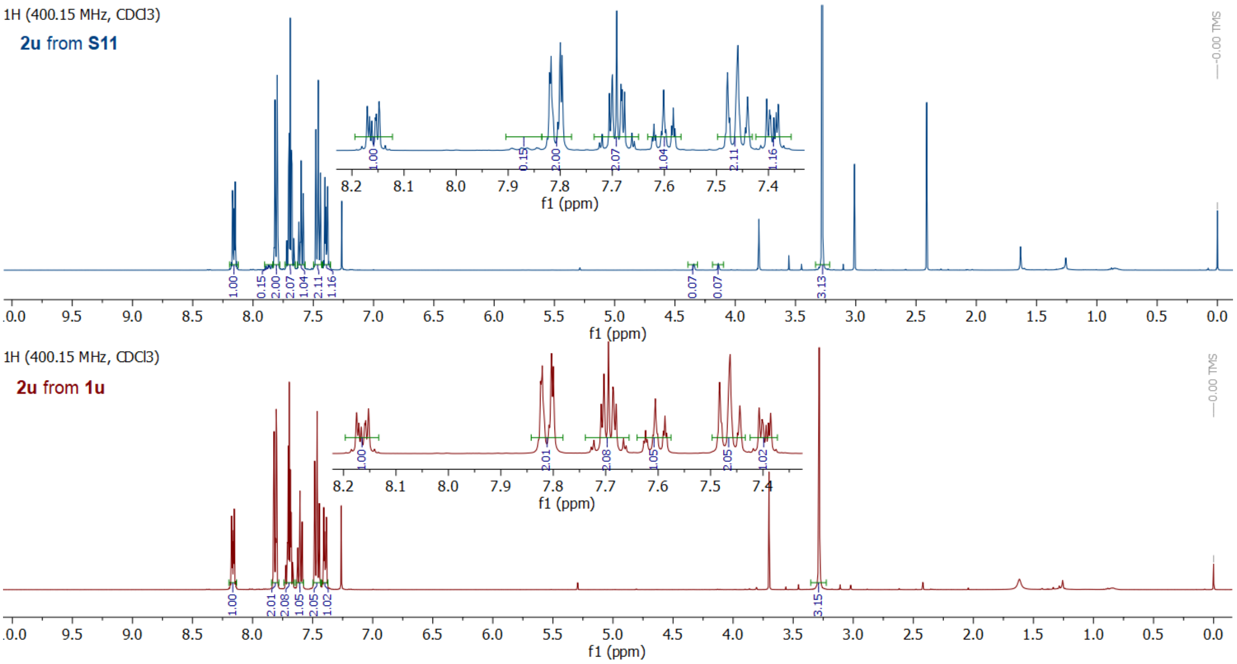


## Compound 3 and its *O*-protected derivatives 4a-g

### 3

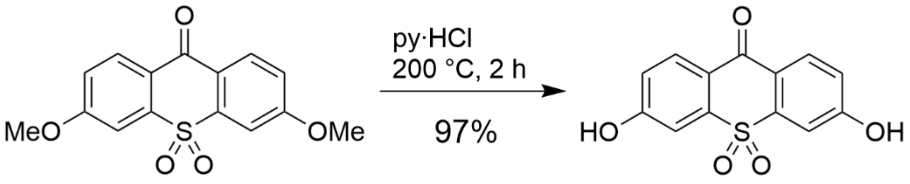


Solid **2h** (5.29 g, 17.4 mmol) and pyridine hydrochloride (30 g, 259 mmol, ~15 equiv.) were placed in a 250 mL round-bottom flask equipped with a stirring bar and capped with a rubber septum pierced with a 20G needle and stirred at 200 °C (Heat-On block, part No. RR61041, Radleys, United Kingdom) for 2 h. On cooling, the solidified residue was treated with 0.5 N HCl (150 mL) and EtOAc (75 mL), the layers were separated and the aqueous layer was extracted with EtOAc (2×50 mL). The combined extracts were washed with brine (150 mL), dried over $Na_2SO_4$, filtered and evaporated. To separate the minor colored impurity, the residue was heated

to boiling with EtOAc (80 mL), diluted gradually with hexane (40 mL) and left in freezer overnight to complete crystallization. The product (4.38 g) was filtered off, and the filtrate was evaporated and the residue was recrystallized from EtOAc–hexane to give the second crop (0.29 g) of pure material. The combined yield of **3** is 4.67 g (97%), cream-colored solid.

$^{1}$H NMR (400 MHz, DMSO-$d_6$): δ 11.52 (br.s, 2H), 8.15 (d, *J* = 8.7 Hz, 2H), 7.38 (d, *J* = 2.4 Hz, 2H), 7.23 (dd, *J* = 8.7, 2.4 Hz, 2H).
$^{13}$C NMR (101 MHz, DMSO-$d_6$): δ 175.5, 163.1, 142.3, 131.9, 121.5, 120.9, 108.7.
HRMS (ESI-QTOF) *m/z*: $[M + H]^+$ Calcd for $C_{13}H_9O_5S$ 277.0165; Found 277.0165.

**4a**

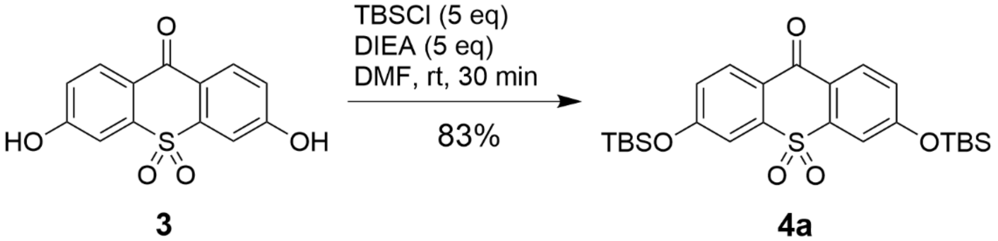


A mixture of **3** (500 mg, 1.81 mmol), imidazole (616 mg, 9.06 mmol, 5 equiv.) and *tert*-butyldimethylsilyl chloride (TBSCl; 1.37 g, 9.06 mmol, 5 equiv.) in dry DMF (20 mL) was stirred at rt for 30 min. It was then diluted with water (50 mL), the precipitated crude product was filtered off, washed with water and dried *in vacuo*. The product was isolated by flash column chromatography (25 g Interchim SiHP 30 µm cartridge, gradient 0% to 20% EtOAc/hexane) to give 760 mg (83%) of **2a** as white solid.

$^{1}$H NMR (400 MHz, $CDCl_3$): δ 8.26 (d, *J* = 8.7 Hz, 2H), 7.51 (d, *J* = 2.4 Hz, 2H), 7.13 (dd, *J* = 8.7, 2.4 Hz, 2H), 1.01 (s, 18H), 0.31 (s, 12H).
$^{13}$C NMR (101 MHz, $CDCl_3$): δ 176.7, 161.4, 142.9, 132.0, 124.7, 124.2, 114.4, 25.6, 18.4, -4.2.
HRMS (ESI-QTOF) *m/z*: $[M + H]^+$ Calcd for $C_{25}H_{37}O_5SSi_2$ 505.1895; Found 505.1896.

**4b**

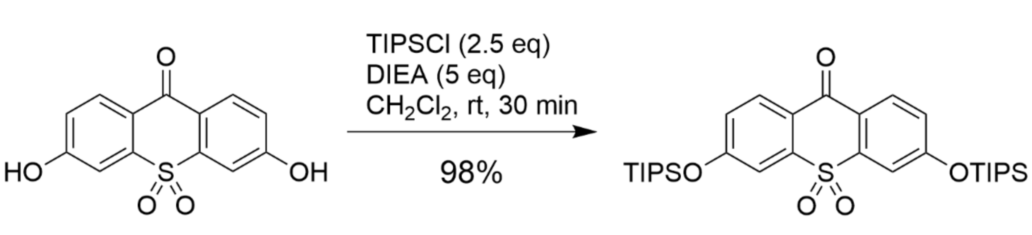


A mixture of **3** (200 mg, 0.72 mmol), triisopropylsilyl chloride (TIPSCl; 350 mg, 1.81 mmol, 2.5 equiv.) and *N*-ethyldiisopropylamine (DIEA; 0.63 mL, 3.62 mmol, 5 equiv.) in dry $CH_2Cl_2$ (5 mL)

was stirred at rt for 30 min. It was then washed with water (3×5 mL), the organic layer was separated, dried over $Na_2SO_4$, the filtrate was evaporated and dried *in vacuo* to give 416 mg (98%) of **4b** as an oil.

$^1$H NMR (400 MHz, $CDCl_3$): δ 8.25 (d, *J* = 8.7 Hz, 2H), 7.53 (d, *J* = 2.4 Hz, 2H), 7.16 (dd, *J* = 8.7, 2.4 Hz, 2H), 1.42 – 1.28 (m, 6H), 1.13 (d, *J* = 7.4 Hz, 36H).
$^{13}$C NMR (101 MHz, $CDCl_3$): δ 176.6, 161.8, 142.9, 132.0, 124.4, 124.0, 114.1, 18.0, 12.8.
HRMS (ESI-QTOF) *m/z*: $[M + H]^+$ Calcd for $C_{31}H_{49}O_5SSi_2$ 589.2835; Found 589.2835.

**4c**

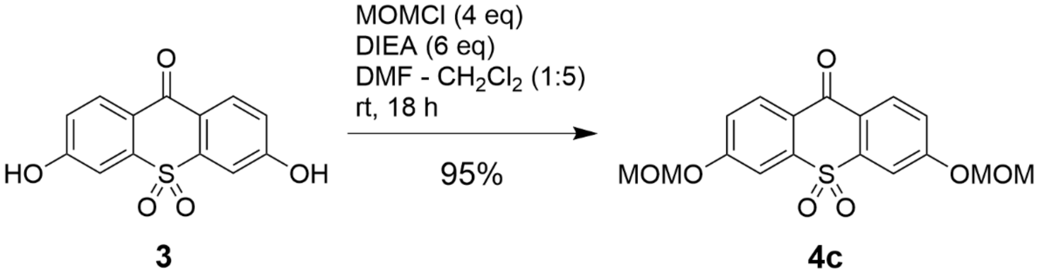


In a 50 mL round-bottom flask, placed in a rt water bath, compound **3** (439 mg, 1.59 mmol) was suspended in the mixture of *N*-ethyldiisopropylamine (DIEA; 1.66 mL, 9.54 mmol, 6 equiv.), dry $CH_2Cl_2$ (10 mL) and dry DMF (2 mL). The flask was flushed with argon and capped, and chloromethyl methyl ether (MOMCl; 0.48 mL, 6.36 mmol, 4 equiv.) was then injected dropwise. The remaining solid **3** dissolved, and the color of the reaction mixture changed from yellow to brown-orange and gradually lightened; the poorly soluble product partially precipitated overnight (18 h). The reaction mixture was poured in water (100 mL), and the product was extracted with EtOAc (2×50 mL) and $CH_2Cl_2$ (2×50 mL), the combined extracts were washed with water (100 mL), brine and dried over $Na_2SO_4$, filtered and evaporated. The residue was heated to boiling with EtOAc (30 mL), diluted gradually with hexane (50 mL) and left in freezer overnight to complete crystallization. Light tan crystals of the product were then filtered off, washed with hexane and dried *in vacuo*. The yield of **4c** is 547 mg (95%).

$^1$H NMR (400 MHz, $CDCl_3$): δ 8.31 (d, *J* = 8.8 Hz, 2H), 7.74 (d, *J* = 2.5 Hz, 2H), 7.36 (dd, *J* = 8.8, 2.5 Hz, 2H), 5.33 (s, 4H), 3.52 (s, 6H).
$^{13}$C NMR (101 MHz, $CDCl_3$): δ 176.6, 162.0, 142.9, 131.9, 124.5, 121.0, 110.3, 94.7, 56.8.
HRMS (ESI-QTOF) *m/z*: $[M + H]^+$ Calcd for $C_{17}H_{17}O_7S$ 365.0690; Found 365.0689.

**4d**

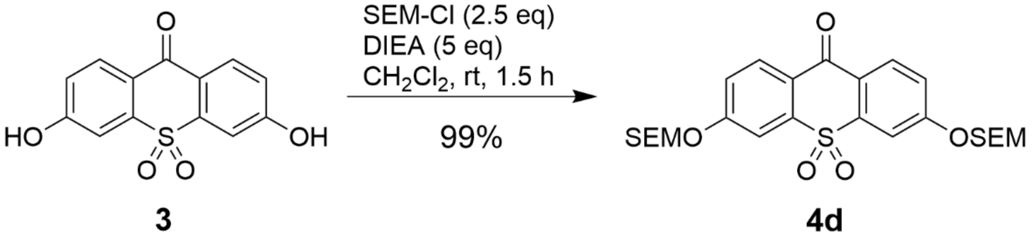


In a 50 mL round-bottom flask, placed in a rt water bath, *N*-ethyldiisopropylamine (DIEA; 1.6 mL, 9.05 mmol, 5 equiv.) was added quickly dropwise to the stirred suspension of **3** (500 mg, 1.81 mmol) and 2-(trimethylsilyl)ethoxymethyl chloride (SEM-Cl; 0.8 mL, 4.53 mmol, 2.5 equiv.) in dry $CH_2Cl_2$ (12 mL). The solids dissolved and yellow solution formed (the yellow color quickly faded). After 1.5 h, the reaction mixture was diluted with brine (50 mL), the product was extracted with $CH_2Cl_2$ (3×25 mL) and the combined extracts were dried over $Na_2SO_4$. The product was isolated by flash column chromatography (25 g Interchim SiHP 30 µm cartridge, gradient 5% to 50% EtOAc/hexane) and freeze-dried from 1,4-dioxane to give 941 mg (99%) of **4d** as white solid.

$^1$H NMR (400 MHz, $CDCl_3$): δ 8.30 (d, *J* = 8.8 Hz, 2H), 7.73 (d, *J* = 2.4 Hz, 2H), 7.36 (dd, *J* = 8.8, 2.4 Hz, 2H), 5.37 (s, 4H), 3.84 – 3.74 (m, 4H), 1.02 – 0.93 (m, 4H), 0.01 (s, 18H).
$^{13}$C NMR (101 MHz, $CDCl_3$): δ 176.6, 162.3, 142.9, 131.8, 124.4, 120.8, 110.4, 93.2, 67.4, 18.2, -1.3.
HRMS (ESI-QTOF) *m/z*: $[M + H]^+$ Calcd for $C_{25}H_{37}O_7SSi_2$ 537.1793; Found 537.1793.

**4e**

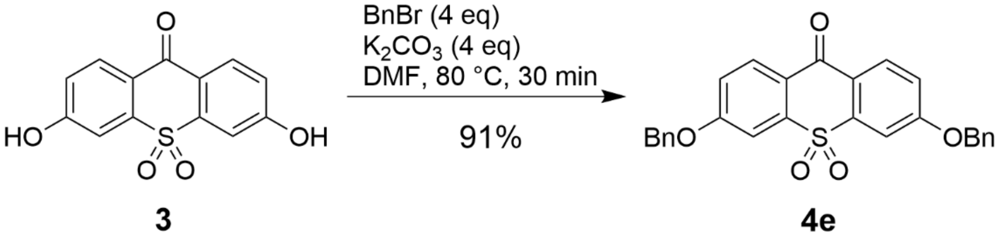


A mixture of **3** (1.00 g, 3.62 mmol), benzyl bromide (2.48 g, 14.5 mmol, 4 equiv.) and $K_2CO_3$ (2.00 g, 14.5 mmol, 4 equiv.) in DMF (50 mL) was stirred at 80 °C for 30 min. It was then diluted with water (50 mL), the precipitated product was filtered off, washed with water and dried *in vacuo* to give 1.50 g (91%) of **4e** as white solid.

$^1$H NMR (400 MHz, $CDCl_3$): δ 8.31 (d, *J* = 8.8 Hz, 2H), 7.67 (d, *J* = 2.5 Hz, 2H), 7.51 – 7.34 (m, 10H), 7.29 (dd, *J* = 8.8, 2.5 Hz, 2H), 5.24 (s, 4H).
$^{13}$C NMR (101 MHz, $CDCl_3$) δ 176.3, 163.4, 142.9, 135.1, 131.9, 129.0, 128.8, 127.9, 123.9, 120.4, 108.3, 71.2.

HRMS (ESI-QTOF) *m/z*: $[M + H]^+$ Calcd for $C_{27}H_{21}O_5S$ 457.1104; Found 457.1104.

**4f**

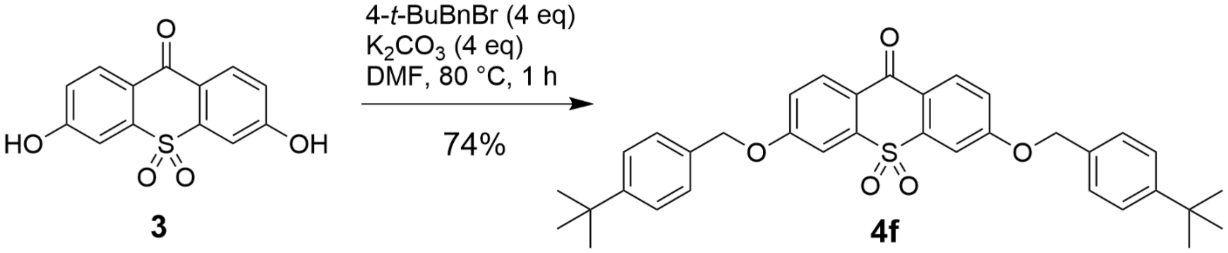


A mixture of **3** (100 mg, 0.36 mmol), 4-*tert*-butylbenzyl bromide (329 mg, 266 µL, 1.45 mmol, 4 equiv.) and $K_2CO_3$ (200 mg, 1.45 mmol, 4 equiv.) in DMF (2 mL) was stirred at 80 °C for 1 h. It was then diluted with water (50 mL), the precipitated product was filtered off, washed with water and dried *in vacuo* to give 152 mg (74%) of **4f** as white solid.

$^1H$ NMR (400 MHz, $CDCl_3$): δ 8.31 (d, *J* = 8.8 Hz, 2H), 7.67 (d, *J* = 2.5 Hz, 2H), 7.51 – 7.43 (m, 4H), 7.43 – 7.34 (m, 4H), 7.28 (dd, *J* = 8.8, 2.5 Hz, 2H), 5.20 (s, 4H), 1.34 (s, 18H).
$^{13}C$ NMR (101 MHz, $CDCl_3$): δ 176.4, 163.6, 152.0, 142.9, 132.1, 131.9, 127.9, 126.0, 123.9, 120.5, 108.3, 71.1, 34.8, 31.5.
HRMS (ESI-QTOF) *m/z*: $[M + H]^+$ Calcd for $C_{35}H_{37}O_5S$ 569.2356; Found 569.2359.

**4g**

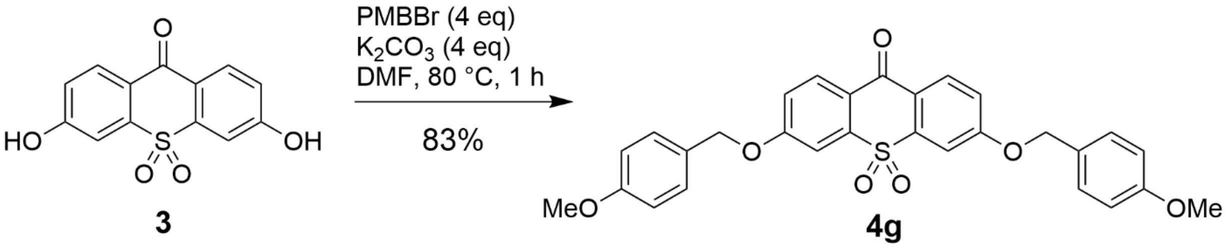


A mixture of **3** (100 mg, 0.36 mmol), 4-methoxybenzyl bromide (227 mg, 196 µL, 1.45 mmol, 4 equiv.) and $K_2CO_3$ (200 mg, 1.45 mmol, 4 equiv.) in DMF (2 mL) was stirred at 80 °C for 1 h. It was then diluted with water (50 mL), the precipitated product was filtered off, washed with water and dried *in vacuo* to give 155 mg (83%) of **4g** as white solid.

$^1H$ NMR (400 MHz, $CDCl_3$): δ 8.30 (d, *J* = 8.8 Hz, 2H), 7.65 (d, *J* = 2.5 Hz, 2H), 7.42 – 7.34 (m, 4H), 7.29 – 7.24 (m, 2H), 6.98 – 6.91 (m, 4H), 5.17 (s, 4H), 3.83 (s, 6H).
$^{13}C$ NMR (101 MHz, $CDCl_3$): δ 176.4, 163.5, 160.1, 142.9, 131.8, 129.7, 127.1, 123.8, 120.5, 114.4, 108.2, 71.1, 55.5.
HRMS (ESI-QTOF) *m/z*: $[M + H]^+$ Calcd for $C_{29}H_{25}O_7S$ 517.1316; Found 517.1326.

## Preparation of sulfone-fluoresceins 5a,b and sulfone-fluorone 5c

### 5a

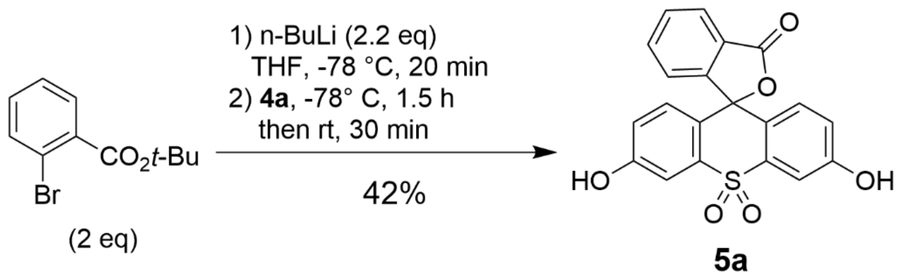


**5a**

In a flame-dried flask under argon, *tert*-butyl 2-bromobenzoate (102 mg, 0.4 mmol, 2 equiv.) was dissolved in THF (5 mL) and cooled to -78 °C (dry ice-acetone bath). *n*-Butyllithium (0.18 mL of 2.5 M in hexanes, 0.44 mmol, 2.2 equiv.) was added dropwise and the mixture was stirred for 20 min. A solution of **4a** (100 mg, 0.2 mmol) in THF (2 mL) was then added dropwise and the reaction mixture was stirred at -78 °C for 1.5 h. It was then allowed to warm up to rt and stirred for 30 min before quenching by addition of water (5 mL) and acetic acid (1 mL), extracted with EtOAc (3×25 mL) and the combined extracts were dried over $Na_2SO_4$. The product was isolated by flash column chromatography (12 g Interchim SiHP 30 µm cartridge, gradient 0% to 5% methanol/$CH_2Cl_2$) to give 51 mg of crude product. It was further purified by preparative HPLC (ThermoFisher Hypersil GOLD 250×21.2 mm 5 µm, solvent flow rate 18 mL/min, gradient 10% to 80% A:B, A – acetonitrile + 0.1% (v/v) $HCO_2H$, B – water + 0.1% (v/v) $HCO_2H$) to give 32 mg (42%) of **5a** as white solid.

$^1H$ NMR (400 MHz, $CD_3CN$): δ 8.06 (br.s, 2H), 7.98 (dt, *J* = 7.5, 1.1 Hz, 1H), 7.76 – 7.56 (m, 3H), 7.54 (d, *J* = 2.6 Hz, 2H), 7.17 (d, *J* = 8.8 Hz, 2H), 7.03 (dd, *J* = 8.8, 2.6 Hz, 2H).
$^{13}C$ NMR (101 MHz, $CD_3CN$): δ 170.6, 159.3, 153.9, 138.3, 136.8, 131.3, 130.0, 128.4, 127.0, 124.7, 124.2, 121.9, 110.5, 83.0.
HRMS (ESI-QTOF) *m/z*: $[M + H]^+$ Calcd for $C_{20}H_{13}O_6S$ 381.0427; Found 381.0430.

### 5b

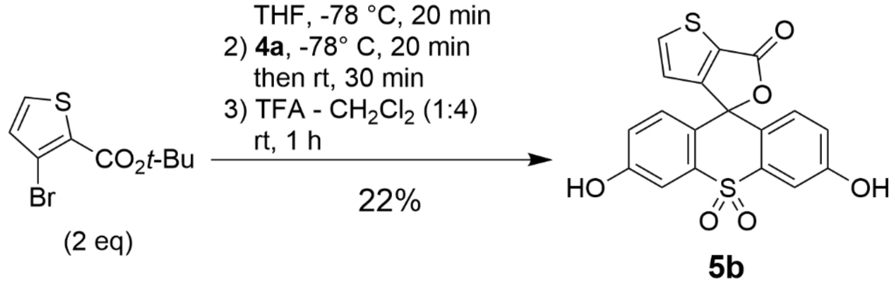


**5b**

In a flame-dried flask under argon, *tert*-butyl 3-bromothiophene-2-carboxylate (104 mg, 0.4 mmol, 2 equiv.; known compound [18]) was dissolved in THF (5 mL) and cooled to -78 °C (dry ice-acetone bath). *n*-Butyllithium (0.18 mL of 2.5 M in hexanes, 0.44 mmol, 2.2 equiv.) was added dropwise and the mixture was stirred for 20 min. A solution of **4a** (100 mg, 0.2 mmol) in THF (2 mL) was then added dropwise and the reaction mixture was stirred at -78 °C for 20 min. It was then allowed to warm up to rt and quenched by addition of water (5 mL) and acetic acid (1 mL), extracted with EtOAc (3×25 mL) and the combined extracts were dried over $Na_2SO_4$, filtered and evaporated. The crude product was dissolved in TFA-$CH_2Cl_2$ mixture (1:4, 5 mL) mixture and stirred for 1 hour before washing with water, separating the organic layer, drying over $Na_2SO_4$ and evaporating the filtrate. The product was isolated by flash column chromatography (12 g Interchim SiHP 30 µm cartridge, gradient 0% to 5% methanol/$CH_2Cl_2$) to give 48 mg of crude product. It was further purified by preparative HPLC (ThermoFisher Hypersil GOLD 250×21.2 mm 5 µm, solvent flow rate 18 mL/min, gradient 10% to 100% A:B, A – acetonitrile + 0.1% (v/v) $HCO_2H$, B – water + 0.1% (v/v) $HCO_2H$) to give 17 mg (22%) of **5b** as pink solid.

$^1$H NMR (400 MHz, $CD_3CN$): δ 8.40 – 7.58 (br.s, 2H), 7.99 (d, *J* = 4.9 Hz, 1H), 7.52 (d, *J* = 2.6 Hz, 2H), 7.37 (d, *J* = 8.8 Hz, 2H), 7.19 (d, *J* = 4.9 Hz, 1H), 7.07 (dd, *J* = 8.8, 2.6 Hz, 2H).
$^{13}$C NMR (101 MHz, $CD_3CN$) δ 166.9, 164.8, 159.3, 144.0, 138.3, 129.4, 128.4, 127.9, 122.6, 121.8, 110.9, 82.2.
HRMS (ESI-QTOF) *m/z*: $[M + H]^+$ Calcd for $C_{18}H_{11}O_6S_2$ 386.9992; Found 386.9995.

**5c**

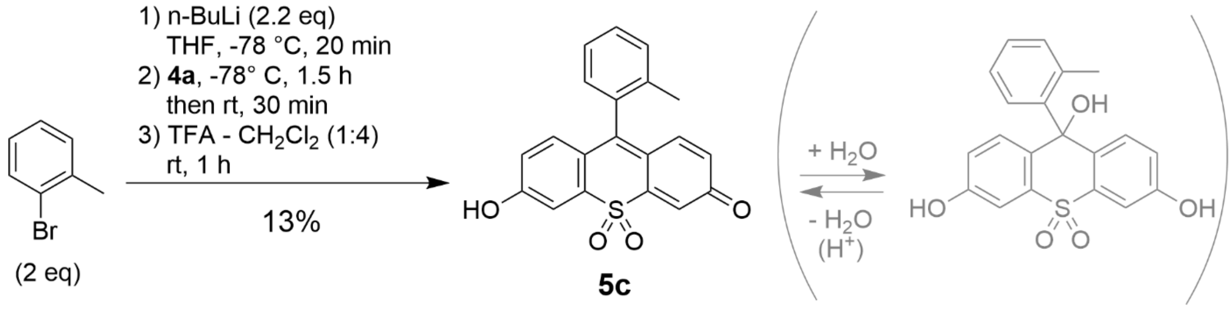


In a flame-dried flask under argon, 2-bromotoluene (48 µL, 0.4 mmol, 2 equiv.) was dissolved in THF (5 mL) and cooled to -78 °C (dry ice-acetone bath). *n*-Butyllithium (0.18 mL of 2.5 M in hexanes, 0.44 mmol, 2.2 equiv.) was added dropwise and the mixture was stirred for 20 min. A solution of **4a** (100 mg, 0.2 mmol) in THF (2 mL) was then added dropwise and the reaction mixture was stirred at -78 °C for 1.5 h. It was then allowed to warm up to rt and quenched by addition of water (5 mL) and acetic acid (1 mL), extracted with EtOAc (3×25 mL) and the combined extracts were dried over $Na_2SO_4$, filtered and evaporated. The crude product was dissolved in TFA-$CH_2Cl_2$ mixture (1:4, 5 mL) mixture and stirred for 1 hour before washing with

water, separating the organic layer, drying over $Na_2SO_4$ and evaporating the filtrate. The product was isolated by flash column chromatography (12 g Interchim SiHP 30 µm cartridge, gradient 0% to 5% methanol/$CH_2Cl_2$) to give 72 mg of crude product. It was further purified by preparative HPLC (ThermoFisher Hypersil GOLD 250×21.2 mm 5 µm, solvent flow rate 18 mL/min, gradient 10% to 60% A:B, A – acetonitrile + 0.1% (v/v) $HCO_2H$, B – water + 0.1% (v/v) $HCO_2H$) to give 9 mg (13%) of **5c** as red solid. The product undergoes reversible water addition but can be quickly reverted to the sulfone-fluorone form by dissolving it in $CH_2Cl_2$ and adding TFA to the solution, then removing the solvents.

$^1$H NMR (400 MHz, $CDCl_3$:TFA-*d* 10:1 (v/v)): δ 7.67 (br.s, 2H), 7.58 – 7.49 (m, 1H), 7.47 – 7.39 (m, 2H), 7.27 – 7.14 (m, 3H), 6.88 (br.d, *J* = 9.3 Hz, 2H), 2.11 (s, 3H).
$^{13}$C NMR (101 MHz, $CDCl_3$:TFA-*d* 10:1 (v/v)): δ 174.1, 160.3, 143.7, 140.8, 136.3, 133.2, 131.2, 130.9, 129.9, 129.7, 129.2, 126.5, 124.3, 123.4, 119.5, 19.6.
HRMS (ESI-QTOF) *m/z*: $[M + H]^+$ Calcd for $C_{20}H_{15}O_4S$ 351.0686; Found 351.0688.

## Preparation of HaloTag ligands 7a-c

**6a**

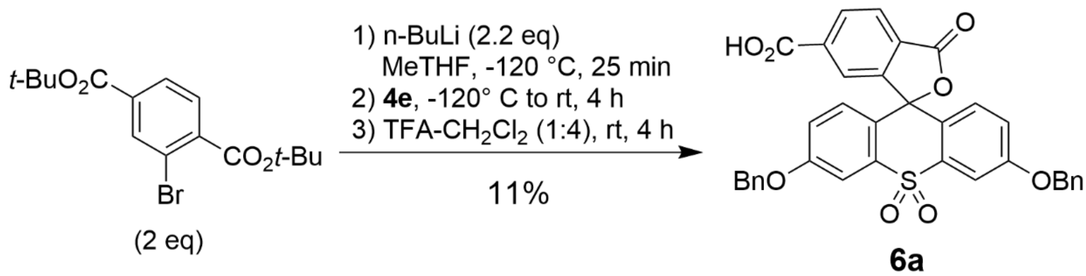


**6a**

In a flame-dried flask under argon, di-*tert*-butyl 2-bromoterephthalate (156 mg, 0.4 mmol, 2 equiv.) was dissolved in dry 2-methyltetrahydrofuran (MeTHF; 5 mL) and cooled to -120 °C (liquid $N_2$-pentane bath). *n*-Butyllithium (0.18 mL of 2.5 M in hexanes, 0.44 mmol, 2.2 equiv.) was added dropwise and the mixture was stirred for 20 min. A suspension of **4e** (100 mg, 0.2 mmol) in MeTHF (2 mL) was then added dropwise at -120 °C and the reaction mixture was allowed to slowly warm up to rt over the course of 4 h. It was then quenched by addition of water (5 mL) and acetic acid (1 mL), extracted with EtOAc (3×25 mL) and the combined extracts were dried over $Na_2SO_4$. The product was isolated by flash column chromatography (12 g Interchim SiHP 30 µm cartridge, gradient 0% to 20 % EtOAc/hexane) to give 55 mg of crude material, which was dissolved in TFA-$CH_2Cl_2$ mixture (1:4, 5 mL). The mixture was stirred at rt for 4 h before washing with water (3×25 mL), the organic layer was dried over $Na_2SO_4$, filtered and evaporated. The product was isolated by flash column chromatography (12 g Interchim SiHP 30 µm cartridge, gradient 0% to 10% methanol/$CH_2Cl_2$) to give 15 mg (11%) of **6a** as colorless oil which crystallized in the freezer.

$^1$H NMR (400 MHz, $CDCl_3$): δ 8.64 (t, *J* = 1.0 Hz, 1H), 8.21 (dd, *J* = 8.0, 1.3 Hz, 1H), 8.05 (dd, *J* = 8.0, 0.8 Hz, 1H), 7.80 (d, *J* = 2.7 Hz, 2H), 7.46 – 7.36 (m, 10H), 7.33 (d, *J* = 8.9 Hz, 2H), 7.14 (dd, *J* = 8.9, 2.7 Hz, 2H), 5.15 (s, 4H).

$^{13}$C NMR (101 MHz, $CDCl_3$): δ 169.1, 167.3, 159.7, 153.6, 136.8, 135.7, 135.4, 131.7, 128.8, 128.5, 128.1, 127.8, 127.7, 127.0, 126.4, 125.8, 121.2, 109.0, 82.4, 70.8.

HRMS (ESI-QTOF) *m/z*: $[M + H]^+$ Calcd for $C_{35}H_{25}O_8S$ 605.1265; Found 605.1267.

## 6a-Halo

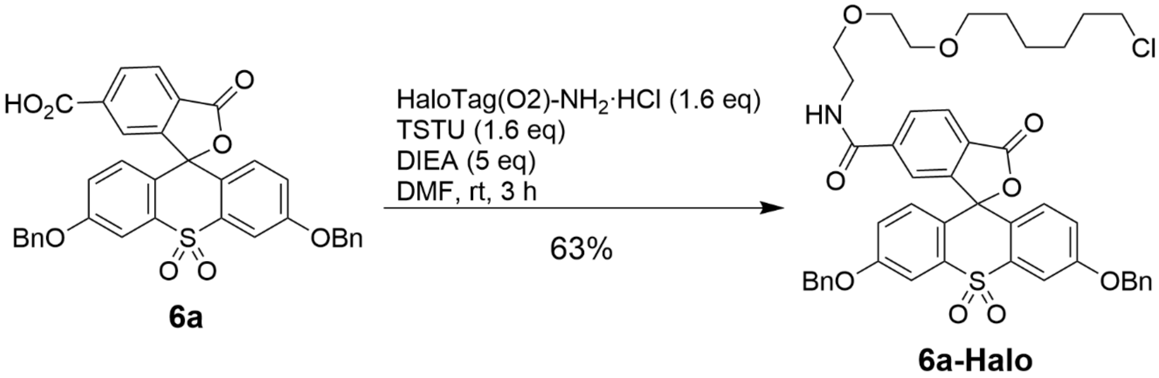


Compound **6a** (20 mg, 33 µmol) was dissolved in dry DMF (2 mL). 2-(2-(6-Chlorohexyloxy)ethoxy)ethan-1-amine hydrochloride (HaloTag(O2) amine; 11 mg, 42 µmol, 1.3 equiv; known compound [19]), *N,N,N′N′*-tetramethyl-*O*-(*N*-succinimidyl)uronium tetrafluoroborate (TSTU; 11 mg, 37 µmol, 1.1 equiv.), *N*-ethyldiisopropylamine (DIEA; 21 mg, 163 µmol, 5 equiv.) were then added, and the reaction mixture was stirred at rt for 2 h. HPLC-MS analysis showed incomplete conversion of **6a**, so additional TSTU (5 mg, 17 µmol, 0.5 equiv.) and HaloTag(O2) amine (5 mg, 19 µmol, 0.6 equiv.) were added and the stirring was continued for 1 h. The solvents were removed *in vacuo*, and the product was isolated by flash column chromatography (12 g Interchim SiHP 30 µm cartridge, gradient 20% to 80% EtOAc/hexane) to give 17 mg (63%) of **6a-Halo** as white solid.

$^{1}$H NMR (400 MHz, $CDCl_3$): δ 8.24 (t, *J* = 1.0 Hz, 1H), 8.06 – 7.99 (m, 2H), 7.78 (d, *J* = 2.7 Hz, 2H), 7.46 – 7.32 (m, 10H), 7.36 (d, *J* = 8.9 Hz, 2H), 7.15 (dd, *J* = 8.9, 2.7 Hz, 2H), 6.74 (t, *J* = 5.3 Hz, 1H), 5.16 (s, 4H), 3.69 – 3.58 (m, 4H), 3.60 – 3.54 (m, 4H), 3.48 (t, *J* = 6.7 Hz, 2H), 3.35 (t, *J* = 6.6 Hz, 2H), 1.77 – 1.63 (m, 2H), 1.51 – 1.41 (m, 2H), 1.41 – 1.32 (m, 2H), 1.32 – 1.26 (m, 2H).

$^{13}$C NMR (101 MHz, $CDCl_3$): δ 169.5, 165.7, 159.8, 153.8, 141.8, 136.8, 135.6, 130.1, 129.0, 128.7, 128.5, 128.1, 127.8, 126.7, 125.1, 121.7, 121.2, 109.2, 82.3, 71.3, 71.0, 70.6, 70.2, 69.7, 45.1, 40.2, 32.6, 29.5, 26.8, 25.5.

HRMS (ESI-QTOF) *m/z*: $[M + Na]^+$ Calcd for $C_{45}H_{44}ClNO_9SNa$ 832.2318; Found 832.2318.

**7a-Halo**

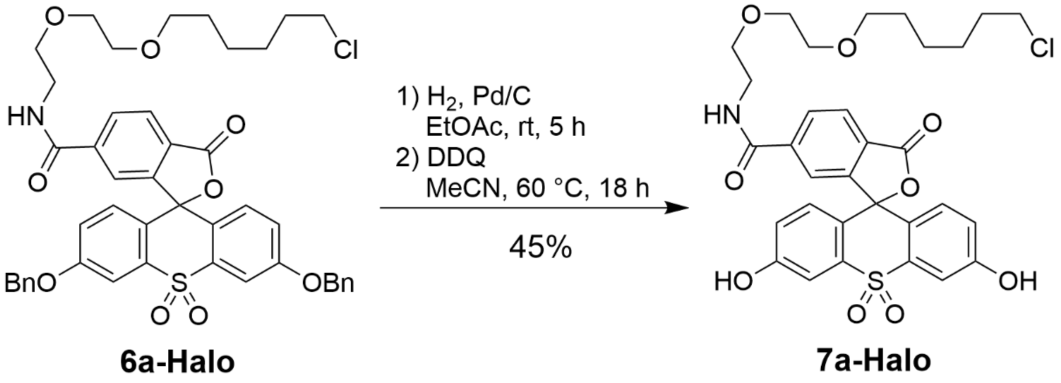


Compound **6a-Halo** (40 mg, 49 μmol) was dissolved in EtOAc (10 mL) in a 50 mL round-bottom flask. Palladium on carbon (10 mg; 10% Pd, cat. No. 205699, Sigma-Aldrich) was then added, the flask was evacuated and filled with hydrogen three times, and the mixture was vigorously stirred for 5 h. HPLC-MS analysis showed complete conversion of the starting **6a-Halo** into **7a-Halo** with significant overreduction into the corresponding triarylmethane. The supernatant was filtered through a PTFE syringe filter (0.22 μm) and evaporated. The product was isolated by preparative HPLC (ThermoFisher Hypersil GOLD 250×21.2 mm 5 μm, solvent flow rate 18 mL/min, gradient 10% to 80% A:B, A – acetonitrile + 0.1% (v/v) $HCO_2H$, B – water + 0.1% (v/v) $HCO_2H$) to give 8 mg of **7a-Halo**; the fractions containing the overreduced product (9 mg) were pooled, evaporated and treated with excess DDQ (65 mg, 20 equiv.) in acetonitrile (1 mL) in a closed vial at 60 °C overnight (18 h). The reaction mixture was diluted with water (10 mL), the product was extracted with EtOAc (3×10 mL), the combined organic layers were washed with brine and dried over $Na_2SO_4$. Additional product was isolated from this mixture by preparative HPLC (same conditions), the combined yield of **7a-Halo** – 14 mg (45%), white solid.

$^1$H NMR (400 MHz, $CD_3CN$): δ 8.08 (br.s, 2H), 8.05 – 8.00 (m, 2H), 7.95 (dd, *J* = 7.8, 1.5 Hz, 1H), 7.54 (d, *J* = 2.6 Hz, 2H), 7.22 – 7.12 (m, 3H), 7.03 (dd, *J* = 8.8, 2.6 Hz, 2H), 3.57 – 3.48 (m, 6H), 3.50 – 3.39 (m, 4H), 3.29 (t, *J* = 6.5 Hz, 2H), 1.71 – 1.56 (m, 2H), 1.42 – 1.33 (m, 2H), 1.33 – 1.25 (m, 2H), 1.25 – 1.14 (m, 2H).
$^{13}$C NMR (101 MHz, $CD_3CN$): δ 169.8, 166.6, 159.4, 153.8, 142.9, 138.4, 130.04, 130.00, 127.9, 127.3, 126.8, 123.3, 121.9, 110.6, 83.2, 71.5, 70.9, 70.7, 69.8, 46.1, 40.6, 33.2, 30.1, 27.2, 26.0.
HRMS (ESI-QTOF) *m/z*: $[M + H]^+$ Calcd for $C_{31}H_{33}ClNO_9S$ 630.1559; Found 630.1559.

**S12**

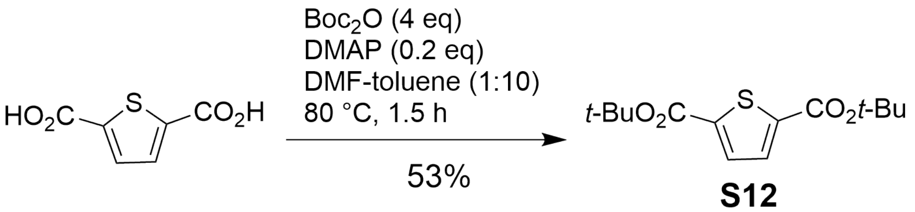


**S12**

2,5-Thiophenedicarboxylic acid (1.00 g, 6 mmol), 4-dimethylaminopyridine (DMAP; 142 mg, 0.1 mmol, ~0.2 equiv.) and di-*tert*-butyl dicarbonate (5.07 g, 23 mmol, 4 equiv) were mixed with toluene (100 mL) and DMF (10 mL) in a 250 mL round-bottom flask equipped with a bubbler and a reflux condenser. The reaction mixture was stirred at 80 °C for 1.5 h, after which no more bubbling was observed. The solvents were removed on a rotary evaporator, the residue was dissolved in EtOAc (20 mL) and washed with sat. aq. $NaHCO_3$ (3×25 mL). The organic layer was dried over $Na_2SO_4$, and the product was isolated by flash column chromatography (12 g Interchim SiHP 30 µm cartridge, gradient 0% to 10 % EtOAc/hexane) to give 870 mg (53%) of **S12** as colorless oil (analytical data match those of the known compound [20]).

$^1$H NMR (400 MHz, $CDCl_3$): δ 7.62 (s, 2H), 1.58 (s, 18H).
$^{13}$C NMR (101 MHz, $CDCl_3$): δ 161.1, 140.6, 132.4, 82.7, 28.3.

**6b**

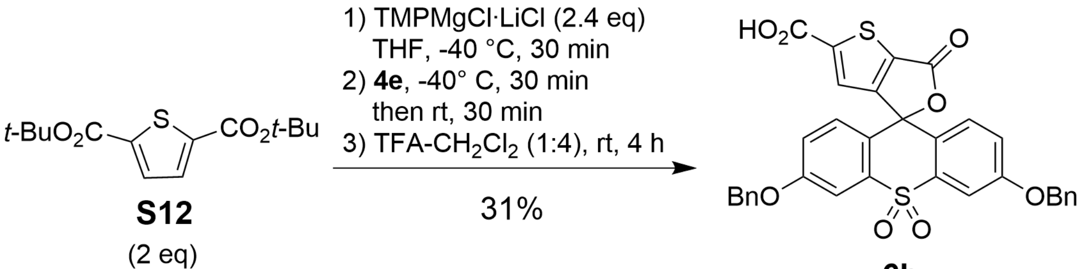


**S12**
(2 eq)

**6b**

In a flame-dried flask under argon, di-*tert*-butyl thiophene-2,5-dicarboxylate **S12** (122 mg, 0.4 mmol, 2 equiv.) was dissolved in dry THF (5 mL) and cooled to -40 °C (dry ice-acetonitrile bath). TMPMgCl·LiCl (0.52 mL of 1 M in THF-toluene, 0.52 mmol, 2.4 equiv.; cat. No. 703540, Sigma-Aldrich) was added dropwise, and the reaction mixture was stirred at -40 °C for 30 min. A suspension of **4e** (100 mg, 0.2 mmol) in THF (2 mL) was then added dropwise, the reaction mixture was stirred at -40 °C for 30 min and allowed to warm up to rt. It was then quenched by addition of water (5 mL) and acetic acid (1 mL), extracted with EtOAc (3×25 mL) and the combined extracts were dried over $Na_2SO_4$. The product was isolated by flash column chromatography (12 g Interchim SiHP 30 µm cartridge, gradient 0% to 30 % EtOAc/hexane) to give 82 mg of crude material, which was dissolved in TFA-$CH_2Cl_2$ mixture (1:4, 5 mL). The mixture was stirred at rt for 4 h until the conversion was complete (HPLC-MS). The mixture was

washed with water (3×10 mL), the organic layer was dried over $Na_2SO_4$, filtered and evaporated. The product was isolated by flash column chromatography (12 g Interchim SiHP 30 µm cartridge, gradient 0% to 10% methanol/$CH_2Cl_2$) to give 50 mg (31%) of **6b** as yellow solid.

$^1$H NMR (400 MHz, $CDCl_3$): δ 8.03 (br.s, 1H), 7.76 (d, *J* = 2.1 Hz, 2H), 7.52 (d, *J* = 8.9 Hz, 2H), 7.48 – 7.30 (m, 10H), 7.16 (dd, *J* = 8.9, 2.1 Hz, 2H), 5.14 (s, 4H).
$^{13}$C NMR (101 MHz, $CDCl_3$): δ 180.0, 165.2, 163.9, 159.9, 136.8, 135.5, 128.9, 128.7, 127.83, 127.77, 127.4, 121.0, 109.5, 81.9, 70.9.
HRMS (ESI-QTOF) *m/z*: $[M + H]^+$ Calcd for $C_{33}H_{23}O_8S_2$ 611.0829; Found 611.0828.

**6b-Halo**

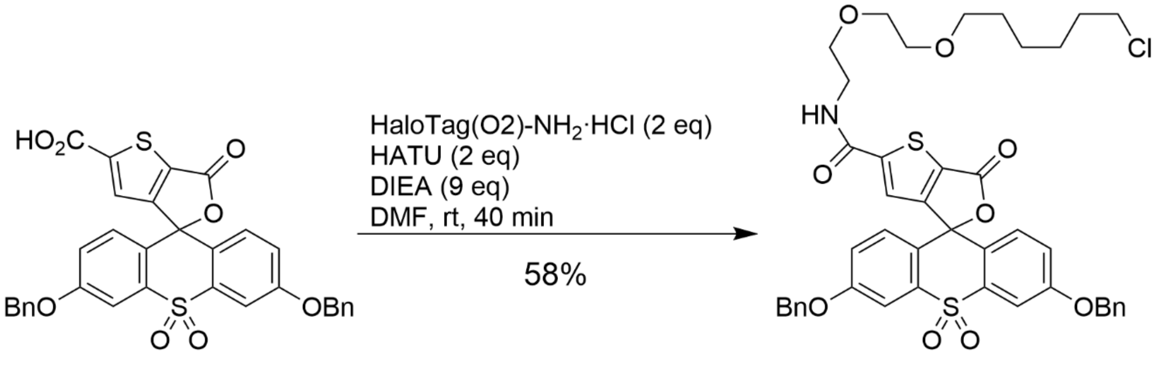


**6b** **6b-Halo**

Compound **6b** (40 mg, 65 µmol) was dissolved in dry DMF (2 mL). 2-(2-(6-Chlorohexyloxy)ethoxy)ethan-1-amine hydrochloride (HaloTag(O2) amine; 34 mg, 130 µmol, 2 equiv; known compound [19]), 1-[bis(dimethylamino)methylene]-1*H*-1,2,3-triazolo[4,5-*b*]pyridinium 3-oxid hexafluorophosphate (HATU; 50 mg, 131 µmol, 2 equiv.), *N*-ethyldiisopropylamine (DIEA; 100 µL, 573 µmol, 9 equiv.) were then added, and the reaction mixture was stirred at rt for 40 min. HPLC-MS analysis showed complete conversion of **6b**. The solvents were removed *in vacuo*, and the product was isolated by flash column chromatography (12 g Interchim SiHP 30 µm cartridge, gradient 20% to 80% EtOAc/hexane) to give 31 mg (58%) of **6a-Halo** as pink solid.

$^1$H NMR (400 MHz, $CDCl_3$) δ 7.75 (d, *J* = 2.7 Hz, 2H), 7.65 (s, 1H), 7.56 (d, *J* = 8.9 Hz, 2H), 7.48 – 7.31 (m, 10H), 7.19 (dd, *J* = 8.9, 2.7 Hz, 2H), 7.17 – 7.08 (m, 1H), 5.15 (s, 4H), 3.66 – 3.52 (m, 8H), 3.50 (t, *J* = 6.7 Hz, 2H), 3.40 (t, *J* = 6.6 Hz, 2H), 1.77 – 1.66 (m, 2H), 1.53 – 1.42 (m, 2H), 1.42 – 1.32 (m, 2H), 1.32 – 1.21 (m, 2H).
$^{13}$C NMR (101 MHz, $CDCl_3$) δ 165.7, 164.3, 160.5, 159.8, 155.7, 136.6, 135.5, 128.9, 128.6, 128.3, 128.2, 127.7, 127.6, 120.89, 120.85, 109.4, 81.4, 71.4, 70.9, 70.4, 70.2, 69.7, 45.2, 40.2, 32.6, 29.4, 26.7, 25.5.
HRMS (ESI-QTOF) *m/z*: $[M + H]^+$ Calcd for $C_{43}H_{43}ClNO_9S_2$ 816.2063; Found 816.2073.

**7b-Halo**

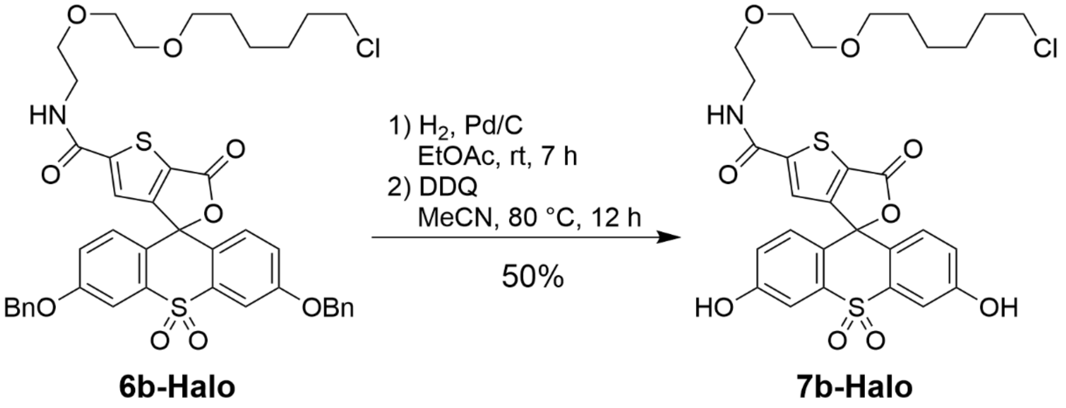

Compound **6b-Halo** (30 mg, 37 μmol) was dissolved in EtOAc (10 mL) in a 50 mL round-bottom flask. Palladium on carbon (40 mg; 10% Pd, cat. No. 205699, Sigma-Aldrich) was then added, the flask was evacuated and filled with hydrogen three times, and the mixture was vigorously stirred for 7 h. HPLC-MS analysis showed complete conversion of the starting **6b-Halo** into **7b-Halo** with overreduction into the corresponding triarylmethane. The supernatant was filtered through a PTFE syringe filter (0.22 μm) and evaporated. The crude product was dissolved in acetonitrile (10 mL), DDQ (140 mg, 0.62 mmol) was added and the reaction mixture was stirred in a closed vial at 80 °C for 12 h, monitoring the conversion with HPLC-MS. The solvent was then evaporated, the residue was dissolved in $CH_2Cl_2$ and washed with water, the organic layer was dried over $Na_2SO_4$ and evaporated. The product was isolated by preparative HPLC (ThermoFisher Hypersil GOLD 250×21.2 mm 5 μm, solvent flow rate 18 mL/min, gradient 10% to 80% A:B, A – acetonitrile + 0.1% (v/v) $HCO_2H$, B – water + 0.1% (v/v) $HCO_2H$) to give 11.7 mg (50%) of **7b-Halo** as pink solid.

$^1$H NMR (400 MHz, $CD_3CN$): δ 8.22 (br.s, 2H), 7.59 – 7.49 (m, 3H), 7.42 (t, *J* = 5.7 Hz, 1H), 7.37 (d, *J* = 8.7 Hz, 2H), 7.07 (dd, *J* = 8.7, 2.6 Hz, 2H), 3.59 – 3.49 (m, 6H), 3.49 – 3.43 (m, 2H), 3.44 – 3.38 (m, 2H), 3.31 (t, *J* = 6.5 Hz, 2H), 1.72 – 1.56 (m, 2H), 1.43 – 1.31 (m, 2H), 1.31 – 1.24 (m, 2H), 1.24 – 1.08 (m, 2H).

$^{13}$C NMR (101 MHz, $CD_3CN$): δ 165.6, 164.7, 161.3, 159.5, 156.8, 138.3, 130.3, 129.6, 127.4, 121.8, 121.7, 110.9, 82.6, 71.5, 70.9, 70.7, 69.7, 46.2, 40.6, 33.3, 30.1, 27.3, 26.1.

HRMS (ESI-QTOF) *m/z*: $[M + H]^+$ Calcd for $C_{29}H_{31}ClNO_9S_2$ 636.1123; Found 636.1131.

## 6c *tert*-butyl ester

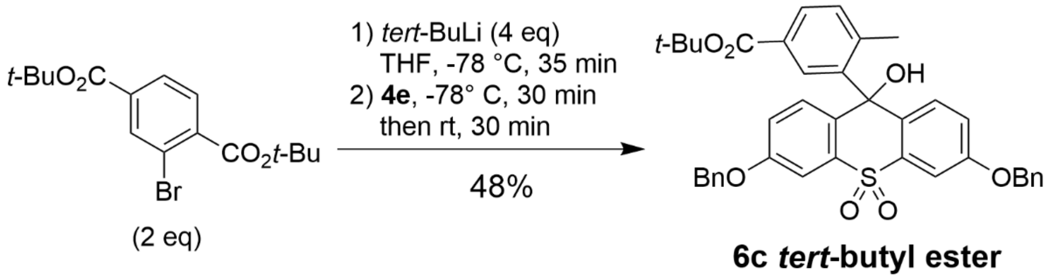


In a flame-dried flask under argon, *tert*-butyl 3-bromo-4-methylbenzoate (119 mg, 0.4 mmol, 2 equiv.) was dissolved in dry THF (5 mL) and cooled to -78 °C (dry ice-acetone bath). *tert*-Butyllithium (0.46 mL of 1.7 M in pentane, 0.78 mmol, 4 equiv.) was added dropwise and the mixture was stirred for 35 min. A suspension of **4e** (100 mg, 0.2 mmol) in THF (2 mL) was then added dropwise and the reaction mixture was stirred at -78 °C for 30 min. The mixture was allowed to warm up to rt, quenched by addition of water (5 mL) and acetic acid (1 mL), extracted with EtOAc (3×25 mL) and the combined extracts were dried over $Na_2SO_4$. The product was isolated by flash column chromatography (12 g Interchim SiHP 30 µm cartridge, gradient 0% to 20 % EtOAc/hexane) to give 68 mg (48%) of **6c *tert*-butyl ester** as yellow solid.

$^1$H NMR (400 MHz, $CDCl_3$): δ 8.89 (d, *J* = 1.9 Hz, 1H), 7.98 (dd, *J* = 7.8, 1.9 Hz, 1H), 7.73 (d, *J* = 2.7 Hz, 2H), 7.45 – 7.32 (m, 10H), 7.17 (d, *J* = 7.8 Hz, 1H), 6.99 (dd, *J* = 8.8, 2.7 Hz, 2H), 6.89 (d, *J* = 8.8 Hz, 2H), 5.14 (d, *J* = 1.6 Hz, 4H), 3.71 (br.s, 1H), 1.64 (s, 9H), 1.41 (s, 3H).
$^{13}$C NMR (101 MHz, $CDCl_3$): δ 166.1, 159.0, 141.4, 140.5, 137.6, 135.8, 134.7, 132.1, 130.8, 130.1, 129.7, 128.9, 128.6, 128.4, 127.8, 121.0, 108.3, 81.4, 72.2, 70.8, 28.4.
HRMS (ESI-QTOF) *m/z*: $[M – OH]^+$ Calcd for $C_{39}H_{35}O_6S$ 631.2149; Found 631.2150 – corresponds to the triarylmethyl cation:

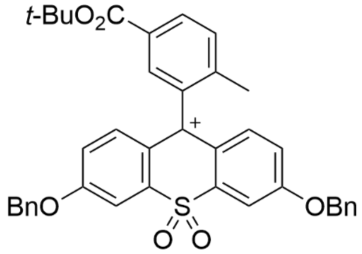


## 6c

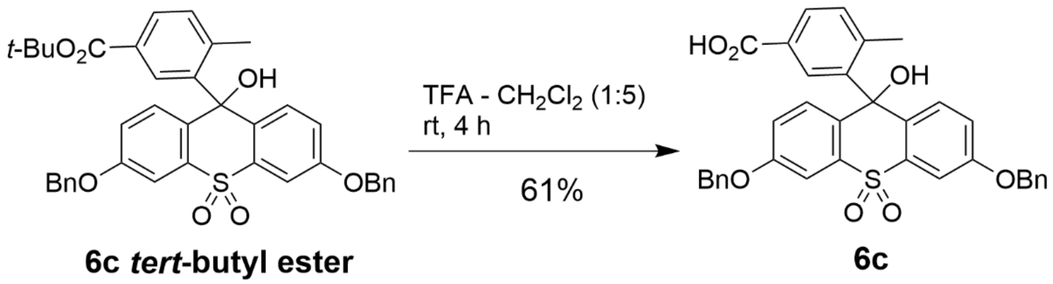

**6c *tert*-butyl ester** (68 mg, 105 µmol) was dissolved in TFA-$CH_2Cl_2$ mixture (1:5, 6 mL). The mixture was stirred at rt for 4 h until the conversion was complete (HPLC-MS). The mixture was washed with water (3×25 mL), the organic layer was dried over $Na_2SO_4$, filtered and evaporated. The product was isolated by flash column chromatography (12 g Interchim SiHP 30 µm cartridge, gradient 0% to 80 % EtOAc/hexane) to give 38 mg (61%) of **6c** as yellow solid.

$^1$H NMR (400 MHz, $CD_3CN$): δ 8.85 (s, 1H), 8.10 – 7.90 (m, 1H), 7.63 (d, *J* = 2.7 Hz, 2H), 7.50 – 7.30 (m, 11H), 7.19 (d, *J* = 7.9 Hz, 1H), 7.09 (dd, *J* = 8.9, 2.7 Hz, 2H), 6.94 (d, *J* = 8.9 Hz, 2H), 5.19 (s, 4H), 4.73 (s, 1H), 1.43 (s, 3H).
$^{13}$C NMR (101 MHz, $CD_3CN$): δ 160.0, 144.6, 138.5, 137.4, 134.1, 133.3, 132.2, 130.5, 129.6, 129.2, 128.9, 128.8, 121.7, 108.5, 72.5, 71.4, 21.0.
HRMS (ESI-QTOF) *m/z*: $[M - OH]^+$ Calcd for $C_{35}H_{27}O_6S$ 575.1723; Found 575.1520 – corresponds to the triarylmethyl cation:

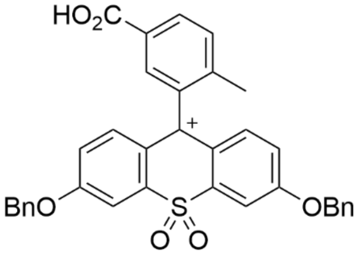


## 6c-Halo

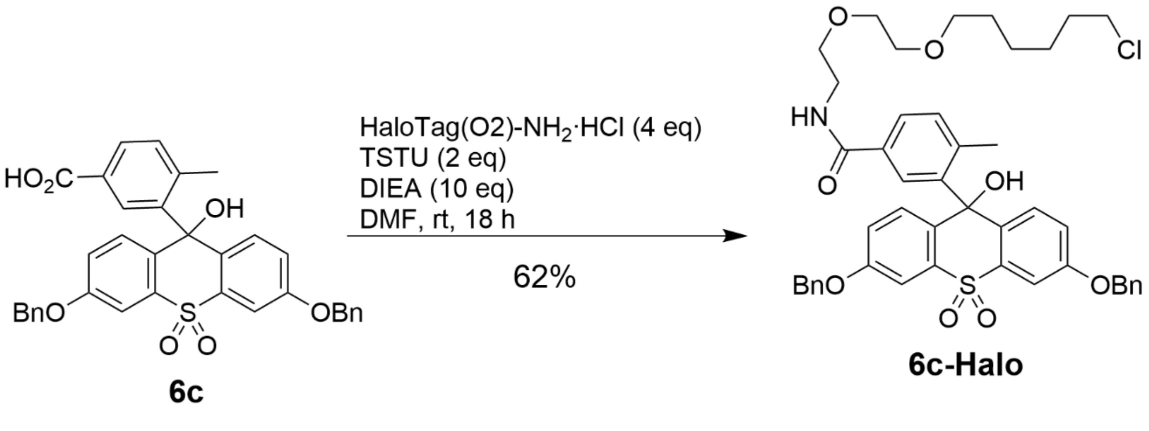


Compound **6c** (38 mg, 64 µmol) was dissolved in dry DMF (2 mL). *N,N,N′N′*-Tetramethyl-*O*-(*N*-succinimidyl)uronium tetrafluoroborate (TSTU; 38 mg, 128 µmol, 2 equiv.) and *N*-ethyldiisopropylamine (DIEA; 83 mg, 640 µmol, 10 equiv.) were then added followed by 2-(2-(6-chlorohexyloxy)ethoxy)ethan-1-amine hydrochloride (HaloTag(O2) amine; 33 mg, 128 µmol, 2 equiv; known compound [19]), and the reaction mixture was stirred at rt for 3 h. HPLC-MS analysis showed incomplete conversion, so additional HaloTag(O2) amine (33 mg, 128 µmol, 2 equiv.) were added and the stirring was continued overnight (18 h). The solvents were removed *in vacuo*, and the product was isolated by flash column chromatography (12 g Interchim SiHP

30 µm cartridge, gradient 20% to 100% EtOAc/hexane) to give 32 mg (62%) of **6c-Halo** as yellow solid.

$^{1}$H NMR (400 MHz, $CDCl_3$): δ 8.64 (d, *J* = 2.0 Hz, 1H), 7.88 (dd, *J* = 7.8, 2.0 Hz, 1H), 7.73 (d, *J* = 2.7 Hz, 2H), 7.47 – 7.30 (m, 10H), 7.20 (d, *J* = 7.9 Hz, 1H), 6.99 (dd, *J* = 8.8, 2.7 Hz, 2H), 6.97 – 6.93 (m, 1H), 6.88 (d, *J* = 8.8 Hz, 2H), 5.14 (s, 4H), 3.95 (br.s, 1H), 3.74 – 3.69 (m, 4H), 3.69 – 3.65 (m, 2H), 3.61 – 3.56 (m, 2H), 3.48 (t, *J* = 6.7 Hz, 2H), 3.44 (t, *J* = 6.7 Hz, 2H), 1.75 – 1.67 (m, 2H), 1.55 (p, *J* = 6.8 Hz, 2H), 1.41 (s, 3H), 1.40 – 1.34 (m, 2H), 1.34 – 1.26 (m, 2H).
$^{13}$C NMR (101 MHz, $CDCl_3$): δ 167.5, 159.0, 141.4, 139.4, 137.6, 135.7, 134.6, 132.5, 132.4, 130.8, 128.9, 128.6, 127.9, 127.8, 125.4, 120.9, 108.3, 72.3, 71.4, 70.8, 70.5, 70.2, 70.1, 45.2, 40.0, 32.6, 29.5, 26.8, 25.5, 21.3.^
HRMS (ESI-QTOF) *m/z*: $[M + Na]^+$ Calcd for $C_{45}H_{48}ClNO_8SNa$ 820.2681; Found 820.2684.

**7c-Halo**

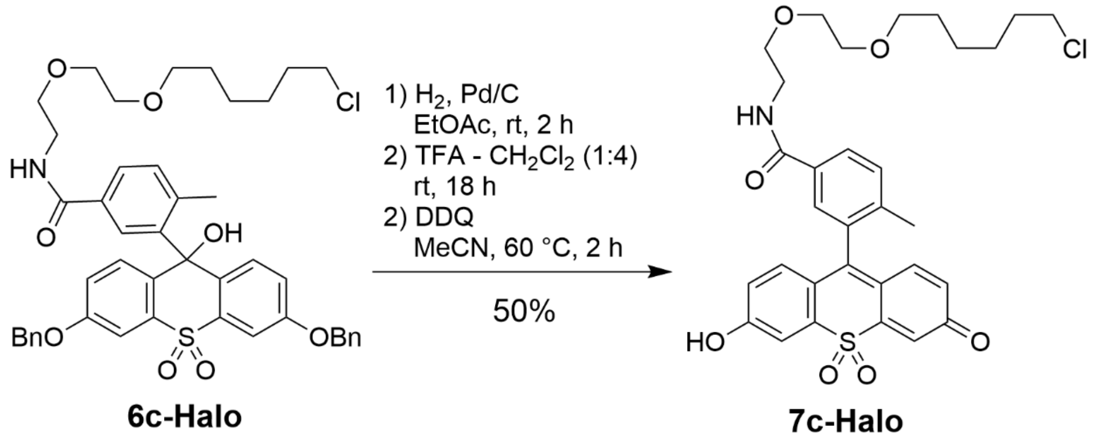


Compound **6c-Halo** (32 mg, 49 µmol) was dissolved in EtOAc (5 mL) in a 50 mL round-bottom flask. Palladium on carbon (10 mg; 10% Pd, cat. No. 205699, Sigma-Aldrich) was then added, the flask was evacuated and filled with hydrogen three times, and the mixture was vigorously stirred for 2 h. HPLC-MS analysis showed complete debenzylation of the starting **6b-Halo** with significant overreduction into the corresponding triarylmethane. The reaction mixture was filtered through Celite, evaporated and the residue was dissolved in TFA-$CH_2Cl_2$ mixture (1:4, 5 mL) and stirred overnight (18 h). The reaction mixture was then washed with water (3×25 mL), the organic layer was dried over $Na_2SO_4$, filtered and evaporated. The crude product was dissolved in acetonitrile (2 mL), DDQ (100 mg, 0.44 mmol) was added and the reaction mixture was stirred at 60 °C for 2 h. The solvent was evaporated and the product was isolated by preparative HPLC (ThermoFisher Hypersil GOLD 250×21.2 mm 5 µm, solvent flow rate 18 mL/min, gradient 10% to 80% A:B, A – acetonitrile + 0.1% (v/v) $HCO_2H$, B – water + 0.1% (v/v) $HCO_2H$) to give 12 mg (50%) of **7c-Halo** as red solid.

$^{1}$H NMR (400 MHz, $CDCl_3$:TFA-*d* 10:1 (v/v)): δ 7.88 (d, *J* = 8.1 Hz, 1H), 7.73 – 7.58 (m, 3H), 7.55 (d, *J* = 8.1 Hz, 1H), 7.02 (d, *J* = 9.3 Hz, 2H), 6.80 (d, *J* = 9.3 Hz, 2H), 3.99 – 3.68 (m, 8H), 3.63 (t, *J* = 7.0 Hz, 2H), 3.52 (t, *J* = 6.5 Hz, 2H), 2.18 (s, 3H), 1.87 – 1.66 (m, 2H), 1.62 (p, *J* = 7.1 Hz, 2H), 1.55 – 1.38 (m, 2H), 1.38 – 1.28 (m, 2H).
$^{13}$C NMR (101 MHz, $CDCl_3$:TFA-*d* 10:1 (v/v)): δ 168.6, 150.0, 137.8, 136.5, 134.0, 128.9, 126.3, 125.2, 124.3, 124.2, 123.5, 122.9, 119.0, 117.6, 114.3, 66.5, 64.2, 63.8, 39.5, 35.1, 34.9, 26.9, 24.4, 23.1, 21.0, 19.5, 14.2.
HRMS (ESI-QTOF) *m/z*: $[M + H]^+$ Calcd for $C_{31}H_{35}ClNO_7S$ 600.1817; Found 600.1824.

## Supplementary references


[1] Britton, H. T. S.; Robinson, R. A. Universal buffer solutions and the dissociation constant of veronal. *J. Chem. Soc.* **1931**, 1456-1462; https://doi.org/10.1039/JR9310001456.

[2] Butkevich, A. N.; Ta, H.; Ratz, M.; Stoldt, S.; Jakobs, S.; Belov, V. N.; Hell, S. W. Two-color 810 nm STED nanoscopy of living cells with endogenous SNAP-tagged fusion proteins. *ACS Chem. Biol.* **2018**, *13(2)*, 475-480; https://doi.org/10.1021/acschembio.7b00616.

[3] Lu, S.; Ong, J.-Y.; Yang, H.; Poh, S. B.; Liew, X.; Seow, C. S. D.; Wong, M. W.; Zhao, Y. Diastereo- and atroposelective synthesis of bridged biaryls bearing an eight-membered lactone through an organocatalytic cascade. *J. Am. Chem. Soc.* **2019**, *141*, 17062-17067; https://doi.org/10.1021/jacs.9b08510.

[4] Wang, D.; Cui, S. Rh(III)-catalyzed aldehyde C–H bond functionalization of salicylaldehydes with arylboronic acids. *Tetrahedron* **2015**, *71*, 8511; https://doi.org/10.1016/j.tet.2015.09.053.

[5] Butkevich, A. N.; Bossi, M. L.; Lukinavičius, G.; Hell, S. W. Triarylmethane fluorophores resistant to oxidative photobluing. *J. Am. Chem. Soc.* **2019**, *141*, 981-989; https://doi.org/10.1021/jacs.8b11036.

[6] Rao, M. L. N.; Ramakrishna, B. S. Rh-catalyzed direct synthesis of 2,2′-dihydroxybenzophenones and xanthones. *RSC Adv.* **2016**, *6*, 75505-75511; https://doi.org/10.1039/C6RA18647E.

[7] Zheng, Y.; Clarkson, G. J.; Wills, M. Asymmetric transfer hydrogenation of *o*-hydroxyphenyl ketones: utilizing directing effects that optimize the asymmetric synthesis of challenging alcohols. *Org. Lett.* **2020**, *22*, 3717-3721; https://doi.org/10.1021/acs.orglett.0c01213.

[8] Wang, D.; Ma, Z.; Xi, J.; Wang, N.; Wang, T.; Liang, Y.; Zhang, Z. Synthesis of V-shaped bis-coumarins through aldol reaction/double lactonization cascade reaction from bis(2-hydroxyphenyl)methanone and Meldrum's acid. *Eur. J. Org. Chem.* **2022**, *13*, e202101489; https://doi.org/10.1002/ejoc.202101489.

[9] Heller, J.; Mura, J.-L. Manufacture of α-hydroxybenzophenone derivatives, their aqueous dispersions and their use for treating textiles. European patent application **1998**, EP 0 820 978 A1.

[10] Jing, Q.; Sandoval, C. A.; Wang, Z.; Ding, K. Complete chiral induction from enantiopure 1,2-diamines to benzophenone-based achiral bisphosphane ligands in Noyori-type $Ru^{II}$ catalysts. *Eur. J. Org. Chem.* **2006**, *16*, 3606-3616; https://doi.org/10.1002/ejoc.200600299.

[11] Liu, J.; Sun, Y.-Q.; Zhang, H.; Shi, H.; Shi, Y.; Guo, W. Sulfone-Rhodamines: A New Class of Near-Infrared Fluorescent Dyes for Bioimaging. *ACS Appl. Mater. Interfaces* **2016**, *8*, 22953-22962; https://doi.org/10.1021/acsami.6b08338.

[12] Zhang, W.-S.; Ji, D.-W.; Li, Y.; Zhang, X.-X.; Mei, Y.-K.; Chen, B.-Z.; Chen, Q.-A. Nickel-catalyzed divergent Mizoroki-Heck reaction of 1,3-dienes. *Nat. Commun.* **2023**, *14*, 651; https://doi.org/10.1038/s41467-023-36237-1.

[13] Zhao, P.; Yin, H.; Gao, H.; Xi, C. Cu-catalyzed synthesis of diaryl thioethers and S-cycles by reaction of aryl iodides with carbon disulfide in the presence of DBU. *J. Org. Chem.* **2013**, *78*, 10, 5001-5006; https://doi.org/10.1021/jo400709s.

[14] Huo, J.; Fu, Y.; Tang, M. J.; Liu, P.; Dong, G. Escape from palladium: nickel-catalyzed Catellani annulation. *J. Am. Chem. Soc.* **2023**, *145*, 11005-11011; https://doi.org/10.1021/jacs.3c03780.

[15] Mampuys, P.; Zhu, Y.; Sergeyev, S.; Puijter, E.; Orru, R. V. A.; Van Doorslaer, S.; Maes, B. U. W. Iodide-catalyzed synthesis of secondary thiocarbamates from isocyanides and thiosulfonates. *Org. Lett.* **2016**, *18*, 2808-2811; https://doi.org/10.1021/acs.orglett.6b01023.

[16] Grazia Cabiddu, M:, Cabiddu, S.; Cadoni, E.; Demontis, S.; Fattuoni, C.; Melis, S. A convenient synthesis of benzothiophene derivatives. *Tetrahedron* **2002**, *58*, 4529-4533; https://doi.org/10.1016/S0040-4020(02)00399-X.

[17] Kalkman, E. D.; Hartwig, J. F. Direct observation of diastereomeric α-C-bound enolates during enantioselective α-arylations: synthesis, characterization, and reactivity of arylpalladium fluorooxindole complexes. *J. Am. Chem. Soc.* **2021**, *143*, 11741-11750; https://doi.org/10.1021/jacs.1c05346.

[18] Habert, L.; Retailleau, P.; Gillaizeau, I. Rapid synthesis of 3-amino isocoumarin derivatives from ynamides. *Org. Biomol. Chem.* **2018**, *16*, 7351-7355; https://doi.org/10.1039/C8OB02305K.

[19] Liu, Y.; Wolstenholme, C. H.; Carter, G. C.; Liu, H.; Hu, H.; Grainger, L. S.; Miao, K.; Fares, M.; Hoelzel, C. A.; Yennawar, H. P.; Ning, G.; Du, M.; Bai, L.; Li, X.; Zhang, X. Modulation of fluorescent protein chromophores to detect protein aggregation with turn-on fluorescence. *J. Am. Chem. Soc.* **2018**, *140*, 7381-7384; https://doi.org/10.1021/jacs.8b02176.

[20] Goda, H.; Kimura, N.; Kimura, S.; Yoshikawa, N.; Teramoto, M.; Masuda, Y.; Matuzaki, Y. Preparation of thiophene-2,5-dicarboxylic acid diesters, tetrahydrothiophene-2,5-dicarboxylic acid diesters and dibenzoxazolylthiophenes. European patent application **1990**, EP 0 387 725 A2.

# NMR spectra

1H (400.15 MHz, CDCl3)

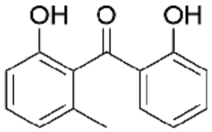


**S1**

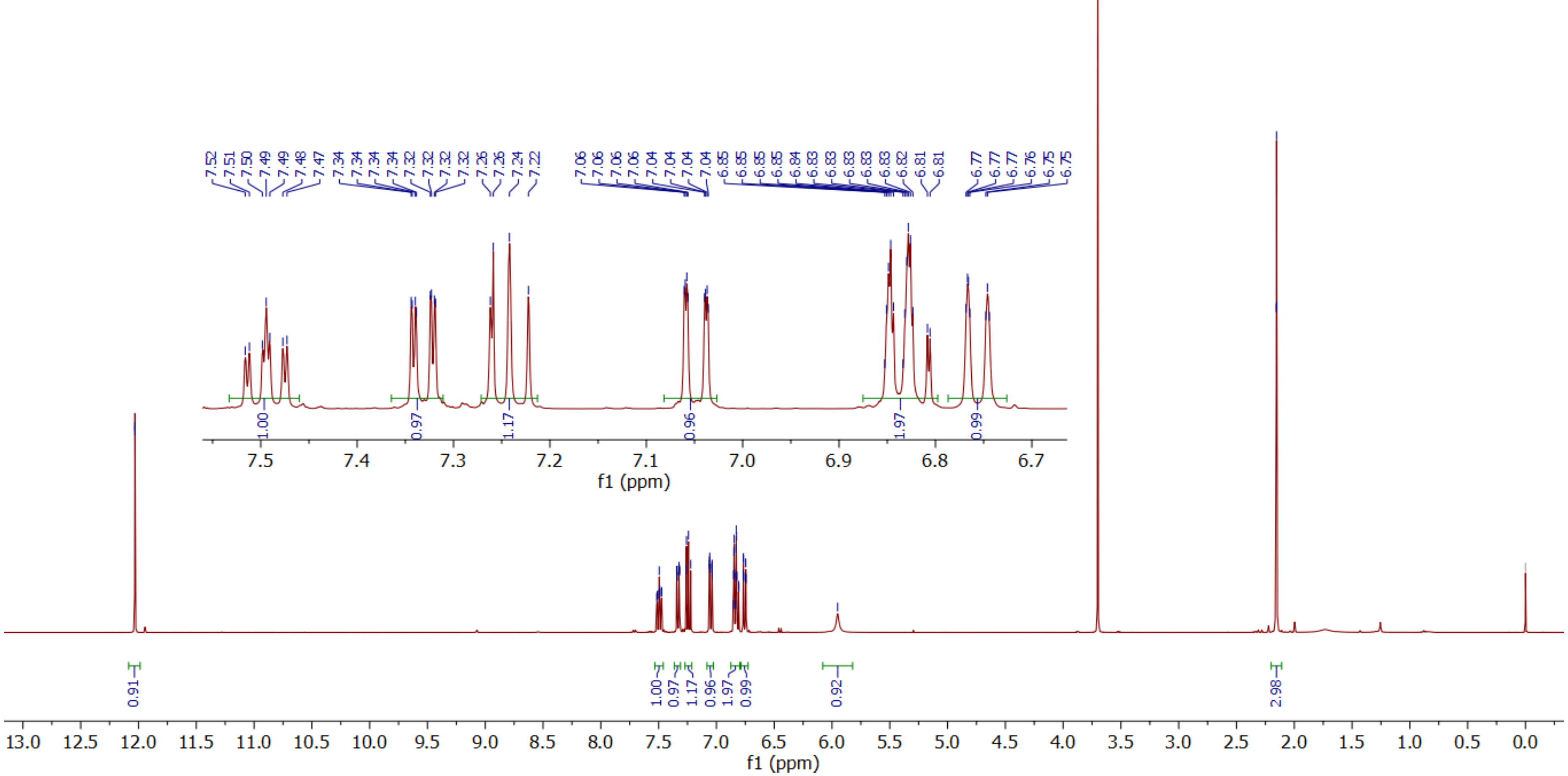

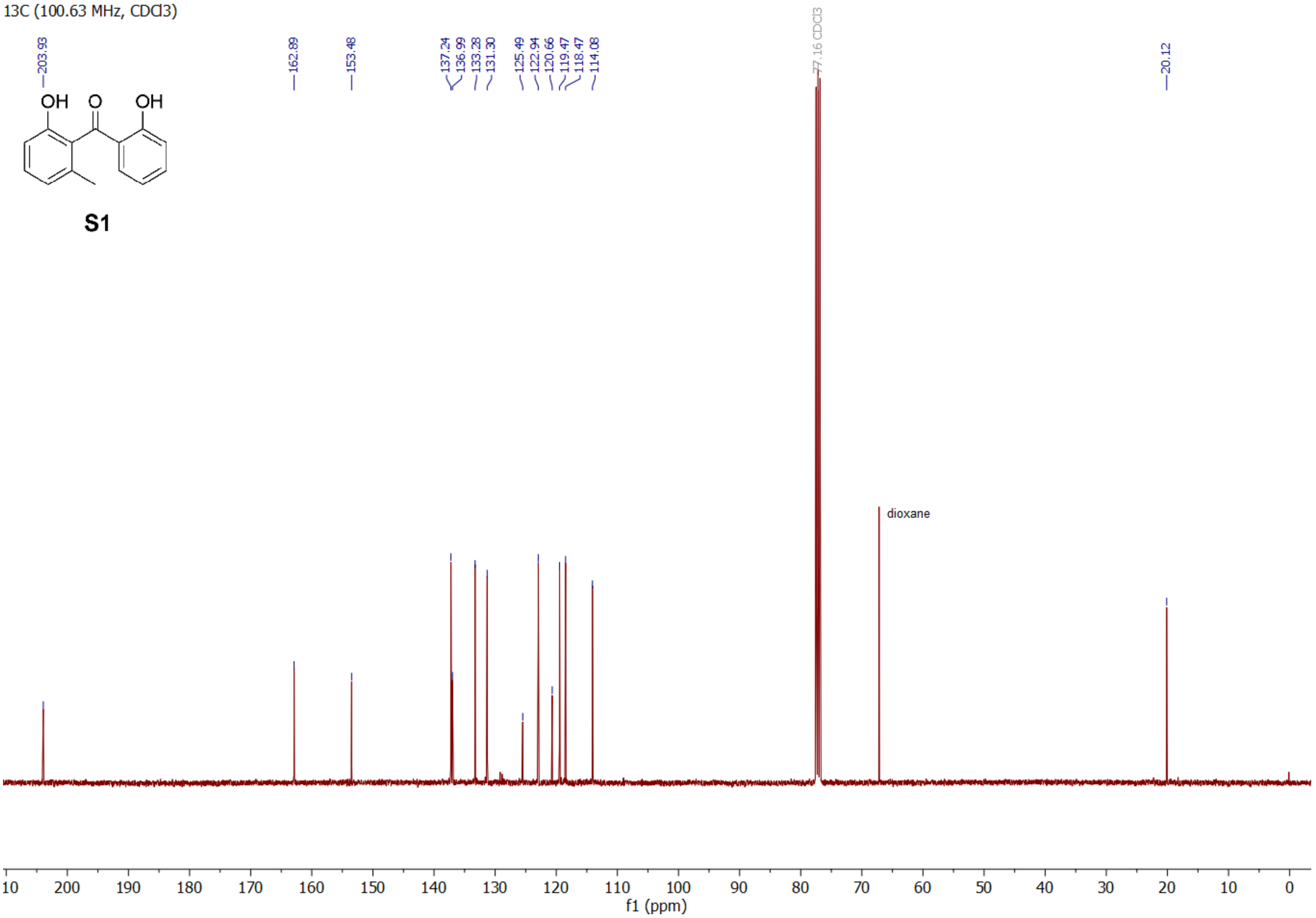
13C (100.63 MHz, CDCl3)
203.93
162.89
153.48
137.24
136.99
133.28
131.30
125.49
122.94
120.66
119.47
118.47
114.08
77.16 CDCl3
20.12
OH
O
OH
S1
dioxane
10 200 190 180 170 160 150 140 130 120 110 100 90 80 70 60 50 40 30 20 10 0
f1 (ppm)

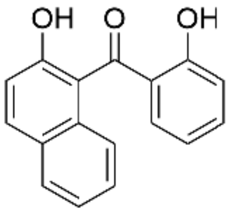

**S2**

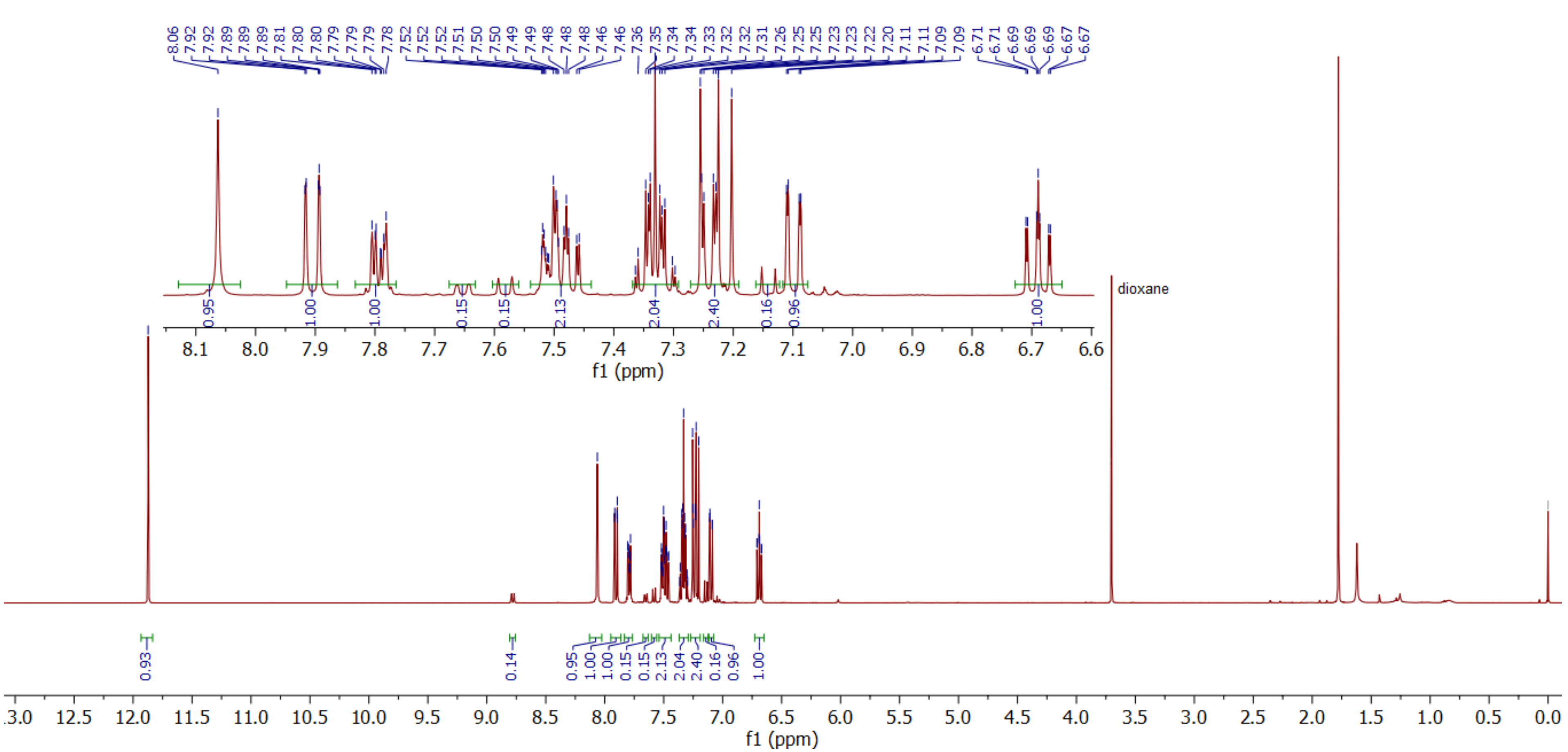

13C (100.63 MHz, CDCl3)

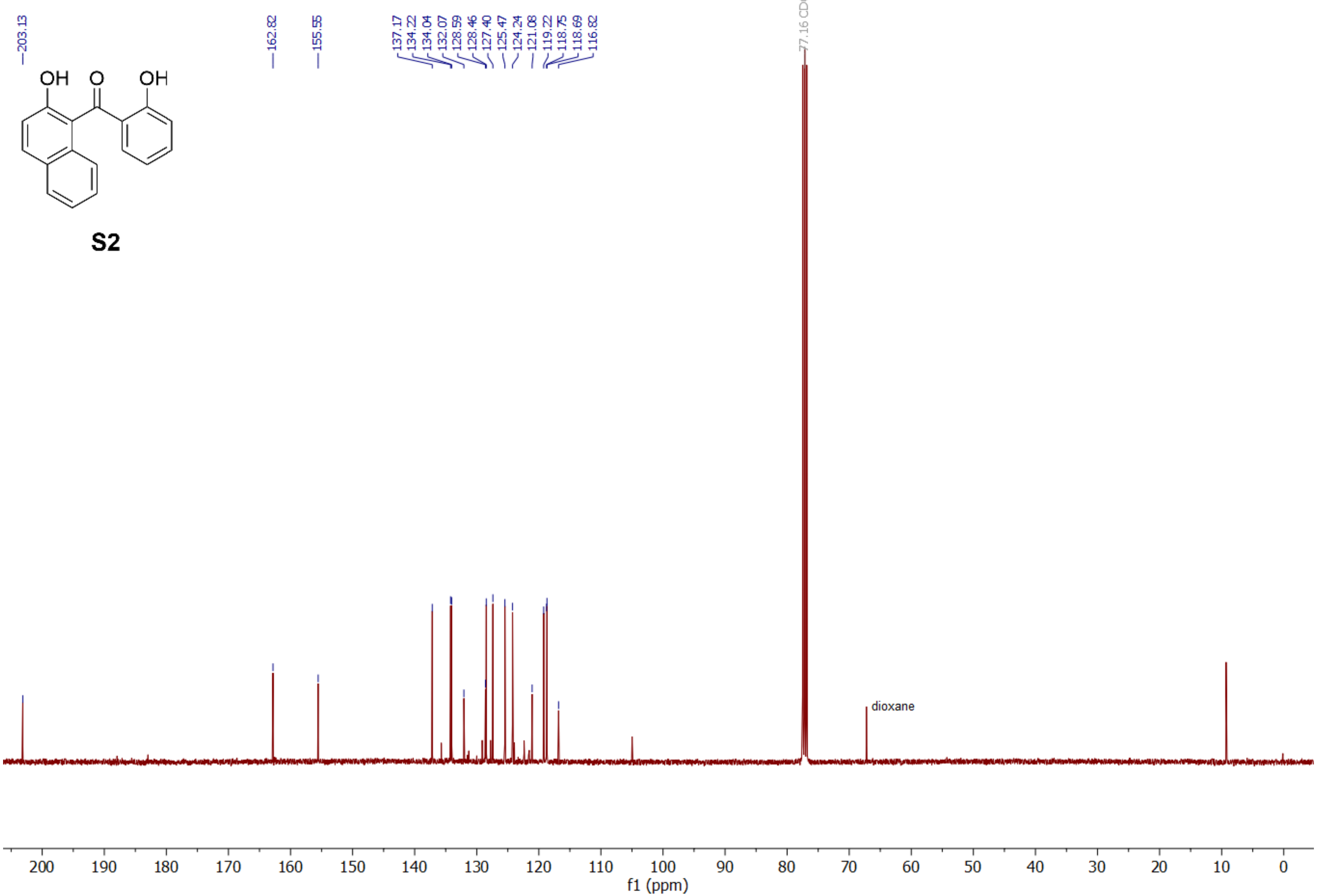


**S2**

1H (400.15 MHz, CDCl3)

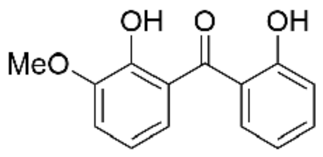


**S3**

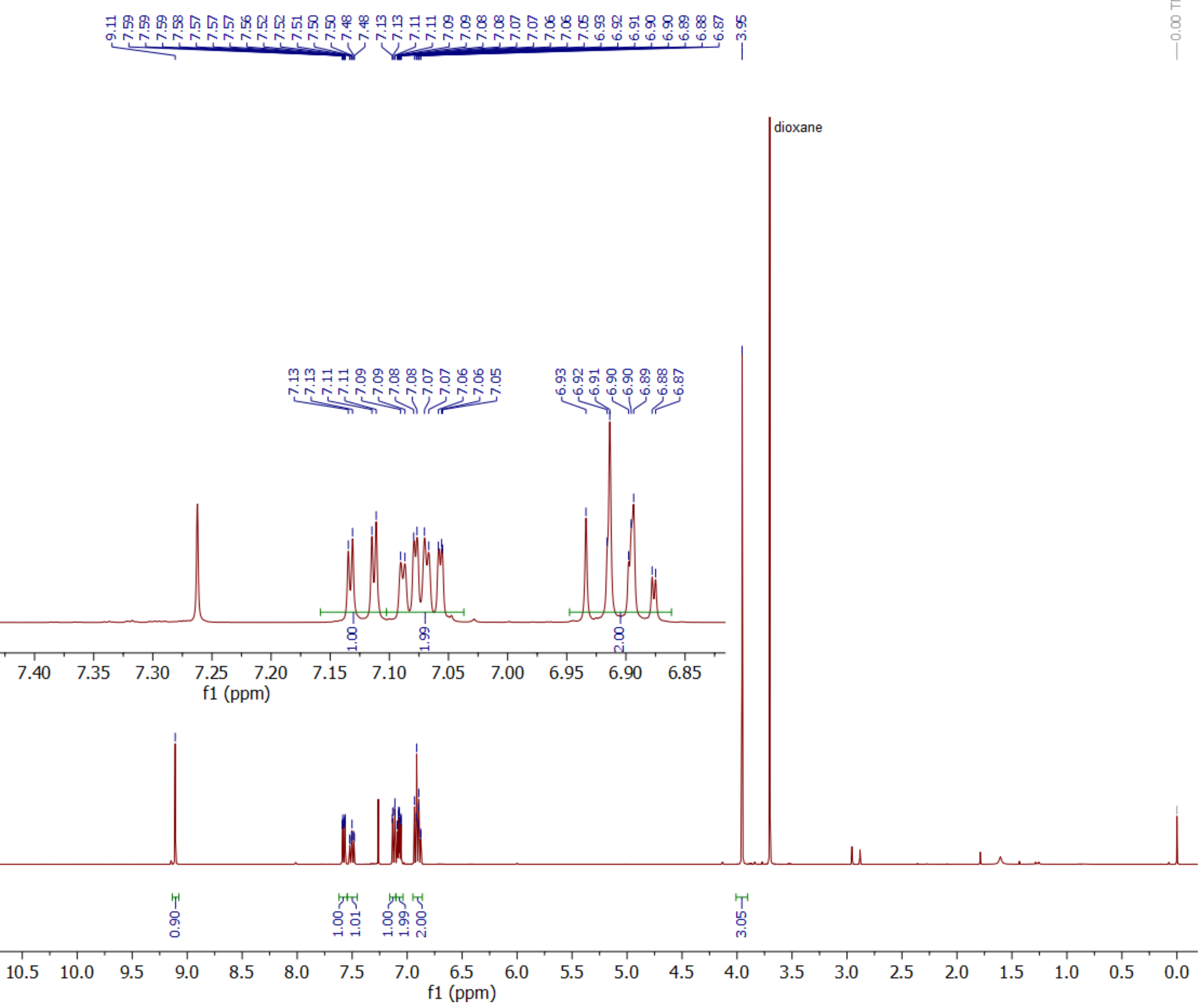

13C (100.63 MHz, CDCl3)

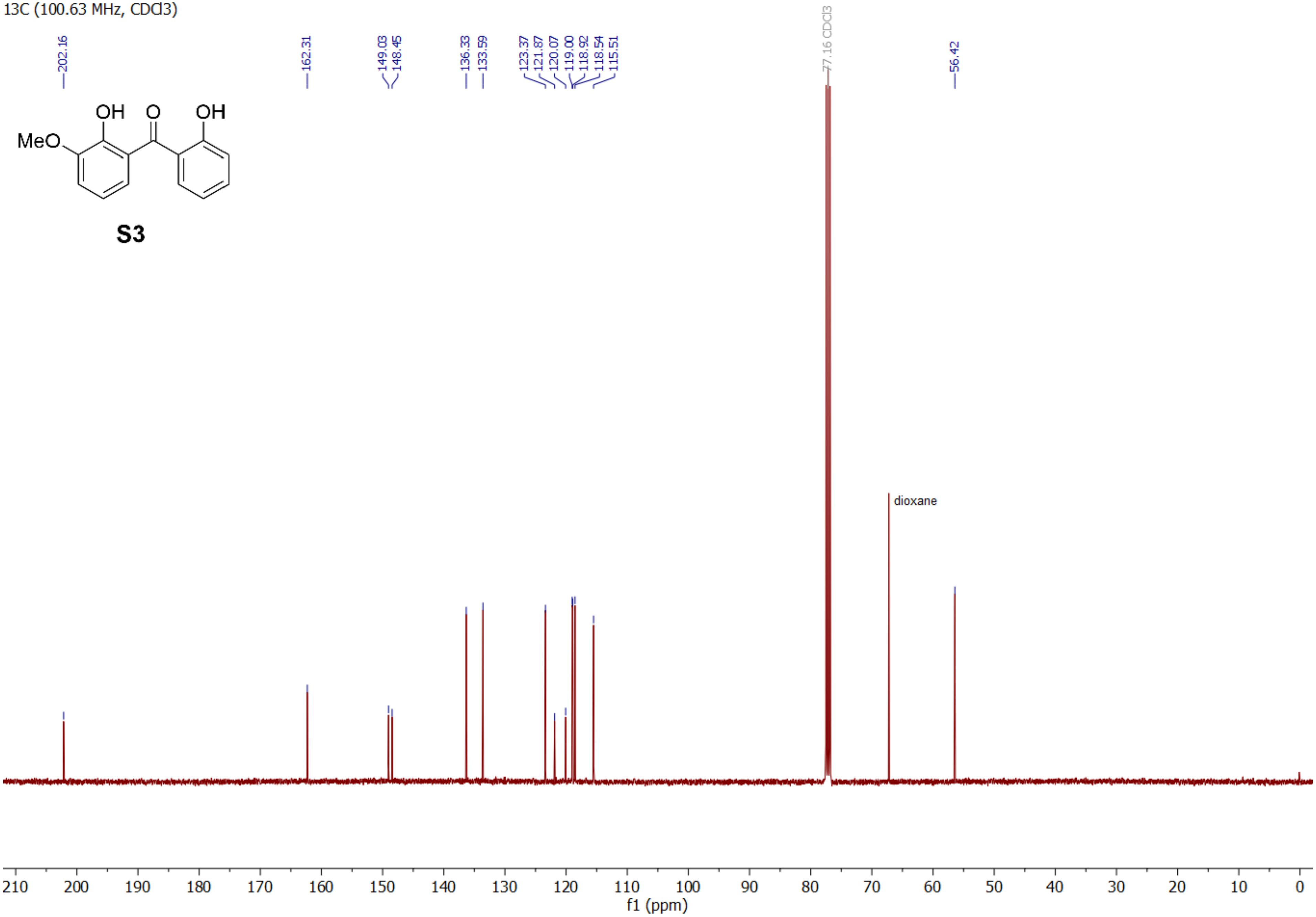

1H (400.15 MHz, CDCl3)

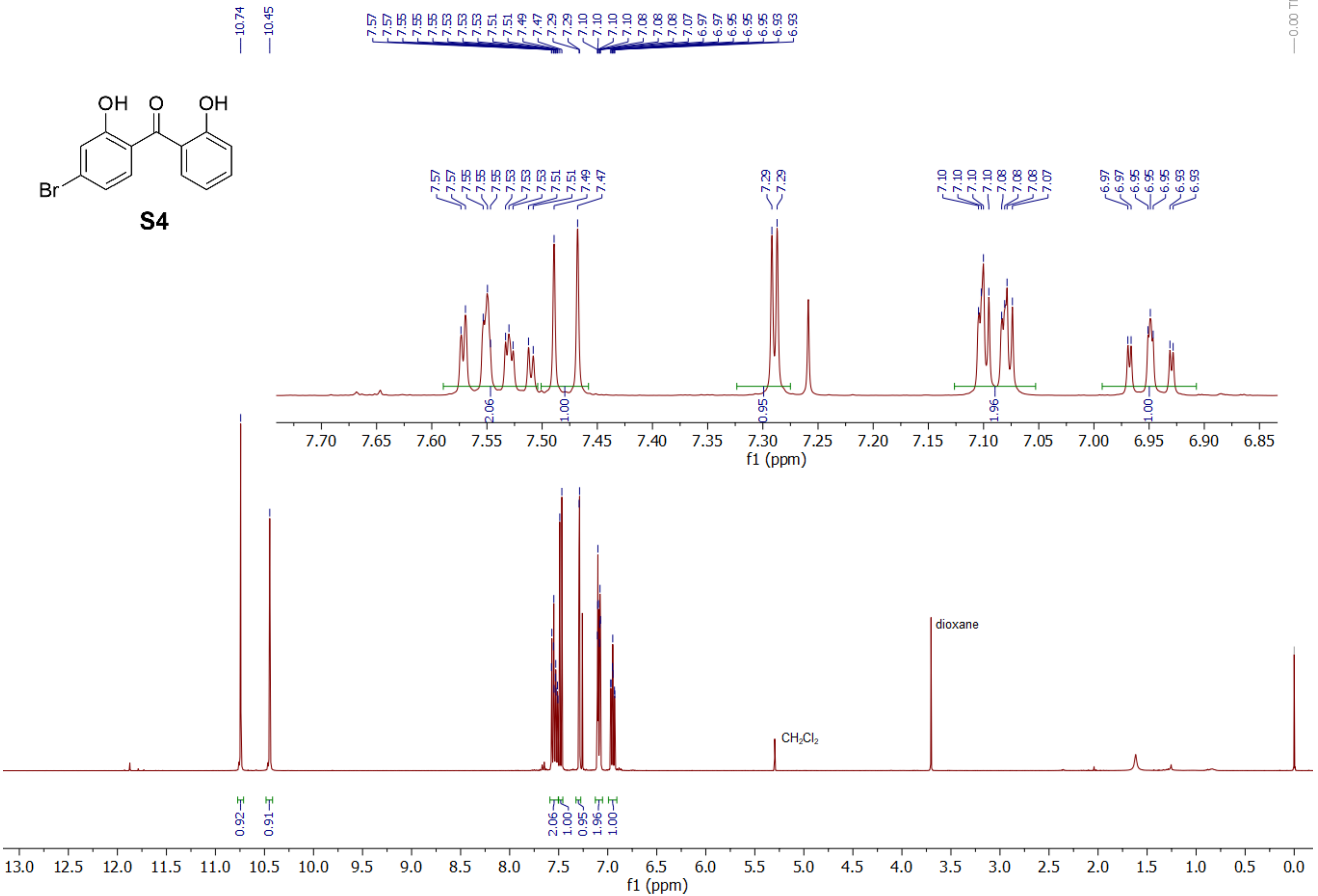

13C (100.63 MHz, CDCl3)

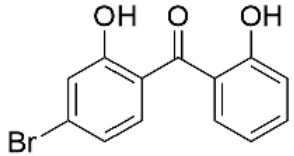


**S4**

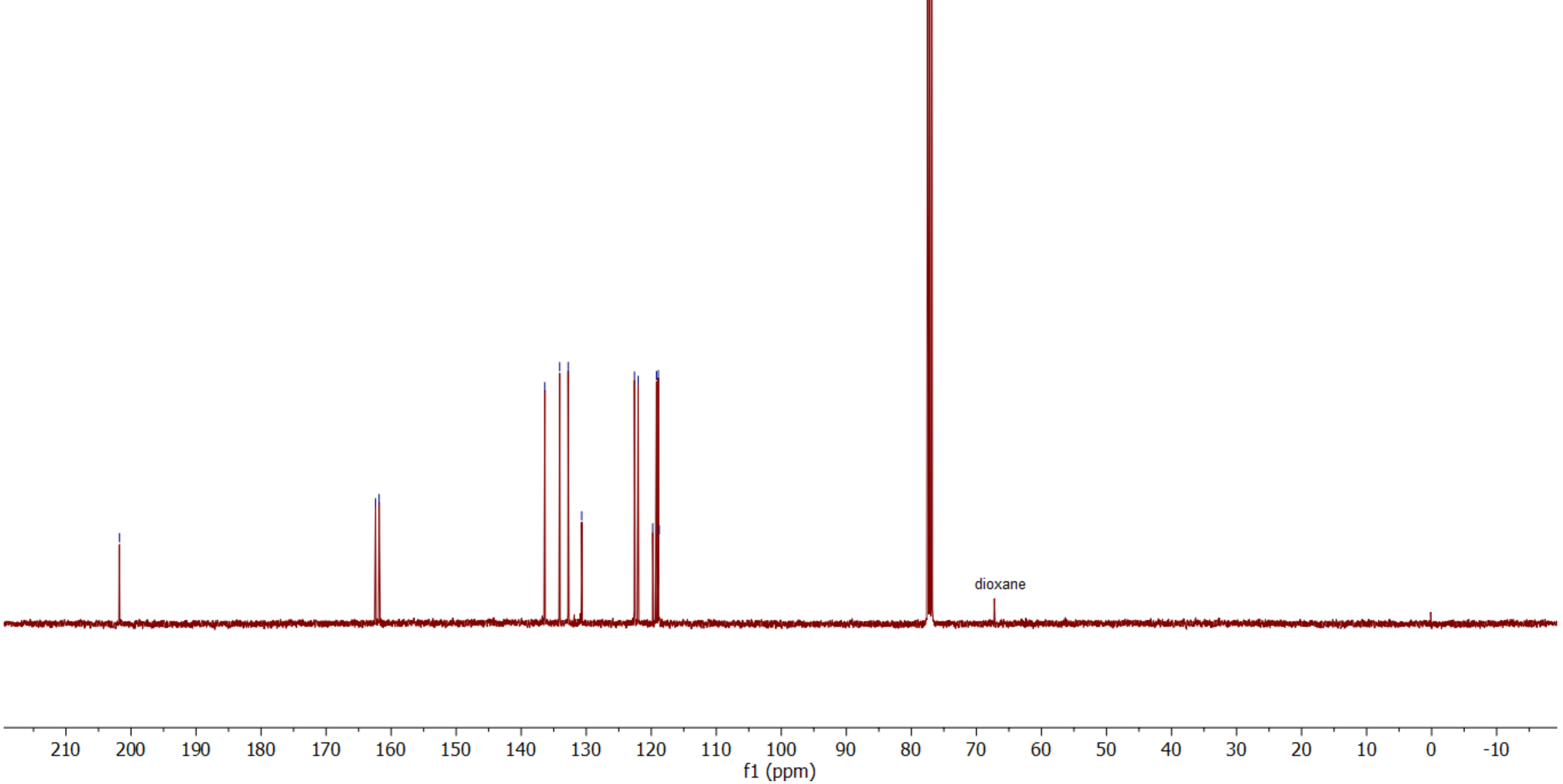

1H (400.15 MHz, CDCl3)

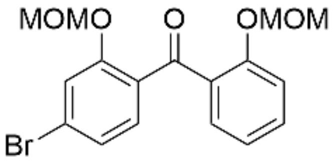


**S5**

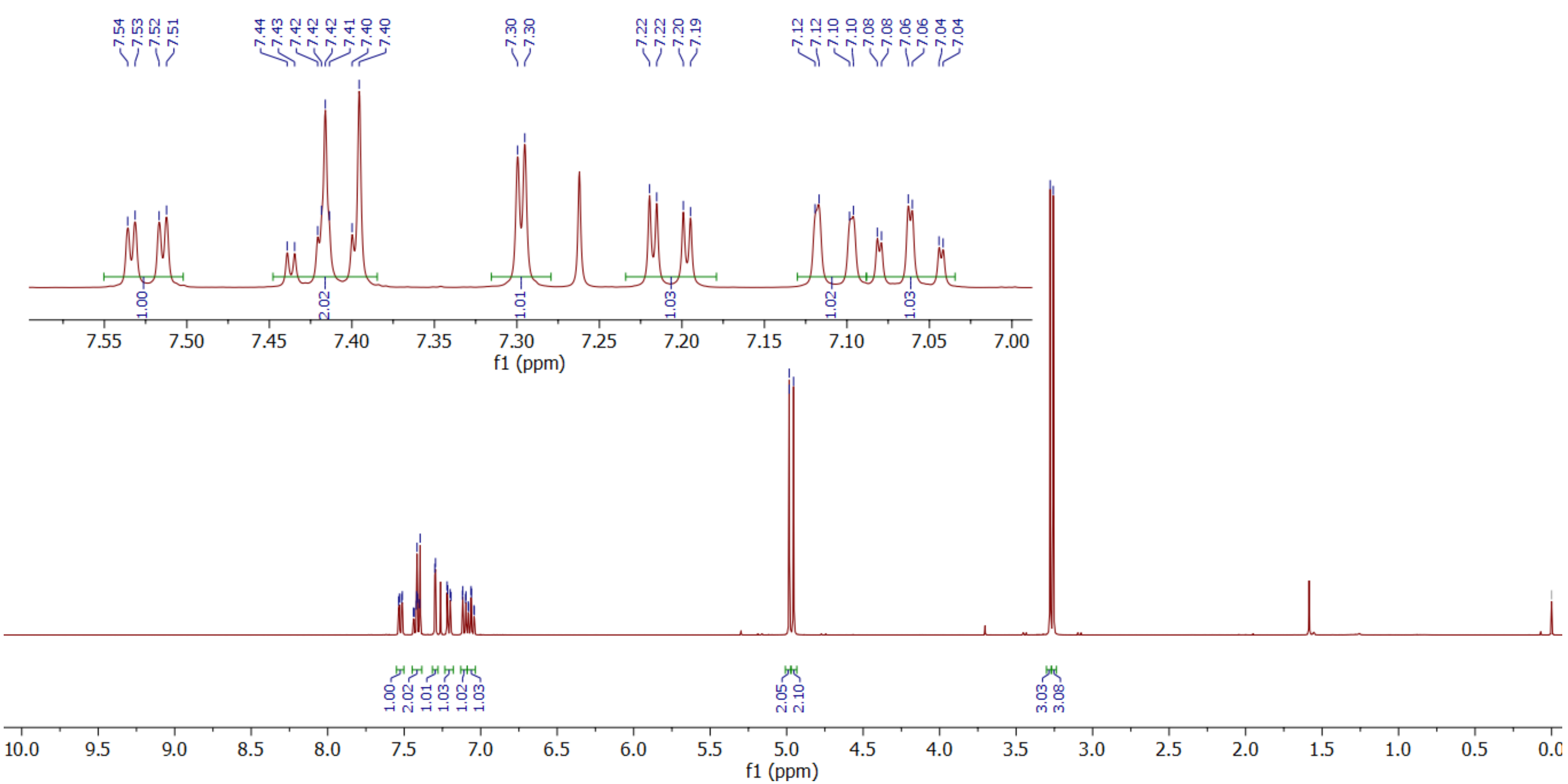

13C (100.63 MHz, CDCl3)

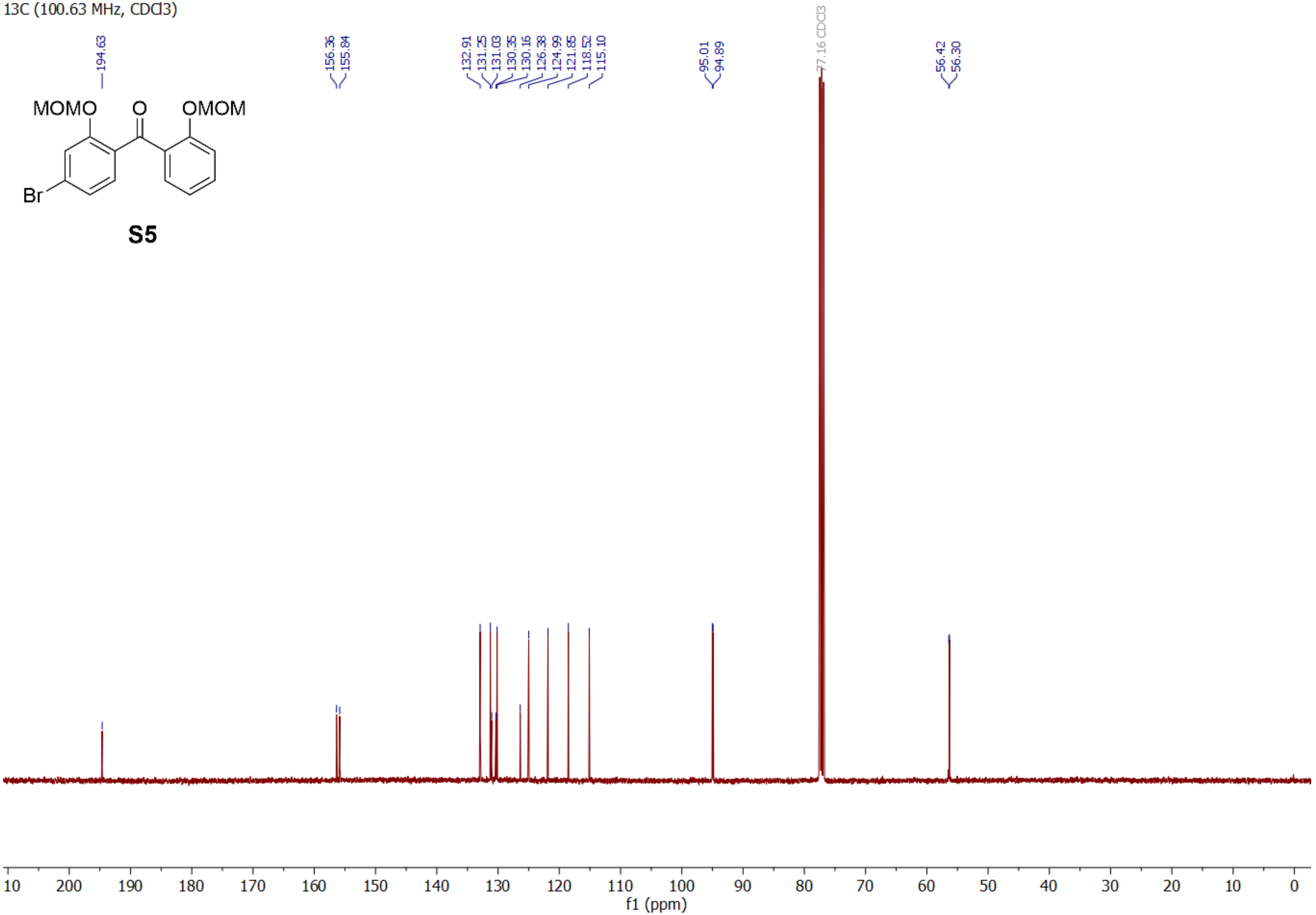

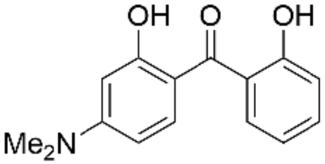
OH
O
OH
Me2N

**S6**

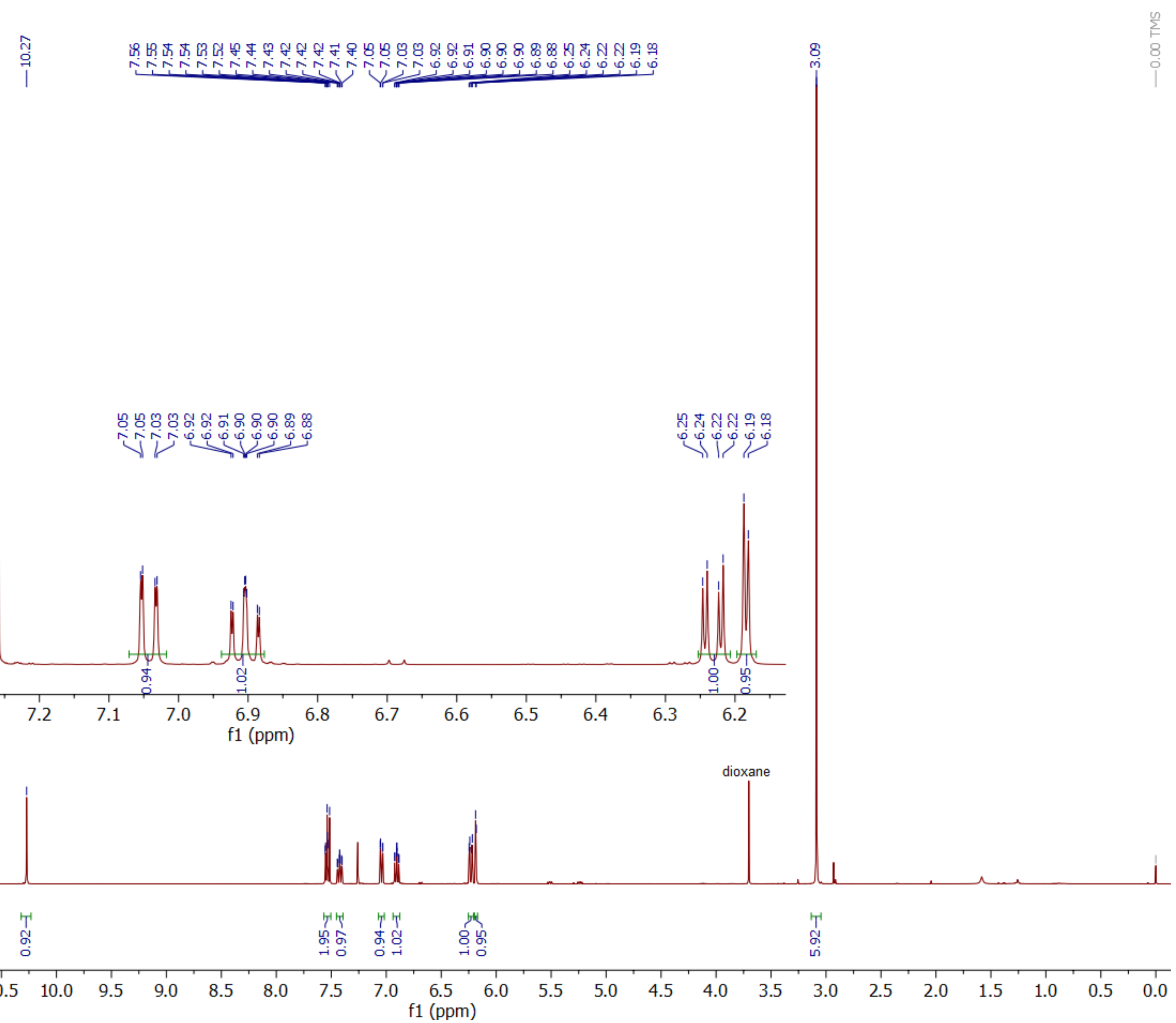
1H (400.15 MHz, CDCl3)
12.17
10.27
7.56 7.55 7.54 7.54 7.53 7.52 7.45 7.44 7.43 7.42 7.42 7.42 7.41 7.40 7.05 7.05 7.03 7.03 6.92 6.92 6.91 6.90 6.90 6.90 6.89 6.88 6.25 6.24 6.22 6.22 6.19 6.18
3.09
0.00 TMS
1.95 0.97 0.94 1.02 1.00 0.95
f1 (ppm)
dioxane
0.92 0.92 1.95 0.97 0.94 1.02 1.00 0.95 5.92
f1 (ppm)

13C (100.63 MHz, CDCl3)

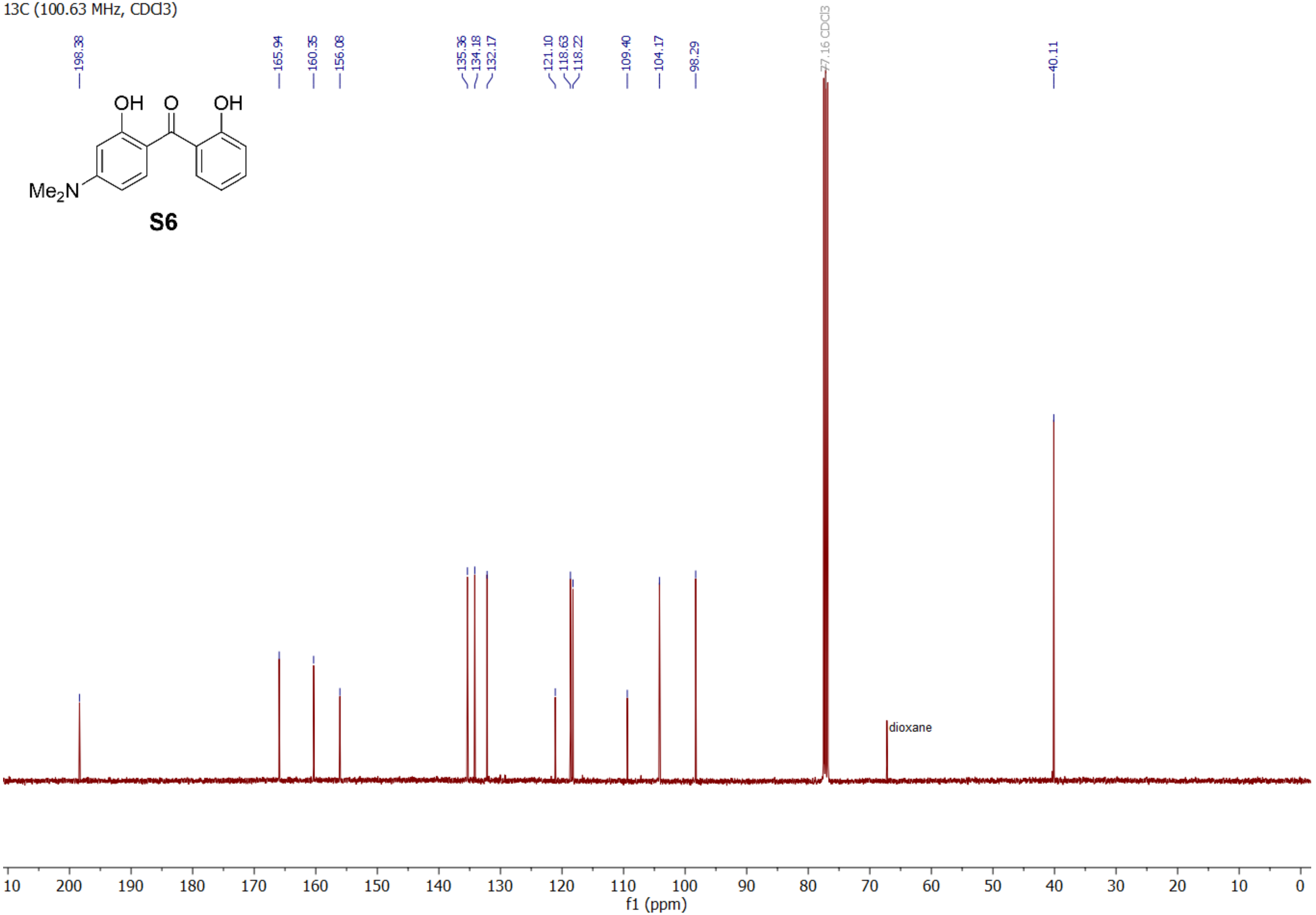

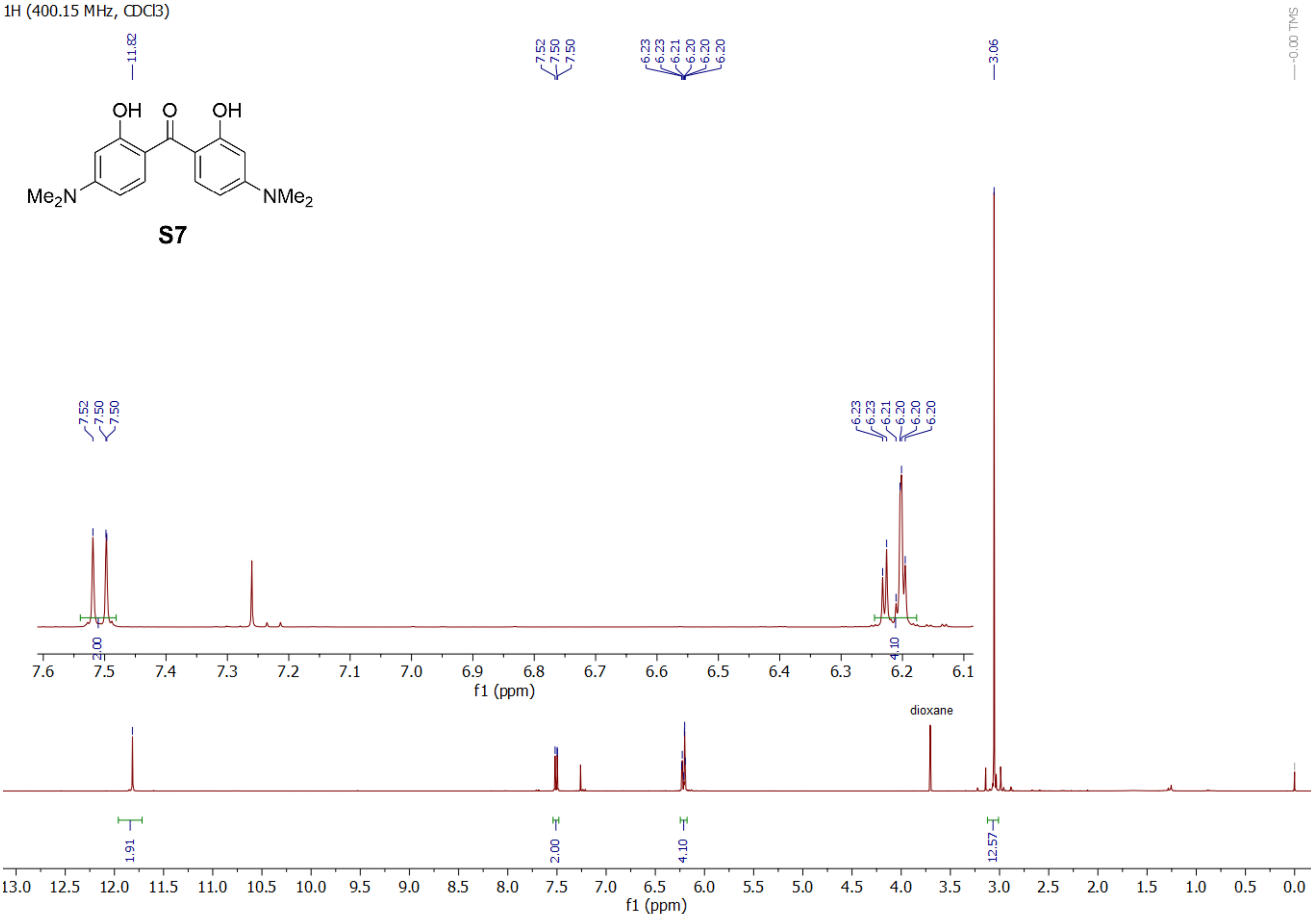
1H (400.15 MHz, CDCl3)
11.82
7.52
7.50
7.50
6.23
6.23
6.21
6.20
6.20
6.20
3.06
0.00 TMS
OH
O
OH
Me2N
NMe2
S7
2.00
4.10
f1 (ppm)
dioxane
1.91
2.00
4.10
12.57
f1 (ppm)

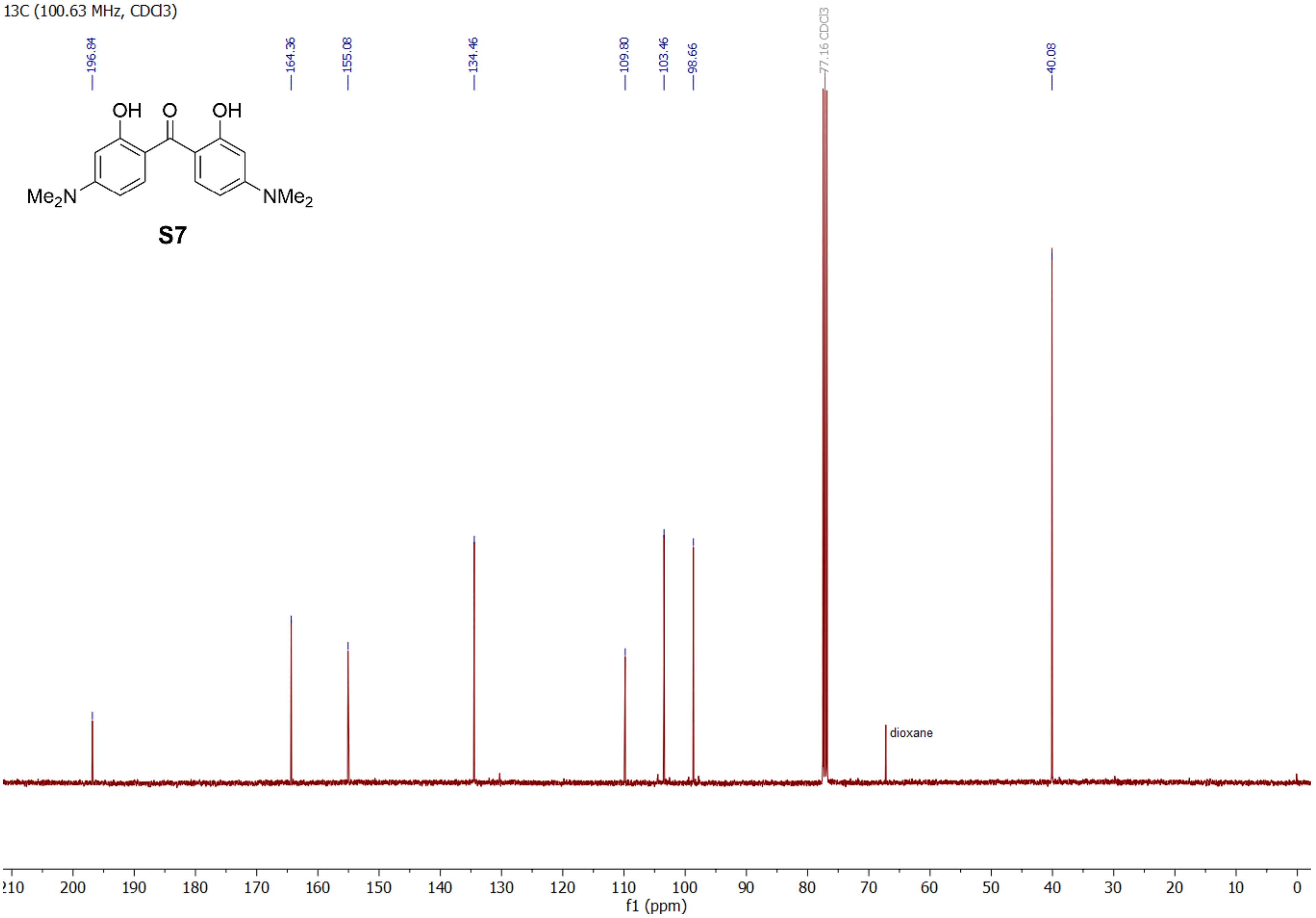
13C (100.63 MHz, CDCl3)
196.84
164.36
155.08
134.46
109.80
103.46
98.66
77.16 CDCl3
40.08
OH
O
OH
Me2N
NMe2
S7
dioxane
210
200
190
180
170
160
150
140
130
120
110
100
90
80
70
60
50
40
30
20
10
0
f1 (ppm)

1H (400.15 MHz, CDCl3)

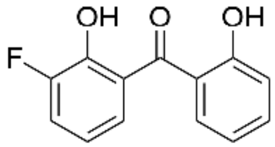


**S8**

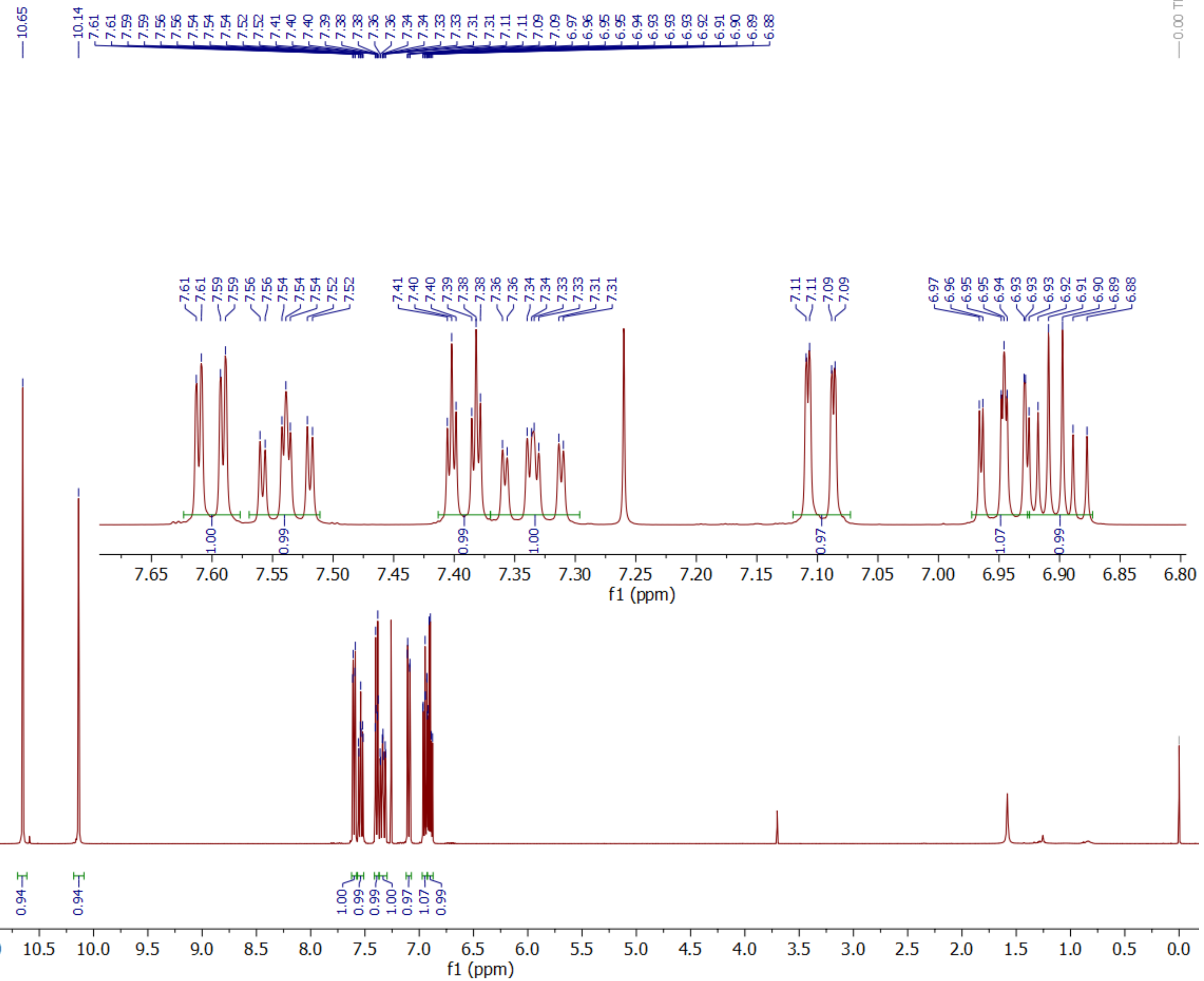

19F (376.48 MHz, CDCl3)

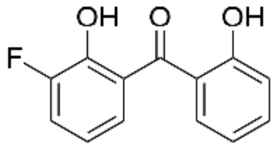

**S8**

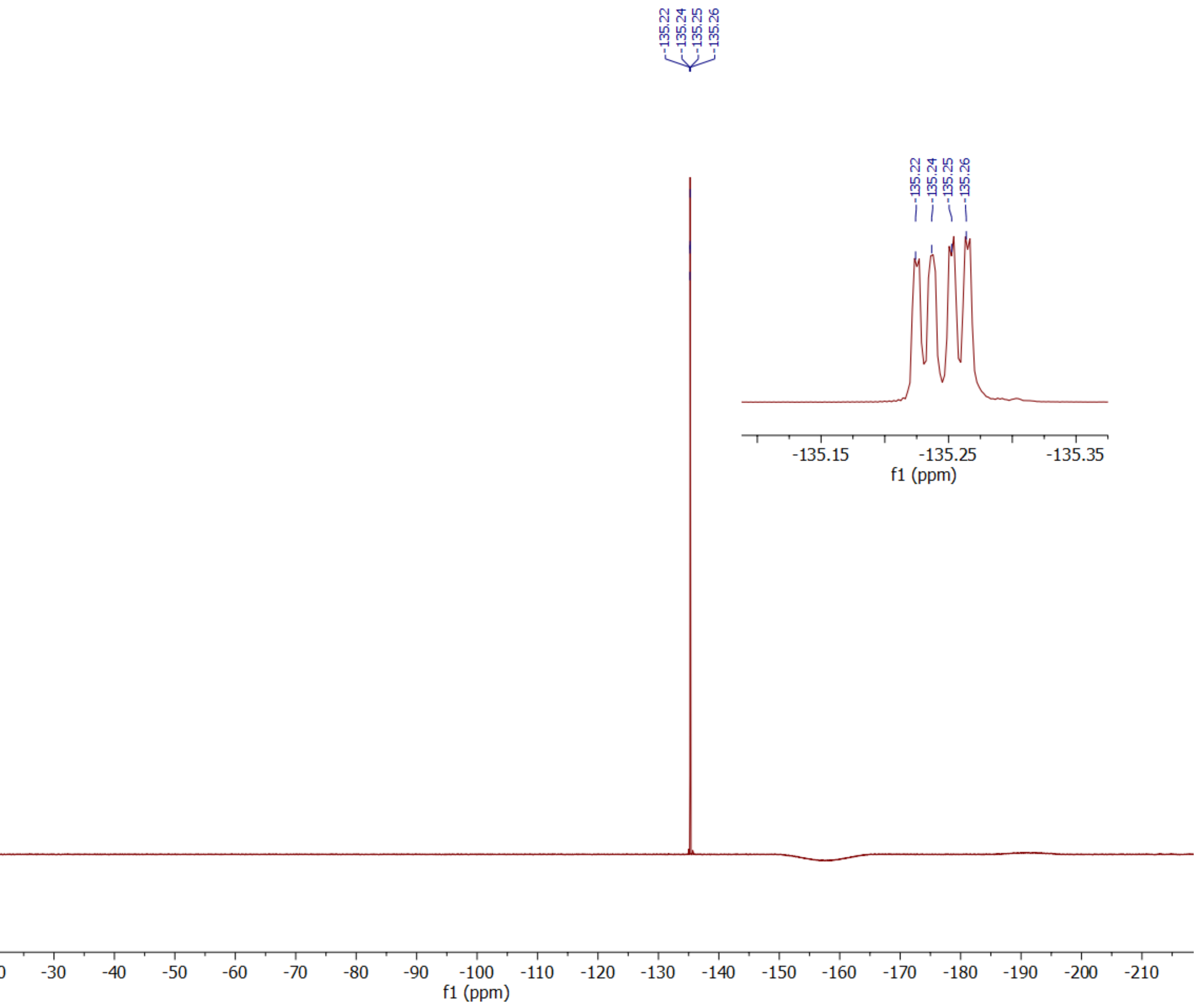

13C (100.63 MHz, CDCl3)

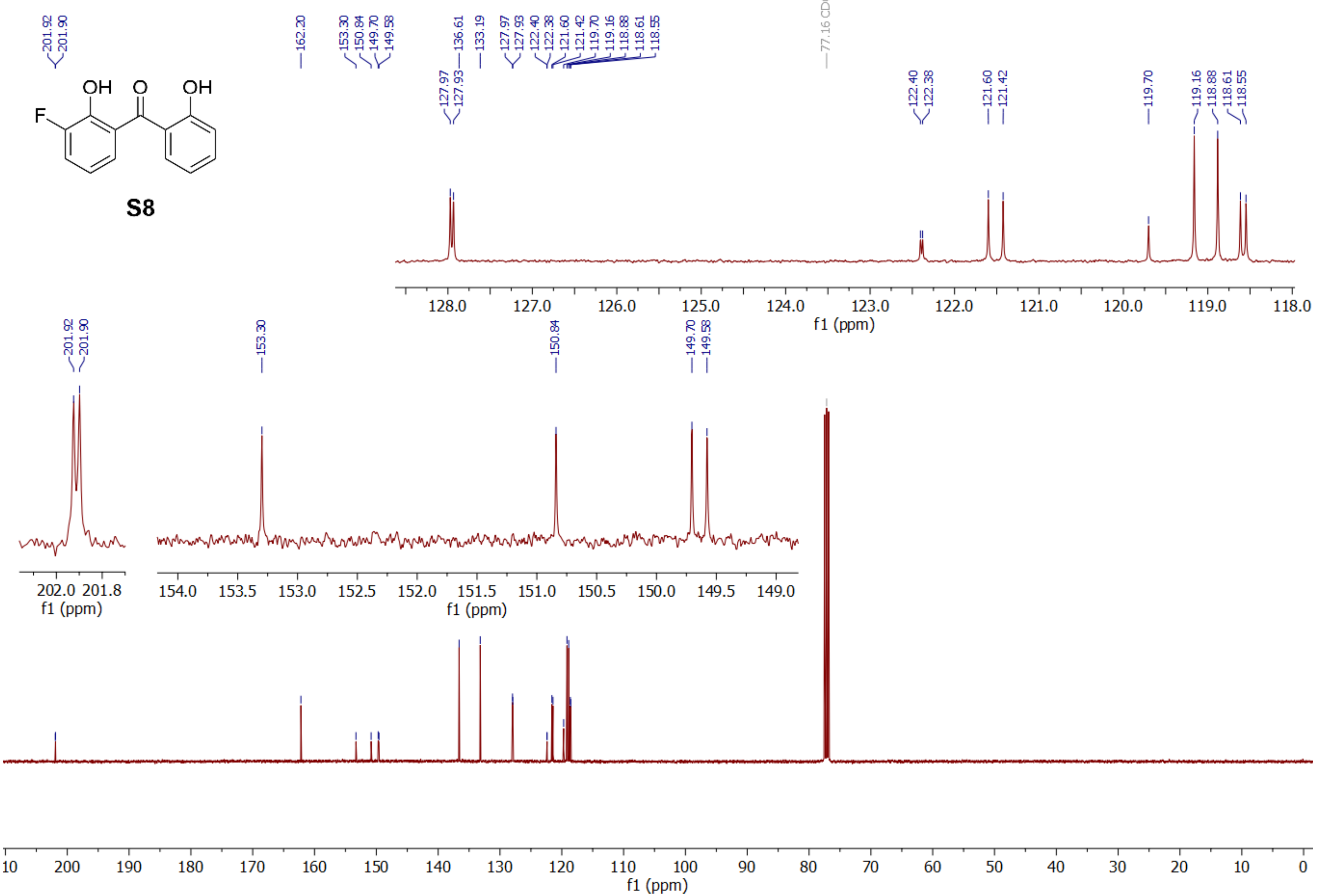

1H (400.15 MHz, CDCl3)

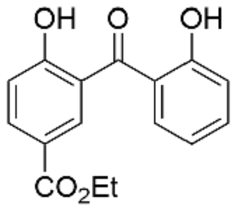


**S9**

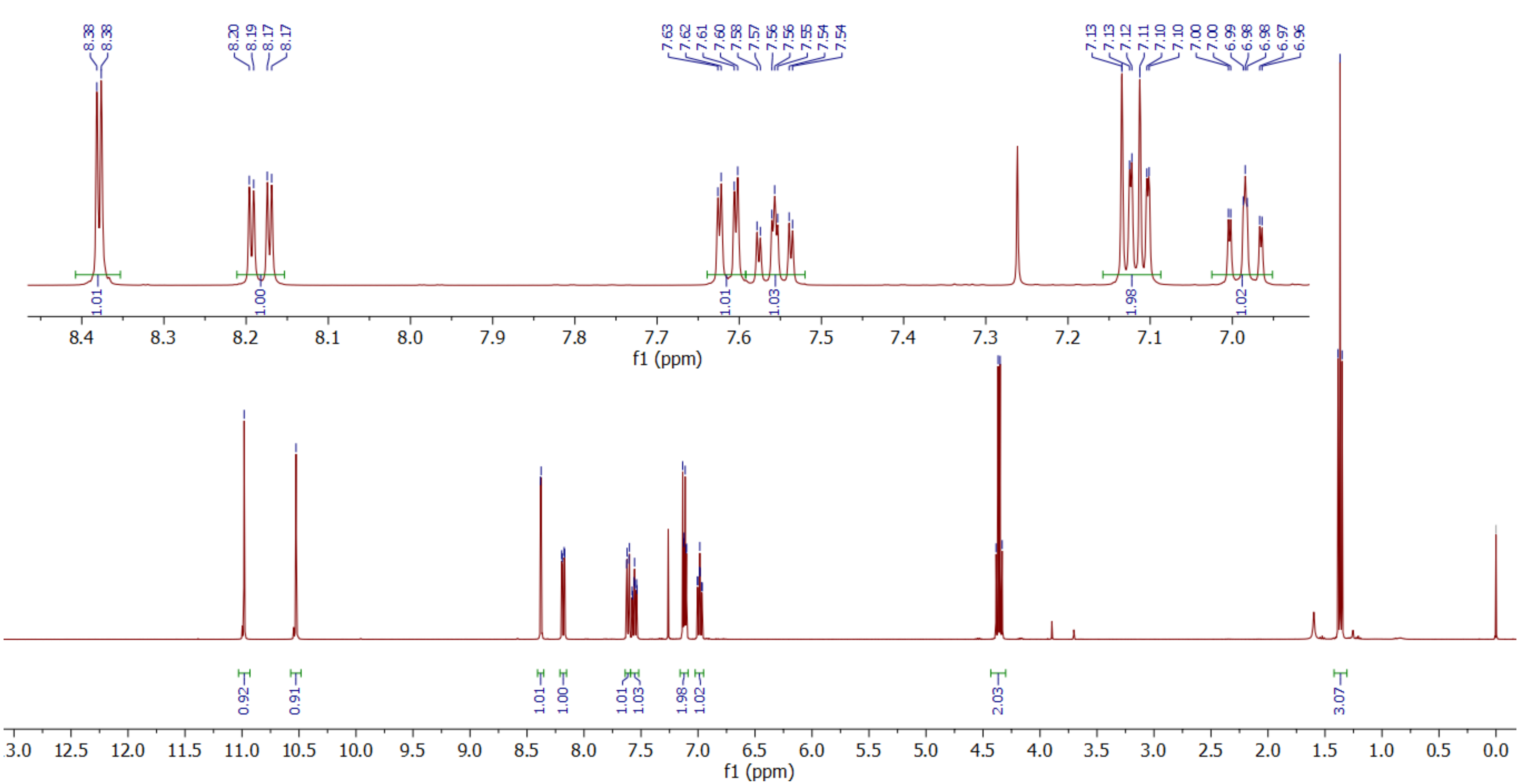

13C (100.63 MHz, CDCl3)

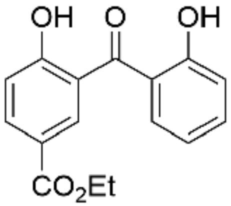

**S9**

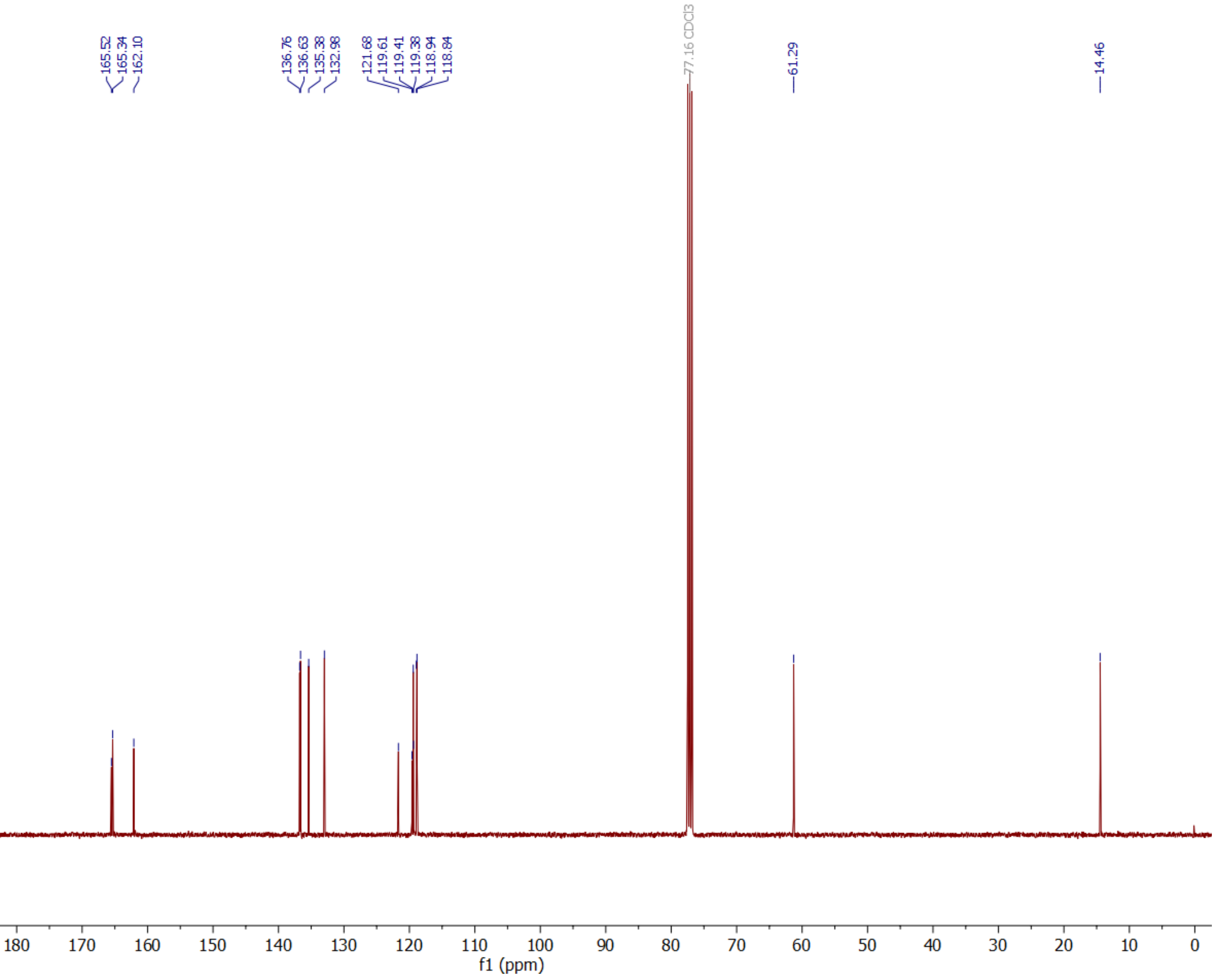

1H (400.15 MHz, CDCl3)

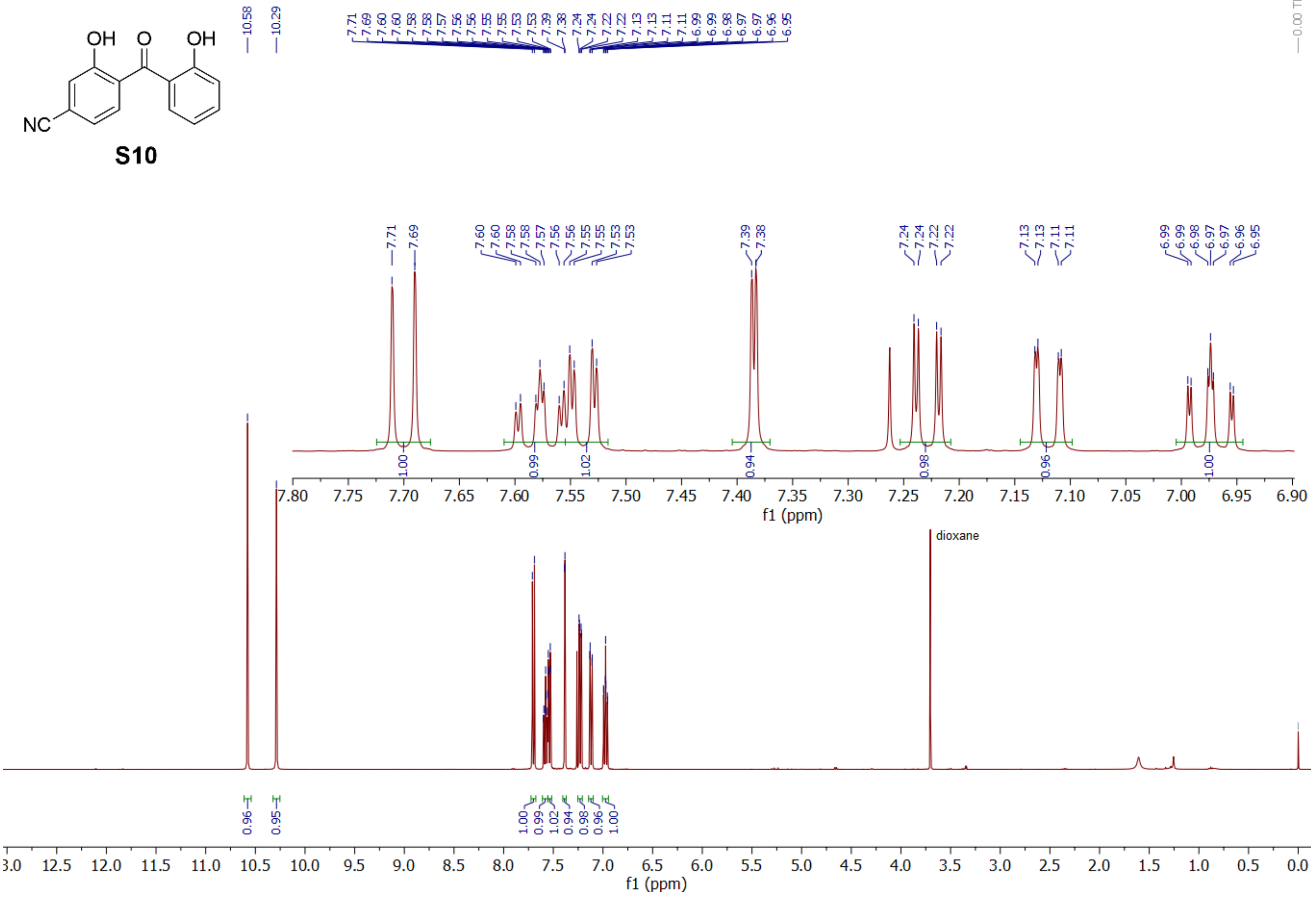

13C (100.63 MHz, CDCl3)

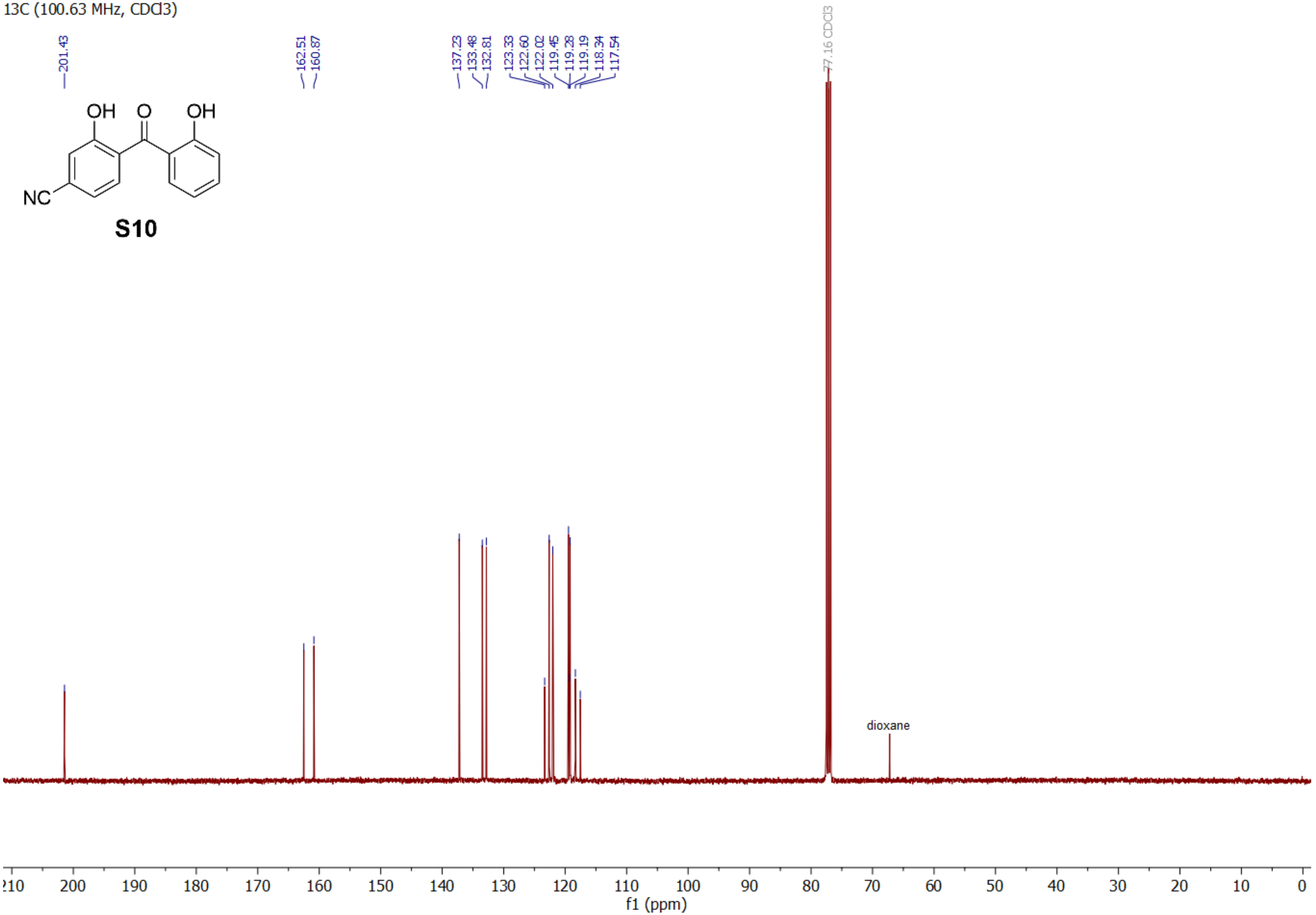


**S10**

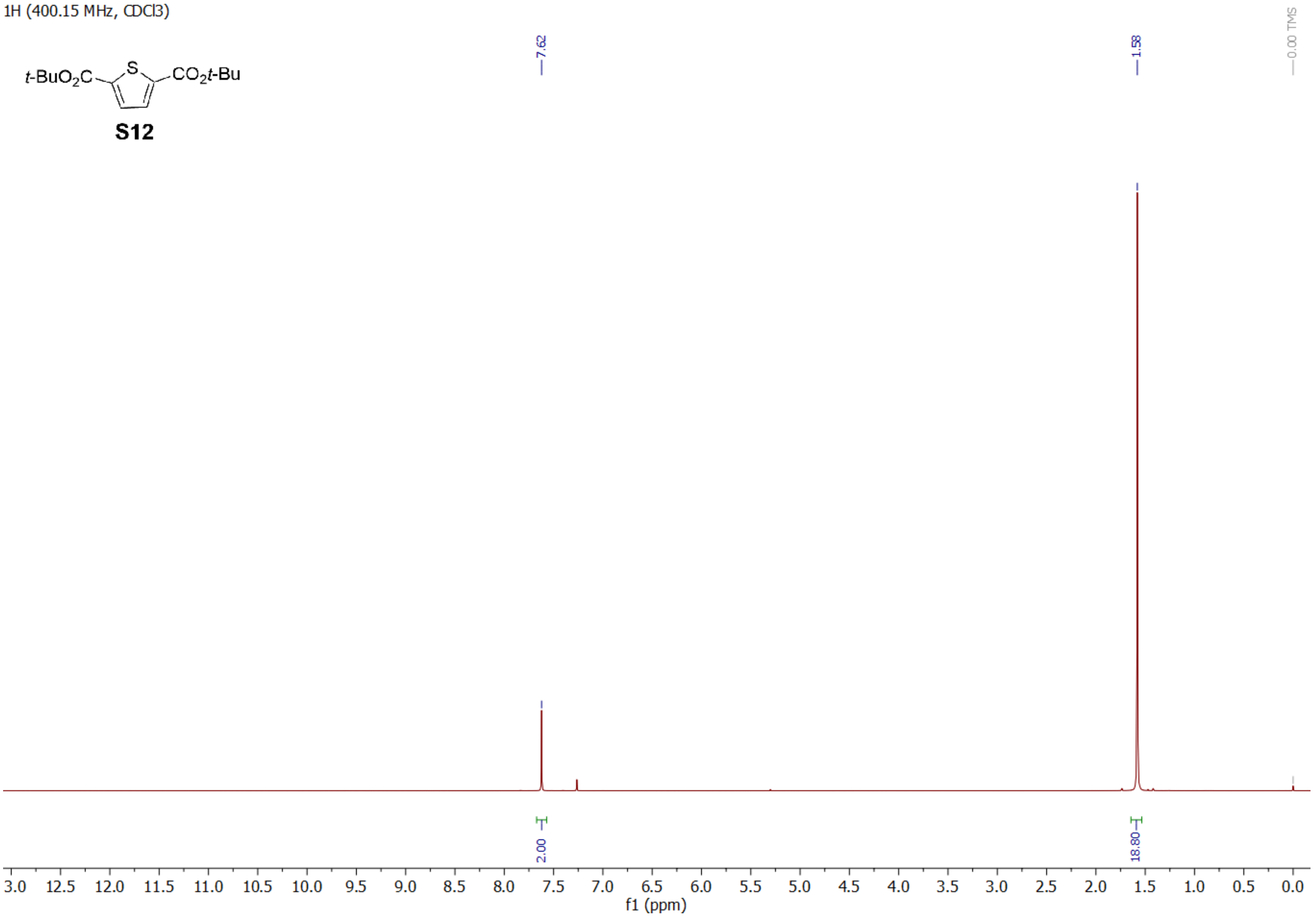
1H (400.15 MHz, CDCl3)
t-BuO2C
S
CO2t-Bu
S12
7.62
1.58
0.00 TMS
2.00
18.80
3.0
12.5
12.0
11.5
11.0
10.5
10.0
9.5
9.0
8.5
8.0
7.5
7.0
6.5
6.0
5.5
5.0
4.5
4.0
3.5
3.0
2.5
2.0
1.5
1.0
0.5
0.0
f1 (ppm)

13C (100.63 MHz, CDCl3)

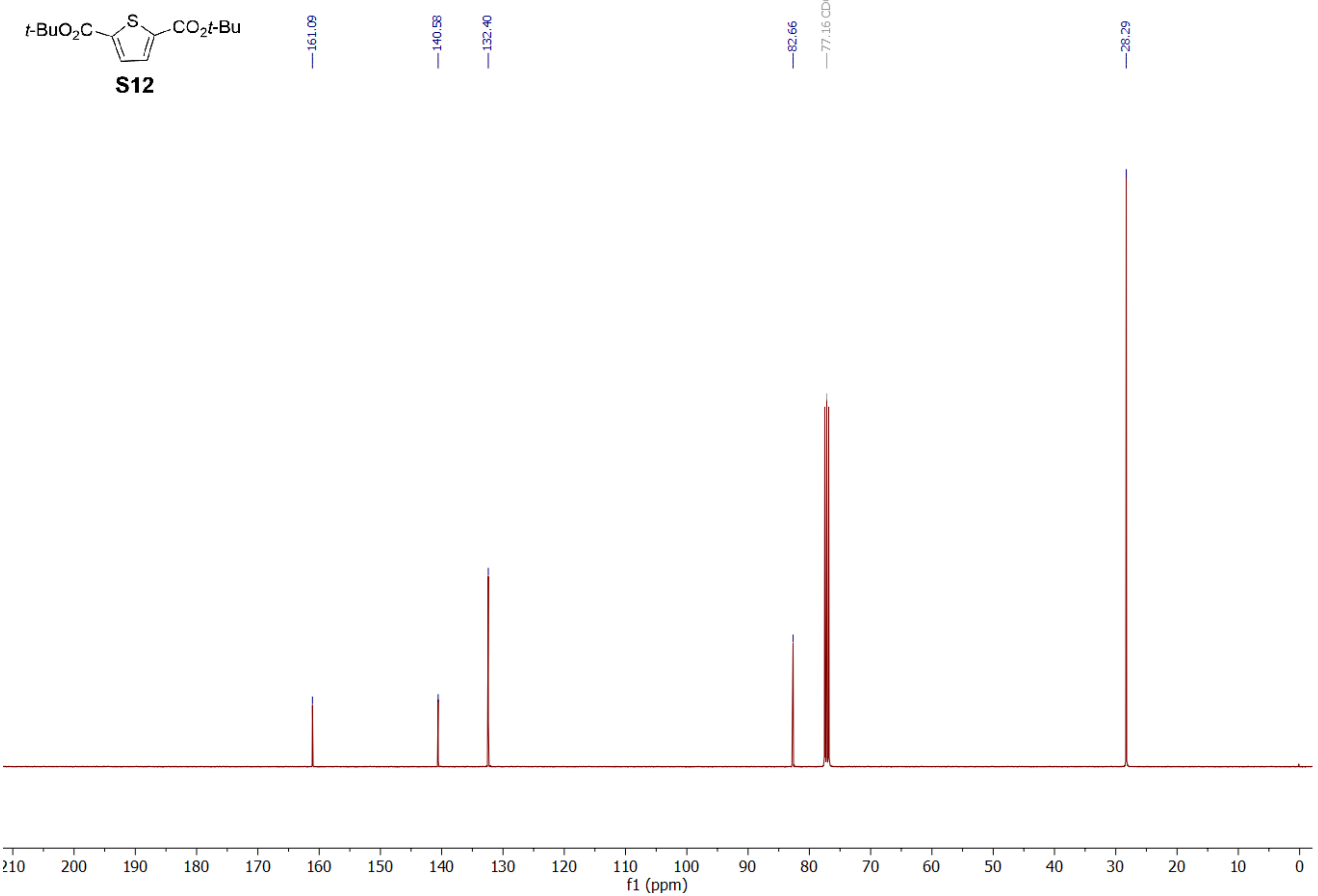

1H (400.15 MHz, CDCl3)

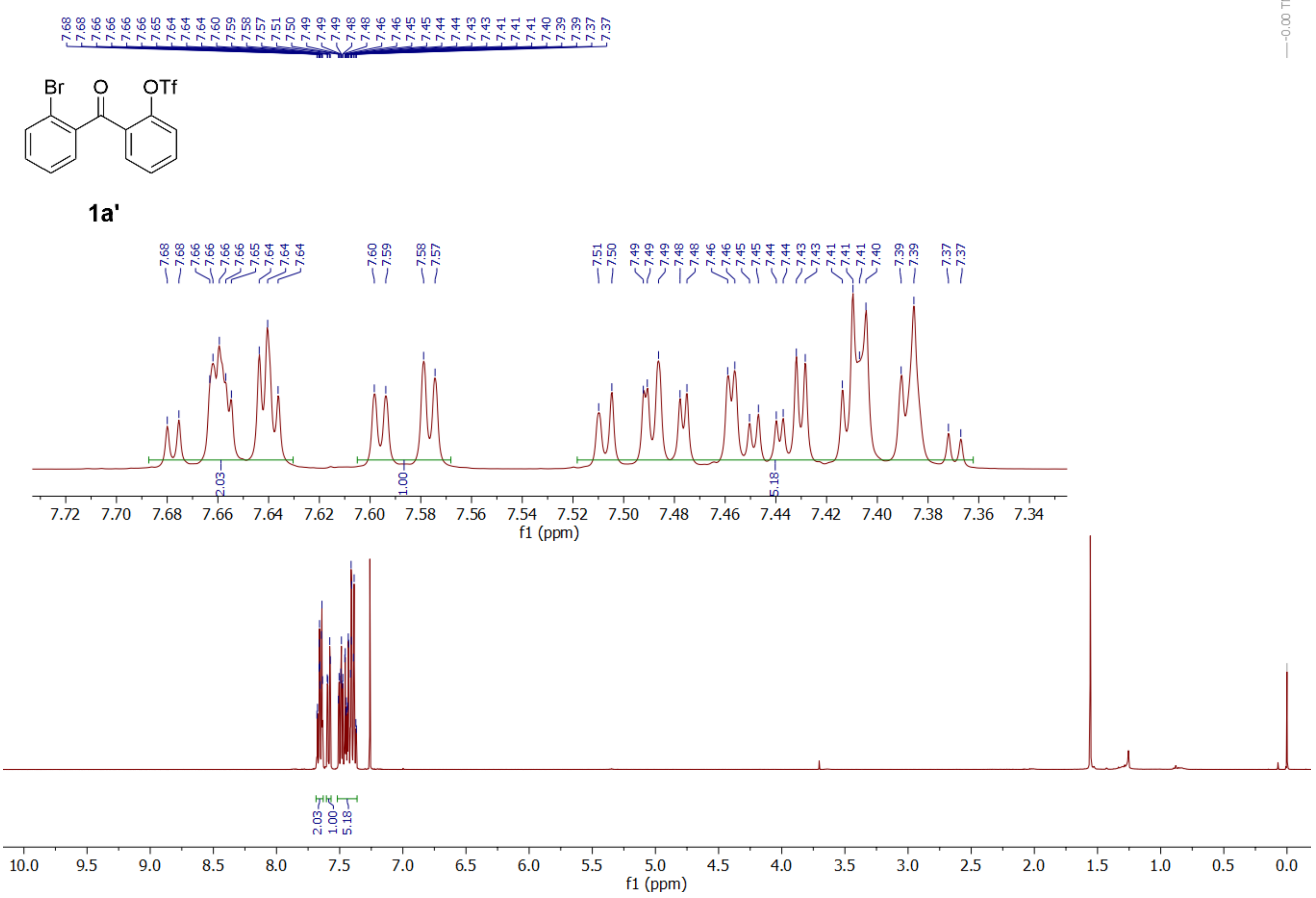

19F (376.48 MHz, CDCl3)

-73.32

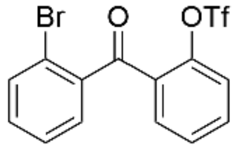


**1a'**

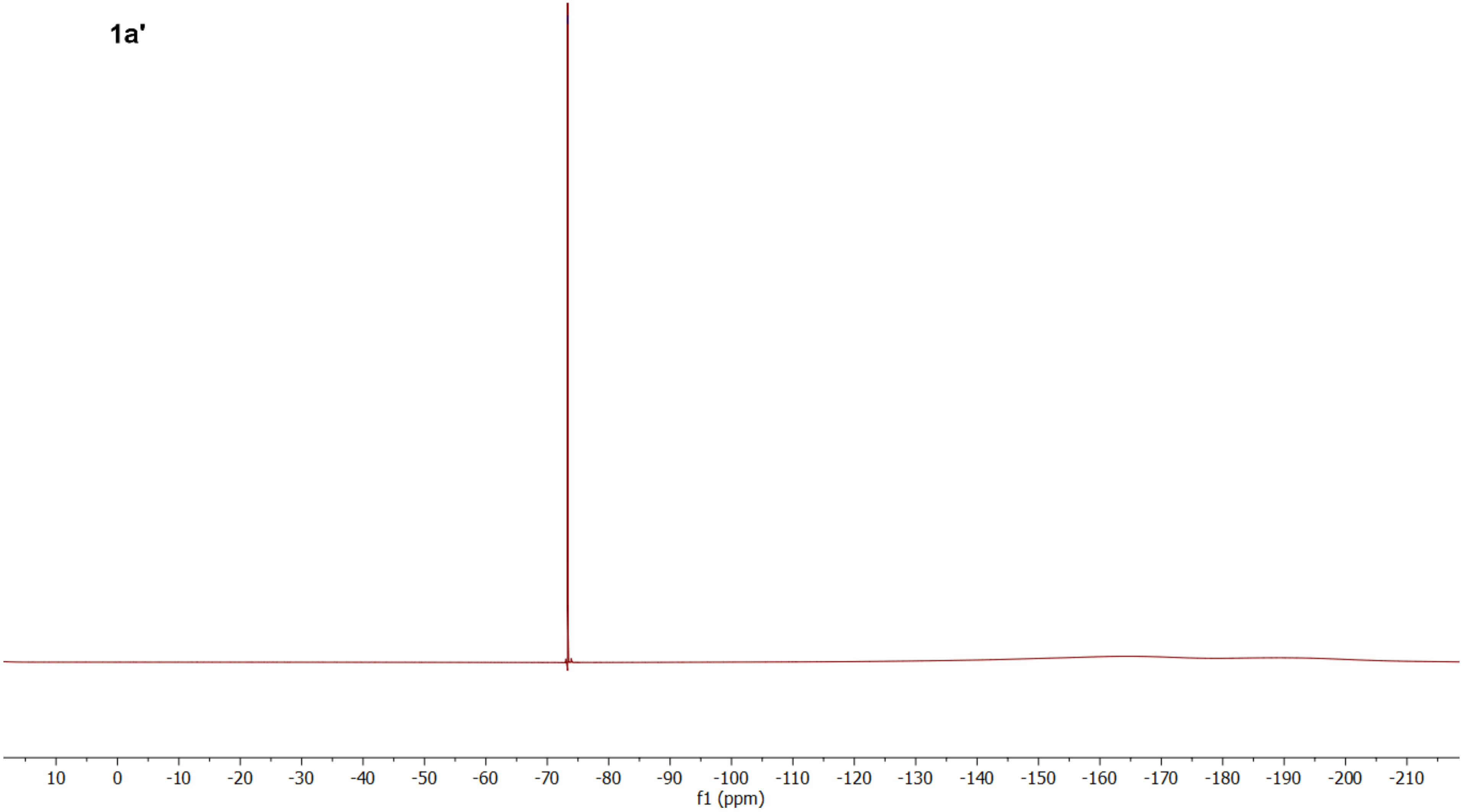

13C (100.63 MHz, CDCl3)

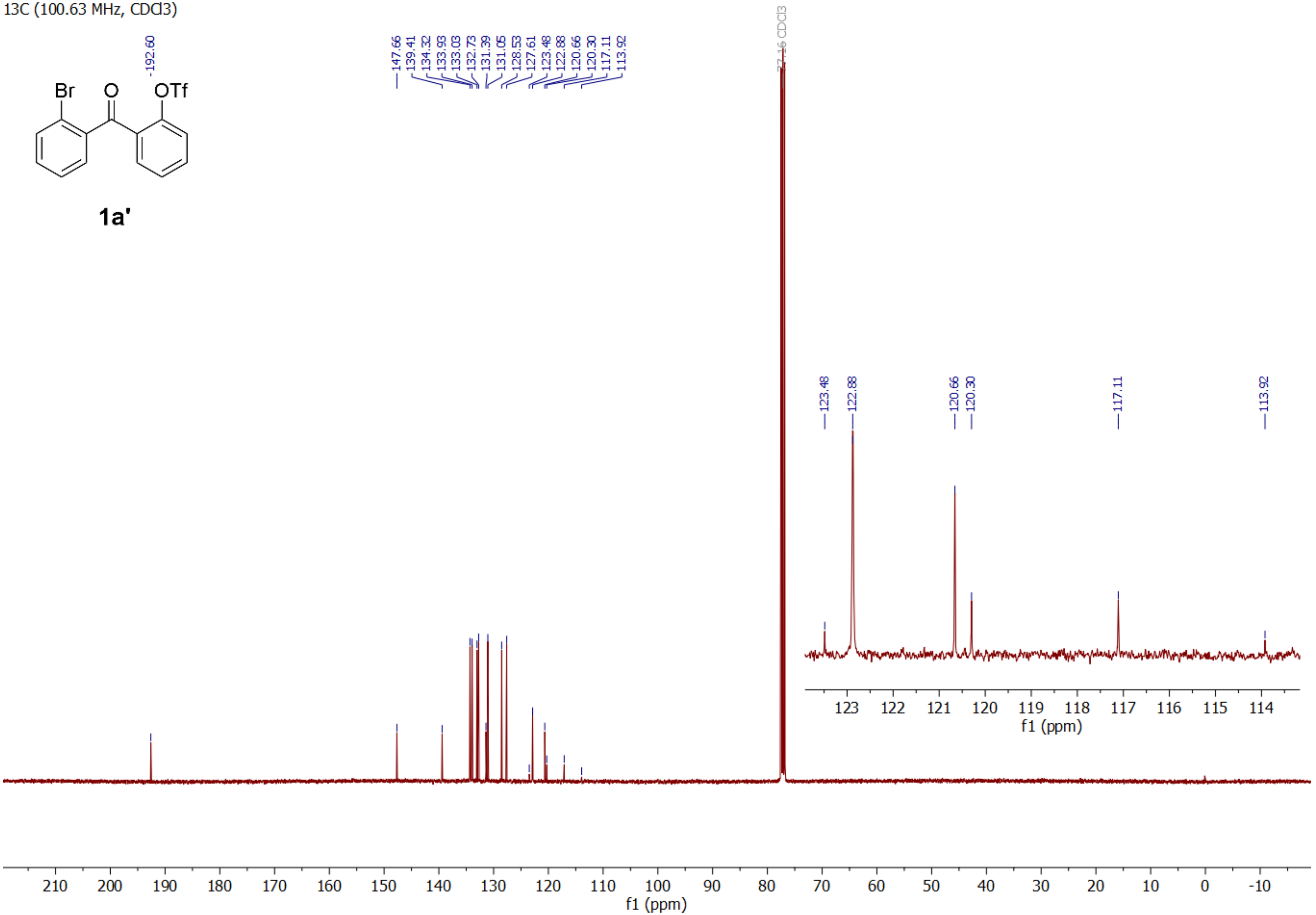


**1a'**

1H (400.15 MHz, CDCl3)

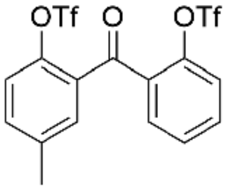


**1b**

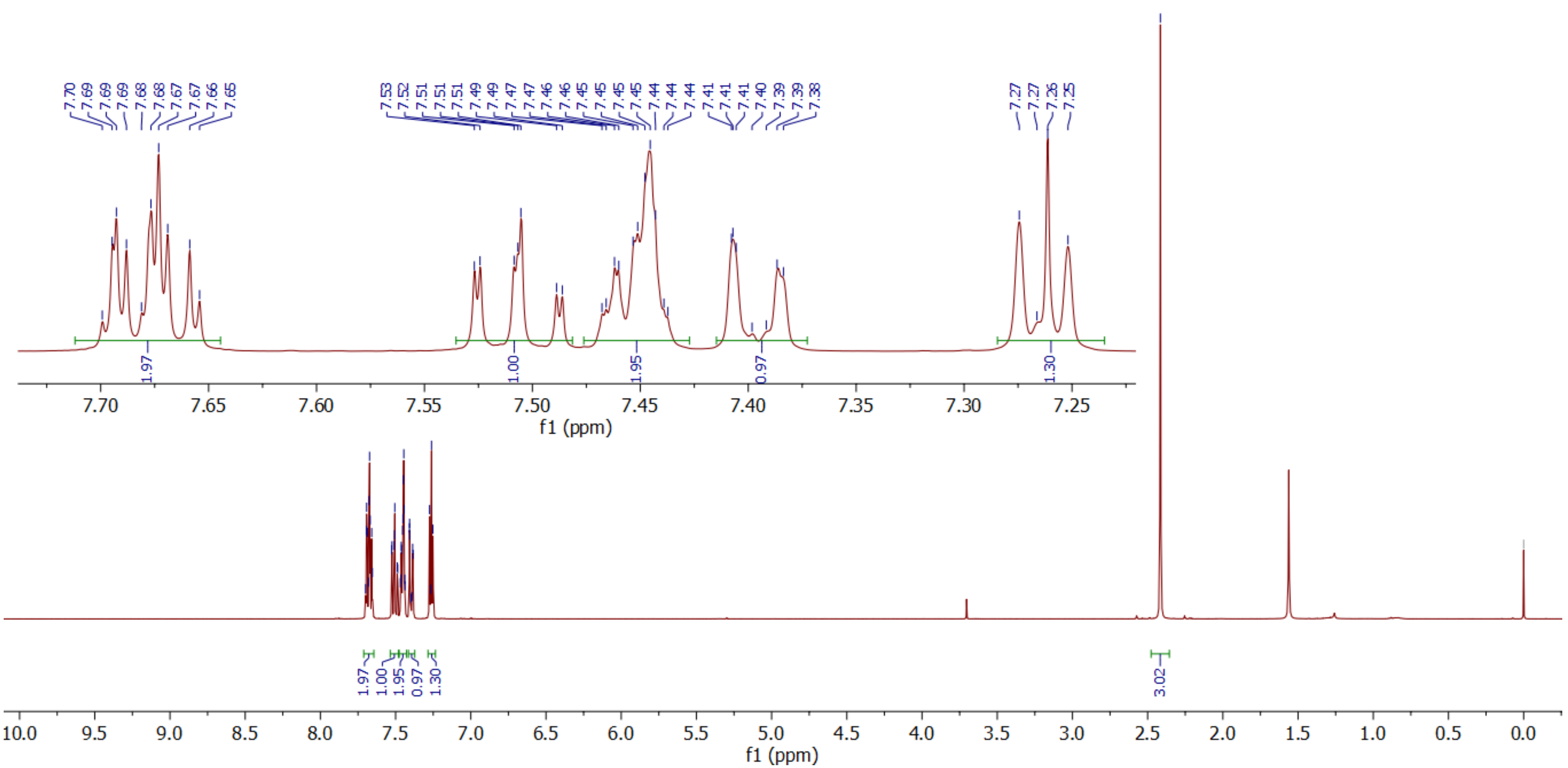

19F (376.48 MHz, CDCl3)

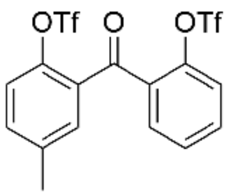


**1b**

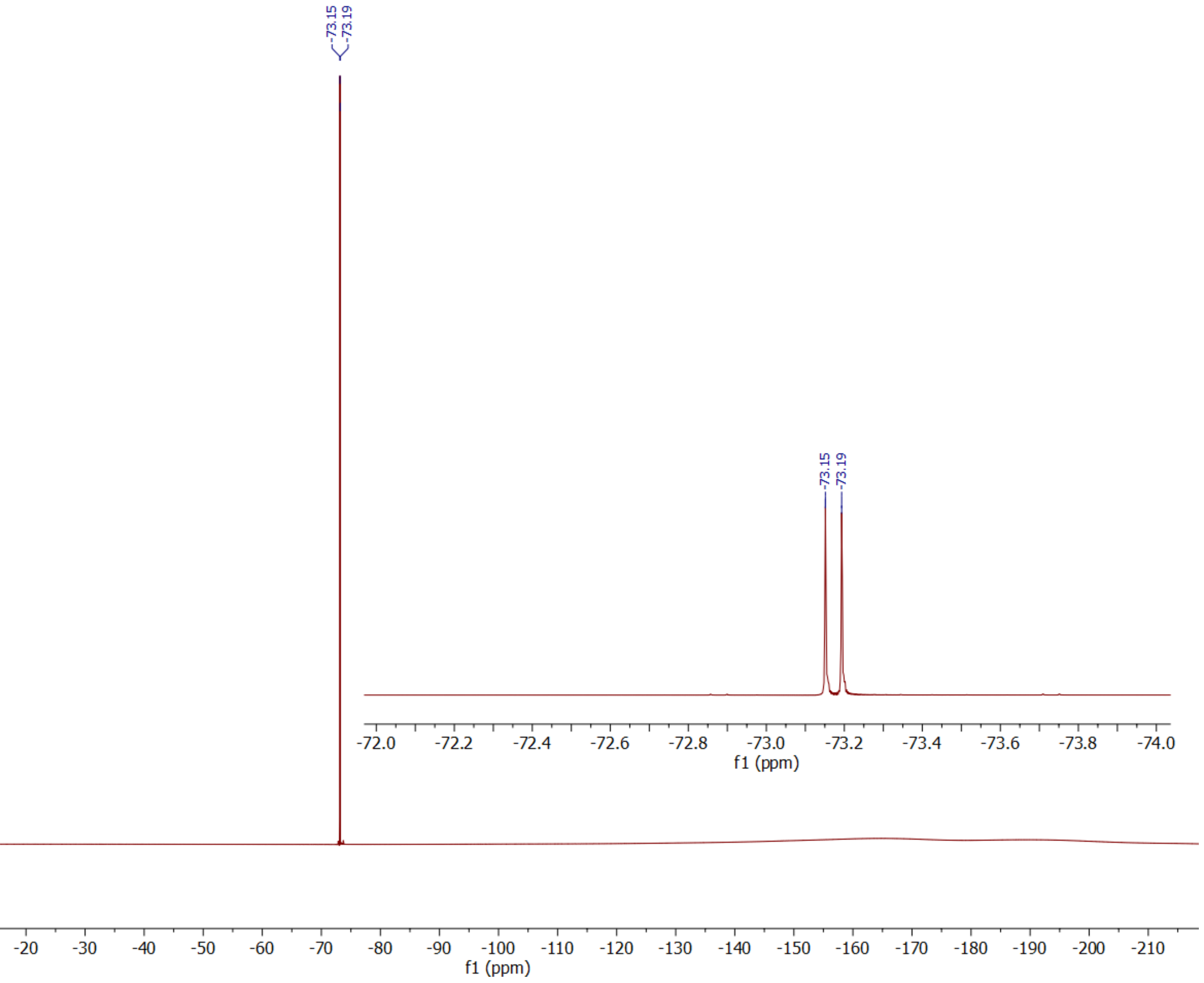

13C (100.63 MHz, CDCl3)

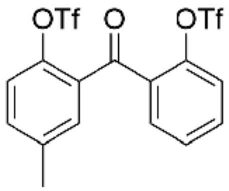

**1b**

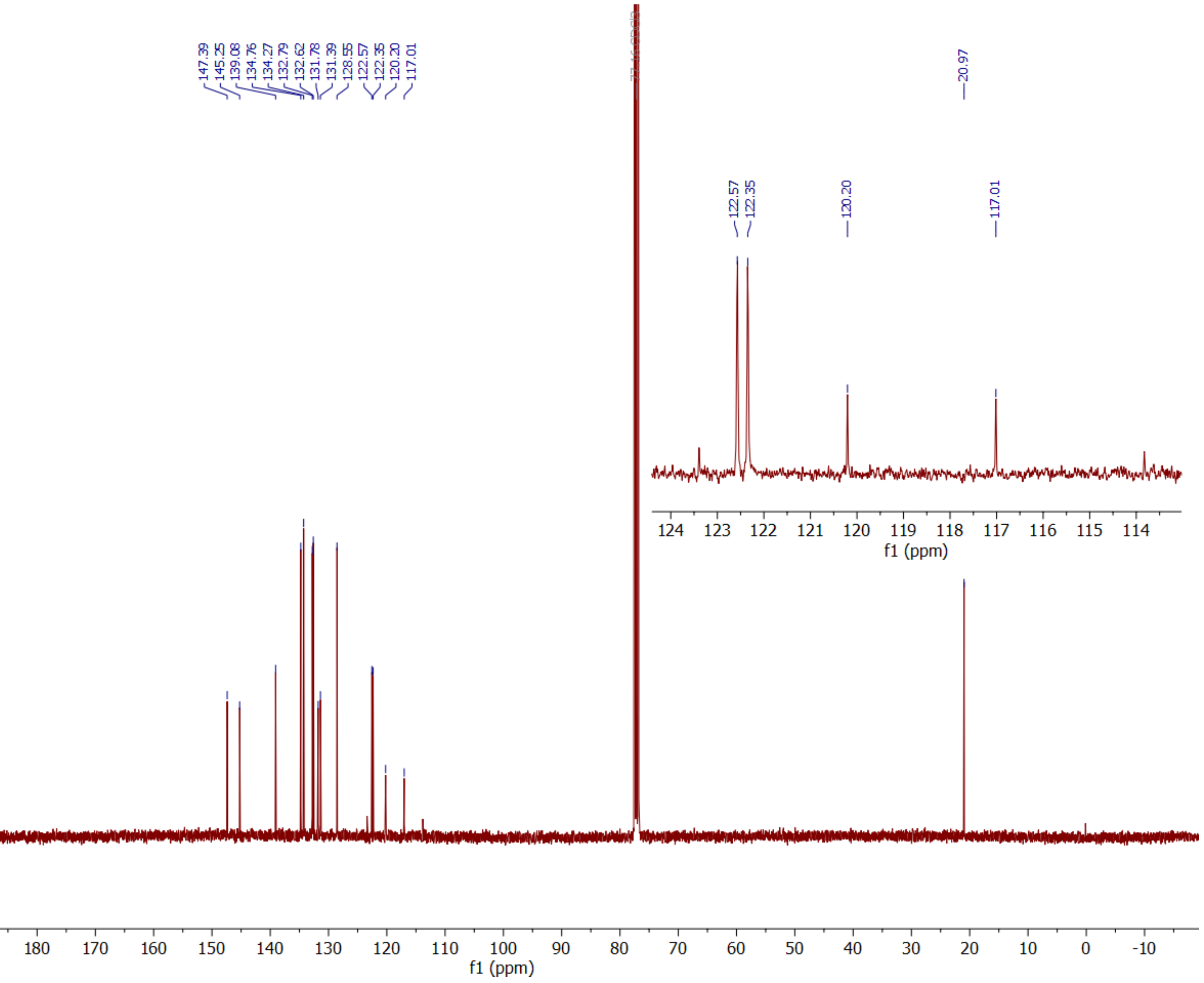

1H (400.15 MHz, CDCl3)

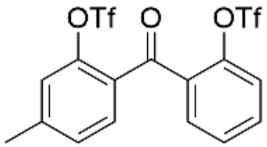

1c

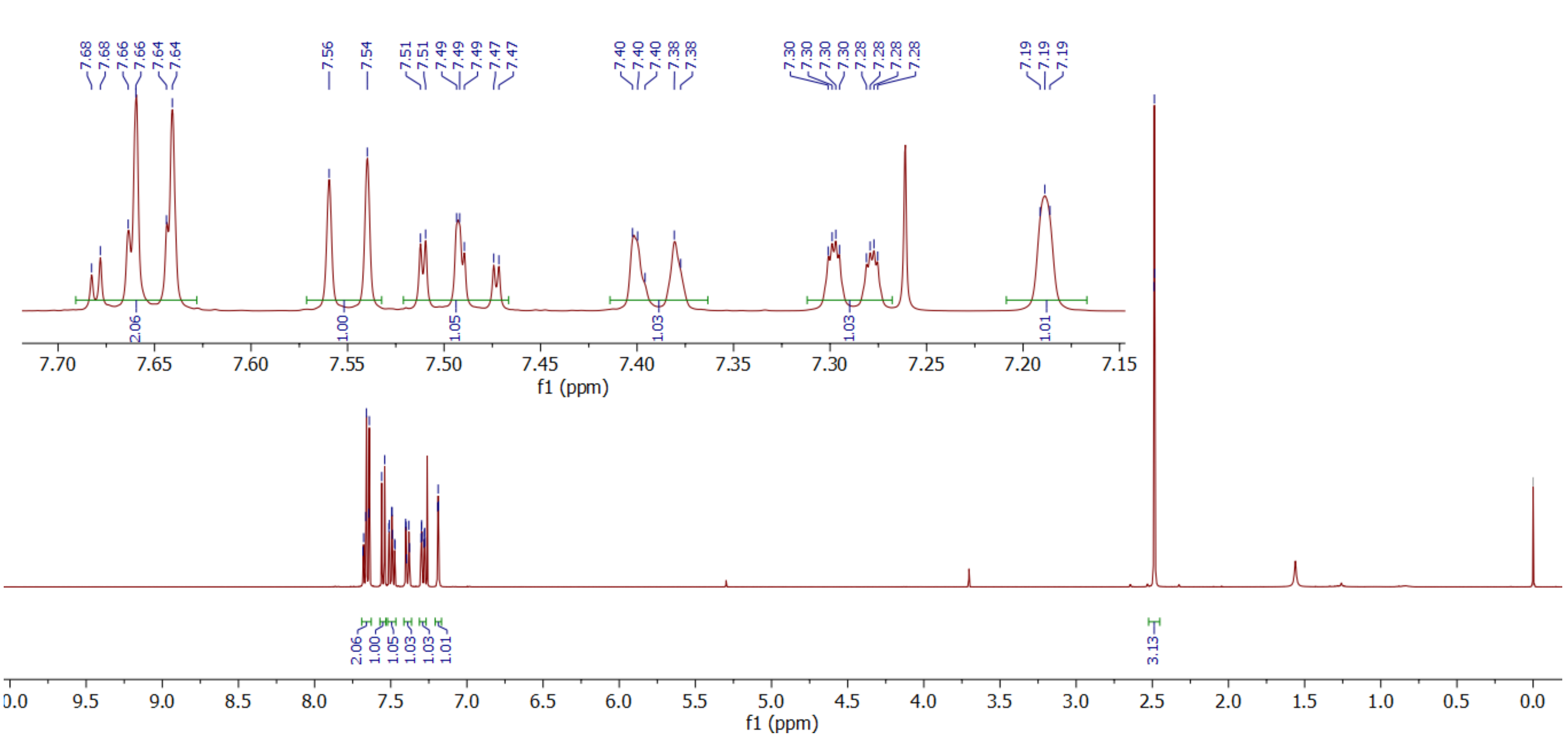

19F (376.48 MHz, CDCl3)

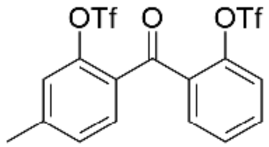


**1c**

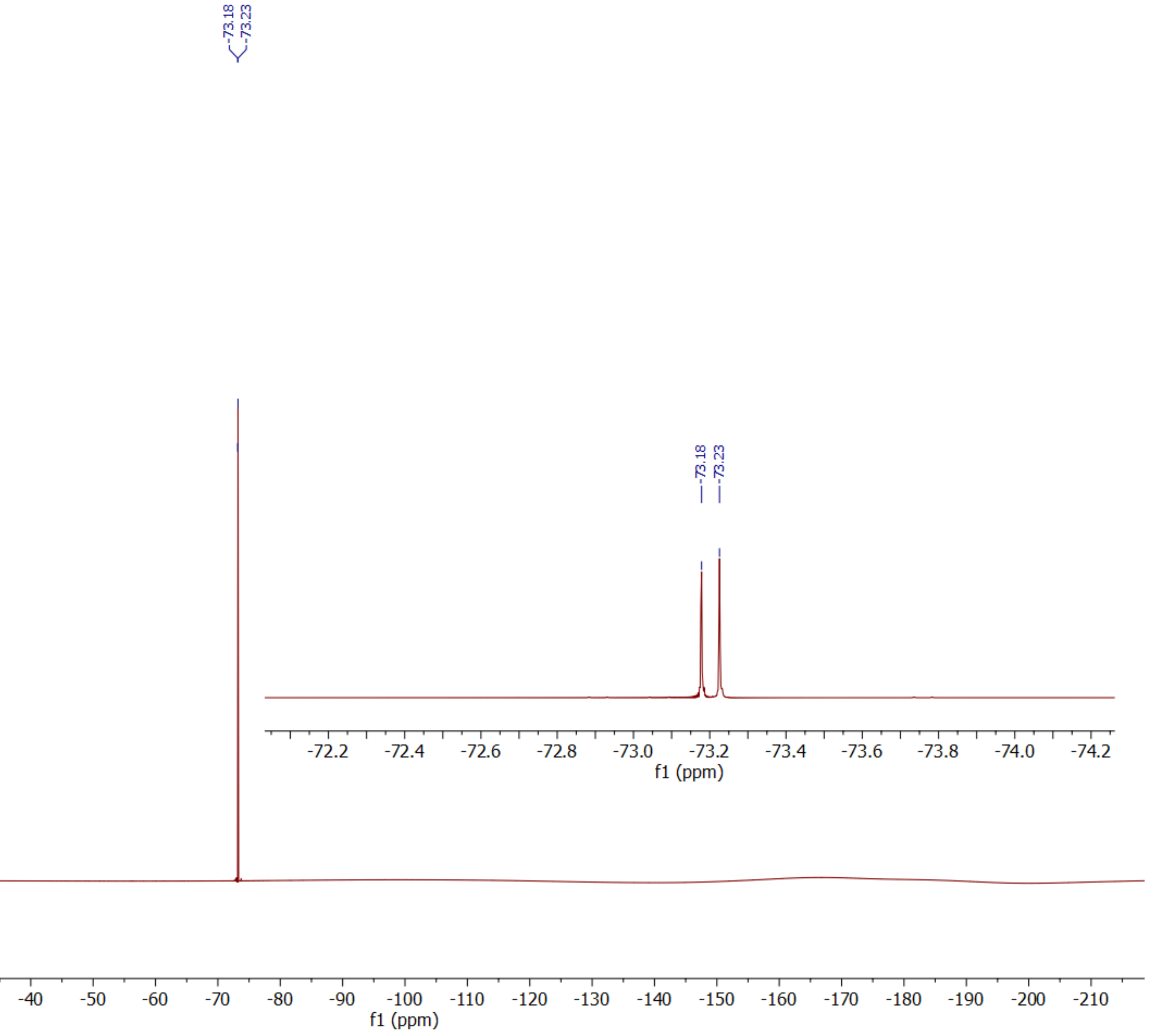

13C (100.63 MHz, CDCl3)

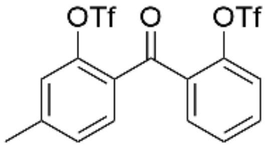

1c

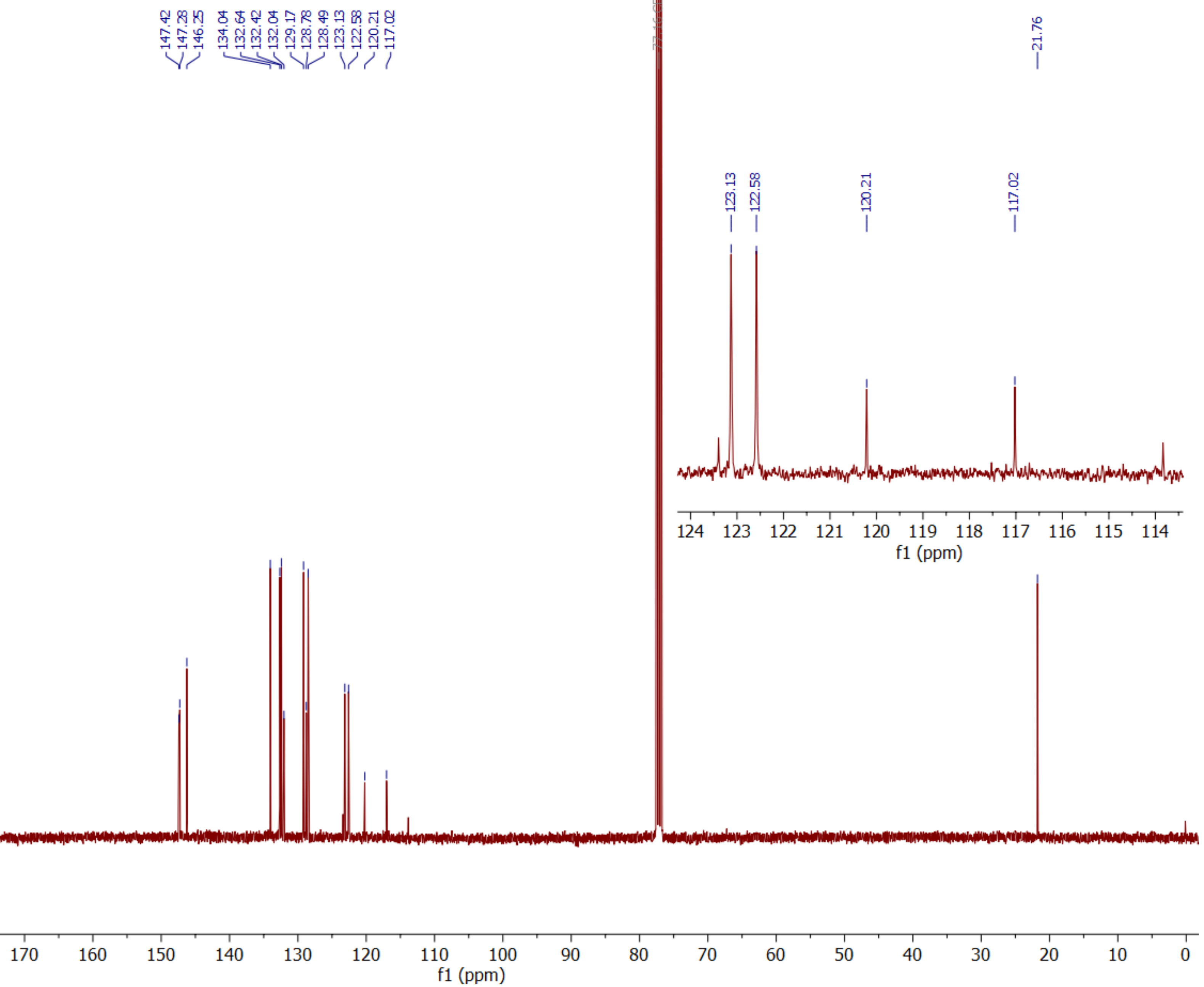

1H (400.15 MHz, CDCl3)

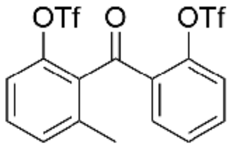


**1d**

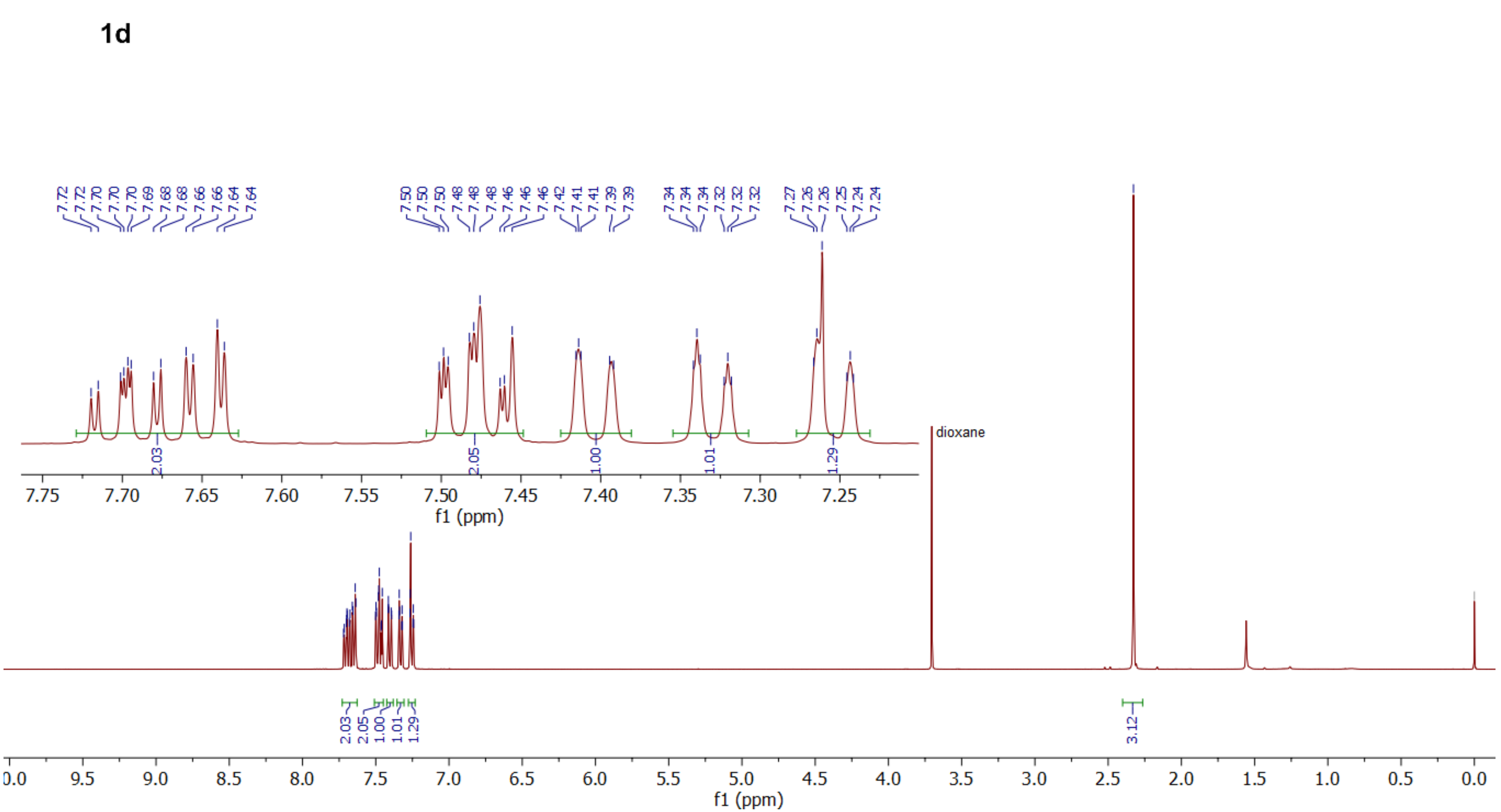

19F (376.48 MHz, CDCl3)

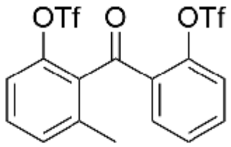

**1d**

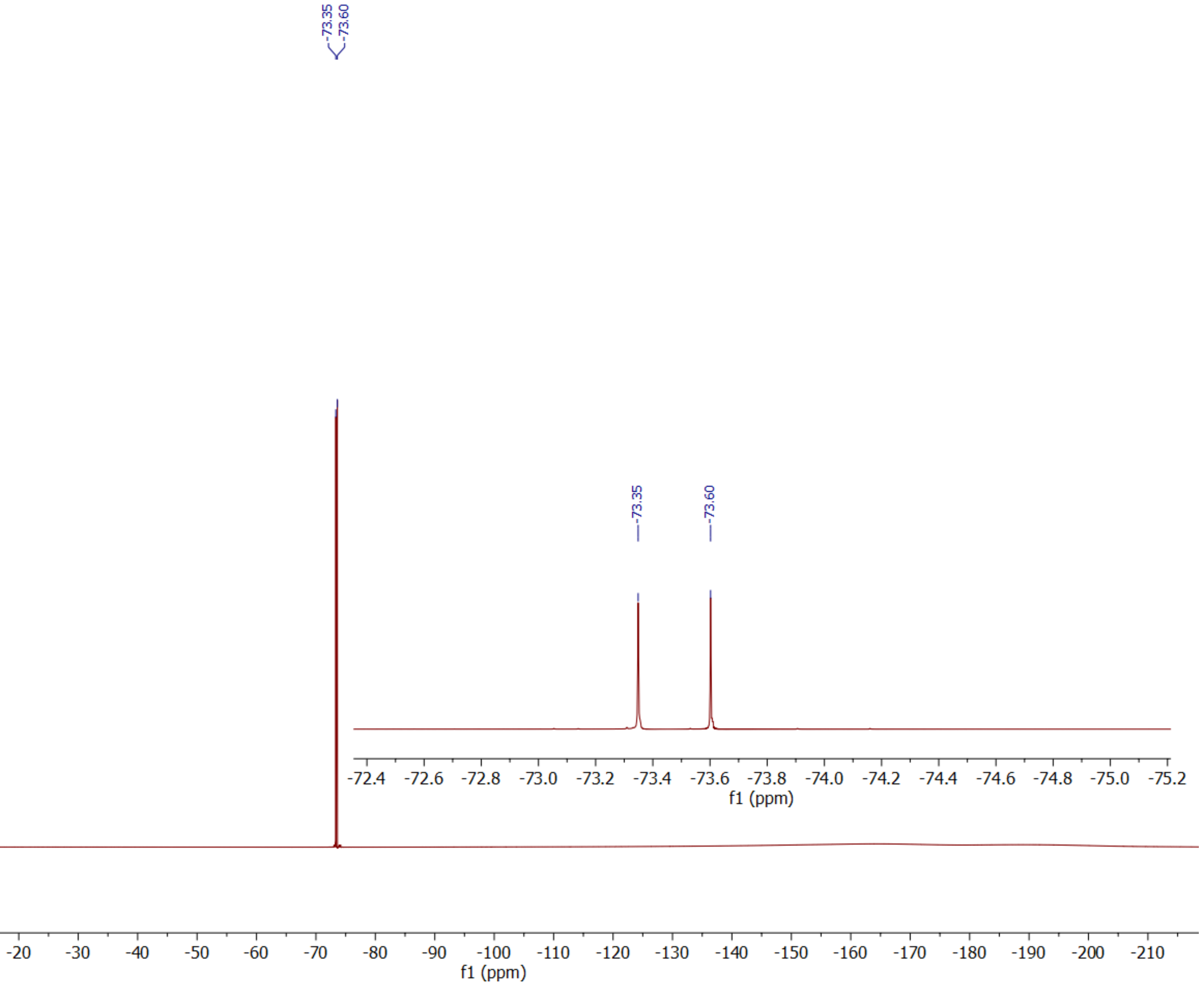

13C (100.63 MHz, CDCl3)

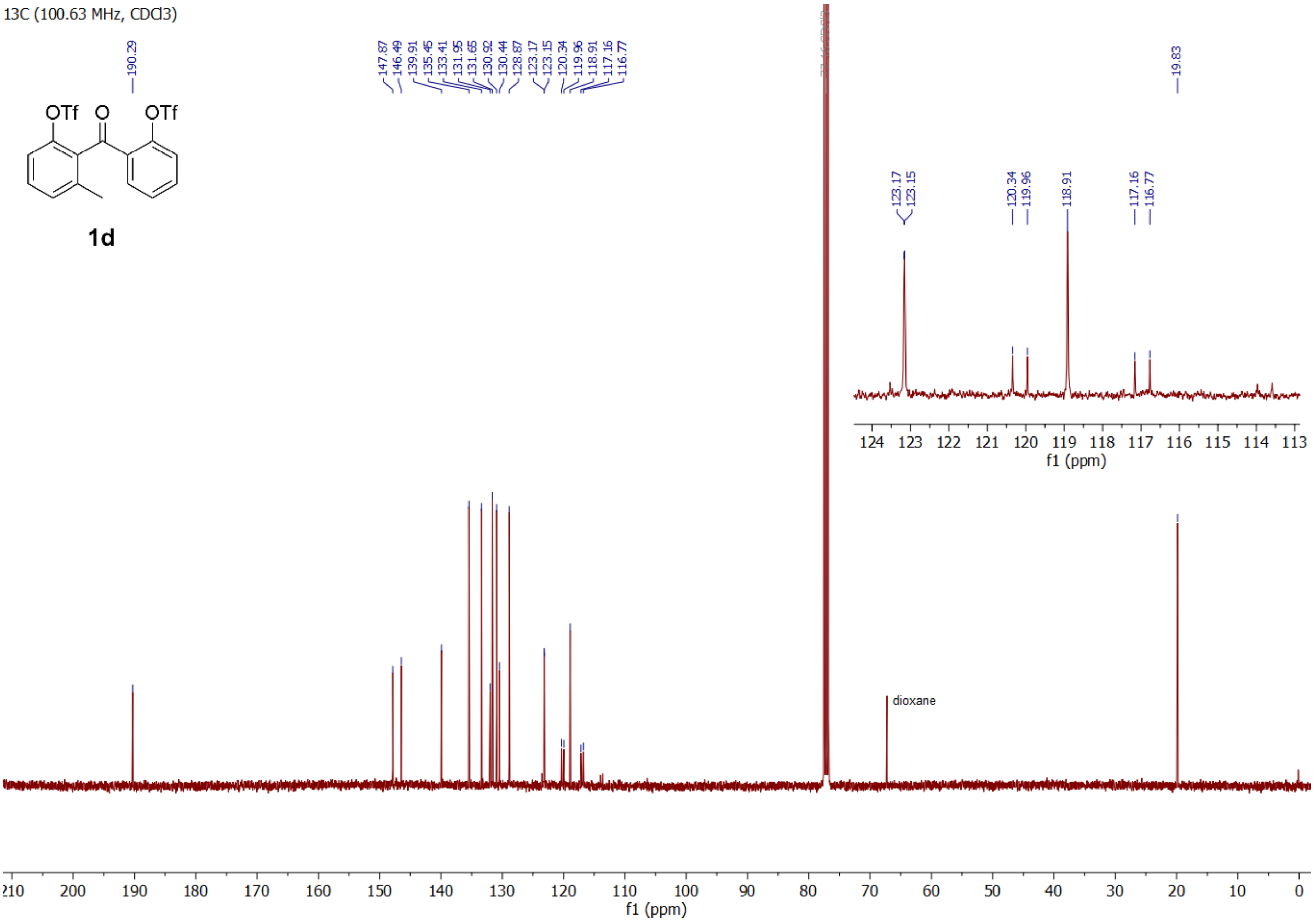

1H (400.15 MHz, CDCl3)

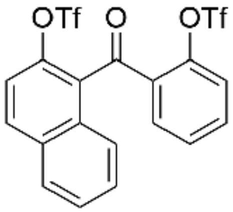


**1e**

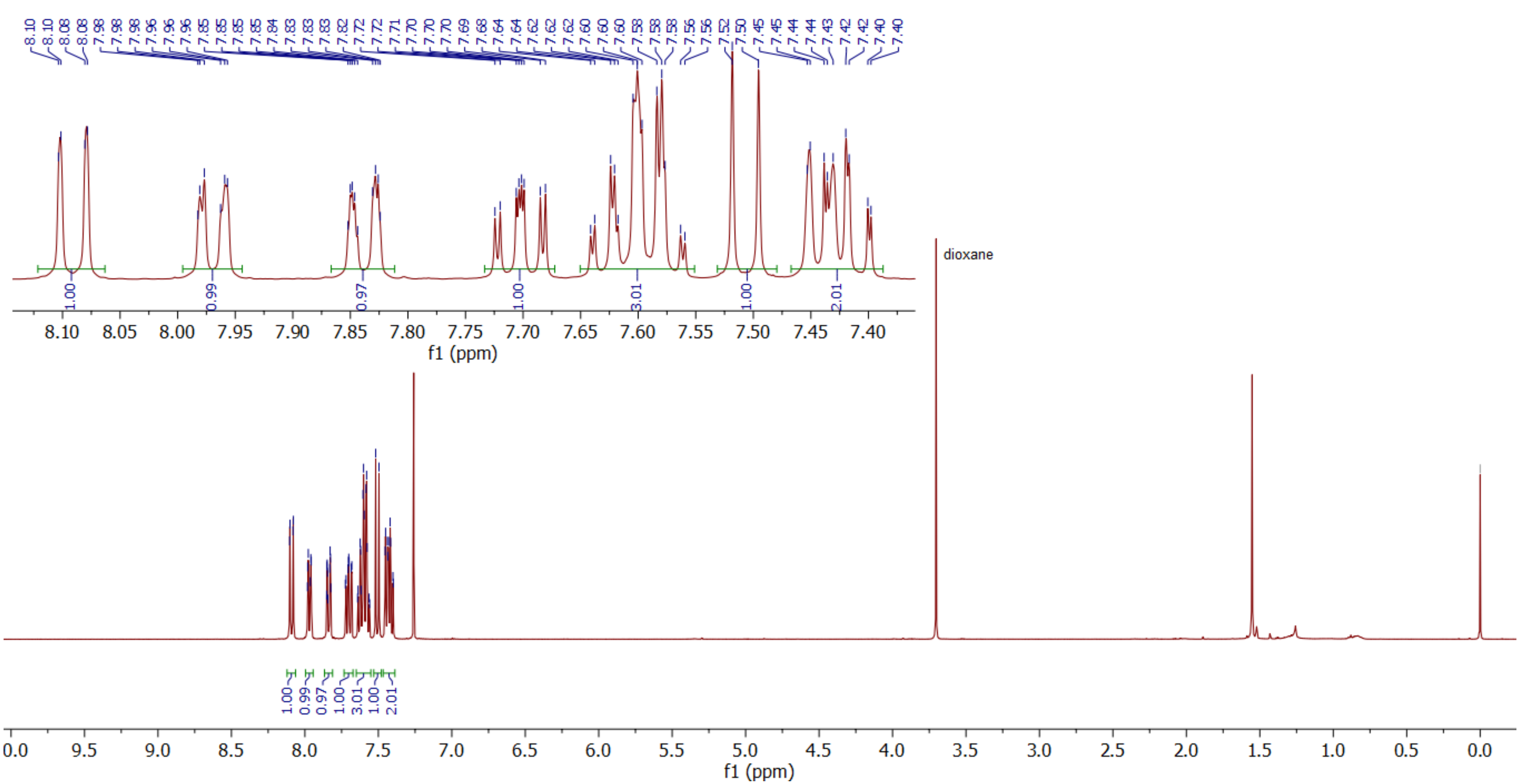

19F (376.48 MHz, CDCl3)

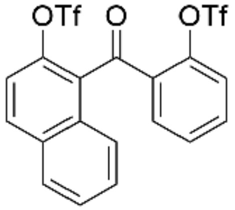

**1e**

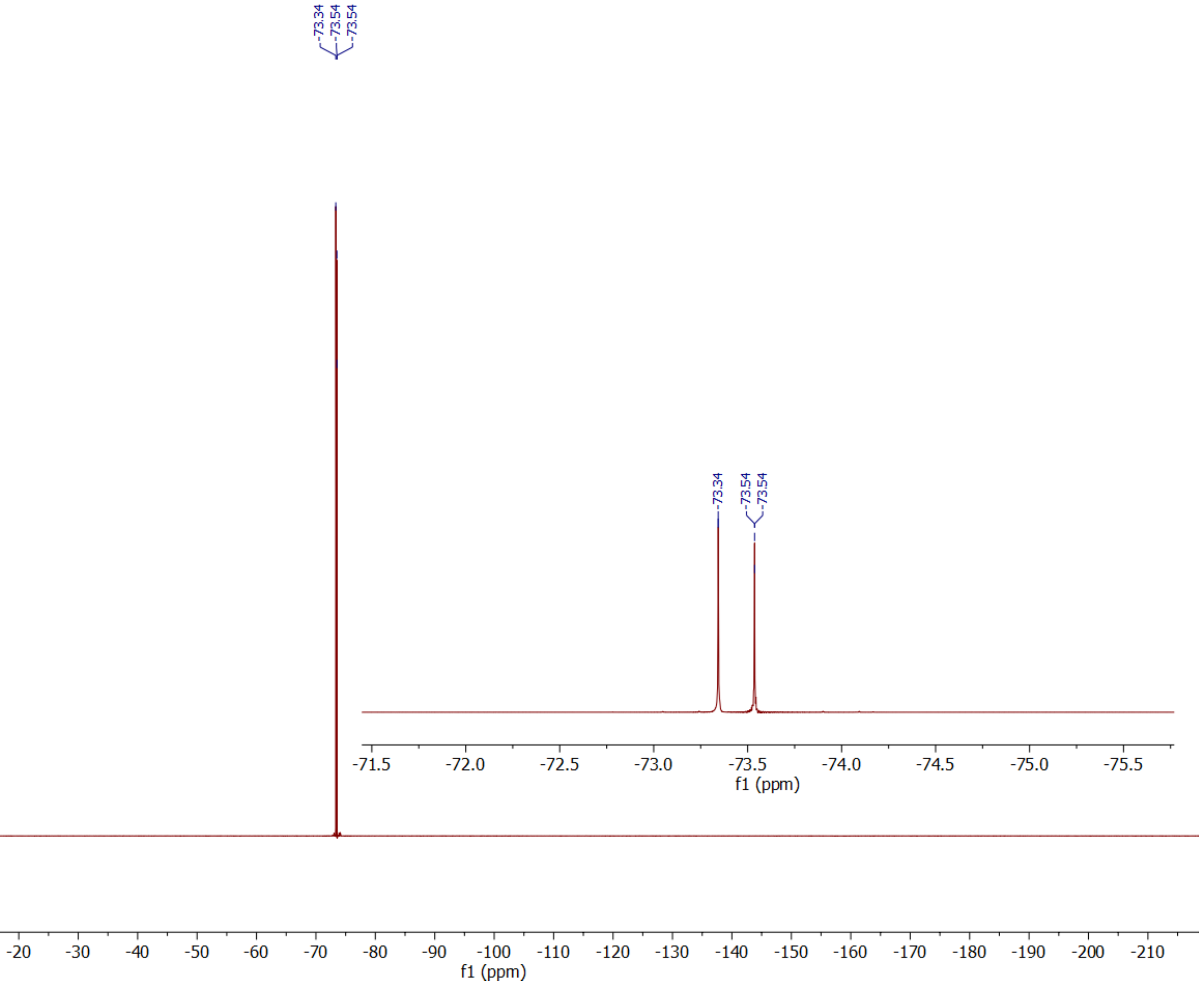

13C (100.63 MHz, CDCl3)

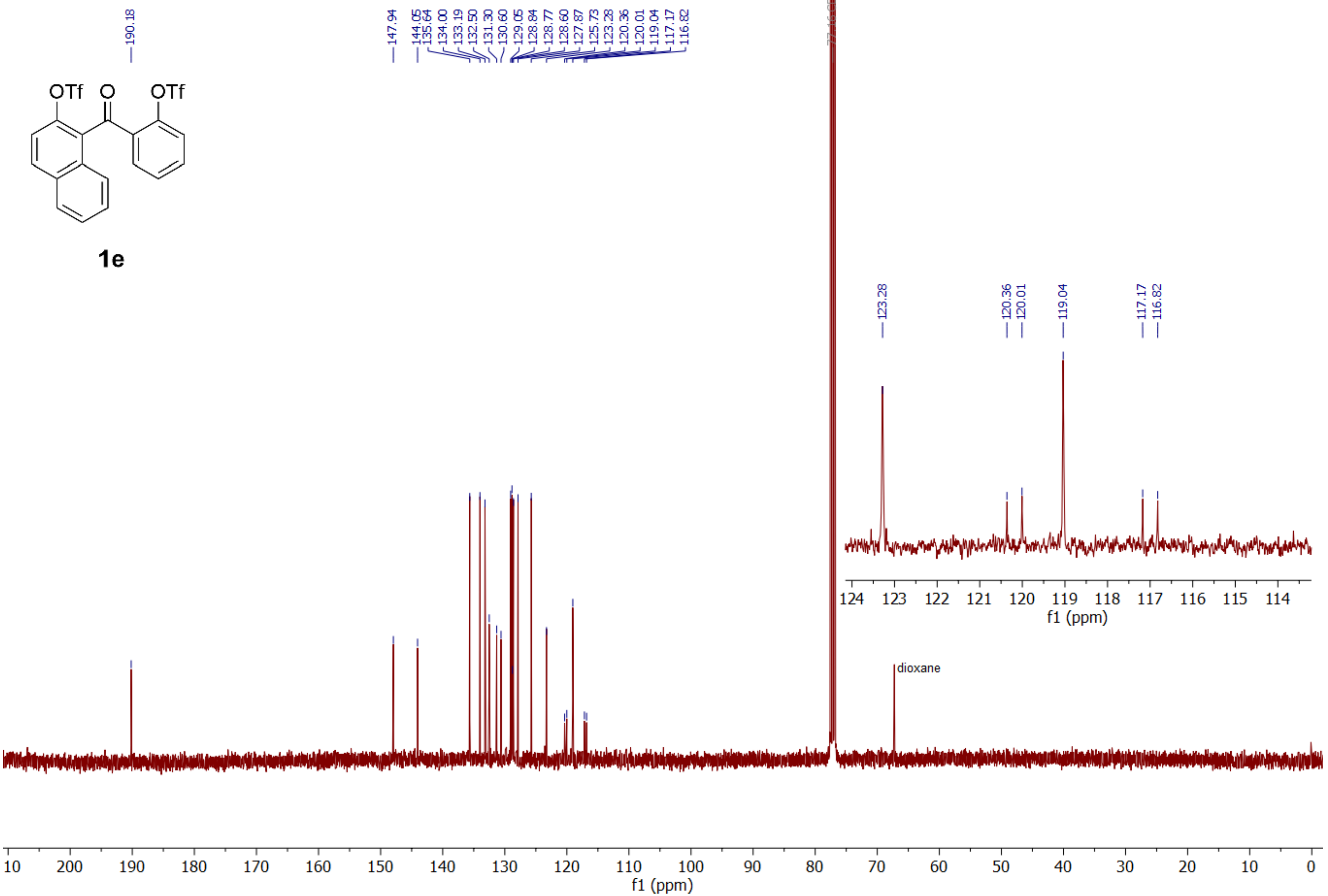


1e

1H (400.15 MHz, CDCl3)

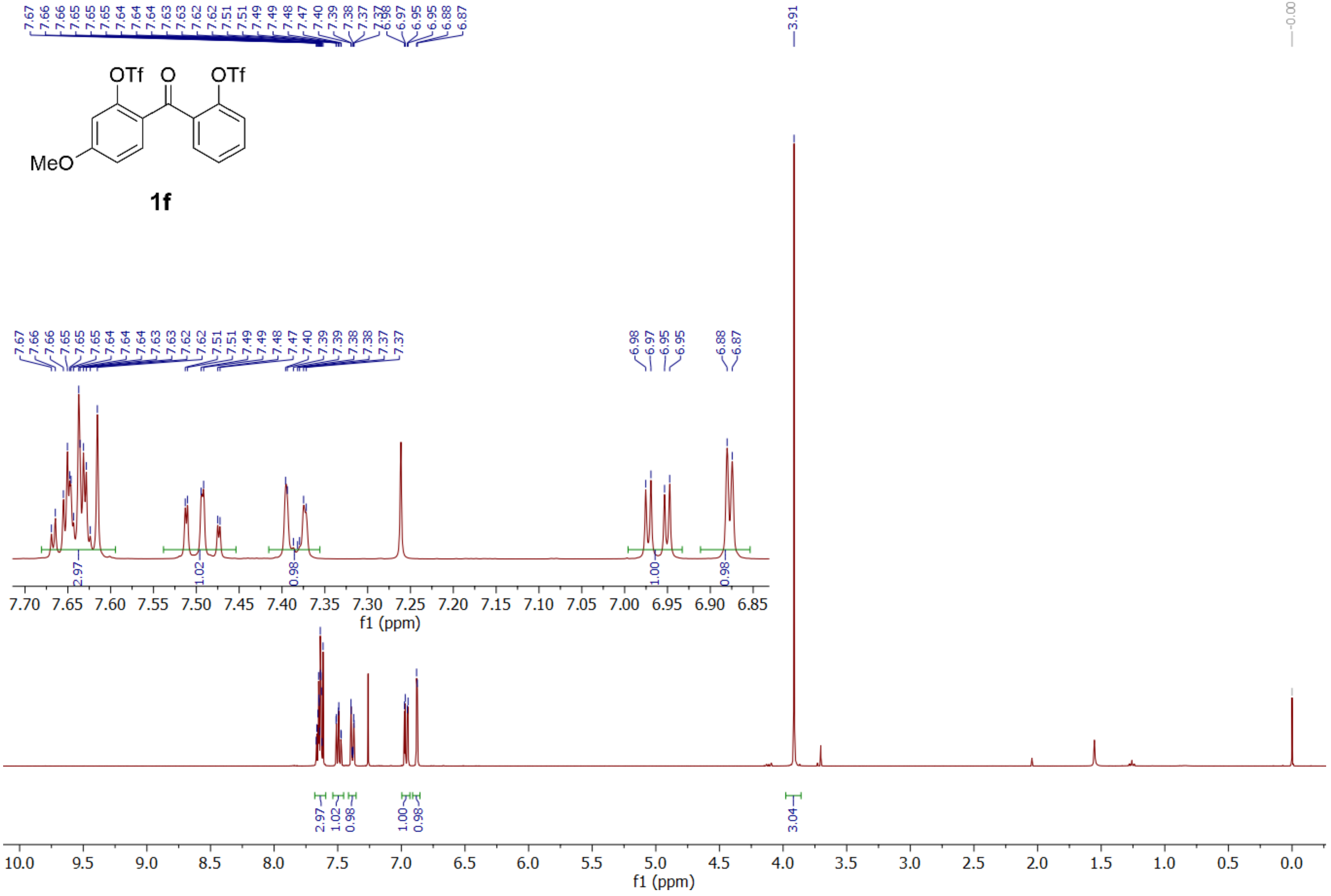

19F (376.48 MHz, CDCl3)

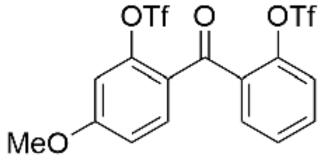


**1f**

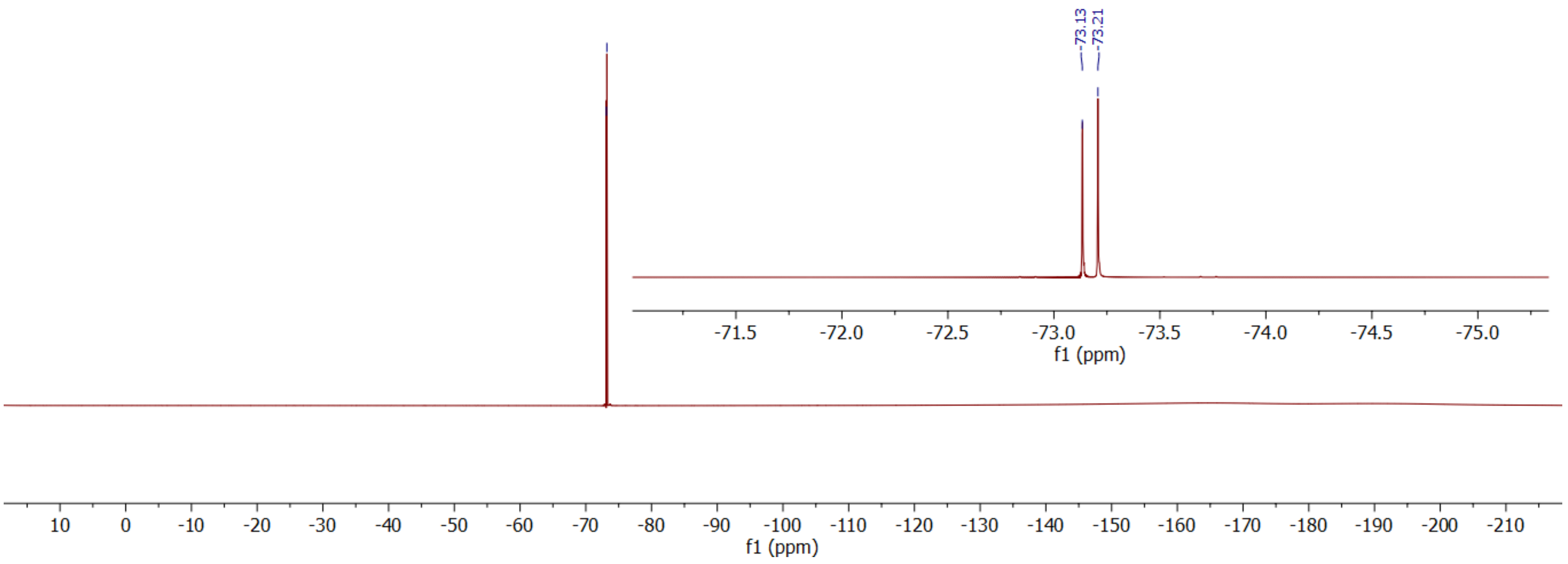

13C (100.63 MHz, CDCl3)

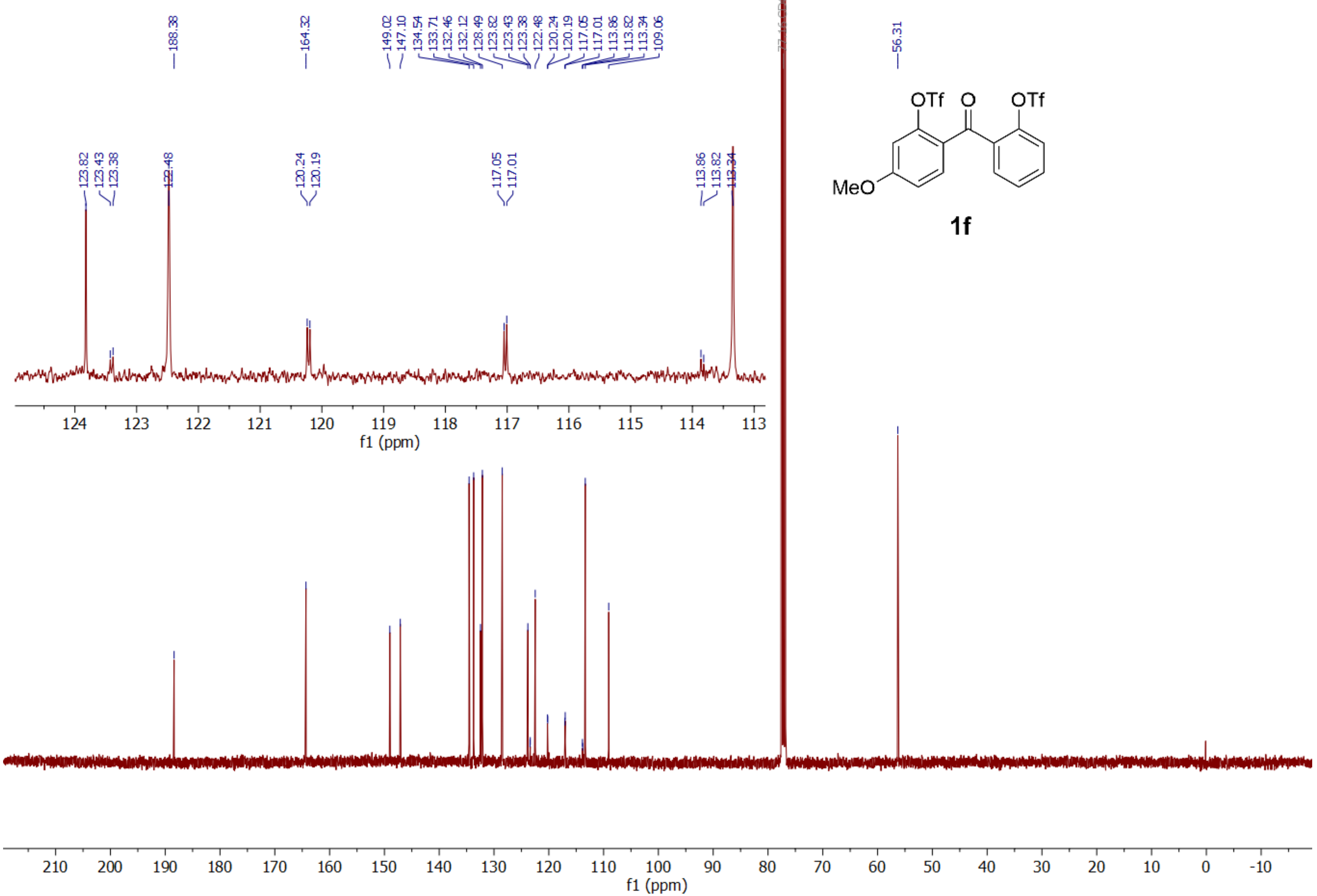

1H (400.15 MHz, CDCl3)

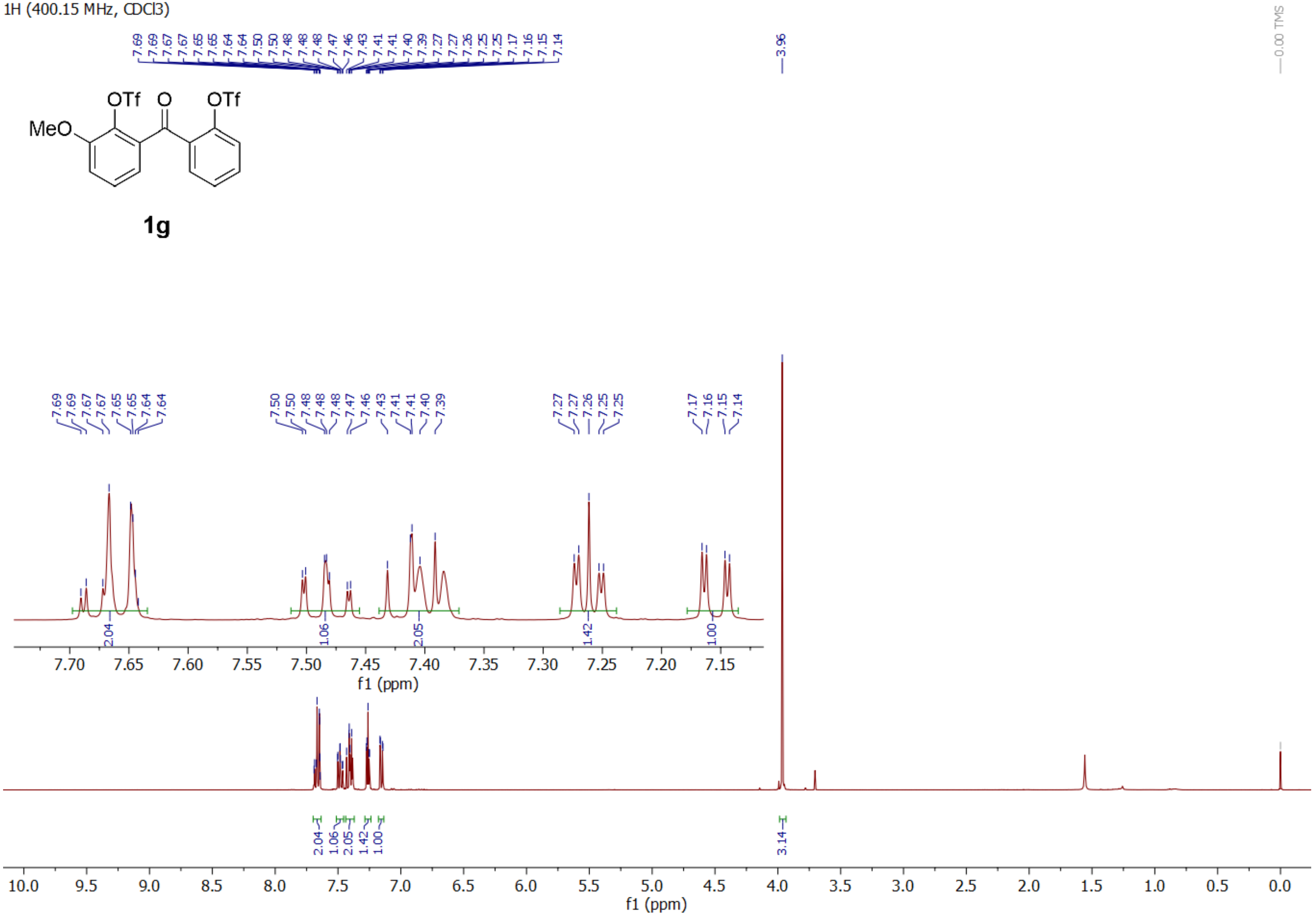


**1g**

19F (376.48 MHz, CDCl3)

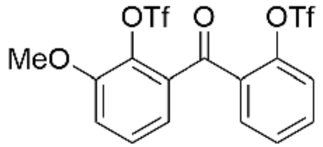


**1g**

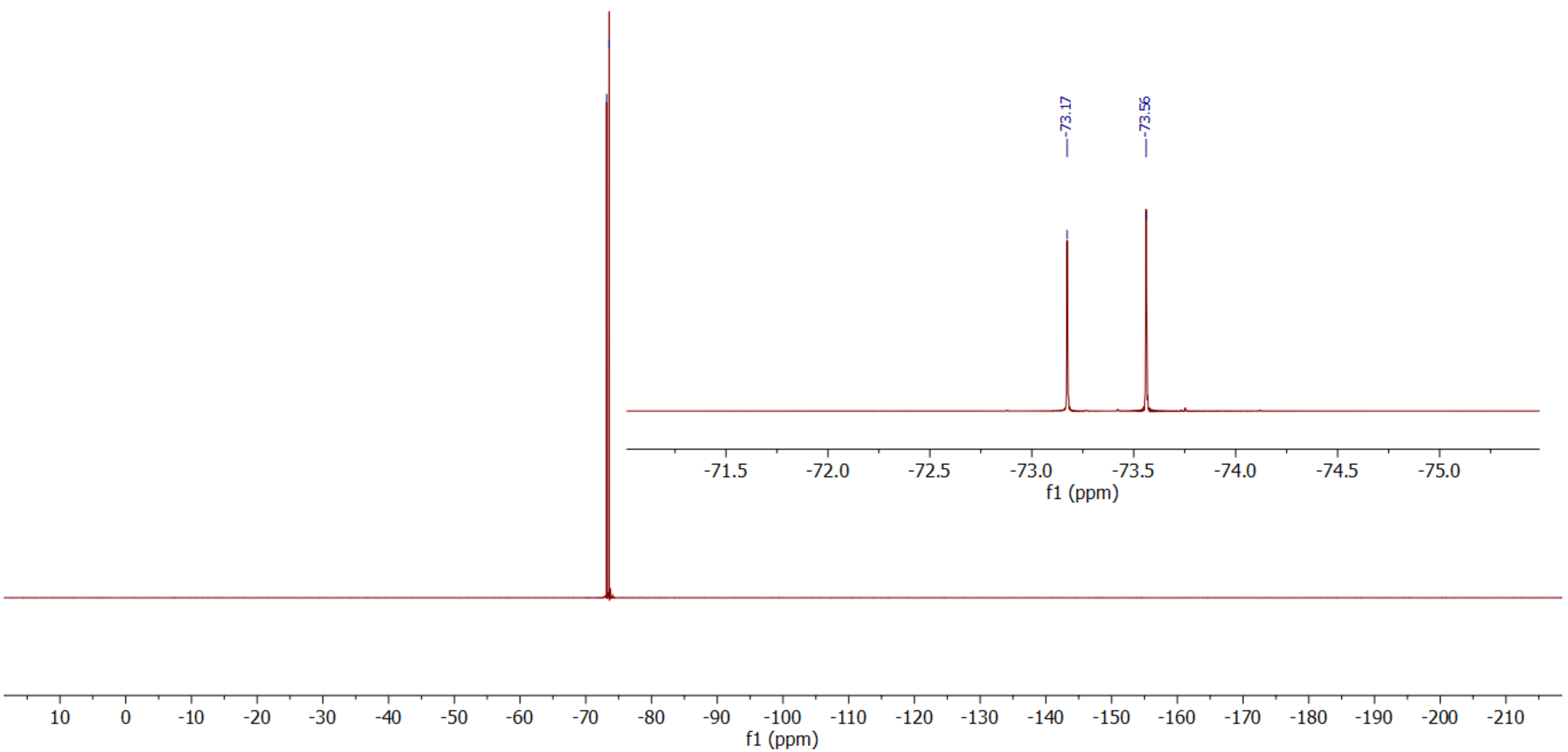

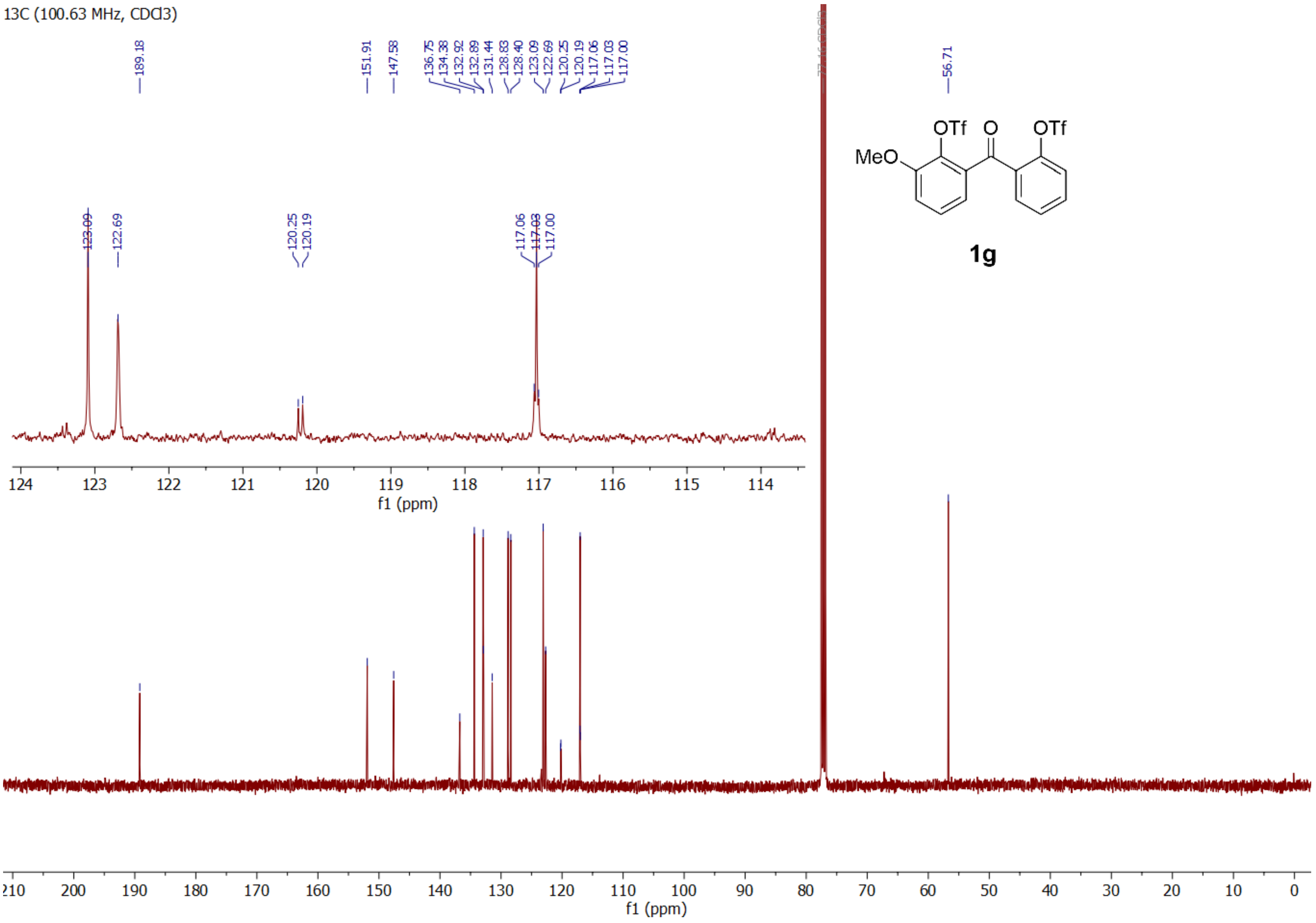

13C (100.63 MHz, CDCl3)
189.18
151.91
147.58
136.75
134.38
132.92
132.89
131.44
128.83
128.40
123.09
122.69
120.25
120.19
117.06
117.03
117.00
56.71
123.09
122.69
120.25
120.19
117.06
117.03
117.00
f1 (ppm)
f1 (ppm)
MeO
OTf
O
OTf
1g

1H (400.15 MHz, CDCl3)

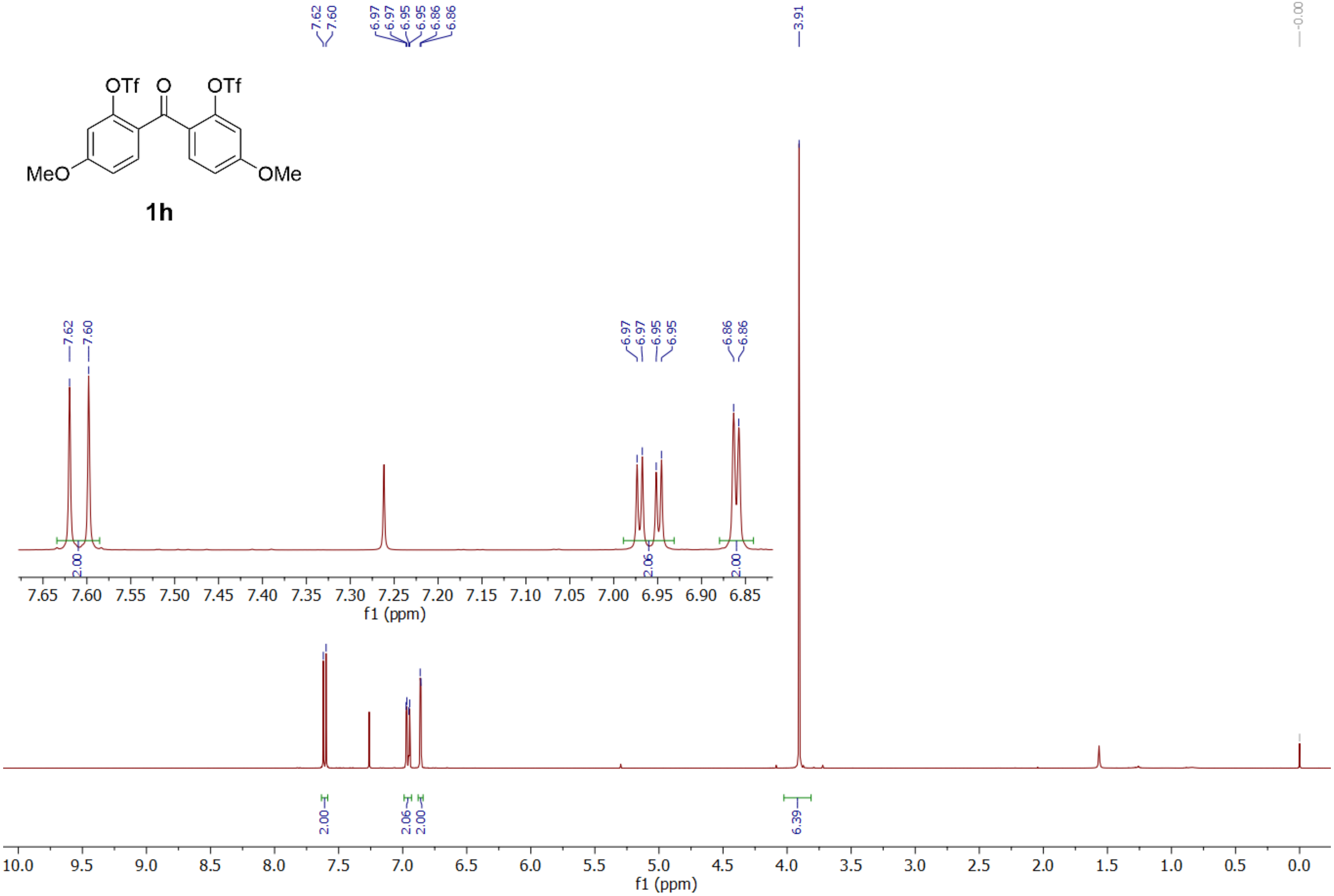

19F (376.48 MHz, CDCl3)

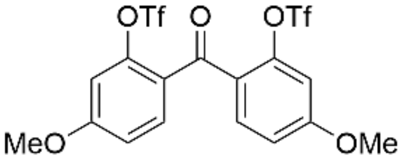


**1h**

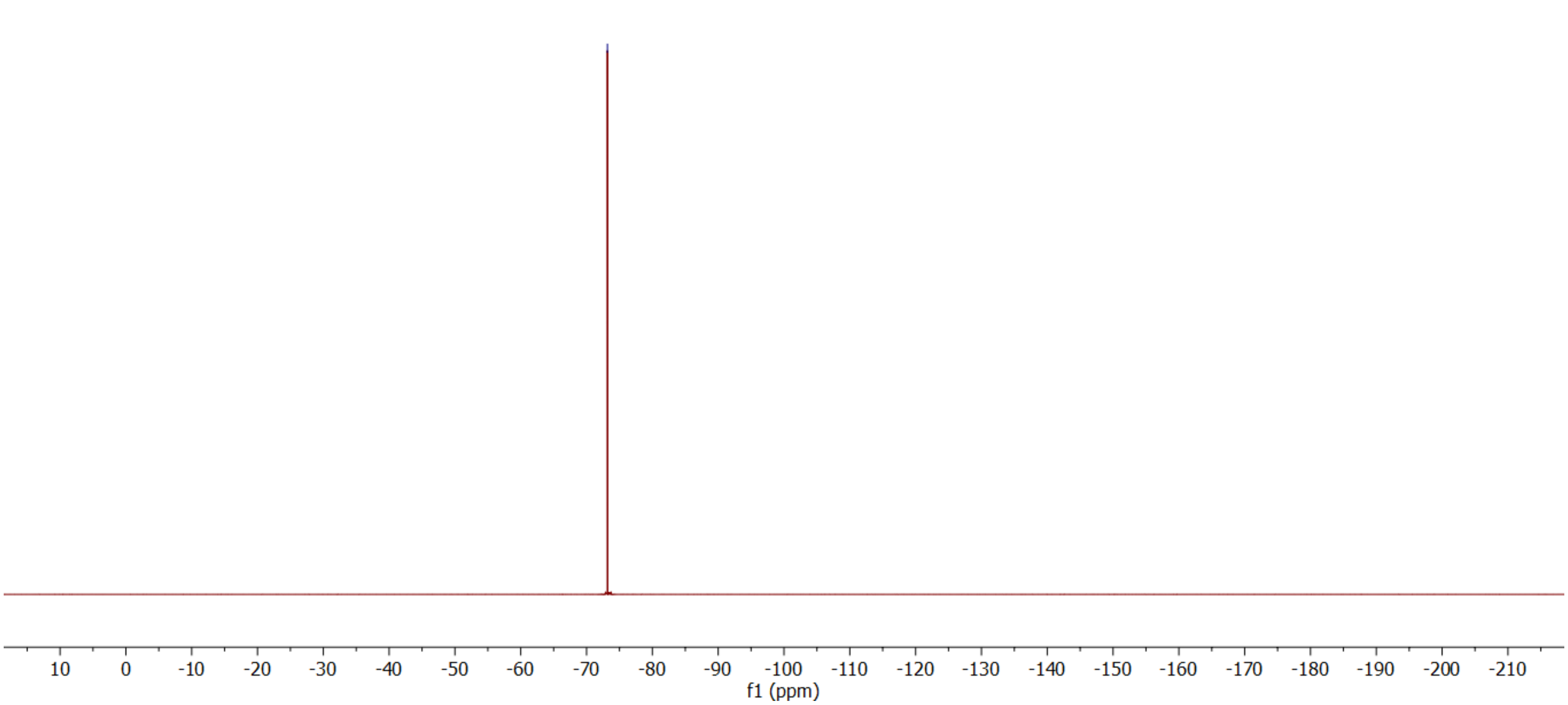

13C (100.63 MHz, CDCl3)

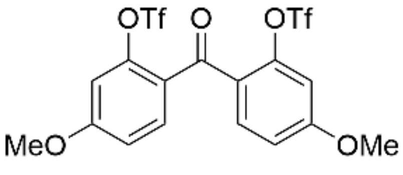

**1h**

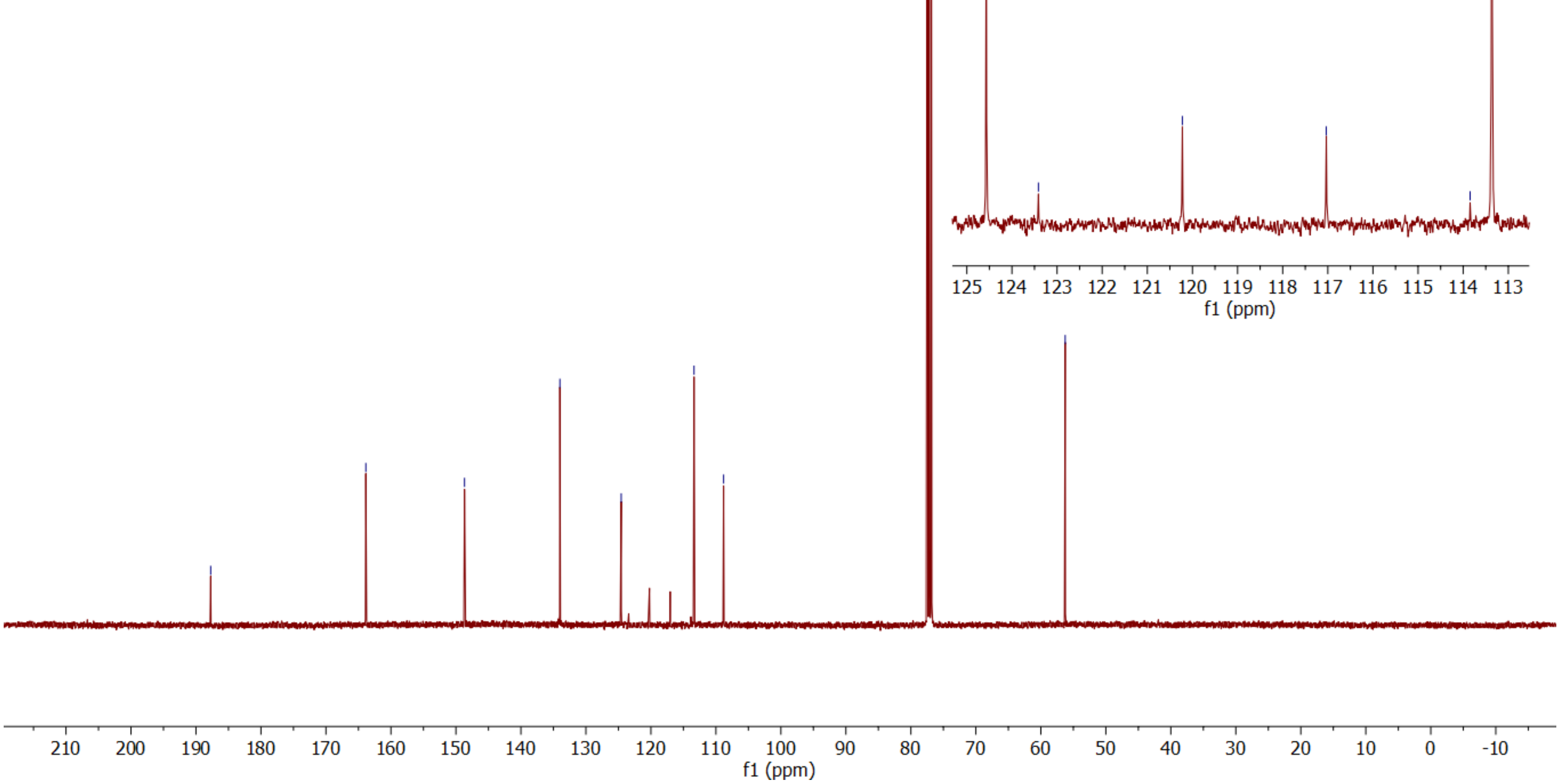

1H (400.15 MHz, CDCl3)

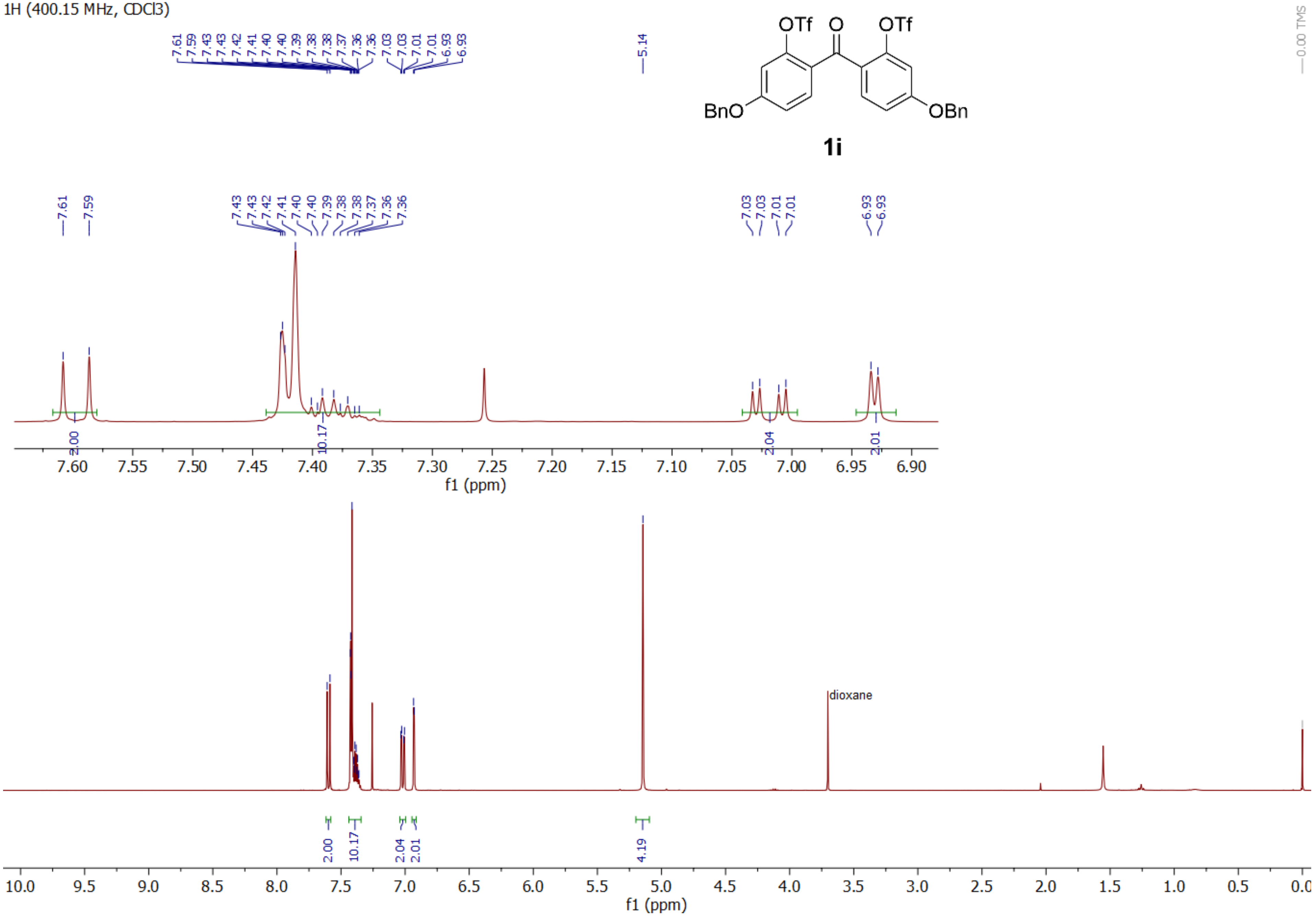

19F (376.48 MHz, CDCl3)

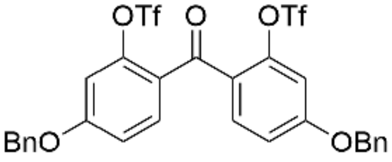

**1i**

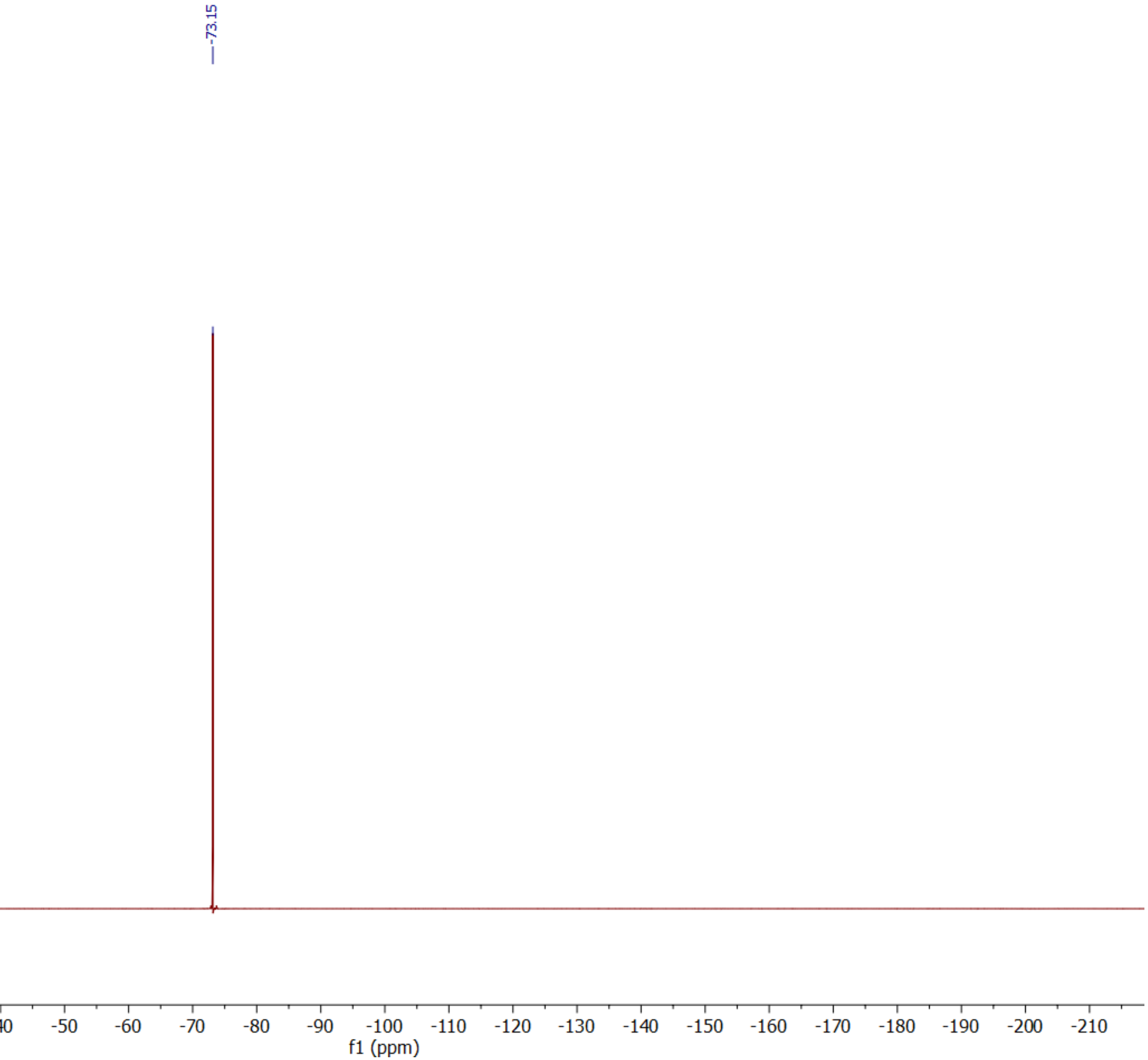

13C (100.63 MHz, CDCl3)

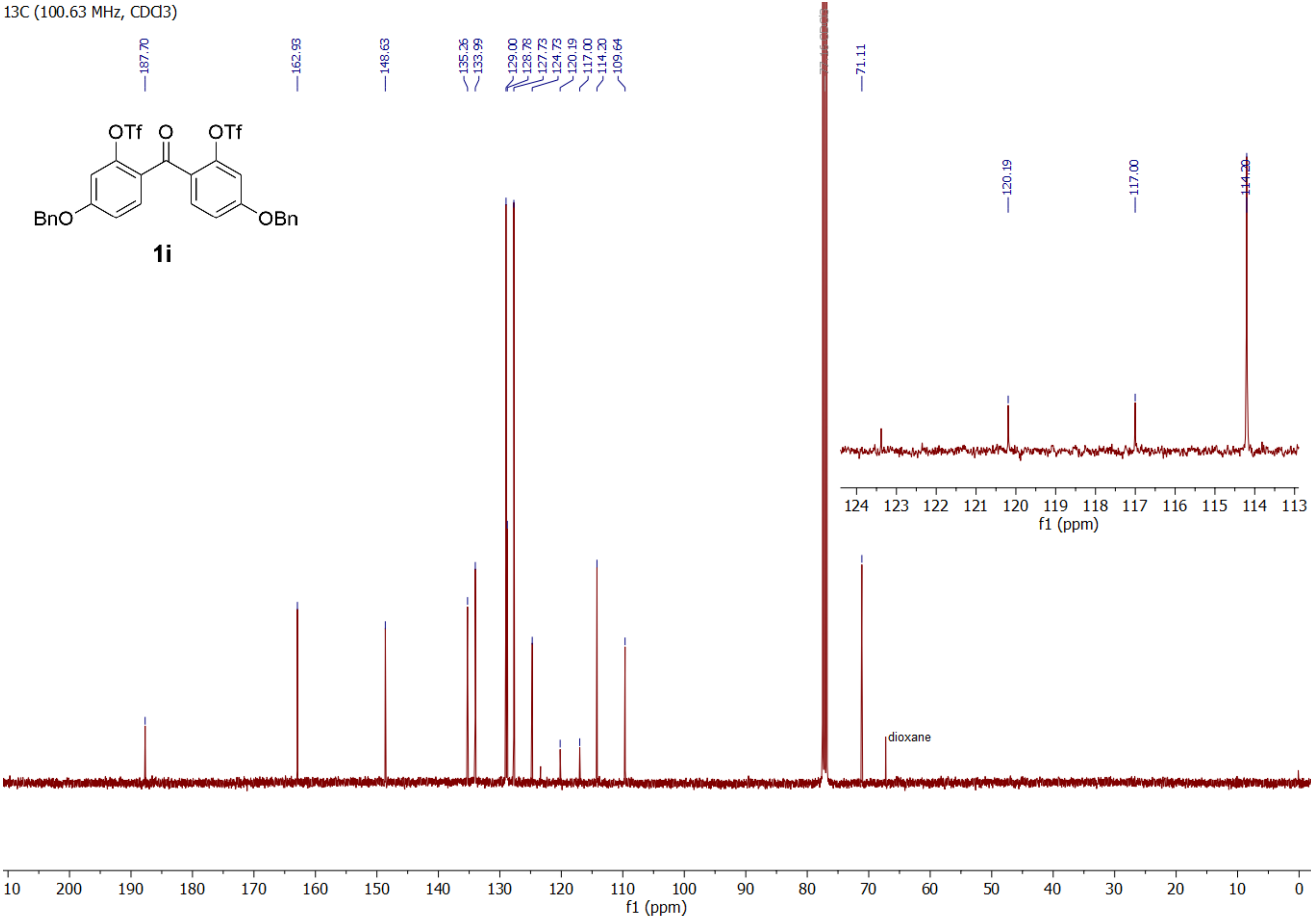

1H (400.15 MHz, CDCl3)

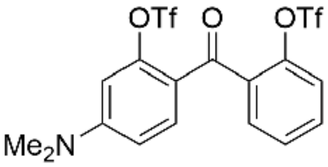


**1j**

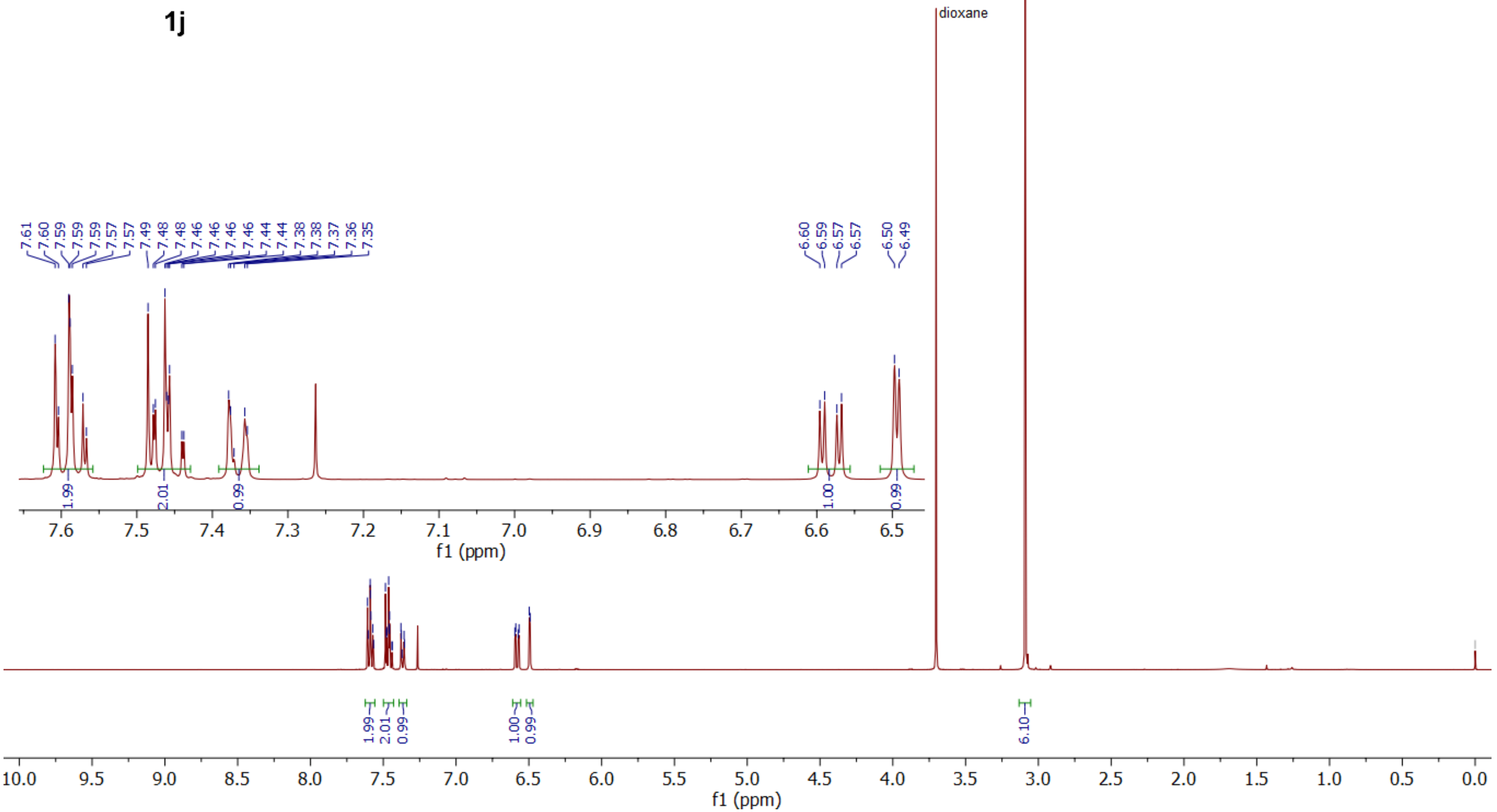

19F (376.48 MHz, CDCl3)

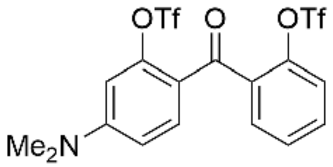

**1j**

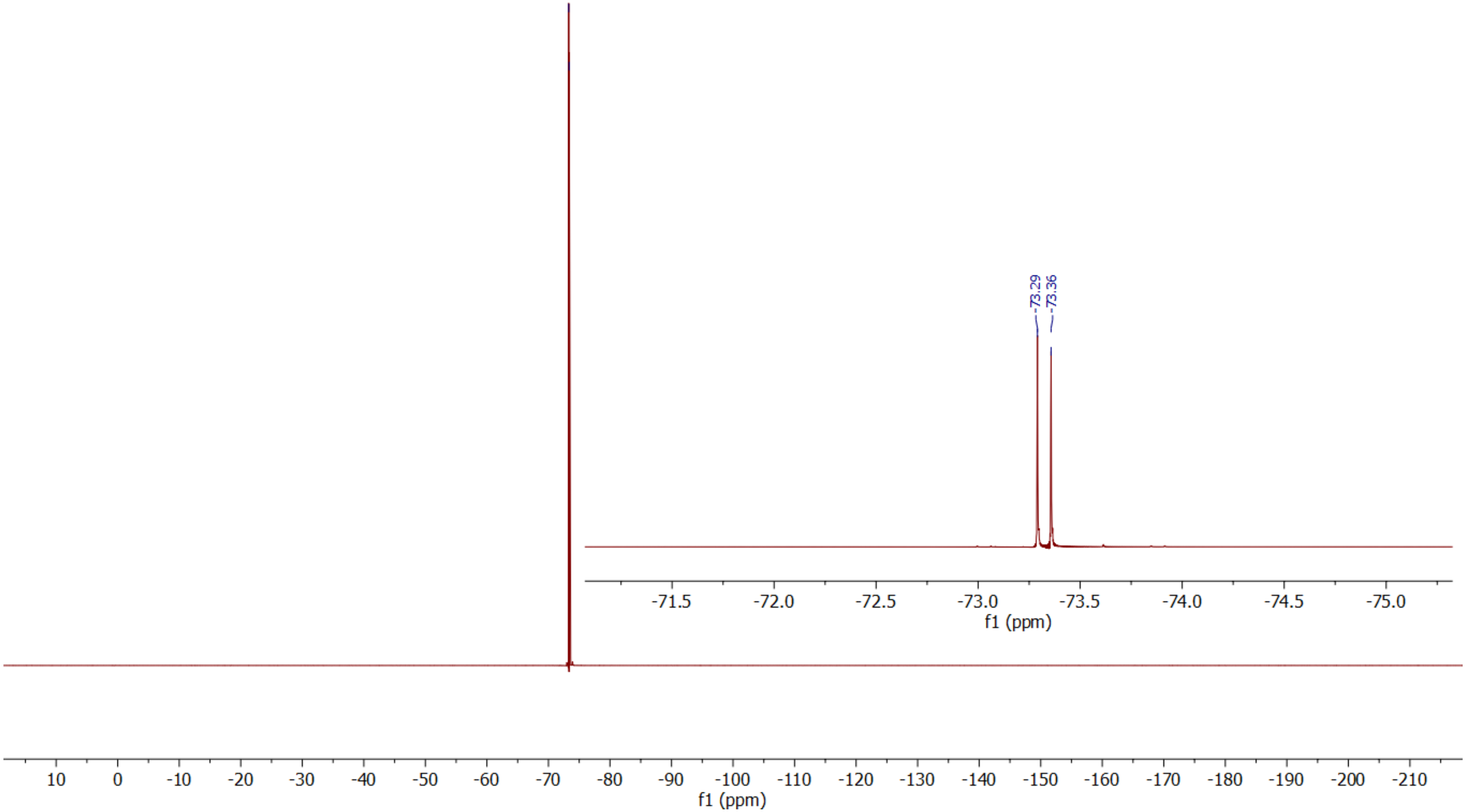

13C (100.63 MHz, CDCl3)

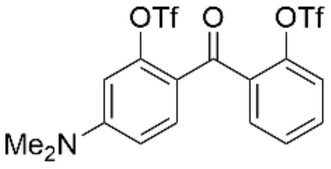


**1j**

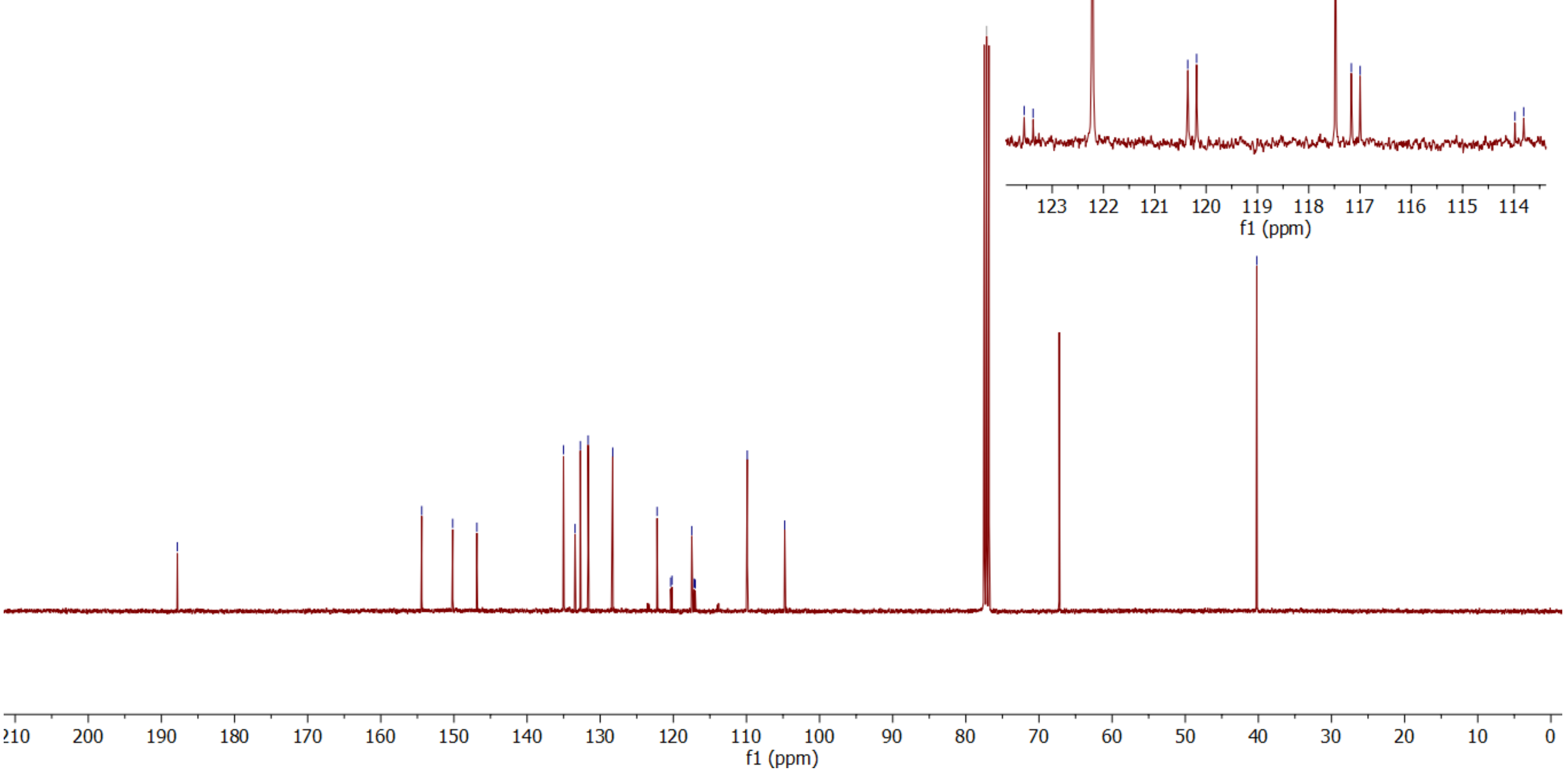

1H (400.15 MHz, CDCl3)

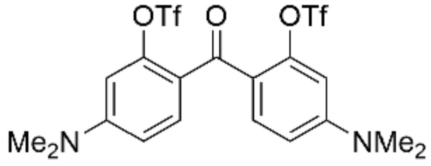


**1k**

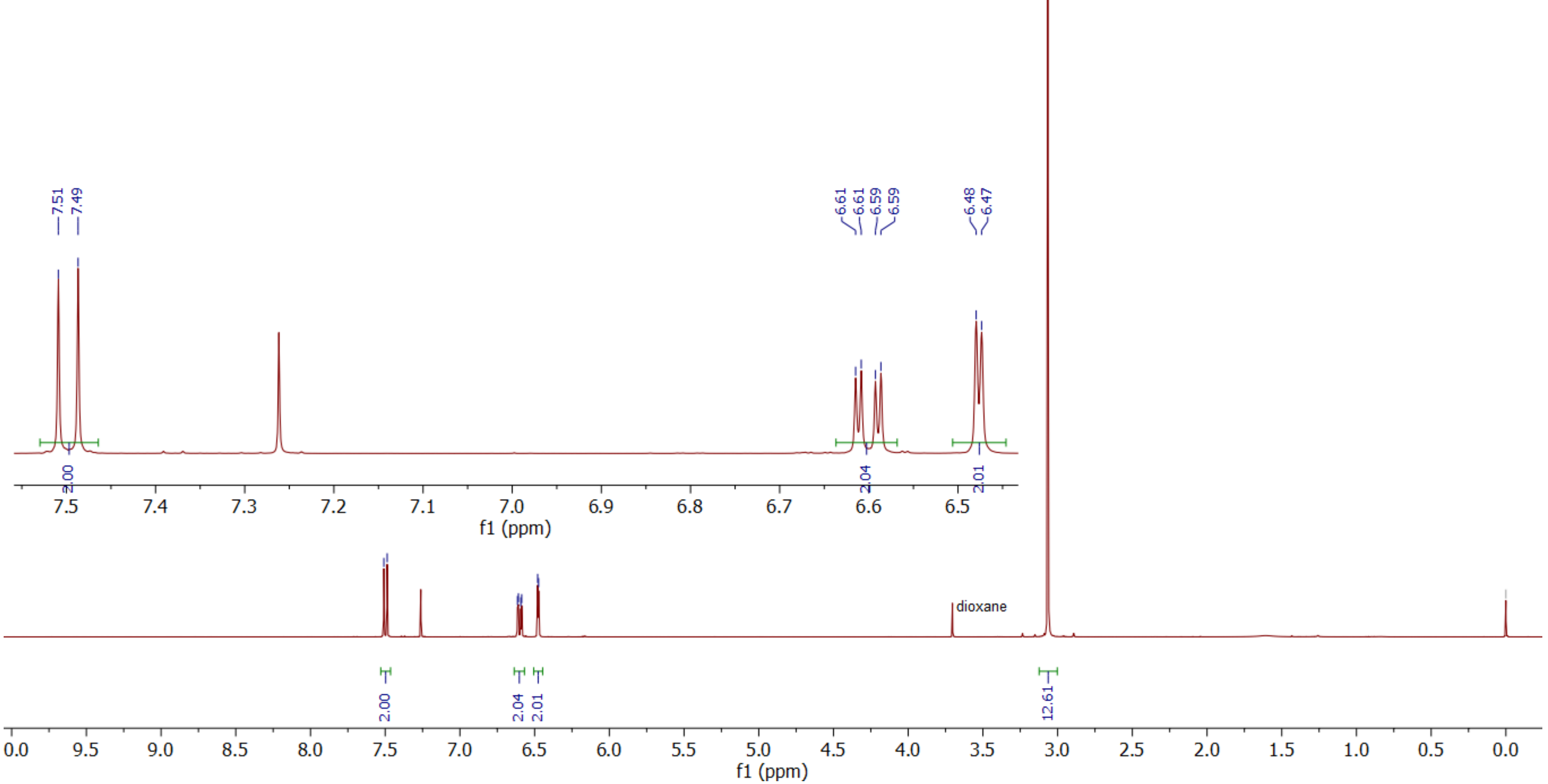

19F (376.48 MHz, CDCl3)

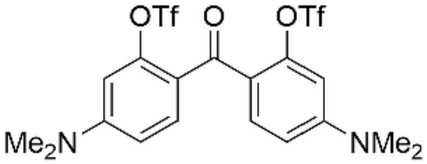


**1k**

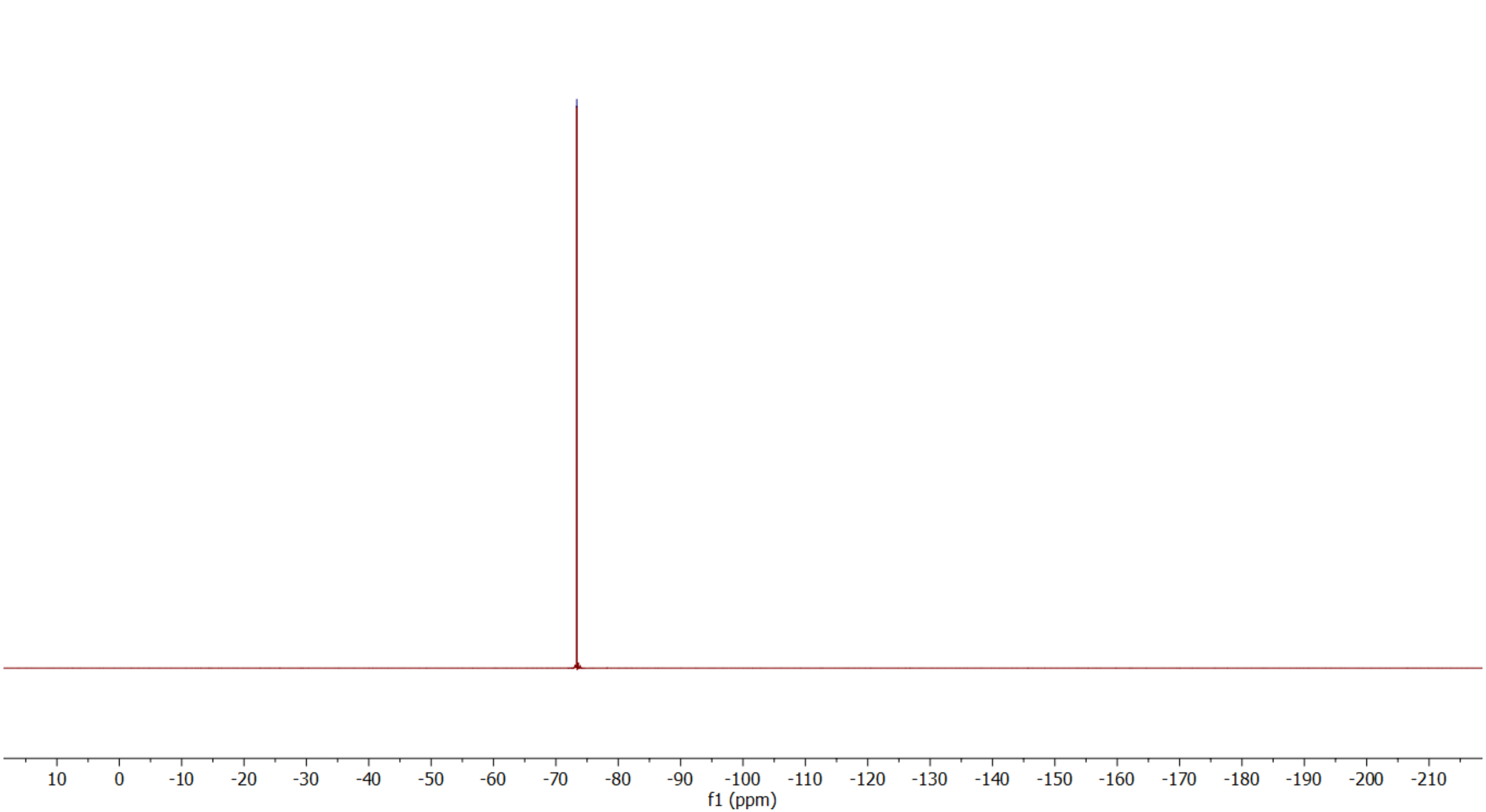

13C (100.63 MHz, CDCl3)

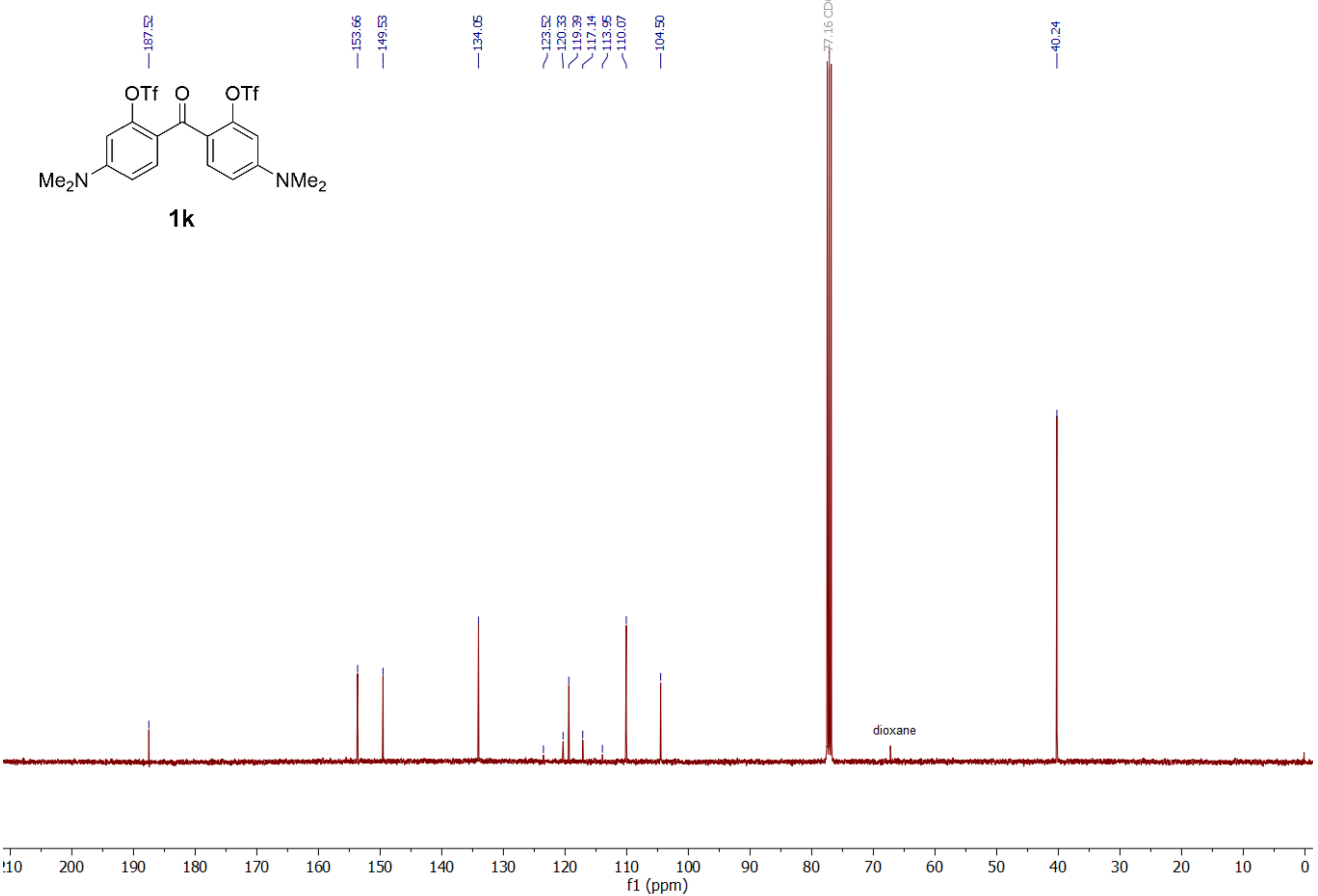

1H (400.15 MHz, CDCl3)

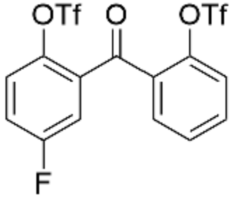

**1l**

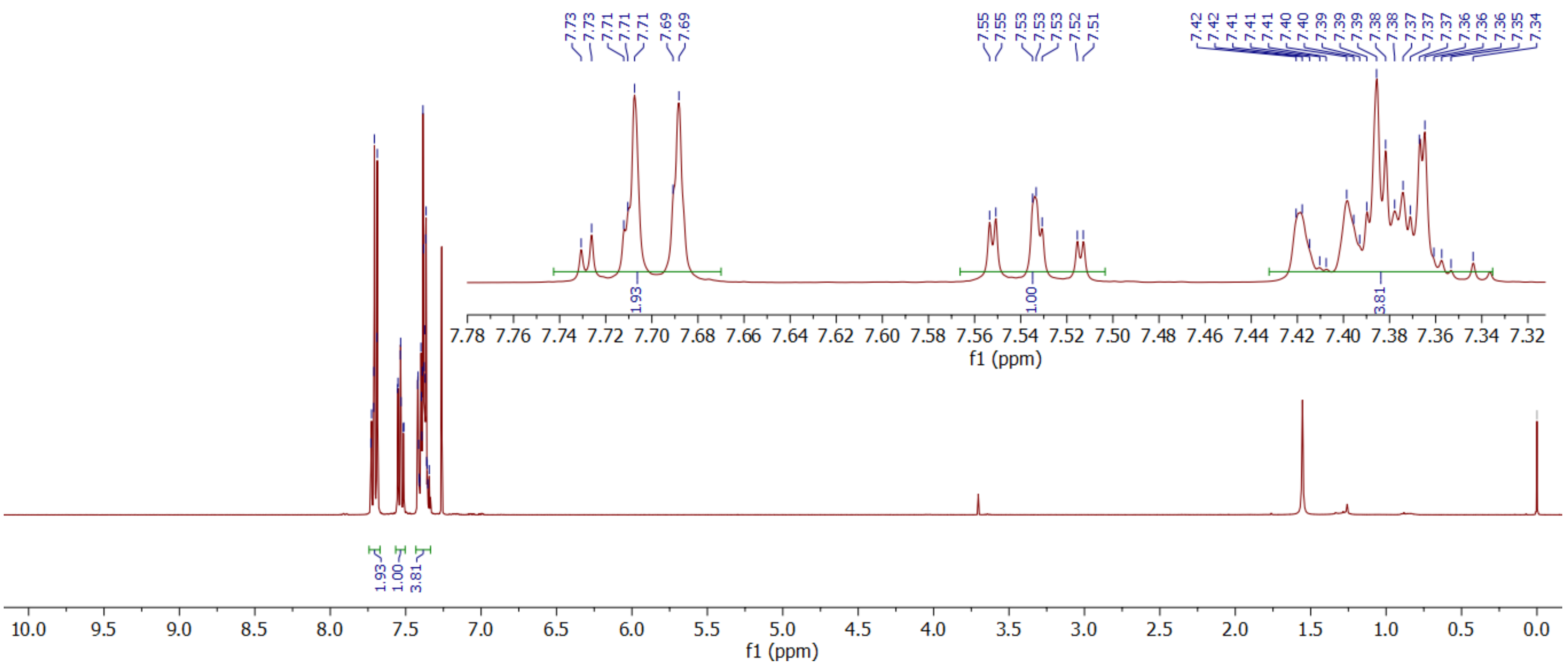

19F (376.48 MHz, CDCl3)

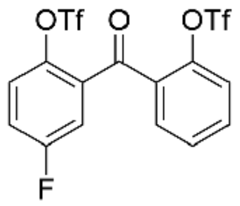

**1l**

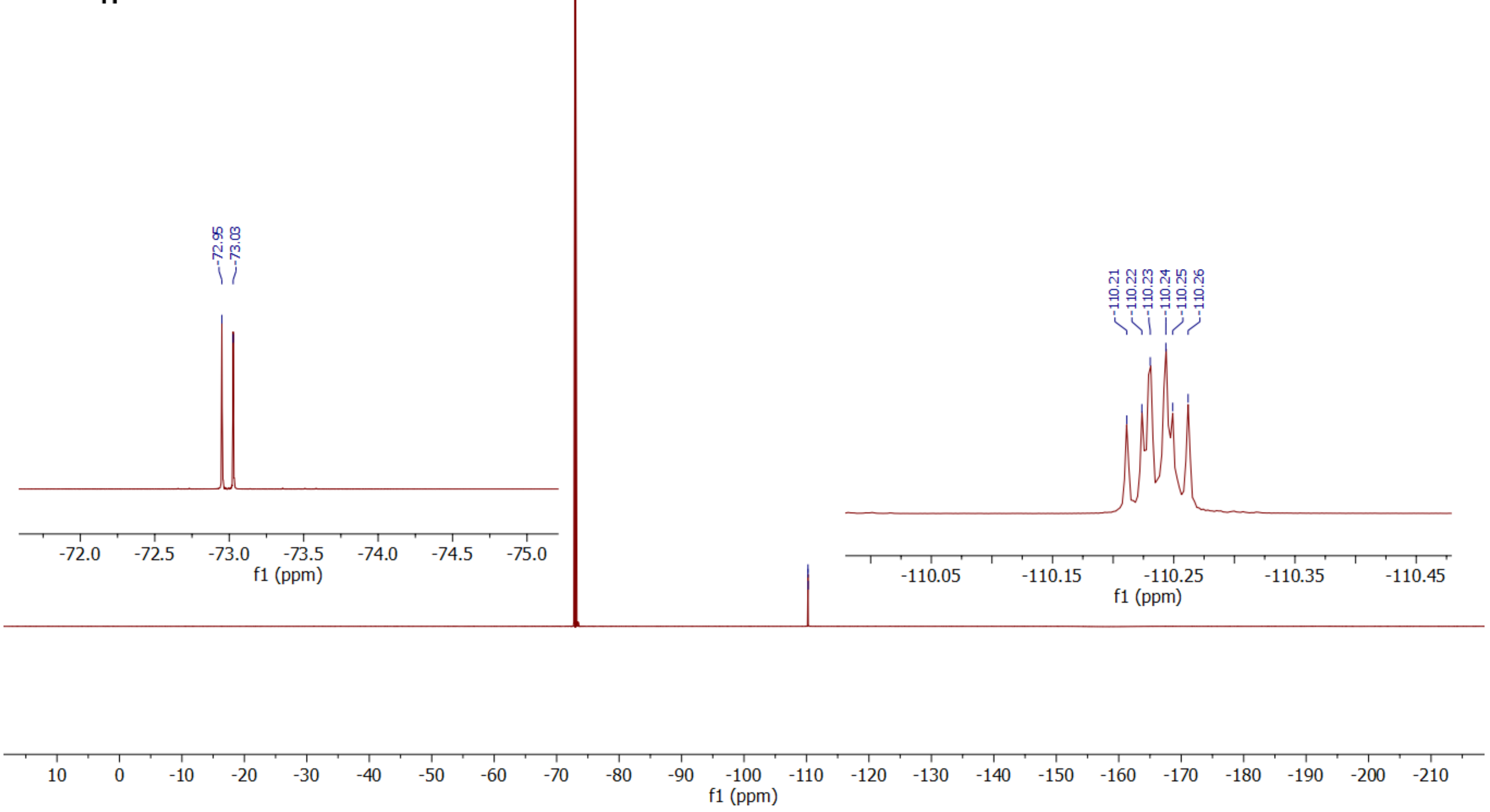

13C (100.63 MHz, CDCl3)

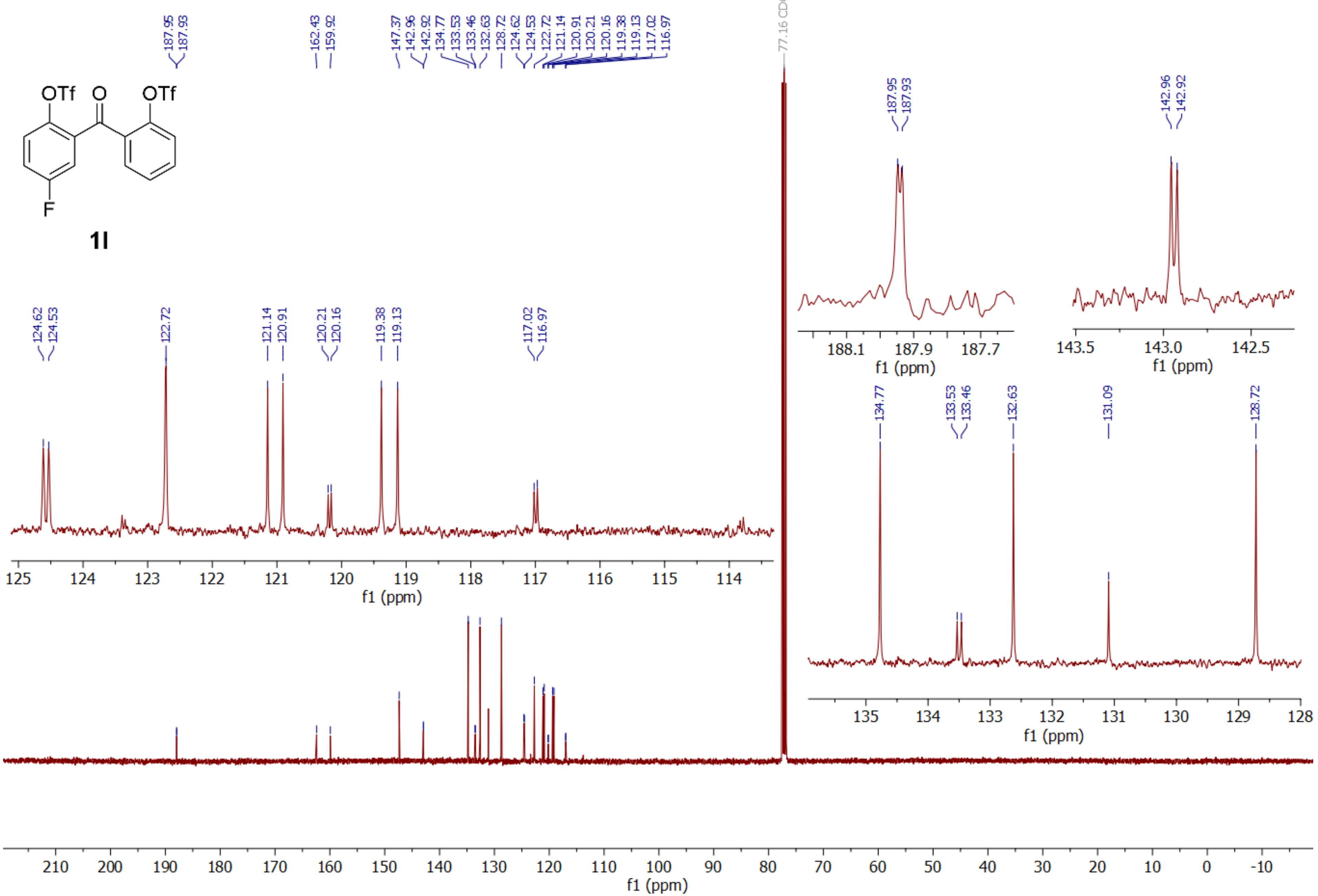

1H (400.15 MHz, CDCl3)

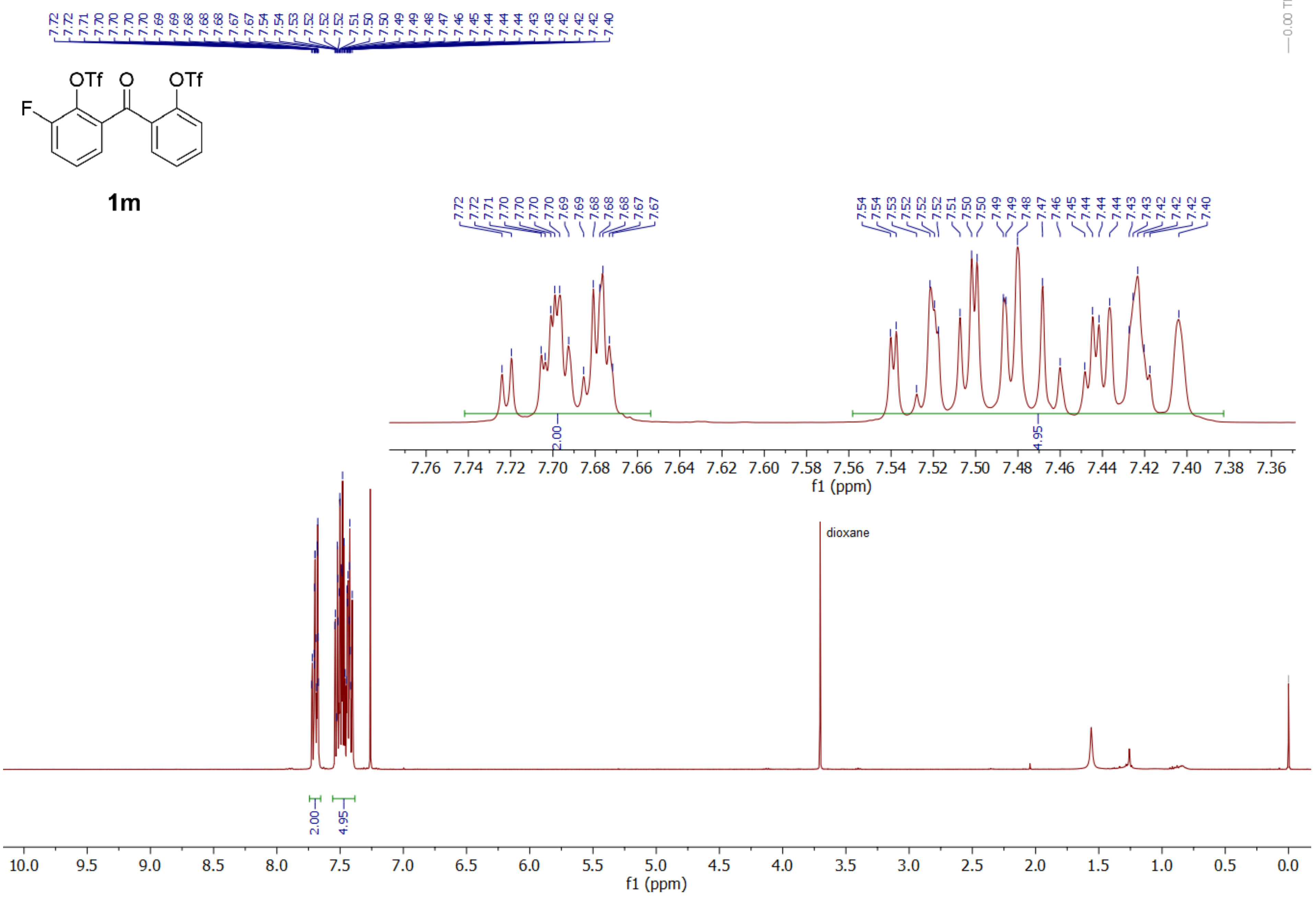

19F (376.48 MHz, CDCl3)

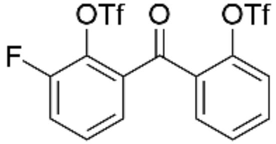

**1m**

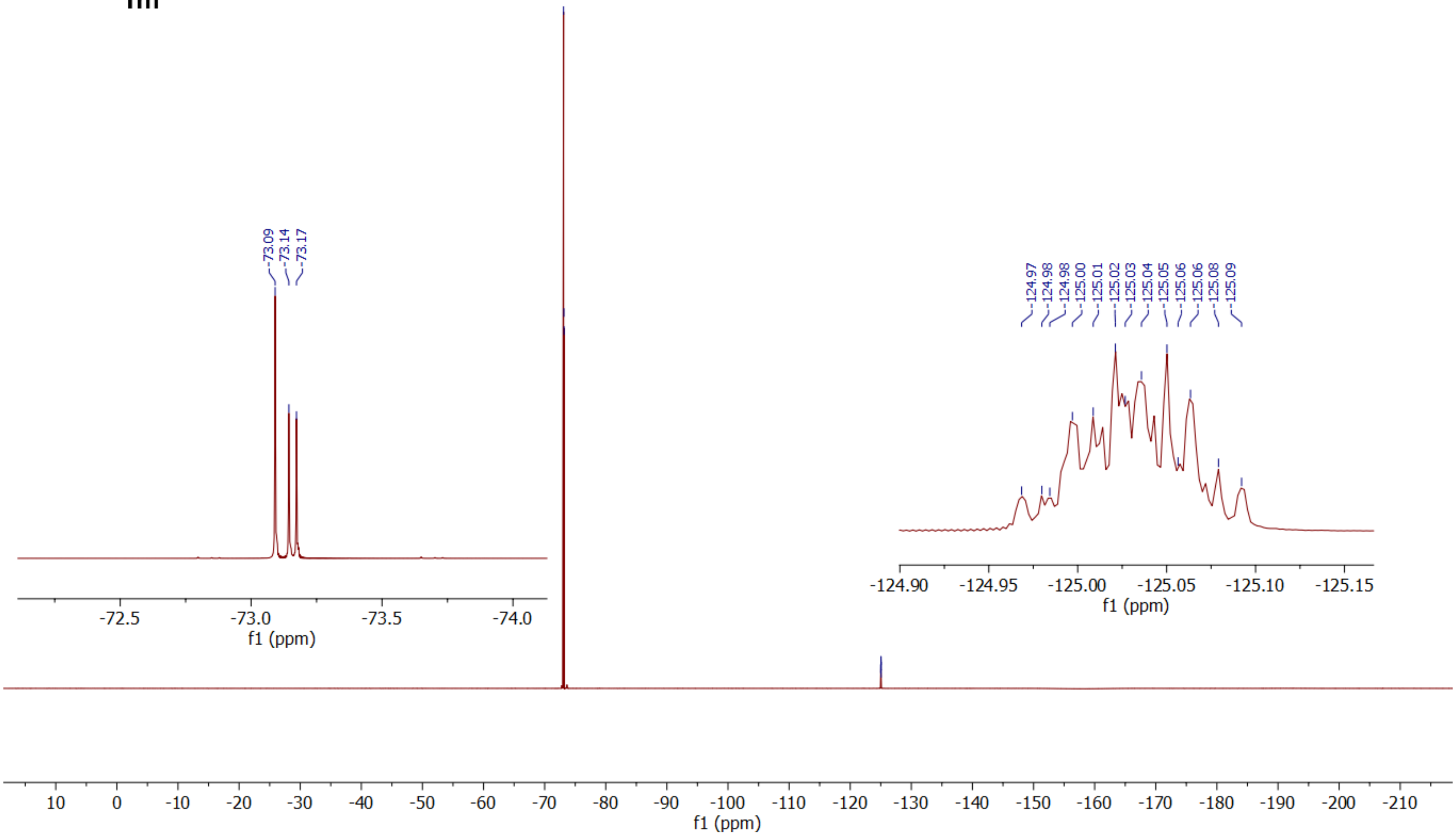

13C (100.63 MHz, CDCl3)

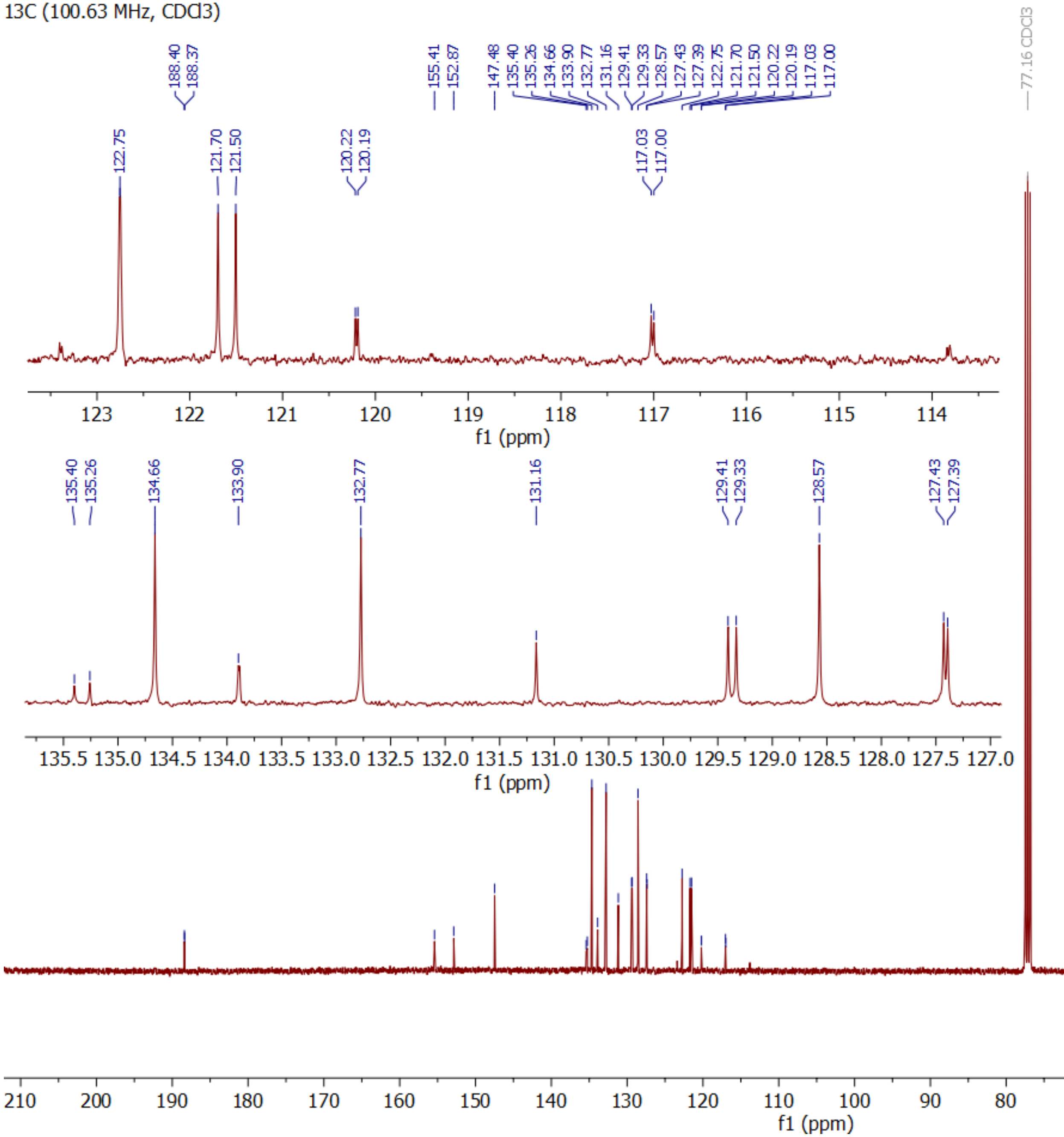


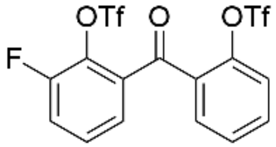


**1m**

1H (400.15 MHz, CDCl3)

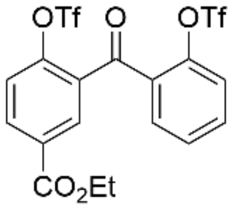

**1n**

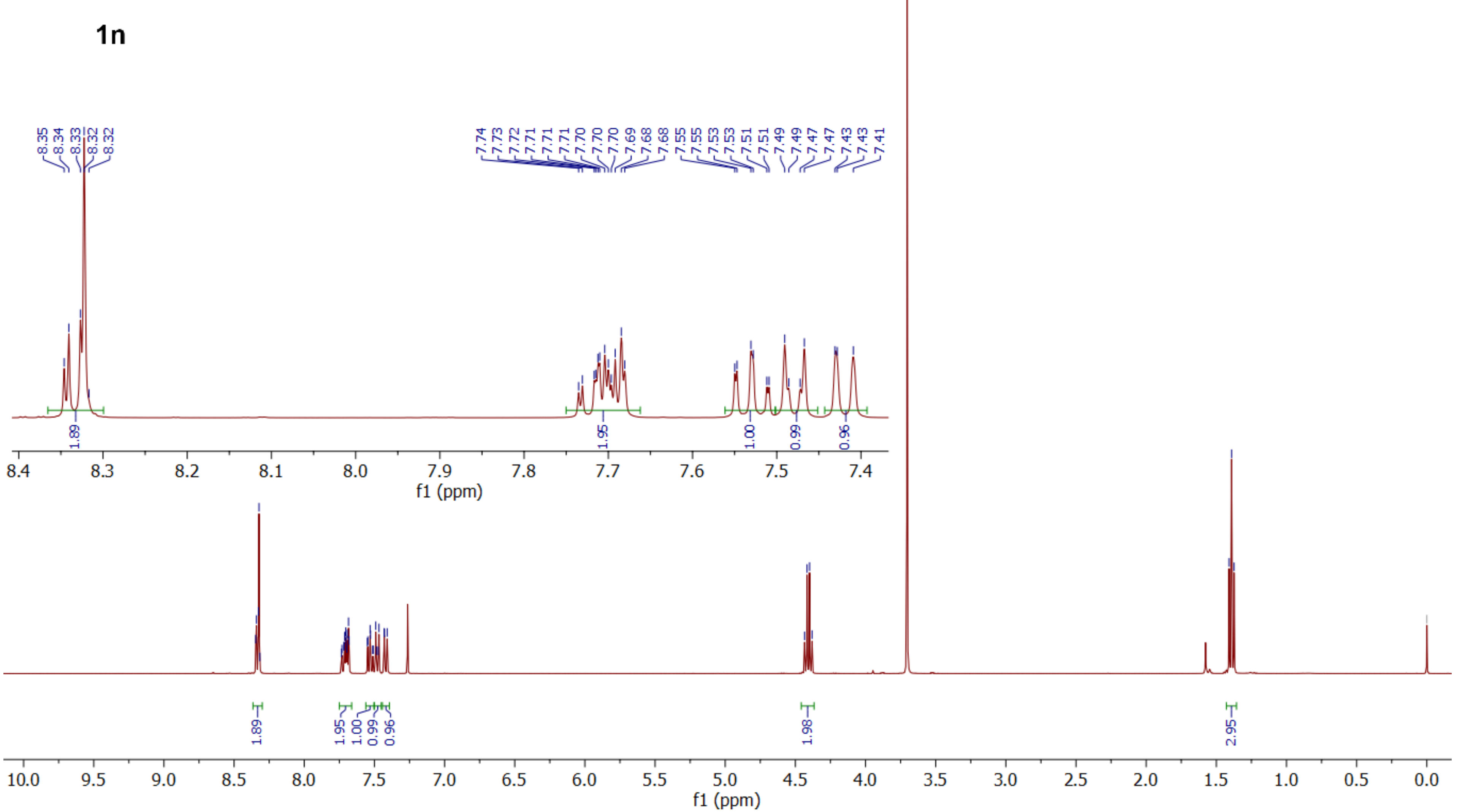

19F (376.48 MHz, CDCl3)

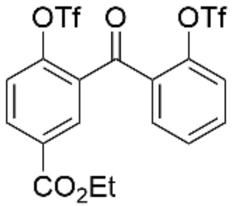

**1n**

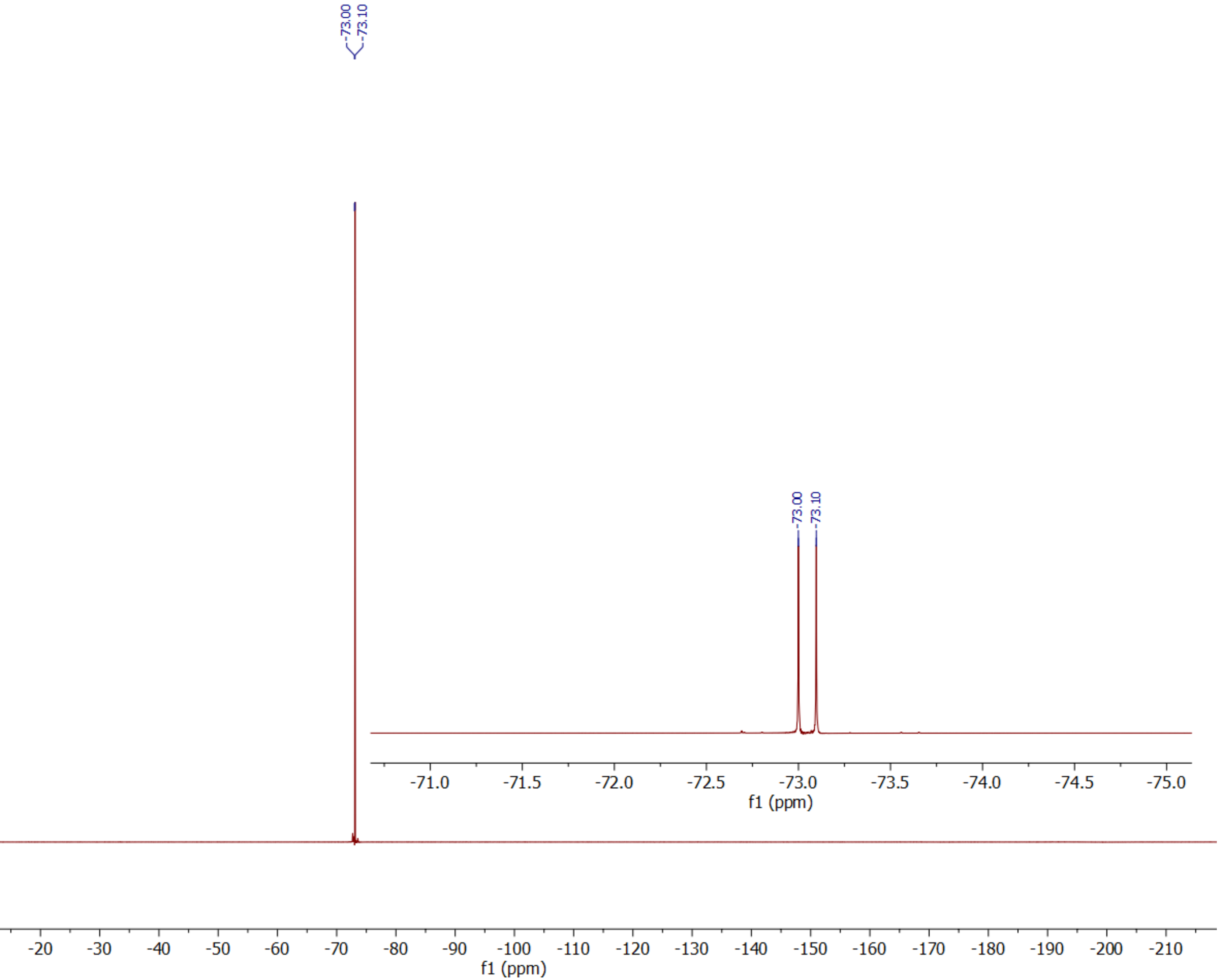

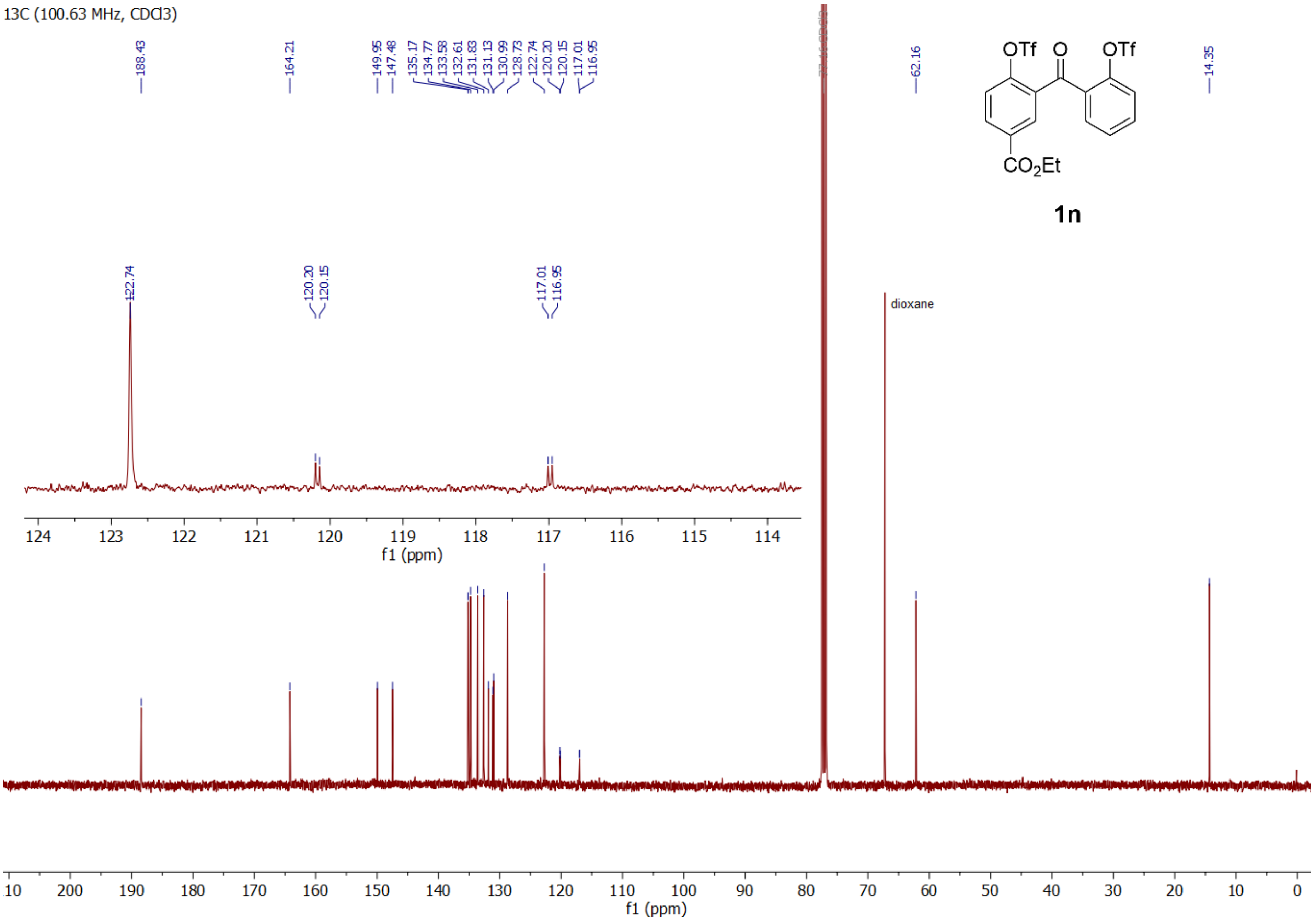

13C (100.63 MHz, CDCl3)
188.43
164.21
149.95
147.48
135.17
134.77
133.58
132.61
131.83
131.13
130.99
128.73
122.74
120.20
120.15
117.01
116.95
62.16
14.35
OTf
O
OTf
CO2Et
1n
dioxane
f1 (ppm)

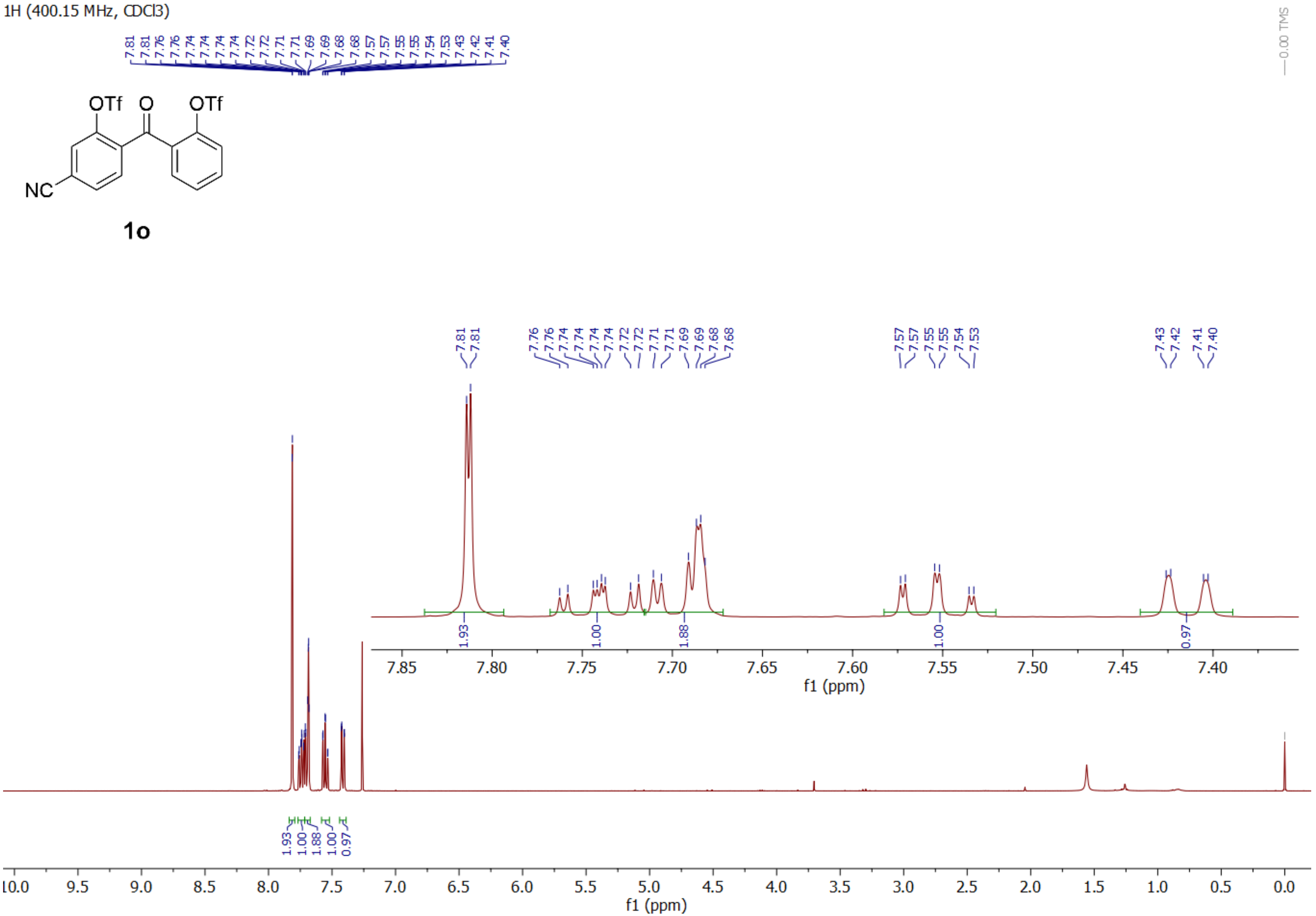
1H (400.15 MHz, CDCl3)
OTf O OTf
NC
1o
7.81 7.81 7.76 7.76 7.74 7.74 7.74 7.74 7.72 7.72 7.71 7.71 7.69 7.69 7.68 7.68 7.57 7.57 7.55 7.55 7.54 7.53 7.43 7.42 7.41 7.40
0.00 TMS
1.93 1.00 1.88 1.00 0.97
f1 (ppm)

19F (376.48 MHz, CDCl3)

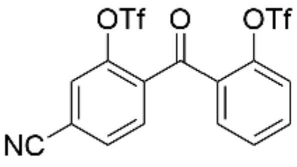


**1o**

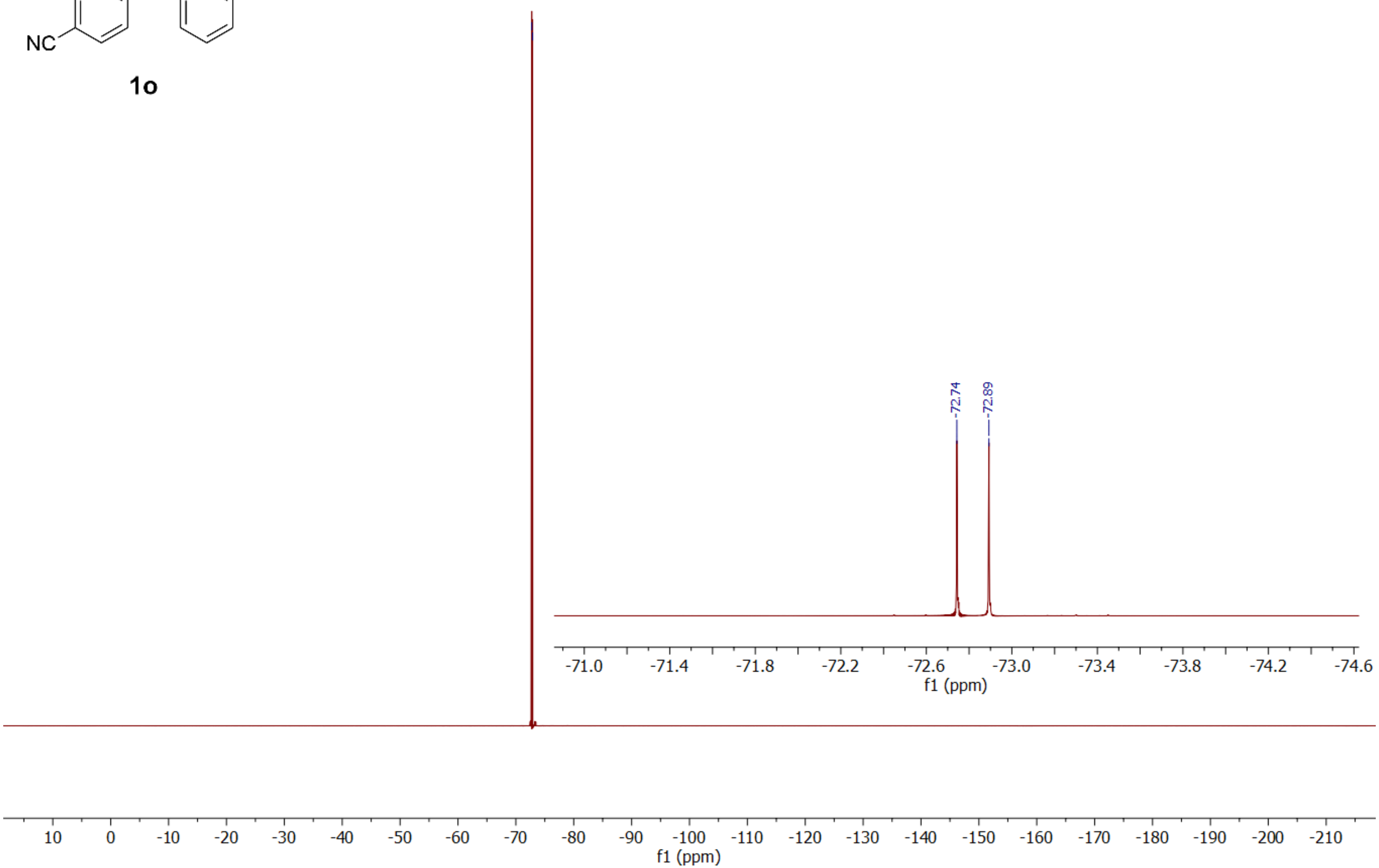

13C (100.63 MHz, CDCl3)

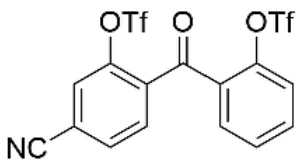


**1o**

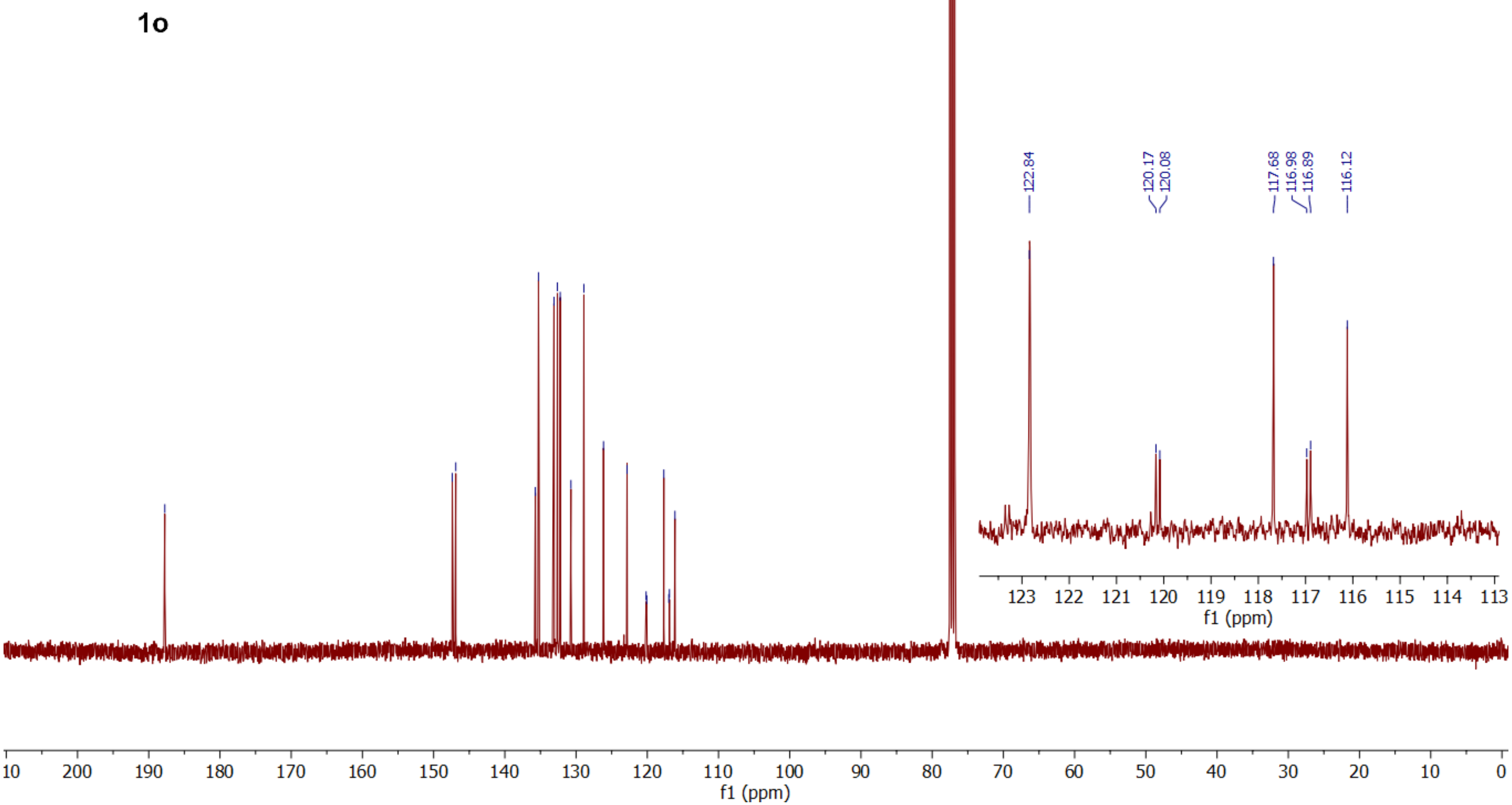

1H (400.15 MHz, CDCl3)

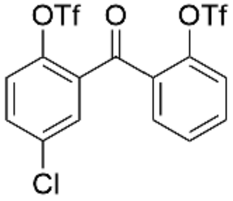


1r

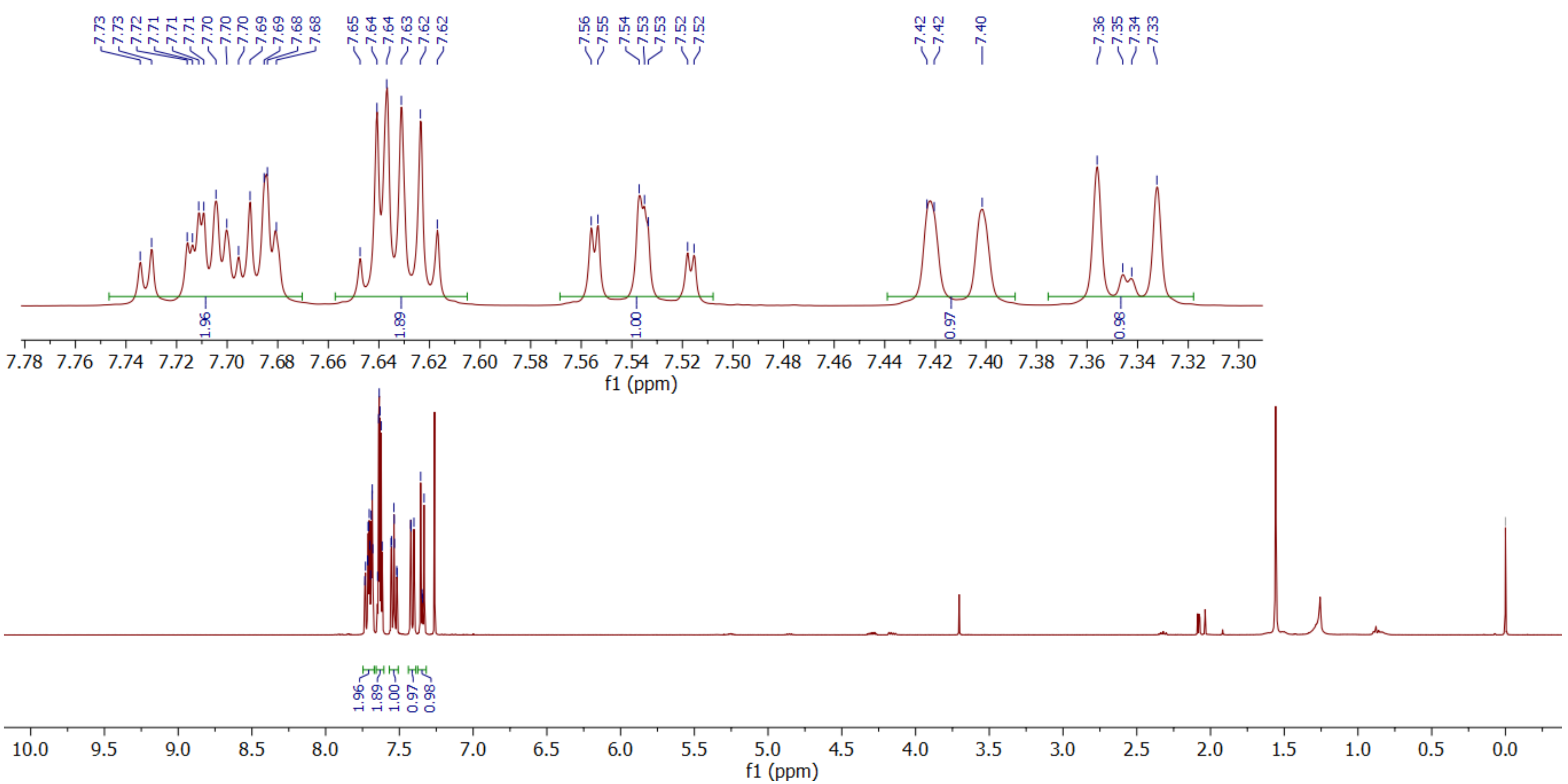

19F (376.48 MHz, CDCl3)

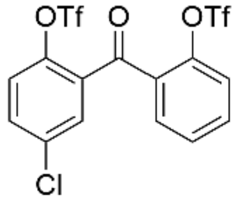


**1r**

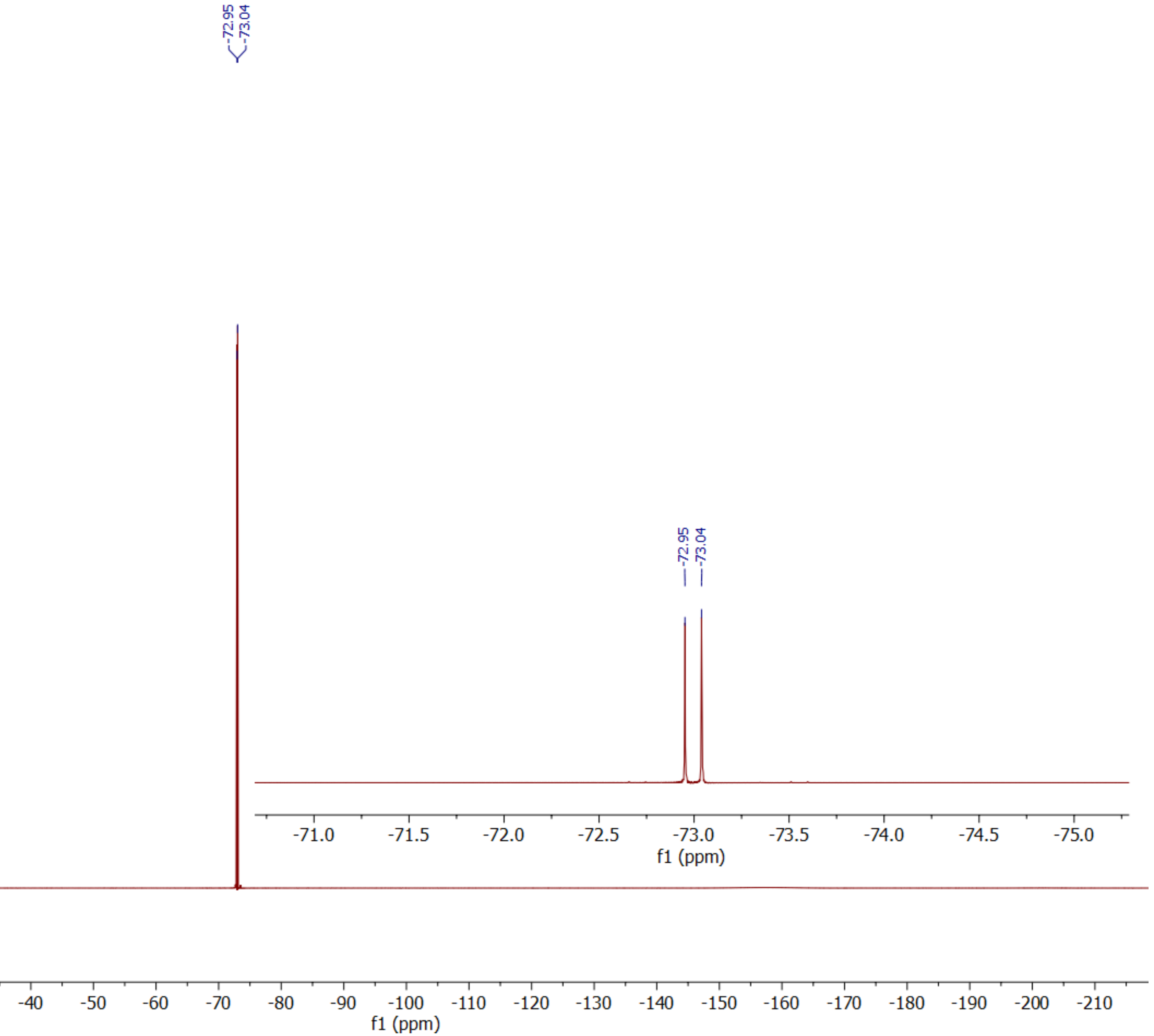

13C (100.63 MHz, CDCl3)

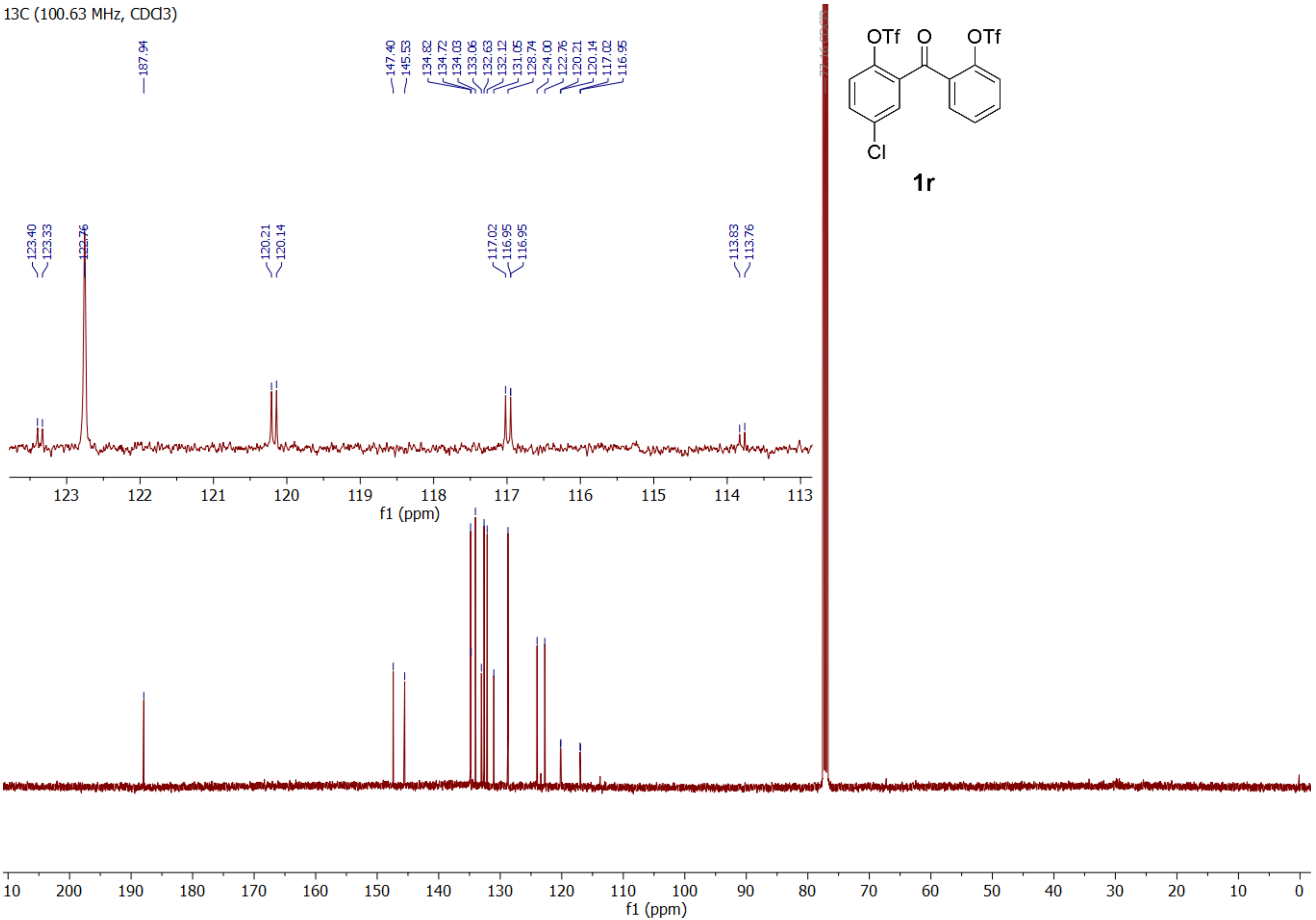


**1r**

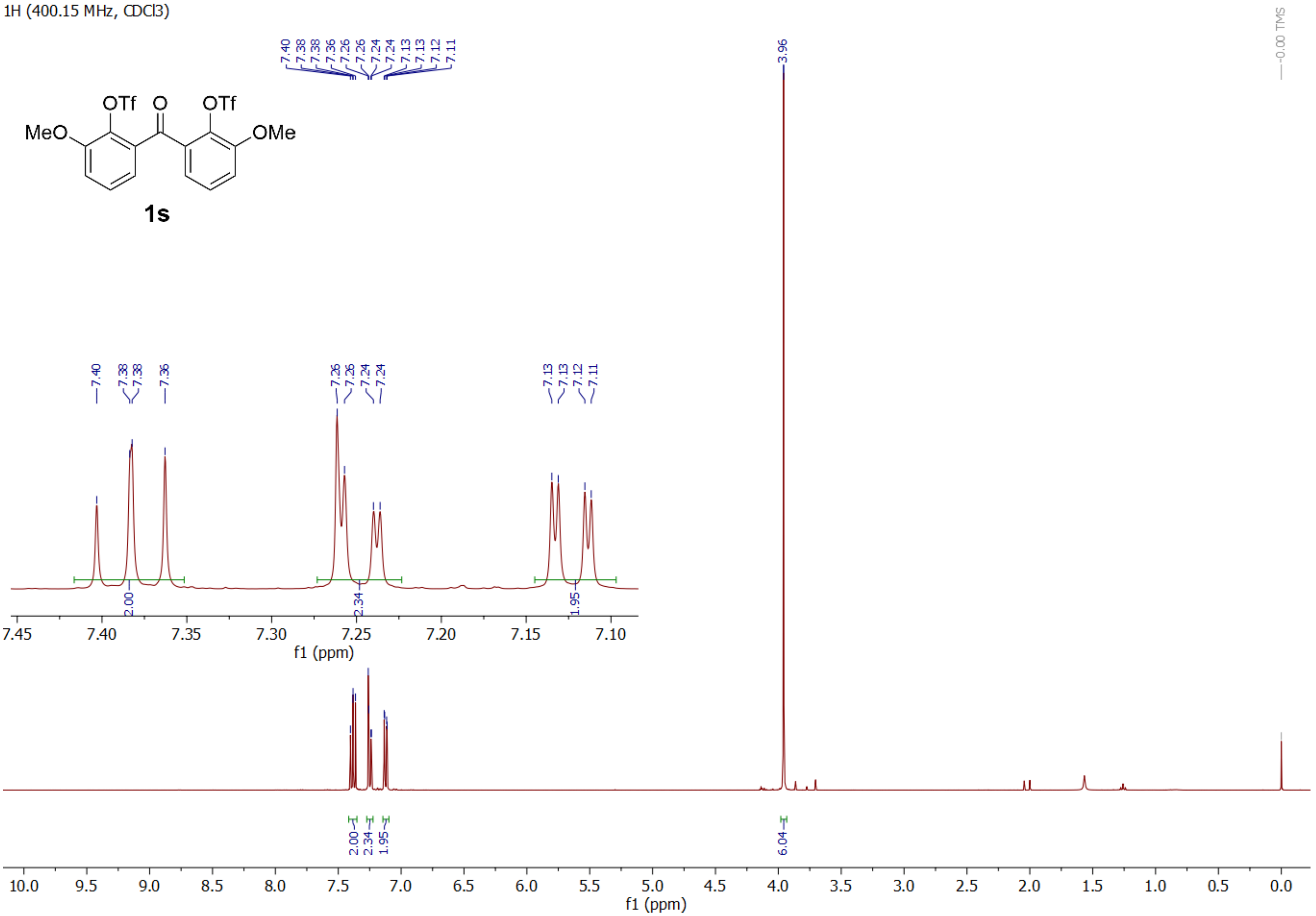
1H (400.15 MHz, CDCl3)
OTf O OTf
MeO OMe
1s
7.40 7.38 7.38 7.36 7.26 7.26 7.24 7.24 7.13 7.13 7.12 7.11
3.96
0.00 TMS
2.00 2.34 1.95
6.04
f1 (ppm)

19F (376.48 MHz, CDCl3)

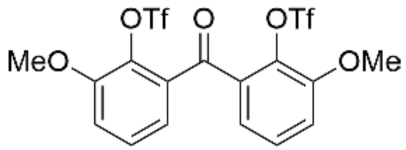

**1s**

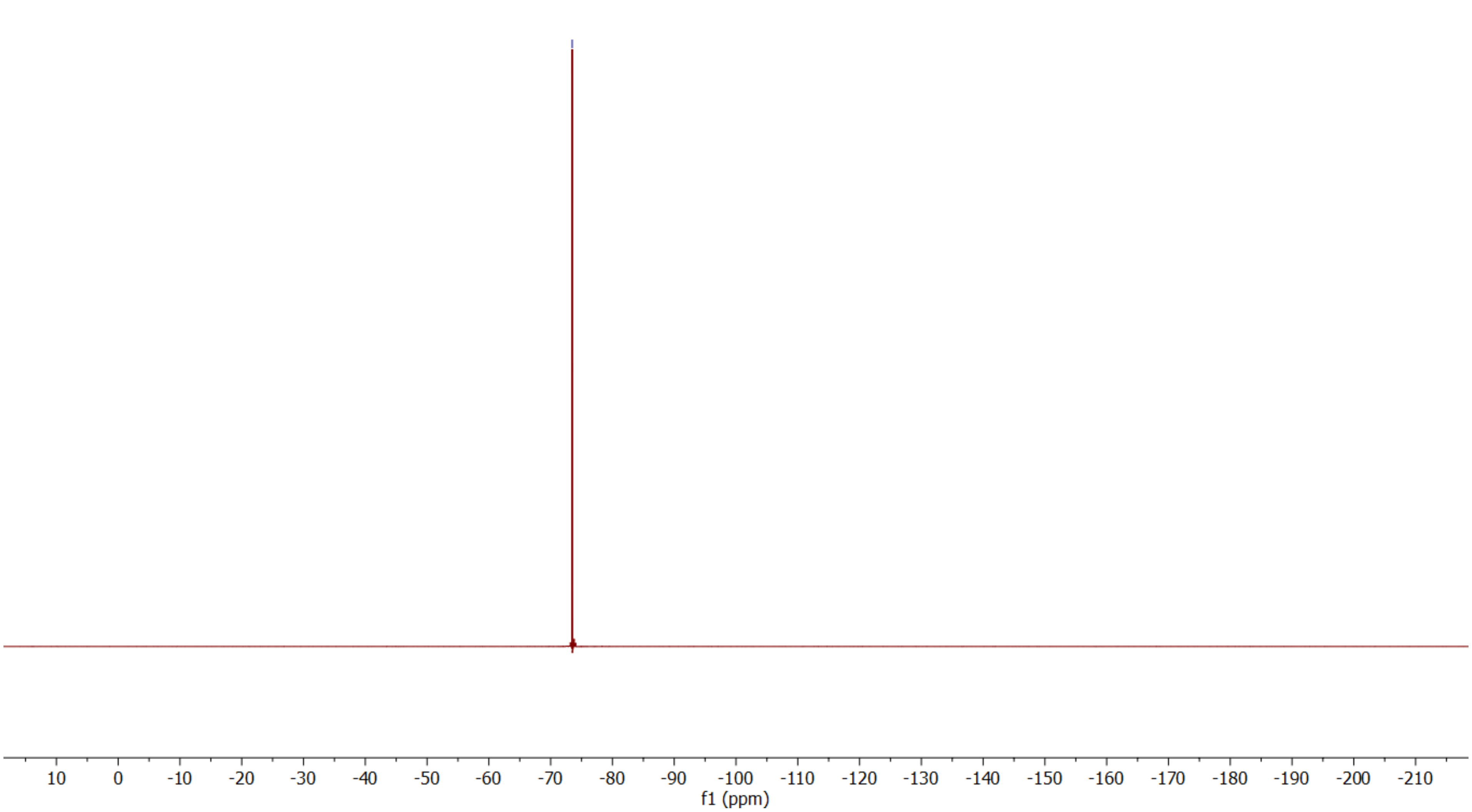

13C (100.63 MHz, CDCl3)

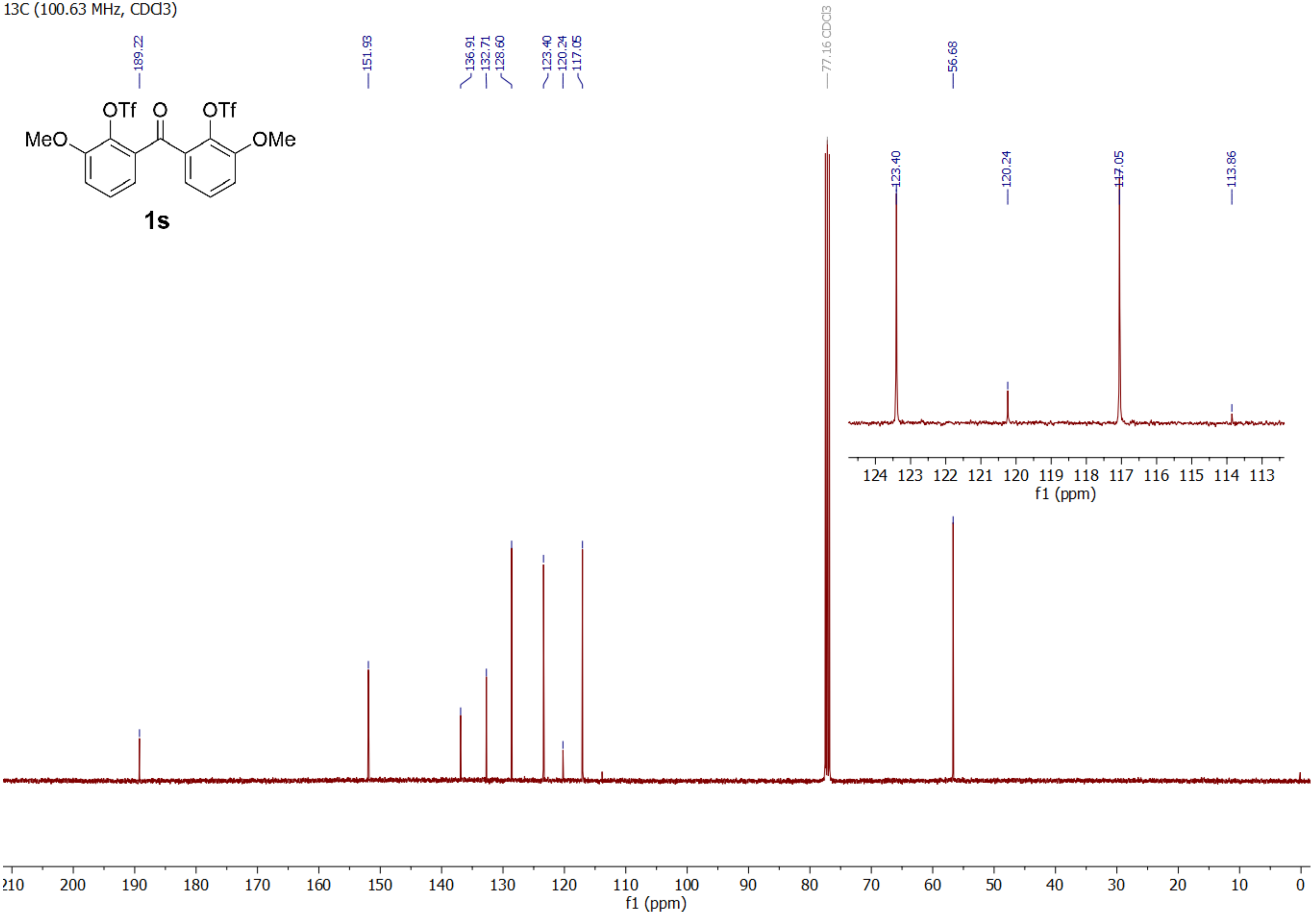

1H (400.15 MHz, CDCl3)

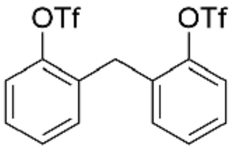


**1t**

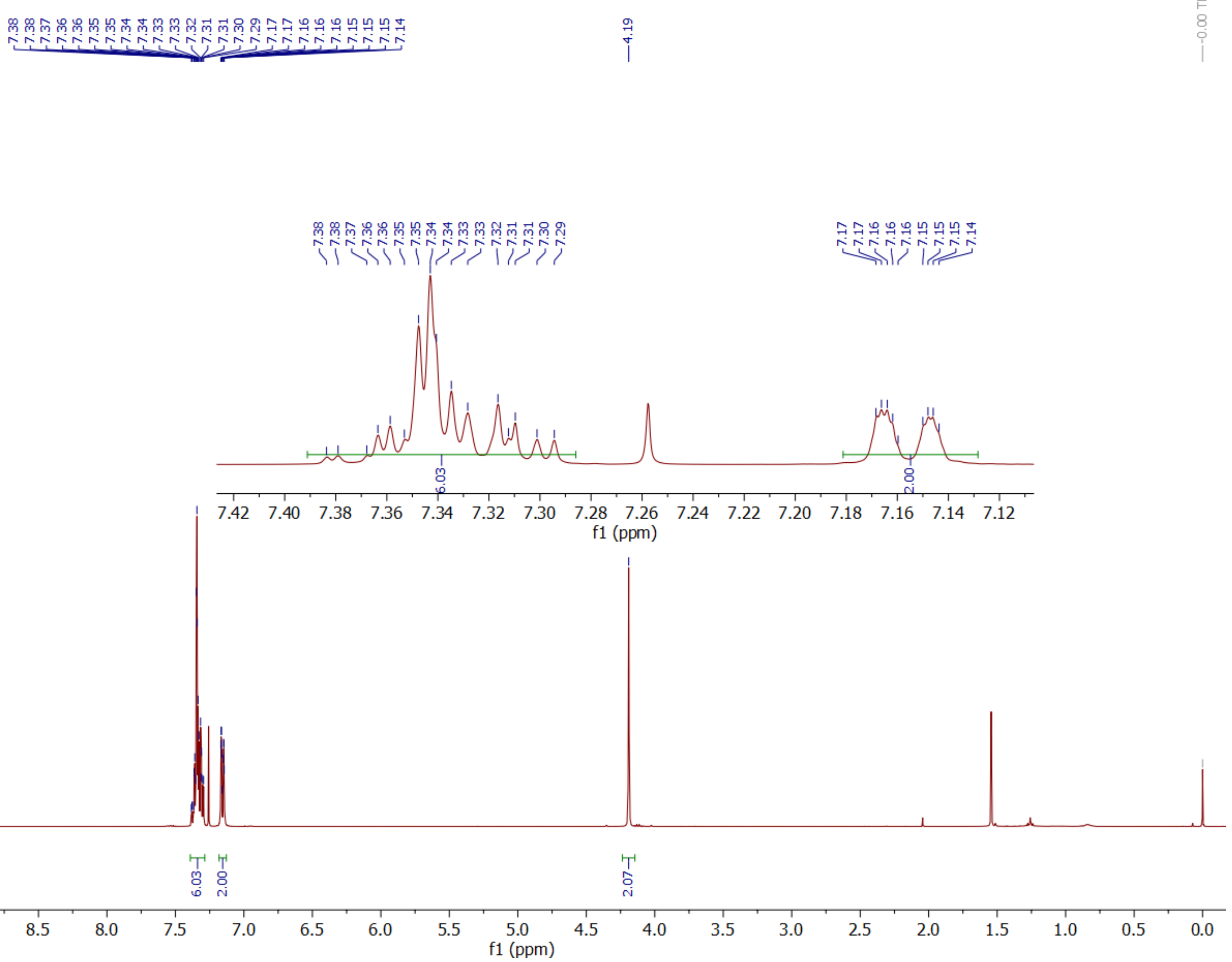

19F (376.48 MHz, CDCl3)

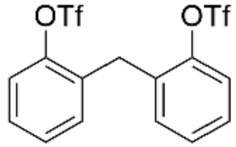


**1t**

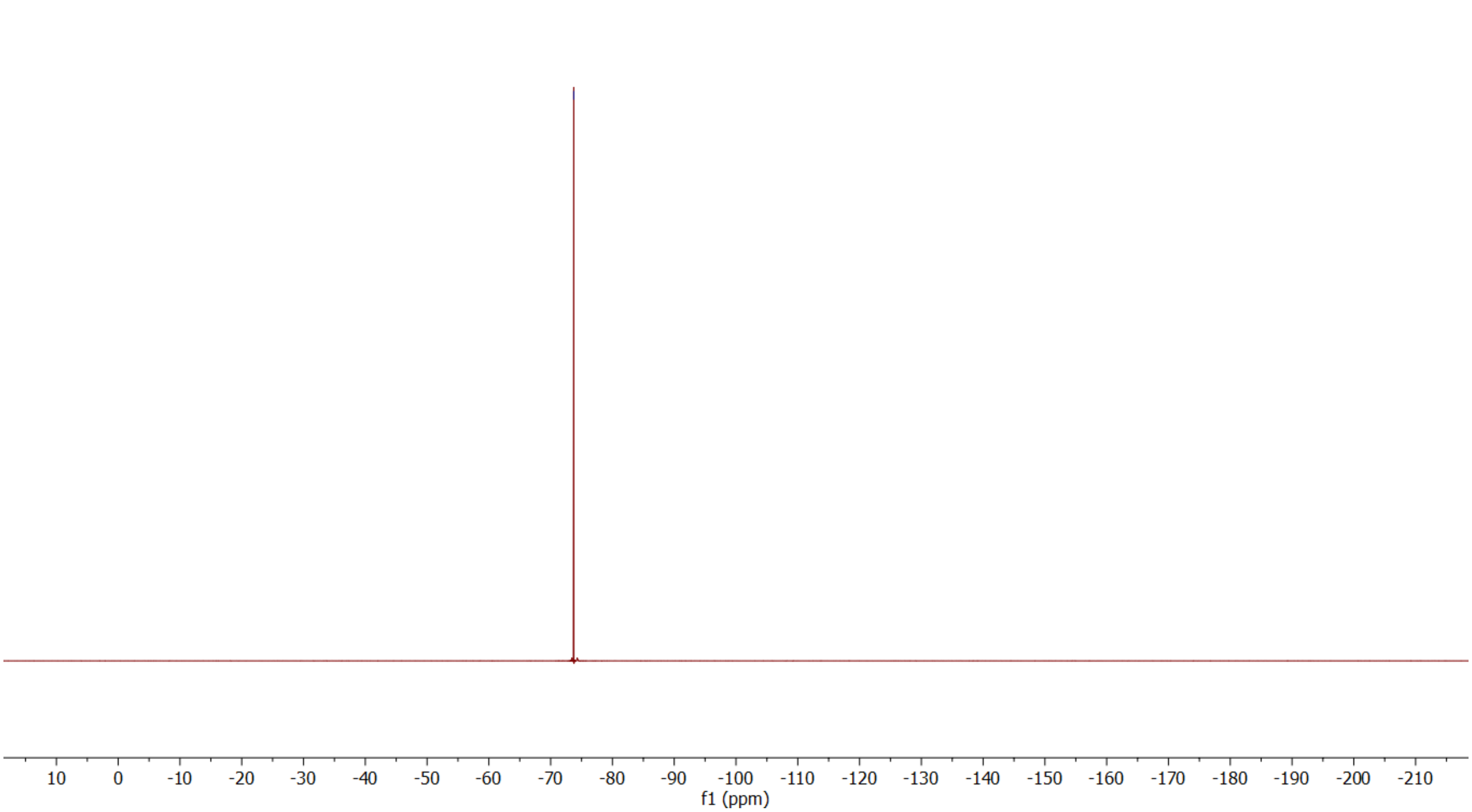

13C (100.63 MHz, CDCl3)

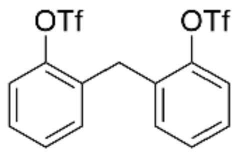


**1t**

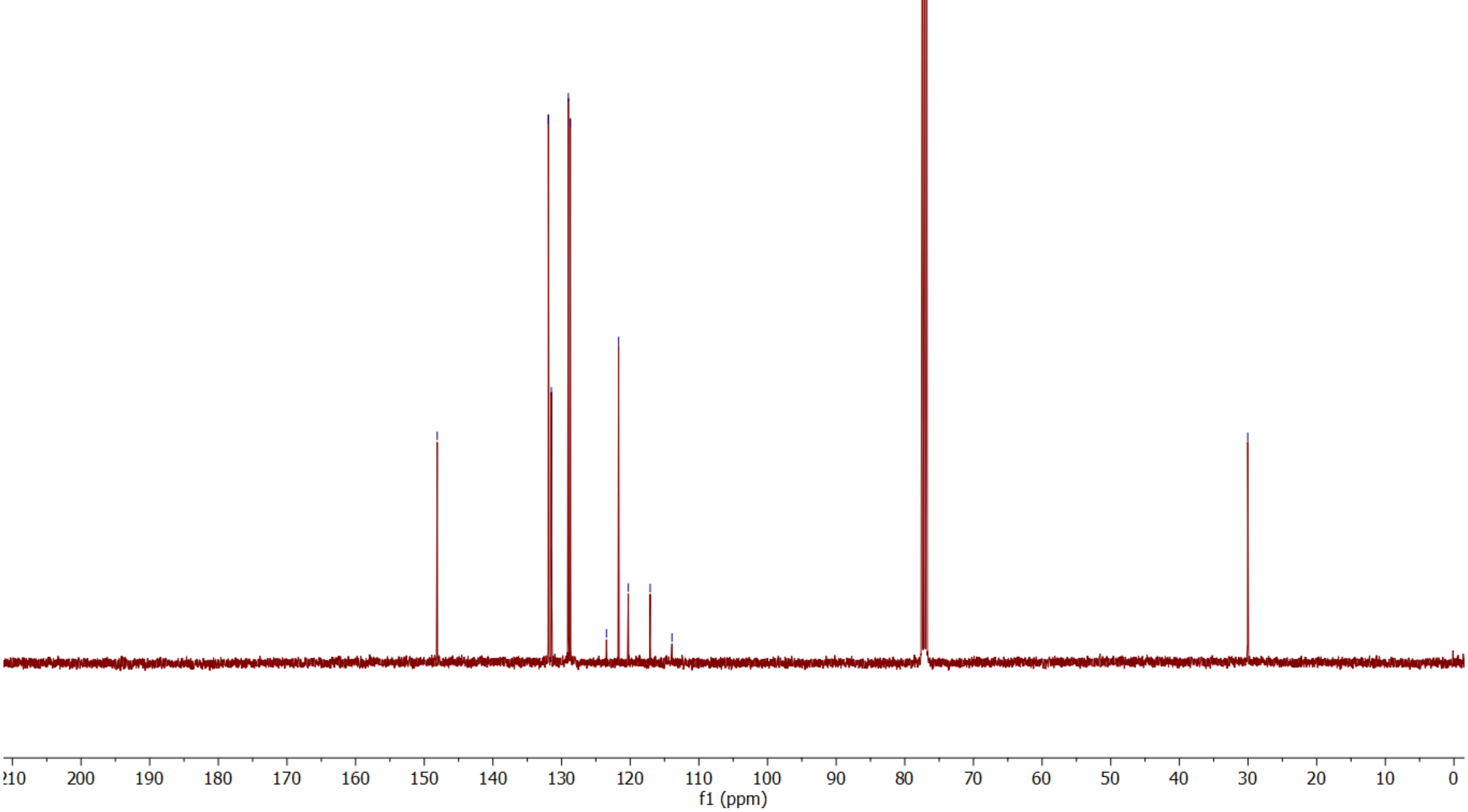

1H (400.15 MHz, CDCl3)

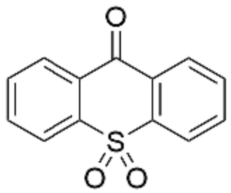

**2a**

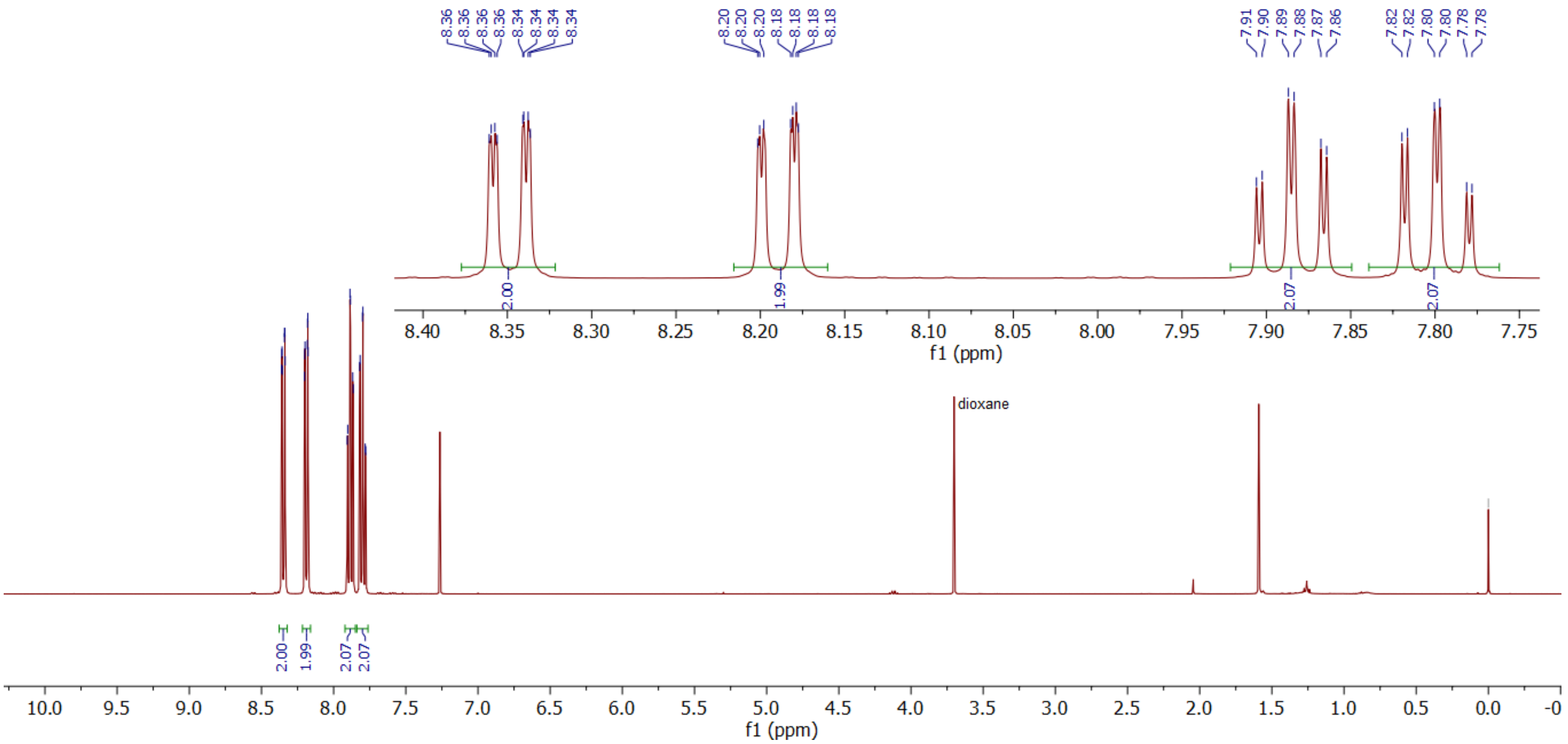

13C (100.63 MHz, CDCl3)

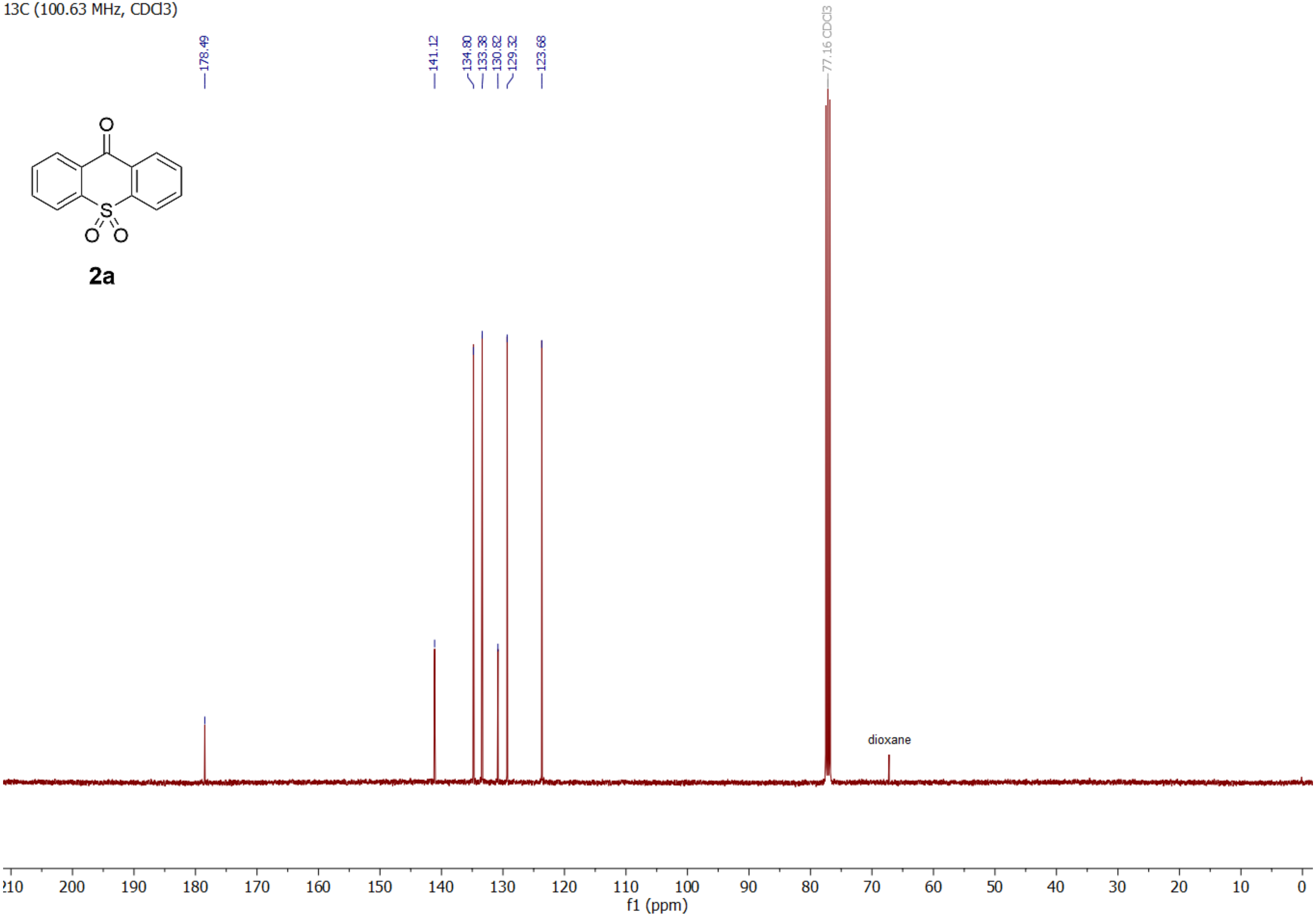

1H (400.15 MHz, CDCl3)

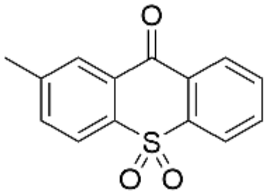

**2b**

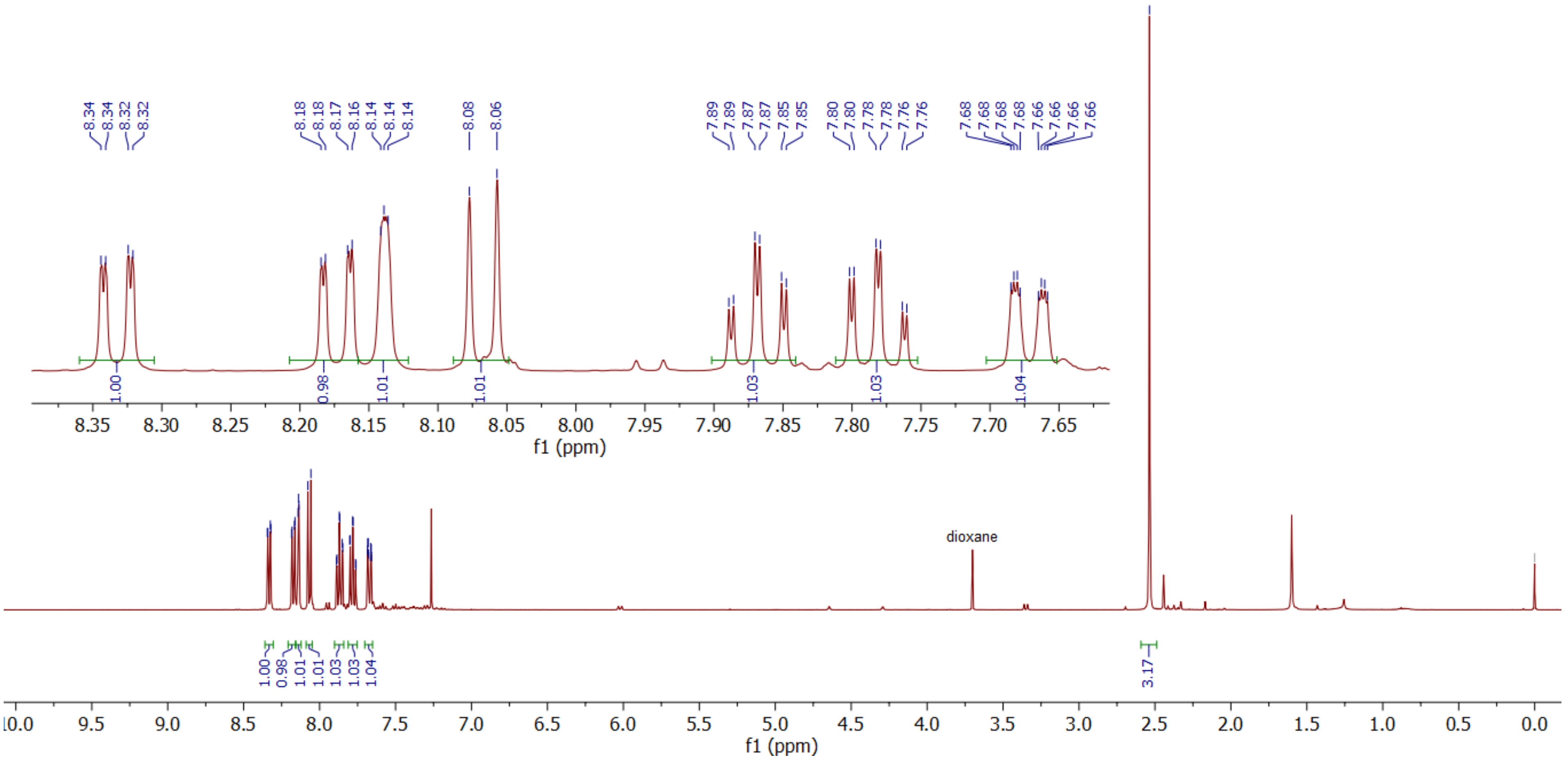

13C (100.63 MHz, CDCl3)

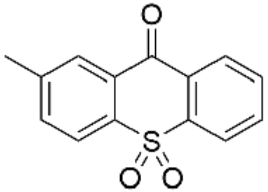

**2b**

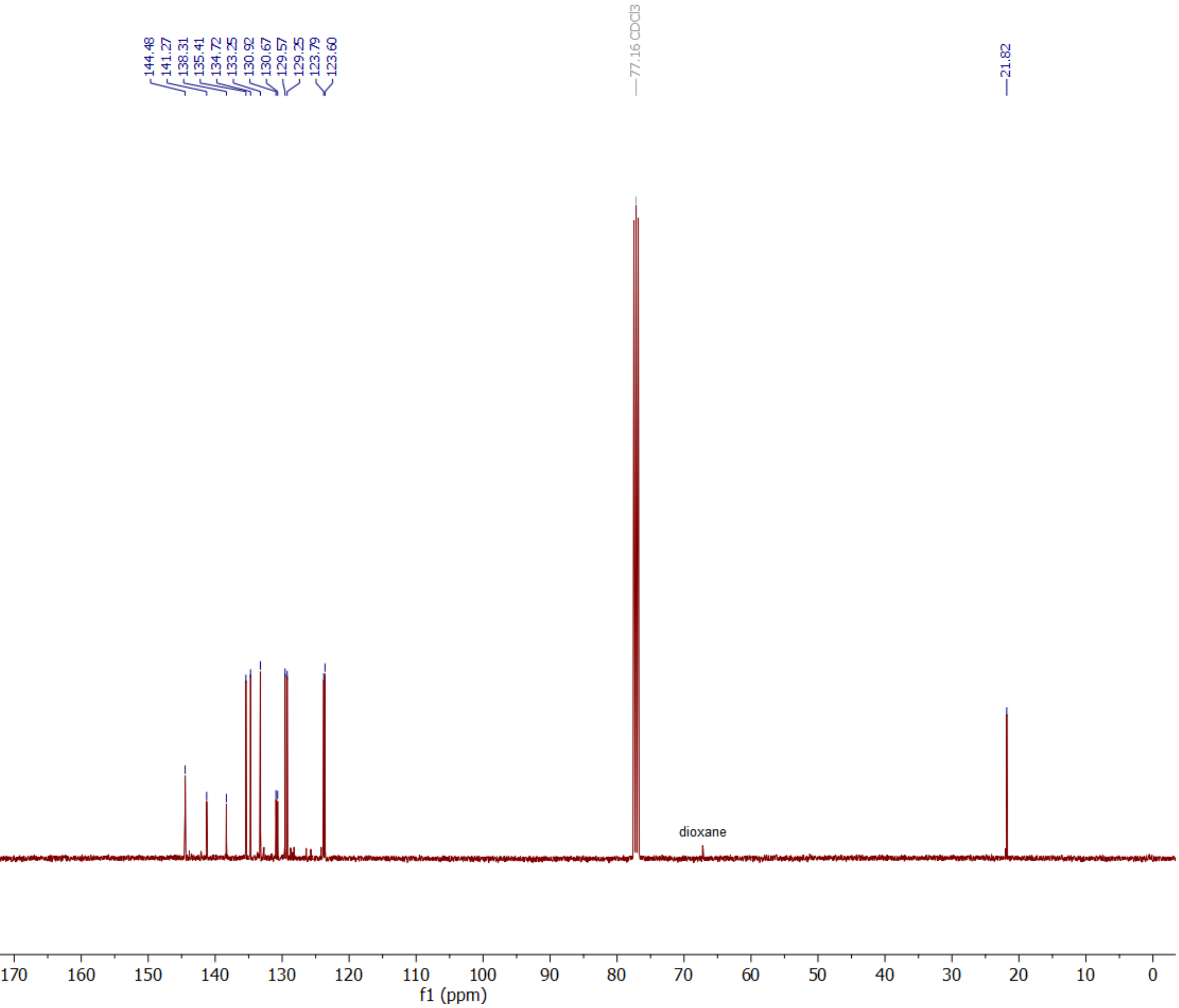

1H (400.15 MHz, CDCl3)

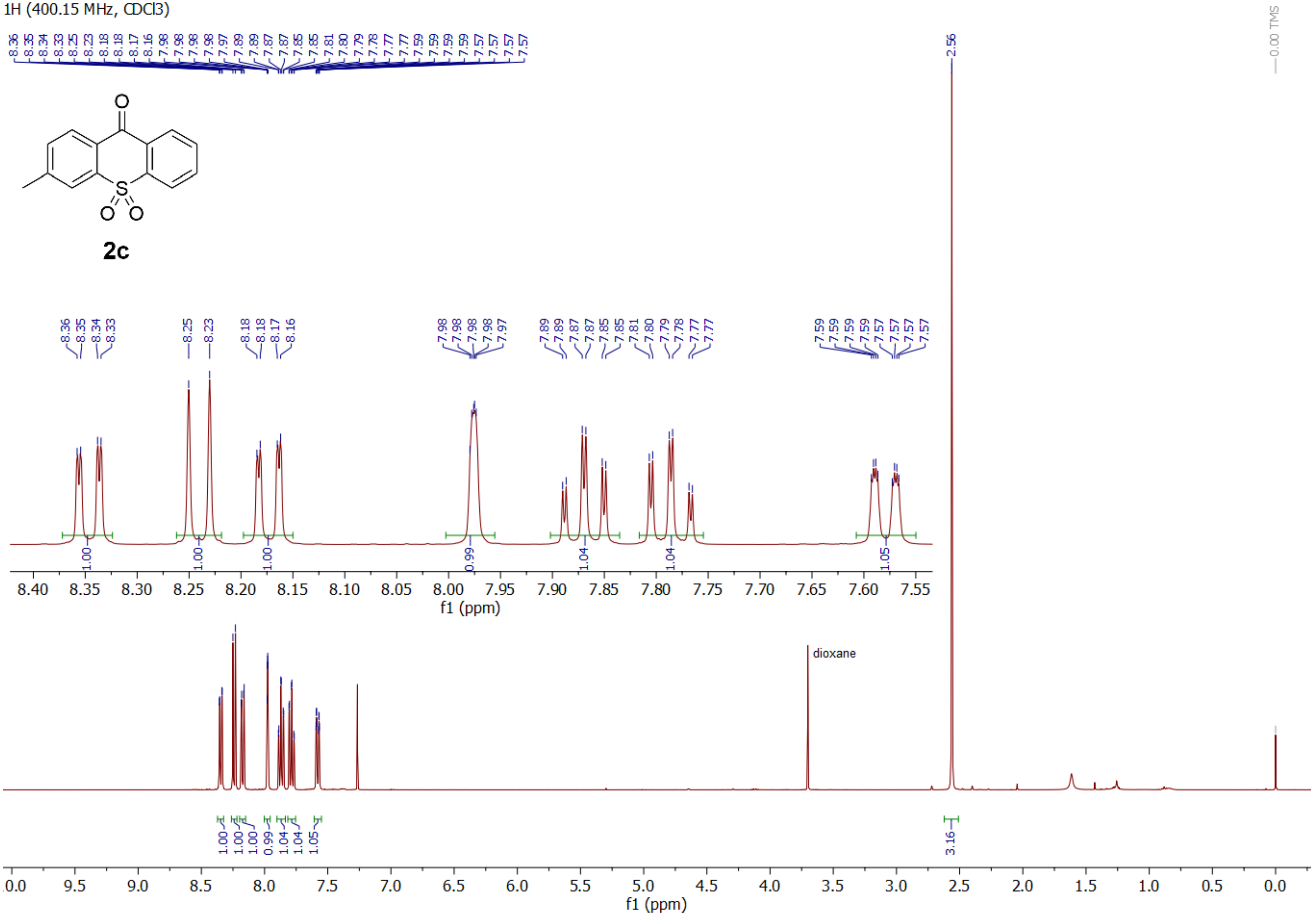


2c

13C (100.63 MHz, CDCl3)

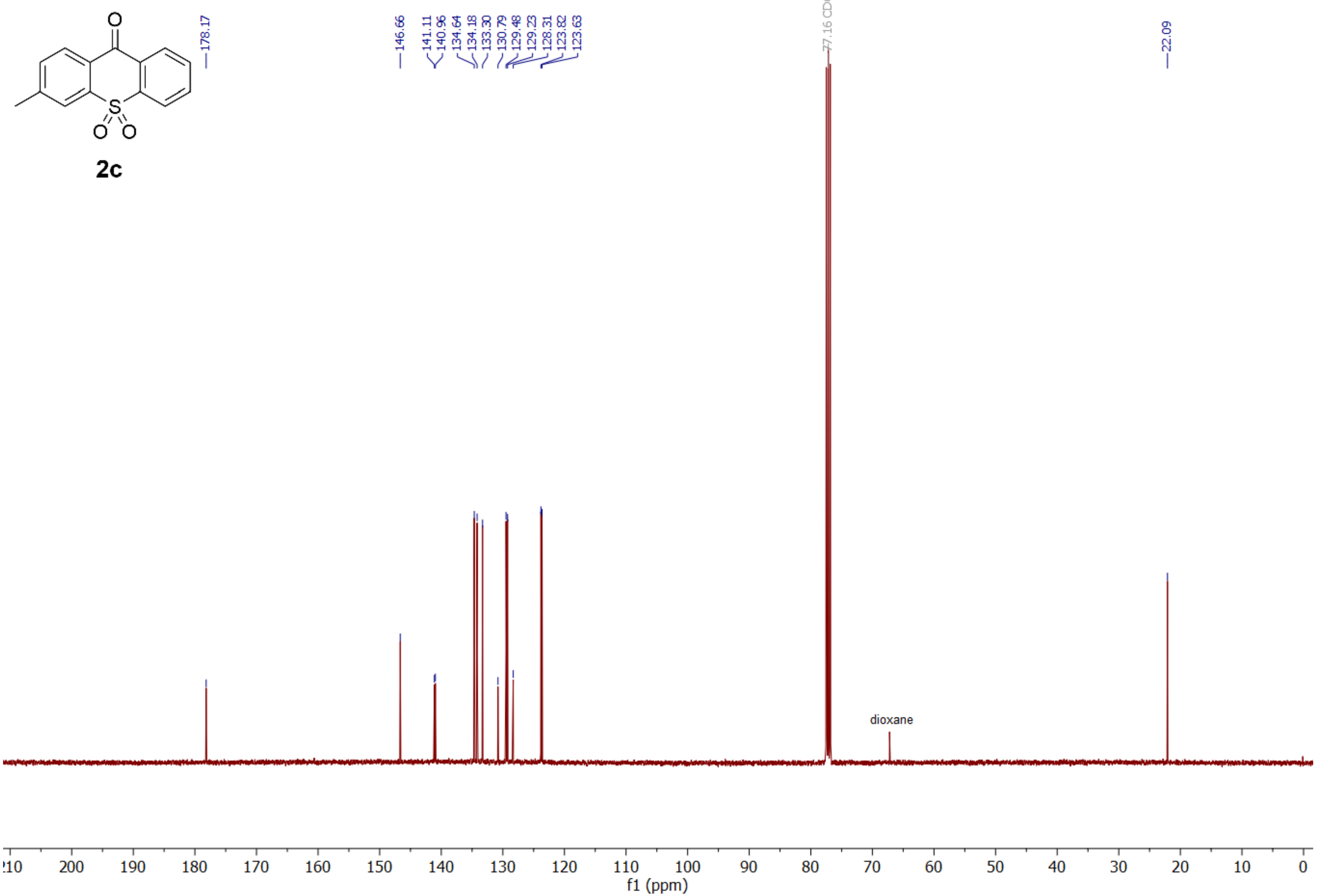

1H (400.15 MHz, CDCl3)

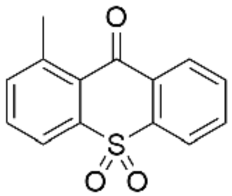

**2d**

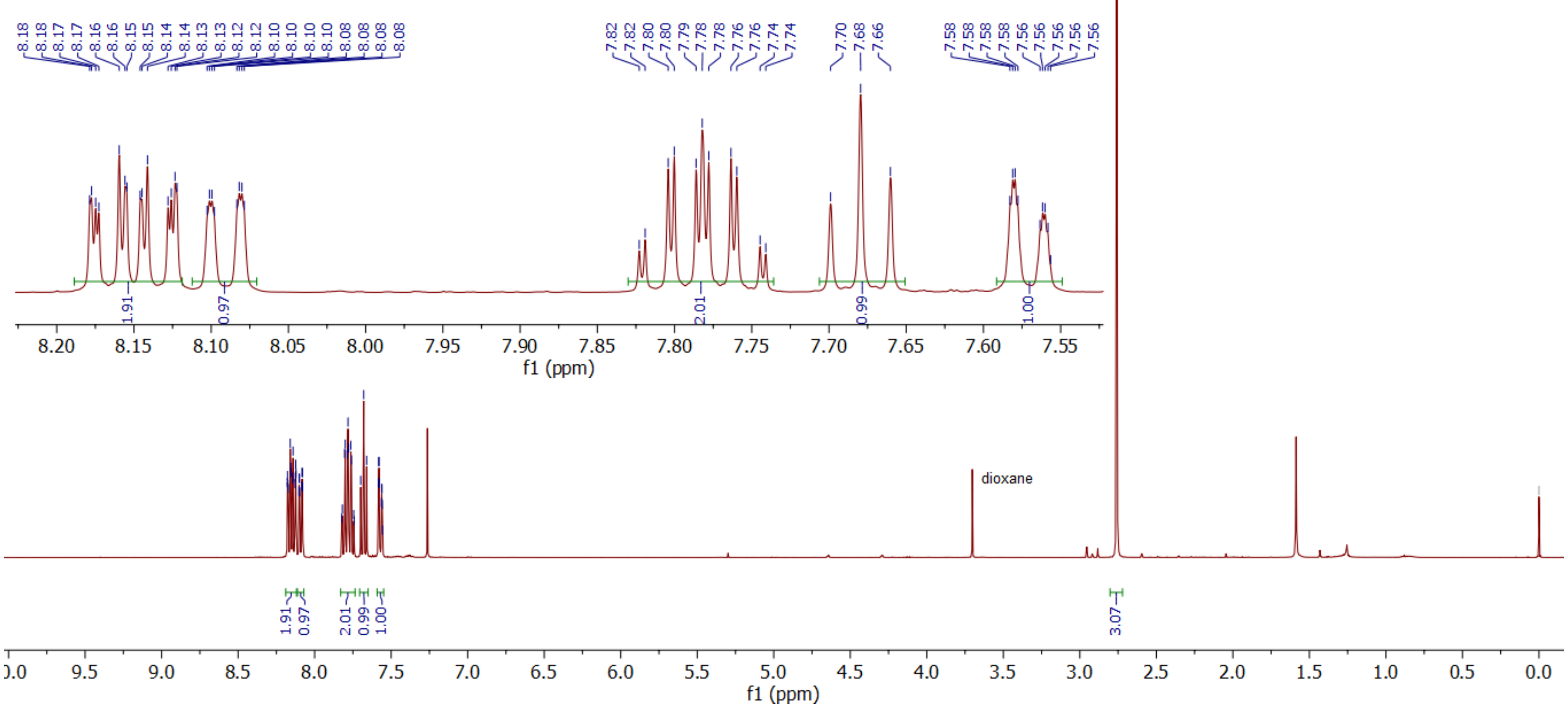

13C (100.63 MHz, CDCl3)

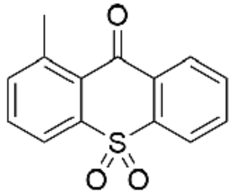

**2d**

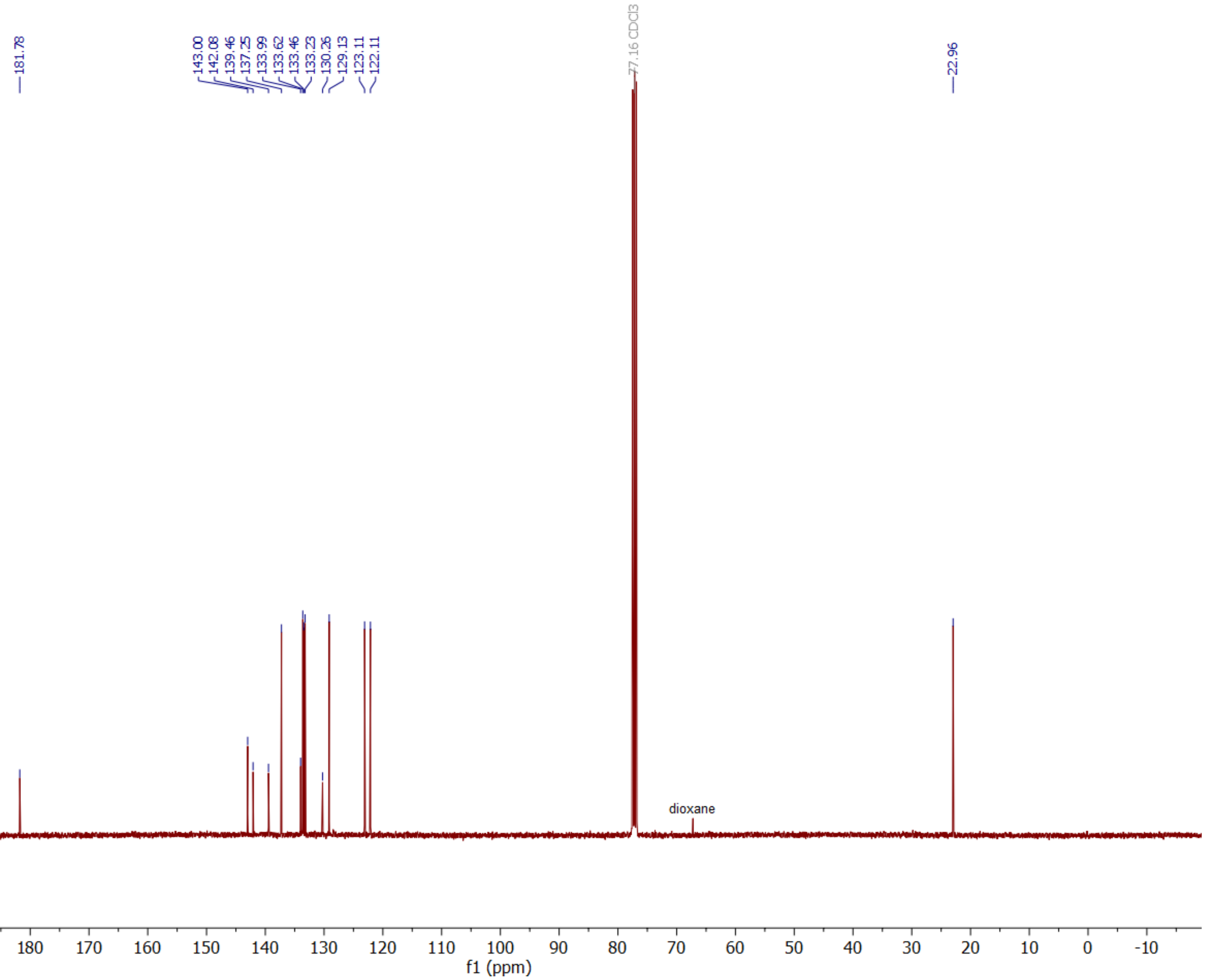

1H (400.15 MHz, CDCl3)

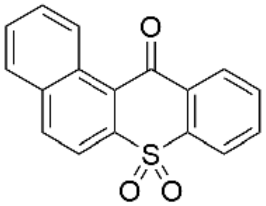

**2e**

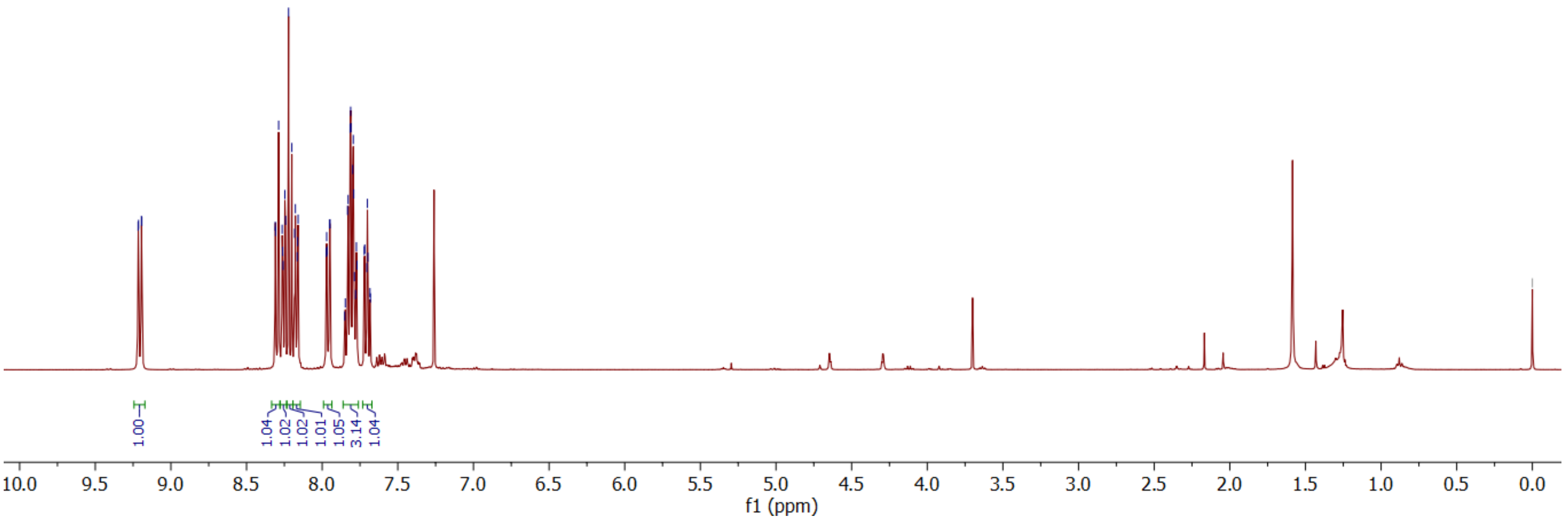

13C (100.63 MHz, CDCl3)

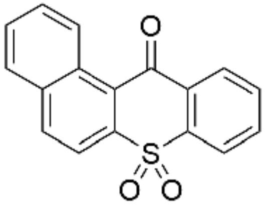

**2e**

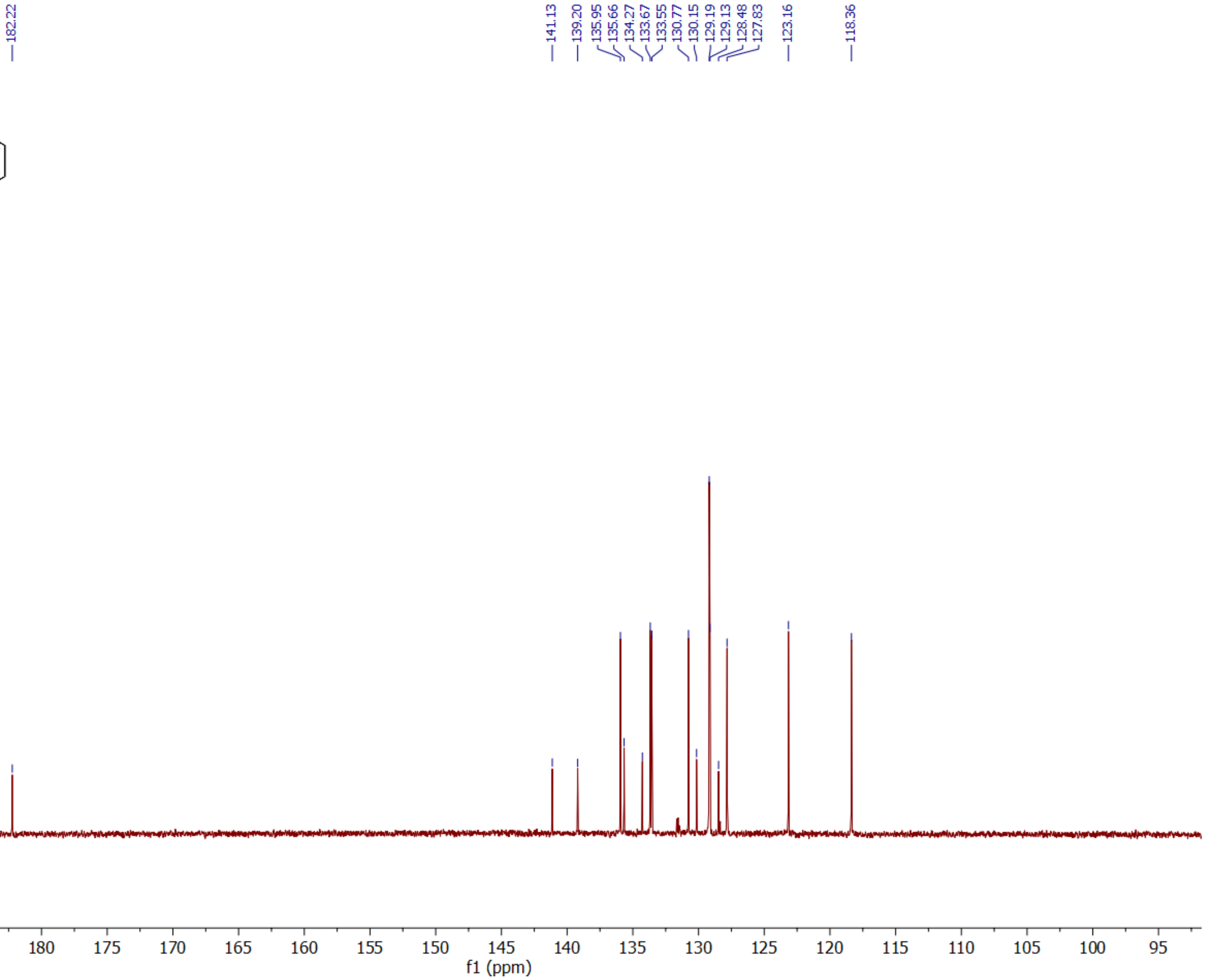

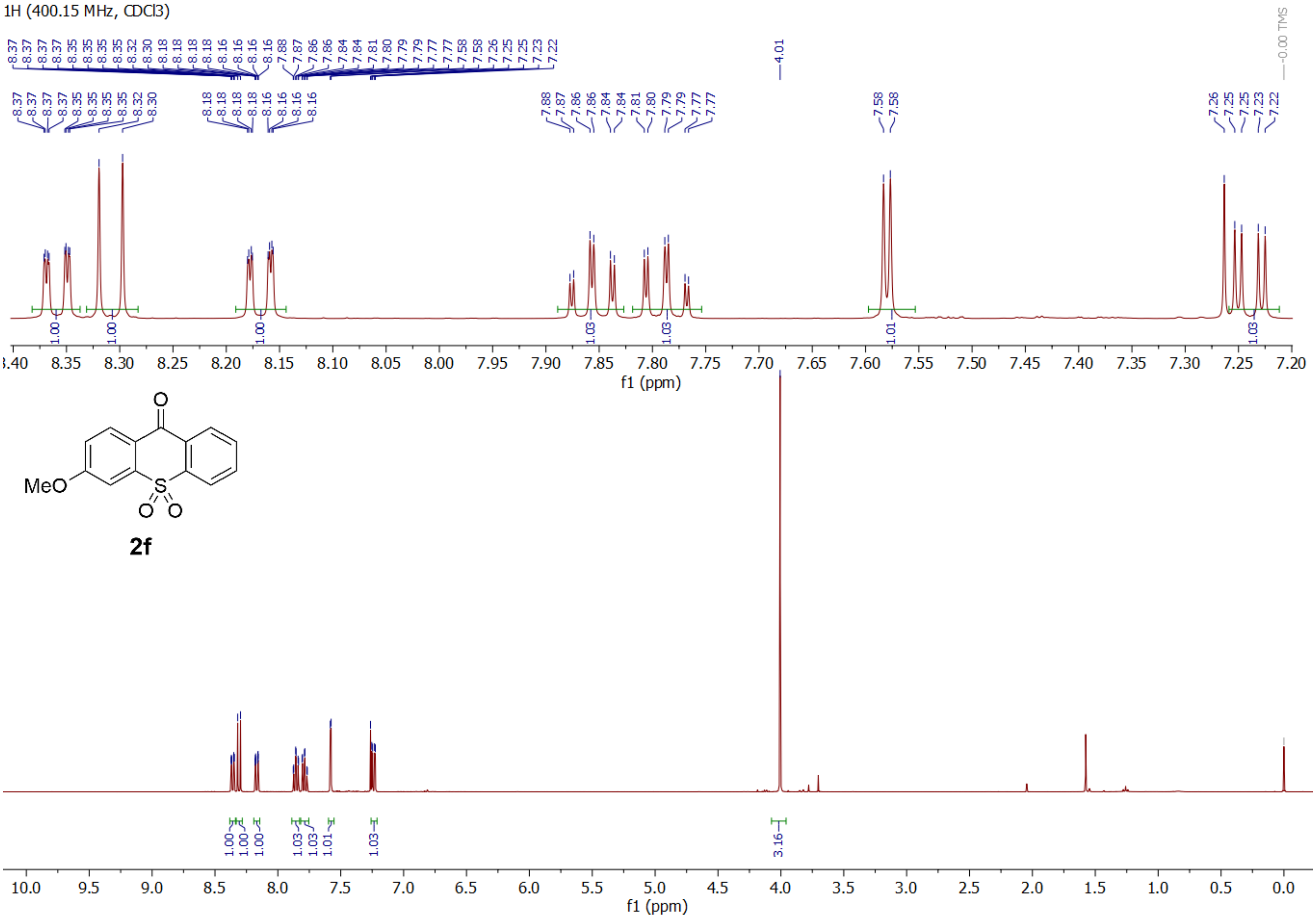
1H (400.15 MHz, CDCl3)
MeO
2f
f1 (ppm)
f1 (ppm)
4.01
-0.00 TMS
8.37 8.37 8.37 8.37 8.35 8.35 8.35 8.35 8.32 8.30 8.18 8.18 8.18 8.18 8.16 8.16 8.16 8.16 7.88 7.87 7.86 7.86 7.84 7.84 7.81 7.80 7.79 7.79 7.77 7.77 7.58 7.58 7.26 7.25 7.25 7.23 7.22
1.00 1.00 1.00 1.03 1.03 1.01 1.03 3.16

13C (100.63 MHz, CDCl3)

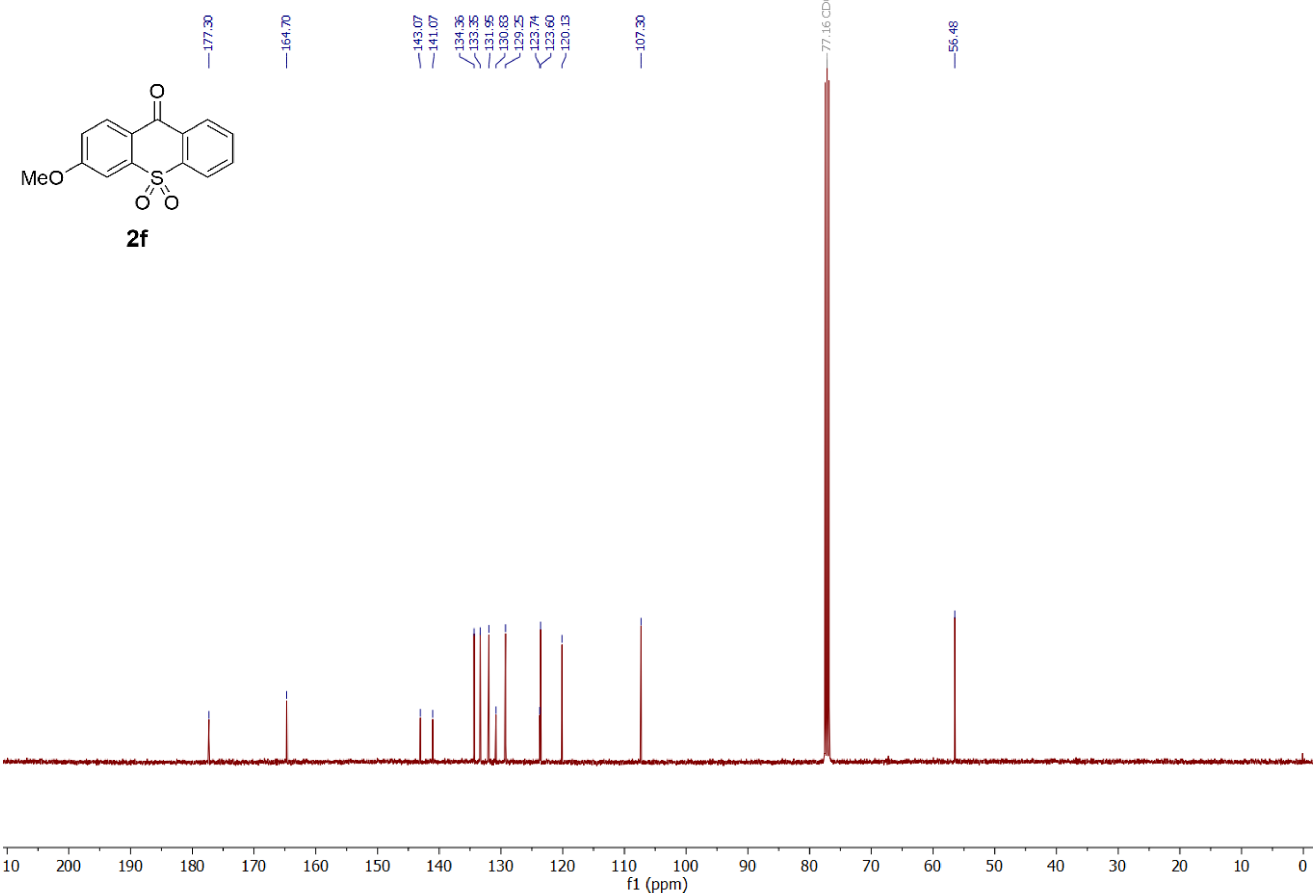

1H (400.15 MHz, CDCl3)

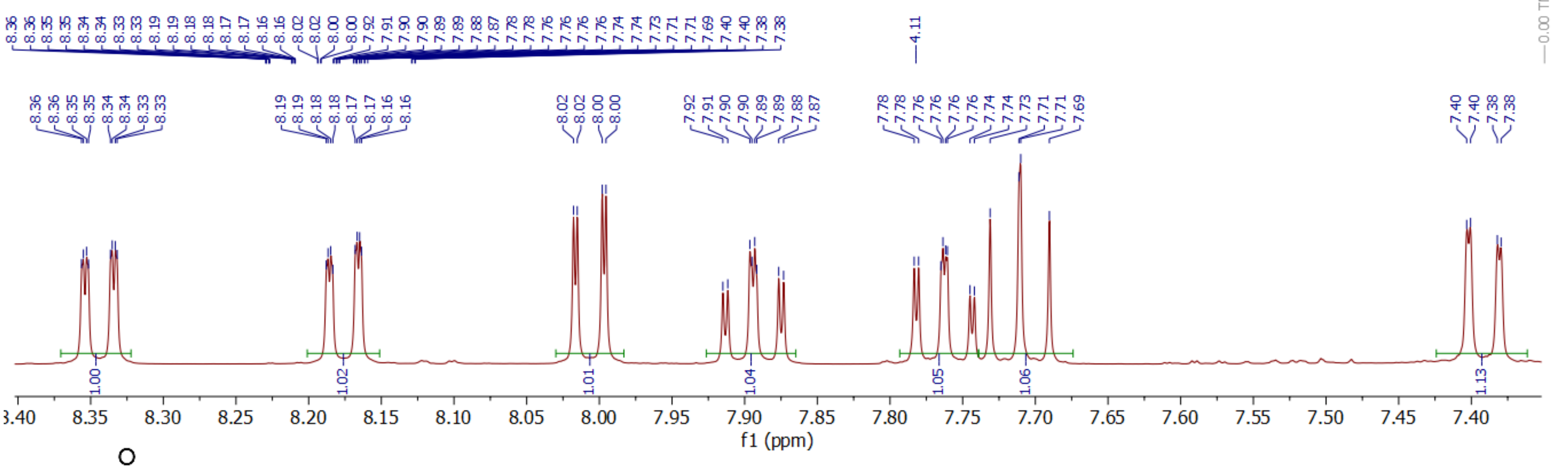

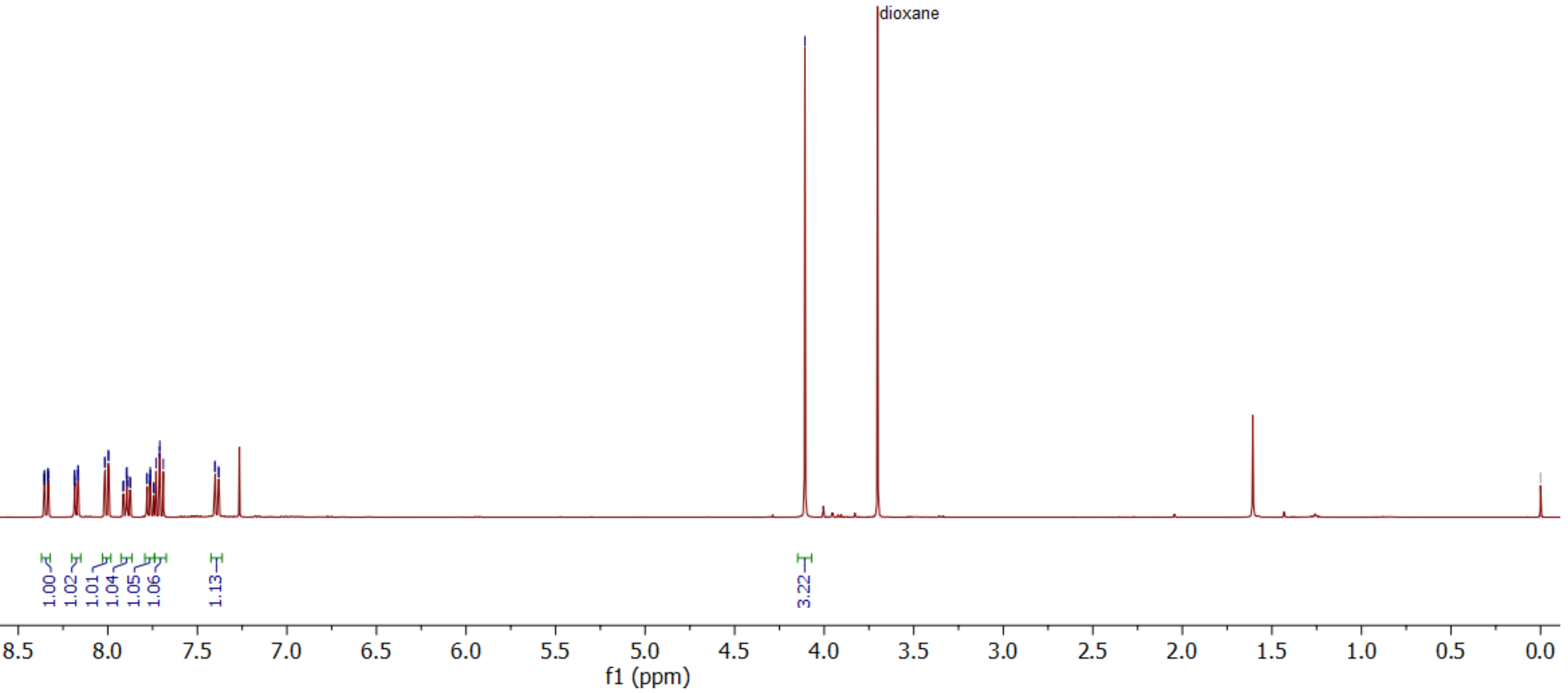


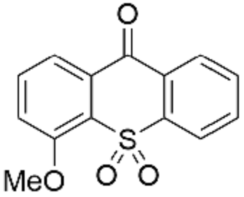


**2g**

13C (100.63 MHz, CDCl3)

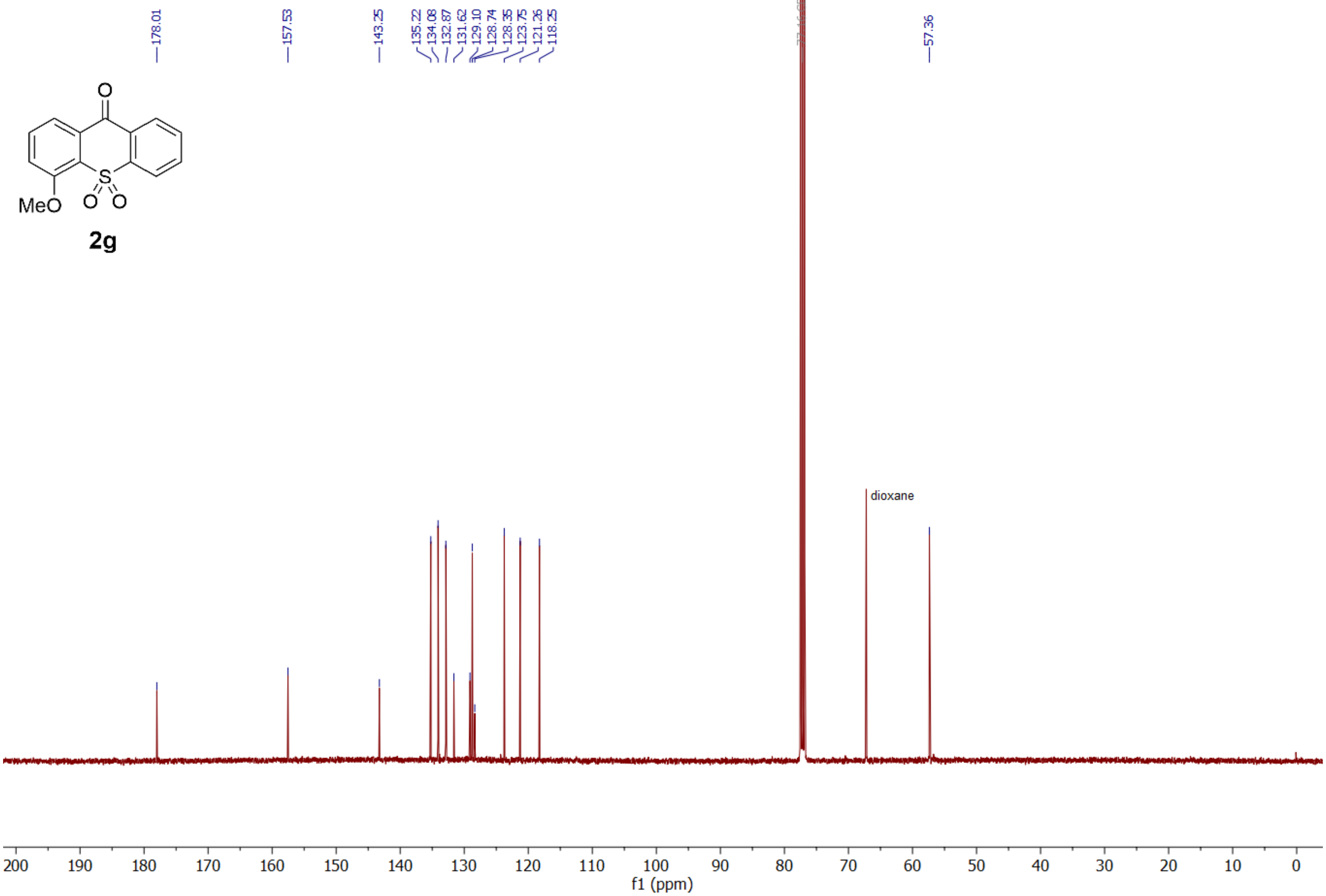

1H (400.15 MHz, CDCl3)

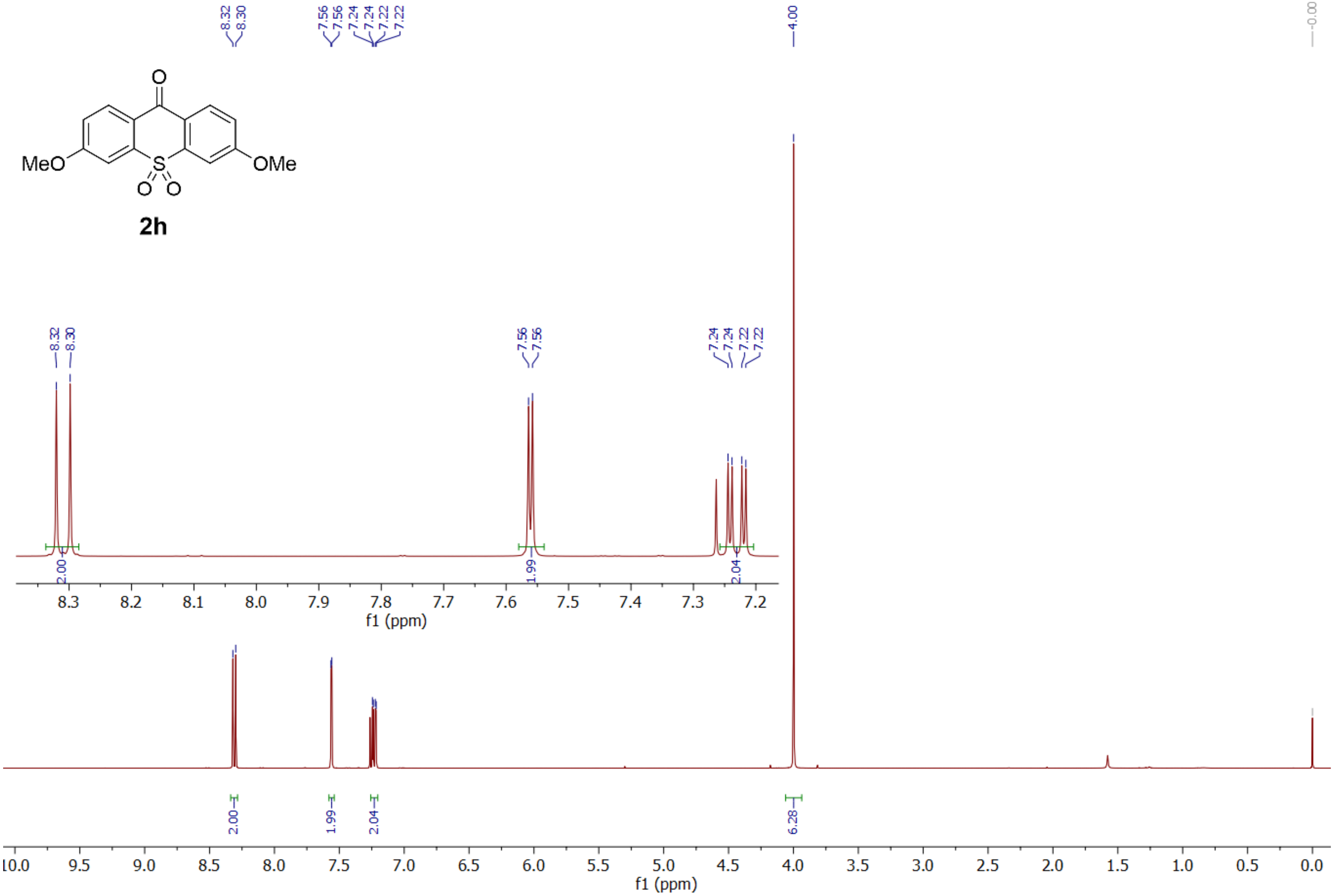

13C (100.63 MHz, CDCl3)

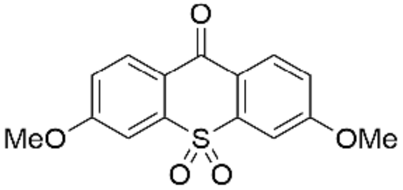

2h

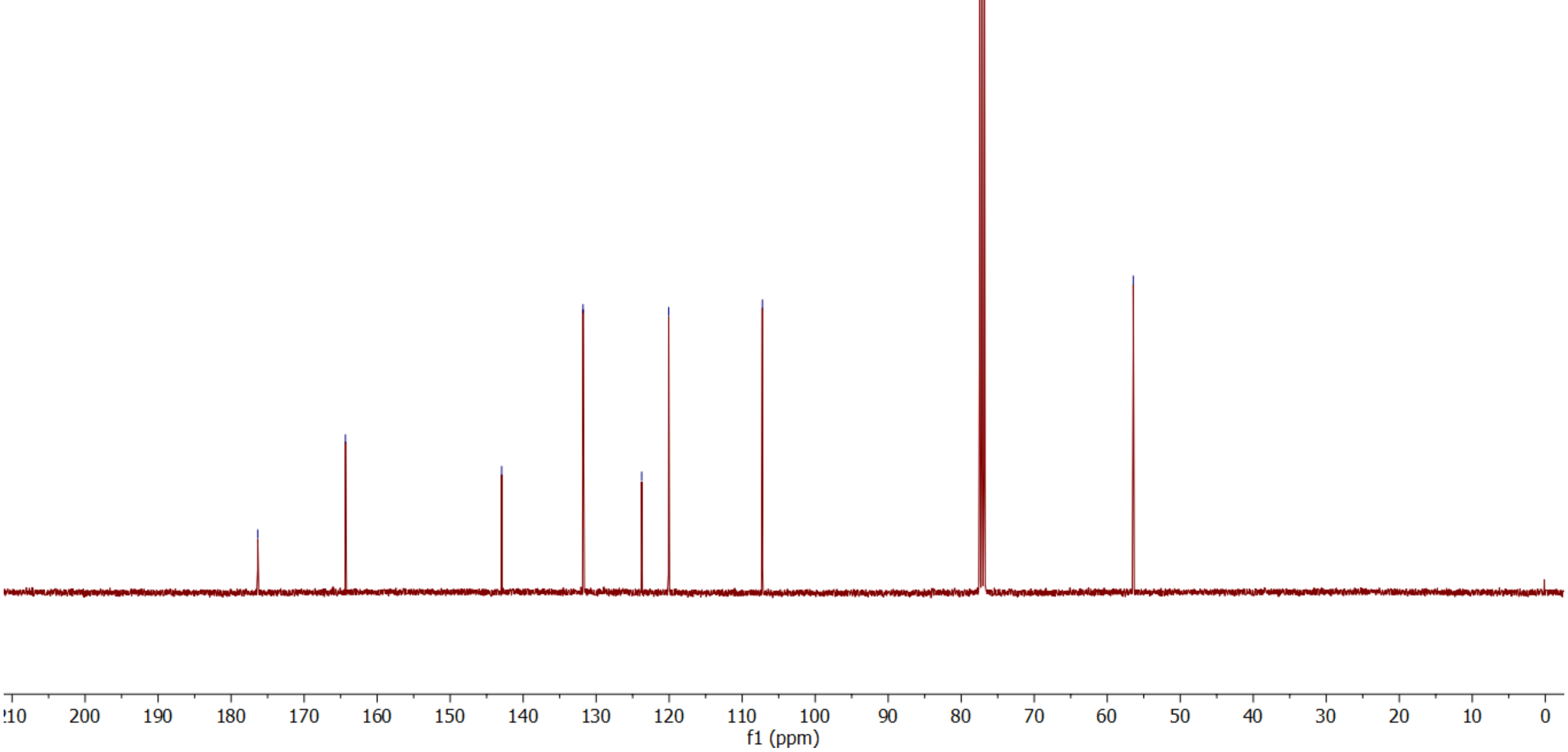

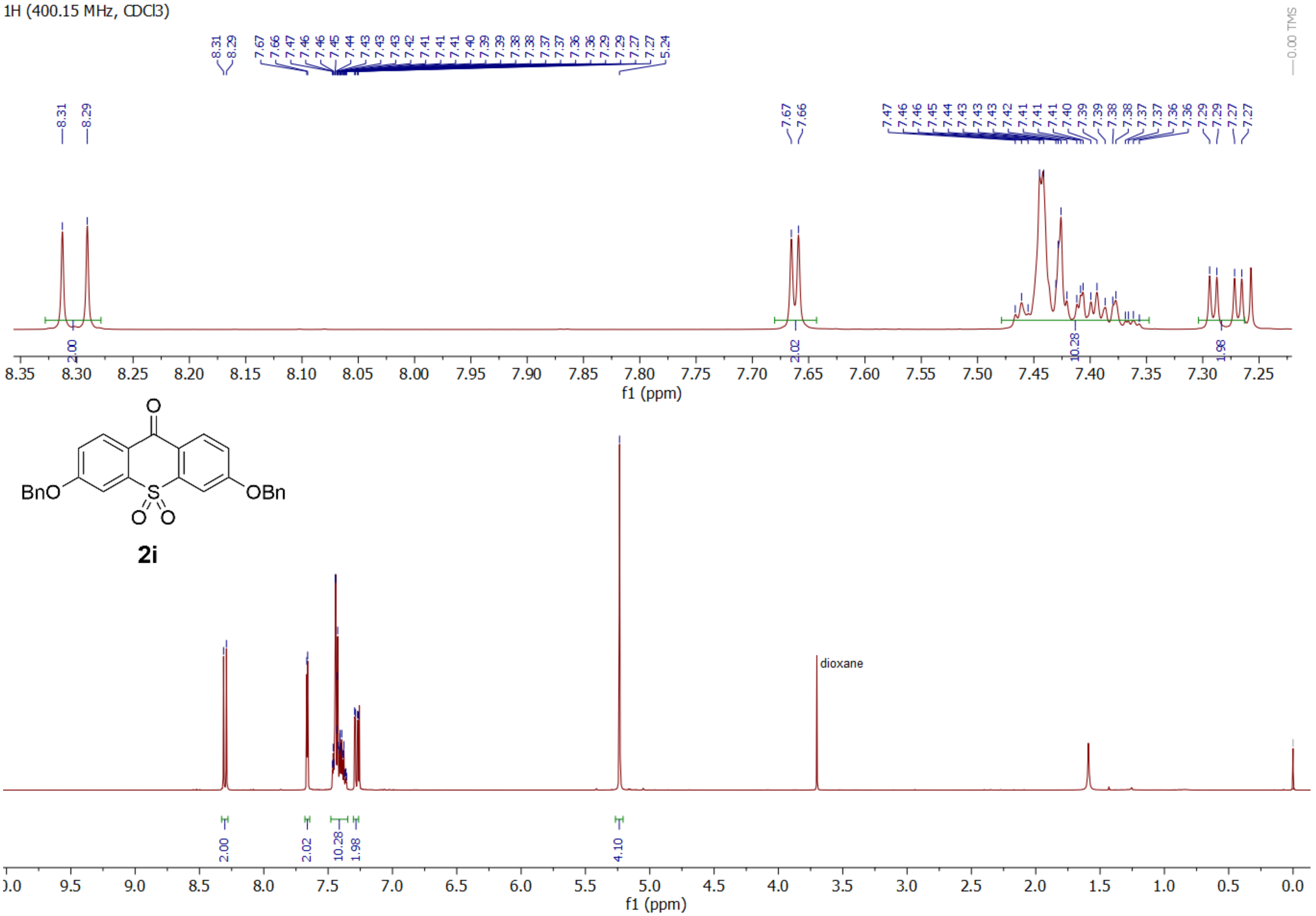
1H (400.15 MHz, CDCl3)
0.00 TMS
f1 (ppm)
BnO
OBn
2i
dioxane
f1 (ppm)

13C (100.63 MHz, CDCl3)

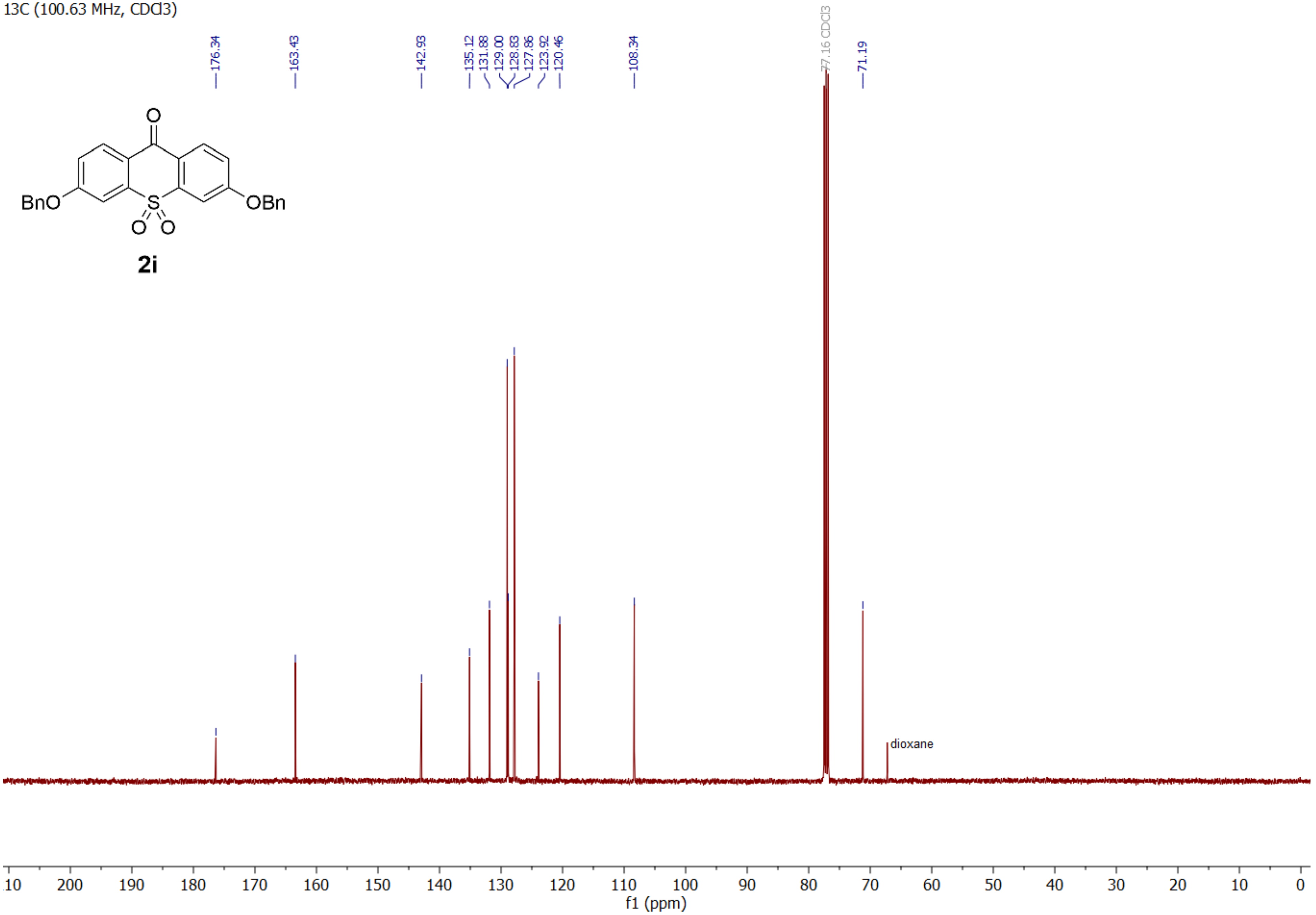


**2i**

1H (400.15 MHz, CDCl3)

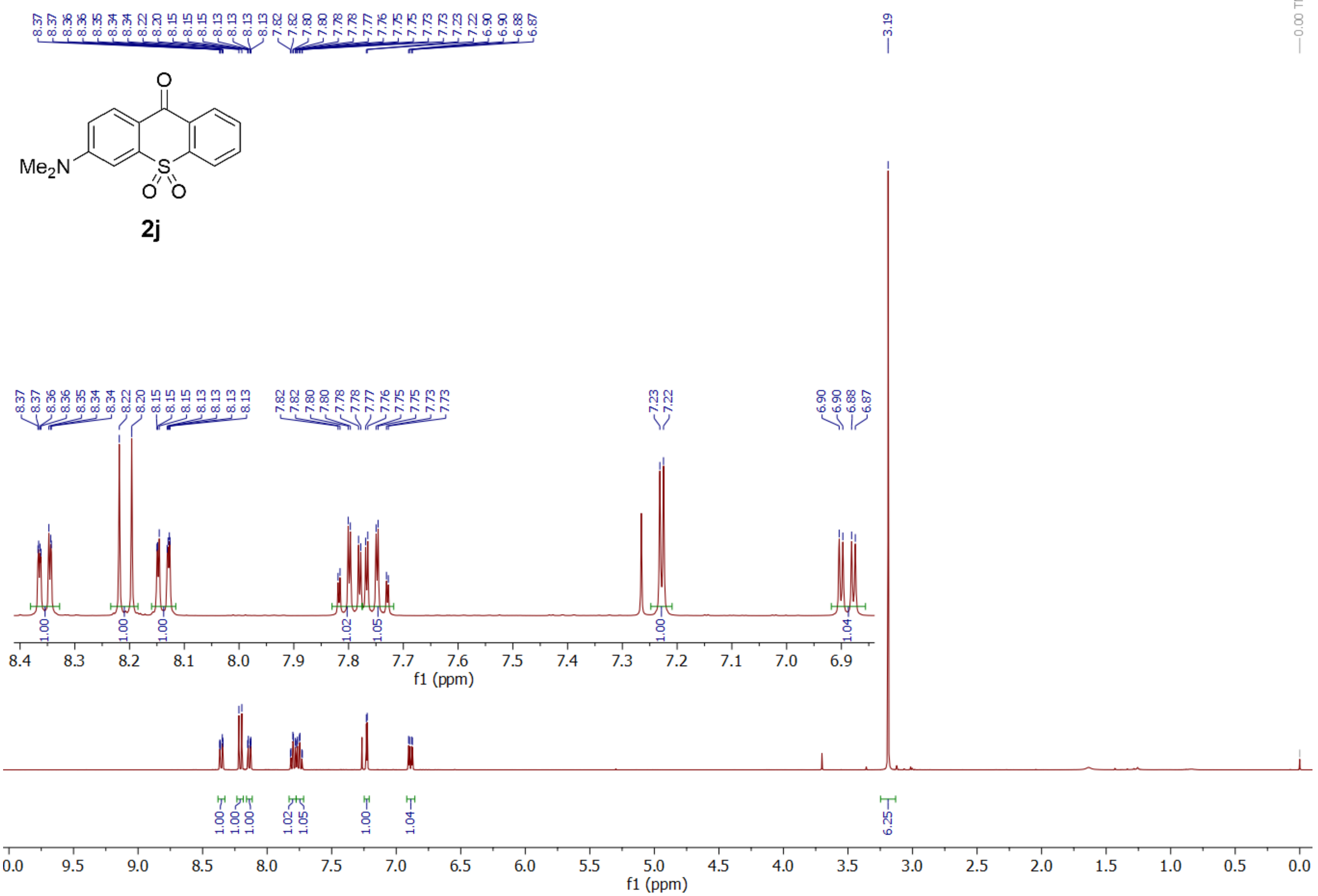

13C (100.63 MHz, CDCl3)

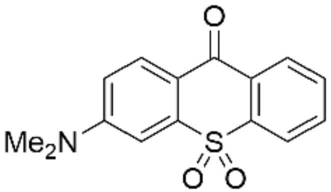

2j

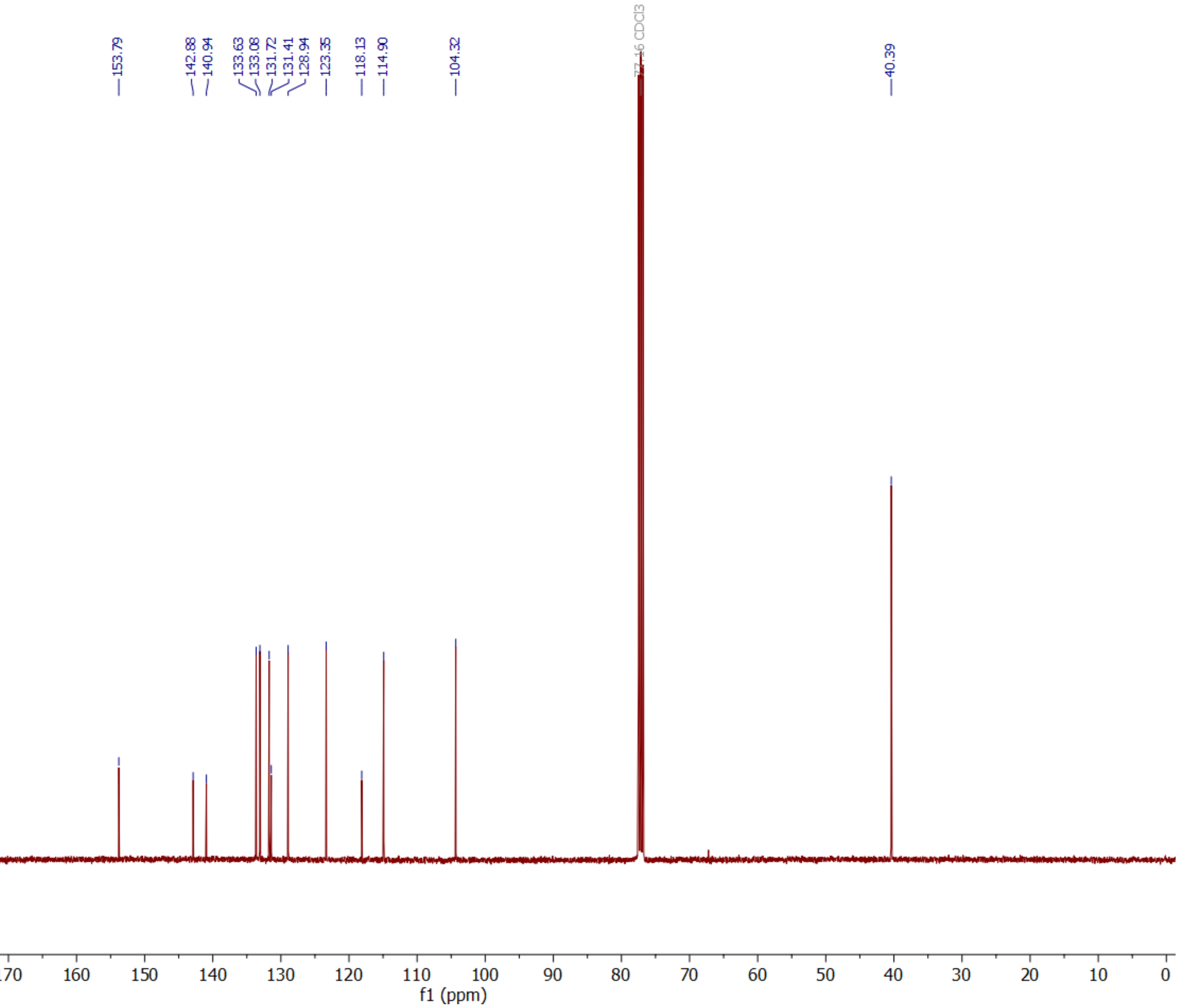

1H (400.15 MHz, CDCl3)

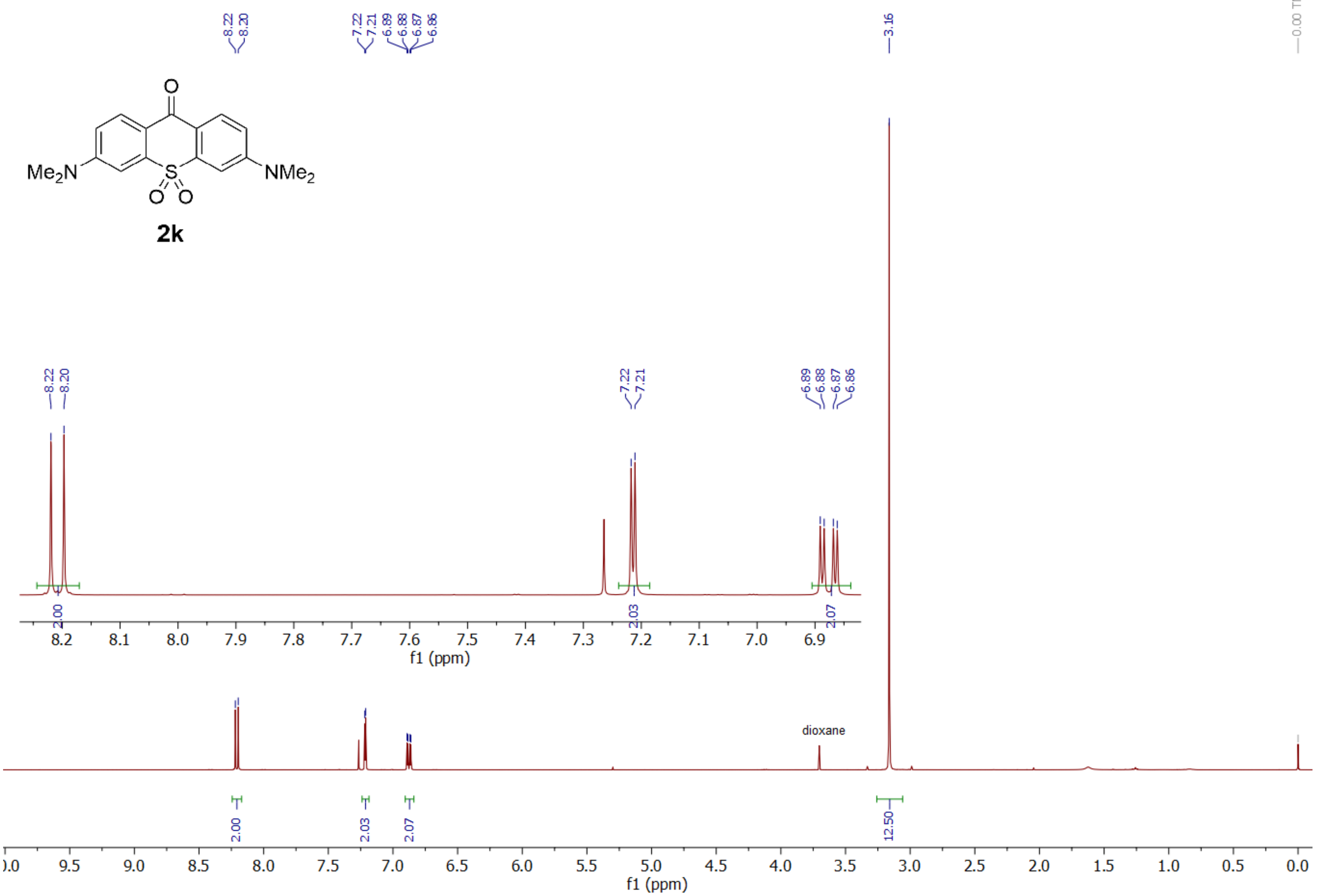

13C (100.63 MHz, CDCl3)

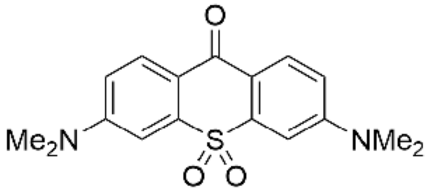

**2k**

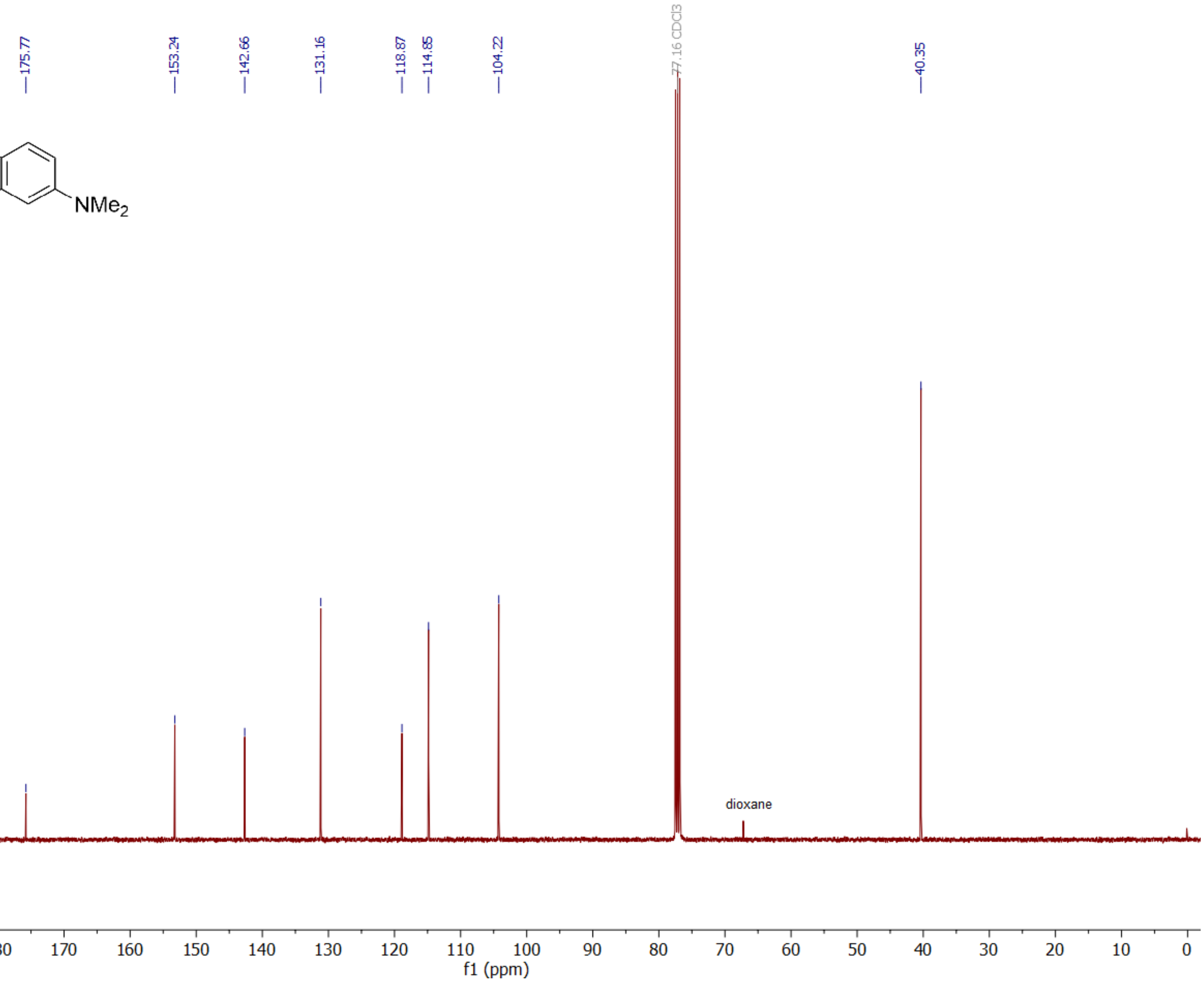

1H (400.15 MHz, CDCl3)

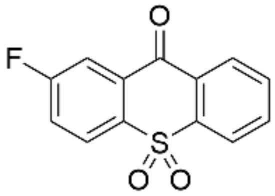

**2l**

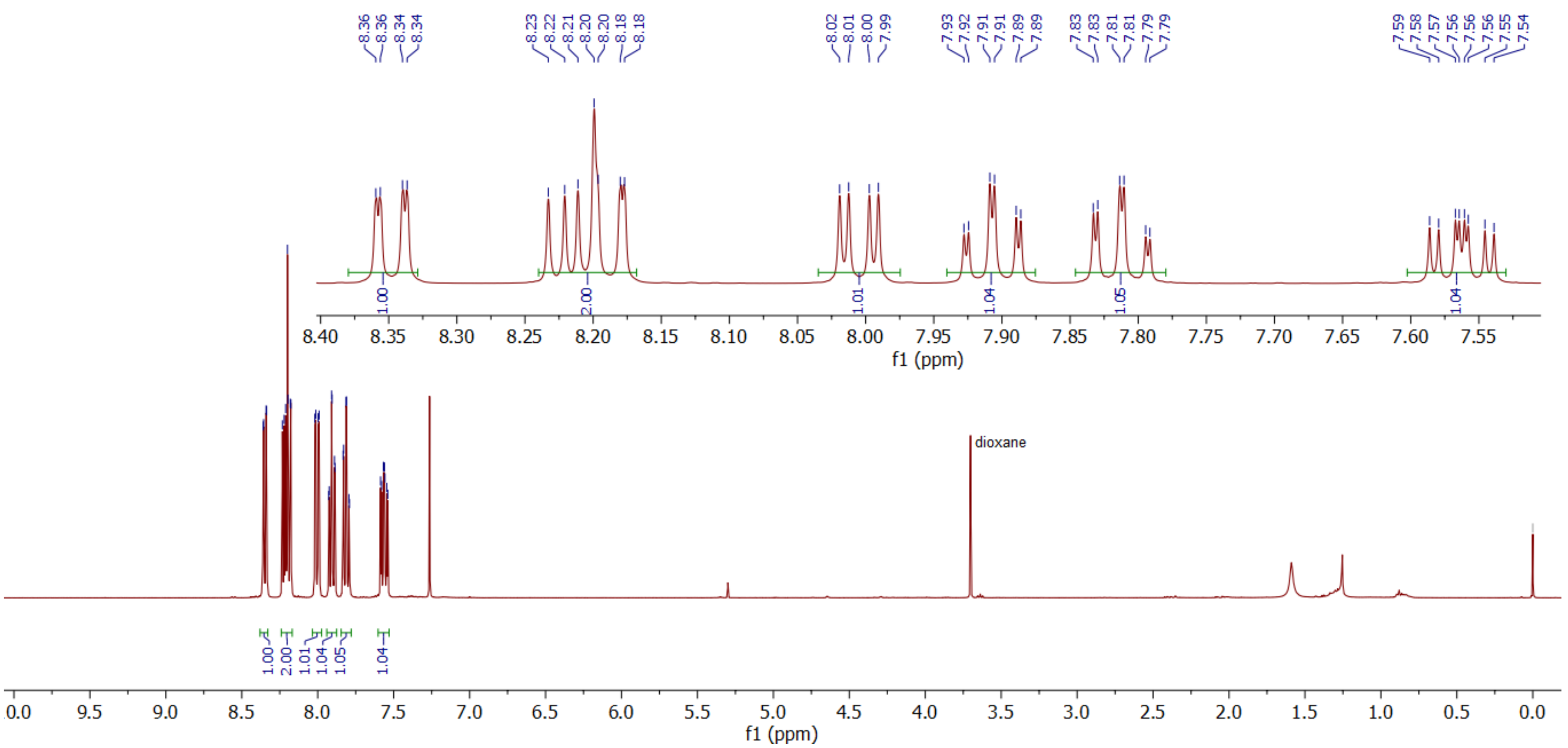

19F (376.48 MHz, CDCl3)

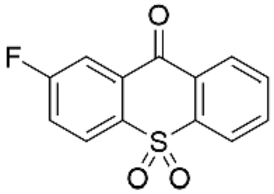

**2l**

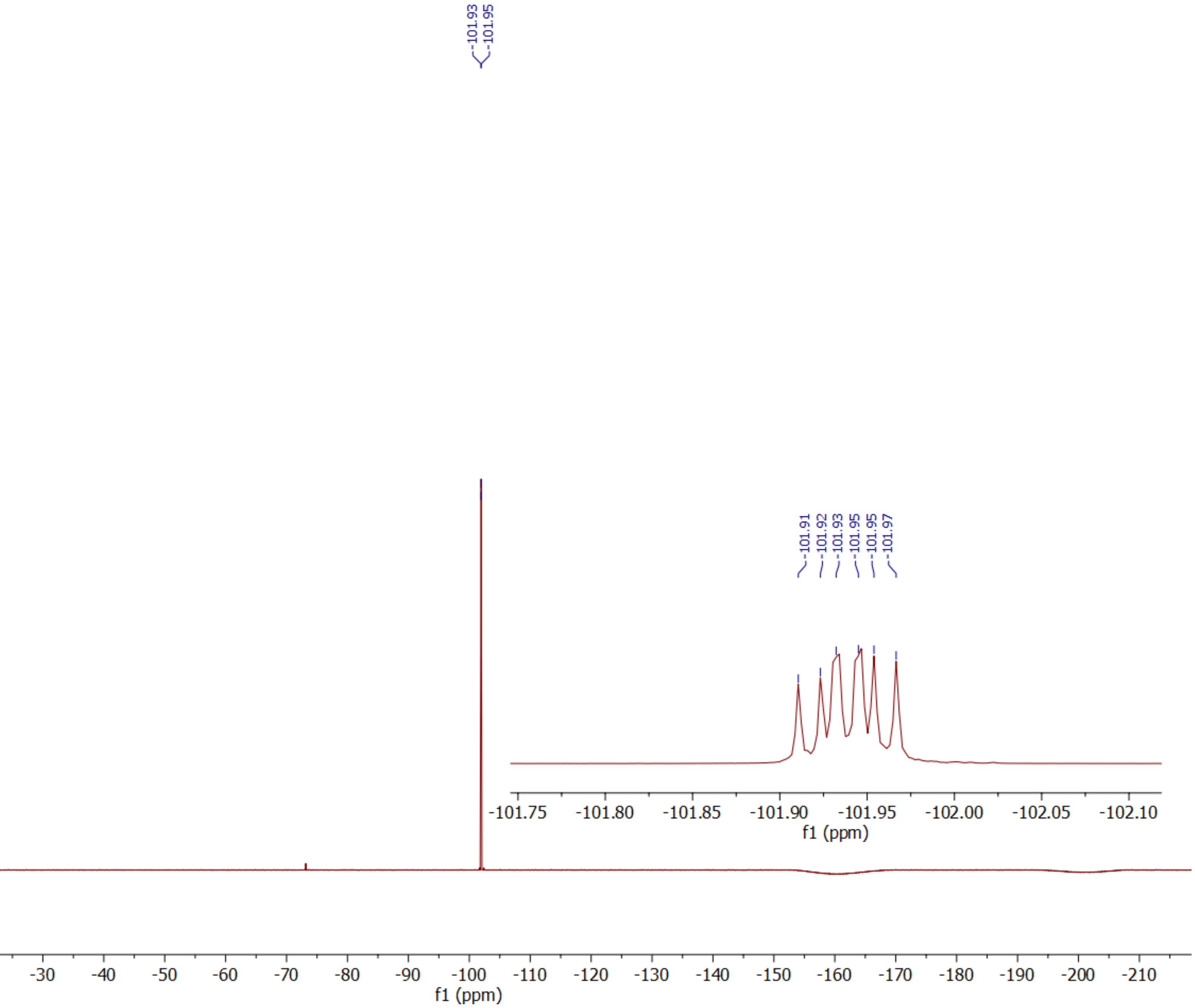

13C (100.63 MHz, CDCl3)

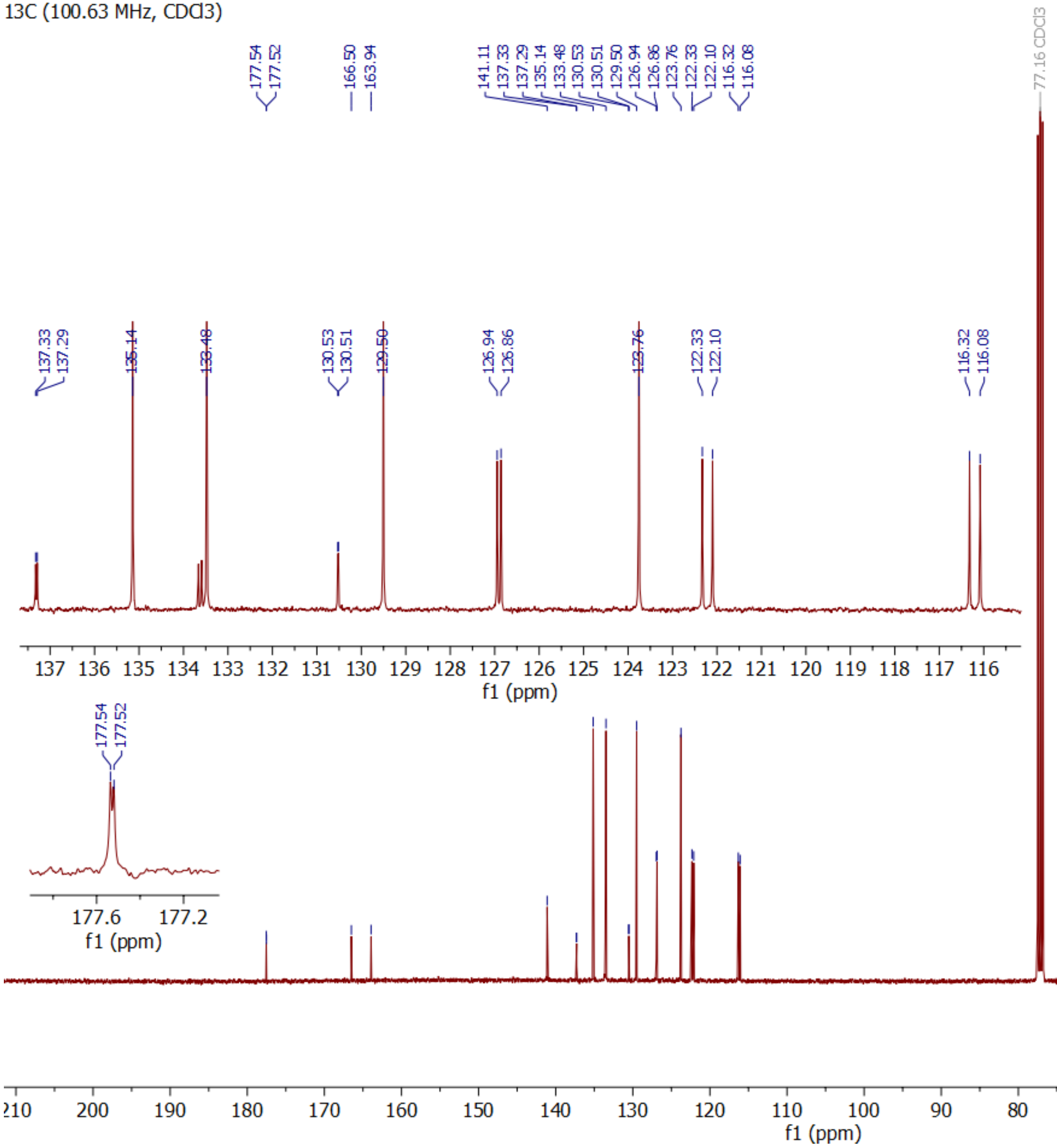


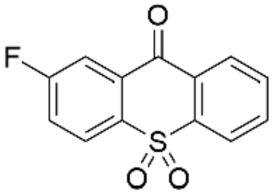

**2l**

1H (400.15 MHz, CDCl3)

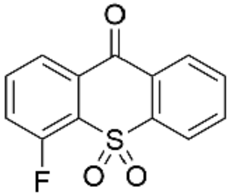

**2m**

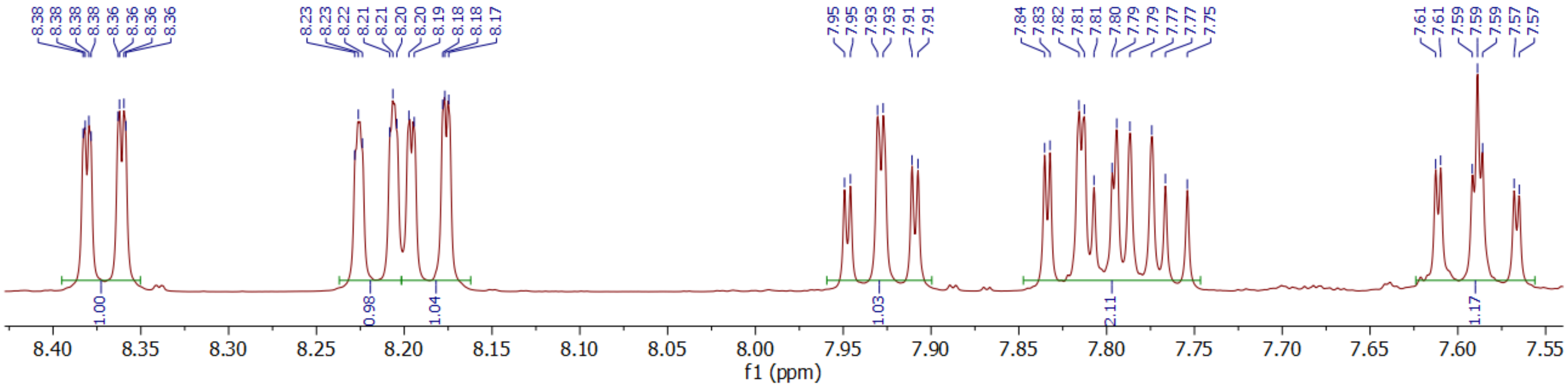

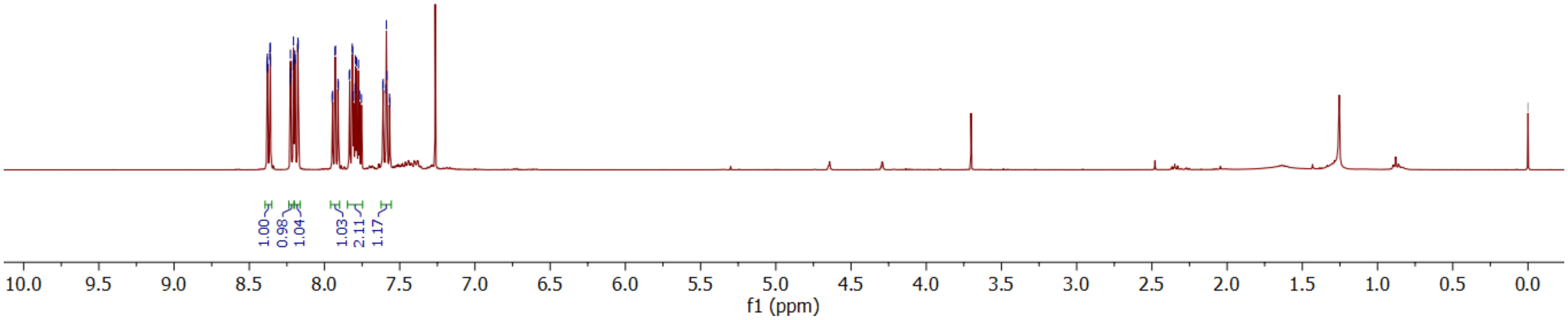

19F (376.48 MHz, CDCl3)

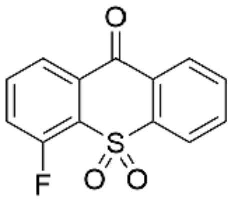

**2m**

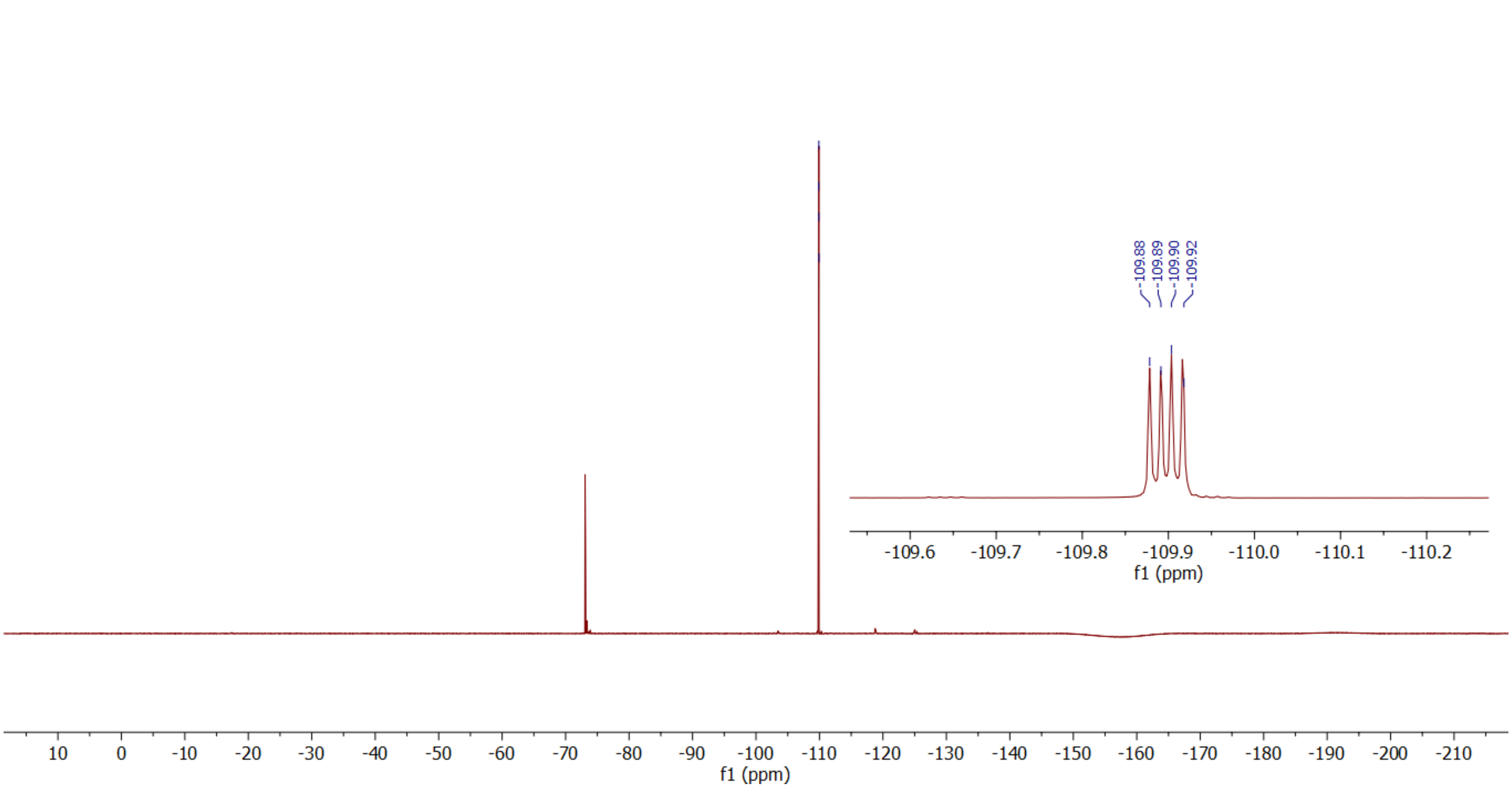

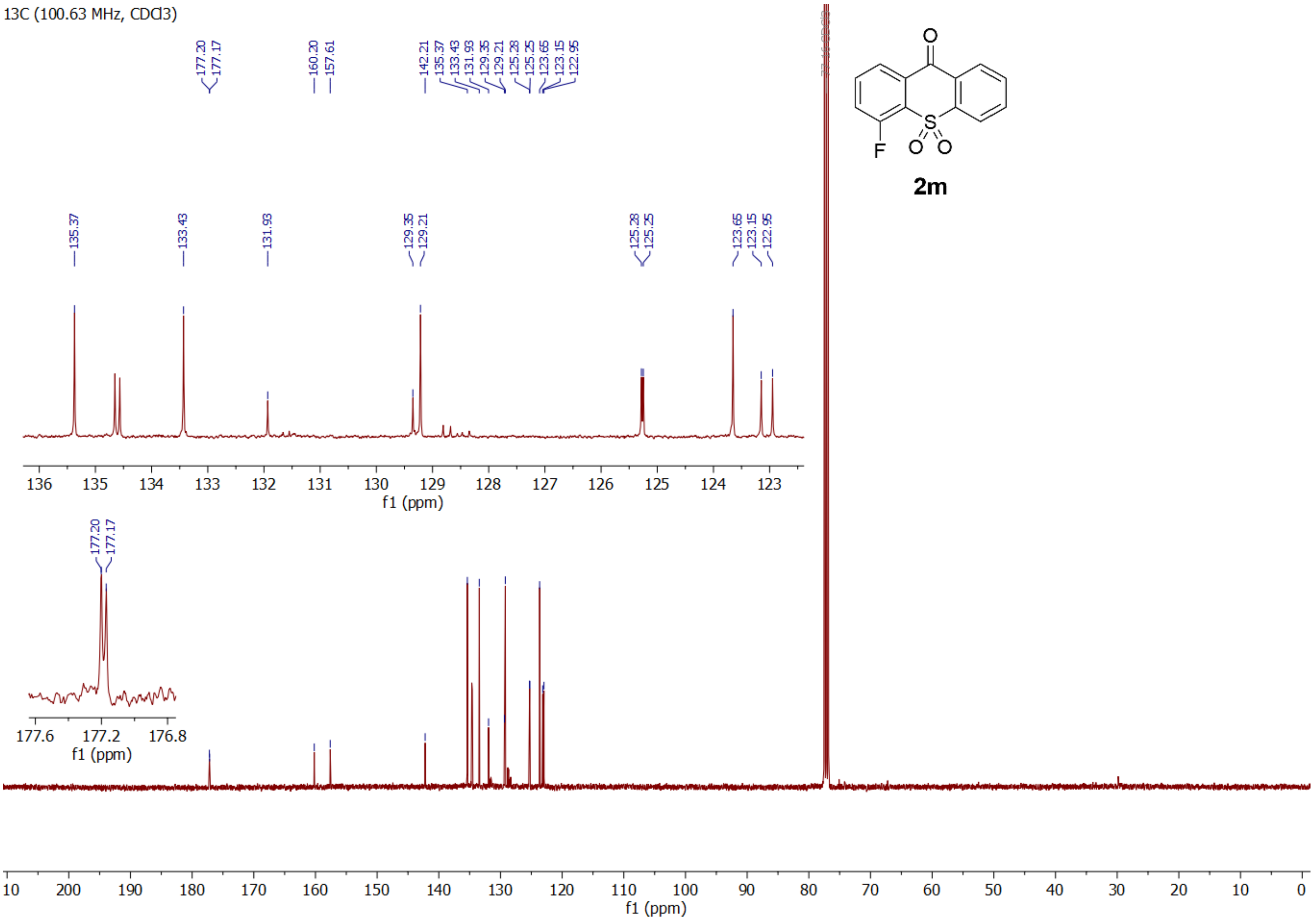
13C (100.63 MHz, CDCl3)
177.20
177.17
160.20
157.61
142.21
135.37
133.43
131.93
129.35
129.21
125.28
125.25
123.65
123.15
122.95
O
S
O
O
F
2m
f1 (ppm)

1H (400.15 MHz, CDCl3)

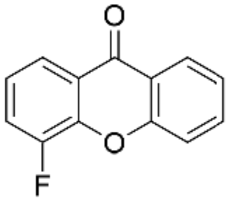

2m'

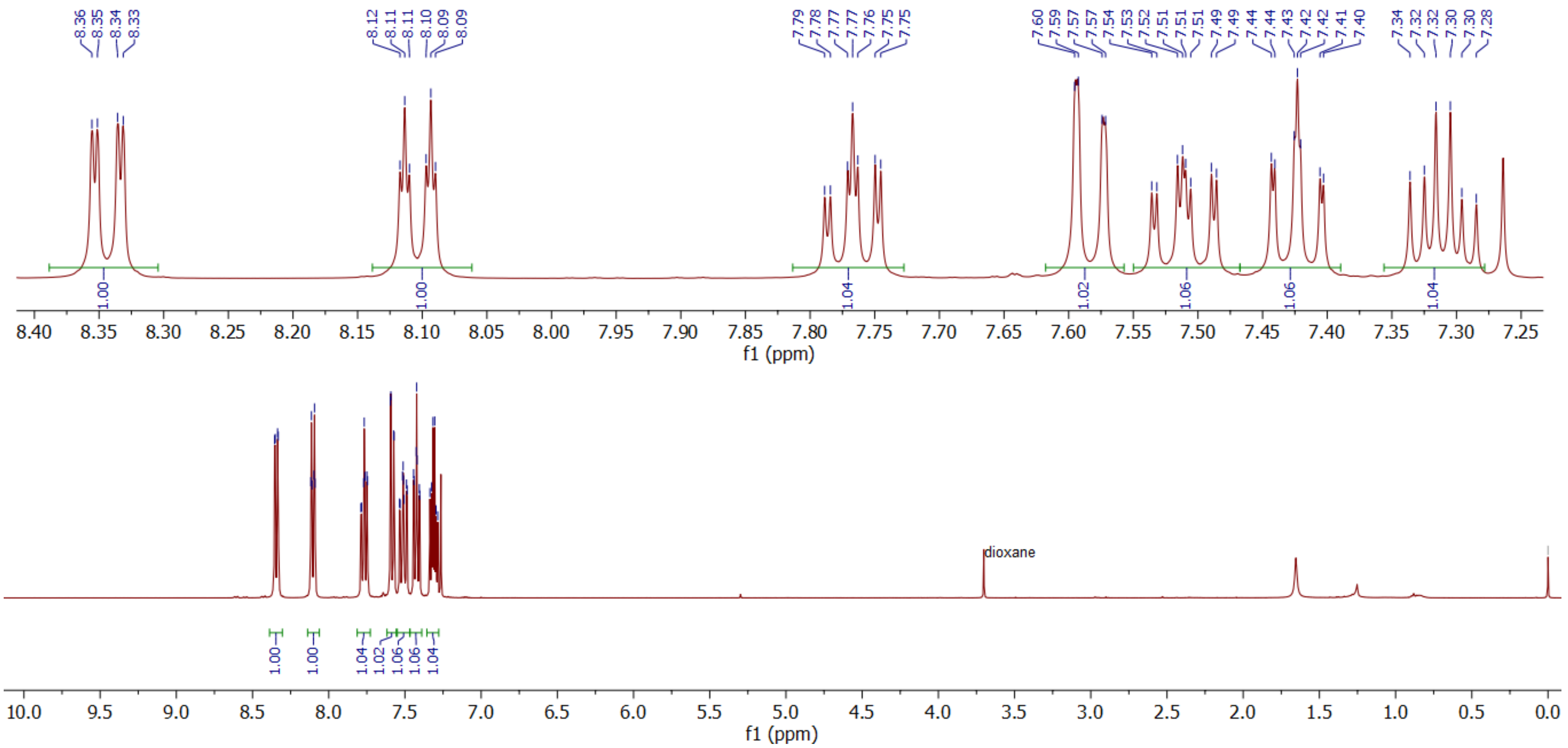

19F (376.48 MHz, CDCl3)

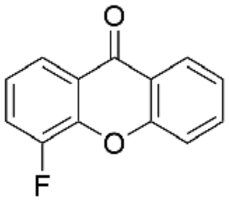

**2m'**

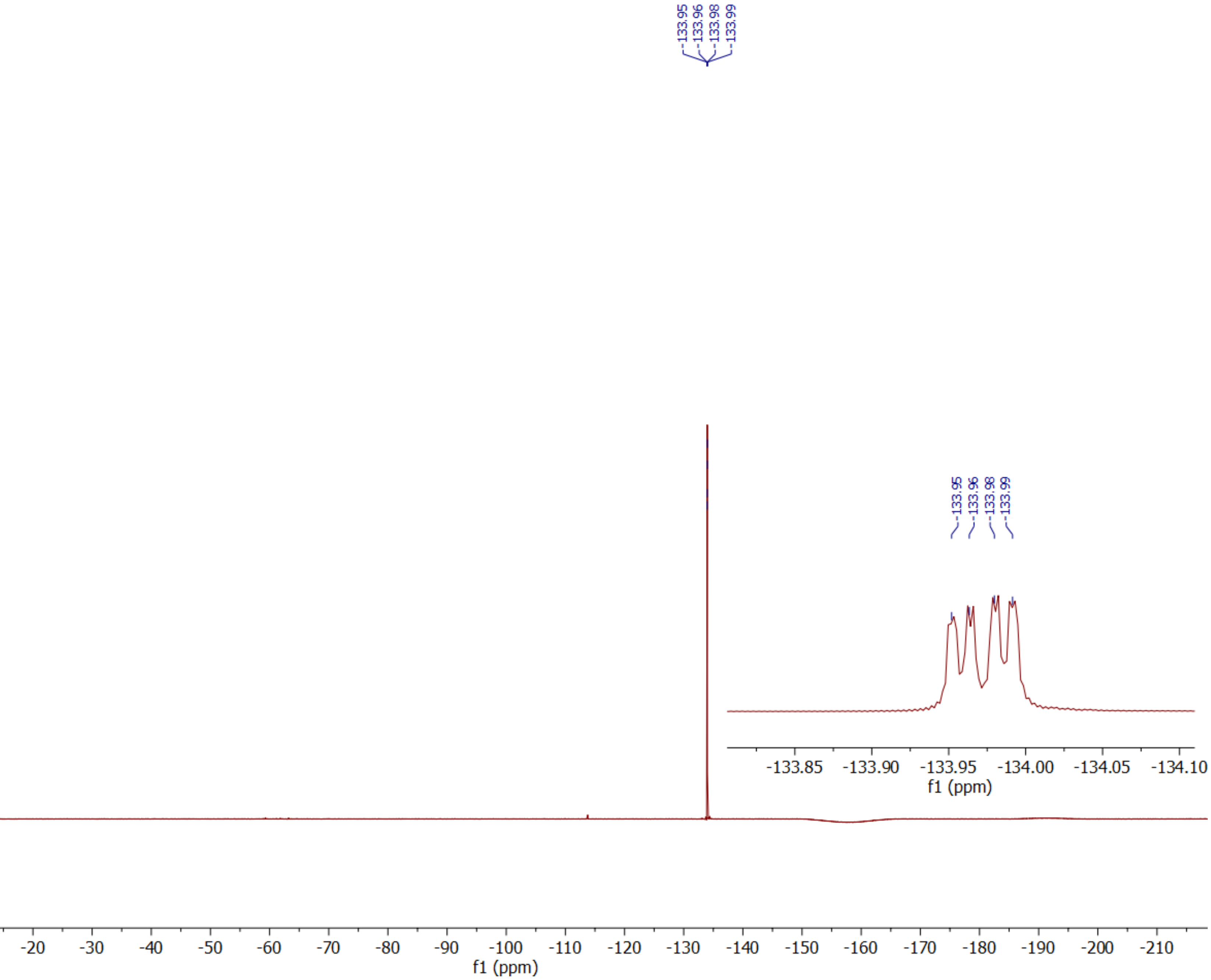

13C (100.63 MHz, CDCl3)

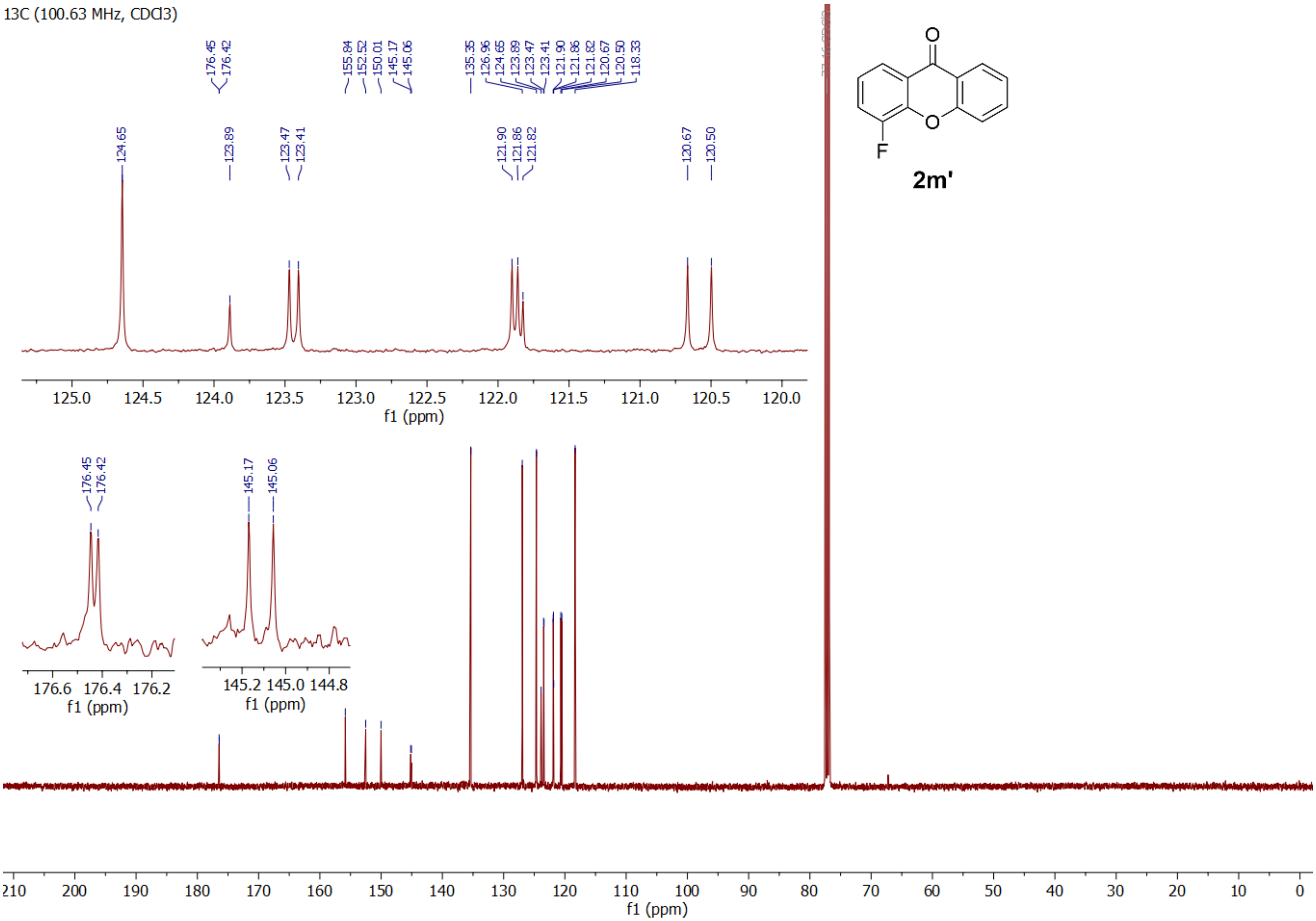


2m'

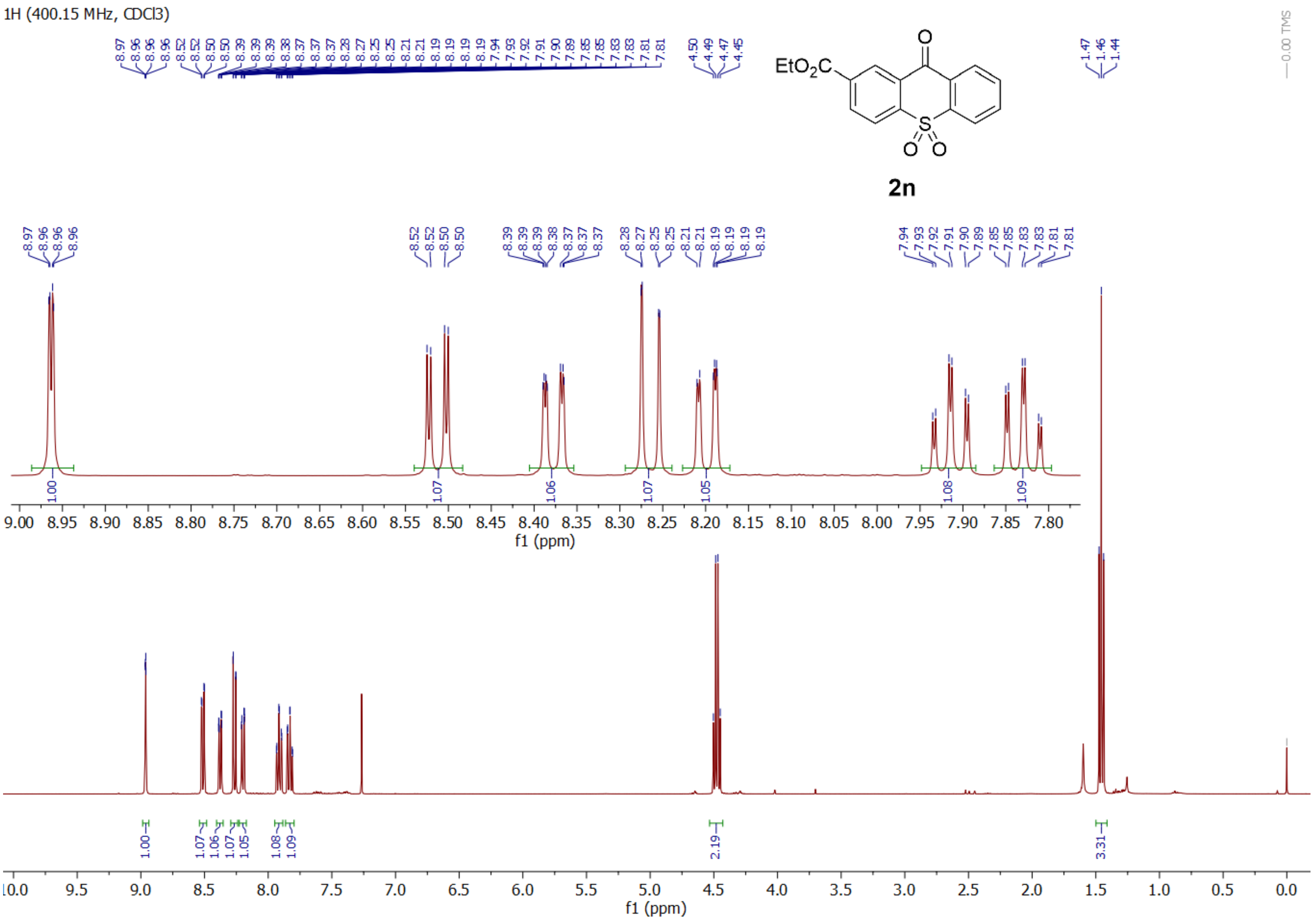
1H (400.15 MHz, CDCl3)
EtO2C
O
S
O O
2n
0.00 TMS
1.00
1.07
1.06
1.07
1.05
1.08
1.09
2.19
3.31
f1 (ppm)
f1 (ppm)

13C (100.63 MHz, CDCl3)

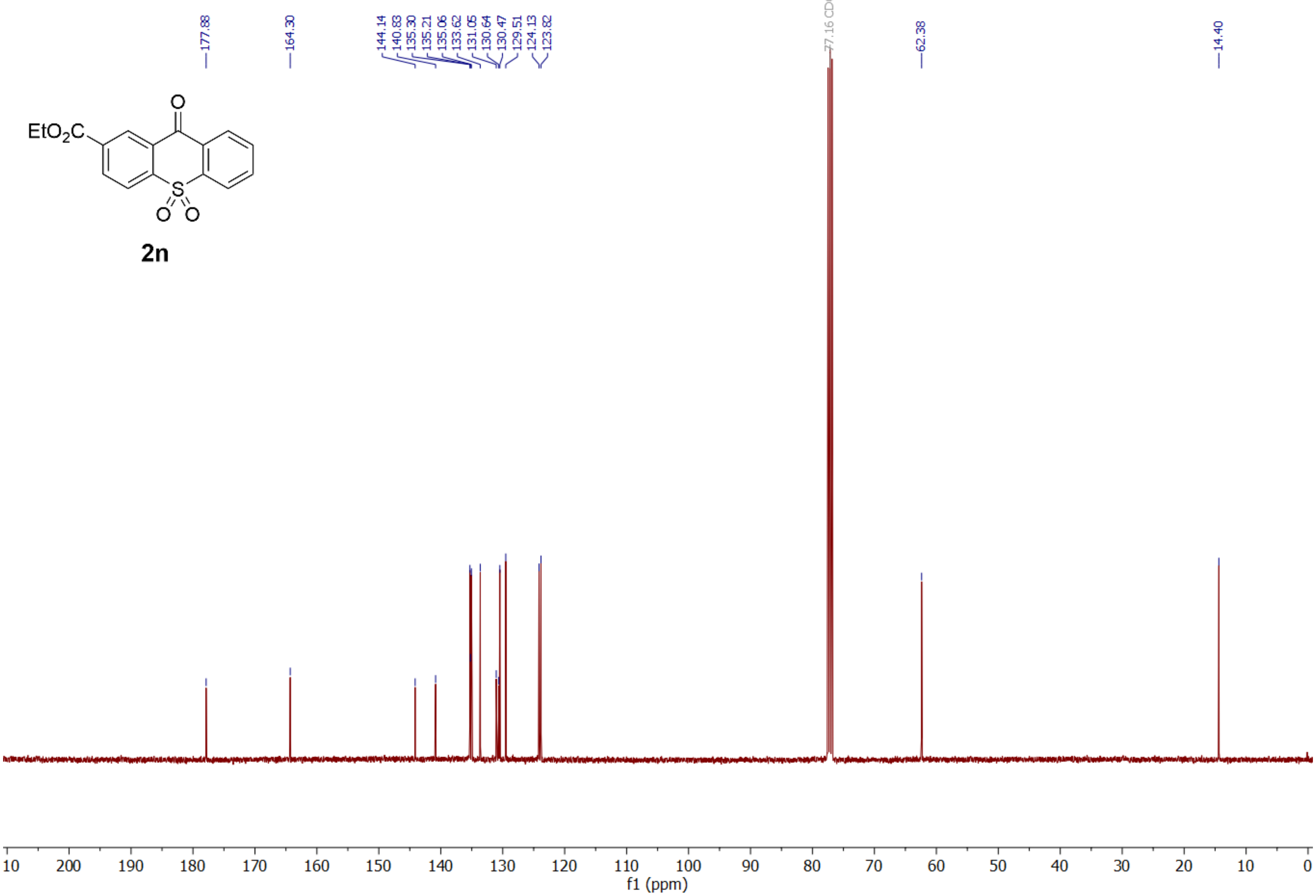


2n

1H (400.15 MHz, CDCl3 + 10% (v/v) DMSO-d6)

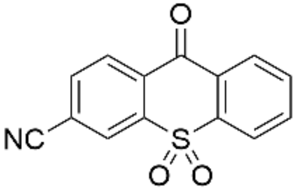

**2o**

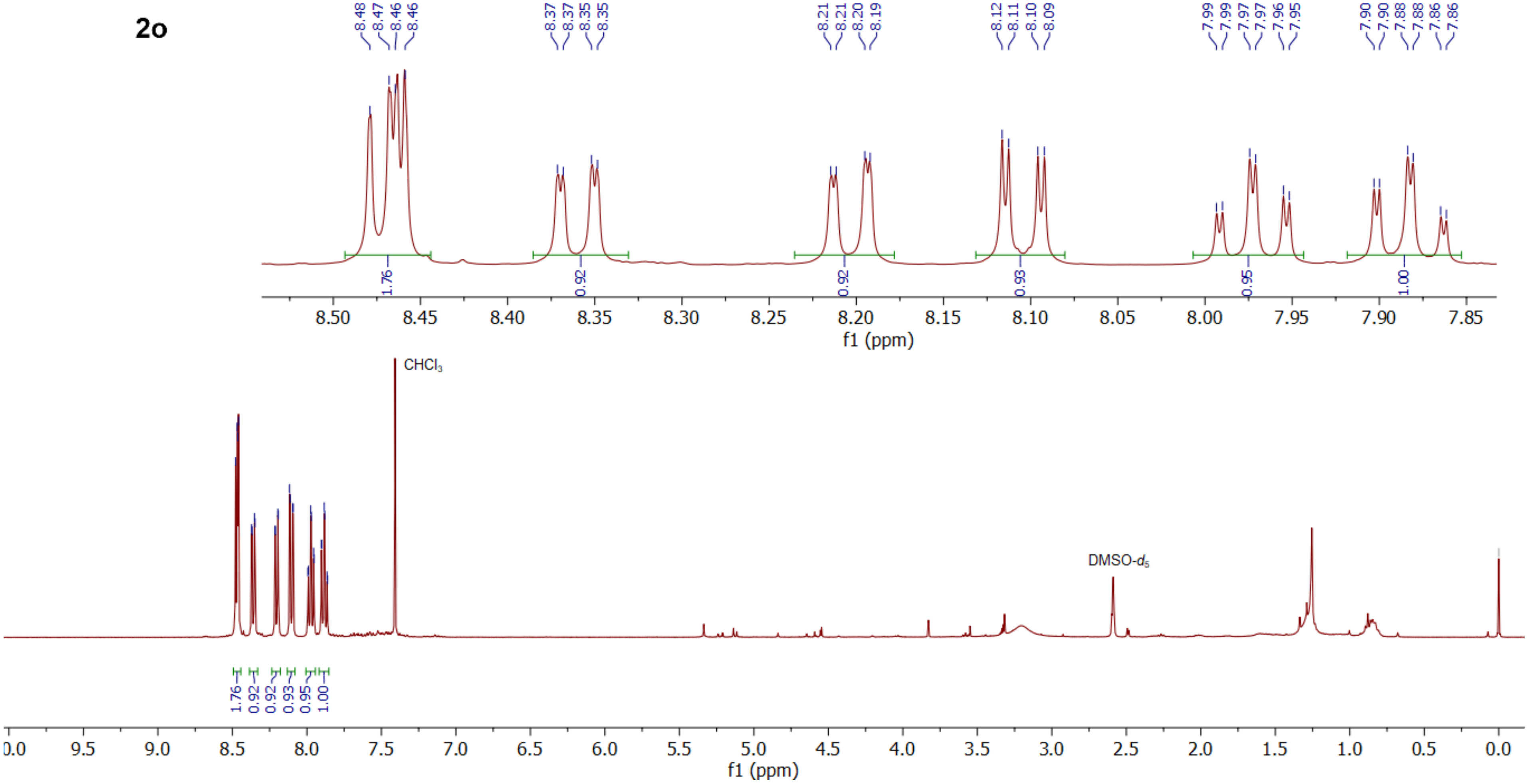

13C (100.63 MHz, CDCl3 + 10% (v/v) DMSO-d6)

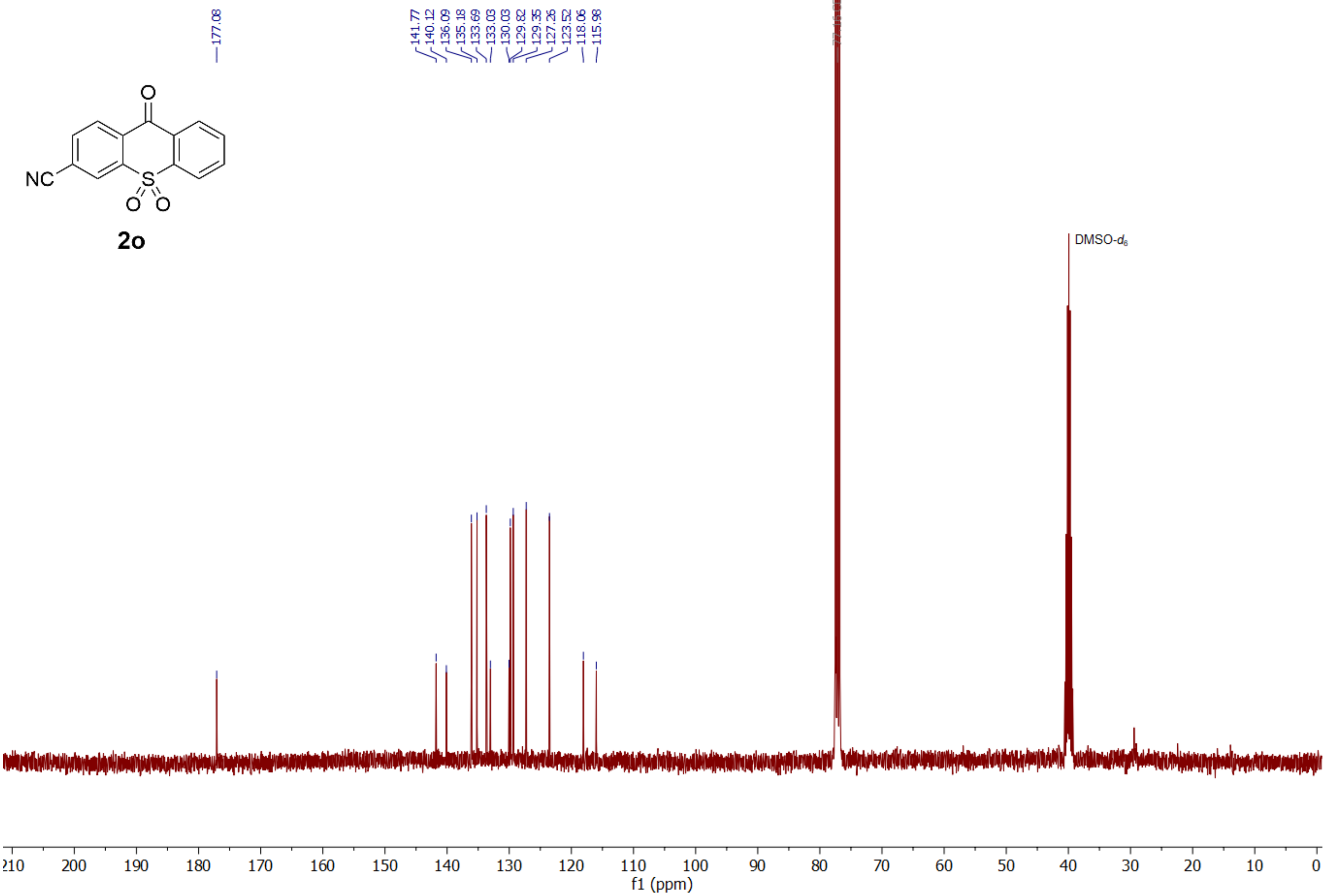

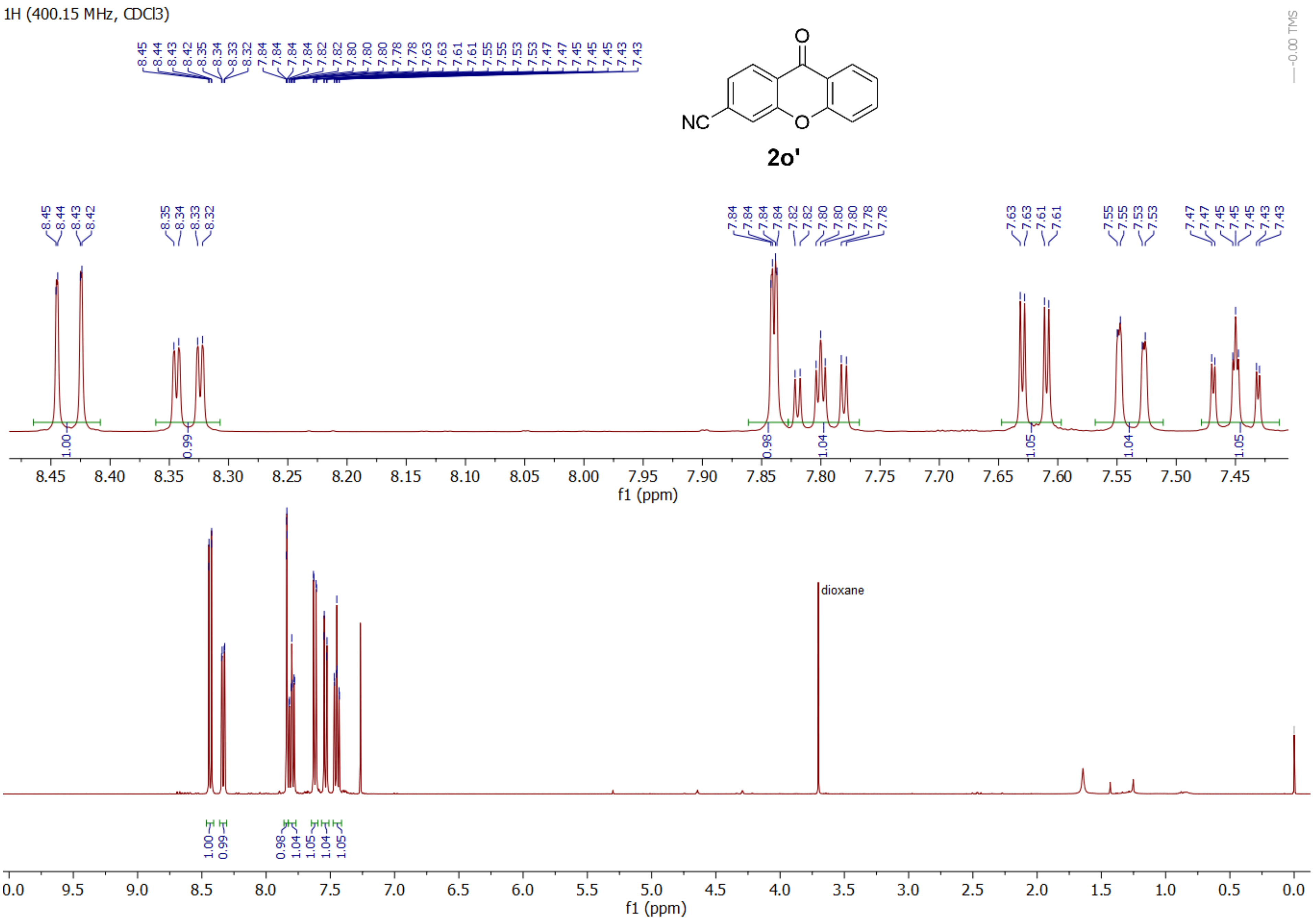
1H (400.15 MHz, CDCl3)
NC
O
2o'
-0.00 TMS
dioxane
f1 (ppm)
f1 (ppm)

13C (100.63 MHz, CDCl3)

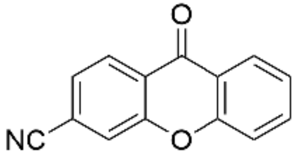

**2o'**

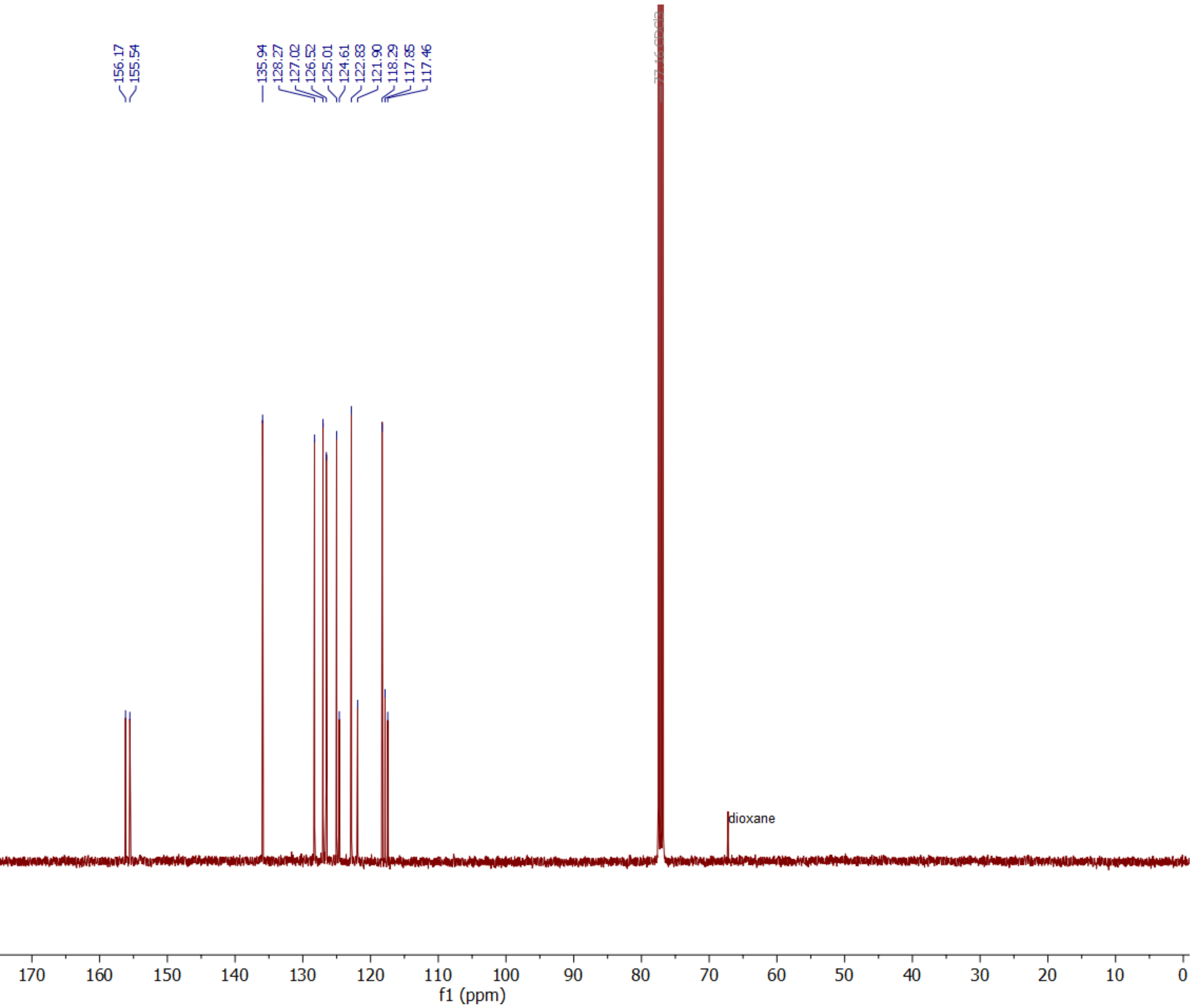

1H (400.15 MHz, CDCl3)

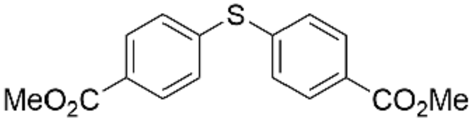

**2p**

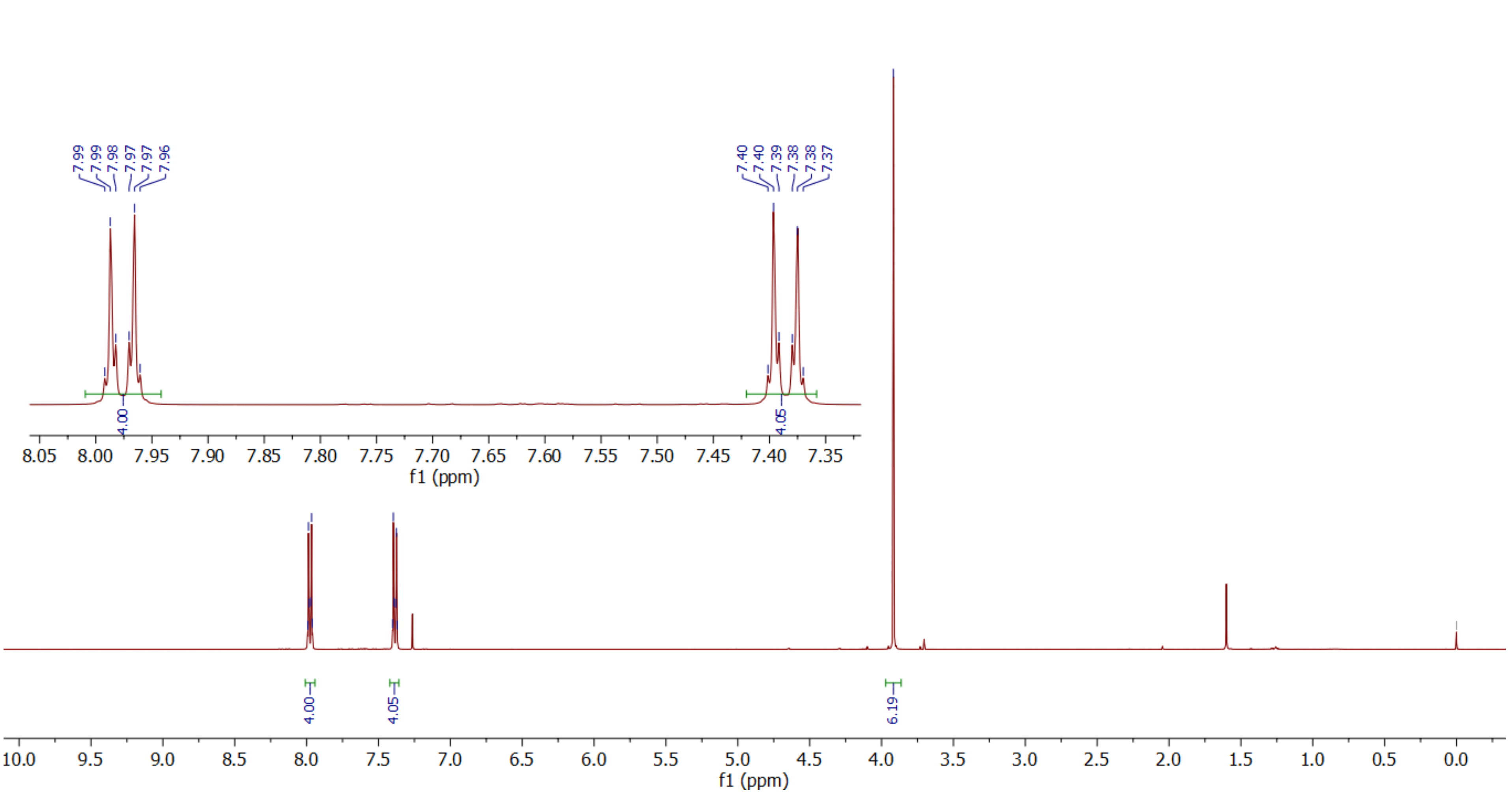

13C (100.63 MHz, CDCl3)

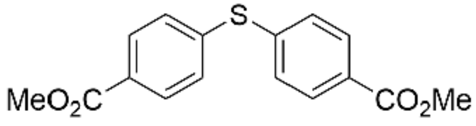


**2p**

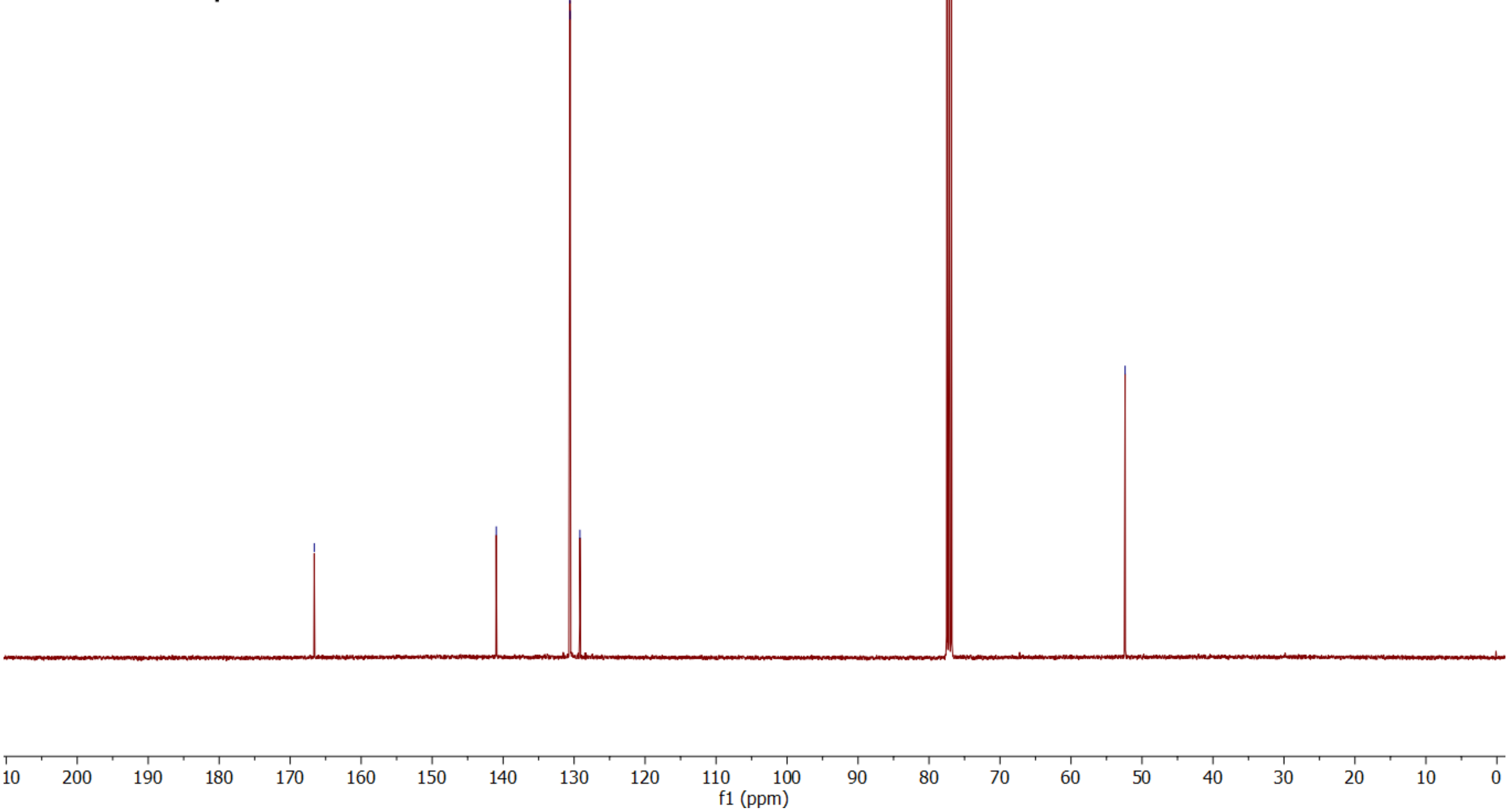

1H (400.15 MHz, CDCl3)

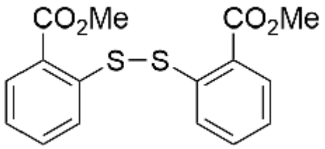


**2q**

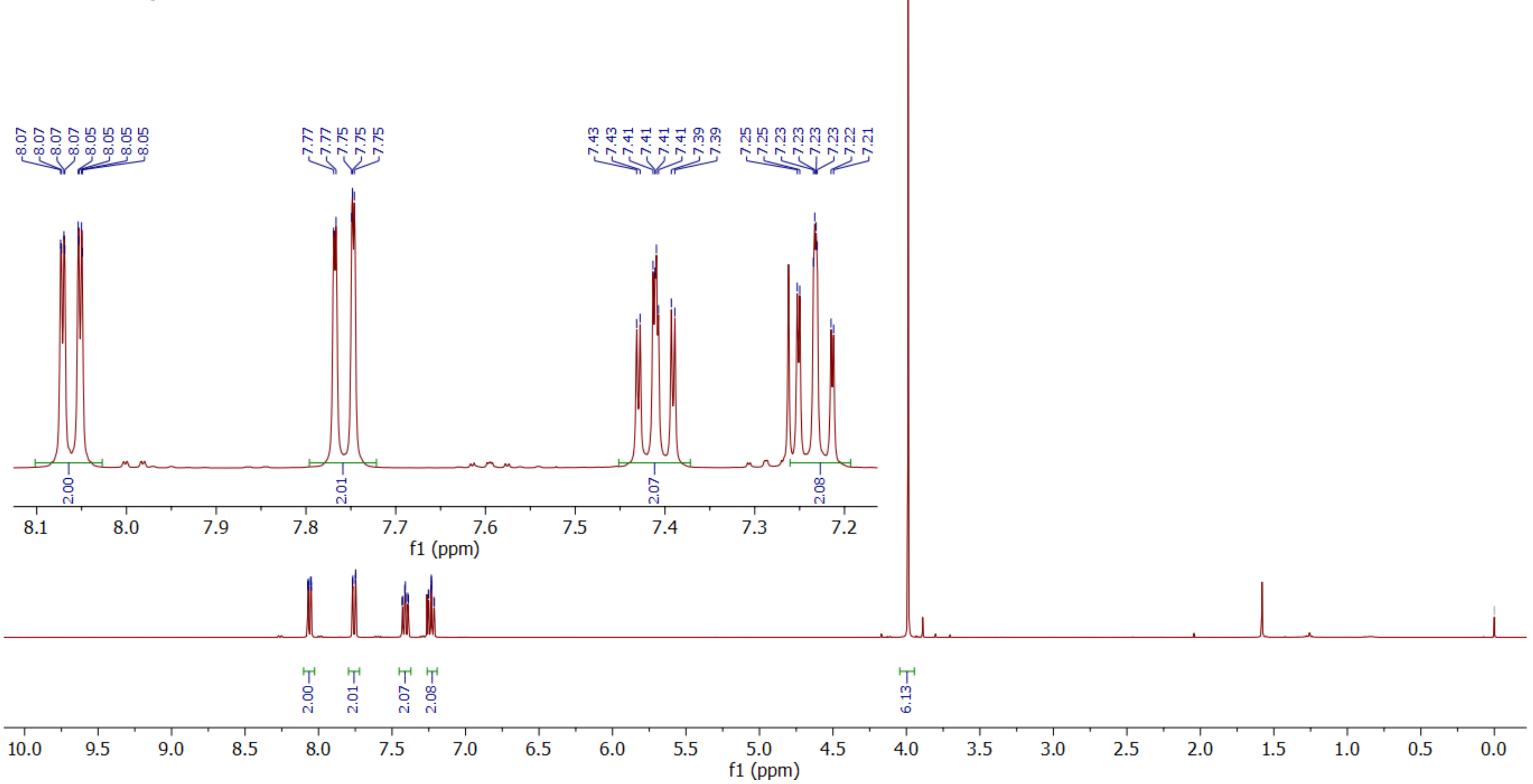

13C (100.63 MHz, CDCl3)

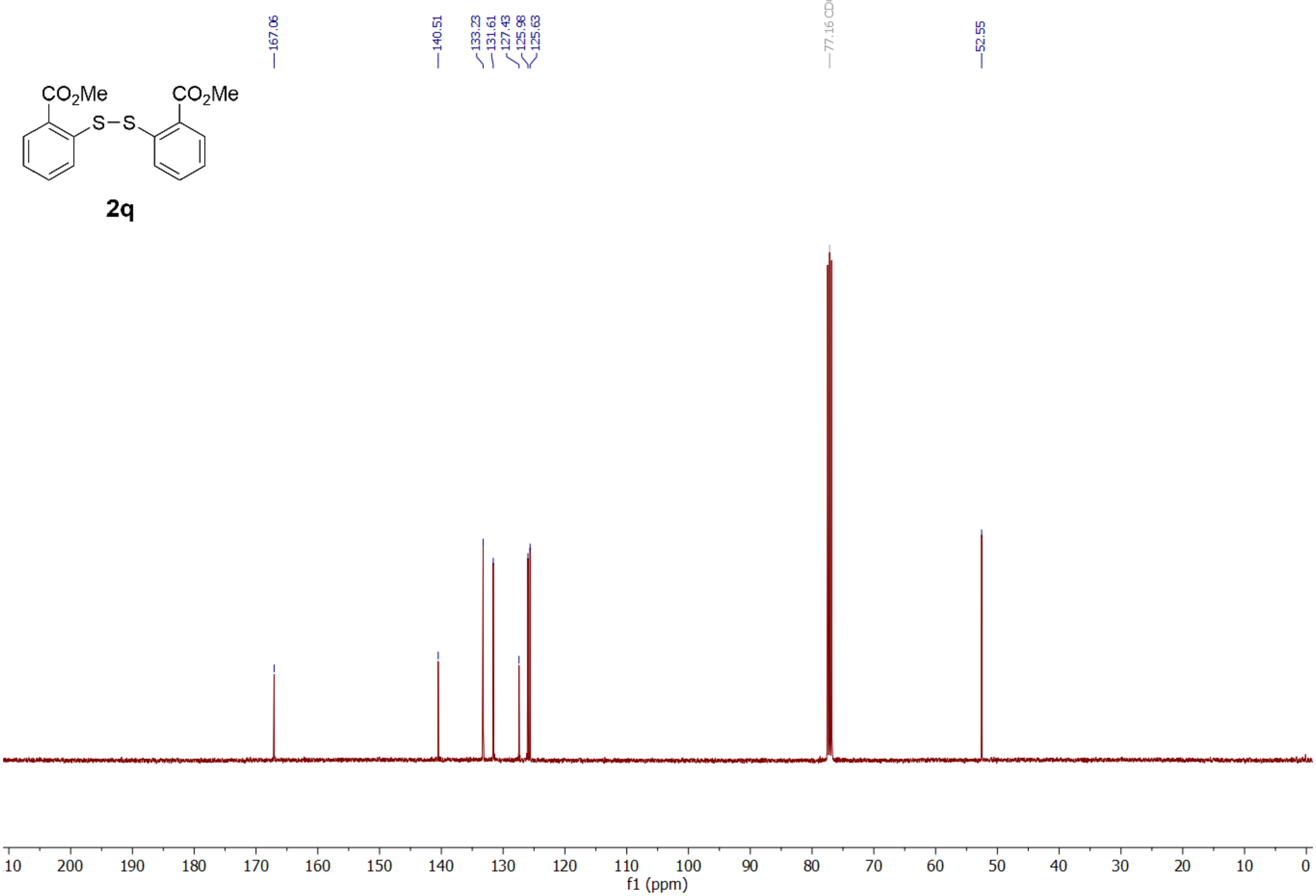

1H (400.15 MHz, CDCl3)

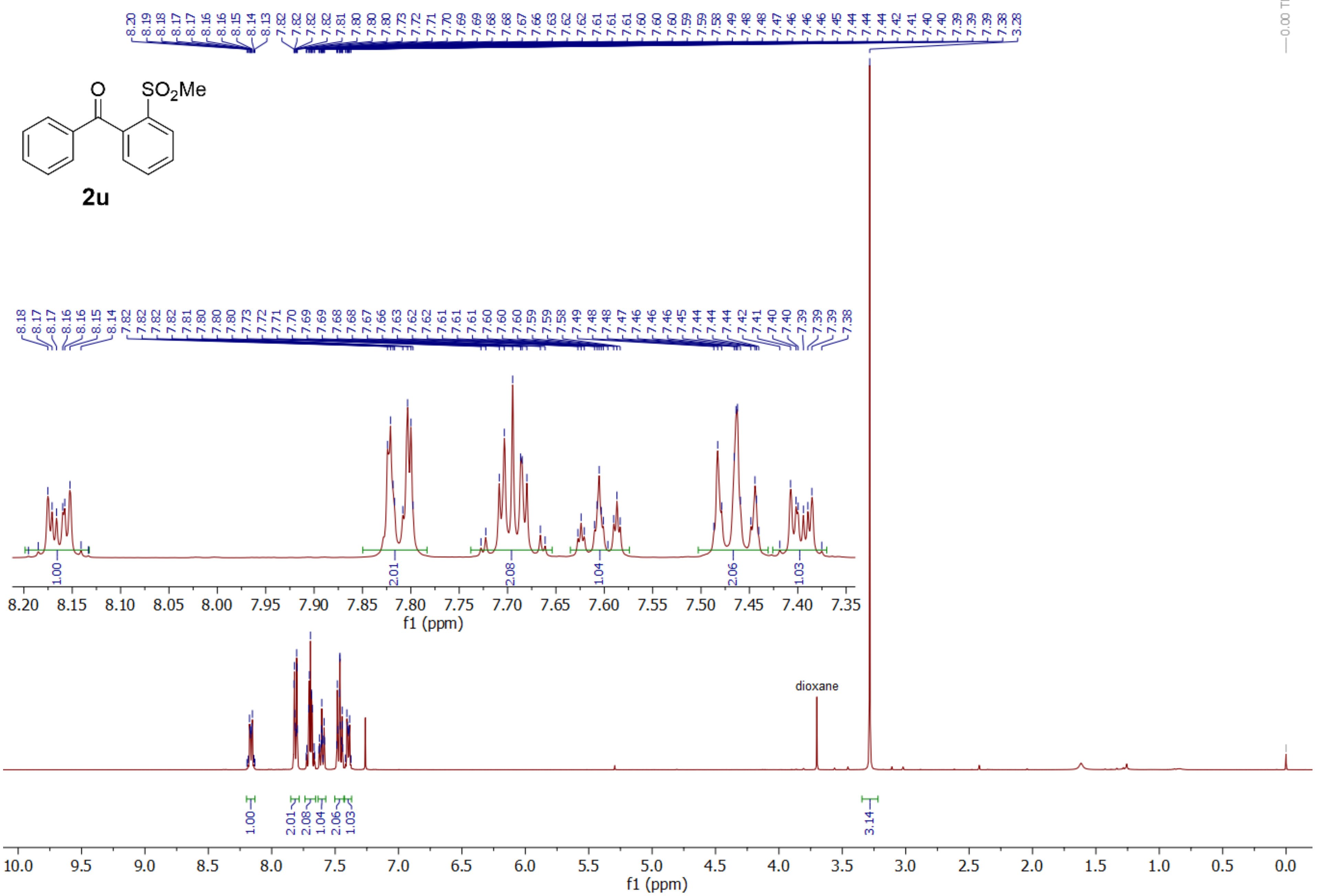

13C (100.63 MHz, CDCl3)

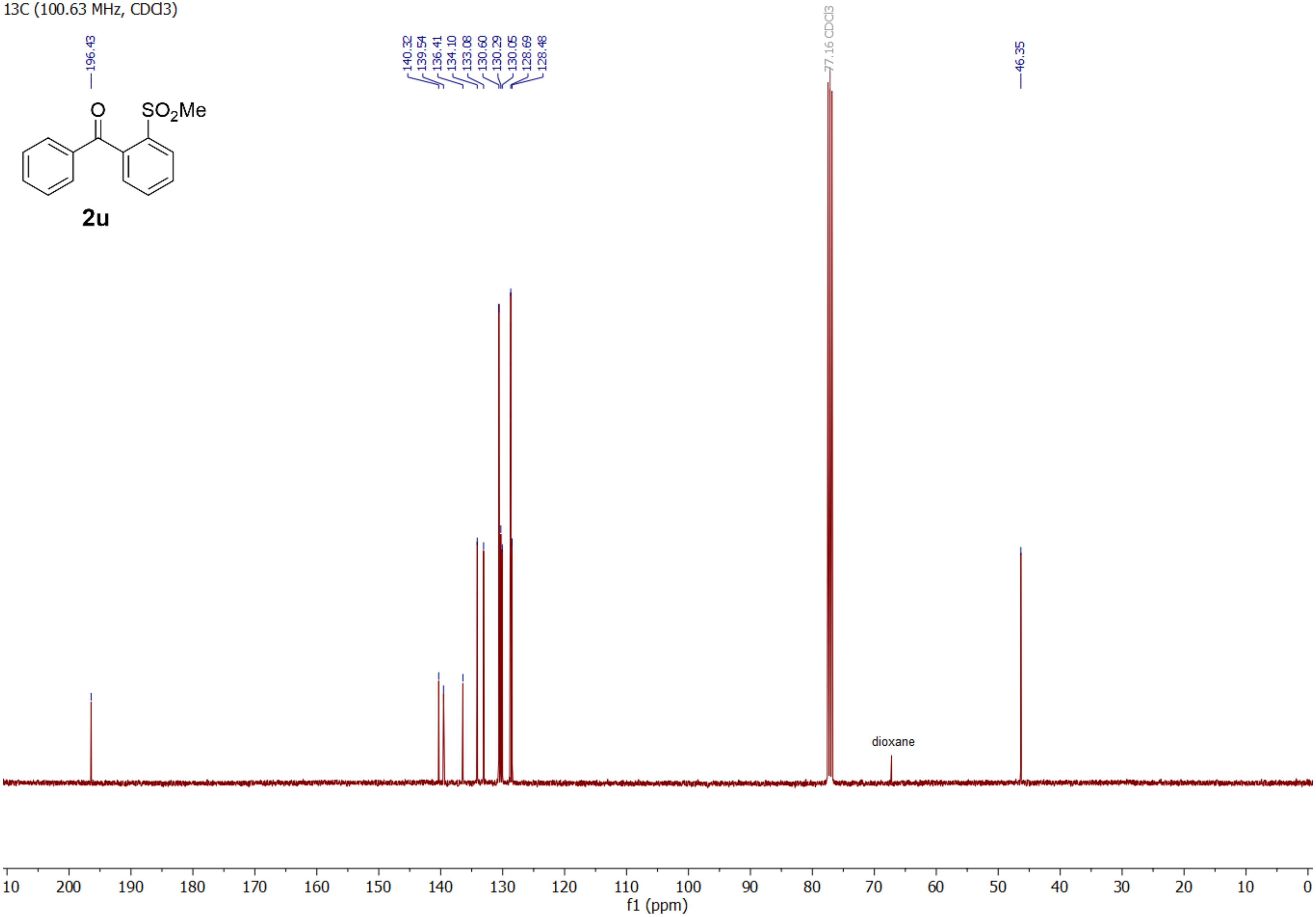

1H (400.15 MHz, DMSO)

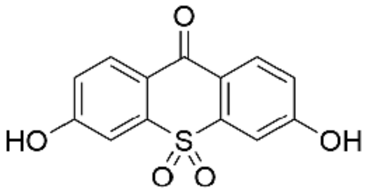

**3**

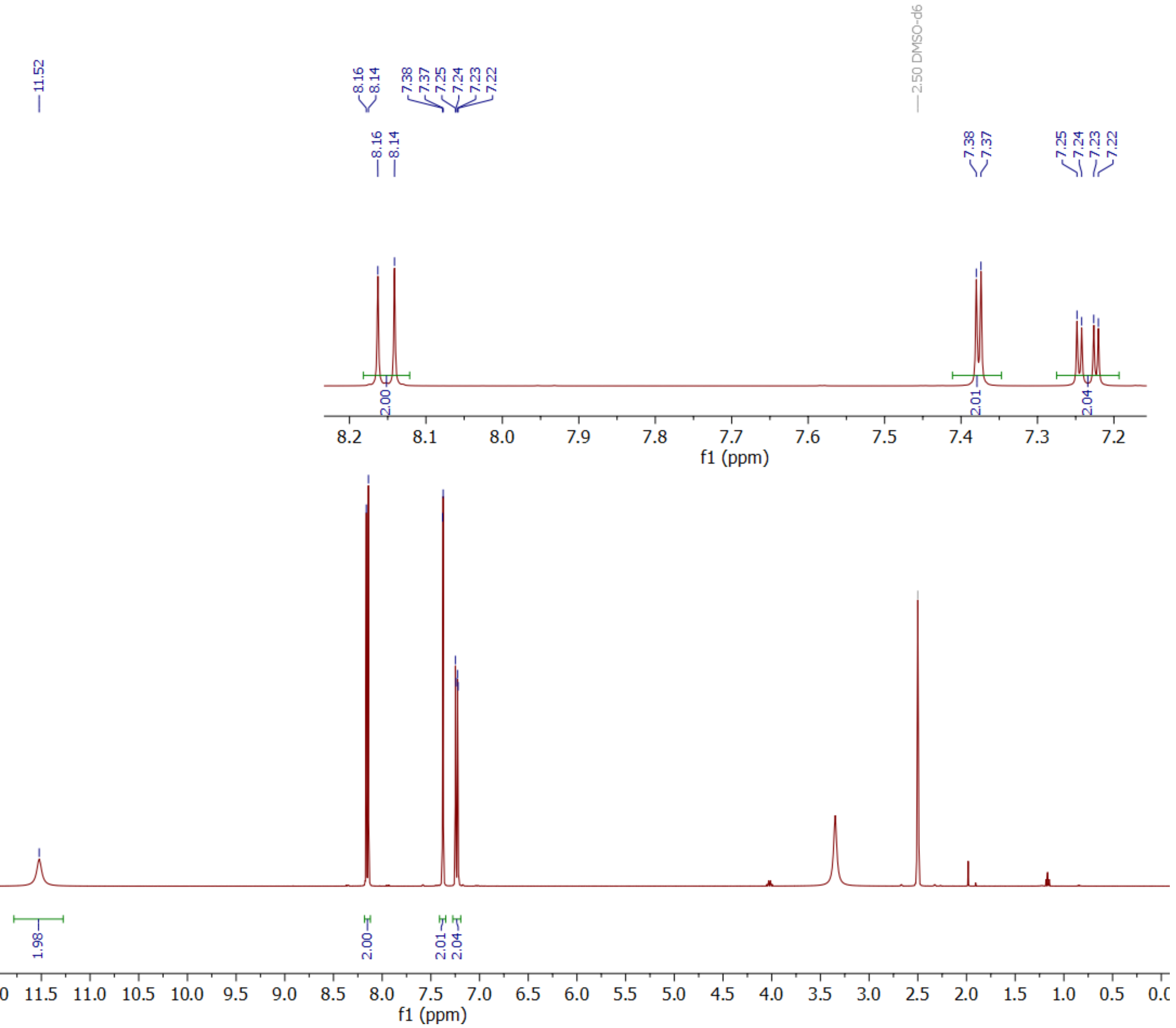

13C (100.63 MHz, DMSO)

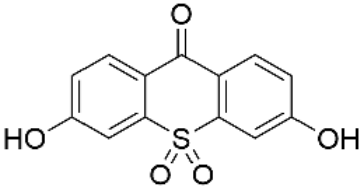


3

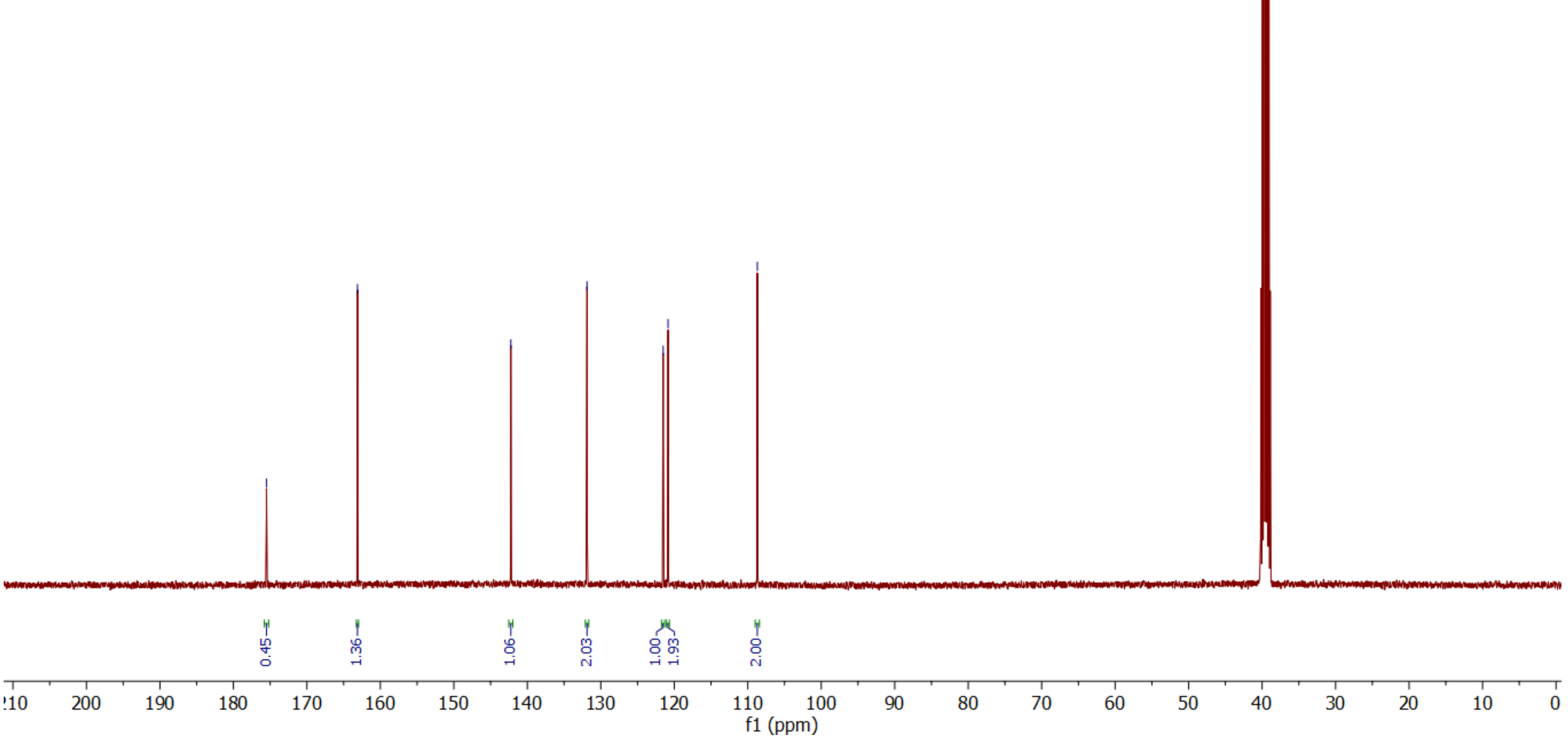

1H (400.15 MHz, CDCl3)

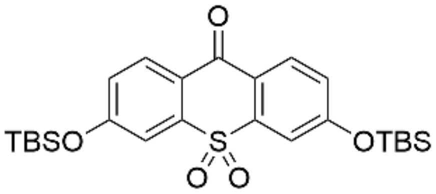


**4a**

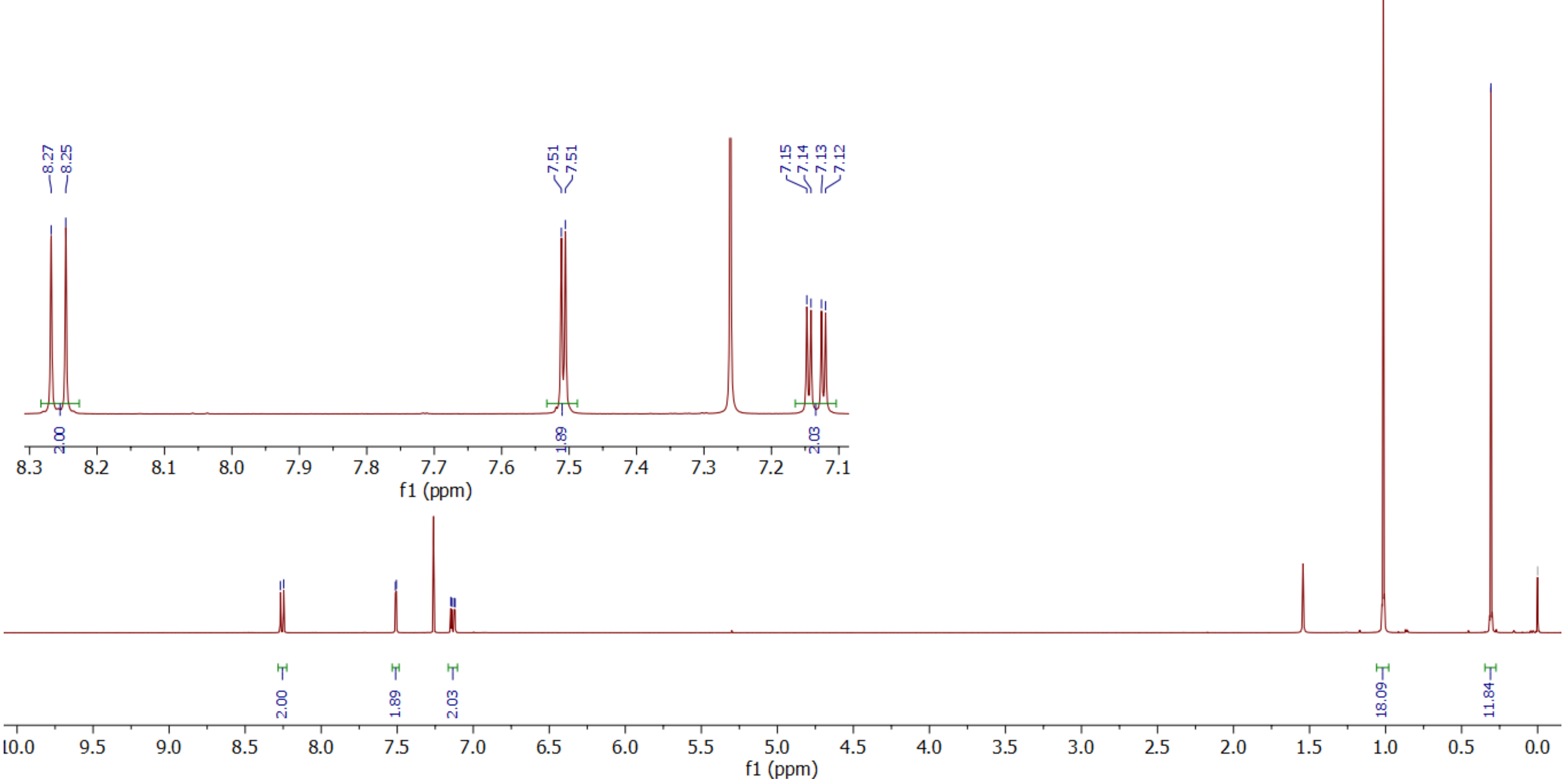

13C (100.63 MHz, CDCl3)

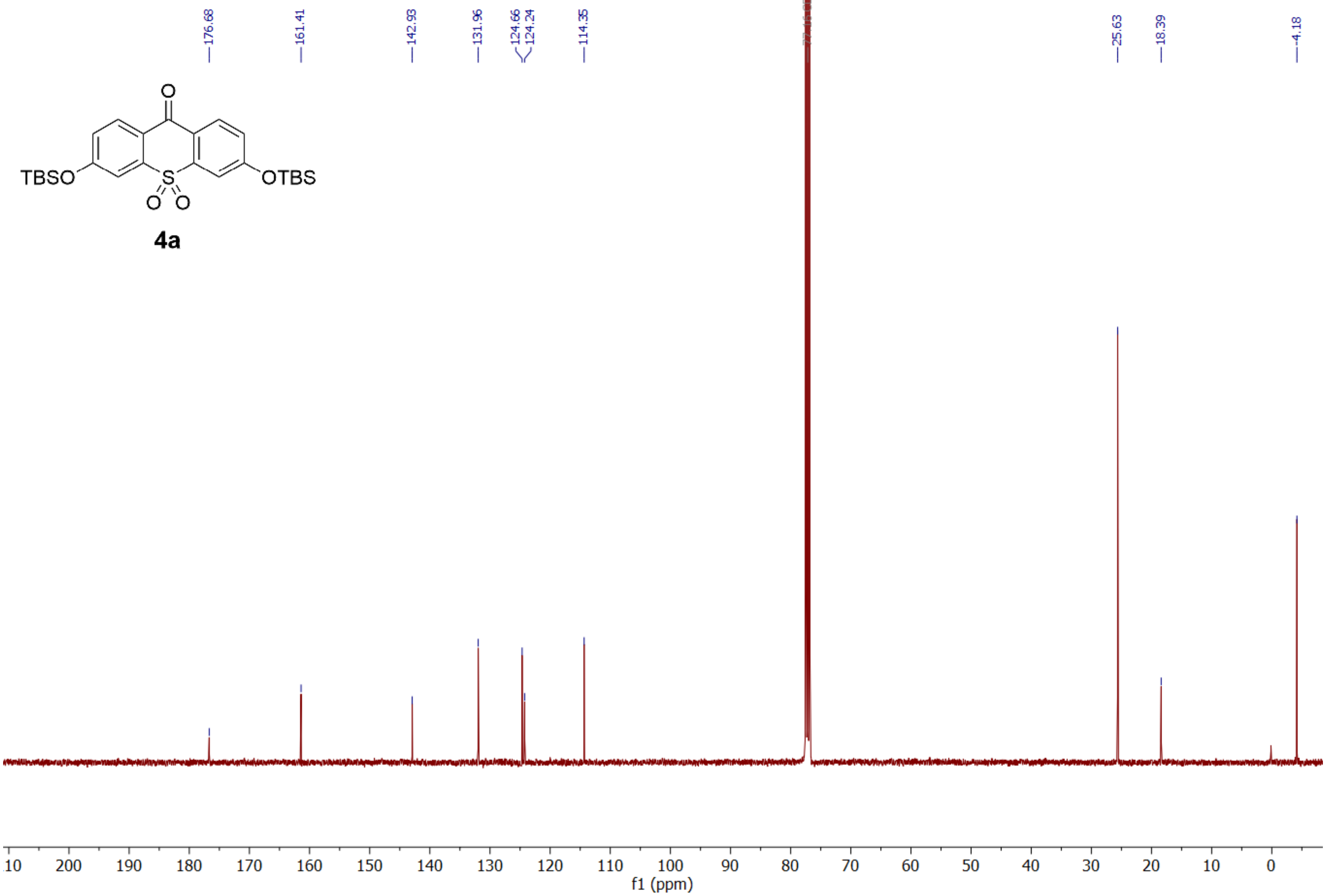

1H (400.15 MHz, CDCl3)

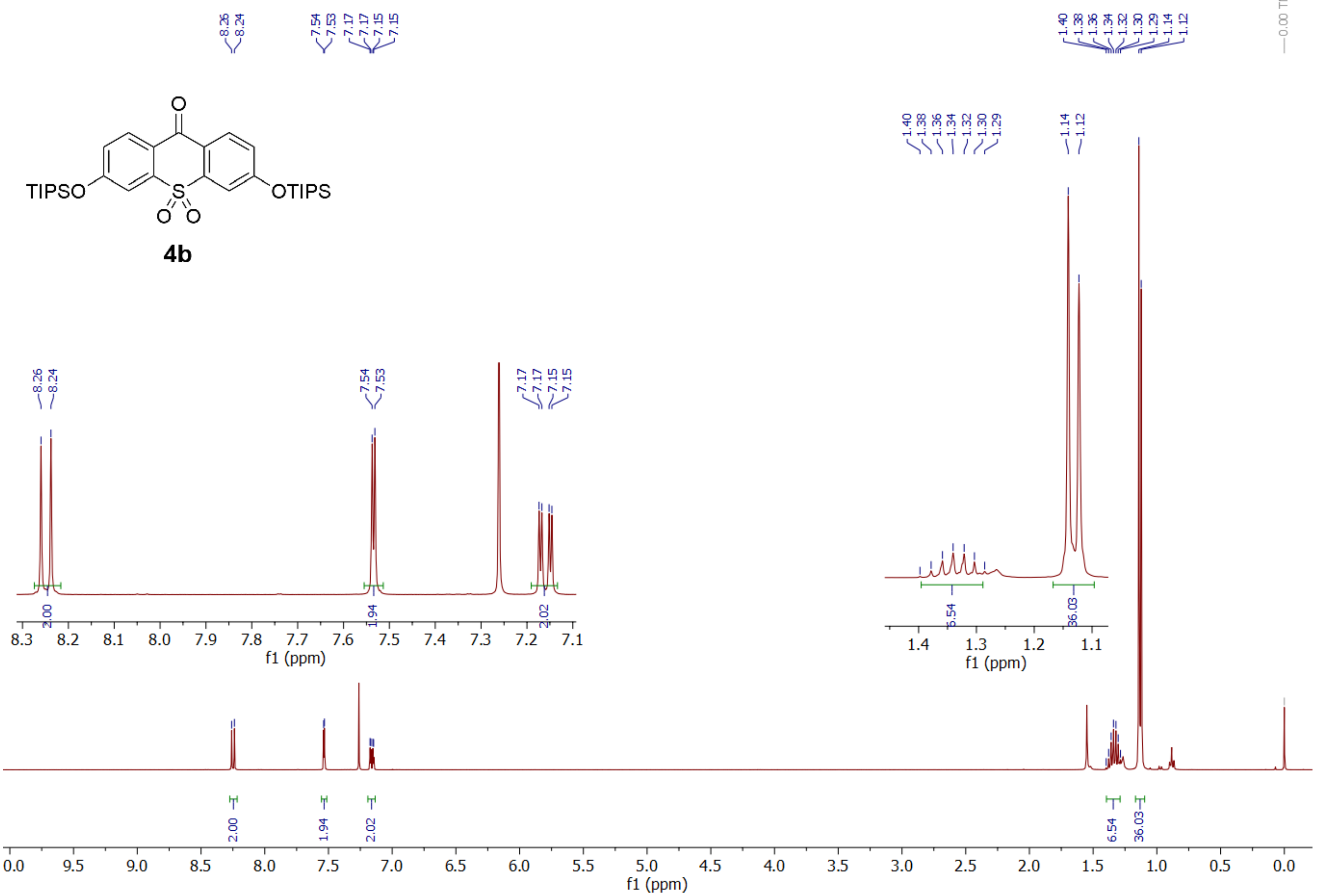

13C (100.63 MHz, CDCl3)

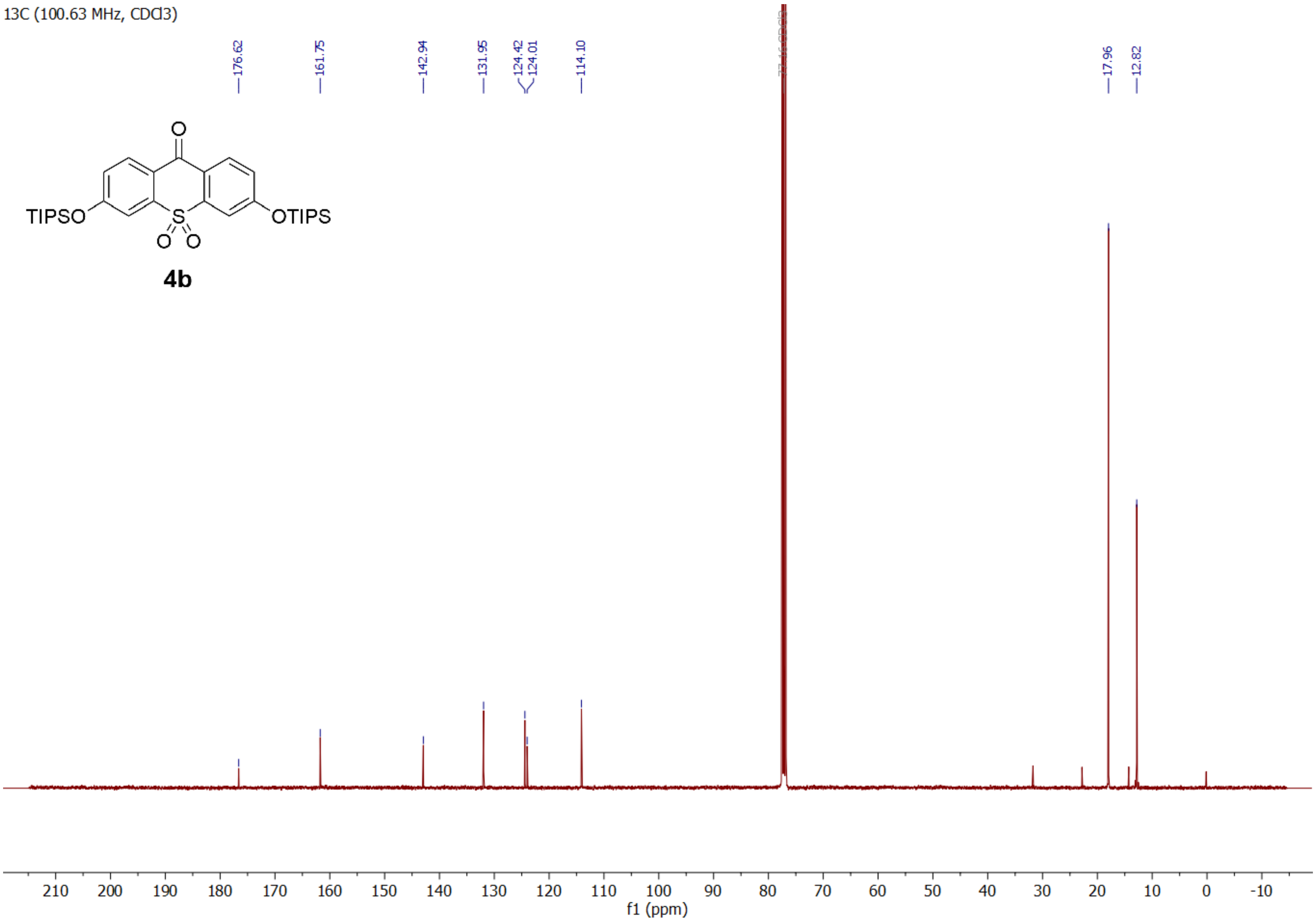

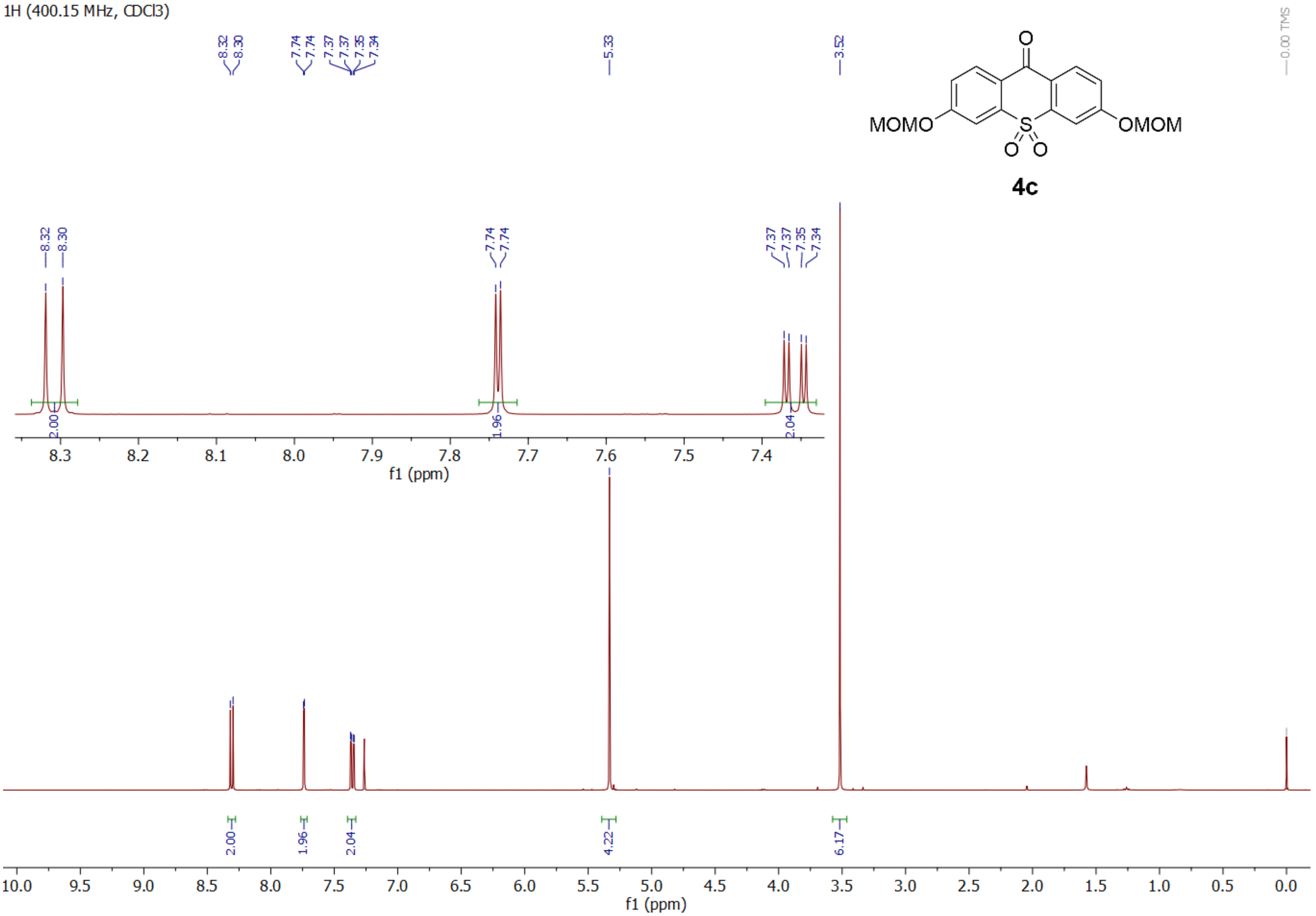

1H (400.15 MHz, CDCl3)
8.32
8.30
7.74
7.74
7.37
7.37
7.35
7.34
5.33
3.52
0.00 TMS
MOMO
OMOM
O
S
2.00
1.96
2.04
4.22
6.17
f1 (ppm)

4c

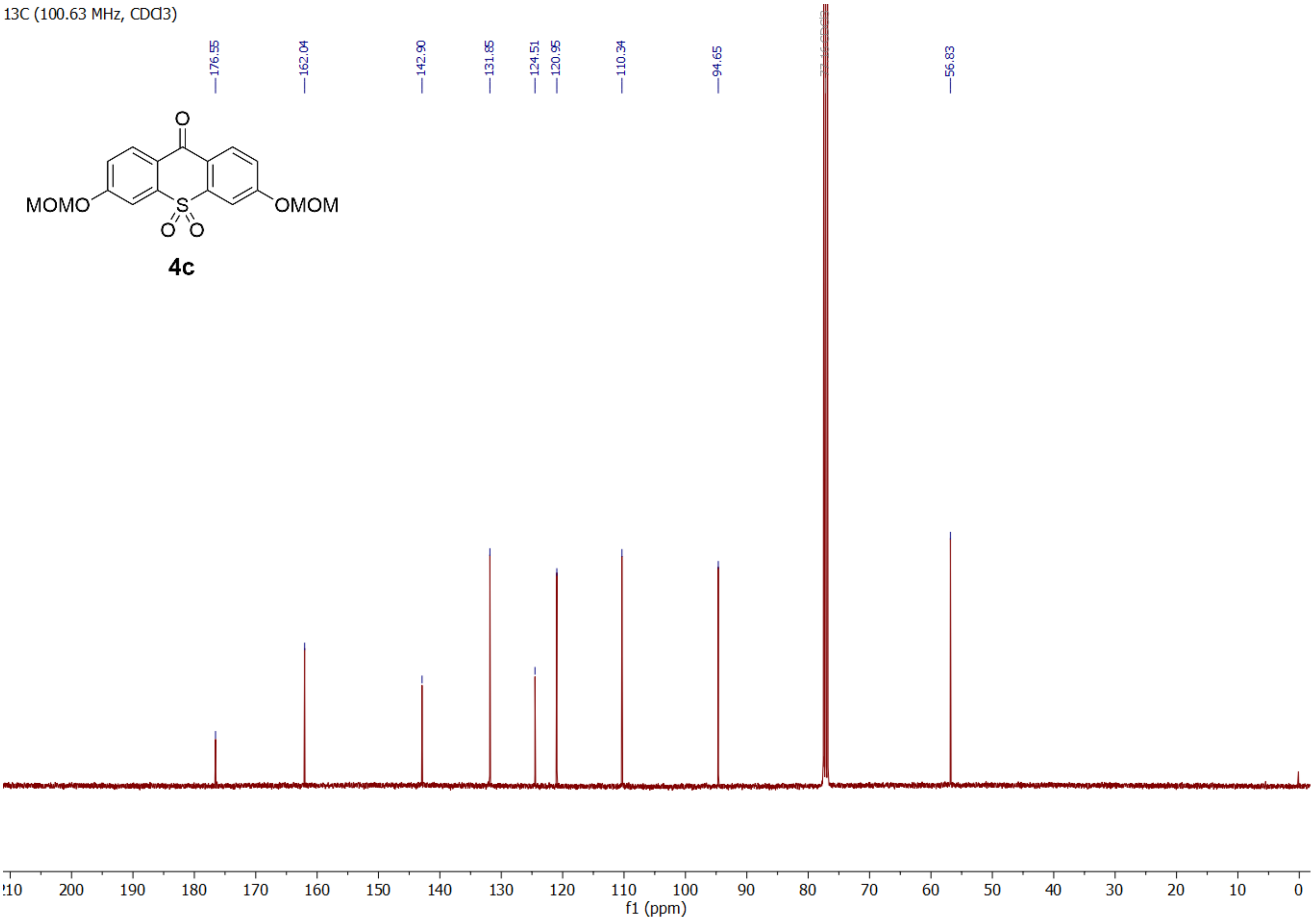
13C (100.63 MHz, CDCl3)
176.55
162.04
142.90
131.85
124.51
120.95
110.34
94.65
56.83
MOMO
OMOM
O
S
4c
f1 (ppm)

1H (400.15 MHz, CDCl3)

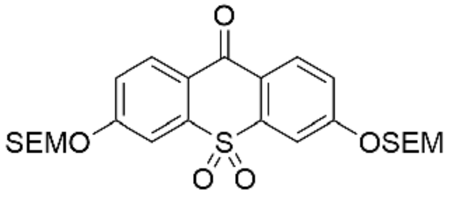


**4d**

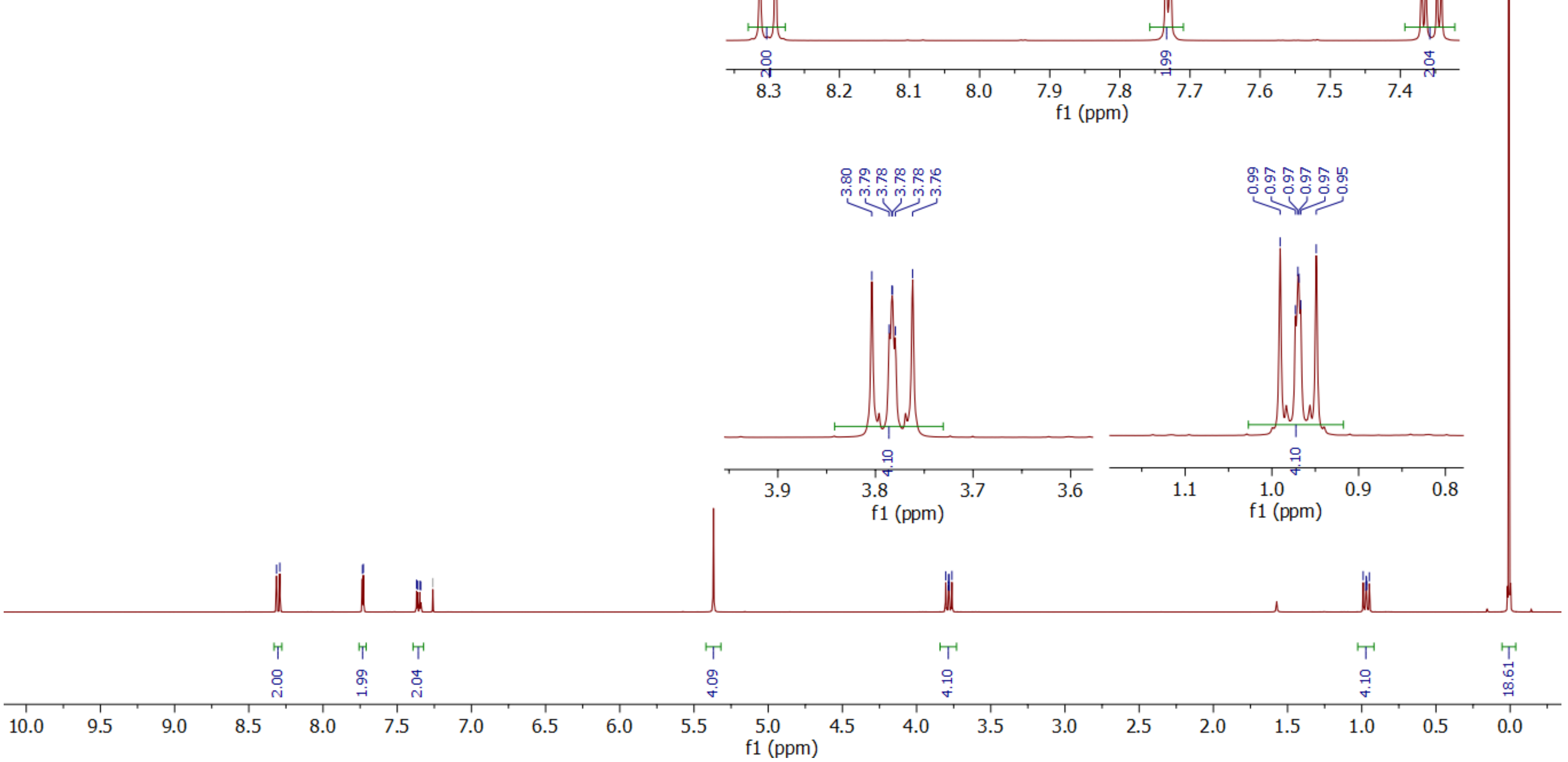

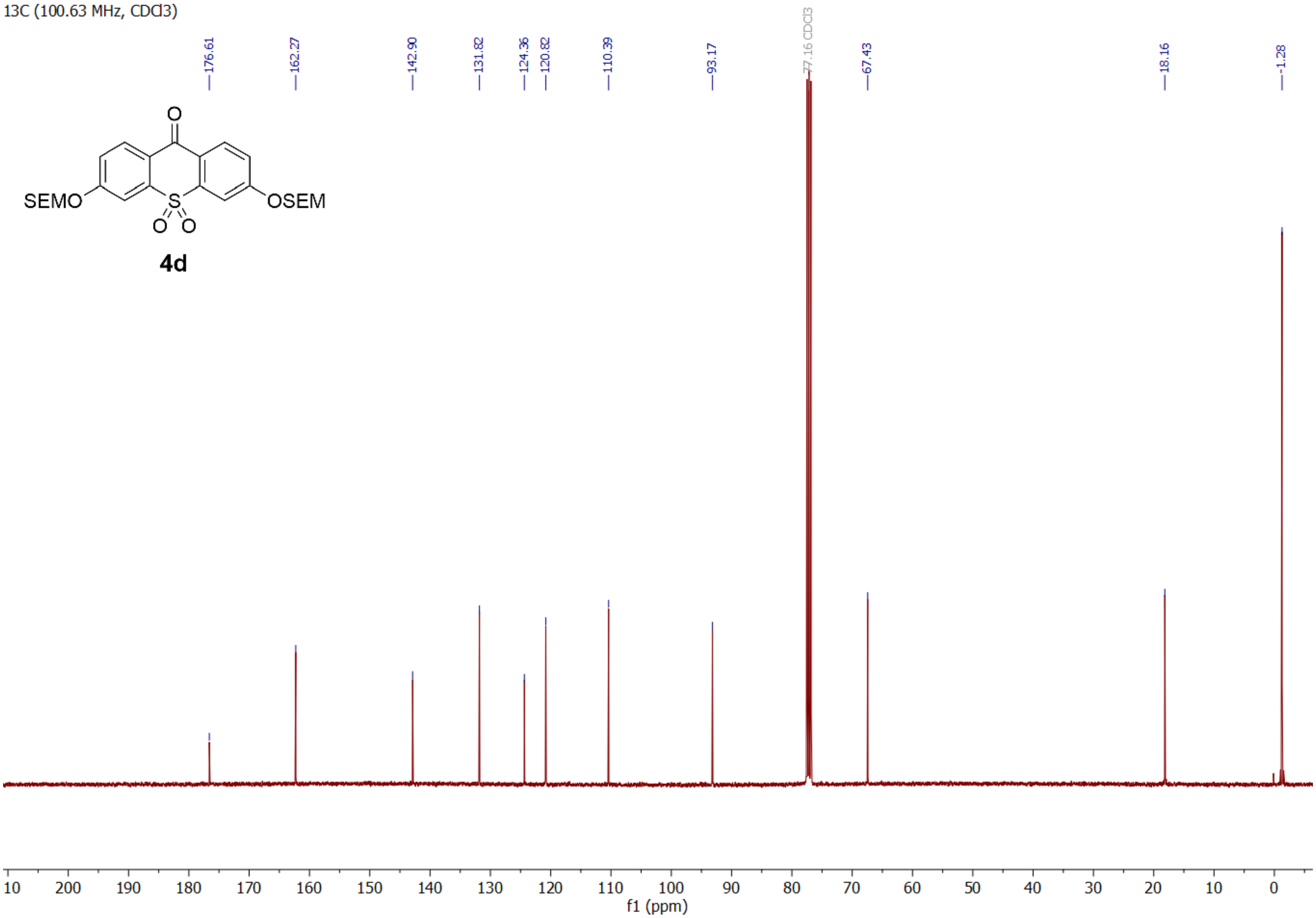
13C (100.63 MHz, CDCl3)
176.61
162.27
142.90
131.82
124.36
120.82
110.39
93.17
77.16 CDCl3
67.43
18.16
-1.28
10
200
190
180
170
160
150
140
130
120
110
100
90
80
70
60
50
40
30
20
10
0
f1 (ppm)
O
SEMO
S
OSEM
O
O

**4d**

1H (400.15 MHz, CDCl3)

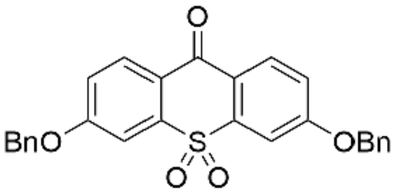


**4e**

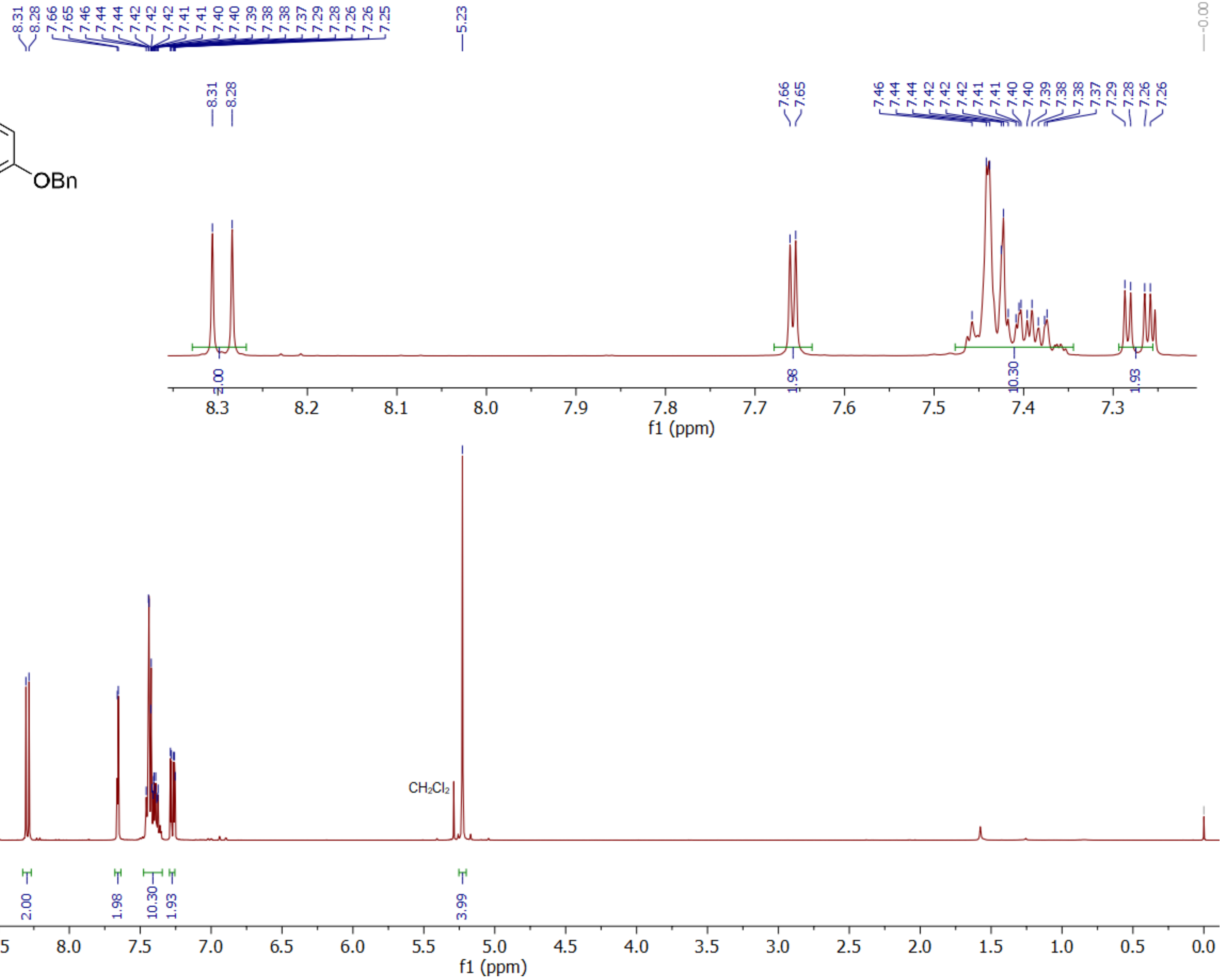

13C (100.63 MHz, CDCl3)

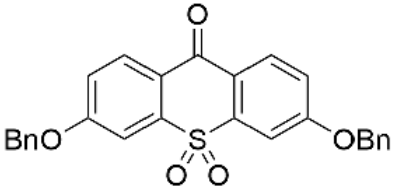

**4e**

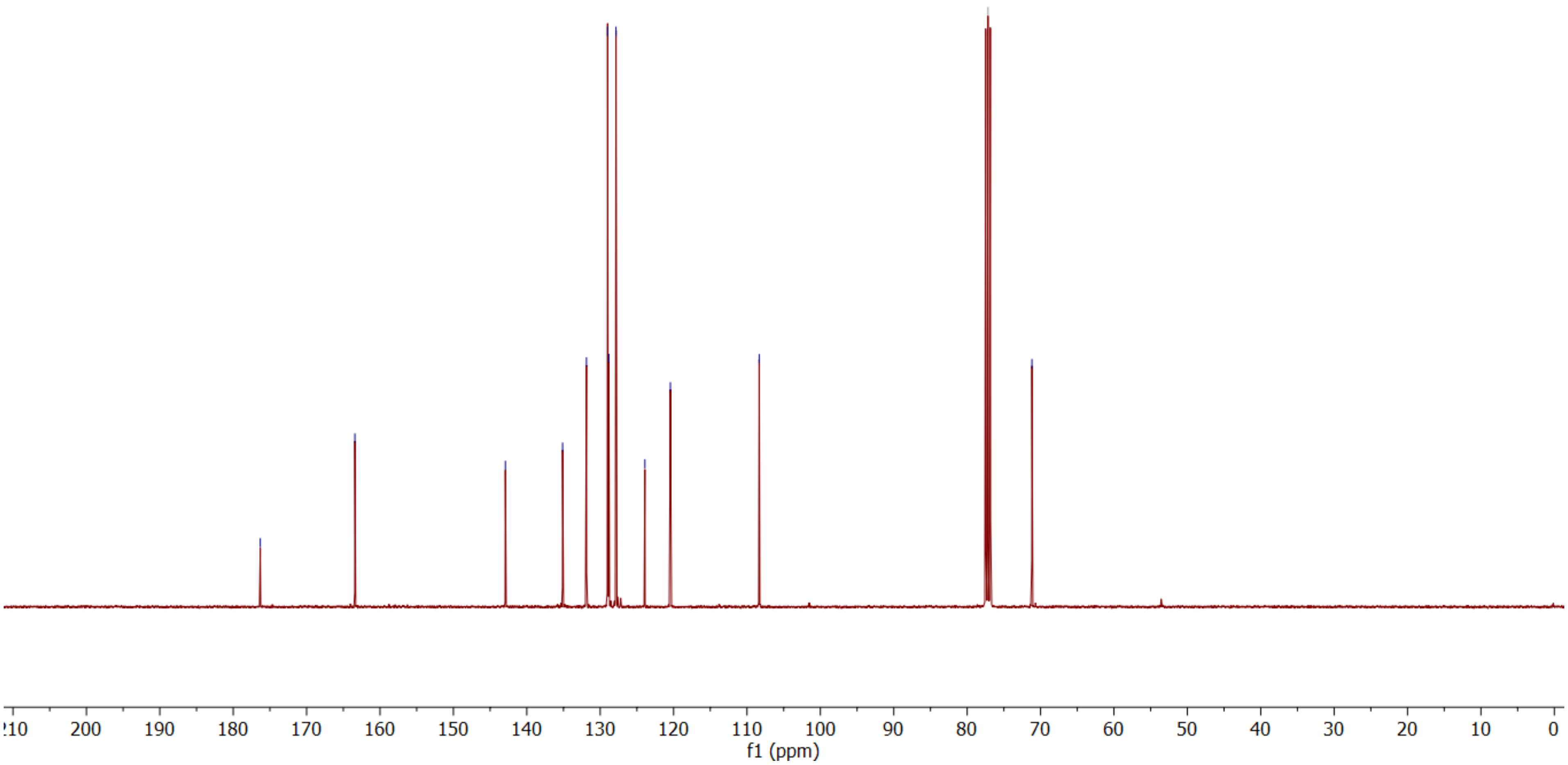

1H (400.15 MHz, CDCl3)

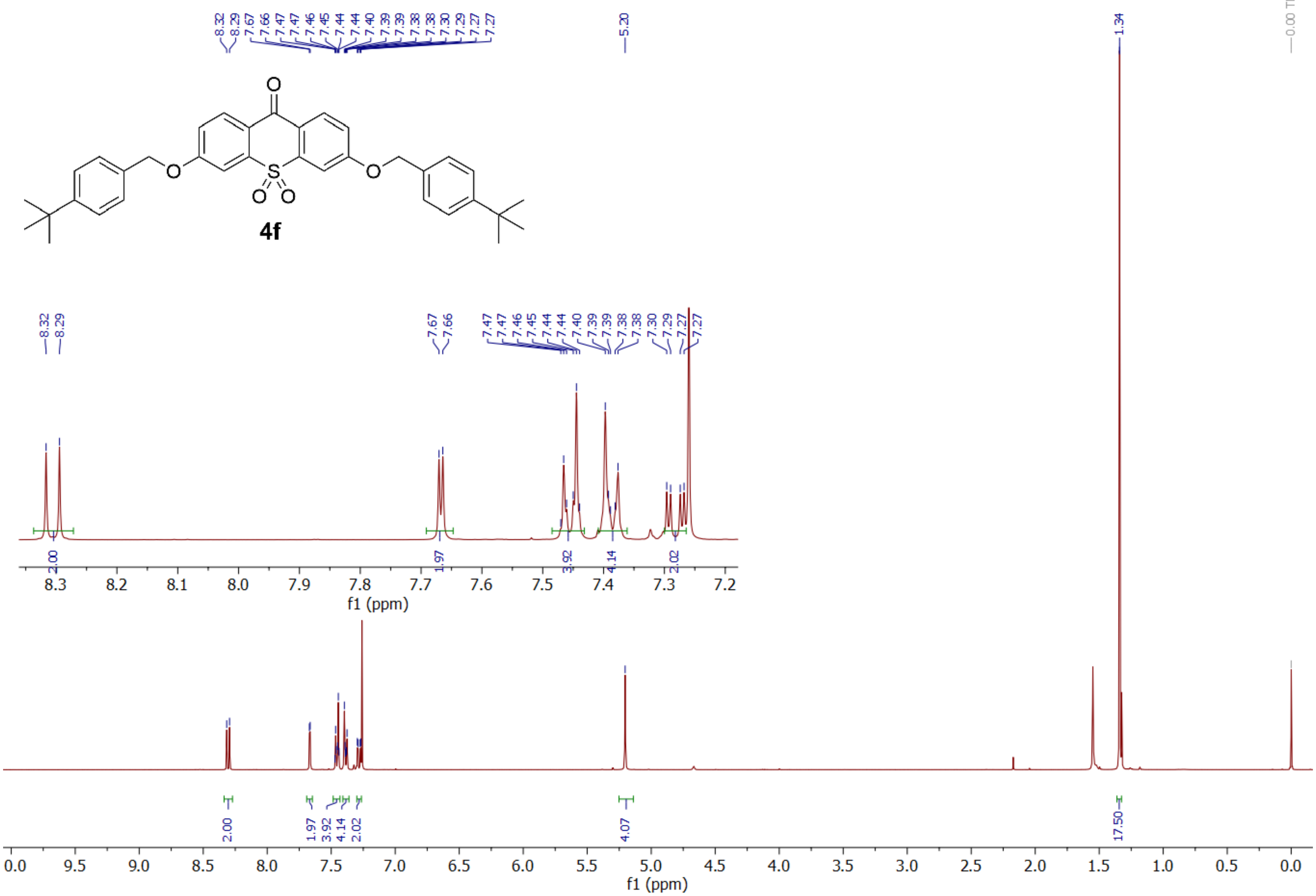

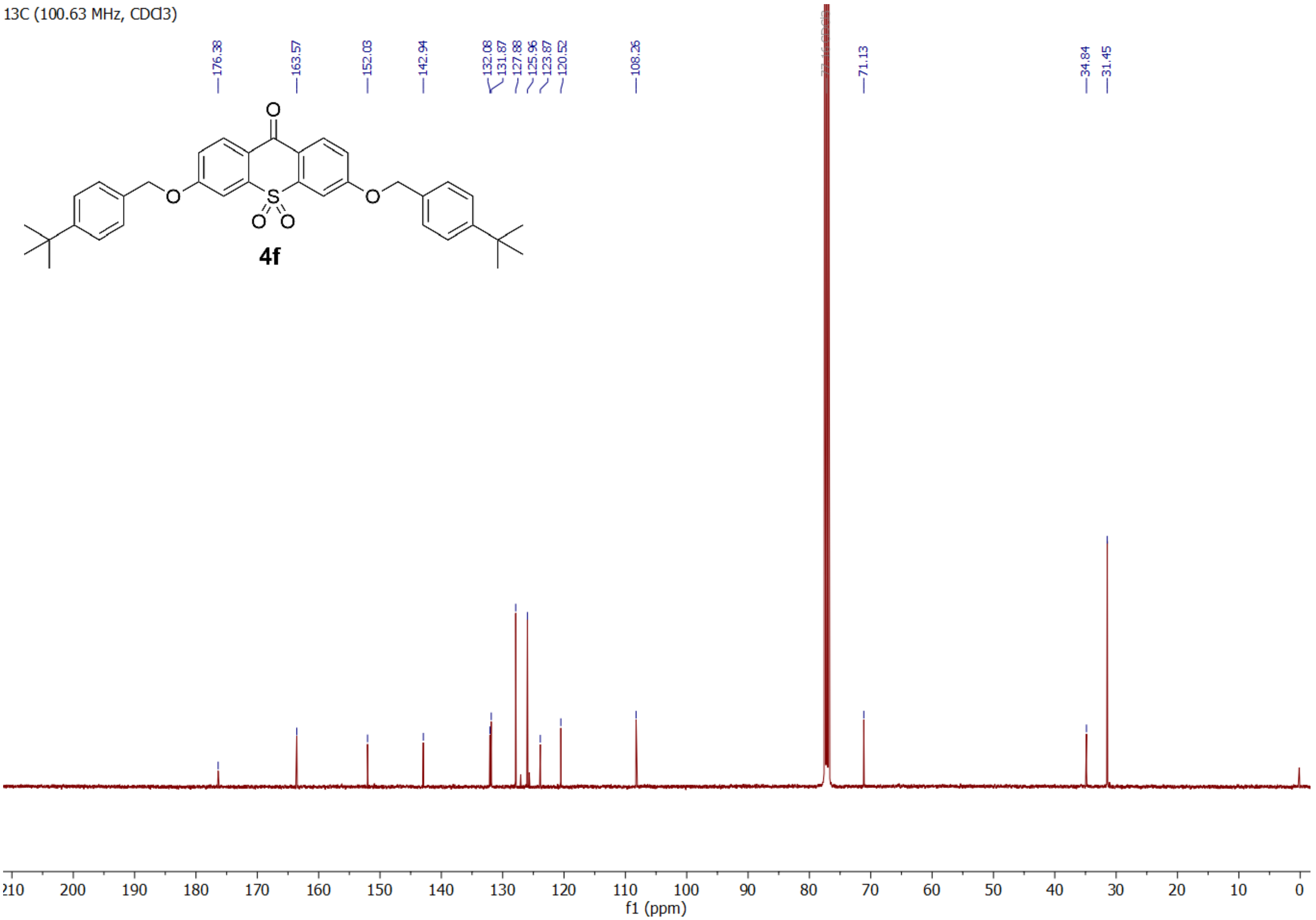
13C (100.63 MHz, CDCl3)
176.38
163.57
152.03
142.94
132.08
131.87
127.88
125.96
123.87
120.52
108.26
71.13
34.84
31.45
4f
f1 (ppm)

1H (400.15 MHz, CDCl3)

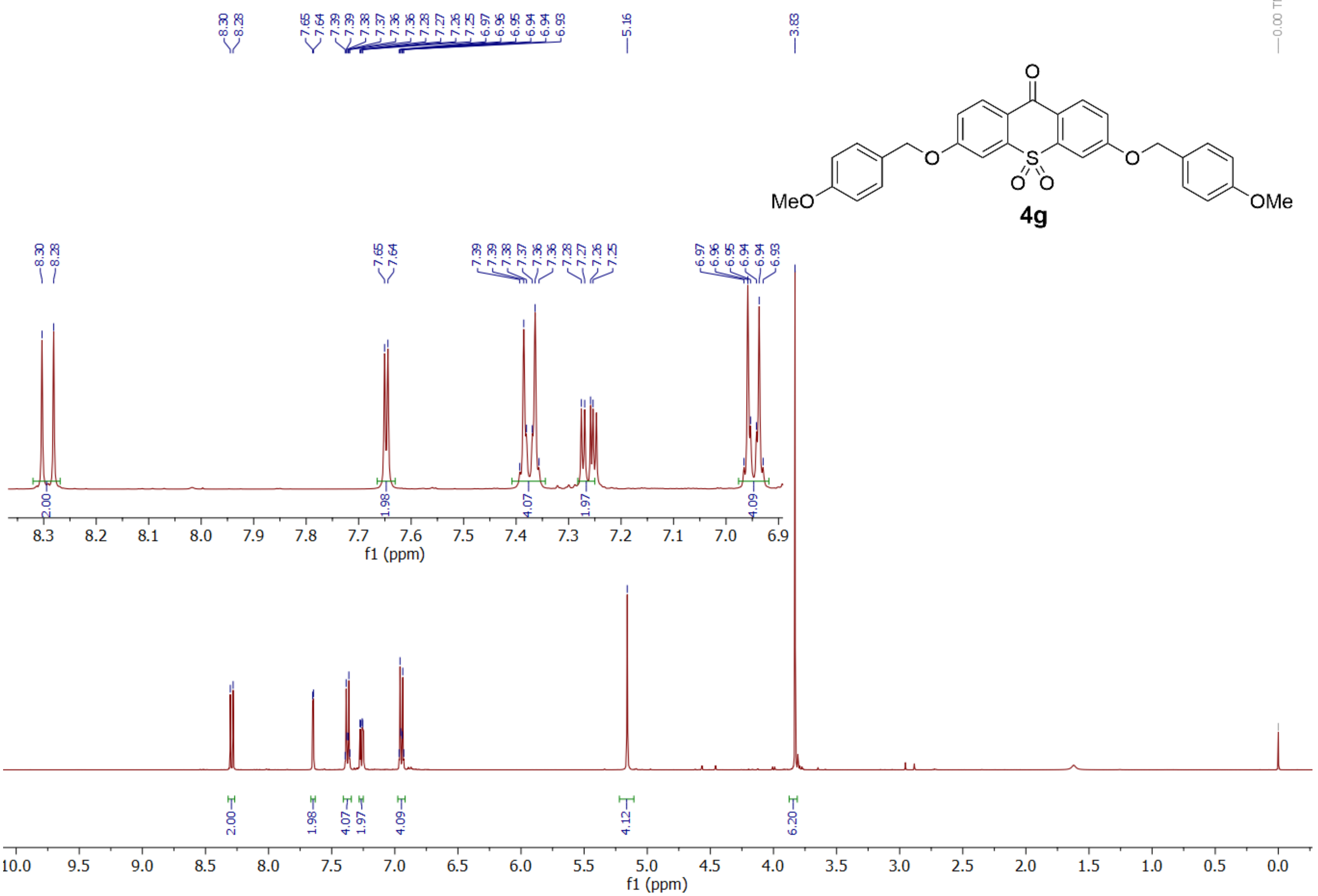

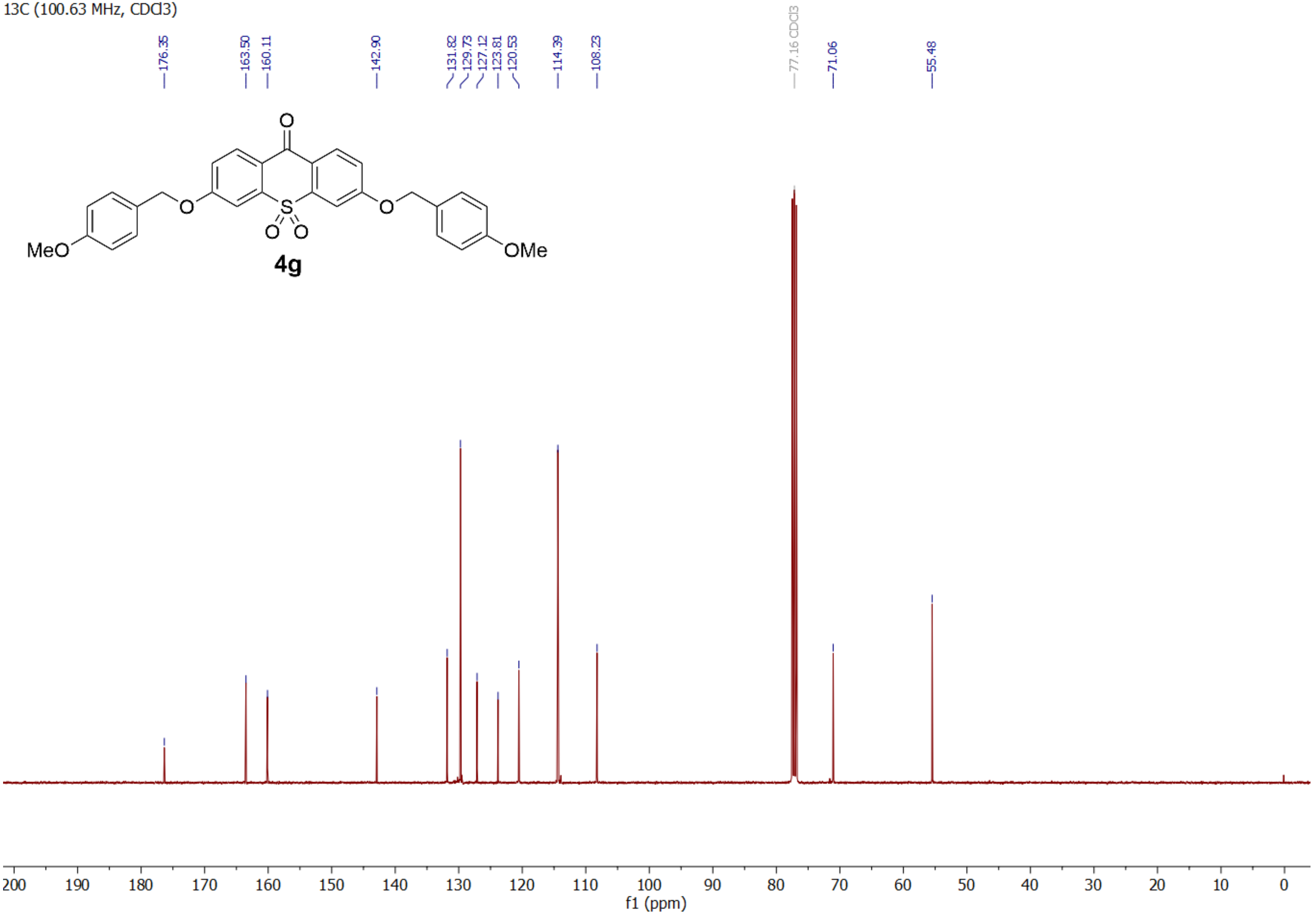
13C (100.63 MHz, CDCl3)
176.35
163.50
160.11
142.90
131.82
129.73
127.12
123.81
120.53
114.39
108.23
77.16 CDCl3
71.06
55.48
O
S
O
O
MeO
O
O
OMe
4g
200 190 180 170 160 150 140 130 120 110 100 90 80 70 60 50 40 30 20 10 0
f1 (ppm)

1H (400.15 MHz, CD3CN)

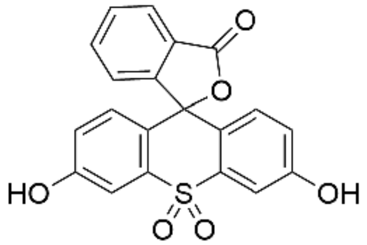
5a

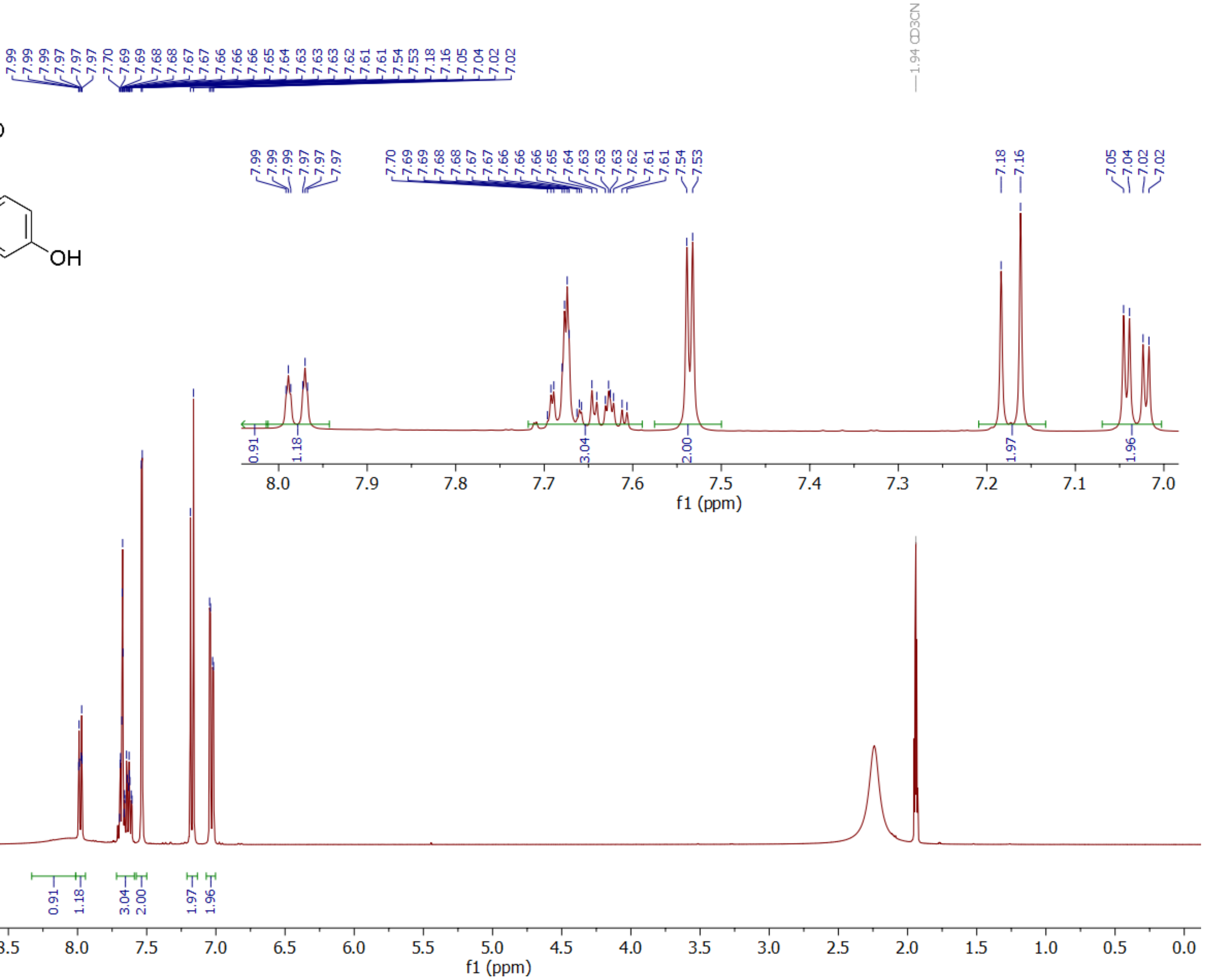

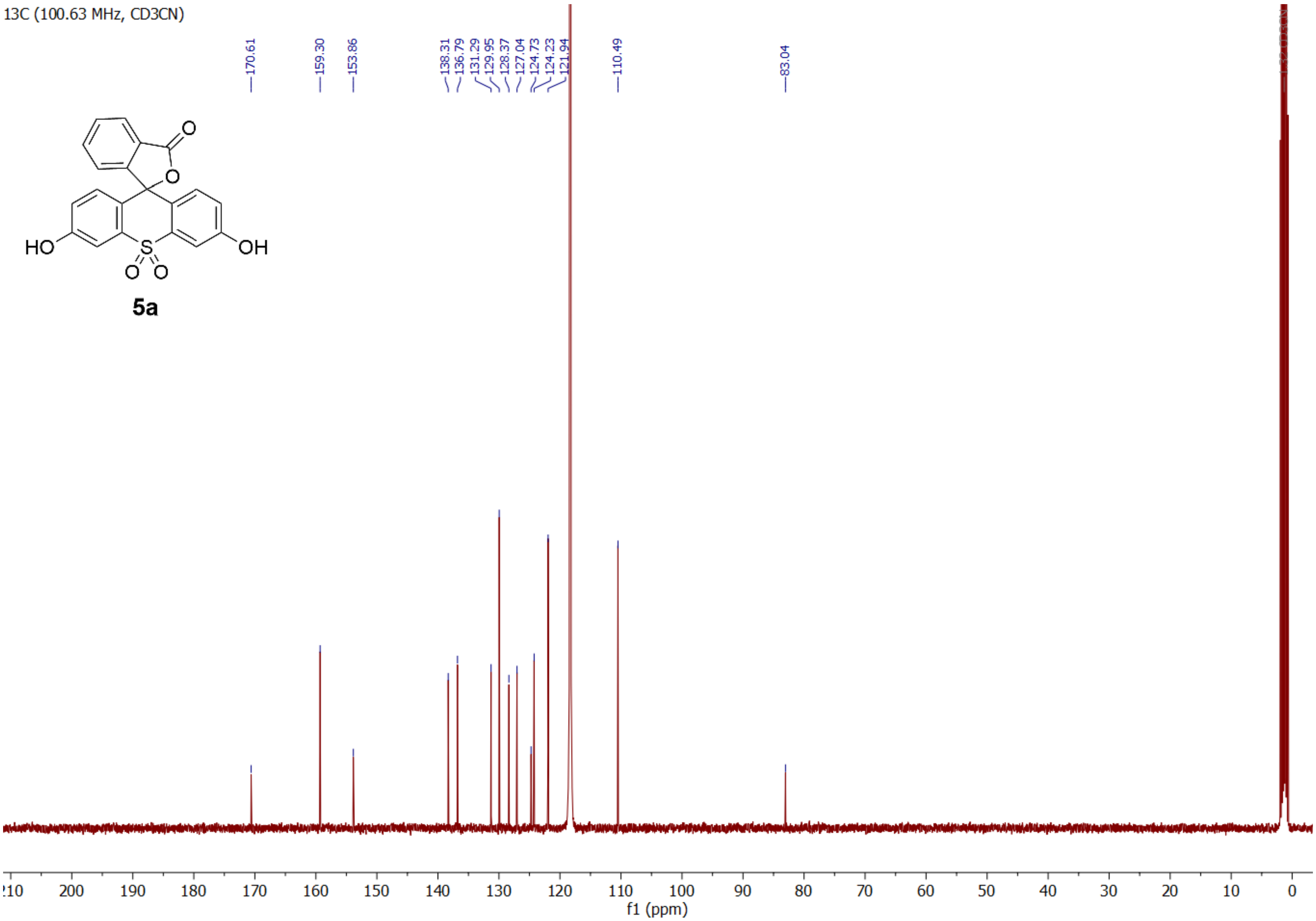
13C (100.63 MHz, CD3CN)
170.61
159.30
153.86
138.31
136.79
131.29
129.95
128.37
127.04
124.73
124.23
121.94
110.49
83.04
O
O
HO
S
O
O
OH
5a
200
190
180
170
160
150
140
130
120
110
100
90
80
70
60
50
40
30
20
10
0
f1 (ppm)

1H (400.15 MHz, CD3CN)

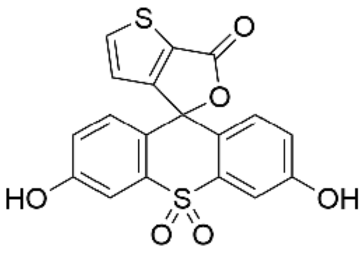

5b

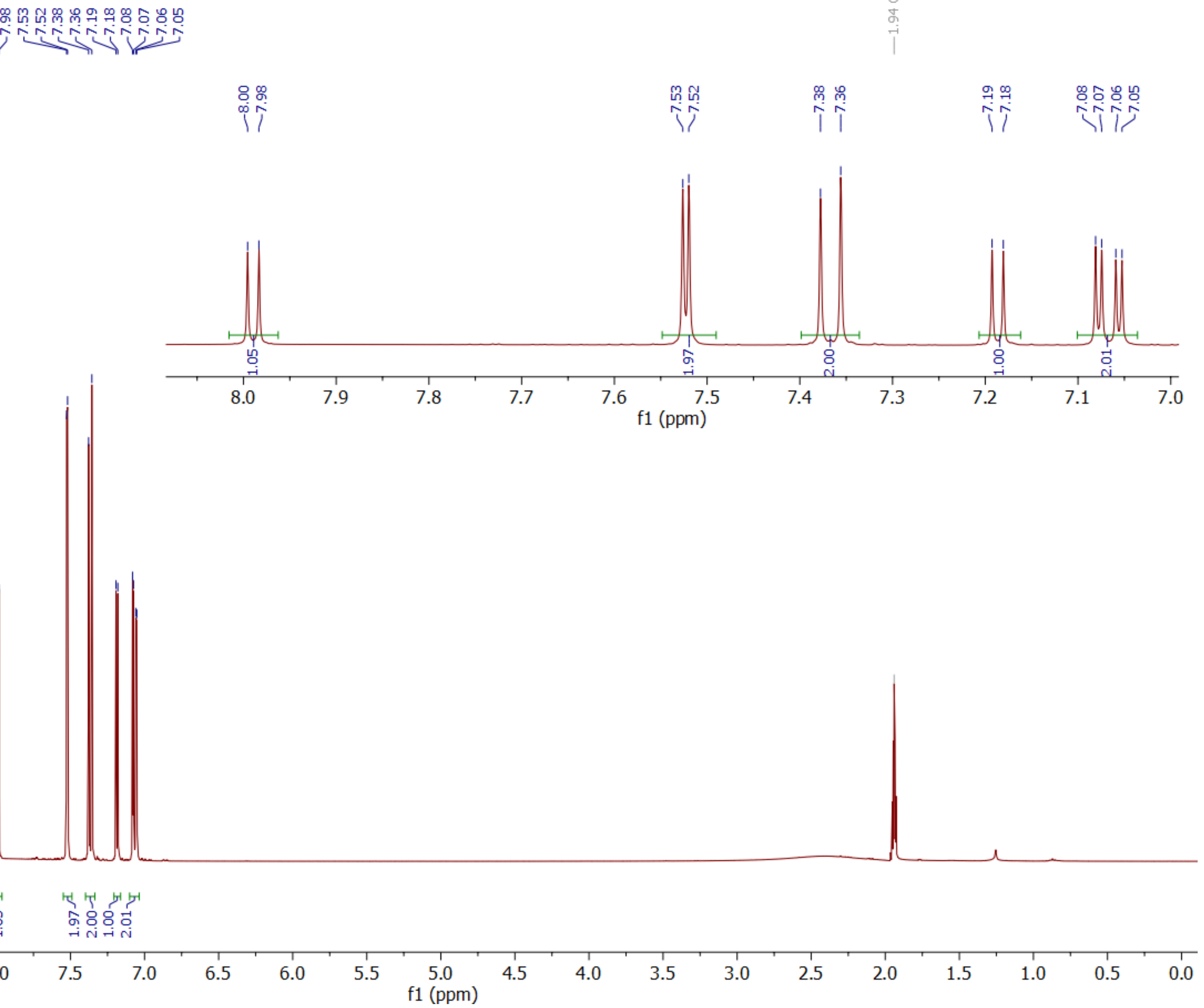

13C (100.63 MHz, CD3CN)

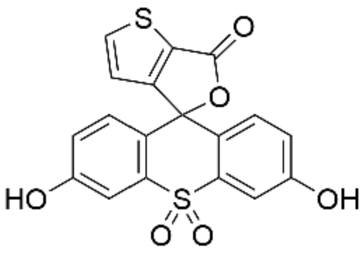

**5b**

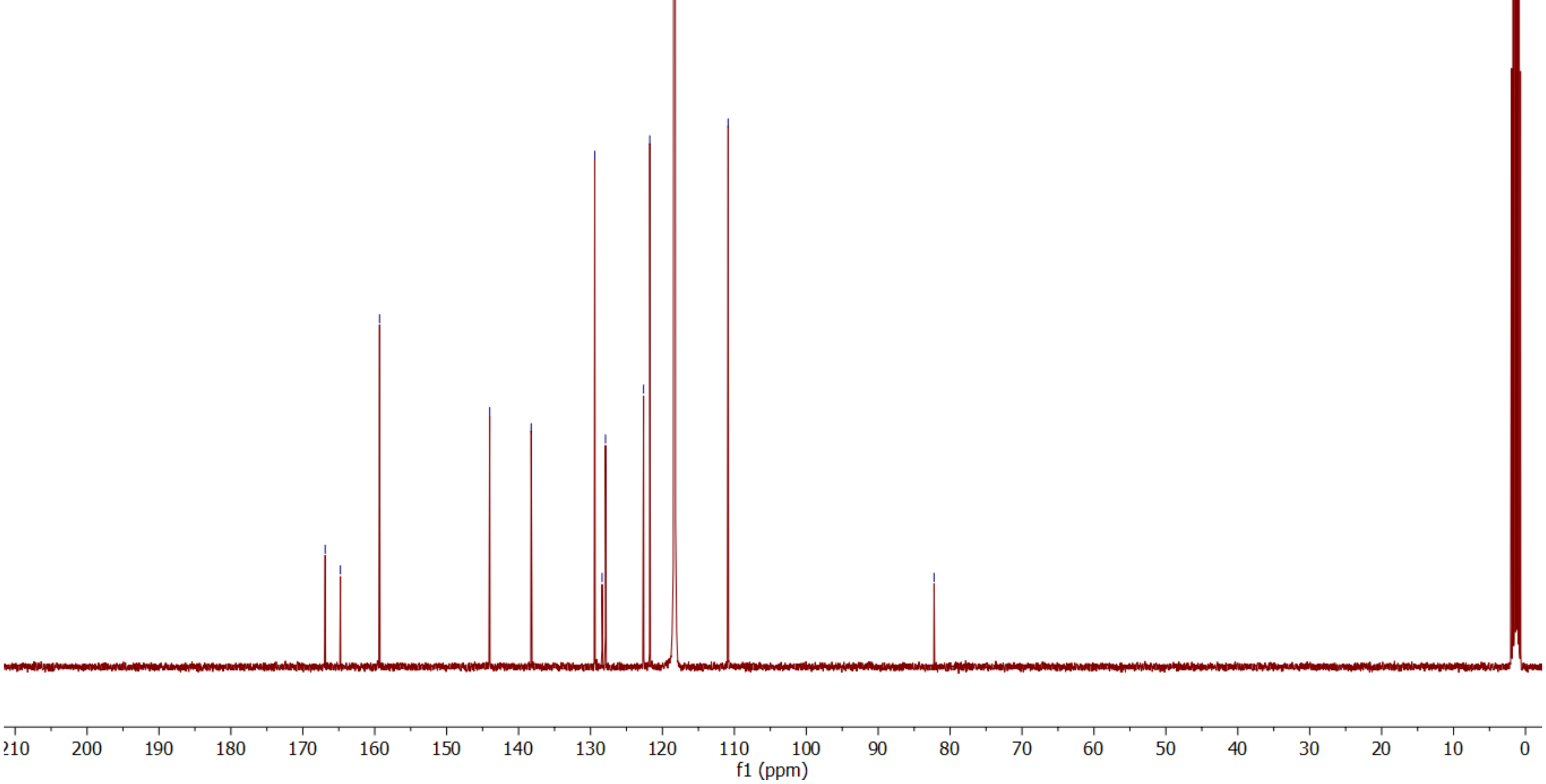

1H (400.15 MHz, CDCl3:TFA-d 10:1 (v/v))

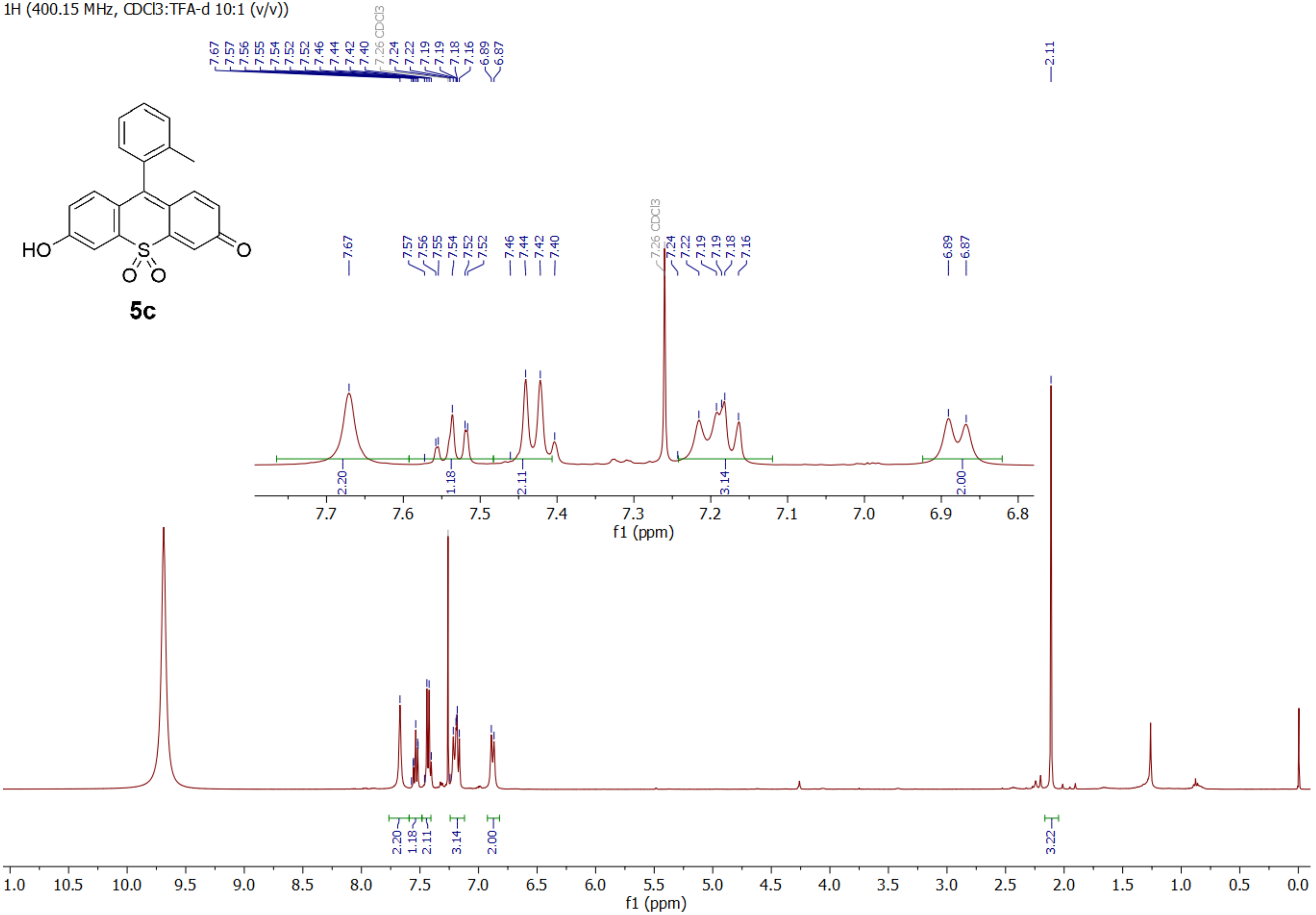

13C (100.63 MHz, CDCl3:TFA-d 10:1 (v/v))

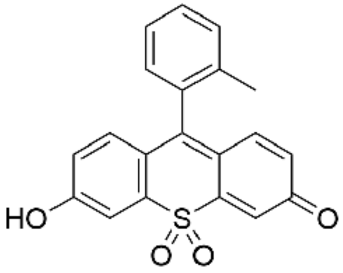

5c

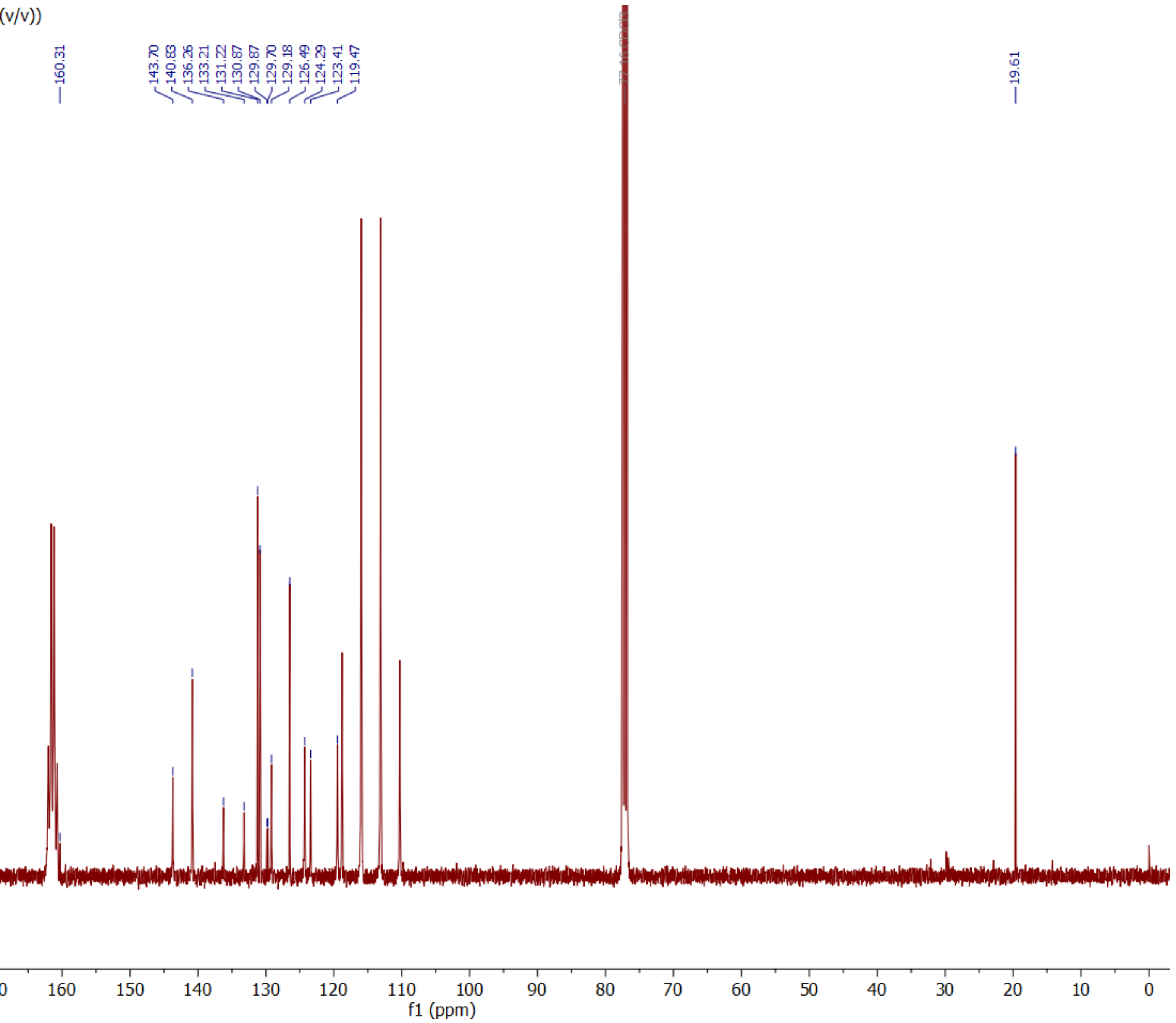

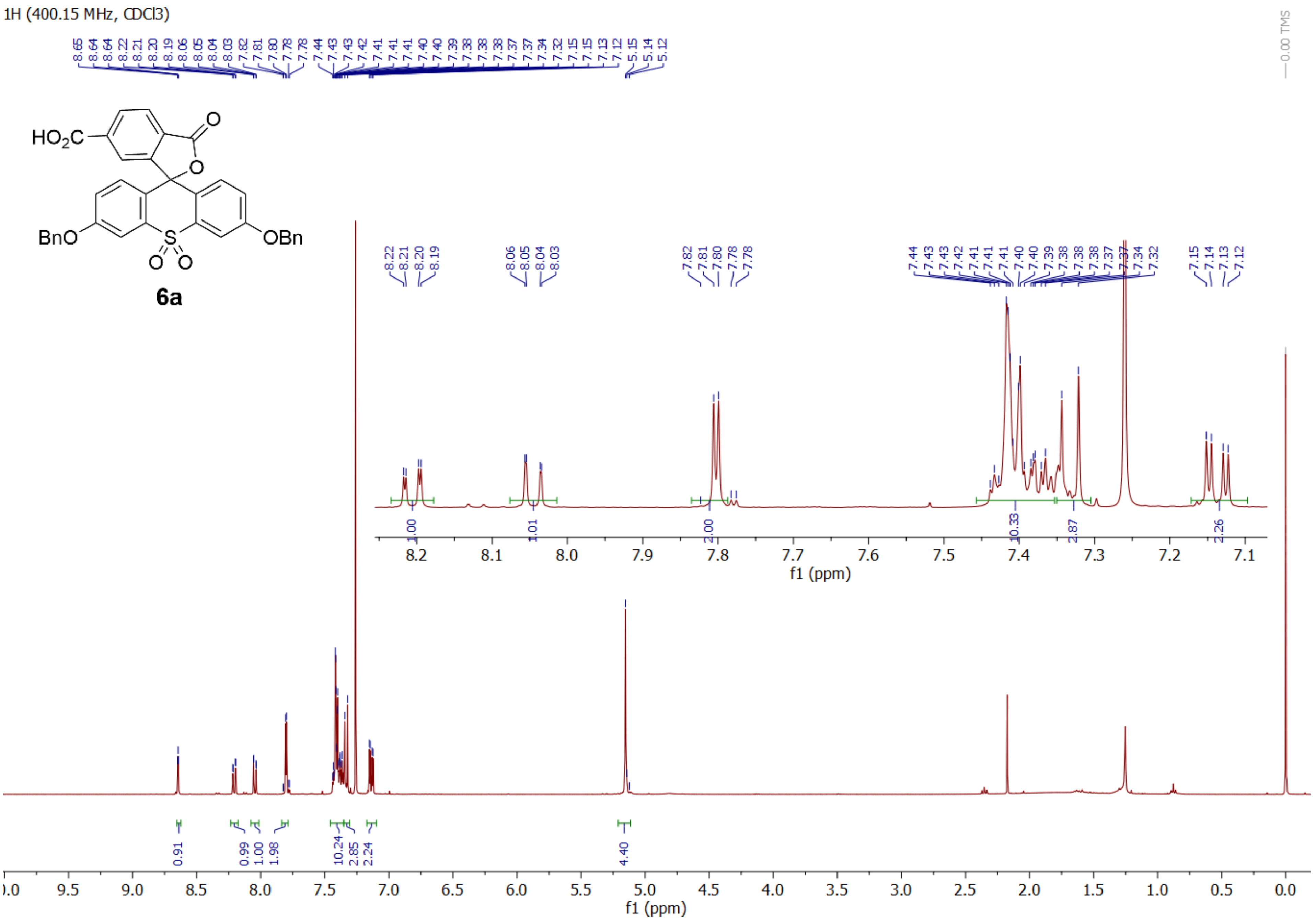
1H (400.15 MHz, CDCl3)
8.65 8.64 8.64 8.22 8.21 8.20 8.19 8.06 8.05 8.04 8.03 7.82 7.81 7.80 7.78 7.78 7.44 7.43 7.43 7.42 7.41 7.41 7.41 7.40 7.40 7.39 7.38 7.38 7.38 7.37 7.37 7.34 7.32 7.15 7.15 7.13 7.12 5.15 5.14 5.12
0.00 TMS
HO2C
O
O
BnO
OBn
S
O O
6a
1.00 1.01 2.00 10.33 2.87 2.26
f1 (ppm)
0.91 0.99 1.00 1.98 10.24 2.85 2.24 4.40
f1 (ppm)

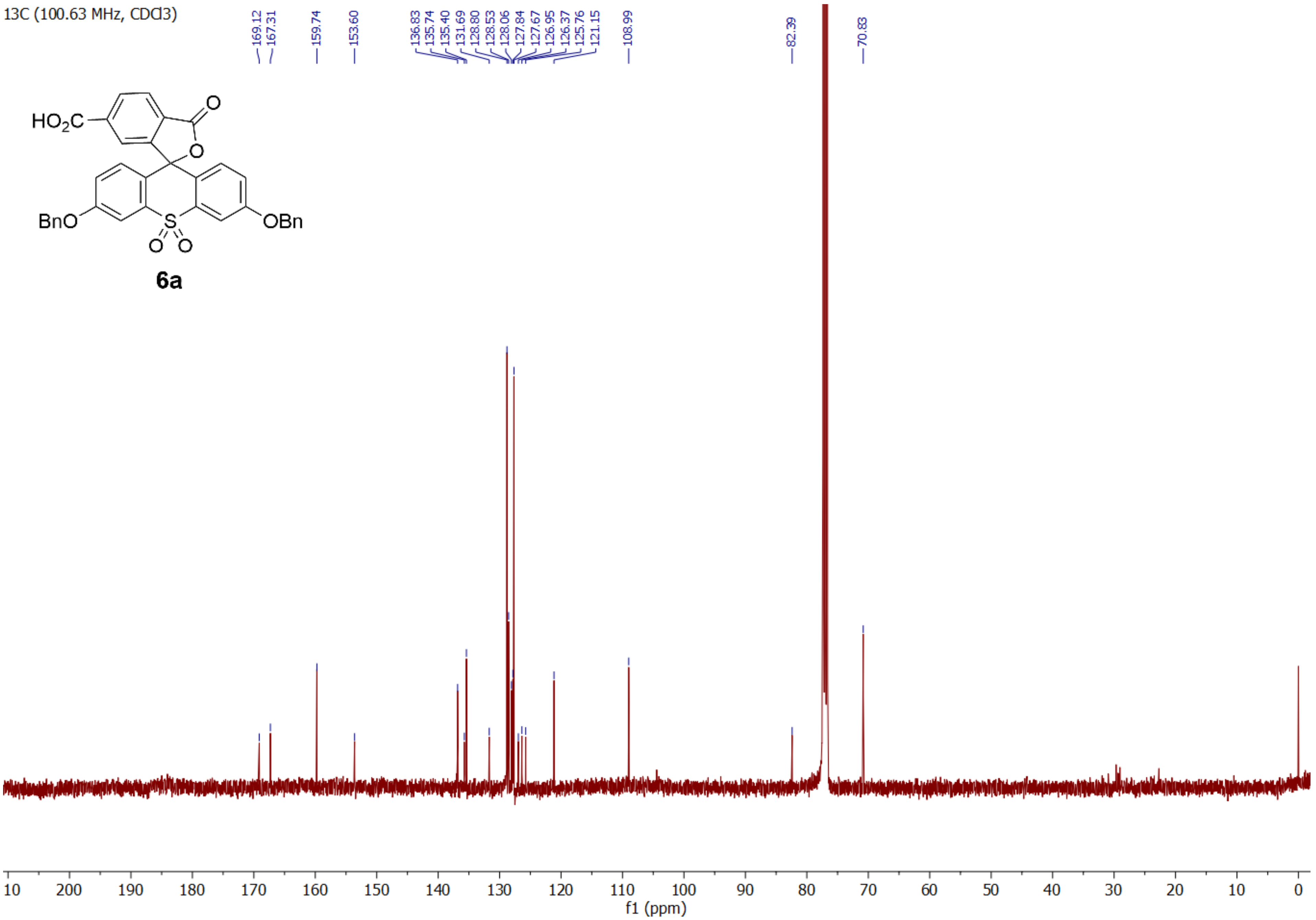
13C (100.63 MHz, CDCl3)
169.12
167.31
159.74
153.60
136.83
135.74
135.40
131.69
128.80
128.53
128.06
127.84
127.67
126.95
126.37
125.76
121.15
108.99
82.39
70.83
HO2C
O
O
BnO
S
O
O
OBn
6a
f1 (ppm)

1H (400.15 MHz, CDCl3)

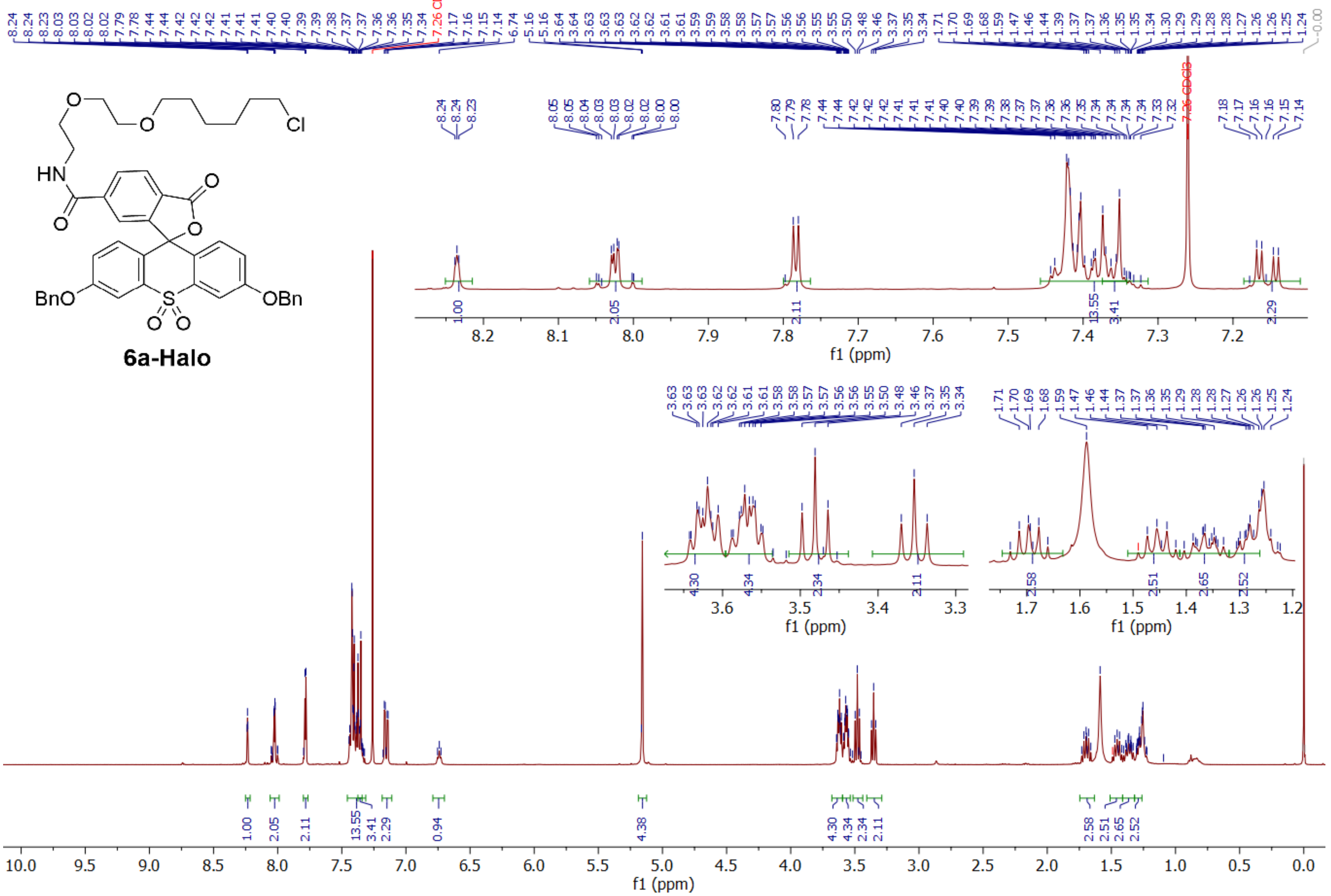


**6a-Halo**

13C (100.63 MHz, CDCl3)

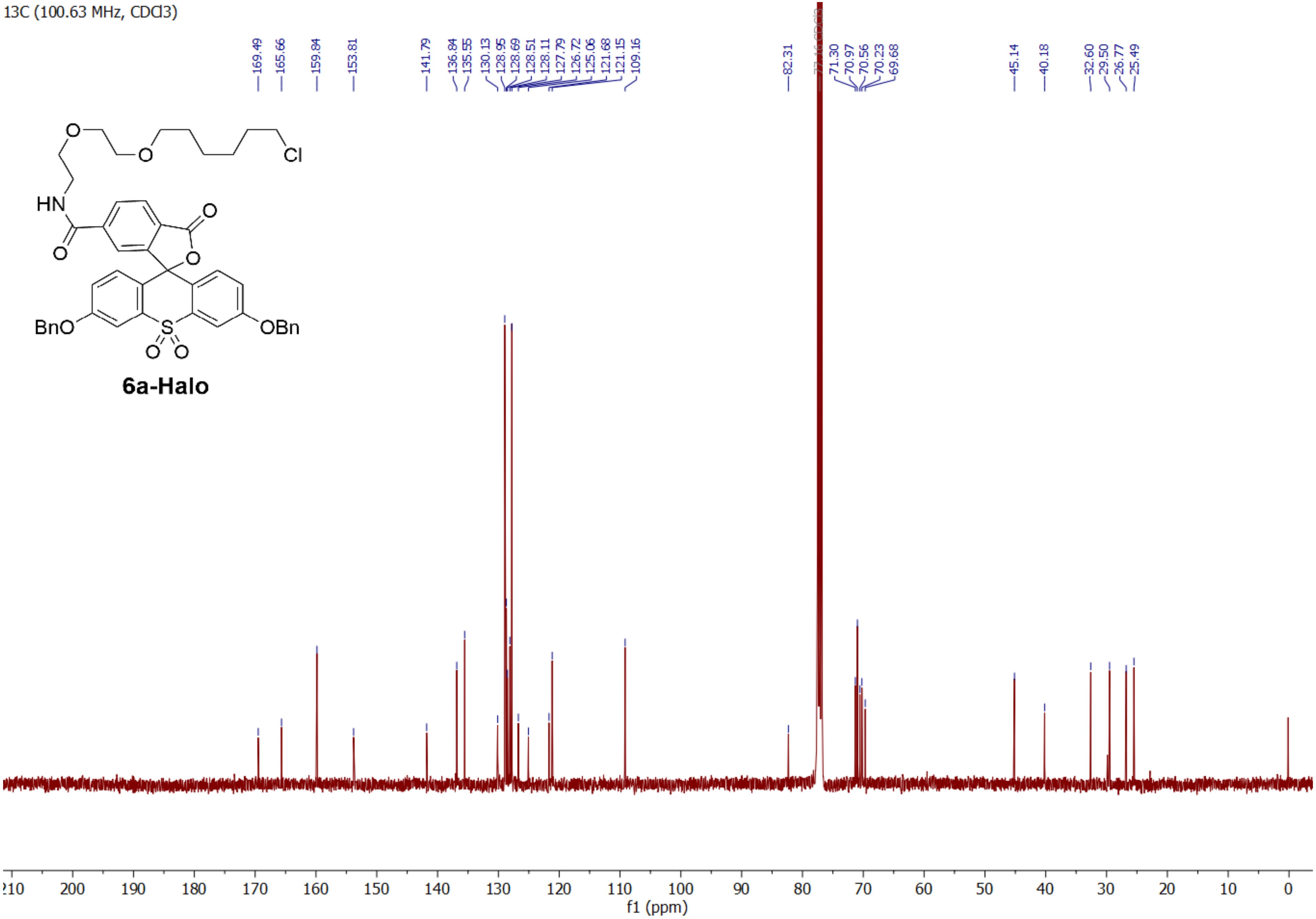

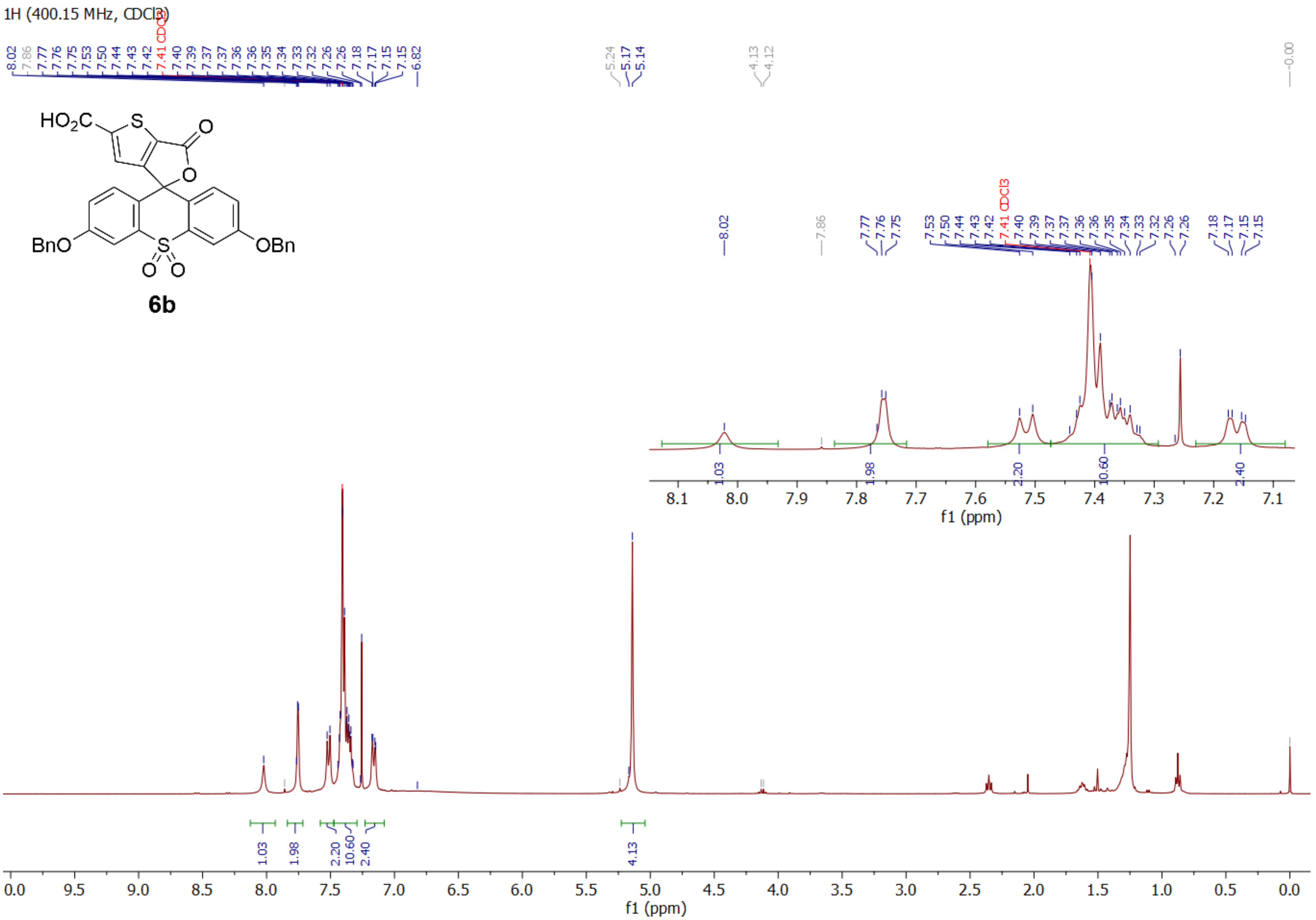
1H (400.15 MHz, CDCl3)
6b
f1 (ppm)

13C (100.63 MHz, CDCl3)

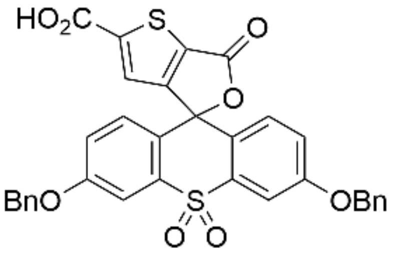


**6b**

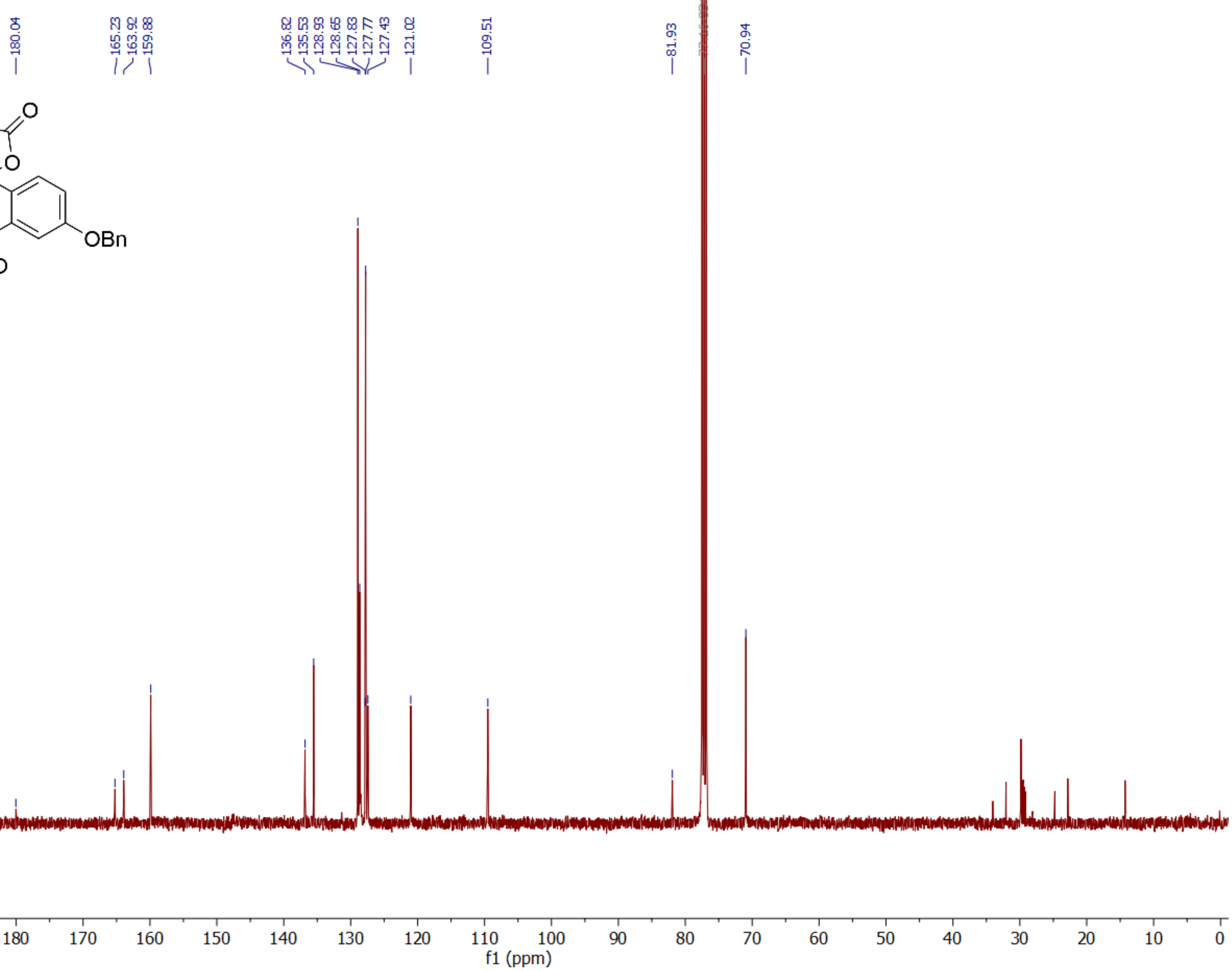

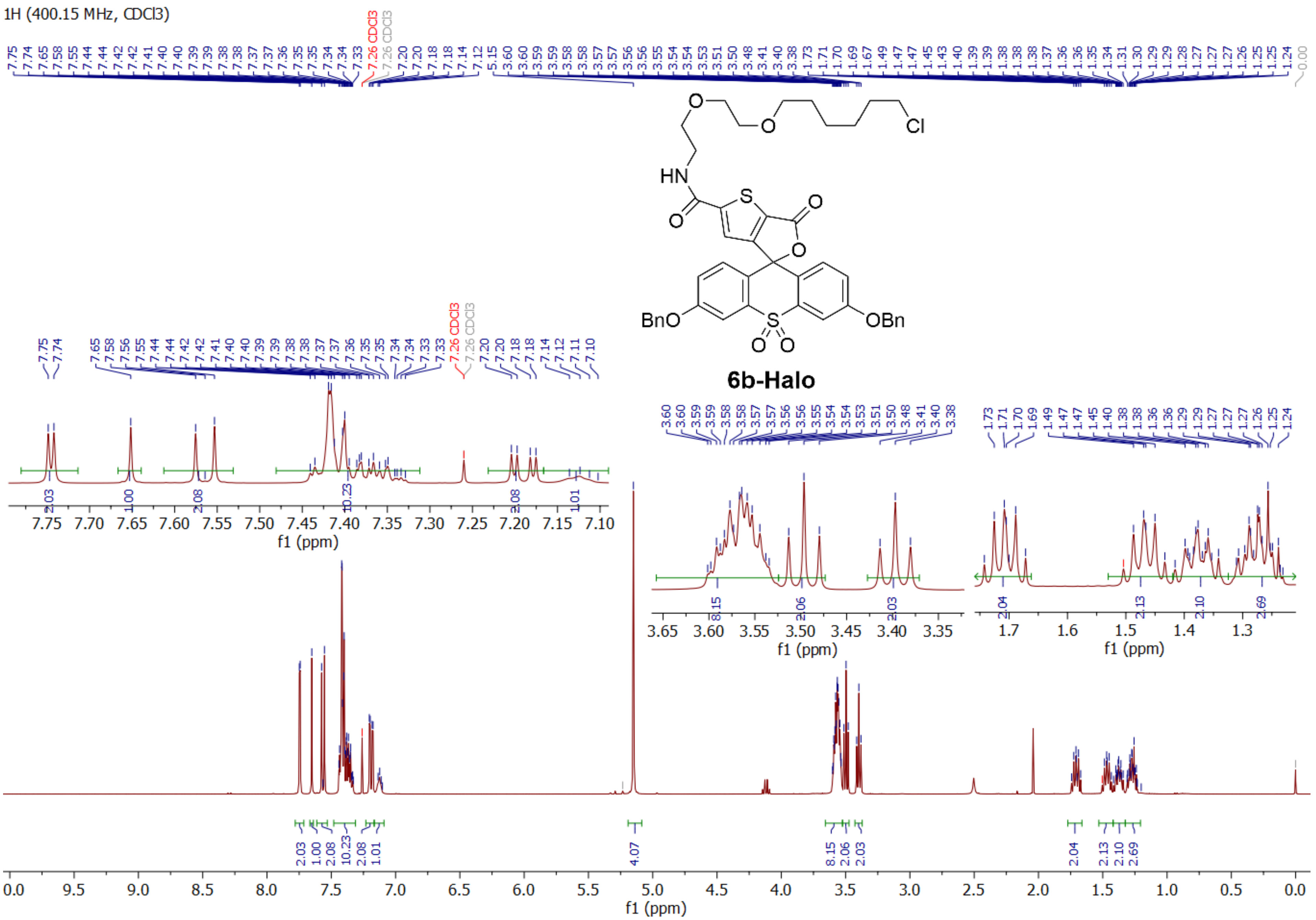
1H (400.15 MHz, CDCl3)
7.75 7.74 7.65 7.58 7.55 7.44 7.44 7.42 7.42 7.41 7.40 7.39 7.39 7.38 7.38 7.37 7.36 7.35 7.35 7.34 7.34 7.33 7.26 CDCl3 7.26 CDCl3 7.20 7.20 7.18 7.18 7.14 7.12 5.15 3.60 3.60 3.59 3.59 3.58 3.58 3.57 3.57 3.56 3.56 3.55 3.54 3.54 3.53 3.51 3.50 3.48 3.41 3.40 3.38 1.73 1.71 1.70 1.69 1.67 1.49 1.47 1.47 1.45 1.43 1.40 1.39 1.39 1.38 1.38 1.38 1.37 1.36 1.36 1.35 1.34 1.31 1.30 1.29 1.29 1.28 1.27 1.27 1.27 1.26 1.25 1.25 1.24 0.00
HN
S
O
Cl
BnO
OBn
6b-Halo
2.03 1.00 2.08 10.23 2.08 1.01 4.07 8.15 2.06 2.03 2.04 2.13 2.10 2.69
f1 (ppm)

13C (100.63 MHz, CDCl3)

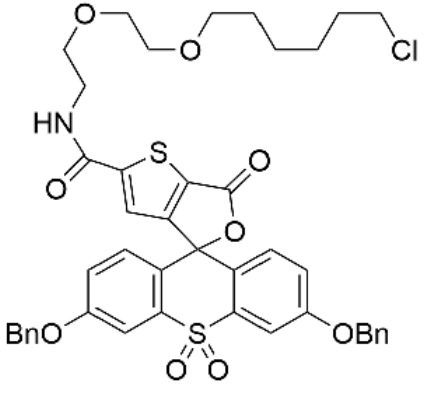


**6b-Halo**

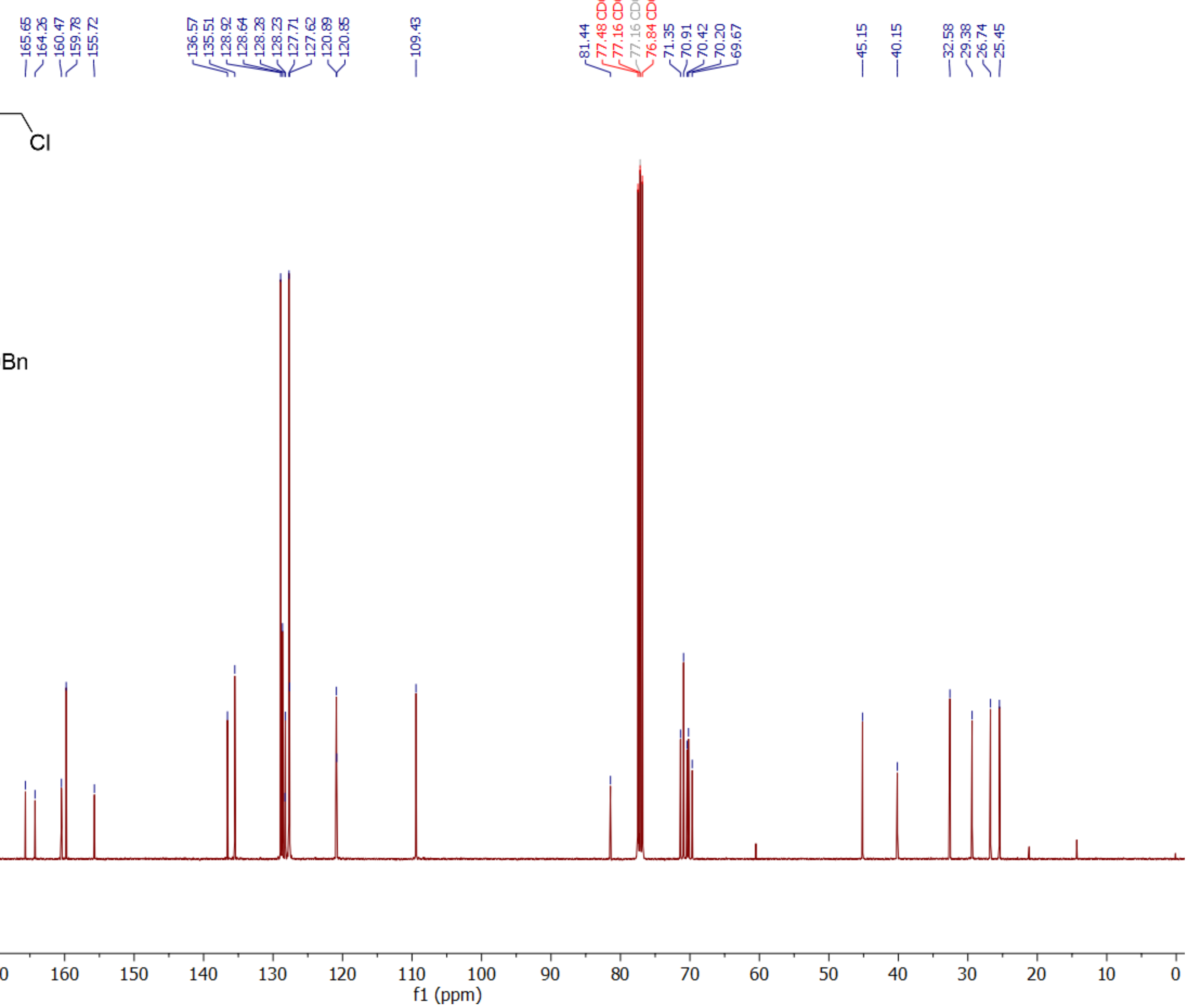

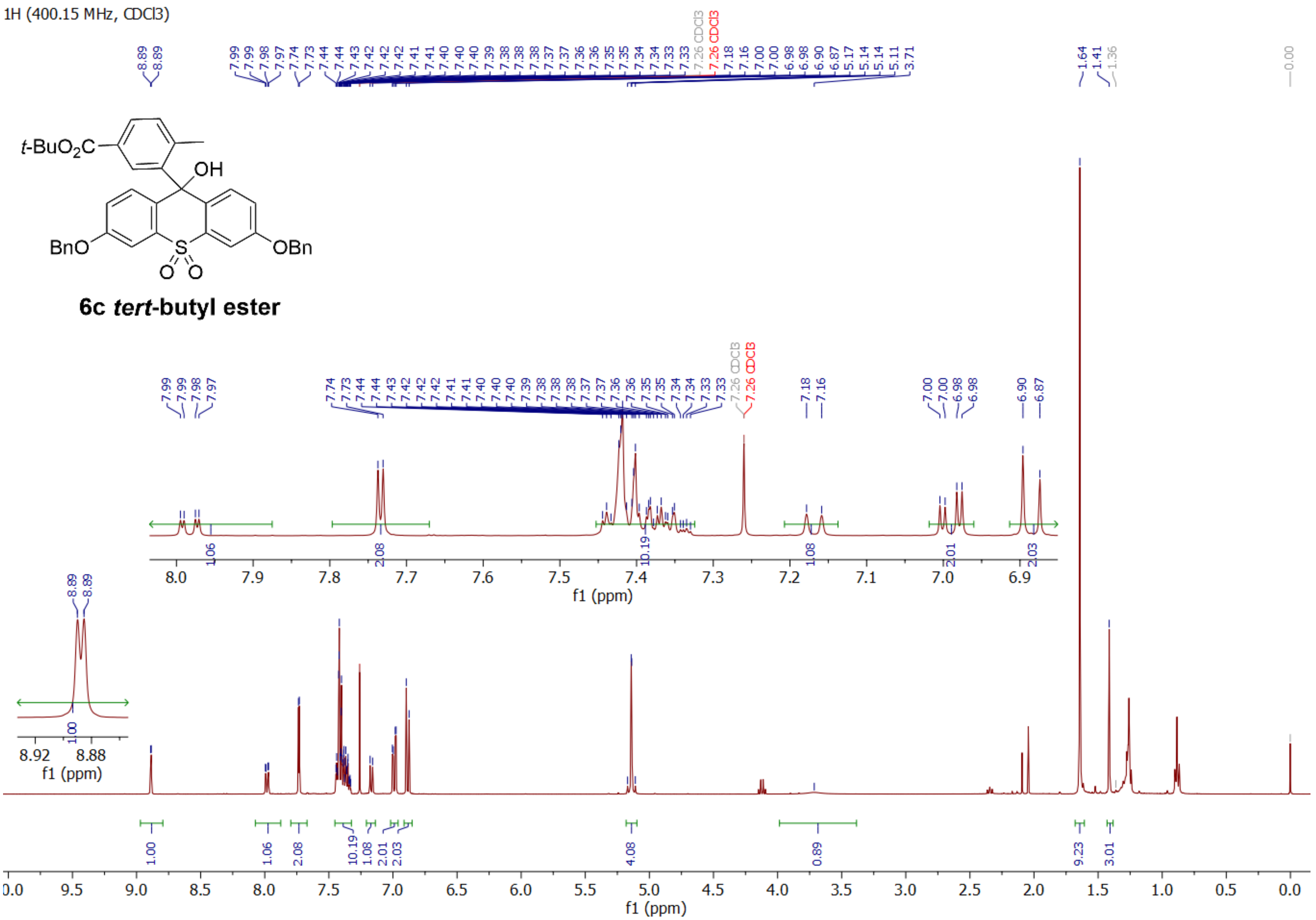


**6c *tert*-butyl ester**

13C (100.63 MHz, CDCl3)

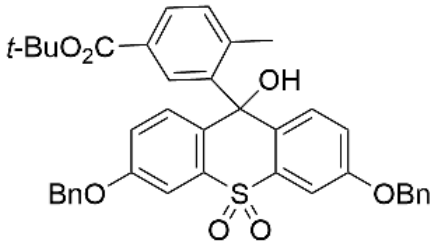


**6c *tert*-butyl ester**

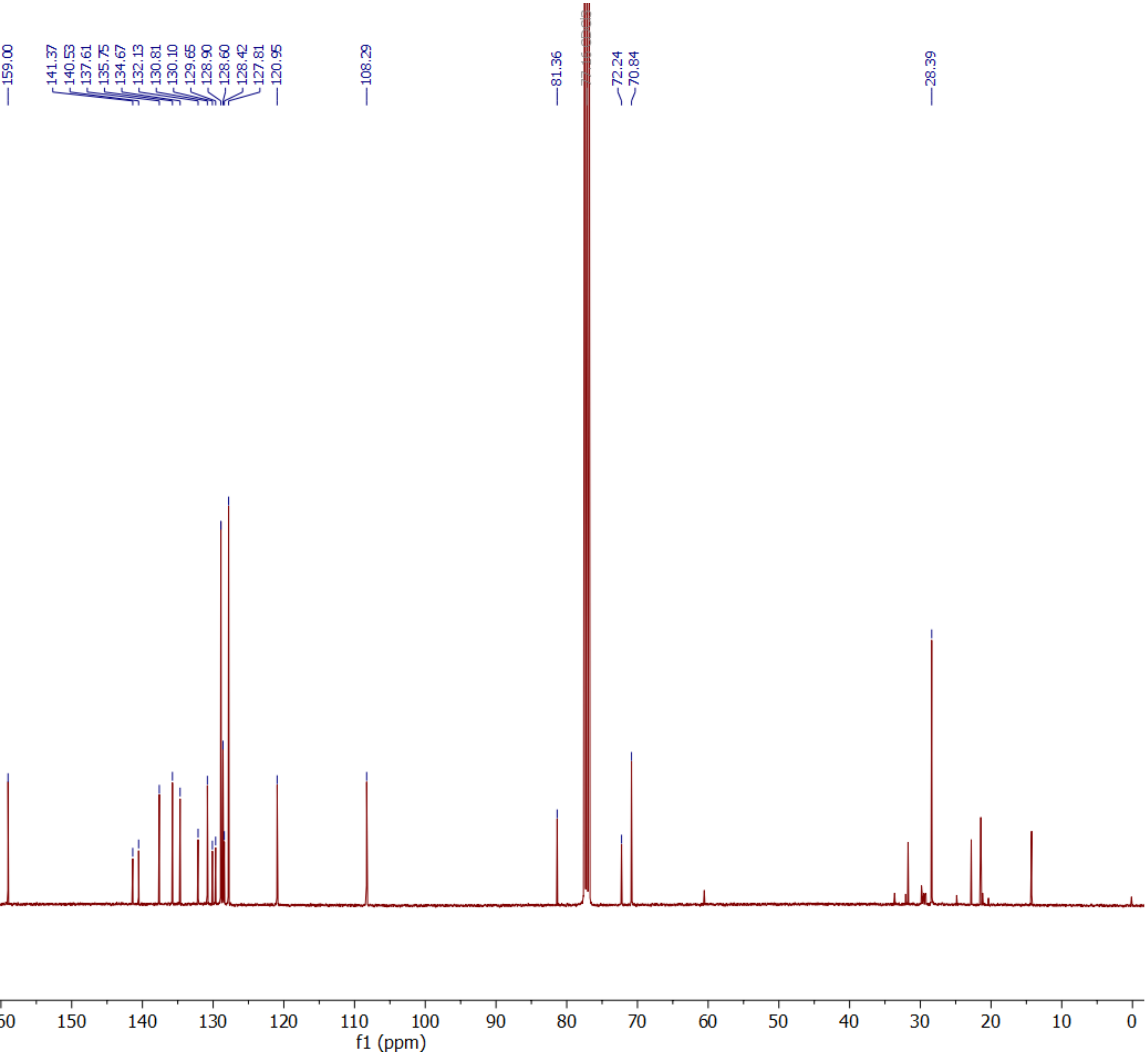

1H (400.15 MHz, CD3CN)

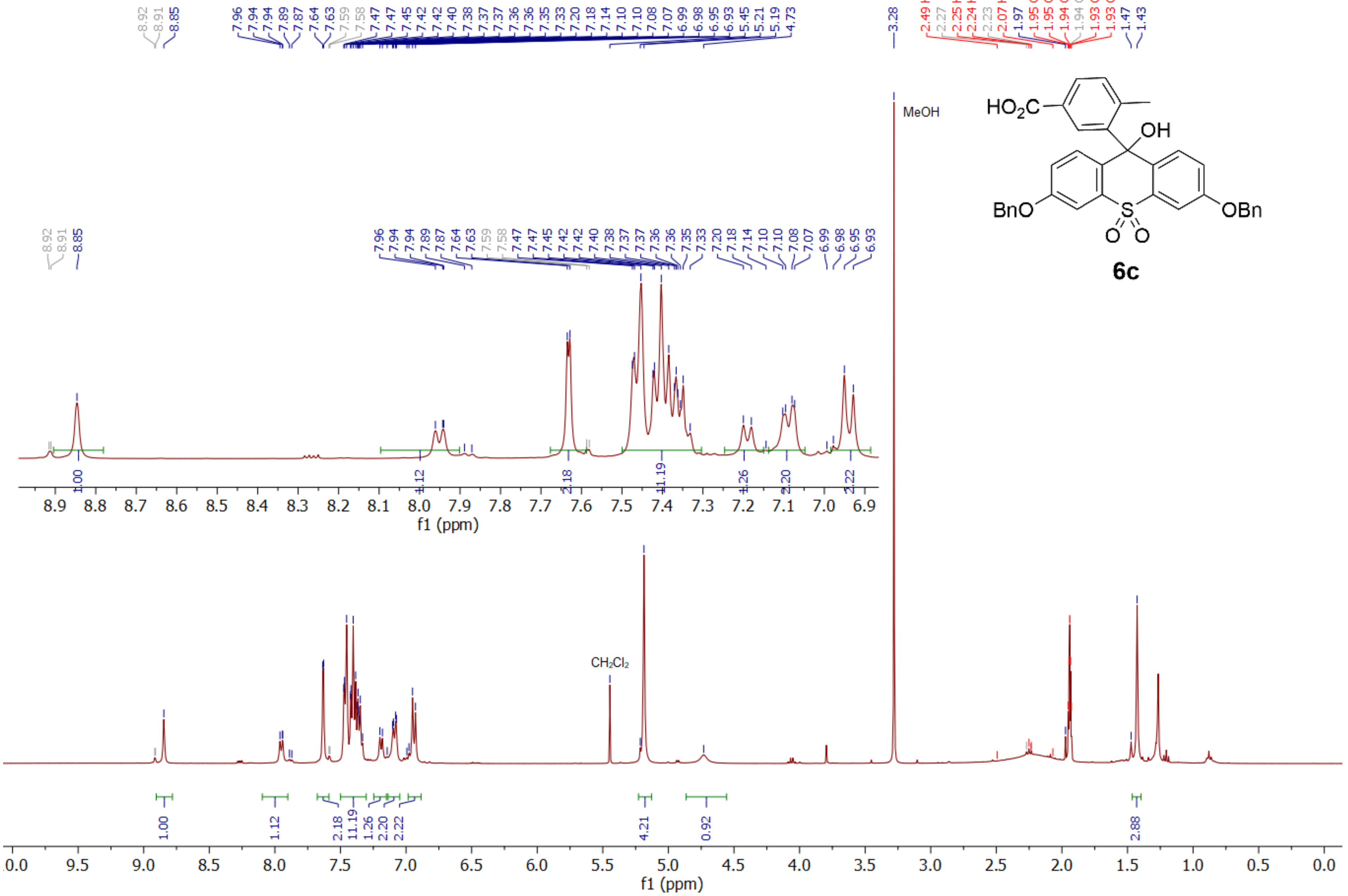


**6c**

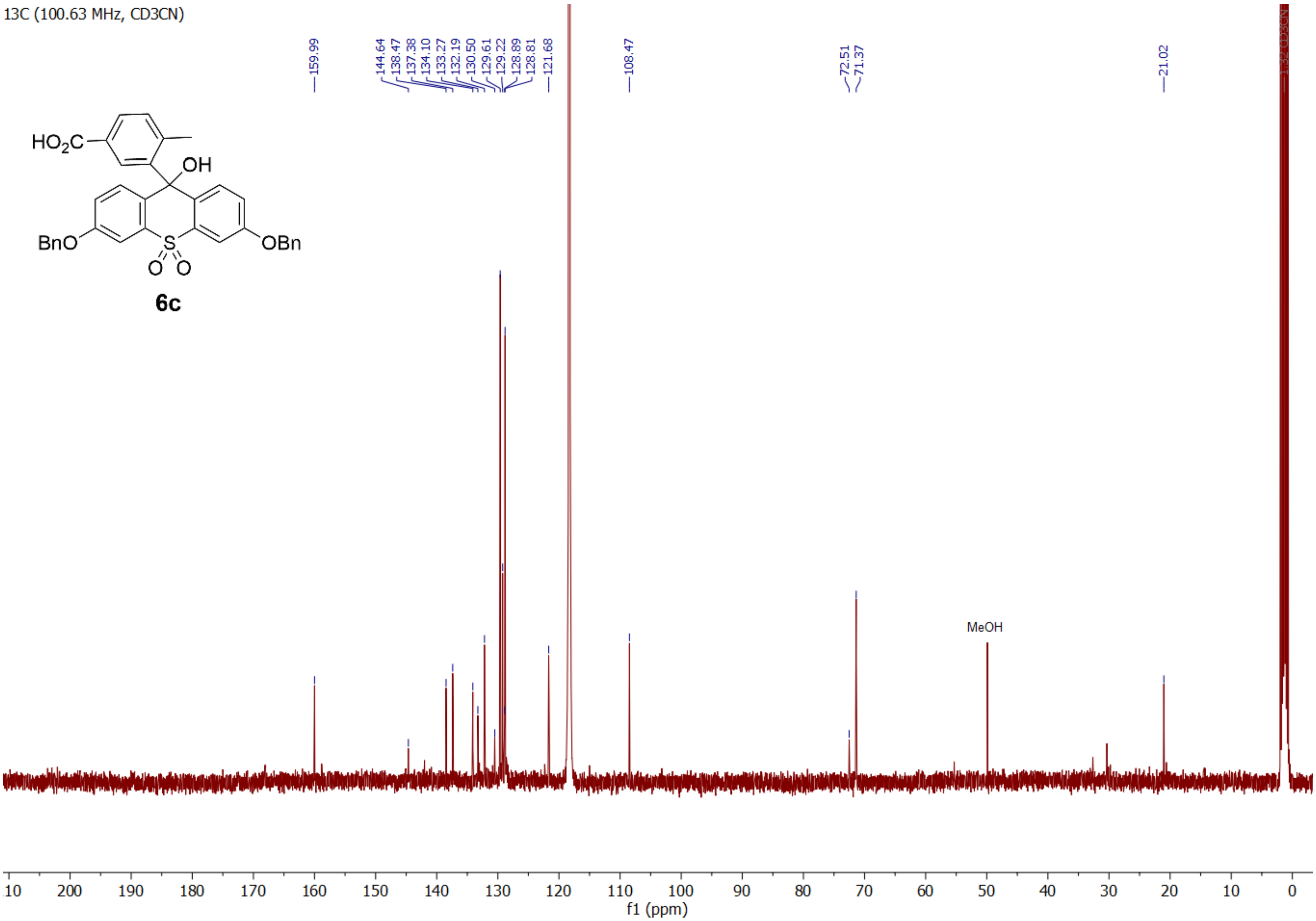
13C (100.63 MHz, CD3CN)
159.99
144.64
138.47
137.38
134.10
133.27
132.19
130.50
129.61
129.22
128.89
128.81
121.68
108.47
72.51
71.37
21.02
1.32 CD3CN
HO2C
OH
BnO
OBn
S
O O
6c
MeOH
f1 (ppm)

1H (400.15 MHz, CDCl3)

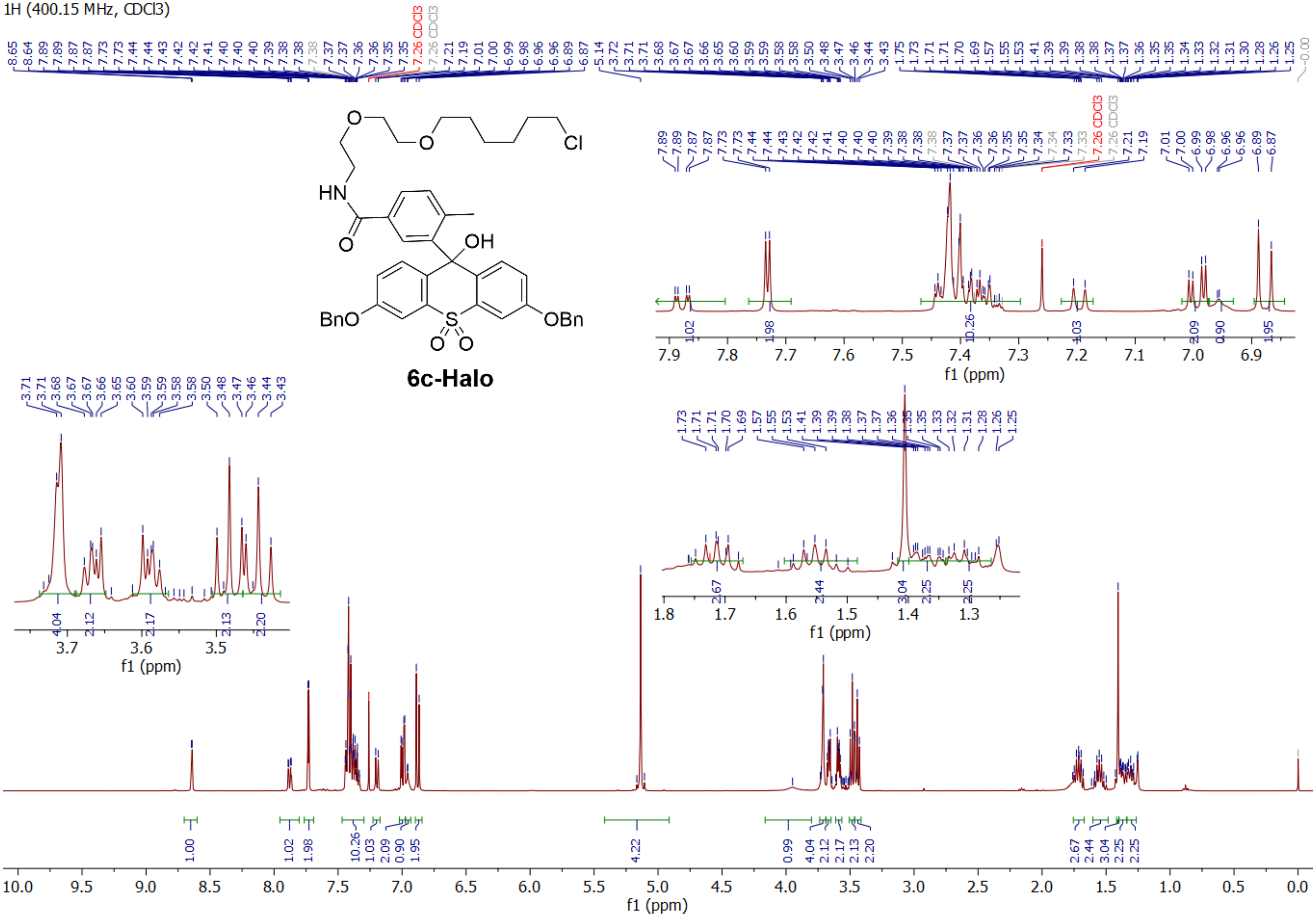

13C (100.63 MHz, CDCl3)

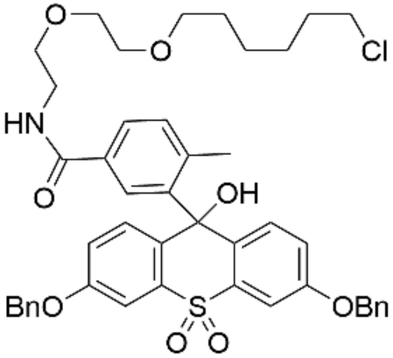


**6c-Halo**

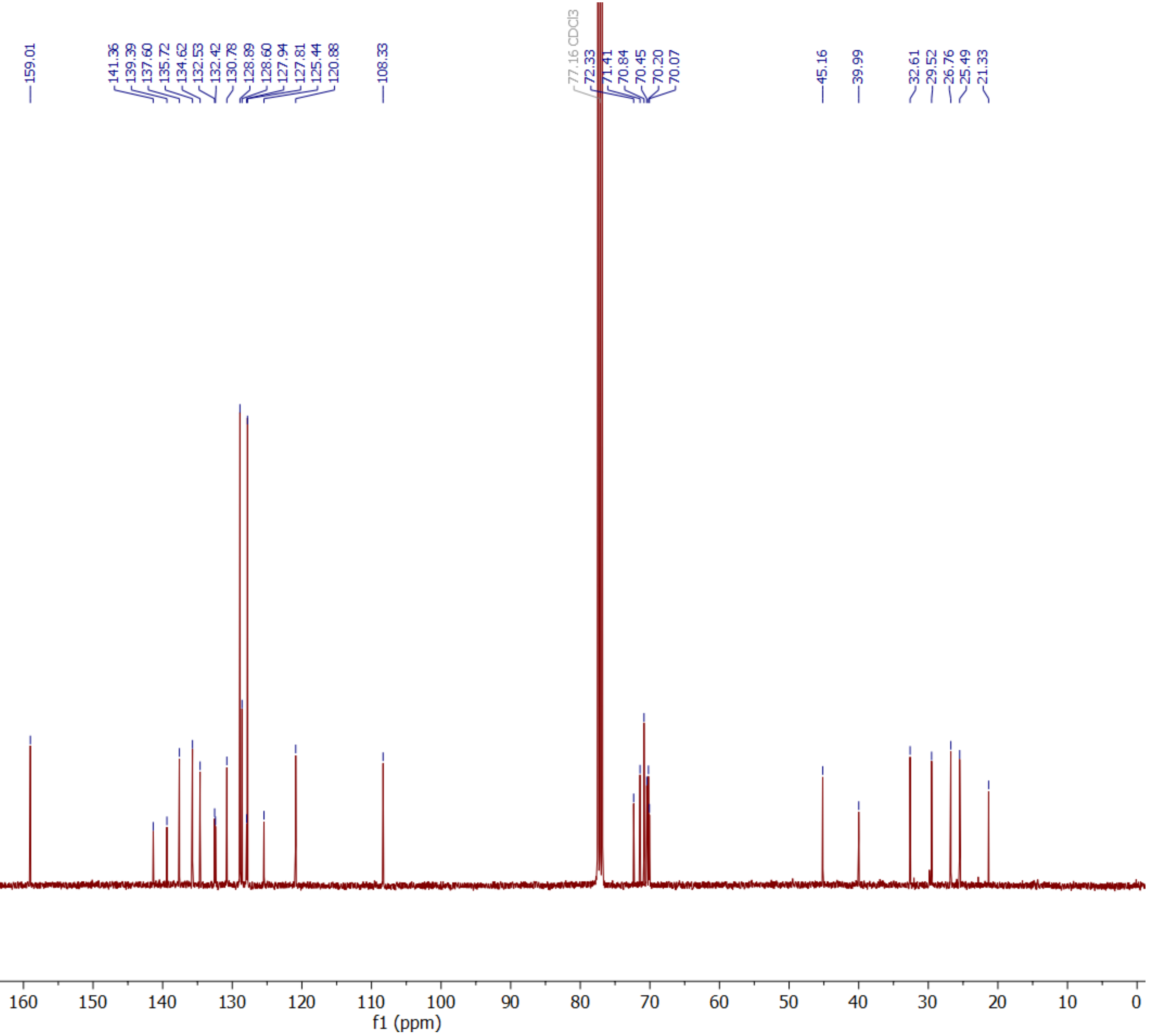

1H (400.15 MHz, CD3CN)

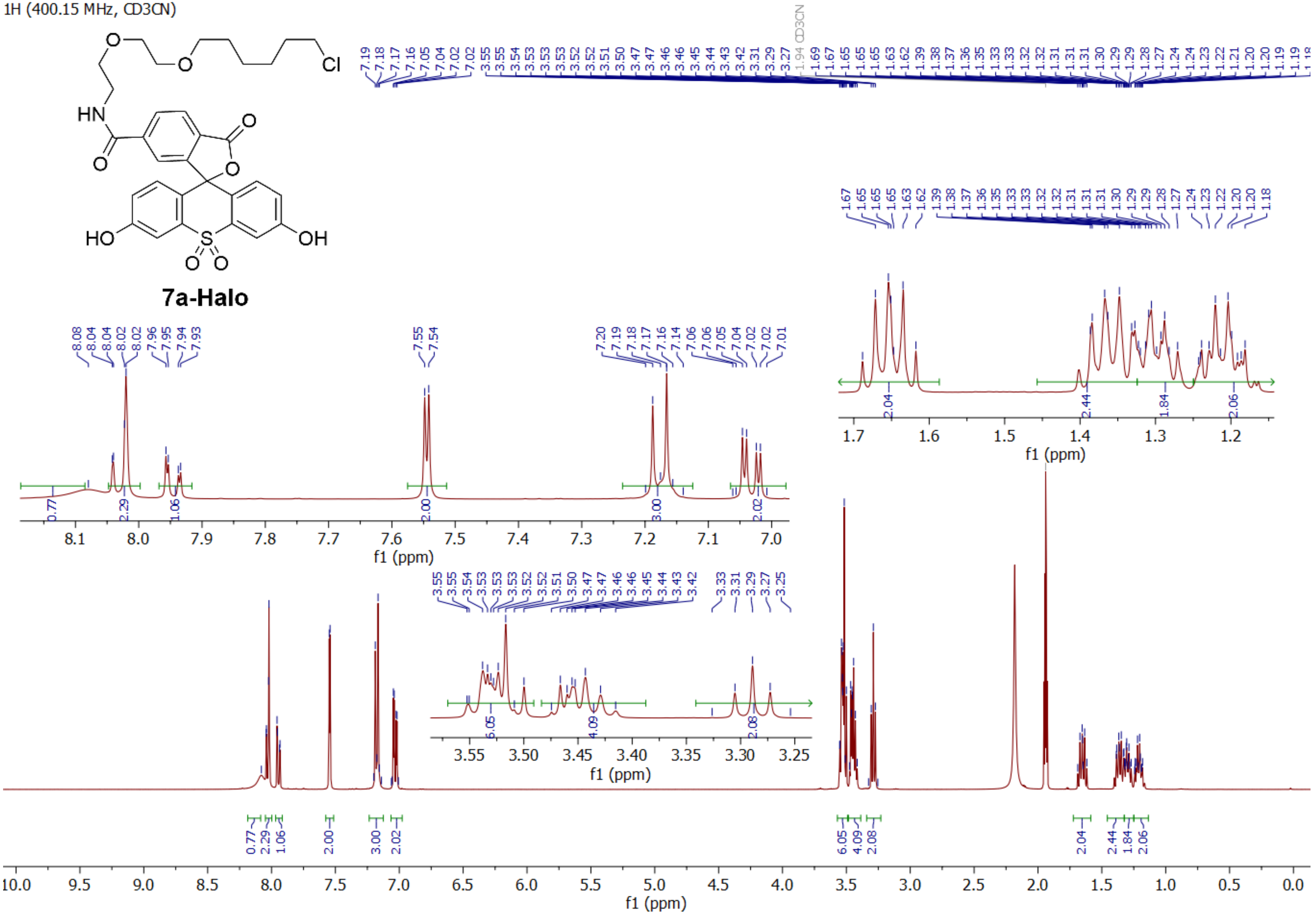

7a-Halo

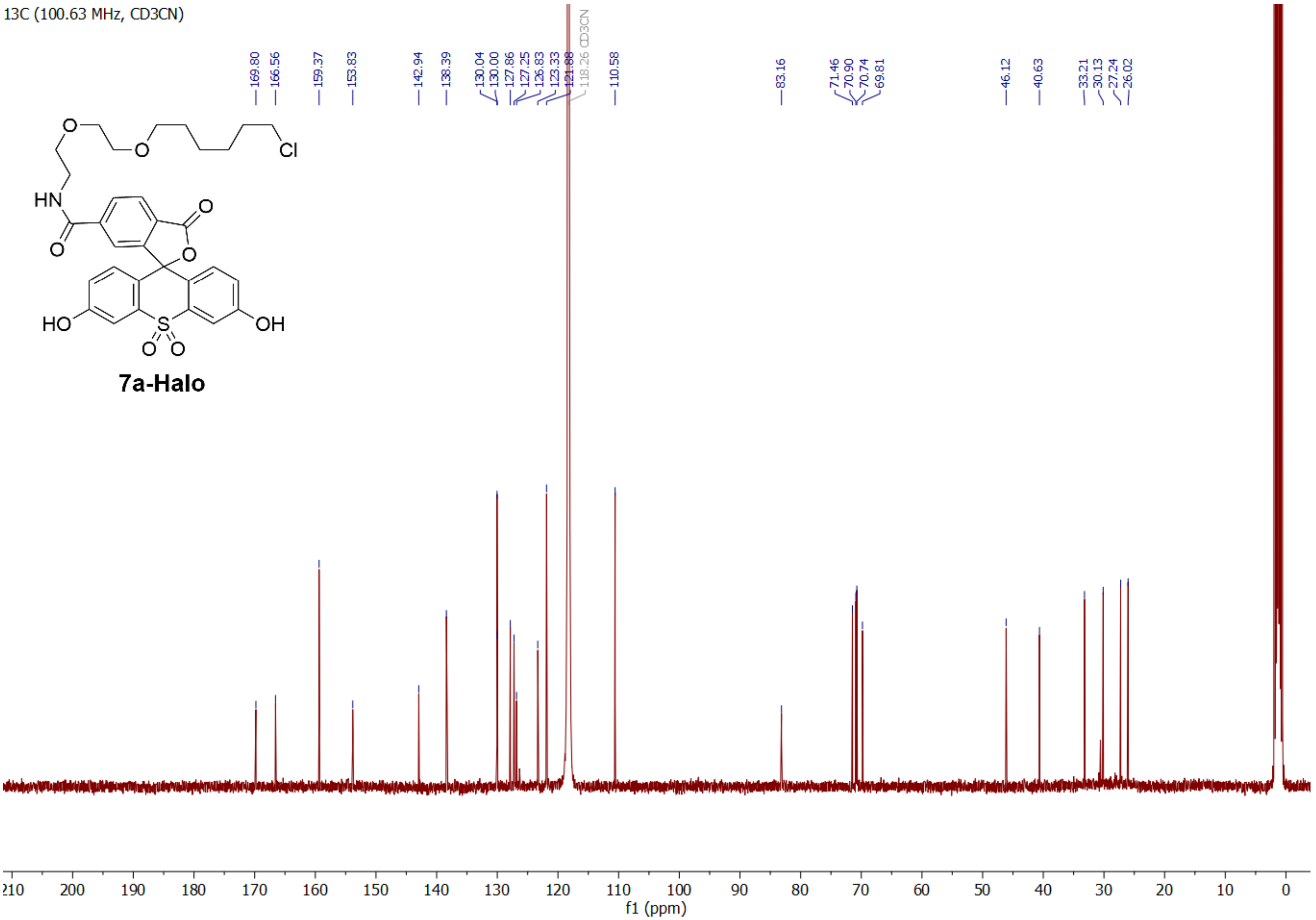

13C (100.63 MHz, CD3CN)
169.80
166.56
159.37
153.83
142.94
138.39
130.04
130.00
127.86
127.25
126.83
123.33
121.88
118.26 CD3CN
110.58
83.16
71.46
70.90
70.74
69.81
46.12
40.63
33.21
30.13
27.24
26.02
O
O
Cl
HN
O
O
O
HO
OH
S
O O
7a-Halo
210 200 190 180 170 160 150 140 130 120 110 100 90 80 70 60 50 40 30 20 10 0
f1 (ppm)

1H (400.15 MHz, CD3CN)

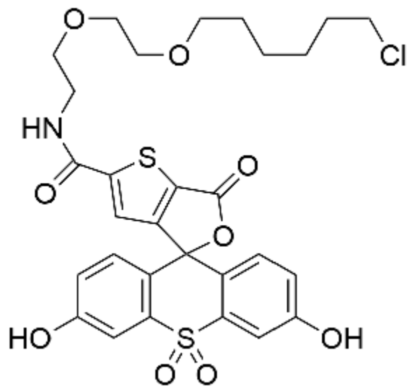

**7b-Halo**

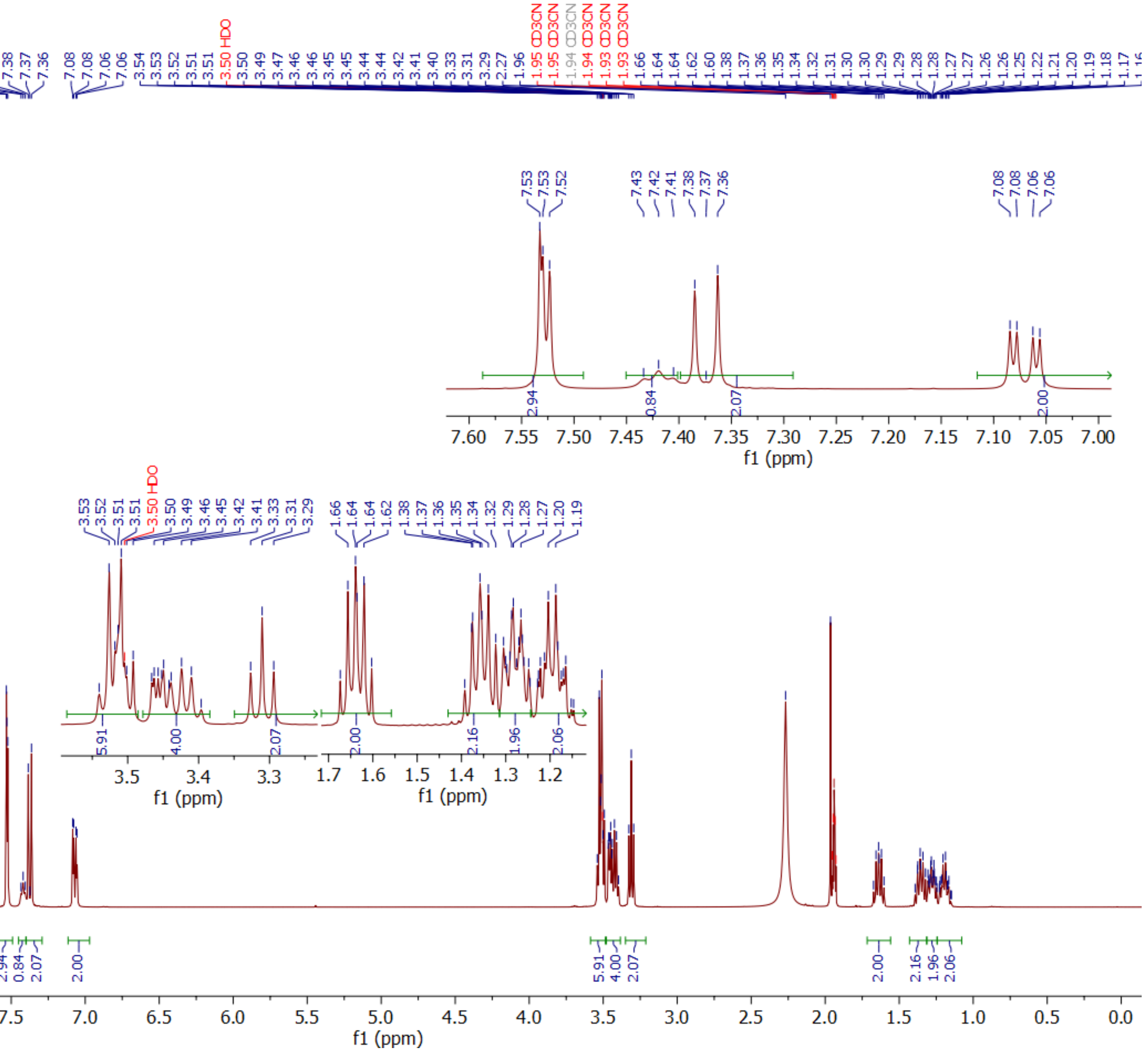

13C (100.63 MHz, CD3CN)

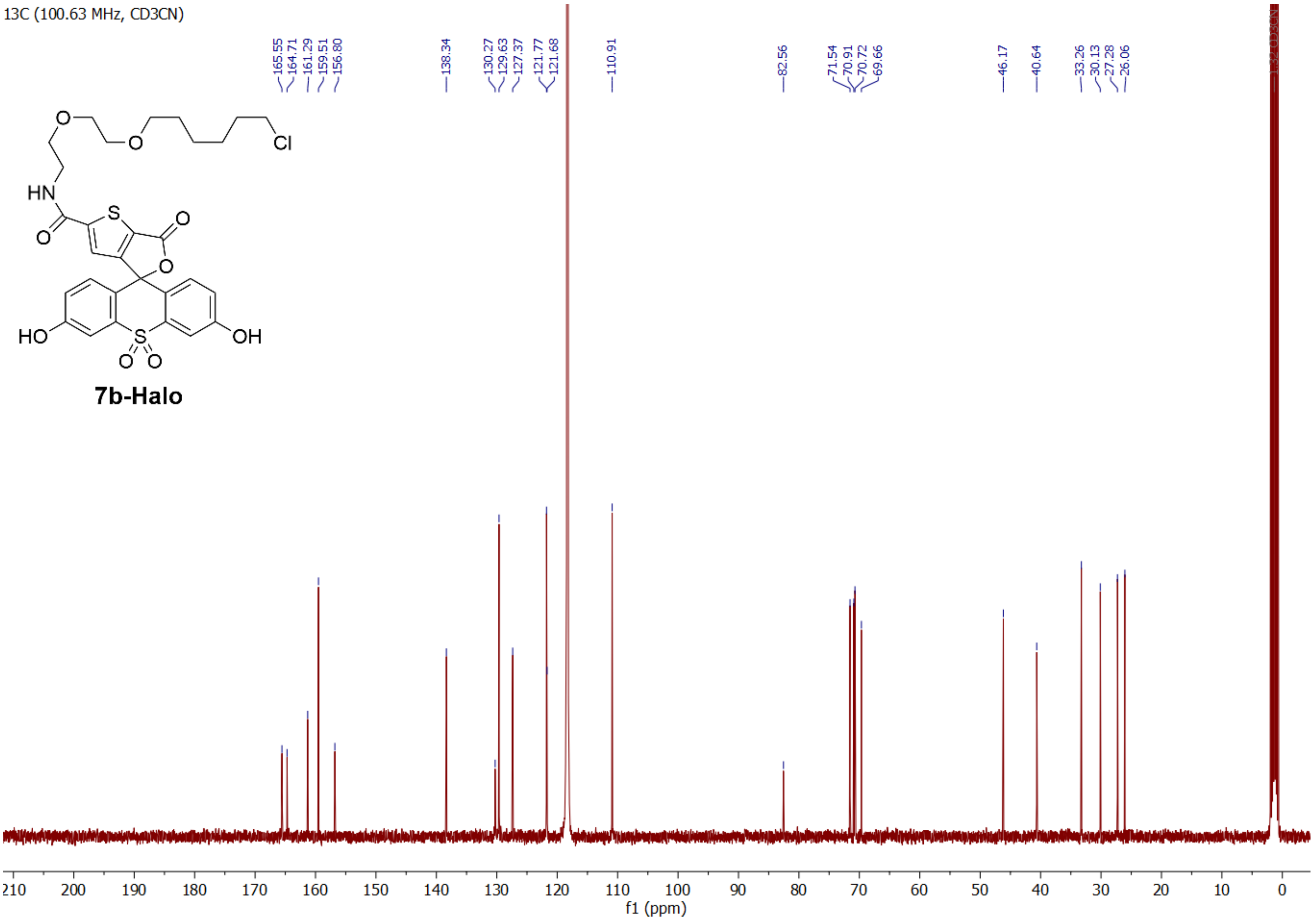

1H (400.15 MHz, CDCl3:TFA-d 10:1 (v/v))

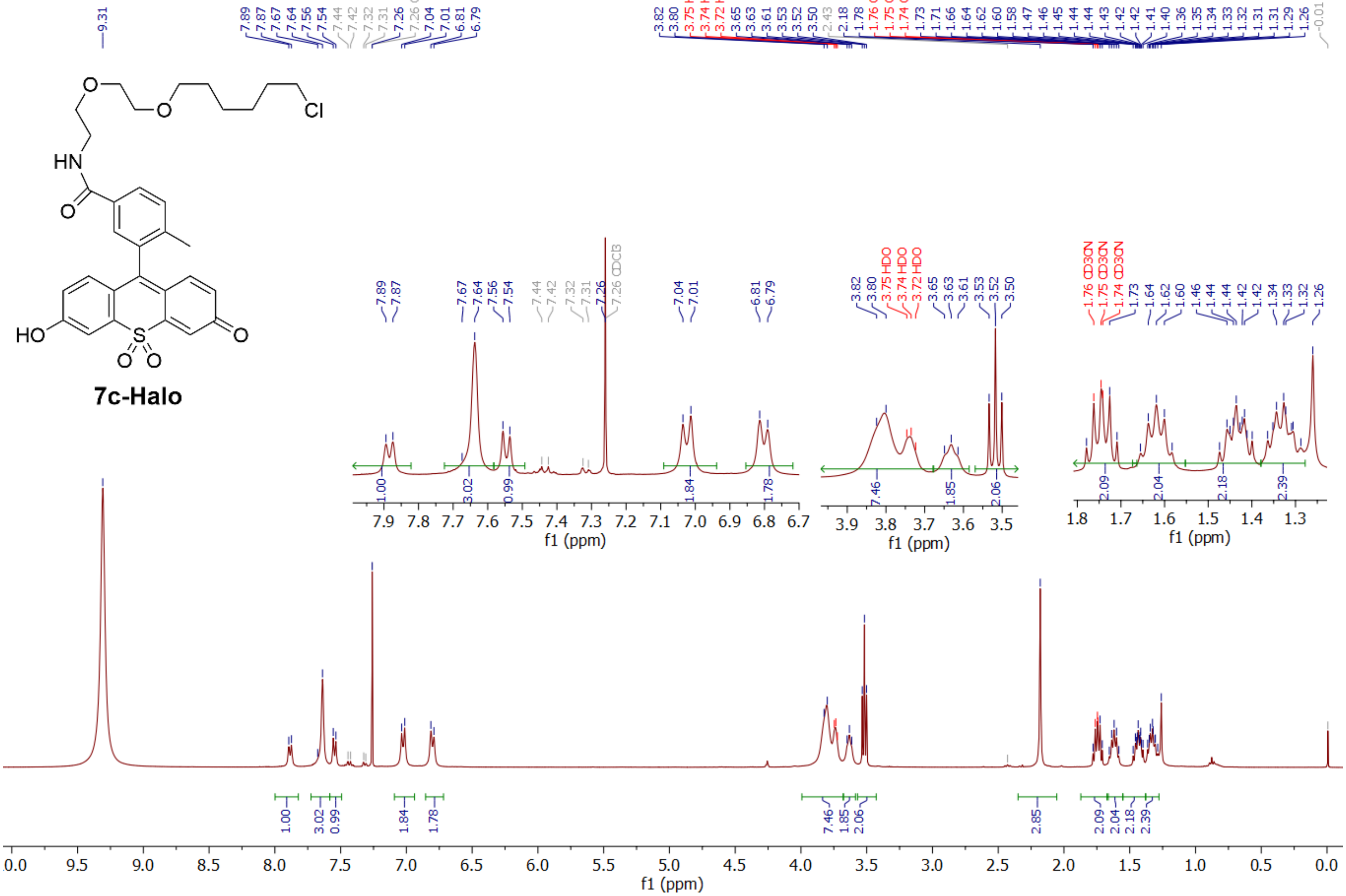


**7c-Halo**

13C (100.63 MHz, CDCl3:TFA-d 10:1 (v/v))

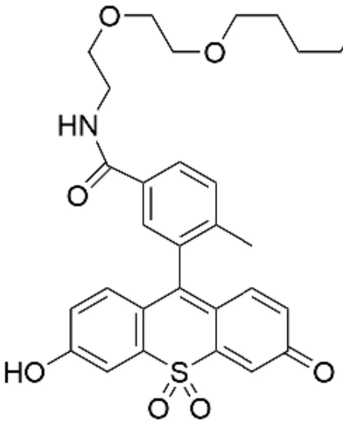

7c-Halo

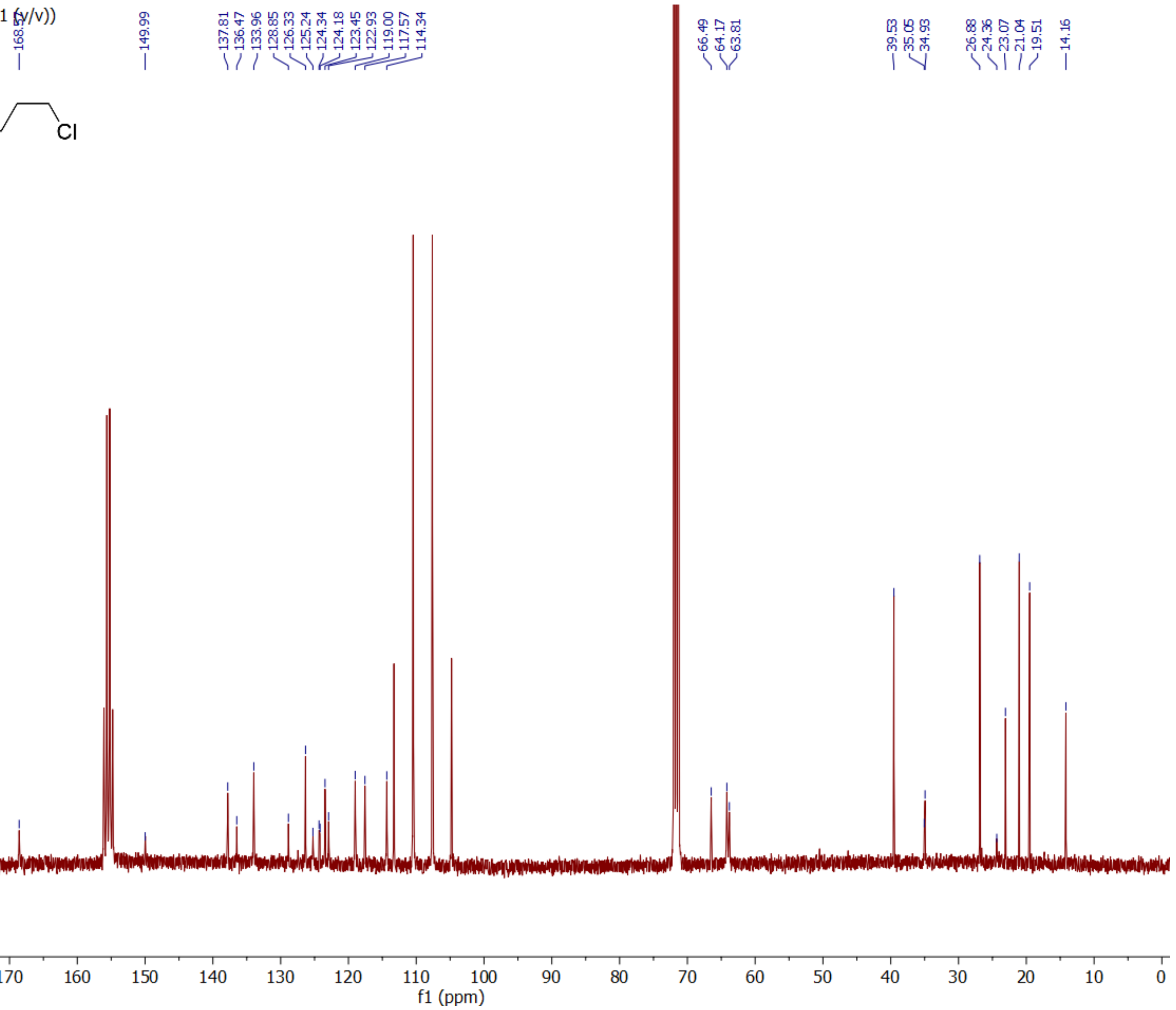

Supplement: Supplementary file 2 — ol3c04300_si_002.pdf [file ol3c04300_si_002.pdf]
